# Connectomic reconstruction predicts visual features used for navigation

**DOI:** 10.1038/s41586-024-07967-z

**Published:** 2024-10-02

**Authors:** Dustin Garner, Emil Kind, Jennifer Yuet Ha Lai, Aljoscha Nern, Arthur Zhao, Lucy Houghton, Gizem Sancer, Tanya Wolff, Gerald M. Rubin, Mathias F. Wernet, Sung Soo Kim

**Affiliations:** 1 Molecular, Cellular, and Developmental Biology, University of California Santa Barbara, Santa Barbara, CA USA; 2 Department of Biology, Freie Universität Berlin, Berlin, Germany; 3 Janelia Research Campus, Howard Hughes Medical Institute, Ashburn, VA USA; 4 Department of Neuroscience, Yale University, New Haven, CT USA; 5 Neuroscience Research Institute, University of California Santa Barbara, Santa Barbara, CA USA; 6 Dynamical Neuroscience, University of California Santa Barbara, Santa Barbara, CA USA

**Keywords:** Neural circuits, Visual system

## Abstract

Many animals use visual information to navigate^1–4^, but how such information is encoded and integrated by the navigation system remains incompletely understood. In *Drosophila melanogaster*, EPG neurons in the central complex compute the heading direction^5^ by integrating visual input from ER neurons^6–12^, which are part of the anterior visual pathway (AVP)^10,13–16^. Here we densely reconstruct all neurons in the AVP using electron-microscopy data^17^. The AVP comprises four neuropils, sequentially linked by three major classes of neurons: MeTu neurons^10,14,15^, which connect the medulla in the optic lobe to the small unit of the anterior optic tubercle (AOTUsu) in the central brain; TuBu neurons^9,16^, which connect the AOTUsu to the bulb neuropil; and ER neurons^6–12^, which connect the bulb to the EPG neurons. On the basis of morphologies, connectivity between neural classes and the locations of synapses, we identify distinct information channels that originate from four types of MeTu neurons, and we further divide these into ten subtypes according to the presynaptic connections in the medulla and the postsynaptic connections in the AOTUsu. Using the connectivity of the entire AVP and the dendritic fields of the MeTu neurons in the optic lobes, we infer potential visual features and the visual area from which any ER neuron receives input. We confirm some of these predictions physiologically. These results provide a strong foundation for understanding how distinct sensory features can be extracted and transformed across multiple processing stages to construct higher-order cognitive representations.

## Abstract (web summary)

Electron-microscopy data are used to reconstruct the neurons that make up the anterior visual pathway in the *Drosophila* brain, providing insight into how visual features are encoded to guide navigation.

## Main

The AVP encodes visual features that are essential for navigation, potentially including landmarks, intensity gradients, colour, celestial bodies and skylight polarization^1,5,11,18–22^. Considering its fundamental role in navigation, it is not surprising that this anatomical structure is largely conserved across most known insect species^10,23–25^. It is likely that deep similarities exist across species in the basic logic of visual feature extraction for navigation^19,23–28^. However, despite many studies of the AVP across species, researchers’ knowledge about the AVP neurons has been fragmented by the lack of a complete circuit diagram to frame systematic investigations. Here we aim to provide such a framework in flies, and we anticipate that this will also be invaluable for designing and prioritizing physiological experiments to interrogate the AVP in other species.

**Fig. 1:**
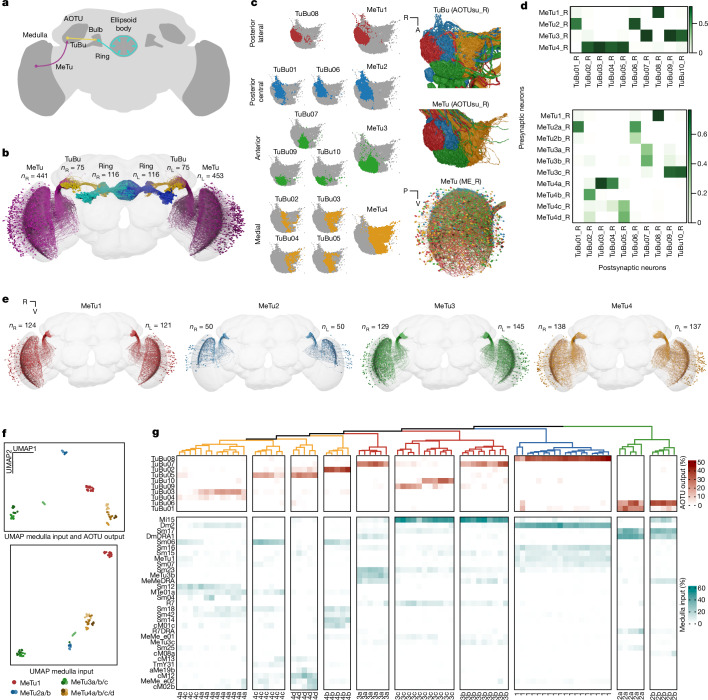
Identification and classification of MeTu neurons in the AVP. **a**, Diagram of the *D. melanogaster* central brain, emphasizing the AVP. Important regions are darker grey, including the medulla, AOTU, bulb and ellipsoid body (the former three have counterparts in both hemispheres). The three crucial neurons of the AVP are MeTu (purple), TuBu (yellow) and ER (cyan). **b**, All MeTu (*n* = 453 left; *n* = 441 right), TuBu (*n* = 75 left; *n* = 75 right) and visual ER (*n* = 116 left; *n* = 116 right) neurons. **c**, Left, synapse plots of TuBu (left) and MeTu (middle) neurons in the posterior lateral (red), posterior central (blue), anterior (green) and medial (yellow) region of the AOTUsu. Right, renders of TuBu in the AOTUsu_R (top) and MeTu in the AOTUsu_R (middle) and ME_R (bottom) with the same regional colours as the synapse plots on the left. Coordinates: A, anterior; R, lateral side of the right hemisphere; P, posterior; V, ventral. **d**, Synaptic weight matrices of MeTu type (top) or MeTu subtype (bottom) to TuBu type connectivity (right hemisphere). **e**, All neurons of types MeTu1 (far left), MeTu2 (left middle), MeTu3 (right middle) and MeTu4 (far right). **f**, UMAPs of all MeTu neurons with identified upstream partners on the basis of the synaptic weight of both the top five medulla input neuron types and AOTU output neuron types (top), or just the top five medulla input types (bottom) (see Methods for details). Groupings are generally consistent with MeTu1–MeTu4 groups in the main text, except MeTu3a neurons, which are closer to MeTu2 neurons (because of the similar polarization input) than other MeTu3 neurons. **g**, Synaptic weight matrix of all MeTu neurons with identified upstream partners (columns) and their AOTU output partners (red top rows) and top five medulla input types (teal bottom rows). Dendrogram branches and column labels are colour-coded according to MeTu. See Supplementary Data 1h,i for analyses with entire MeTu neurons.

**Extended Data Fig. 1:**
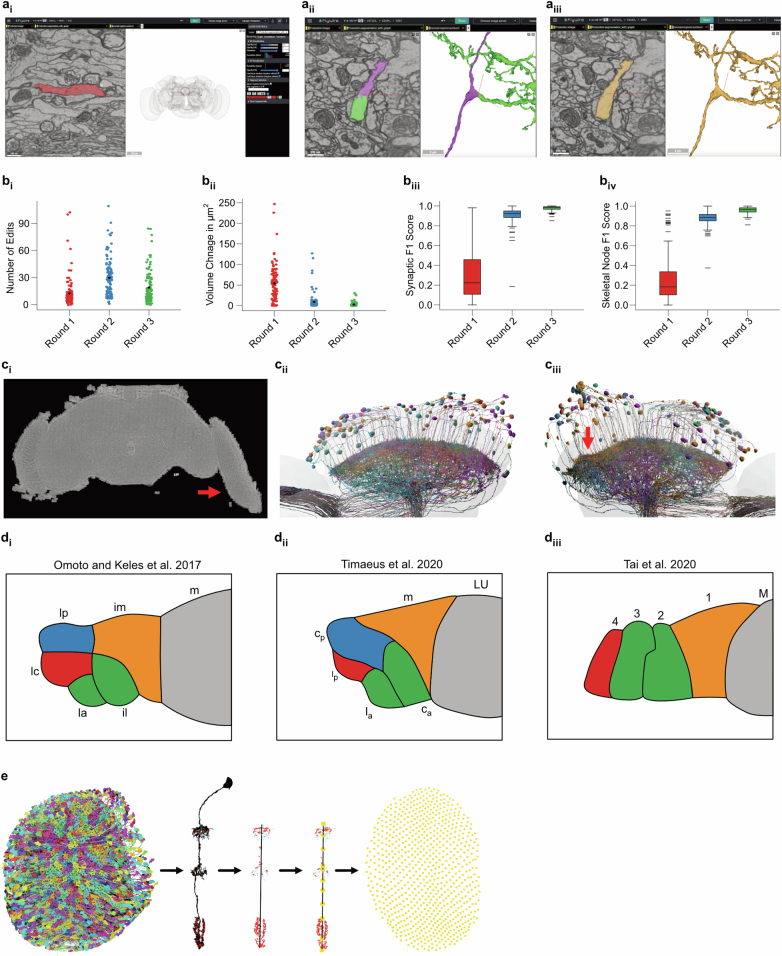
Methods of analysis of the AVP and AOTUsu. **a**, Diagrams of FlyWire. Image of the FlyWire interface with the EM volume on the left and neuropils on the right (**a**_**i**_). An example of proofreading/, with images before (**a**_**ii**_) and after (**a**_**iii**_) a merge of two neuron fragments. **b**, Quality control through three rounds of proofreading among 113 MeTu neurons of the right hemisphere. The total number of edits per neuron per round (**b**_**i**_), the change in neuronal volume after each round (**b**_**ii**_) and the F1 scores calculated between rounds for synapse counts (**b**_**iii**_) and skeletal nodes (**b**_**iv**_). All pair-wise test in each plot was significant (p < 0.0001, Wilcoxon rank sum test). **c**_**i–iii**_, EM data quality of the left and right optic lobe. **c**_**i**_, EM slice of the fly brain, note the partially detached lamina (arrow) on the left optic lobe. **c**_**ii**_,**c**_**iii**_, MeTu1 neurons of the right and left optic lobe, respectively, viewed from the dorsal side, note the uneven image alignment on the posterior side (arrow) of the left optic lobe (**c**_**iii**_). **d**_**i–iii**_, Comparison of different illustrations of the AOTUsu subregion (see section ‘AOTUsu subdivision in comparison with previous studies’ in the Methods for more details)^14,15,39^. **e**, Process of defining medulla columns and layers from all Mi1 neurons, a unicolumnar cell type, shown for the right optic lobe. From left to right: render of all Mi1 neurons of the right optic lobe (n = 796), a single Mi1 neuron with pre- (red) and postsynaptic (cyan) sides, distal–proximal axis of a column is given by PC1 of a PCA on all synaptic sides of the corresponding Mi1 neuron, defining layer markers based on the upper and lower bound of the distal–proximal axis, m6 layer marker of all columns.

We identified all neurons (Fig. 1) and their connectivity (Supplementary Data 1) in the AVP, using a publicly available electron-microscopy (EM) dataset (full adult female brain, FAFB) that contains the entire adult fly brain, with FlyWire, an AI-assisted collaborative platform^17,29,30^ (Extended Data Fig. 1). MeTu neurons, the first stage of the AVP, leave the medulla, the largest neuropil in the fly visual system^31–36^. The axons of MeTu neurons innervate the AOTUsu^10,14,16,32,37,38^ (Fig. 1a,b), where the information is further processed by the TuBu neurons that connect the AOTUsu to the bulb^10,11,38^ (Fig. 1a–d and Supplementary Data 1f_i,ii_). There are ten classes of TuBu neurons (Fig. 1c), each synapsing onto the dendrites of distinct classes of ER neurons (Fig. 1a,b and Supplementary Data 1f_iii,iv_). ER neurons then send ring-like processes to a donut-shaped structure, the ellipsoid body^16,39,40^ (Fig. 1a), where they together form a complex recurrent neural network^5,41,42^ (Fig. 1b and Supplementary Data 1e). Finally, the visual information from the AVP—along with other sensory modalities^43^—is compiled to compute the heading direction by EPG neurons^5^ that share many similarities with mammalian ‘head direction’ cells^5^.

**Extended Data Fig. 2:**
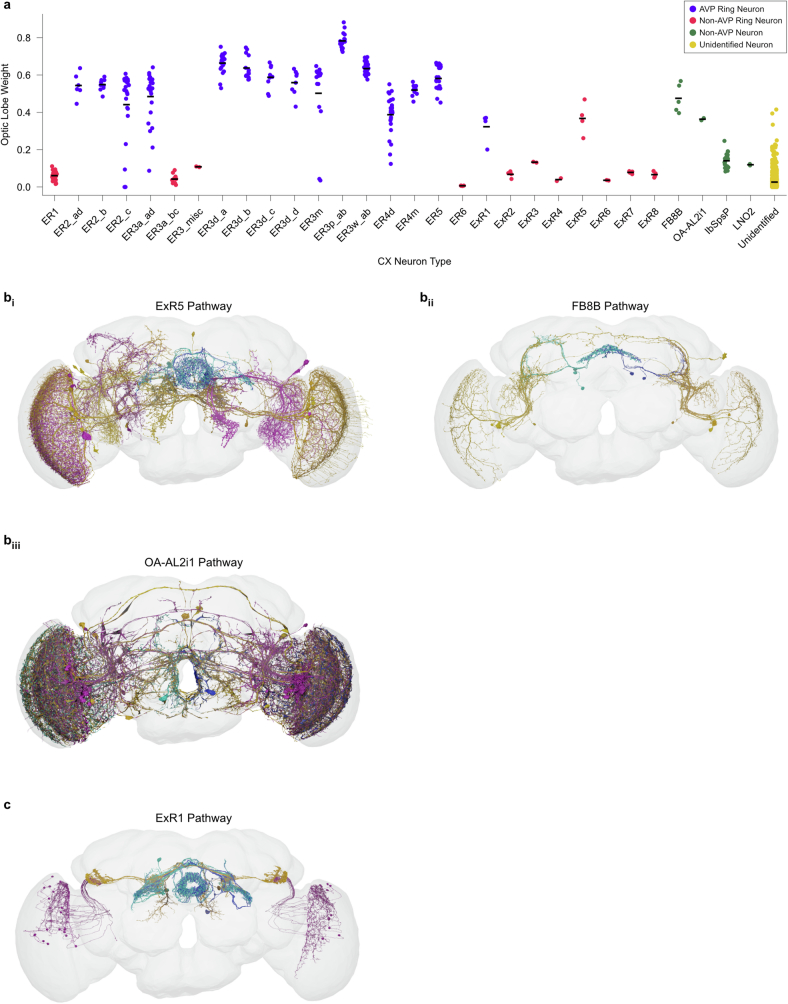
Alternative pathways from the optic lobe to the central complex. **a**, Strip plot that includes all neurons providing input to the central complex. Those of known types have been labelled (blue dots: ER neurons in the AVP, red dots: non-AVP ER neurons, green dots: non-EB neurons), and others (yellow dots) are labelled “Unidentified”. The y-axis represents the total synaptic weight from neurons that contain dendrites within the optic lobe that are at most two neurons upstream of the target neuron. The relative synaptic contribution of visual information, calculated from synapse counts, is less than 10% for most neurons outside the AVP, much less than 40% of those in the AVP. Further, only 11 neurons (four ExR5, five FB8B, and two OA-AL2i1) outside the AVP showed significant synaptic contribution to the central complex, compared to 236 neurons in the AVP. ER1: n = 28, ER2_ad: n = 6, ER2_b: n = 10, ER2_c: n = 26, ER3_misc: n = 2, ER3a_ad: n = 24, ER3a_bc: n = 11, ER3d_a: n = 21, ER3d_b: n = 14, ER3d_c: n = 9, ER3d_d: n = 8, ER3m: n = 15, ER3p_ab: n = 17, ER3w_ab: n = 26, ER4d: n = 24, ER4m: n = 11, ER5: n = 21, ER6: n = 4, ExR1: n = 4, ExR2: n = 4, ExR3: n = 2, ExR4: n = 2, ExR5: n = 4, ExR6: n = 2, ExR7: n = 4, ExR8: n = 4, FB8B: n = 5, IbSpsP: n = 24, LNO2: n = 2, OA-AL2i1: n = 2, Unidentified: n = 1261. **b**_**i–iii**_, Renders of pathways from the optic lobe to three types of neurons that convey significant visual information from outside the AVP: ExR5 (**b**_**i**_), FB8B (**b**_**ii**_) and OA-AL2i1 (**b**_**iii**_). Only ExR5 neurons are in the EB and may provide visual information to EPG neurons. With n = 4, however, they are unlikely to provide retinotopically organized visual information. These renders include the relevant central complex neurons (blue for right, cyan for left), neurons one layer upstream (yellow), and optic lobe neurons two layers upstream (pink). Note the the lack retinotopy and their diffuse connectivity patterns. **c**, Renders of the AVP pathways to ExR1.

Our survey of the entire brain^29^ showed that visual information originating from optic lobe structures outside the AVP (the lobula and the lobula plate) constitutes minimal input to EPG neurons (Extended Data Fig. 2). Thus, we focused our analyses on the medulla-originating AVP. Furthermore, although previous anatomical studies of various types of MeTu neuron have generally agreed at the macroscale^10,14,15^ (Extended Data Fig. 1d), the insufficient resolution of light microscopy has resulted in considerable inconsistencies in grouping MeTu types and predicting their connectivity towards the central complex (see Methods for a more detailed discussion on the differences). Hence, we sought to provide a comprehensive view of this pathway in synapse-level detail. Finally, although the connectivity from TuBu to ER to EPG neurons has been studied at synaptic resolution^16^ (a dataset we refer to here as hemibrain data), this dataset contains only one hemisphere and lacks upstream medulla neuropils and photoreceptor terminals. Therefore, we sought to reconstruct the entire AVP in both hemispheres (Supplementary Data 1) to provide a solid foundation for understanding how brains vary across hemispheres and across animals, as well as which visual features are extracted in the AVP.

## Reconstruction of the AVP

We densely proofread all MeTu neurons (453 on the left hemisphere, 441 on the right; Fig. 1a,b and Supplementary Data 2), TuBu neurons (75 on the left, 75 on the right), and ER neurons (116 on the left, 116 on the right). We further reconstructed all medulla-intrinsic Mi1 neurons in the medulla (782 on the left and 792 on the right^31^) from both hemispheres (Extended Data Fig. 1e) to map the exact locations of all reconstructed neurons relative to the retinotopic columns in the medulla. To assess our proofreading quality^29^, we selected 113 (of 441) MeTu neurons from the right hemisphere and performed multiple rounds of proofreading (Extended Data Fig. 1b). We found that—after the first round—any additional volume reconstructed in each round markedly decreased and there were no changes in the main backbone (Extended Data Fig. 1b_ii_). Moreover, all MeTu neurons (894 neurons—both hemispheres and including neurons with a single round of proofreading) shared stereotypical arborization patterns in the medulla. Therefore, we were confident that our reconstruction quality of the 894 MeTu neurons was sufficiently accurate for categorization and morphological and connectivity analyses.

We focused on the detailed connectivity of MeTu neurons because the logic of their connections between optic lobes and the central brain was missing in previous studies^16^. We included results from both hemispheres in most analyses, but, where indicated, some detailed analyses were restricted to the right hemisphere because the left hemisphere had an incomplete lamina and (minor) EM image alignment issues^17,29,44^.

## AOTUsu anatomy defines four MeTu classes

**Extended Data Fig. 3:**
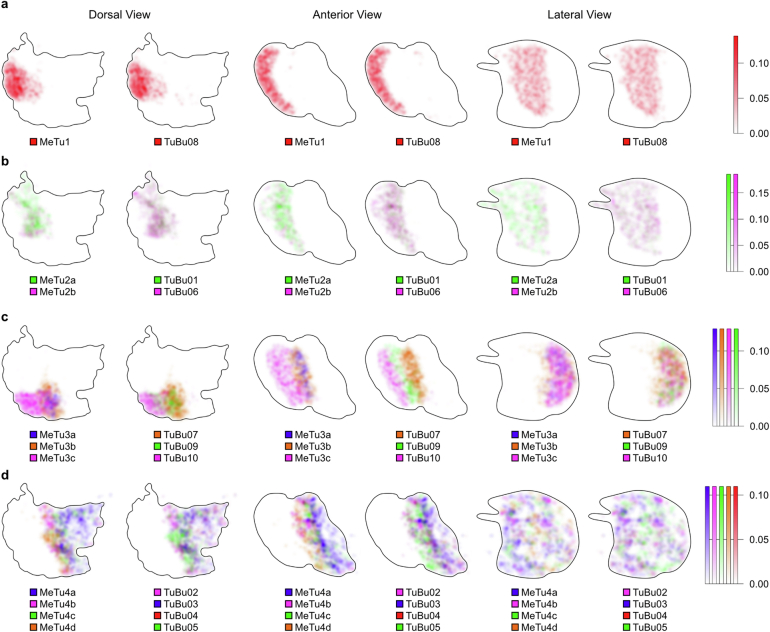
Synapse density maps of MeTu-to-TuBu connections. **a**–**d**, Synapse density maps in the AOTUsu_R from the dorsal, anterior, and lateral perspectives. All perspectives have been rotated 30° with respect to the anterior–posterior axis, and synapse densities were blurred with a Gaussian filter with a sigma value of 10. Figures include MeTu1 (**a**), MeTu2a-b (**b**), MeTu3a-c (**c**), and MeTu4a-d (**d**) to their relevant downstream TuBu partners. **a**, The AOTUsu_PL comprises the outermost lateral volume of the AOTUsu, facing the posterior side (Figs. 1c_i_ and 2t). All MeTu axons and TuBu dendrites found in this area arborize solely there and do not extend processes to other regions of the AOTUsu. We therefore designated all MeTu neurons arborizing in AOTUsu_PL as MeTu1 (Fig. 1c,e). All MeTu1 neurons form synapses with TuBu08 neurons (Fig. 1d and Supplementary Data 1f_i-ii_). The dendrites of any given TuBu08 neuron partially overlap with those of neighbouring TuBu08 neurons (Fig. 2t). As a population, the dendrites of TuBu08 neurons therefore roughly form a one-dimensional line along the dorsal–ventral axis, with a small positional variation along the medial–lateral axis (Fig. 2t). The AOTUsu_PL contains only one more cell type, being sparsely innervated by all four AOTU046 neurons (Extended Data Fig. 4b), which may provide motor context from superior posterior slope (SPS) and also potentially mediate bilateral communication between hemispheres (Extended Data Fig. 4 and Supplementary Data 1f). **b**, The AOTUsu_PC is located directly medial to AOTUsu_PL (Fig. 1c and Extended Data Fig. 5l). We designated all MeTu neurons that innervate AOTUsu_PC as MeTu2. Their dendrites in medulla were limited to the DRA, where input from photoreceptors that are sensitive to skylight polarization is processed ^21,74^. MeTu2 neurons make synapses onto TuBu01 and TuBu06 (Fig. 1d and Supplementary Data 1f_i-ii_). The dendrites of individual TuBu01 neurons do not overlap with neighbouring neurons of the same type (Extended Data Fig. 5l). As a population, TuBu01 and TuBu06 neurons form a one-dimensional line along the dorsal–ventral axis (Extended Data Fig. 5l). AOTUsu_PC contains two more types of neurons TuTuB_a and TuTuB_b, both of which interconnect the two hemispheres (Extended Data Fig. 4a,d). **c**, The AOTUsu_A is located in front of both AOTUsu_PL and AOTUsu_PC (Fig. 1c and Extended Data Fig. 6m). We designated all MeTu neurons that innervate AOTUsu_A as MeTu3. MeTu3 neurons synapse onto TuBu07, TuBu09, and TuBu10 neurons. These TuBu neurons are located in the medial, central, and lateral portions of the AOTUsu_A, respectively (Extended Data Fig. 6m). Of these three TuBu types, dendrites of the same type partially overlap each other but do not overlap with dendrites of other types (Fig. 1c and Extended Data Fig. 3c). In addition, they do not form clear one-dimensional lines along the dorsal–ventral axis as do the TuBu neurons in the AOTUsu_PL/PC. Finally, the entire anterior area is covered by dendritic and axonal processes of TuTuB_a (Extended Data Fig. 4d), and innervated by axonal boutons of AOTU046 (Extended Data Fig. 4e) exclusively in the TuBu09 location (Extended Data Fig. 4a). **d**, The AOTUsu_M is adjacent to both AOTUsu_PC and AOTUsu_A (Fig. 1c and Extended Data Fig. 8l). We designated all MeTu neurons that innervate AOTUsu_M as MeTu4. Their axonal boutons tile the entire volume with varying densities and spans. MeTu4 neurons synapse onto TuBu02, TuBu03, TuBu04, and TuBu05 neurons. TuBu02 dendrites are wide along the anterior–posterior axis and have thin dendritic clumps (Extended Data Fig. 8l). Dendrites of TuBu03 neurons form a rough one-dimensional line (Extended Data Fig. 8l). TuBu04 dendrites are sparse but cover an exceptionally wide area, with some filling the entire volume. As a population, they are not linearly arranged (Extended Data Fig. 8l). Dendrites of TuBu05 branch widely along the anterior–posterior axis but are narrower along the dorsal–ventral axis, forming a rough one-dimensional line (Extended Data Fig. 8l). The AOTUsu_M has a thin two-layer structure: TuBu02 and TuBu05 both cluster along the border between the AOTUsu_M and the other AOTUsu areas, while TuBu03 and TuBu04 cluster more medially. Finally, only AOTU046 neurons, but not TuTuB_a/b neurons, innervate AOTUsu_M, thus potentially conveying motor information from SPS (Extended Data Fig. 4b).

The AOTUsu is innervated by four types of processes: the axons of MeTu neurons; the dendrites of downstream TuBu neurons (Fig. 1c); and the synaptic terminals of bilaterally projecting AOTU046 and tubercle-to-tubercle (TuTu) neurons^16^. Drawing on the axonal arborization pattern of MeTu and the dendritic arborization pattern of TuBu, we divided the AOTUsu into four major subregions: posterior lateral (AOTUsu_PL), posterior central (AOTUsu_PC), anterior (AOTUsu_A) and medial (AOTUsu_M; Fig. 1c, left). These anatomical divisions led us to categorize MeTu neurons into four major classes (MeTu1–MeTu4; Fig. 1c–e). Downstream TuBu neurons were categorized into ten types, consistent with previous works (Fig. 1c, left; TuBu1–TuBu10; numbering follows the nomenclature of a previous study^16^). See Extended Data Fig. 3 for a detailed anatomical description of these four areas and the neurons that innervate them.

## Connectivity reveals ten MeTu subtypes

Within each MeTu class except MeTu1, we observed discrete morphologies and anatomical innervations, suggesting multiple channels for visual features. To systematically categorize all possible MeTu neuron subtypes, we focused our analysis on the 5 strongest synaptic partner types; this resulted in 28 types of upstream neuron. Applying a nonlinear dimensionality reduction analysis (uniform manifold approximation and projection; UMAP) based on the connectivity in the medulla but not in the AOTUsu revealed four major patterns of presynaptic inputs, mostly consistent with the four major MeTu classes defined in the previous section (Fig. 1f and Supplementary Data 1h). We also performed categorization analyses (Fig. 1g and Supplementary Data 1i) and found ten subtypes. Comparing the expression pattern of genetic driver lines (see Methods) with EM data further corroborated our subtyping of MeTu neurons.

## MeTu1 neurons form a homogeneous group

**Fig. 2:**
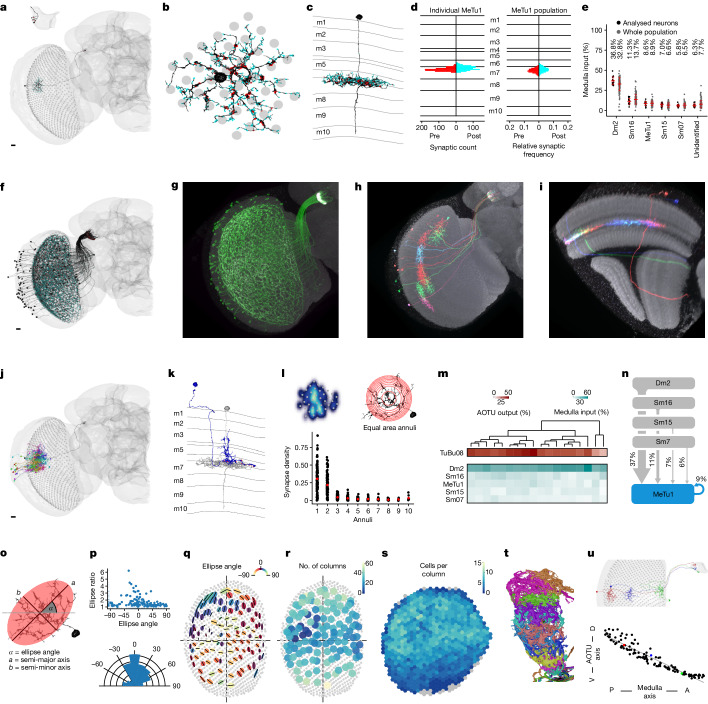
MeTu1 neurons form a homogeneous group. **a**, A single MeTu1_R neuron with presynapses (red) and postsynapses (cyan). Corner: magnified view of AOTUsu portion. Scale bar, 10 μm. **b**, Top view of the same neuron spanning medulla columns (grey). **c**, Side view of the neuron in **a**,**b**, with medulla layers labelled. **d**, Synapse distribution of the individual neuron in **a**–**c** (left) and of all MeTu1_R neurons (right) within medulla layers (as count per 100 nm or relative frequency, respectively). **e**, Medulla input percentage of the top five synaptic input types and unidentified types for analysed neurons (*n* = 18, black; see Methods) and all MeTu1_R neurons (*n* = 124, grey); mean and s.d. in red. **f**, All MeTu1_R neurons with synapses. Scale bar, 10 μm. **g**, Confocal image of MeTu1-specific split-GAL4 driver (SS00385). **h**,**i**, MeTu1 MultiColor FlpOut (MCFO) image, from anterior (**h**) and medial (**i**) medulla views. **j**, MeTu1_R neurons from **a** (black), with all upstream Dm2 partners. Scale bar, 10 μm. **k**, Side view of same MeTu1_R neuron (grey) and one Dm2 partner (blue, with red presynapses). **l**, Top left, synapse density of one MeTu1 neuron. Top right, equal area annuli (314.15 µm^2^). Bottom, synapse density for each annulus (red averages). **m**, Percentage of output (red) and input (cyan) of analysed MeTu1 neurons with TuBu (top) and upstream partners (bottom). **n**, Diagram of analysed MeTu1 top medulla inputs. **o**, Illustration of MeTu1’s dendritic span with elliptical measurement (see Methods). **p**, Top, all MeTu1_R ellipse ratios (semi-major axis to semi-minor axis) plotted against ellipse angles. Bottom, ellipse angles’ relative frequencies (Rayleigh test of uniformity: *P* = 2.061 × 10^−13^, z-value = 0.345, mean ellipse angle = 29.1). **q**, Ellipses of all MeTu1_R, semi-major axes (black lines) and ellipse angles (colour range). **r**, Number of columns spanned by each MeTu1_R. **s**, Number of MeTu1_R bounded by each medulla column (grey if none). **t**, All TuBu08_R rendered from the AOTUsu_R lateral side (*n* = 11). **u**, MeTu1 retinotopy. Top, three MeTu1_R neurons (red, blue and green) with similar dorsal–ventral medulla positions. Bottom, all MeTu1_R AOTUsu dorsal–ventral positions plotted against medulla posterior–anterior positions.

Compass neurons are strongly influenced by vertical stripes and their locations in azimuth^5,7^, whose information is conveyed by ER neurons, probably ER4d^6,9,12,39^. This ER neuron type is the only partner downstream of TuBu08, which is, in turn, the only neuron type downstream of MeTu1 neurons (Figs. 1d and 2): Our analysis of the anatomy and connectivity of MeTu1 neurons helps to explain the mechanisms that underlie the selectivity of ER4d neurons.

MeTu1 neurons (*n* = 121 left and *n* = 124 right; Fig. 2) form thick dendritic branches in the medulla layer 7, with small vertical protrusions extending to layer 6 (Fig. 2c,d). Dendrites span about 30–40 medulla columns (Fig. 2b,r) and each medulla column is innervated by multiple MeTu1 neurons (Fig. 2s). MeTu1 neurons receive the strongest input from Dm2 neurons covering the entire visual field (Fig. 2e,m,n; on average, 36 Dm2 neurons make 311 synapses per MeTu1 neuron), followed by Sm16 (serpentine layer neurons in medulla; following the FlyWire nomenclature^45,46^; see ref. ^47^ for matches between FlyWire names and male optic lobe names), MeTu1 (Supplementary Data 1a_i,ii_), Sm15 and Sm07 (Fig. 2e,m,n). The density of synapses drops at a distance of 20 to 30 µm from the medulla centroid of a MeTu1 neuron (Fig. 2l, bottom). The functional implication of recurrent connections between MeTu1 neurons in the medulla remains to be examined. The orientation of the MeTu1 dendritic span, when fitted with a two-dimensional Gaussian function, tends to be vertical. We observe that, near the anterior and posterior edges, the dendritic spans of MeTu neurons narrow (Fig. 2p,q), but future studies are required to ascertain whether this change has functional implications.

**Extended Data Fig. 4:**
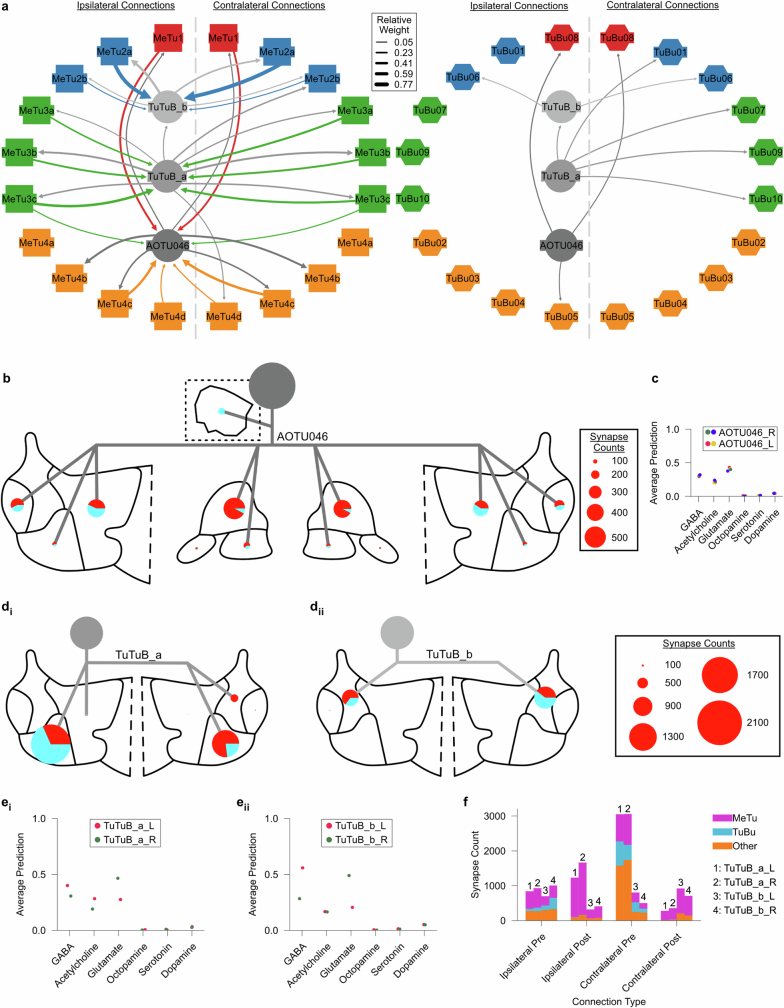
Bilateral neurons. **a**, Left: synaptic weight between bihemispheric neuron types and MeTu subtypes on the ipsilateral (left) and contralateral (right) sides. Right: similar to the left, Synaptic weight between bihemispheric neuron types and TuBu types. MeTu2a receives strong synaptic inputs from TuTuB_b on both sides, but none from TuTuB_a. It also reciprocally provides strong inputs to TuTuB_b on both sides, but only very weak input to TuTuB_a. **b**, Diagram of an AOTU046 neuron. AOTU046 neurons innervates AOTUsu_M, where they send sparse axons along the anterior/posterior–lateral face and extend boutons toward the posterior–medial triangle vertex at only a single latitude halfway down the dorsal–ventral axis. Pie charts are the ratio of presynaptic (red) to postsynaptic (cyan) connections to AVP neurons in the AOTU and Bulb regions, and connections to all neurons in the SPS. The SPS depicted in a cutout. Pie chart sizes are based on the relative number of connections (legend on the right). **c**, Average neurotransmitter prediction score over all synapses in each AOTU046 neuron for each type of neurotransmitter. **d**_**i,ii**_, Diagrams of TuBuB_a (**d**_**i**_) and TuBuB_b (**d**_**ii**_) neurons. Boutons of TuTuB_a sparsely protrude into the posterior central area only on the opposite side of the soma, while both axons and dendrites of TuTuB_b neurons innervate the entire AOTUsu_PC^16^. Pie charts are the ratio of presynaptic (red) to postsynaptic (cyan) connections to AVP neurons in the AOTU and Bulb. Pie chart sizes are based on the relative amount of connections (legend on the right). **e**_**i,ii**_, The average neurotransmitter prediction score over all synapses in each TuTuB_a (**e**_**i**_) (n = 2) or TuTuB_b (**e**_**ii**_) (n = 2) neuron for each type of neurotransmitter. **f**, Number of synapses of each TuTuB neuron between the ipsilateral and contralateral hemisphere, based on the type of neuron it is connected to.

MeTu1 neurons project axons to the AOTUsu_PL, where they synapse with TuBu08 neurons (Figs. 1d and 2m, Extended Data Fig. 4 and Supplementary Data 1j_i_), among other connections (Extended Data Fig. 4a and Supplementary Data 1f_i,ii_). The connection from MeTu1 neurons to TuBu08 neurons is retinotopic; the more anteriorly or posteriorly MeTu1 dendrites are located in the medulla, the more ventrally or dorsally they project in the AOTUsu_PL, respectively (Fig. 2u). In other words, each TuBu08 neuron receives input from a group of MeTu1 neurons at a particular azimuth, regardless of their elevation in the medulla. Such one-dimensional mapping provides a potential anatomical basis for the selectivity of TuBu08 neurons to vertical bars or to the azimuthal location of visual stimuli, but not to elevation (Supplementary Video 1), an anatomical structure similar to the classic Hubel & Wiesel model of how simple cells in the mammalian primary visual cortex receive input from the lateral geniculate nucleus in the thalamus^48^.

## MeTu2 subtypes process polarized light

**Fig. 3:**
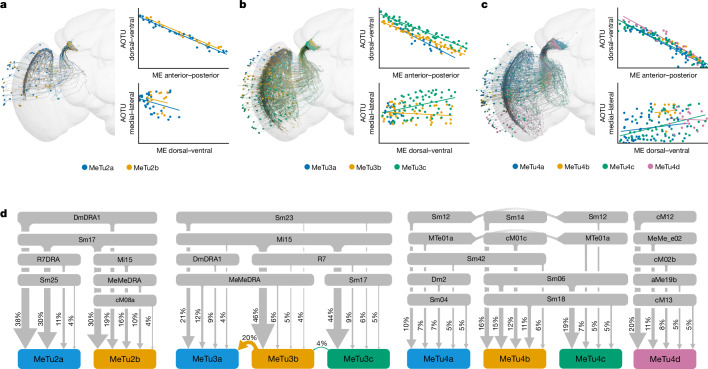
Retinotopy and inputs of other MeTu subtypes. **a**–**c**, Renders and retinotopy of MeTu2, MeTu3 and MeTu4, respectively. **a**, Left, render of MeTu2a_R (blue, *n* = 36) and MeTu2b_R (orange, *n* = 14). **b**, Left, render of MeTu3a_R (blue, *n* = 19), MeTu3b_R (orange, *n* = 46) and MeTu3c_R (green, *n* = 64). **c**, Left, render of MeTu4a_R (blue, *n* = 60), MeTu4b_R (orange, *n* = 12), MeTu4c_R (green, *n* = 48) and MeTu4d_R (purple, *n* = 18). **a–c**, Top right, AOTU dorsal–ventral positions plotted against medulla (ME) anterior–posterior positions for each neuron of the respective types. This retinotopy is maintained in all types. Bottom right, AOTU medial–lateral positions plotted against medulla dorsal–ventral positions for each neuron of the respective types. This retinotopy is seen mainly in MeTu3c. **d**, Top medulla input types of analysed MeTu neurons (see Methods) for MeTu2 (left), MeTu3 (middle) and MeTu4 (right). Percentages of input contribution to each type are shown.

**Extended Data Fig. 5:**
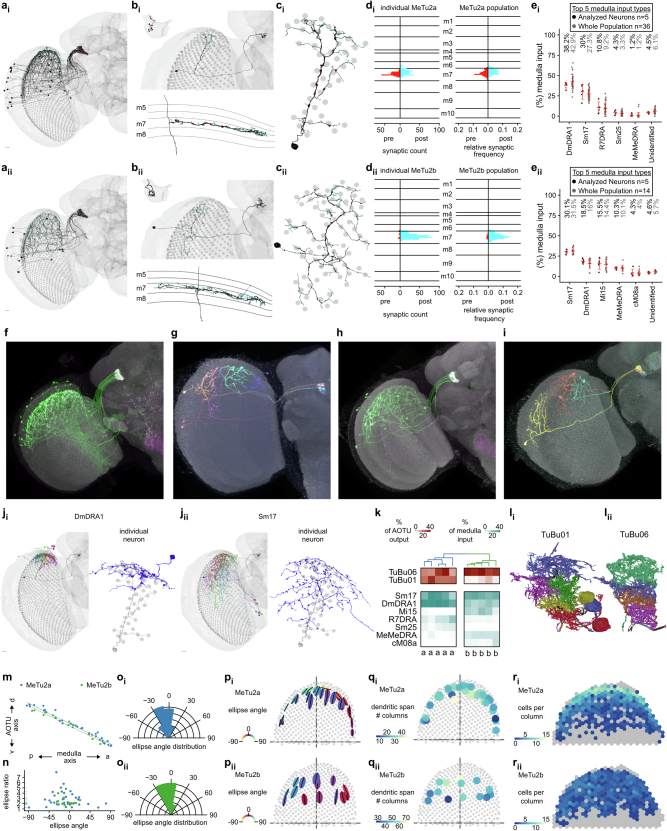
The two MeTu2 subtypes both process polarized skylight. **a**, All MeTu2a (**a**_**i**_) and MeTu2b (**a**_**ii**_) neurons of the right optic lobe. Presynapses are red and postsynapses are cyan. **b**, Top: single MeTu2a (**b**_**i**_) or MeTu2b (**b**_**ii**_) neuron, with a closeup of the AOTU portion in the top-left corner. Bottom: side view of the same neuron, with the medulla layers labelled on the left. **c**, Top view of the same neurons as in **b**, with the medulla columns it spans as grey circles. **d**, Left: synapse distribution of presynapses (red) and postsynapses (cyan) of the neurons in (**b**) with reference to the medulla layers. Right: synapse distribution of all MeTu2a/b respectively with reference to the medulla layers (relative frequency). **e**_**i,ii**_, Medulla input percentage of top 5 synaptic input types and unidentified types for MeTu2a (**e**_**i**_) and MeTu2b (**e**_**ii**_). Includes analysed neurons (black, MeTu2a n = 5, MeTu2b n = 5, see Methods) and all MeTu2_R (grey, MeTu2a n = 36, MeTu2b n = 14); means and SD in red. **f**, Confocal image of a MeTu2a specific split-GAL4 driver (SS00336). **g**, MCFO image of MeTu2a neurons (SS00336). **h**, Confocal image of a MeTu2b specific split-GAL4 driver (SS03744). **i**, MCFO image of MeTu2b neurons (SS03744). **j**, MeTu2 main presynaptic partners. **j**_**i**_, Left: MeTu2a from **b**_**i**_ in black, with all presynaptic DmDRA1 partners. Right: same MeTu2a in grey, along with a single DmDRA1 partner in blue. Presynapses from the DmDRA1 are red. **j**_**ii**_, Left: MeTu2a from **b**_**i**_ in black, with all presynaptic Sm17 partners. Right: Same MeTu2a shown in grey, along with a single Sm17 partner in blue. Presynapses from the Sm17 are red. **k**, Connectivity dendrogram of all analysed MeTu2a/b neurons (labelled on the bottom). Percentage of output (red) and input (cyan) to their top TuBu and upstream partners respectively. **l**_**i,ii**_, All TuBu01_R (**l**_**i**_, n = 5) and TuBu06_R (**l**_**ii**_, n = 4) rendered from the AOTUsu_R lateral side. **m**, Dorsal–ventral positions in the AOTU of all MeTu2a (blue) and MeTu2b (green) neurons in the right hemisphere as a function of their posterior–anterior positions in the medulla. **n**, Ellipse ratios (semi-major axis to semi-minor axis) of all MeTu2a (blue) and MeTu2b (green) of the right hemisphere as a function of the ellipse angles. **o**, Relative frequency of ellipse angles for all MeTu2a (**o**_**i**_) and MeTu2b (**o**_**ii**_) neurons in the right hemisphere (MeTu2a: Rayleigh Test of Uniformity: p = 2.136e-11, z-value = 0.584, mean ellipse angle=20.7; MeTu2b: Rayleigh Test of Uniformity: p = 4.905e-07, z-value = 0.682, mean ellipse angle=33.9). **p**, Ellipses of all MeTu2a (**p**_**i**_) and MeTu2b (**p**_**ii**_) neurons of the right optic lobe with their semi-major axes as black lines and the colour of the ellipse as a function of the ellipse angle. **q**, Number of columns spanned by each MeTu2a (**q**_**i**_) and MeTu2b (**q**_**ii**_) neuron of the right optic lobe. **r**, Number of MeTu2a (**r**_**i**_) and MeTu2b (**r**_**ii**_) neurons each medulla column of the right optic lobe contains within its bounds. Grey columns have zero cells.

Many insects navigate relying on skylight polarization^18,20,22,49^. In *Drosophila*, ER4m neurons are the prominent ER neurons that process skylight polarization^11,16^. MeTu2 neurons (Fig. 3a,d, left and Extended Data Fig. 5), previously designated as MeTu-DRA^37^ are notable as the only upstream inputs of these ER neurons^16^ (Supplementary Data 1e; through TuBu01 for ER4m; through TuBu06 in addition for ER5). They are clustered in the dorsal half of the medulla, with dendrites mainly tiling the dorsal rim area (DRA) (Extended Data Fig. 5a,r), where neurons process skylight polarization^11,35,50^.

Our clustering analysis identified two MeTu2 subtypes (Extended Data Fig. 5k and Supplementary Data 1j_ii_) with distinct ramifications in the medulla and notably distinct connectivity patterns in the AOTUsu_PC (Supplementary Data 1c_iii=iv_), which we named MeTu2a (*n* = 33 left and *n* = 36 right; Fig. 3d, left) and MeTu2b (*n* = 17 left and *n* = 14 right). Both subtypes exhibit generally vertical arborizations and similar dendritic spans (Extended Data Fig. 5n–p), but the interconnectivity between MeTu2b in the medulla is much stronger than that between MeTu2a (Supplementary Data 1a_iii,iv_,b_iii,iv_). Furthermore, MeTu2b neurons appear to receive more input from MeTu2a than they provide input to MeTu2a. Finally, although both MeTu2 subtypes mainly stratify within medulla layer 7 (Extended Data Fig. 5b,d), MeTu2a neurons are postsynaptic to polarization-sensitive R7 neurons only in the DRA and local interneurons DmDRA1, both of which are potentially sensitive to light polarization as well^37^, whereas MeTu2b inputs also include Mi15, the function of which remains unknown, and a stronger connection from interhemispheric MeMeDRA^37^ (Extended Data Figs. 5e,k and 3d_i_). Hence, MeTu2b might integrate additional inputs from the contralateral hemisphere, enabling the processing of a more global skylight polarization pattern.

MeTu2 innervation of the AOTUsu respects the same topographic rules as those described for MeTu1 (Fig. 3a and Extended Data Fig. 5m; MeTu2 neurons with dendrites in the anterior medulla project axons ventrally; those with posteriorly localized dendrites project dorsally in the AOTUsu_PC). Also, as in the medulla, MeTu2b neurons are more strongly interconnected in the AOTUsu than are MeTu2a neurons (Supplementary Data 1b_iii,iv_). Moreover, although MeTu2a and MeTu2b both synapse onto TuBu01 and TuBu06, MeTu2b is only weakly connected to TuBu01 (Extended Data Fig. 5k and Supplementary Data 1c_iii,iv_). Finally, MeTu2a and MeTu2b differ in their connections to the bilateral neurons TuTuB_a and TuTuB_b (Extended Data Figs. 5a,b and 4a,d). Overall, these connectivity differences in the AOTUsu_PC, combined with their distinct anatomical features in the medulla, indicate that MeTu2a and MeTu2b are likely to convey distinct features of skylight polarization to downstream circuits, with MeTu2b processing potentially more complex and global polarization patterns.

## MeTu3 neurons comprise three subtypes

**Extended Data Fig. 6:**
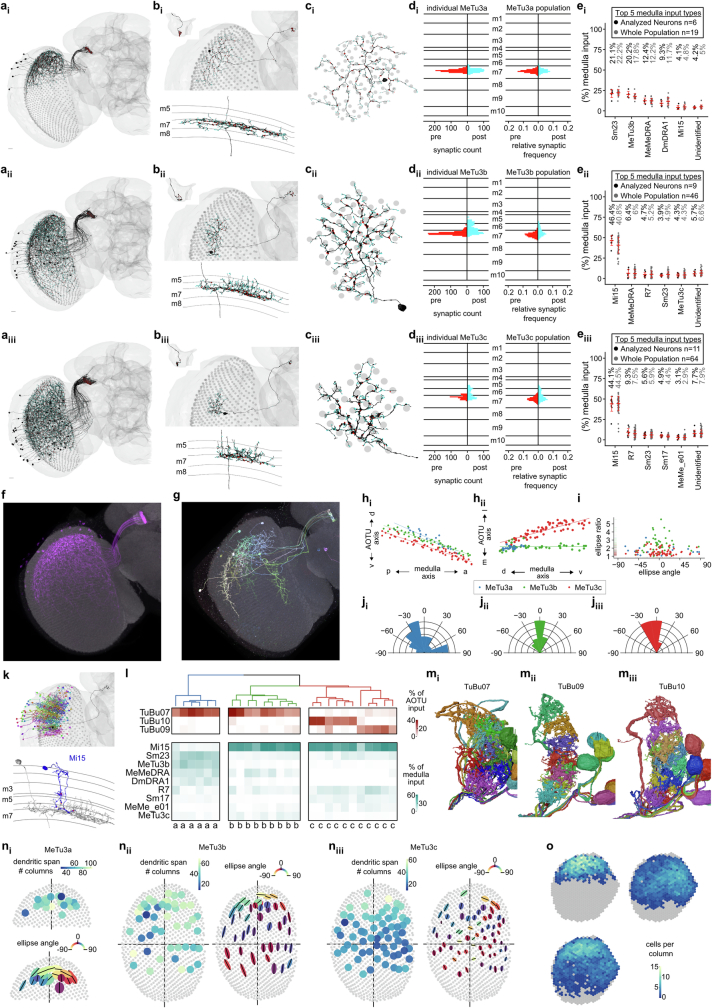
Three subtypes of MeTu3 are functionally segregated. **a**_**i–iii**_, Entire population of MeTu3a, MeTu3b and MeTu3c neurons respectively of the right hemisphere. Presynapses are red and postsynapses are cyan. **b**_**i–iii**_, Top: Single MeTu3a/b/c neuron respectively, with a closeup of the AOTU portion in the top-left corner. Bottom: Side view of the same, with the medulla layers labelled on the left. **c**_**i–iii**_, Top view of the same neurons in **b**_**i–iii**_, with the medulla columns it spans as grey circles. **d**_**i–iii**_, Synapse distribution of presynapses (red) and postsynapses (cyan) of the neurons in (**b**_**i-iii**_) with reference to the medulla layers. Right: Synapse distribution of all MeTu3a/b/c respectively with reference to the medulla layers (relative frequency). **e**_**i–iii**_, Medulla input percentage of top 5 synaptic input types and unidentified types for MeTu3a (**e**_**i**_), MeTu3b (**e**_**ii**_), and MeTu3c (**e**_**iii**_). Includes analysed neurons (black, MeTu3a n = 6, MeTu3b n = 9, MeTu3c n = 11, see Methods) and all MeTu3_R (grey, MeTu3a n = 19, MeTu3b n = 46, MeTu3c n = 64); means and SD in red. **f**, Confocal image of a MeTu3b specific split-GAL4 driver (SS00988). **g**, MCFO image of MeTu3b neurons (SS00988). **h**, Dorsal–ventral positions in the AOTU of all MeTu3a (blue), MeTu3b (green), and MeTu3c (red) neurons in the right hemisphere as a function of their posterior–anterior positions in the medulla. **i**, Ellipse ratios (semi-major axis to semi-minor axis) of all MeTu3 neurons of the right hemisphere as a function of the ellipse angles. **j**_**i–iii**_, Relative frequency of ellipse angles for all MeTu3a/b/c neurons in the right hemisphere respectively (MeTu3a: Rayleigh Test of Uniformity: p = 7.598e-04, z-value = 0.427, mean ellipse angle=1.2; MeTu3b: Rayleigh Test of Uniformity: p = 1.941e-14, z-value = 0.586, mean ellipse angle=33.3, MeTu3c: Rayleigh Test of Uniformity: p = 1.987e-08, z-value = 0.545, mean ellipse angle=35.9). **k**_,_ Top: MeTu3b from **b**_**ii**_ in black, along with all presynaptic Mi15 partners. Bottom: Side view of same MeTu3b in grey, with a single Mi15 partner in blue. Mi15 to MeTu3b synapses in red. **l**, Connectivity dendrogram of all analysed MeTu3a/b/c neurons (labelled on the bottom). Percentage of output (red) and input (cyan) to their top TuBu and upstream partners respectively. **m**_**i–iii**_, All TuBu07_R (**m**_**i**_, n = 9), TuBu09_R (**m**_**ii**_, n = 8), and TuBu10_R (**m**_**iii**_, n = 10) rendered from the AOTUsu_R lateral side. **n**, Morphometric analysis of MeTu3a (**n**_**i**_), MeTu3b (**n**_**ii**_) and MeTu3c (**n**_**iii**_) neurons. First plot: fitted ellipses with semi-major axes as black lines and the colour of the ellipse as a function of the ellipse angle. Second plot: number of columns spanned by each MeTu3 neuron of the right optic lobe. **o**, Number of MeTu3a (top left), MeTu3b (top right) and MeTu3c (bottom left) neurons each medulla column in the right optic lobe contains within its bounds. Grey columns have zero neurons.

The *Drosophila* compass neurons can use the two-dimensional organization of the surrounding world to compute the head direction^7^, but the source of this information was unclear. Here, we provide evidence that MeTu3 (Fig. 3b,d, middle and Extended Data Fig. 6) and its downstream neurons process, in addition to skylight polarization (through ER3w_ab), the two-dimensional organization of the scene (through ER2_ad, ER2_b and ER2_c).

Our connectivity analysis identified three distinct MeTu3 subtypes (MeTu3a, MeTu3b and MeTu3c; Fig. 3d, middle, Extended Data Fig. 6l and Supplementary Data 1j_iii_) with regionalized clusters of dendrites (Extended Data Fig. 6a). MeTu3a (*n* = 20 left and *n* = 19 right; Extended Data Fig. 6a_i_–e_i_) has dendrites that cluster in the dorsal third of the medulla (similar to MeTu2), are confined to layer 7 (Extended Data Fig. 6b_i_,d_i_) and specifically lack vertical protrusions across medulla layers (Extended Data Fig. 6b_i_,d_i_). MeTu3a is the only MeTu3 subtype that receives polarization information through input from DmDRA1, similar to MeTu2a and MeTu2b. MeTu3b cells (*n* = 53 left and *n* = 46 right) have dendrites that are clustered most densely in the dorsal half of the medulla but also extend to the ventral two-thirds, with pronounced vertical protrusions that cover layers 5, 6 and 7 (Extended Data Fig. 6b_ii_,d_ii_). They receive direct inhibitory input from ultraviolet (UV)-sensitive R7 photoreceptors and indirect input from blue/green-sensitive R8 cells^37^ through Mi15, which suggests that they have a role in processing chromatic information (Extended Data Figs. 6e_i,ii_,l,m and 7). MeTu3c cells (*n* = 72 left and *n* = 64 right) have dendrites that are more ventral than those of MeTu3b, covering the equator and some of the ventral part of the medulla (Extended Data Fig. 6b_iii_,e_iii_). Dendritic processes innervate the same layers (5, 6 and 7) as MeTu3b and receive the same direct and indirect photoreceptor inputs (Extended Data Fig. 6e_iii_), suggesting similar chromatic coding to that of MeTu3b.

MeTu3 innervation of the AOTUsu_A respects the same topographic rules as those described for MeTu1 and MeTu2 (anterior–posterior axis in the medulla to ventral–dorsal axis in the AOTUsu_A; Fig. 3b and Extended Data Fig. 6h). Axons of MeTu3a, MeTu3b and MeTu3c are not well segregated in the AOTUsu_A, despite the downstream TuBu neurons (TuBu07, TuBu09 and TuBu10; Supplementary Data 1c_v,vi_) having well-segregated dendrites^16^ (Fig. 1c and Extended Data Fig. 3c). Consequently, some MeTu3 neurons are connected to two TuBu types (Extended Data Fig. 6l and Supplementary Data 1c_v,vi_,j_iii_). All three MeTu3 subtypes are strongly and reciprocally connected to bilateral TuTuB_a neurons (Extended Data Fig. 4g and Supplementary Data 1f_i,ii_). Because the TuTuB_a neurotransmitter is predicted to be inhibitory^51^ (Extended Data Fig. 4e_i_), MeTu3 neurons might exhibit strong bilateral inhibitory interactions across the entire visual field.

MeTu3a and MeTu3b neurons are mainly connected to TuBu07, upstream of ER3w_ab. This convergence of MeTu3a and MeTu3b suggests that ER3w_ab encodes a combination of skylight polarization and chromatic information of the sky. On the other hand, MeTu3c neurons are mostly presynaptic to both TuBu09 and TuBu10 (Extended Data Fig. 6l and Supplementary Data 1c_v,vi_). Notably, TuBu09 neurons receive input from MeTu3c neurons with dendrites located more dorsally in the medulla, whereas TuBu10 neurons receive input from MeTu3c neurons with dendrites located more ventrally in the medulla (Extended Data Fig. 6h_ii_, red dots). Thus, the neurons downstream of MeTu3c can encode the elevation of visual stimuli (for example, the Sun) or a two-dimensional organization of visual objects in a surrounding scene. This capability is unique among all MeTu neurons.

## MeTu4 subtypes transmit widefield inputs

**Extended Data Fig. 8:**
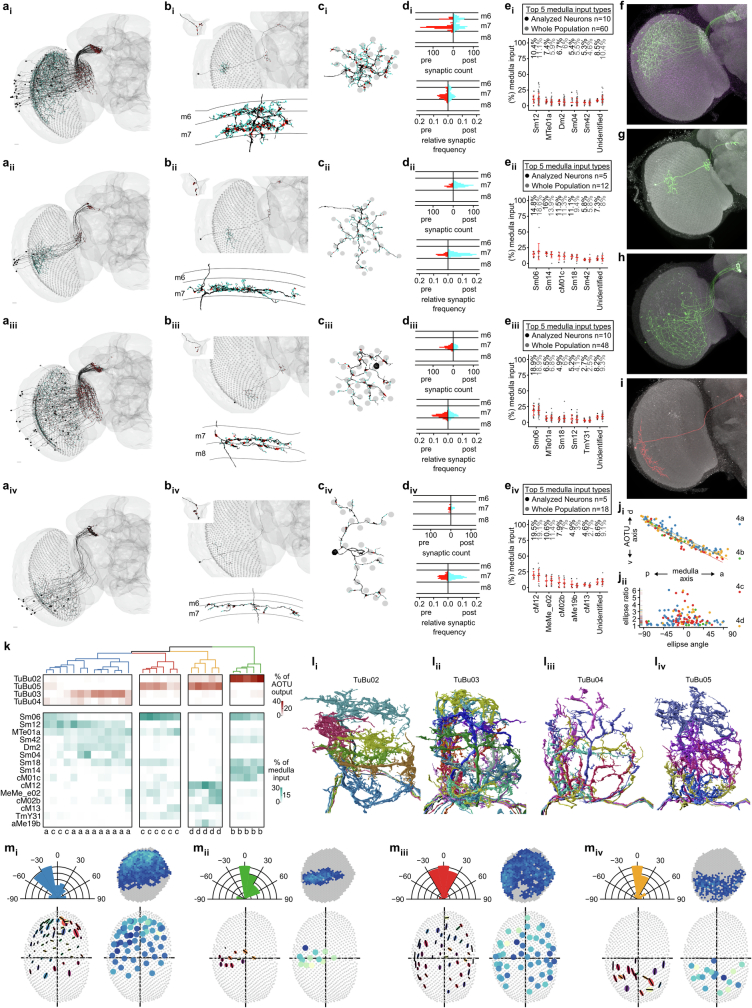
Four MeTu4 subtypes convey widefield visual inputs. **a**_**i–iv**_, Entire population of MeTu4a, MeTu4b, MeTu4c and MeTu4d neurons respectively of the right hemisphere. Presynapses are red and postsynapses are cyan. Note that MeTu4a/b/c have presynaptic connections in lobula (see Extended Data Fig. 9h for synapse counts in lobula). **b**_**i–iv**_, Top: Single MeTu4a/b/c/d neuron respectively, with a closeup of the AOTU portion in the top-left corner. Bottom: Side view of the same neuron, with the medulla layers labelled on the left. **c**_**i–iv**_, Top view of the same neurons in **b**_**i–iv**_, with the medulla columns it spans as grey circles. **d**_**i–iv**_, Top: Synapse distribution of presynapses (red) and postsynapses (cyan) of the neurons in **b**_**i–iv**_ with reference to the medulla columns. Bottom: Synapse distribution of all MeTu4a/b/c/d respectively with reference to the medulla layers (relative frequency). **e**_**i–iv**_, Medulla input percentage of top 5 synaptic input types and unidentified types for MeTu4a (**e**_**i**_), MeTu4b (**e**_**ii**_), MeTu4c (**e**_**iii**_), and MeTu4d (**e**_**iv**_). Includes analysed neurons (black, MeTu4a n = 10, MeTu4b n = 5, MeTu4c n = 10, MeTu4d n = 5, see Methods) and all MeTu4_R (grey, MeTu4a n = 60, MeTu4b n = 12, MeTu4c n = 48, MeTu4d n = 18); means and SD in red. **f**, Confocal image of a MeTu4a specific split-GAL4 driver (SS03719). **g**, MCFO image of MeTu4a neuron (SS03719). **h**, Confocal image of a MeTu4d specific split-GAL4 driver (SS23880). **i**, MCFO image of MeTu4d neuron (SS23880). **j**, Top: dorsal–ventral positions in the AOTU of all MeTu4a (blue), MeTu4b (green), MeTu4c (red) and MeTu4d (yellow) neurons in the right hemisphere as a function of their posterior–anterior positions in the medulla. Bottom: ellipse ratios (semi-major axis to semi-minor axis) of all MeTu4 neurons of the right hemisphere as a function of their ellipse angles. **k**, Connectivity dendrogram of all analysed MeTu4a/b/c/d neurons (labelled on the bottom). Percentage of output (red) and input (cyan) to their top TuBu and upstream partners respectively. **l**_**i–iv**_, All TuBu02_R (**l**_**i**_, n = 6), TuBu03_R (**l**_**ii**_, n = 12), TuBu04_R (**l**_**iii**_, n = 5), and TuBu05_R (**l**_**iv**_, n = 5) rendered from the AOTUsu_R lateral side. **m**, Morphometric analysis of MeTu4a (**m**_**i**_), MeTu4b (**m**_**ii**_), MeTu4c (**m**_**iii**_) and MeTu4d (**m**_**iv**_) neurons. Top-left: Relative frequency of ellipse angles (MeTu4a: Rayleigh Test of Uniformity: p = 3.319e-14, z-value = 0.509, mean ellipse angle=7.4; MeTu4b: Rayleigh Test of Uniformity: p = 3.324e-02, z-value = 0.374, mean ellipse angle=64.4; MeTu4c: Rayleigh Test of Uniformity: p = 1.391e-12, z-value = 0.533, mean ellipse angle=39.8; MeTu4d: Rayleigh Test of Uniformity: p = 1.370e-04, z-value = 0.485, mean ellipse angle=51.6). Top-right: Number of respective MeTu4a/b/c/d neurons each medulla column in the right optic lobe contains within its bounds. Grey columns have zero cells. Color scale is the same as Extended Data Fig. 6o. Bottom-left: Fitted ellipses with their semi-major axes as black lines and the colour of the ellipse as a function of the ellipse angle. Bottom-right: Number of columns spanned by each MeTu4 neuron in the right hemisphere.

Compass neurons receive diverse input from ER neurons, some of which exhibit responses to the contralateral visual field and self-generated motion signals^12,39^. Considering that these ER neurons, the dendrites of which are in the inferior bulb, are downstream of MeTu4 neurons (Fig. 3c,d, right, Extended Data Fig. 8 and Supplementary Data 1c_vii,viii_,d_vii,viii_) that originate in the ipsilateral optic lobe, their response pattern was puzzling. Our analyses show that MeTu4 neurons receive inputs from distinct parts of the visual world (dorsal, frontal or ventral), with virtually no input from a columnar medulla cell type. Instead, they receive input mostly from large interneurons that span many medulla columns (Sm neurons) and from others that potentially convey motor information from a neuropil called the superior posterior slope (SPS) to the medulla (Extended Data Fig. 8e). These unique properties of MeTu4 might explain the mysterious properties of ER neurons in the inferior bulb^12,39^.

On the basis of the connectivity in the medulla and the AOTUsu_M, we categorized MeTu4 into four subgroups: MeTu4a, MeTu4b, MeTu4c and MeTu4d (Extended Data Fig. 8a). The dendrites of MeTu4a cells (*n* = 69 left and *n* = 60 right) cluster densely in the dorsal half of the medulla but also extend ventrally (Extended Data Fig. 8a_i_,m_i_), with unique arborization in two medulla layers (M6 and M7; Extended Data Fig. 8b,d). Despite their dorsal location, they form no synaptic connections with polarized light-sensitive photoreceptors or DRA neurons. MeTu4b neurons (*n* = 8 left and *n* = 12 right) are notable for their unique dendritic arrangement: they span a rather small area in the equator, mostly in the posterior–medial part of the medulla that represents the frontal central visual field (because of the crossover connections from the lamina to the medulla along the anterior–posterior axis; Extended Data Fig. 8a_ii_,m_ii_). The function of this spatial restriction remains unknown (see also the next section about variance across brains). MeTu4c neurons (*n* = 41 left and *n* = 48 right) span the entire dorsal half of the medulla (Extended Data Fig. 8a_iii_,m_iii_), whereas MeTu4d neurons (*n* = 19 left and *n* = 18 right) cluster exclusively in the ventral half of the medulla (Extended Data Fig. 8a_iv_,m_iv_) and are ideally positioned to detect features in the ventral visual field. Both MeTu4c and MeTu4d receive nearly identical input from a wide variety of interneurons, including those that convey information from other brain areas, such as the SPS (Extended Data Figs. 8e_iii,iv_,k and 3d_iii_).

Like all other MeTu types, axonal projections of all MeTu4 neurons maintain anterior–posterior retinotopy in the AOTUsu_M along the ventral–dorsal axis (Fig. 3c, Extended Data Fig. 8j), in contrast to what was found in a previous report^14^. MeTu4a, MeTu4b and MeTu4c also have presynaptic connections in the lobula (Extended Data Fig. 8a_i–iii_), but these connections do not contribute to the AVP and were thus excluded from further analyses. In the AOTUsu_M, all MeTu4a neurons are presynaptic to TuBu03; some are also presynaptic to TuBu04 (Supplementary Data 1f). MeTu4b neurons are presynaptic to TuBu02 neurons. Both MeTu4c and MeTu4d subtypes are mainly presynaptic to TuBu05 (Supplementary Data 1c_vii,viii_), but MeTu4d also makes presynaptic connections with TuBu02. MeTu4b and MeTu4c receive the main interhemispheric connections within the AOTUsu_M (Extended Data Fig. 4a,b and Supplementary Data 1f_i,ii_): MeTu4b receives strong input from AOTU046 but does not provide reciprocal input into AOTU046, whereas MeTu4c is strongly and reciprocally connected to AOTU046. MeTu4d receives no input from AOTU046, and provides only weak input to AOTU046. Finally, MeTu4d receives weak input from TuTuB_a.

## Variance across hemispheres and brains

**Extended Data Fig. 9:**
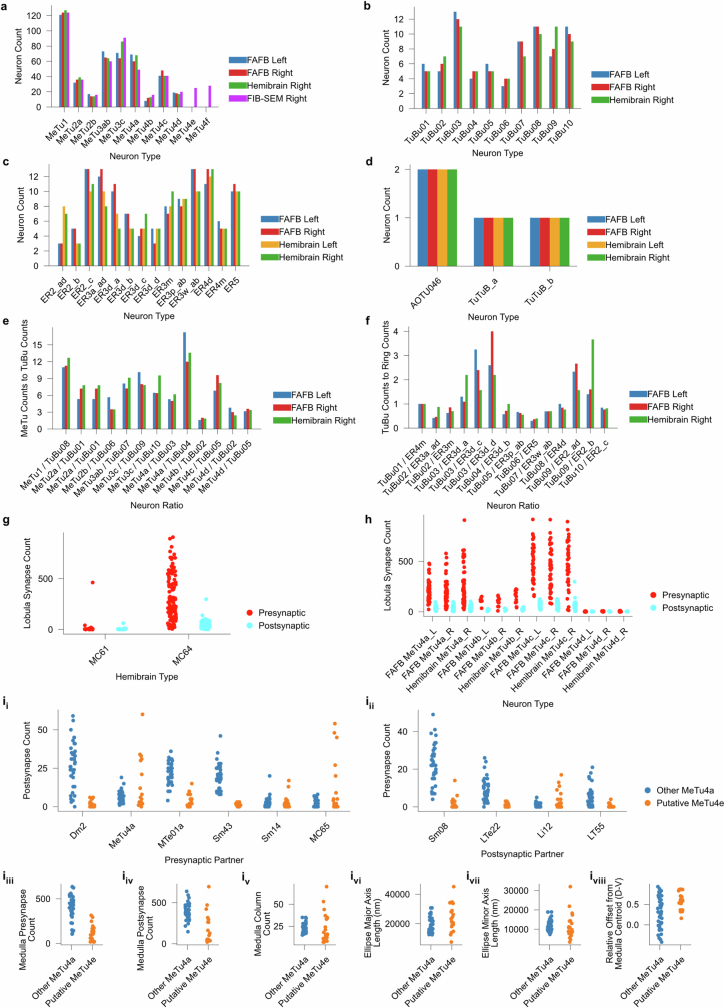
Variations across hemisphere and brains. **a**–**d**, Comparing neuron counts between both hemispheres in the FAFB and applicable hemispheres in the hemibrain and FIB-SEM datasets, of MeTu (**a**), TuBu (**b**), ER (**c**) and bihemispheric (**d**) neurons. Note the differences between the datasets. For example, MeTu4e-f, which have been discovered in the male FIB-SEM connectome, but not in the female FAFB or hemibrain connectomes are noted in **a**. In addition, in FAFB there are only six ER2_a/d neurons (3/side) while the hemibrain has 15 of these neurons (eight on the left, seven on the right) (**c**). Because there were no verifiable differences among these neurons, we categorized them as a single group. Similarly, although the total number of ER3a_ad neurons was similar between FAFB and hemibrain data, we were unable to identify distinct features to differentiate ER3a_a and ER3a_d in FAFB (**c**). Thus, we combined them into a single group as well. We did the same with ER3p_ab and ER3w_ab, as potential subtypes were similarly indistinguishable (**c**). **e**,**f**, Comparing the ratios of MeTu to TuBu (**e**) and TuBu to ER (**f**) between both datasets in applicable hemispheres. **g**, Number of lobula presynapses (red) and postsynapses (cyan) of all MeTu4 neurons in the hemibrain dataset, sorted by whether they were previously classified as MC61 (n = 36) or MC64 (n = 242). MeTu4d neurons were generally classified as MC61, due to not having synapses in the lobula. **h**, Number of lobula presynapses (red) and postsynapses (cyan) of all MeTu4 neurons in both datasets after subclassification. FAFB MeTu4a_L: n = 138, FAFB MeTu4a_R: n = 120, FAFB MeTu4b_L: n = 16, FAFB MeTu4b_R: n = 24, FAFB MeTu4c_L: n = 82, FAFB MeTu4c_R: n = 96, FAFB MeTu4d_L: n = 38, FAFB MeTu4d_R: n = 36, Hemibrain MeTu4a_R: n = 136, Hemibrain MeTu4b_R: n = 26, Hemibrain MeTu4c_R: n = 82, Hemibrain MeTu4d_R: n=34. **i**_**i–viii**_, Comparisons between putative MeTu4e (from MeTu4a) based on the FIB-SEM characterization and other MeTu4a neurons. **i**_**i,ii**_ are comparisons between the number of synaptic connections with neurons in the medulla, specifically presynaptic partners (**i**_**i**_) and postsynaptic partners (**i**_**ii**_). Dm2: n = 60, LT55: n = 60, LTe22: n = 60, Li12: n = 60, MC65: n = 60, MTe01a: n = 60, MeTu4a: n = 60, Sm08: n = 60, Sm14: n = 60, Sm43: n = 60. **i**_**iii–viii**_ are comparisons between various characteristics, including the number of presynapses (**i**_**iii**_) and postsynapses (**i**_**iv**_) in the medulla, the number of columns the neurons occupy in the medulla (**i**_**v**_), the dendritic major axis (**i**_**vi**_) and minor axis (**i**_**vii**_) length in nm and the relative location along the dorsal–ventral axis in the medulla, with respect to the medulla centroid (**i**_**viii**_). Other MeTu4a: n = 39, Putative MeTu4e: n = 21.

We compared the AVP within^17,44^ and across brains^16,47^ (Extended Data Fig. 9). Although our analyses are limited, owing to the lack of optic lobes in the hemibrain dataset and a single optic lobe in the male brain dataset, tentative comparisons are still possible for most MeTu types, on the basis of synapses in the AOTU (hemibrain) or optic lobe synapse and cell shapes (male optic lobe). The number of neurons of each type was very similar across hemispheres of the same brain (in both FAFB and hemibrain), compared to the difference across brains. Notably, across brains, only a few cell types showed clear differences in spatial arborization patterns, numbers or, in very few cases, perhaps even their existence (for example, MeTu3c, MeTu4b, MeTu4e, MeTu4f, TuBu9, ER2_ad, ER3d_a and others; Extended Data Fig. 9). For example, MeTu4b neurons in FAFB (the dataset used here) and the male brain optic lobe both have similar spatially restricted arborizations in the medulla, but the FAFB pattern lacks a few cells (in the lateral and frontal medulla) that are present in the male optic lobe MeTu4b population. Also, the overall number of MeTu4 cells in the male brain dataset is higher and two other MeTu4 subtypes (MeTu4e and MeTu4f) are identified. Careful comparison suggests that MeTu4e in the male optic lobe could be a subset of MeTu4a in FAFB (the segregation of morphological and connectivity patterns between candidate MeTu4e and the remaining MeTu4a appears less clear in FAFB and there are fewer MeTu4e-like neurons; Extended Data Fig. 9i). The source of such variations across brains remains incompletely understood but is likely to be, at least in part, developmental in nature^52^.

## Extracting visual features along the AVP

**Extended Data Fig. 10:**
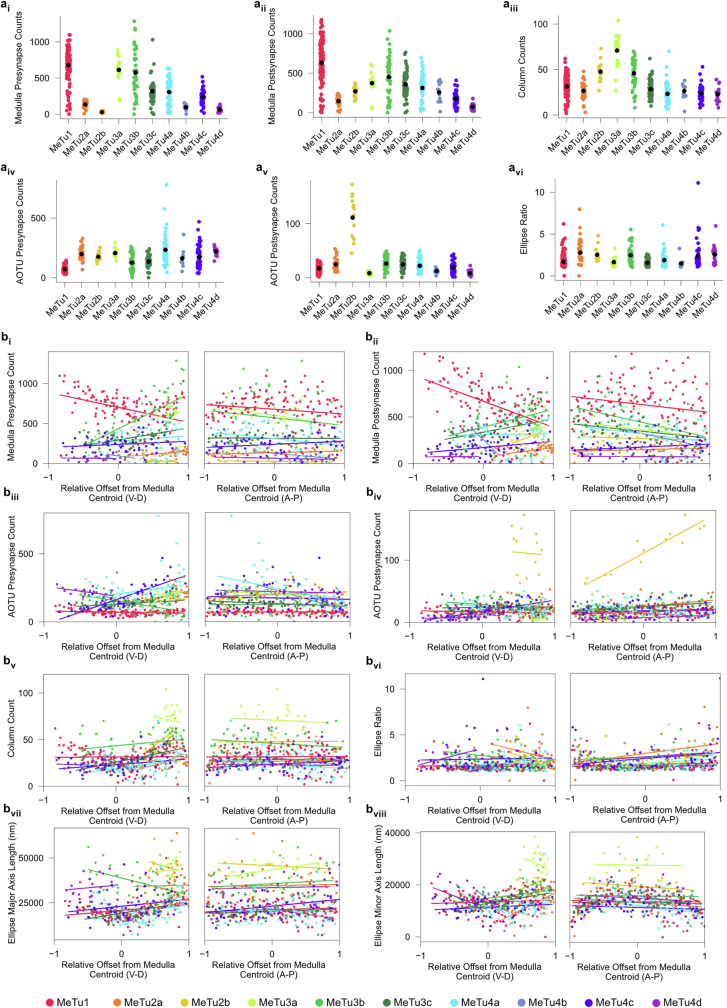
Comparisons between MeTu subtypes. **a**, Comparisons between all MeTu subtypes in the right hemisphere. Number of presynapses in the medulla (**a**_**i**_), number of postsynapses in the medulla (**a**_**ii**_), number of medulla columns that each neuron’s dendritic span occupies (**a**_**iii**_), number of presynapses in the AOTU (**a**_**iv**_), number of postsynapses in the AOTU (**a**_**v**_) and the ellipse ratio of the dendrites in the medulla (**a**_**vi**_). **b**, Comparisons among all MeTu subtypes in the right hemisphere with respect to the ventral–dorsal axis (negative values are ventral, positive values are dorsal) and the anterior–posterior axis (negative values are anterior, positive values are posterior) of the medulla. Scatter plots are of all MeTu neurons and their lines of best fit. The colour legend is at the bottom of **b**. **b**, The values compared with respect to the V-D and A–P axes are the number of presynapses in the medulla (**b**_**i**_), the number of postsynapses in the medulla (**b**_**ii**_), the number of presynapses in the AOTU (**b**_**iii**_), the number of postsynapses in the AOTU (**b**_**iv**_), the number of medulla columns in the dendritic span (**b**_**v**_), the dendritic ellipse ratio (**b**_**vi**_), the length of the major axis of the dendritic ellipse in nm (**b**_**vii**_) and the length of the minor axis of the dendritic ellipse in nm (**b**_**viii**_). See Extended Data Fig. 11 for the number of neurons of each type.

**Extended Data Fig. 11:**
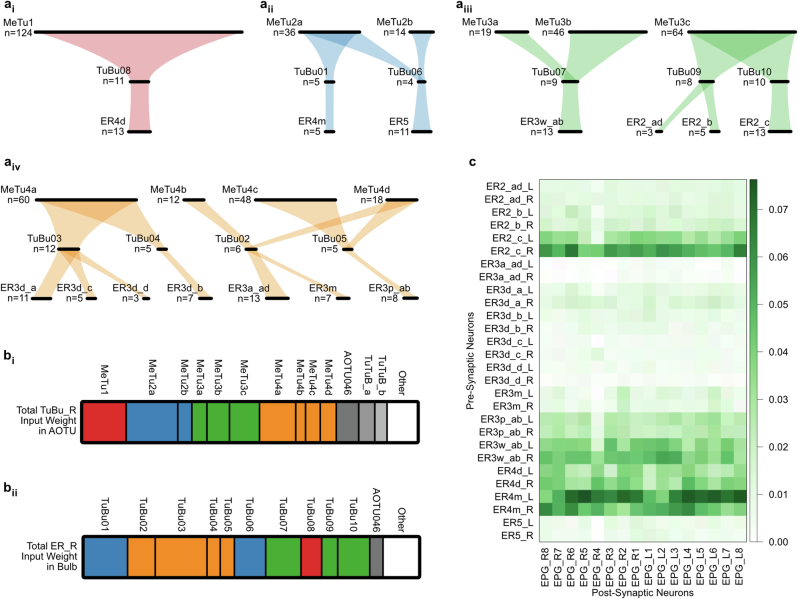
Overview of neuron counts and synaptic weights in the pathway. **a**_**i–iv**_, The number of MeTu, TuBu and ER neurons in the pathway. Line width is directly correlated with neuronal counts in the right hemisphere, and coloured shapes represent connected subtypes. This includes the MeTu1 (**a**_**i**_), MeTu2 (**a**_**ii**_), MeTu3 (**a**_**iii**_) and MeTu4 (**a**_**iv**_) pathways. **b**_**i,ii**_, The total synaptic weight of a broad neuron type in their dendritic region. The outer rectangles represent the sum of all postsynapses in that region for that neuron type. The relative amount of contribution from various input types is shown. **b**_**i**_ shows the synaptic weight of all right hemisphere TuBu neurons in the AOTU from various input types, while **b**_**ii**_ is the synaptic weight of all right hemisphere ER neurons in the bulb. **c**, The synaptic weight matrix of ER neuron-to-EPG type connectivity in the ellipsoid body.

A common pattern across all AVP channels is the convergence of MeTu neurons onto a considerably smaller number of TuBu neurons (Extended Data Fig. 9e and Supplementary Data 1c). In this transformation, each TuBu neuron integrates information from a large area of the visual field, suggesting spatial feature processing with a lower resolution. TuBu neurons also receive strong input from the contralateral visual field through TuTu neurons (Extended Data Fig. 6). Note that individual MeTu neurons sample the visual area differently depending on their location in the medulla over the anterior–posterior axis. This results in synapse counts and dendritic field shapes differing by their locations in the medulla along this axis (Extended Data Fig. 10). The effect of this pattern on TuBu integration is not understood at present. The next step in processing—from TuBu to ER neurons—exhibits a re-expansion in the number of neuronal types (from 10 TuBu types to potentially 18 ER neuron types in hemibrain, or 14 ER neuron types in FAFB). The ratio of connections from TuBu to ER neurons (Extended Data Fig. 9f and Supplementary Data 1d; see also ref. ^16^) varies between 0.33 and, for some neurons, 4 (Extended Data Fig. 11). Thus, the transformation from TuBu to ER may extract several more visual features.

## Putative receptive fields of ER neurons

**Fig. 4:**
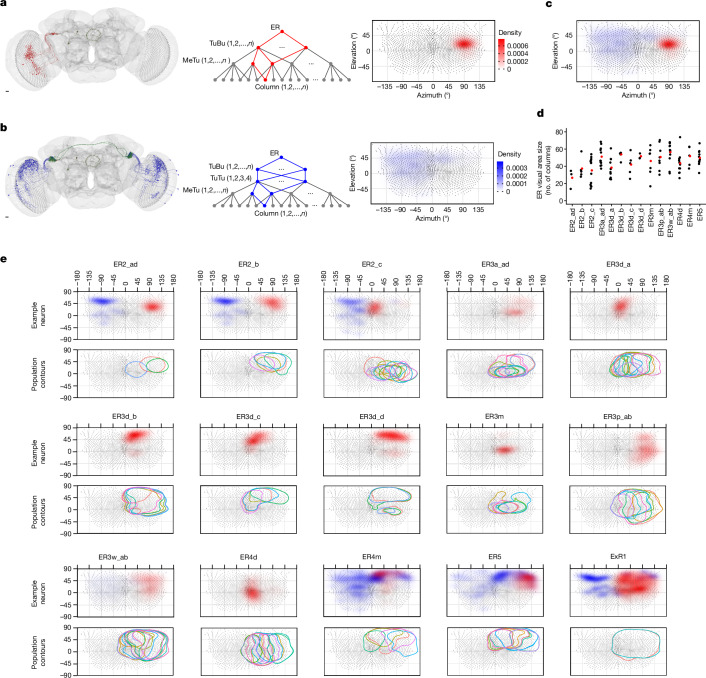
Putative visual areas of ER neurons in the right hemisphere. **a**, Direct pathway (putatively excitatory). Left, all connected TuBu and upstream MeTu neurons for a given exemplary ER neuron. Middle, connectivity graph illustration of the direct pathway. In red are all branches connecting to one given column. Right, resulting putative visual area. The eye map was developed using microCT data. Scale bar, 10 μm. **b**, Indirect pathway (putatively inhibitory). Left, all connected TuBu, upstream TuTu and upstream MeTu neurons for a given exemplary ER neuron. Middle, connectivity graph illustration of the indirect pathway. In blue are all branches connecting to one given column. Right, resulting putative visual area. We did not analyse the AOTU046 pathway because its neurotransmitter was not conclusive^51^. Scale bar, 10 μm. **c**, Overlaid direct and indirect visual areas predicted from **a**,**b**. **d**, Visual area size as the number of covered columns for all visual ER neurons of the right hemisphere. Red point: population average. **e**, For all visual ER types (columns) of the right hemisphere, we show an exemplary visual area of individual neurons (top of each ER type) and a contour outline of the visual area of all neurons of a given ER type (bottom of each ER type). ER2_ad_R, *n* = 3; ER2_b_R, *n* = 5; ER2_c_R, *n* = 13; ER3a_ad_R, *n* = 13; ER3d_a_R, *n* = 11; ER3d_b_R, *n* = 7; ER3d_c_R, *n* = 5; ER3d_d_R, *n* = 3; ER3m_R, *n* = 7; ER3p_ab_R, *n* = 8; ER3w_ab_R, *n* = 13; ER4d_R, *n* = 13; ER4m_R, *n* = 5; ER5_R, *n* = 11; ExR1_R, *n* = 2.

**Extended Data Fig. 12:**
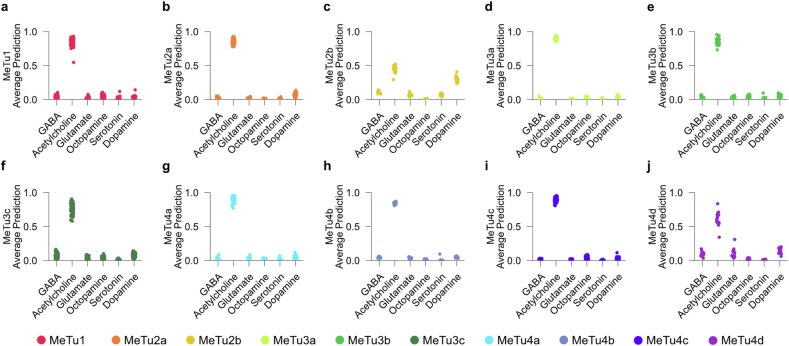
Differences in the predicted neurotransmitters. **a**–**j**, Average neurotransmitter prediction score over all synapses in each MeTu1 (**a**), MeTu2a (**b**), MeTu2b (**c**), MeTu3a (**d**), MeTu3b (**e**), MeTuc3c (**f**), MeTu4a (**g**), MeTu4b (**h**), MeTu4c (**i**) and MeTu4d (**j**) neuron, for each type of neurotransmitter. Note that the neurotransmitter of MeTu2b is not clearly predicted as cholinergic. See Extended Data Fig. 11 for the number of neurons of each type.

To quantify the putative visual area to which each neuron probably responds, we mapped each medulla column (Extended Data Fig. 1e) to a micro-computed tomography (microCT)-based eye map of *D. melanogaster*^53^ (Supplementary Data 4). Then, for each ER neuron, we back-traced the upstream connections in two ways: one followed TuBu to MeTu connections (we call this the direct pathway, putatively excitatory; Fig. 4a, Extended Data Figs. 11 and 12 and Supplementary Videos 1–3) and the other followed TuBu to TuTu to ipsi- and contralateral MeTu connections (we call this the indirect pathway, putatively inhibitory; Fig. 4b and Extended Data Fig. 4d–f). We used the dendritic arborization in the medulla for each pathway to estimate the area of a direct pathway or an indirect pathway. We overlaid them into a single putative receptive field (Fig. 4c,d), which we further analysed to obtain the outline of the visual field of the direct pathway. We then combined outlines of the same type of ER neurons to illustrate the visual area that the population of ER neurons covers (Fig. 4e). Note that the lack of functional data for most optic lobe cell types, including Sm neurons, hinders extensive functional predictions. Thus, our predictions are based mainly on the dendritic fields of MeTu neurons and the known properties of presynaptic neurons.

**Fig. 5:**
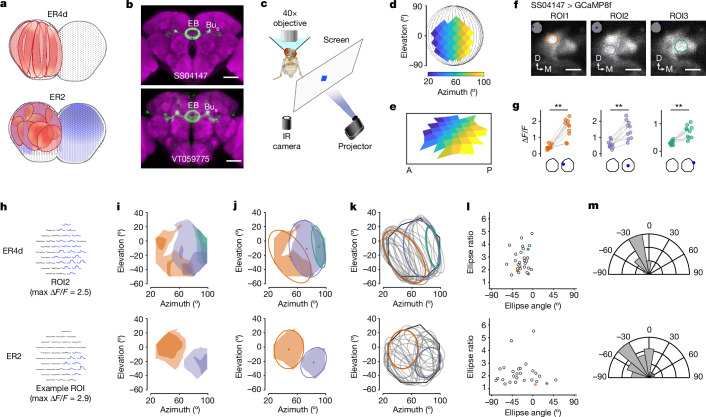
Physiological testing of predictions for ER4d and ER2 neurons. **a**, Putative receptive fields for ER4d and ER2. **b**, Confocal image of ER neuron driver lines. Left, ER4d split-GAL4 line (SS04147). Right, ER2 Gal4 line (VT059775). Dendrites of ER4d and ER2 innervate the superior bulb (BU_s_). Scale bars, 50 µm. EB, ellipsoid body. **c**, Imaging set-up. Fly heads were tilted 30–45^o^ to expose the left eye to the stimulus. IR, infrared. **d**, The stimulated visual area in the fly’s eye coordinates. **e**, The projection of the 38 predetermined squares (dots) on the screen. In each trial, a single dot was back-projected on the screen for 1 s, followed by 1 s of darkness. **f**, Snapshots of two-photon calcium imaging video of ER4d neurons in the BU_s_ with an overlay of regions of interest (ROIs) selected on the basis of responses to stimuli. Individual ER neurons form a microglomerulus in the bulb. ER neurons (ROI1–ROI3) respond to dots appearing at different positions. Scale bars, 3 μm. Coordinates: D, dorsal; M, medial. **g**, Calcium activity (Δ*F*/*F*) of ROI1–ROI3 (left to right) before and during stimulation (*n* = 10 trials each; ROI1 *P* = 0.0020, ROI2 *P* = 0.0030, ROI3 *P* = 0.0039; Wilcoxon signed-rank test). **h**, Top, average calcium traces of ten trials of ROI2 in **f** arranged in the eye coordinates. Bottom, an ROI from an example ER2 neuron. **i**, Receptive field contour plot, contours at 20% (lighter shade) and 50% (darker shade) maximum Δ*F*/*F* of individual ROIs. Top, data are from ROI1–3 shown in **f**. **j**, Fitted ellipses at 20% contour. Dots represent the ellipses’ centroids. **k**, Fitted ellipses at 20% contour of ROIs collected from 11 flies (ER4d: 29 ROIs; ER2: 27 ROIs). **l**, Receptive field ellipse ratios as a function of ellipse angles. Ellipse ratio medians of ER4d and ER2 are significantly different (*P* = 0.0030, Wilcoxon rank sum test). **m**, Polar histograms of ellipse angles. The ellipse angles are not uniformly distributed (ER4d *P* = 4.673 × 10^−14^, ER2 *P* = 0.0033, Rayleigh’s test for nonuniformity). Source Data

**Extended Data Fig. 13:**
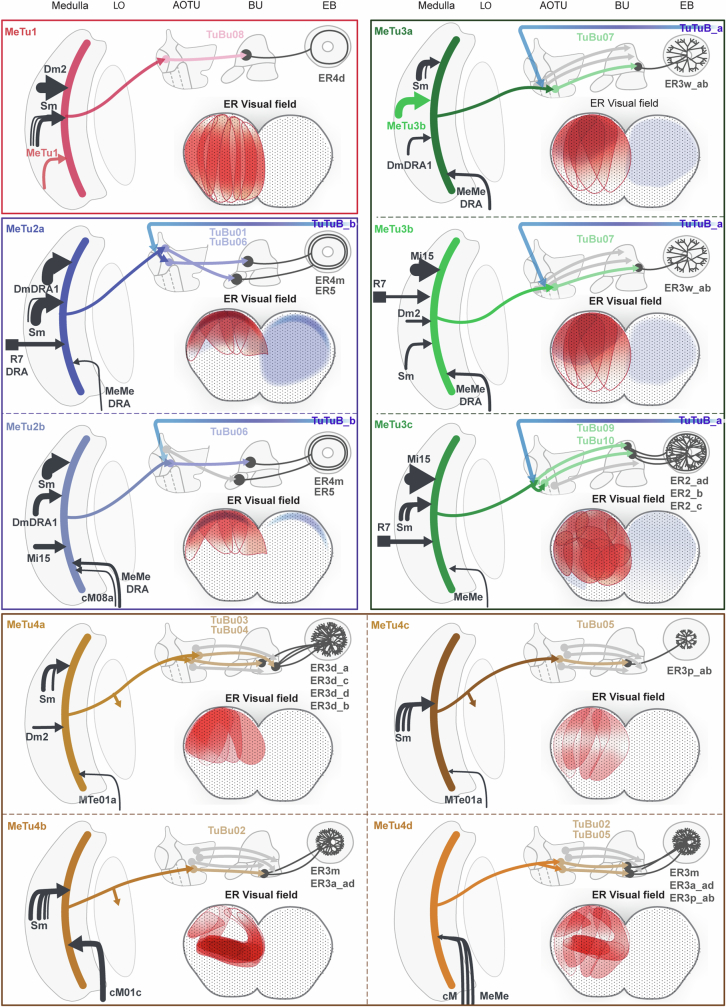
Overviews of parallel AVPs. Each panel shows a generalized neural pathway and receptive field of a MeTu subtype. Note that ExR1 is excluded because it integrates information from all TuBu types. From the left to top right side, there is a diagram of the pathway from the medulla to the EB. Medulla inputs to MeTu are shown on the left of the medulla if they come from the retina or medulla, and on the right if they come from the central brain. Photoreceptor inputs in the retina are shown as squares. The AVP from the MeTu to the TuBu and ER neurons are shown, as well as if the MeTu has outputs in the lobula or synapses with TuTuB neurons in the AOTU. The receptive fields of the relevant ER neurons are on the bottom right. Red indicates input from MeTu neurons via direct pathways, whereas blue indicates indirect input via inhibitory TuTuB neurons. AOTU046 is not included because the excitatory or inhibitory nature of the neuron is unknown^51^. The receptive fields of ER neurons represent all inputs to ER neurons. For example, ER3p_ab receives input from both MeTu4c and MeTu4d. Therefore, the receptive field covers the ventral visual field, as can be seen in both panels of MeTu4c and MeTu4d. ER3m and ER3a_ad receive input from both MeTu4b and MeTu4d. But the synaptic weight from MeTu4b dominates. Thus, the ventral visual field is not significantly represented in the MeTu4b panel. The interaction between AVP channels appears to be minimal. In other words, direct interaction between the four major MeTu types is negligible between the medulla and the ellipsoid body.

Back-tracing the synaptic pathway starting from an ER4d revealed that its upstream MeTu1 neurons are aligned vertically in the medulla (Supplementary Video 1). They cover about 40° azimuth and the entire vertical span (Fig. 4e). This vertical arrangement of MeTu1 neurons was consistent across ER4d neurons and covered the entire visual field as a population, like an array of vertical bars (Figs. 4e and 5 and Extended Data Fig. 13). It suggests that ER4d neurons are selective to vertically elongated visual stimuli or to the location of visual stimuli along the horizontal plane, regardless of the elevation. Such a pathway would be best suited for detecting visual landmarks that are appropriate references for setting a heading.

By contrast, back-tracing starting from single ER2_ad and ER2_b neurons revealed that they receive information from MeTu3c neurons with dendrites located in the dorsal medulla (Fig. 4e and Supplementary Video 2), whereas individual ER2_c neurons receive inputs exclusively from MeTu3c neurons with dendrites in the central medulla (Supplementary Video 3). Similar to the channel converging into ER4d neurons, the MeTu3c populations upstream of ER2_ad, ER2_b and ER2_c neurons tile the entire visual field. In other words, the ER2 population tiles the visual field two-dimensionally, providing more organizational details of the scene in two dimensions than do ER4d neurons (Fig. 4e).

Furthermore, the roles of ER4d and ER2 in processing chromatic information might differ: Dm2, the main upstream input of MeTu1, receives inputs predominantly from UV-sensitive pale photoreceptors^37^ (81%), which suggests that ER4d neurons could process UV stimuli. On the other hand, Mi15, the main upstream input of MeTu3c, receives input from green-sensitive yellow R8 photoreceptors^37^ (67%), which suggests that ER2 neurons process longer-wavelength stimuli.

Unlike the ER4d and ER2 populations, ER4m and ER5 neurons receive strong input from polarization-sensitive channels involving the DRA-specific MeTu2a (both ER4m and ER5) and MeTu2b (ER5) neurons, respectively. As described before, MeTu2b exhibits more complex connectivity than MeTu2a and, therefore, might encode complex features of polarized light. Hence, ER5 might process more complex features of polarized skylight, whereas ER4m seems to process skylight polarization alone as a navigational cue, consistent with a previous report^11^. This difference is notable because ER5 is involved in circadian rhythms^54,55^.

ER3w receives input from MeTu3a and MeTu3b neurons, potentially combining skylight polarization information (from both MeTu3a and MeTu3b; Extended Data Fig. 6a,b) and localized visual feature information from some of the dorsal visual field (Fig. 4e). On the other hand, most other ER3 subtypes with dendrites in the inferior bulb are downstream of MeTu4 subtypes that do not receive columnar input in the medulla. Thus, until the functions of upstream neurons (Extended Data Fig. 8e) are understood, the visual area and features that these ER3 neurons encode will remain unclear.

Overall, we predict that ER neurons downstream of MeTu1–MeTu3 neurons encode diverse information including polarized light (ER4m, ER5 and ER3w), vertical stimuli or the azimuthal location of visual features (ER4d), and the two-dimensional organization of visual scenes, including azimuth and elevation (ER2), a system suitable for processing both the elevation of a celestial body (for example, the Sun)^56^ and the surrounding two-dimensional environment^7^.

## Physiologically testing predictions

We performed two-photon calcium imaging to test some of these predictions, especially the spatial extent of the visual field to which ER4d and ER2 neurons respond (that is, the receptive field; Fig. 5). We imaged the dendritic calcium activity of ER neurons in the superior bulb while presenting square-shaped dot stimuli to the fly using a projector-based visual stimulation set-up (Fig. 5c–e). Both ER neuron types were excited by ipsilateral visual stimuli, consistent with previous studies^6,9,12,39^ (Fig. 5f,g). The ellipse contours of the receptive fields of ER4d and ER2 tile the visual space differently (Fig. 5h–k). As predicted by our connectomic analysis, the receptive fields of ER4d neurons are vertically elongated (76 ± 13° (1 s.d.) in height and 31 ± 12° in width), and tile the visual space horizontally. The receptive fields of ER2 neurons, on the other hand, are markedly smaller (59 ± 13° in height and 30 ± 9° in width) and tile the visual space in two dimensions. These results precisely match our predictions about the spatial extent of these neurons’ receptive fields. Note that the vertical extent of the ER4d receptive fields is much greater than that reported previously^6^; we believe this to be because of the limited span of elevation in the set-up used previously. By contrast, we tilted the fly head to expose the eyes to more vertical visual span. These results show that informed, systematic predictions can guide experimental designs to reveal previously unexplored dimensions of neural functions.

## Discussion

We observed four essential organization principles of the AVP: (1) a convergence of spatial information from MeTu to TuBu, which suggests the compression of visual information into features with coarse spatial resolution; (2) segregated and parallel processing of spatially overlapping visual features in largely feedforward circuits; (3) parallel pathways for interhemispheric integration, selectively mixing visual features; and (4) divergent feature expansion through TuBu-to-ER connections (Extended Data Fig. 11). Furthermore, by back-tracing from ER neurons to MeTu neurons, we inferred the diverse visual features that compass neurons might use to compute the head direction (Extended Data Fig. 13).

Our analysis of the reconstructed synaptic pathways reveals that the fly’s visual field can be roughly subdivided into three regions: a narrow band in the dorsal-most visual field (DRA)^11,35,50^; the remaining upper visual field (both of these two regions are facing the sky); and the rest of the visual field (the equator and ventral field). Of note, the DRA and upper visual fields are occupied by the large majority of MeTu neuron types with overlapping receptive fields (MeTu1, MeTu2a and MeTu2b, MeTu3a and MeTu3b, and MeTu4a and MeTu4b); by contrast, the lower and frontal visual fields are served by lower numbers of cells and subtypes (innervated by MeTu1, MeTu3c, MeTu4c and MeTu4d). In particular, MeTu4d neurons cover only the ventral half of the visual field. We speculate that this serves to process ventral optic flow^57^ or to orient towards shiny surfaces (for example, water) that produce horizontally polarized reflections^49,58^, which flies detect and use to adjust their body orientation^59,60^. Nine out of the ten parallel information channels formed by MeTu neurons seem to maintain only azimuth information, discarding information about elevation; this strategy seems particularly efficient for computing a heading in azimuth. Only the MeTu3c channel may encode both azimuth and elevation—a property that seems ideal for perceiving the two-dimensional organization of the surrounding environment or for tracking the position of the celestial body across the day. Overall, these organizational patterns of the AVP suggest that *D. melanogaster* prioritizes the azimuthal position of celestial cues, including the skylight polarization pattern.

Animals exhibit specific cue preferences during navigation, and we have long known that visual features are processed hierarchically^16^: Dung beetles prioritize skylight polarization or sun location depending on the environmental context^1^, and mammals prioritize the geometry of the surrounding space^2,3^. However, the field is only just beginning to investigate which visual features are extracted and how they are prioritized across insects^4,43^ and mammals^2^. As such, our complete reconstruction of the AVP in flies is essential for mechanistically understanding the circuit implementation and shared functional principles that underlie the prioritization, integration and transformation of this information into a heading signal. This is exemplified by our experiments that physiologically confirm some of the new predictions. Furthermore, with the ability to dissect detailed circuit dynamics of neural populations using the rich genetic tools in flies, we are poised to gain a deeper understanding of how sensory information is transformed into more abstract representations, which is fundamental to and essential for higher cognitive functions in the brain.

## Methods

### Overview

Our study analysed the FAFB, an adult female *D. melanogaster* brain imaged at the synaptic level resolution with serial section transmission electron microscopy^17^. We used the FlyWire interface, which auto-segmented FAFB EM data to construct three-dimensional segmentations of individual neurons^29^. To reconstruct desired neurons, we first identified relevant axons, dendrites and branches. Possible errors by the auto-segmentation were mainly unfinished branches caused by missing EM slices or incorrect connections caused by shifted EM slices. In addition, some neurons had darker cytosols in the EM data, possibly owing to neuronal damage during the dissection process^61^, and were therefore not as well-constructed by the auto-segmentation. We manually corrected each of these errors.

### Dense EM reconstruction

To find all MeTu, TuBu, TuTu and AOTU046 neurons in the AVP, we densely reconstructed the AOTUsu by scanning through every layer of EM in the neuropil volumes and proofreading all neurons composing them (disregarding twigs). ER neurons were identified by following TuBu downstream connectivity^30^. After each of these neurons was proofread, we classified them and compiled lists of their coordinates for further analysis. See Supplementary Table 1 for details of the editing and naming history.

### Region boundaries

Regions were distinguished in our study so as to limit synapses to specified neuropils. These regions included ME_L, ME_R, LO_L, LO_R, AOTU_L, AOTU_R, BU_L, BU_R and EB (in which ME indicates medulla; BU, bulb; LO, lobula; L, left; R, right; and EB, ellipsoid body). Using SciPy’s spatial module, we created Delaunay tessellations using a set of FlyWire coordinates to determine whether synapses were contained within the given regions. The sets of points were not a comprehensive boundary box of individual neuropils, but rather formed polyhedra that contained the ROIs of the relevant neurons. The coordinates were selected with the help of FlyWire’s annotation lines to ensure that all neurons’ synapses were incorporated. In the case of the medullas, in which the Delaunay tessellation incorporated some lobula synapses as well, the ipsilateral lobula region was subtracted.

### AOTUsu subdivision in comparison with previous studies

The connectome of the AVP, which revealed four major MeTu types, clarifies discrepancies in previous literature. MeTu_im_ in Omoto & Keleş et al.^39^ seems to be MeTu4 because DALcl2d TuBu neurons project from AOTU_im_ to the inferior bulb (BU_i_). In addition, on the basis of spatial organization, AOTU_lc_, AOTU_lp_ and AOTU_la/il_ in Omoto & Keleş et al. might correspond to MeTu1, MeTu2 and MeTu3 locations, respectively, although the AOTUsu map is slightly different from our study (Extended Data Fig. 1d). A crucial discrepancy we could not resolve was TuBu_a_ in Omoto & Keleş et al. They described that TuBu_a_ projects from AOTU_il_ to the anterior BU (BU_a_), which we did not observe in the FAFB dataset. In Hulse, Haberkern, Franconville and Turner-Evans et al.^16^, the TuBu neuron type innervating the BU_a_ is TuBu01, which are located in AOTUsu_PC, downstream of MeTu2. However, Omoto & Keles et al. say that these TuBu neurons project from the AOTUsu_A to the BU_a_. We believe that this discrepancy is due to the lower resolution of light microscopy, and think that TuBu_a_ should be reclassified.

Timaeus et al.^14^ divided the AOTUsu into five subdomains, separating the AOTUsu_A into lateral and anterior central parts. They state that R7 might be upstream of MeTu_la_, MeTu_ca_ and MeTu_cp_ (MeTu3 and MeTu2, respectively), which agrees with what we found. However, they only found TuBu neurons projecting from the AOTUsu_la_, AOTUsu_lp_, AOTUsu_ca_ or AOTUsu_cp_ to the BU_s_ and the AOTUsu_m_ to the BU_i_, meaning that they did not discover TuBu01. Finally, they found that MeTu_m_ (MeTu4) dendrites also projected to medulla layers 2 and 8, which was inconsistent with what we found in the FAFB dataset.

Tai et al.^15^, unlike the other two papers, found four subdomains of the AOTUsu (L-AOTU_1–4_), which are connected linearly from the edge of the AOTUlu to the lateral-most edge of the AOTUsu. The respective MeTu neurons in these regions were called MT_1–4_ (not corresponding to our study’s MeTu1–MeTu4). This study only showed an anterior view of the AOTU, and as such, it is possible that they did not find the AOTUsu_PC, which is obscured by the AOTUsu_A from the anterior side. In this case, the corresponding regions are AOTUsu_M (L-AOTU_1_), AOTUsu_A (L-AOTU_2–3_) and AOTUsc_PL (L-AOTU_4_). The corresponding MeTu neurons are thus MeTu4a, MeTu4b, MeTu4c and MeTu4d (MT_1_), MeTu3c (MT_2_), MeTu3a and MeTu3b (MT_3_) and MeTu1 (MT_4_).

### Synaptic connectivity matrices

Synaptic connectivity between neurons was found using automatic synapse detection^30^. For all our connectivity analyses, we used a cleft score of ≥50 and excluded autapses and synapses to the background segmentation. Two types of connectivity matrices were generated throughout the study: Supplementary Data 1a–g show individual neuron weight matrices (purple) and neural type weight matrices (green). For the individual neuron weight matrices (Supplementary Data 1a–d), the number of synapses between each neuron was first calculated. To determine the relative weight within the given region, this quantity was divided by the postsynaptic neuron’s total number of synapses in the region.

Certain outlier neurons heavily skewed the colour plot matrices because they had few connections in their respective regions or nearly exclusively received synaptic weight from a single neuron. To resolve the former issue, neurons with fewer than five total regional connections were not included in the matrices. To resolve the latter issue, a small number of outliers were removed from medulla MeTu interconnectivity plots: one MeTu1_R, one MeTu2a_R and three MeTu4a_R.

The ordering of the neurons within the connectivity matrices was based on the location of TuBu neurons along the dorsal–ventral axis within the AOTUsu. Both MeTu and ER neurons were ordered in groups according to which of these TuBu neurons they were most connected to (MeTu neurons presynaptically in the AOTUsu and ER neurons postsynaptically in the bulb). Within the groups they were ordered by how many synapses they shared with that TuBu neuron.

Neural type weight matrices (Supplementary Data 1e–g) show the connections of whole classes of neurons. First, the total number of synapses between all presynaptic and postsynaptic neurons of the respective given types was calculated. Then, these quantities were divided by the total number of synapses of all postsynaptic neurons of the given type within the region. This gave a measure of the total synaptic weight between the two types.

### Three-dimensional rendering

Three-dimensional renderings were either generated in Blender with neuron meshes retrieved using the Python CloudVolume package or in R with the rgl and fafbseg package.

### Medulla columns and layers

We identified all Mi1 neurons, a unicolumnar cell type, in both hemispheres as a proxy for individual medulla columns because Mi1 neurons are present in each medulla column and span the entire distal–proximal axis of the medulla from layer M1 to layer M10. For each Mi1 neuron we performed a principal component analysis (PCA) on all pre- and postsynaptic sites of the neuron (Extended Data Fig. 1e). PC1 corresponds to the distal–proximal axis of the column. The upper and lower boundary of each column is defined as the 0.03 and 0.97 percentile of synapses on the distal–proximal axis.

Medulla layers are based on the average synapse distribution of Mi1, Mi4, L1, L2, L3, L5, Dm8 and T4 neurons along the distal–proximal axis in three exemplar columns. Layer M1: [−3.9–5.5%]; layer M2: [5.5–17.1%]; layer M3: [17.1–30.8%]; layer M4: [30.8–34.0%]; layer M5: [34.0–43.2%]; layer M6: [43.2–50.1%]; layer M7: [50.1–63.1%]; layer M8: [63.1–75.4%]; layer M9: [75.4–92.4%]; layer M10: [92.4–102.2%].

### MeTu classification

We describe MeTu types (labelled with numbers: MeTu1–MeTu4) and MeTu subtypes (labelled with lowercase letters; for example, MeTu2a). MeTu1, MeTu2 and MeTu3 were previously called MC61 (ref. ^62^) and MeTu4 was called MC64 (ref. ^16^). MeTu2 was also called MeTu-DRA^37^. The location of axons and dendrites of MeTu (Fig. 1c), TuBu (Fig. 1c), TuTu (Extended Data Fig. 4) and AOTU046 (Extended Data Fig. 4) neurons maintain specific patterns of processes within the AOTUsu^16^, through which we determined four distinct regions (posterior lateral, posterior central, anterior and medial). The axonal boutons of each MeTu neuron terminate within one of these four areas, so we classified MeTu1, MeTu2, MeTu3 and MeTu4 as types. Between the left and right hemispheres, respectively, there are 121 and 124 MeTu1, 50 and 50 MeTu2, 145 and 129 MeTu3 and 137 and 138 MeTu4. There was one neuron on the right side whose axonal tract terminated before projecting to the medulla. It was labelled MeTu_incomplete_R and was excluded from further analysis.

Analysis of morphology, up- and downstream connectivity as well as spatial distribution in the medulla revealed distinct MeTu subpopulations within MeTu2, MeTu3 and MeTu4, which led us to define MeTu subtypes.

MeTu1 forms a homogenous neuron population in terms of morphology, and up- and downstream connectivity, without any distinctive features that would allow any further subtyping (Fig. 2m). MeTu2a is connected to both TuBu01 and TuBu06 with a preference for TuBu01, while MeTu2b is primarily connected to TuBu06 with very few synapses onto TuBu01 (Extended Data Fig. 5k).

We found three MeTu3 subtypes: MeTu3a, MeTu3b and MeTu3c. MeTu3a has flat dendrites and lacks presynaptic connections to Mi15, whereas MeTu3b and MeTu3c have vertical dendritic protrusions and connect to Mi15 (Extended Data Fig. 5d,e). MeTu3a was specifically classified as MeTu3 that has 13 or fewer synapses with Mi15 neurons. Note that MeTu2a and MeTu2b cell bodies are located closer to the medulla equator, whereas MeTu3a cell bodies are found above the centre of the branching (data not shown). Within the AOTUsu, all MeTu3a project to TuBu07. MeTu3b is strongly connected to TuBu07, and MeTu3c is most strongly connected to TuBu09 and TuBu10. To further analyse this distinction, we compared their postsynaptic weights with Mi15, Sm17, Sm23 and MeMeDRA. Some MeTu cells sensitive to skylight polarization have so far been physiologically characterized in *Drosophila*^11^, and a careful comparison between their light microscopic data and our connectomic reconstruction identifies these cells as MeTu2b and MeTu3a. Finally, MeTu3c might have subpopulations: MeTu3c_dorsal and MeTu3c_ventral, on the basis of the TuBu connectivity. Their connectivity in the medulla was indistinguishable other than the general location (dorsal medulla versus ventral medulla), which might suggest the same kind of information processing. Furthermore, the axons to downstream TuBu09 and TuBu10 overlap somewhat, suggesting that functional differences may occur downstream of—but not at—the MeTu3c neurons. For these reasons, we decided to combine them into a single subtype.

MeTu4 is generally morphologically distinct from other MeTu types because neurons contain boutons within the lobula. However, light microscopy suggested there is a subtype that does not have these boutons (Extended Data Fig. 8h). We also found a MeTu4 population without lobula boutons and few lobula synapses (fewer than 15 pre- and postsynapses), which we named MeTu4d. MeTu4d in addition only arborizes within the ventral half of the medulla.

We further grouped MeTu4 neurons with lobula boutons into distinctive subtypes on the basis of downstream TuBu connectivity. MeTu4a are presynaptic to TuBu03 and TuBu04, MeTu4b are presynaptic to TuBu02 and MeTu4c are presynaptic to TuBu05.

UMAP in Fig. 1f (top) is based on connectivity to up- and downstream partners as features. We selected a total of 84 neurons (see ‘Upstream connections’ for more information). Downstream neurons include 13 types (all TuBu types, TuTuA, TuTuB and AOTU046), and upstream neurons include 28 types (all top 5 connected neuron types of all MeTu subtypes. UMAP in Fig. 1f_ii_ is based only on the 31 upstream types (28 non-MeTu and 3 MeTu types). All connectivity types are also shown in Fig. 1g.

As the entire optic lobe connectivity became available (FlyWire v.783)^45^, we also performed the same analysis using the entire dataset in the right hemisphere (Supplementary Data 1h).

Finally, we sought to provide light microscopic evidence in the form of cell-type-specific driver lines, corroborating the existence of the genetically defined subclasses of visual projection neurons that are described in this study^33,34,63,64^ (see Supplementary Table 2).

### Proofreading rounds

For a subset of MeTu neurons described in the previous section, we increased the proofreading quality by increasing the rounds of detailed proofreading^29^. We used the right optic lobe because the left optic lobe has a partially detached lamina and parts of the posterior side of the medulla are distorted^17^. We chose 113 of the 441 right MeTu neurons to undergo multiple rounds of proofreading. Originally, 101 neurons were chosen randomly with the same relative ratios of MeTu1–MeTu4 neurons as in the population: 28 MeTu1, 12 MeTu2, 30 MeTu3 and 31 MeTu4. When we later discovered subcategories of the neurons, we wanted at least five of each subtype. In the end, we proofread the following 113 neurons: 29 MeTu1, 7 MeTu2a, 5 MeTu2b, 6 MeTu3a, 13 MeTu3b, 16 MeTu3c, 13 MeTu4a, 5 MeTu4b, 14 MeTu4c and 5 MeTu4d.

Each of these neurons underwent three rounds of proofreading, and synaptic and skeletal comparisons were performed to determine the differences in accuracy between the three rounds. The first round was the cursory proofreading that was done to all 441 MeTu neurons. The next two rounds were split between the two proofreaders (D.G. and E.K.). Each proofreader densely proofread half of the 113 for the second round, and then switched and worked on the other half for the third round. Afterwards, F1 scores were computed on both the number of synapses and the number of skeletal nodes of each neuron between rounds. These scores showed the decrease in the number of edits between the first round and subsequent rounds.

### Upstream connections

We used automatic synapse detection to find presynaptic partners of the proofread MeTu neurons. As stated in the proofreading section, we picked them on the basis of the ratio of the entire population, with a minimum of five neurons per type. In addition, as with the proofreading rounds, we only looked at neurons on the right side. Because several neurons contain a darker cytosol and are not segmented well in FlyWire, we left out any of those neurons in favour of normal neurons. Thus, we analysed the following 84 neurons: 18 MeTu1, 5 MeTu2a, 5 MeTu2b, 6 MeTu3a, 9 MeTu3b, 11 MeTu3c, 10 MeTu4a, 5 MeTu4b, 10 MeTu4c and 5 MeTu4d. For each neuron, we identified all upstream partners with five or more synapses. Many partners had been classified in previous studies, and for any others we used a nomenclature proposed recently^45^.

### Synapse density

MeTu and TuBu synapse density maps in the AOTUsu were created from three angles: from the dorsal side looking towards the ventral side; from the anterior side looking towards the posterior side; and from the lateral side looking towards the medial side (Extended Data Fig. 3a,b). Each of these views was rotated 30° along the anterior–posterior axis. Each map was created by finding all of the connections within small volumes, each 40 nm by 40 nm by the length of the AOTUsu along the viewpoint axis. When the number of connections was computed, they were subjected to a Gaussian blur with a sigma value of 10. Colour maps were then created on the basis of the relative values, with higher values having higher opacity. Demonstrative synapse maps were created as well (Fig. 1c). These were subjected to a Gaussian blur (with a sigma value of 4), and did not vary in opacity according to synapse density.

### Neurotransmitter predictions

We used the neurotransmitter prediction described in a recent study^51^. We calculated the average neurotransmitter probability across all presynaptic sites of an individual neuron (Extended Data Figs. 4c,e and 12).

### AVP classification

The existing connectomic analysis^16^ of the hemibrain^65,66^ provided full classifications of TuTu, TuBu and ER neurons, which we adopted in this study. This study gave detailed classifications to 17 bulbar ER neurons and 5 lateral accessory lobe ER neurons. Of the 17 bulbar neurons, there are 11 distinct morphologies, and we classified the FAFB neurons as follows: ER2_abd, ER2_c, ER3a_ad, ER3d_acd, ER3d_b, ER3m, ER3p_ab, ER3w. ER4d, ER4m and ER5. The study also described patterns of interconnectivity between ER neurons, and using synaptic analysis we distinguished ER2ad and ER2b, and ER3d_a, ER3d_c and ER3d_d. There are multiple morphologies of ER2c neurons (which is consistent with hemibrain), but we did not further subcategorize these neurons. However, some connectivity patterns are not consistent between the hemibrain and FAFB, so we did not subclassify all neurons to the same level of detail. In the instances of ER2_a and ER2_d; ER3a_a and ER3a_d; ER3p_a and ER3p_b; and ER3w_a and ER3w_b, we maintained their names as ER2_ad, ER3a_ad, ER3d_acd, ER3p_ab and ER3w_ab.

In the hemibrain, TuBu neurons were classified on the basis of their downstream ER neuron partners. After classifying all the corresponding ER neurons, we similarly grouped the TuBu neurons as TuBu01–TuBu10. There are three ambiguous TuBu neurons. One TuBu in the right hemisphere is upstream of an ER2c neuron but is located in line with other TuBu09 as opposed to TuBu10, which are generally upstream of ER2c. We labelled this neuron TuBu09 because of its location in AOTUsu. Another TuBu neuron in the right hemisphere has the dendritic morphology of a TuBu04 and is downstream of MeTu4a, but is upstream of ER3p_ab. We classified it as TuBu04 as opposed to TuBu05. One neuron in the left hemisphere has a normal microglomerulus partnered with an ER3a_ad and two ER3m neurons like TuBu02 neurons. However, this neuron projects to the SPS, as opposed to the AOTUsu. Because there is no other neuron in this dataset or hemibrain with this projection pattern, we determined that it might have been a developmental error and labelled it TuBu_misc_L, only including it in connectivity tables between TuBu and ER neurons.

We identified TuTub_a and TuTub_b on the basis of morphology. There is one of each type per hemisphere. There are four AOTU046 neurons, with dendrites in one SPS and axons in both AOTUsu and both bulbs. The quantity of each of these bihemispheric neurons is consistent with hemibrain^16^.

### Bihemispheric connections

Connectivity diagrams of bihemispheric neurons are based on type connectivity matrices from the right hemisphere (Extended Data Fig. 4a). Each arrow represents the weight of the postsynaptic neuron type’s connection to the presynaptic neuron type. Only weights ≥0.05 were represented as arrows. Arrow thickness was determined linearly on the basis of the weight.

The bihemispheric neuron diagrams in Extended Data Fig. 4b,d are made using neurons from the right hemisphere. Pie charts within the figures show the relative amount of presynaptic (red) and postsynaptic (cyan) connections of the neuron. Within the AOTUsu, these only include connections between the bihemispheric neurons and MeTu and TuBu neurons. Within the bulb, the connections shown are between AOTU046 and TuBu and ER neurons. Within the SPS, the connections are between AOTU046 and all SPS neurons. The relative size of the pie charts refers to the quantities of bihemispheric synapses in each subregion. In the case of AOTU046, these were calculated by averaging the two neurons on the right side. Lines are drawn to subregions that have 100 or more synapses.

### Alternative visual pathways

To identify other potential visual pathways, we looked up to two hops upstream of central complex input neurons (Extended Data Fig. 2). We excluded neurons intrinsic to the central complex. For upstream partners, we included neurons that had at least five synapses with their downstream partner. After finding all neurons one to two hops upstream of the central complex input neurons, we determined which of them contained dendrites within optic lobe neuropils. This number of hops was selected to match the number of hops of the direct pathway from MeTu to TuBu to ER neurons. When calculating optic lobe weights, we summed the relative proportion of synapses that came from those optic lobe neurons. Renders of alternate pathways included the central complex neurons, upstream neurons that are either optic lobe neurons or neurons with upstream optic lobe partners, and those upstream optic lobe partners. The renders only contained these neurons if they accounted for ≥1% of the total non-central complex synaptic weight of their postsynaptic partner.

### Mapping medulla columns to ommatidia coordinates

We used microCT data^53^ to assign each medulla column to an ommatidia. We determined the directions of ommatidia to the world as previously described^53^. Separately, we counted the numbers of R1–R6 photoreceptors in the lamina to identify the equatorial medulla columns, which have seven or eight photoreceptors compared to six in non-equatorial columns (data not shown). We then mapped ommatidia and medulla columns to separate hexagonal grids. Finally, using the equator as a reference, we aligned these hexagonal grids by minimizing unmatched points, hence assigning each medulla column a viewing (or sampling) direction in the visual field.

### ER neuron visual area

For each ‘visual column’ of a given ER neuron, we calculate the values for the direct pathway as the sum of all weighted branches connecting the ER neuron via TuBu and MeTu neurons to the ‘visual column’ (Fig. 4a, middle). Each branch is the product of synaptic weights of TuBu neuron to ER neuron connection, MeTu neuron to TuBu neuron connection and MeTu neuron medulla column occupancy. MeTu neuron medulla column occupancy is calculated as the fraction of presynaptic sites closed to the column. The values for the indirect pathway are the sum of all weighted branches connecting the ER neuron via TuBu, TuTuB and MeTu neurons to the ‘visual column’ (Fig. 4b, middle). We overlaid the values for the direct and the indirect pathways with different colours (Fig. 4c,e).

### Hemibrain comparison

The hemibrain dataset contains the entire central complex of the *D. melanogaster* brain, but only extends to include the AOTU of the left hemisphere. Therefore, it contains two sets of ER neurons, one set of TuBu neurons and only the boutons of one set of MeTu neurons. The MeTu neurons were named MC64 or MC61.

We used the Python module neuprint-python to look at the MC61 and MC64 that are presynaptic to the previously defined TuBu neurons. We first distinguished MeTu1–MeTu4 on the basis of their respective TuBu types. All MC64 are MeTu4, but a small population of MeTu4 was MC61 instead. We plotted the number of synapses within the lobula among MC61 and MC64 to determine that this distinction was due to differences in the number of lobula connections (data not shown). We distinguished MeTu4 in the same way as for FAFB, in which fewer than 15 synapses in the lobula denoted MeTu4d. We classified all other MeTu subtypes using their downstream TuBu partners. The only classification we were unable to make was that of MeTu3a and MeTu3b, because they were separated using upstream connections in the medulla, which the dataset did not include. We labelled these neurons MeTu3ab, and adjusted the FAFB one in comparison plots. After classification, there are 127 MeTu1, 39 MeTu2a, 14 MeTu2b, 64 MeTu3ab, 86 MeTu3c, 68 MeTu4a, 13 MeTu4b, 41 MeTu4c and 17 MeTu4d.

After obtaining all AVP neurons in FlyWire and neuprint, we compared the relative numbers of neurons among four hemispheres with ER and bihemispheric neurons, and three hemispheres with TuBu and MeTu neurons. We noticed discrepancies among TuBu and ER neuron counts, so we compared the ratios of ER to TuBu counts in the three hemispheres (Extended Data Fig. 9e,f).

### Histology

To support the EM-based cell typing and to provide genetic tools for future studies of MeTu neurons, we report several split-GAL4 lines with preferential expression in subsets of MeTu types. These split-GAL4 lines were generated before the EM work; that is, are the output of an independent, light-microscopy-based effort to characterize MeTu cell types but are newly reported here. Driver lines and their candidate EM matches are listed in Supplementary Table 2. Figures show processed images displayed using VVD viewer (https://github.com/JaneliaSciComp/VVDViewer/releases). Lines and original image data are available at https://splitgal4.janelia.org/cgi-bin/splitgal4.cgi. Some lines are not currently maintained as stocks but can be reconstructed from the AD and DBD hemidrivers.

The general strategy and methods to generate and characterize these lines were as described for other split-GAL4 lines^33,63^. In brief, we searched for GAL4 lines with expression in MeTu cells using images of GAL4 driver expression patterns^67–69^, then screened the expression patterns of hemidriver (AD, DBD) combinations selected to target candidate MeTu types on the basis of these searches, and constructed stable fly lines for combinations with patterns of interest. Further characterization of these lines included imaging of overall expression patterns and, in most cases, of MCFO-labelled individual cells. Sample preparation and imaging, performed by the FlyLight Project Team using protocols available at https://www.janelia.org/project-team/flylight/protocols, were as in previous work^34,70^. For an overview of the Janelia FlyLight split-GAL4 project, see a previous study^64^. Some additional MeTu driver lines that are not included here are also available^47^.

### Fly preparation for calcium imaging

All experiments were performed with seven-to-nine-day-old female flies. Blinding was impossible, and thus was not performed, because the morphology of the neurons is easily recognizable during imaging for each type of neurons. We did not perform sample size calculation but collected data from a fixed predetermined number of flies. Before experiments, flies were prepared as previously described^6^. In brief, a fly was anaesthetized on ice and transferred to a cold plate. The proboscis of the fly was pressed into the head and fixed with wax. In addition, the front and middle pairs of legs were removed. The fly was glued to a pin and positioned in a pyramid-shaped holder. We tilted the fly head 30–45° to the left and glued it to the holder with UV-curable gel. Next, we removed the cuticle on the head, together with ocelli and trachea, to expose the central brain for optical access. Muscle 16 was severed using a dissection needle to reduce brain movement. To further stabilize the brain and minimize motion, we covered the exposed brain with around 3 µl of saline with 3% low-gelling point agarose (Sigma-Aldrich), which was adopted from an imaging procedure of *Drosophila* Kenyon cells^71^. The brain was bathed in saline, as described in previous studies^5^, with an adjusted calcium concentration at 2.7 mM. The fly was then transferred to the microscope for recording.

### Projector-based visual stimulation set-up

Visual stimuli were rear-projected onto a Teflon screen (0.254 mm thick, McMaster-Carr, item 8569K18) placed at the anterior-left-ventral side of the fly with a 45° inclination. We used a customized projector (Texas Instruments) with filters (AVR) to display blue stimuli with a wavelength peak at 450 nm. The stimuli were displayed at a frame rate of 60 Hz and a resolution of 1,028 × 960. A photodiode was placed on the edge of the screen to detect a small flashing square for synchronization between the visual stimuli and calcium activity^72^. Visual stimuli were drawn and displayed by Psychtoolbox-3.

### Two-photon calcium imaging

We used a custom-built two-photon scanning microscope (Janelia MIMMS 2.0) with a 40× objective (Nikon, NA 0.8, 3.5 mm WD). We used a Chameleon laser tuned to 930 nm with a maximum power of 10 mW for excitation and detected fluorescence by a GaAsP photomultiplier tube. We imaged the superior bulb over 15 focal planes, each separated by 1 µm, acquired at a volume rate of about 10 Hz at a resolution of 128 × 128.

### Calcium image analysis

All data processing and analyses were performed in MATLAB. We corrected for brain movement in *xy* directions by registering individual frames to the reference image using a cross-correlation algorithm. We generated reference images in Fiji by summing up images across the time series. We manually identified individual microglomeruli as an ROI on the basis of the videos of fluorescence changes, and we subtracted the mean fluorescence of an empty ROI from the same frame for each frame to compensate for noise. We defined the baseline fluorescence (*F*_0_) as the lowest 10% signal throughout the experiment. Because each microglomerulus does not occupy the entire depth of 15 planes in a volume, we averaged fluorescence from three consecutive planes and used the strongest value among 13 values in each volume as *F*, to calculate the Δ*F*/*F*_0_.

### Receptive field analysis

The fly was presented with a square-shaped dot for 1 s followed by 1 s of darkness. The dot was randomly chosen from a set of 38 pre-indexed dots, which were 18° by 18° each in size and were not spatially overlapping. Each dot was tested for ten trials. In each trial, we calculated the response by subtracting the mean Δ*F*/*F* during the 1-s stimulation period from the mean Δ*F*/*F* during the 500 ms before a dot appeared. We used the average response over ten trials to plot the receptive field. If the average response had either a *P* value higher than 0.05 in the Wilcoxon signed-rank test or a change smaller than the response of an empty ROI, we considered it unresponsive. We quantified the properties of an excitatory receptive field by fitting an ellipse into it by using the regionprops function in MATLAB.

### Reporting summary

Further information on research design is available in the Nature Portfolio Reporting Summary linked to this article.

## Online content

Any methods, additional references, Nature Portfolio reporting summaries, source data, extended data, supplementary information, acknowledgements, peer review information; details of author contributions and competing interests; and statements of data and code availability are available at 10.1038/s41586-024-07967-z.

## Supplementary information

Supplementary Data 1 Connectivity charts and categorization of entire MeTu neurons. **a**_**i–viii**_, Synaptic weight plots of MeTu interconnectivity in the medulla. Plots alternate right and left for MeTu1-4. **b**_**i–viii**_, Synaptic weight plots of MeTu interconnectivity in the AOTUsu. Plots are ordered the same way as in (**a**). **c**_**i–viii**_, Synaptic weight plots of MeTu to TuBu connections in the AOTUsu. **d**_i**–viii**_, Synaptic weight plots of TuBu to ring neuron connections in the bulb. **e**, Type weight map of neurons in the ellipsoid body, including all ER (visual and non-visual), ExR, EPG, PEN, PEG, EL, and EPGt neurons. **f**_**i,ii**_, Type weight maps of bihemispheric neurons, MeTu, and TuBu neurons in the AOTUsu_R (**f**_**i**_) and AOTUsu_L (**f**_**ii**_). **g**_**i,ii**_, Type weight maps of AOTU046, TuBu, and visual ER neurons in the BU_R (**g**_**i**_) and BU_L (**g**_**ii**_). **h**_**i**_, UMAP analysis of entire MeTu neurons in the right hemisphere based on connectivity in both inputs in medulla and outputs to TuBu neurons in AOTUsu. **h**_**ii**_, The same analysis only based on the medulla connectivity. **h**_**iii**_, Only based on the AOTUsu connectivity to TuBu. **i**, Connectivity dendrogram of the entire MeTu population. **j**_**i**_, Connectivity dendrogram of the entire MeTu1 population. **j**_**ii**_, Connectivity dendrogram of the entire MeTu2 population. **j**_**iii**_, Connectivity dendrogram of the entire MeTu3 population. Note that some of the MeTu3c were grouped with MeTu3b because MeTu3b and 3c share many similarities. **j**_**iv**_, Connectivity dendrogram of the entire MeTu4 population.

Reporting Summary

Supplementary Data 2 A gallery of all MeTu_R neurons and their receptive fields. This file contains information on all right MeTu neurons (n=441). Each page has a render of the MeTu from an anterior view in the brain, a view from the top with its relevant medulla columns, a view from the side with medulla layers, locations of its presynaptic (red) and postsynaptic (cyan) connections, and its putative receptive field. At the top is a label of the MeTu subtype and its FlyWire ID during materialization 783.

Supplementary Data 3 A gallery of all ER_R putative receptive fields. Each page contains a collection of right ring neurons of a given type, and shows their putative receptive fields. Included are their types and FlyWire IDs during materialization 783.

Supplementary Data 4 Eye map. A csv file that contains every Mi1 neuron in both hemisphere. For each of them, their coordinates and point within layer M6 is included.

Supplementary Table 1 Editing and naming history of reconstructed neurons. ‘FlyWire Consortium Edit Record’ Sheet: a record of the relative amount that each user edited each neuron used in the paper. Labs are credited for editing each neuron if they contributed ≥10% of edits for that neuron. ‘Codex Naming History’ Sheet: every annotation that has been given to every neuron looked at in the paper. Included are the annotations, and time, user, and affiliation of each annotation.

Supplementary Table 2 Key resources table.

Supplementary Video 1 Back-tracing from an ER4d neuron to MeTu1 neurons. From this reconstruction, we infer that ER4d neurons respond to vertically elongated visual fields.

Supplementary Video 2 Back-tracing from an ER2b neuron to MeTu3c neurons. From this reconstruction, we infer that ER2b neurons respond to smaller dorsal visual fields.

Supplementary Video 3 Back-tracing from an ER2c neuron to MeTu3c neurons. From this reconstruction, we infer that ER2c neurons respond to smaller visual fields around the equator.

## Source data

Source Data Fig. 5

## Figures and Tables

**Extended Data Fig. 7:**
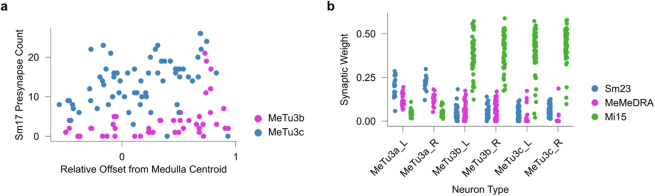
MeTu3 upstream connections. **a**, Number of synapses from Sm17 onto MeTu3b (pink) and MeTu3c (blue) neurons in the right hemisphere as a function of their relative position along the dorsal–ventral axis. 0 on the *x* axis refers to the centre of the medulla, while positive values are more dorsal and negative values are more ventral. **b**, Synaptic weight of upstream neurons to all neurons of the different subtypes of MeTu3. MeTu3a_L: n = 20, MeTu3a_R: n = 19, MeTu3b_L: n = 53, MeTu3b_R: n = 46, MeTu3c_L: n = 72, MeTu3c_R: n = 64.

## Data Availability

All raw data (FAFB) are available at FlyWire.ai (v.783) or Codex: FlyWire. Supplementary Data 2 and 3 provide neuron IDs. Supplementary Data 4 provides a microCT-based eye map. Because of the data size, the raw two-photon calcium imaging data will be available upon request. Source data for Fig. 5 are provided with this paper.

## Code availability

Analysis code can be downloaded from 10.5281/zenodo.13228138 (ref. ^73^).
