# Supplementary material for: Connectomic reconstruction predicts visual features used for navigation
Source: Nature. 2024 Oct 2;634(8032):181–90. doi: 10.1038/s41586-024-07967-z (PMC11446847; doi:10.1038/s41586-024-07967-z)
Supplement: Supplementary file 1 — Connectivity charts and categorization of entire MeTu neurons. ai–viii, Synaptic weight plots of MeTu interconnectivity in the medulla. Plots alternate right and left for MeTu1-4. bi–viii, Synaptic weight plots of MeTu interconnectivity in the AOTUsu. Plots are ordered the same way as in (a). ci–viii, Synaptic weight plots of MeTu to TuBu connections in the AOTUsu. di–viii, Synaptic weight plots of TuBu to ring neuron connections in the bulb. e, Type weight map of neurons in the ellipsoid body, including all ER (visual and non-visual), ExR, EPG, PEN, PEG, EL, and EPGt neurons. fi,ii, Type weight maps of bihemispheric neurons, MeTu, and TuBu neurons in the AOTUsu_R (fi) and AOTUsu_L (fii). gi,ii, Type weight maps of AOTU046, TuBu, and visual ER neurons in the BU_R (gi) and BU_L (gii). hi, UMAP analysis of entire MeTu neurons in the right hemisphere based on connectivity in both inputs in medulla and outputs to TuBu neurons in AOTUsu. hii, The same analysis only based on the medulla connectivity. hiii, Only based on the AOTUsu connectivity to TuBu. i, Connectivity dendrogram of the entire MeTu population. ji, Connectivity dendrogram of the entire MeTu1 population. jii, Connectivity dendrogram of the entire MeTu2 population. jiii, Connectivity dendrogram of the entire MeTu3 population. Note that some of the MeTu3c were grouped with MeTu3b because MeTu3b and 3c share many similarities. jiv, Connectivity dendrogram of the entire MeTu4 population. [file 41586_2024_7967_MOESM1_ESM.pdf]

nature portfolio

https://doi.org/10.1038/s41586-024-07967-z

Supplementary information

Connectomic reconstruction predicts visual features used for navigation

In the format provided by the authors and unedited

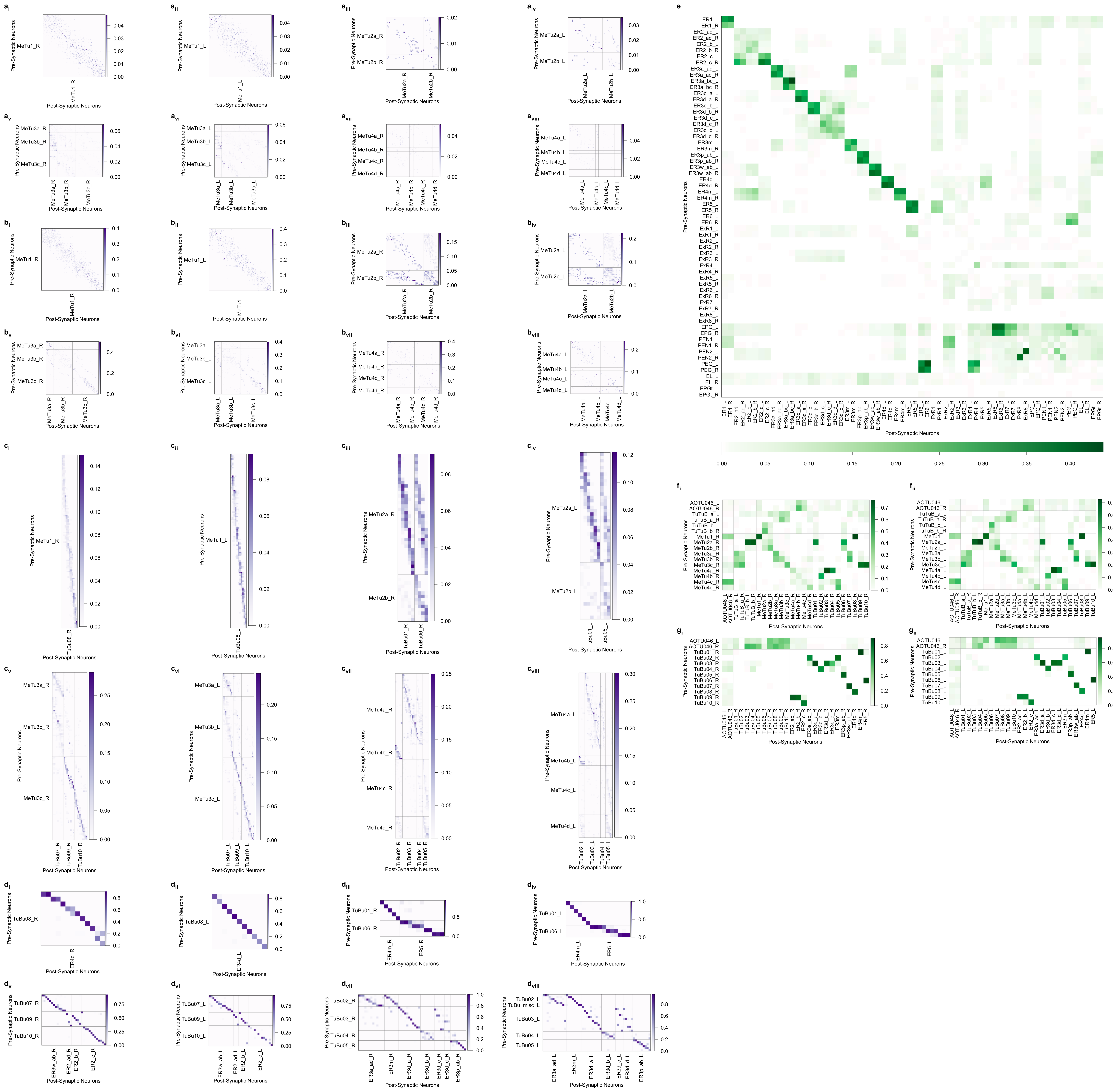

ai
MeTu1_R
Pre-Synaptic Neurons
Post-Synaptic Neurons
aii
MeTu1_L
aiii
MeTu2a_R
MeTu2b_R
aiv
MeTu2a_L
MeTu2b_L
av
MeTu3a_R
MeTu3b_R
MeTu3c_R
avi
MeTu3a_L
MeTu3b_L
MeTu3c_L
avii
MeTu4a_R
MeTu4b_R
MeTu4c_R
MeTu4d_R
aviii
MeTu4a_L
MeTu4b_L
MeTu4c_L
MeTu4d_L
bi
bii
biii
biv
bv
bvi
bvii
bviii
ci
TuBu08_R
cii
TuBu08_L
ciii
TuBu01_R
TuBu06_R
civ
TuBu01_L
TuBu06_L
cv
TuBu07_R
TuBu09_R
TuBu10_R
cvi
TuBu07_L
TuBu09_L
TuBu10_L
cvii
TuBu02_R
TuBu03_R
TuBu04_R
TuBu05_R
cviii
TuBu02_L
TuBu03_L
TuBu04_L
TuBu05_L
di
ER4d_R
dii
ER4d_L
diii
ER4m_R
ER5_R
div
ER4m_L
ER5_L
dv
ER3w_ab_R
ER2_ad_R
ER2_b_R
ER2_c_R
dvi
ER3w_ab_L
ER2_ad_L
ER2_b_L
ER2_c_L
dvii
ER3a_ad_R
ER3m_R
ER3d_a_R
ER3d_b_R
ER3d_c_R
ER3d_d_R
ER3p_ab_R
dviii
TuBu_misc_L
ER3a_ad_L
ER3m_L
ER3d_a_L
ER3d_b_L
ER3d_c_L
ER3d_d_L
ER3p_ab_L
e
fi
fii
gi
gii
AOTU046_L
AOTU046_R
TuTuB_a_L
TuTuB_a_R
TuTuB_b_L
TuTuB_b_R

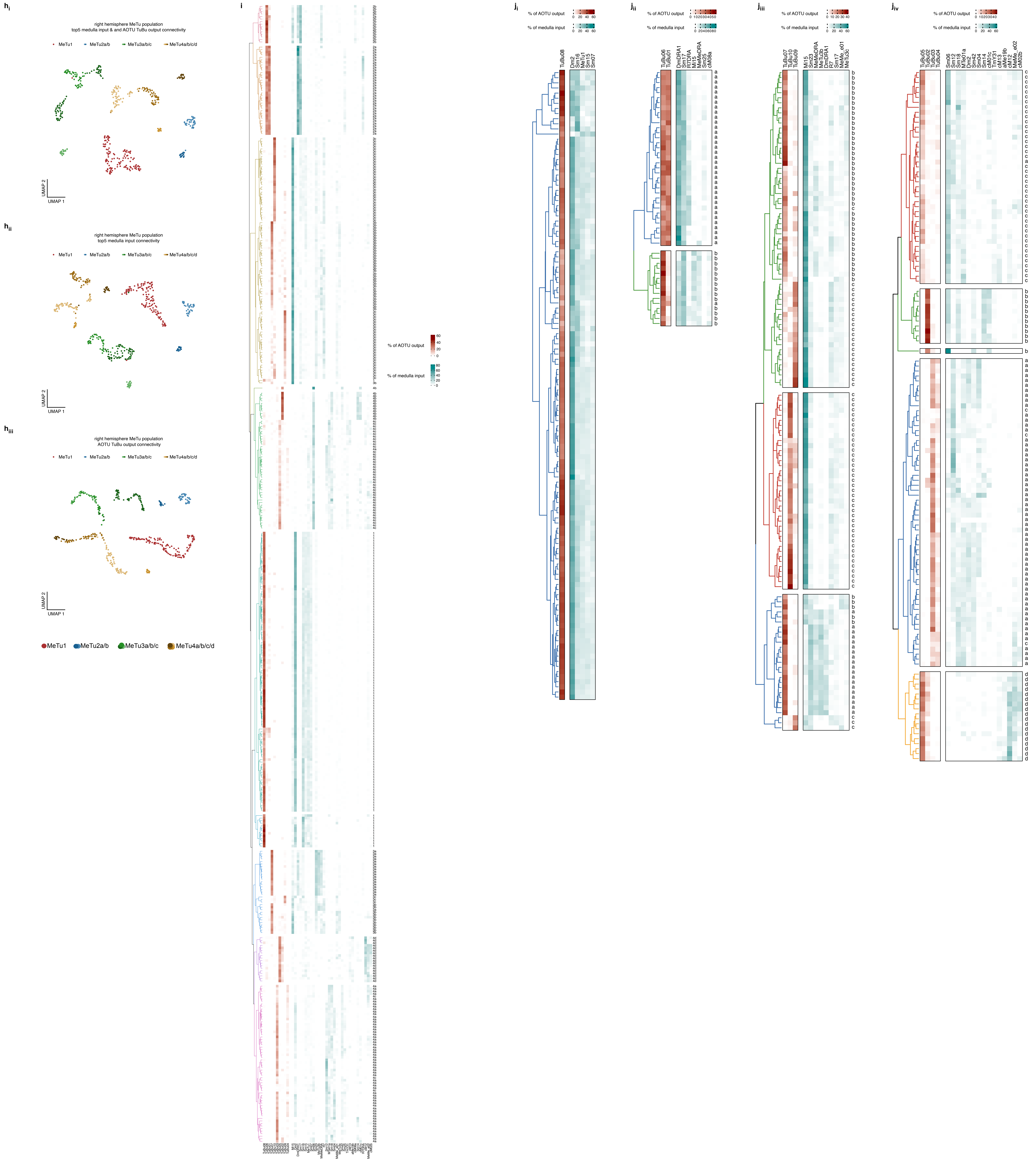
hi
right hemisphere MeTu population
top5 medulla input & and AOTU TuBu output connectivity
MeTu1
MeTu2a/b
MeTu3a/b/c
MeTu4a/b/c/d
UMAP 2
UMAP 1
hii
right hemisphere MeTu population
top5 medulla input connectivity
hiii
right hemisphere MeTu population
AOTU TuBu output connectivity
i
% of AOTU output
% of medulla input
ji
jii
jiii
jiv
TuBu08
Dm2
Sm16
MeTu1
Sm15
Sm07
TuBu06
TuBu01
DmDRA1
Sm17
R7DRA
Mi15
MeMeDRA
Sm25
cM08a
TuBu07
TuBu10
TuBu09
Mi15
Sm23
MeMeDRA
MeTu3b
DmDRA1
R7
Sm17
MeMe_e01
MeTu3c
TuBu05
TuBu02
TuBu03
TuBu04
Sm06
Sm12
Sm18
MTe01a
Dm2
Sm42
Sm04
Sm14
cM01c
TmY31
cM13
aMe19b
cM12
MeMe_e02
cM02b
