# Supplementary material for: Connectomic reconstruction predicts visual features used for navigation
Source: Nature. 2024 Oct 2;634(8032):181–90. doi: 10.1038/s41586-024-07967-z (PMC11446847; doi:10.1038/s41586-024-07967-z)
Supplement: Supplementary file 2 — Reporting Summary [file 41586_2024_7967_MOESM2_ESM.pdf]

nature portfolio

Corresponding author(s): Mathias F. Wernet
Sung Soo Kim

Last updated by author(s): Jun 10, 2024

Reporting Summary

Nature Portfolio wishes to improve the reproducibility of the work that we publish. This form provides structure for consistency and transparency in reporting. For further information on Nature Portfolio policies, see our Editorial Policies and the Editorial Policy Checklist.

Statistics

For all statistical analyses, confirm that the following items are present in the figure legend, table legend, main text, or Methods section.

n/a	Confirmed	
☐	☒	The exact sample size (*n*) for each experimental group/condition, given as a discrete number and unit of measurement
☐	☒	A statement on whether measurements were taken from distinct samples or whether the same sample was measured repeatedly
☐	☒	The statistical test(s) used AND whether they are one- or two-sided<br>*Only common tests should be described solely by name; describe more complex techniques in the Methods section.*
☐	☒	A description of all covariates tested
☐	☒	A description of any assumptions or corrections, such as tests of normality and adjustment for multiple comparisons
☐	☒	A full description of the statistical parameters including central tendency (e.g. means) or other basic estimates (e.g. regression coefficient) AND variation (e.g. standard deviation) or associated estimates of uncertainty (e.g. confidence intervals)
☐	☒	For null hypothesis testing, the test statistic (e.g. *F*, *t*, *r*) with confidence intervals, effect sizes, degrees of freedom and *P* value noted<br>*Give P values as exact values whenever suitable.*
☒	☐	For Bayesian analysis, information on the choice of priors and Markov chain Monte Carlo settings
☒	☐	For hierarchical and complex designs, identification of the appropriate level for tests and full reporting of outcomes
☒	☐	Estimates of effect sizes (e.g. Cohen's *d*, Pearson's *r*), indicating how they were calculated

Our web collection on statistics for biologists contains articles on many of the points above.

Software and code

Policy information about availability of computer code

Data collection	EM data: We used publicly available data. Physiology: We used Scanimage 2023 to collect calcium imaging data.
Data analysis	We used Python 3, pymaid, FAFBseg, R, R Studio, Natverse, Tidyverse, alphashape3d, FAFB, and FlyWire for EM data analyses. We used Matlab for calcium imaging data analyses. Custom code for this work is provided at : DOI: 10.5281/zenodo.13228138

For manuscripts utilizing custom algorithms or software that are central to the research but not yet described in published literature, software must be made available to editors and reviewers. We strongly encourage code deposition in a community repository (e.g. GitHub). See the Nature Portfolio guidelines for submitting code & software for further information.

Data

Policy information about availability of data

All manuscripts must include a data availability statement. This statement should provide the following information, where applicable:

- Accession codes, unique identifiers, or web links for publicly available datasets
- A description of any restrictions on data availability
- For clinical datasets or third party data, please ensure that the statement adheres to our policy

All raw data (FAFB) is available at flywire.ai (v783) or codex.flywire.ai. SI Data Sets 2 and 3 provide neuron IDs. SI Data Set 4 provides micro-CT based eye map. Because of the data size, the raw two-photon calcium imaging data will be available upon request.

Research involving human participants, their data, or biological material

Policy information about studies with human participants or human data. See also policy information about sex, gender (identity/presentation), and sexual orientation and race, ethnicity and racism.

Reporting on sex and gender	N/A
Reporting on race, ethnicity, or other socially relevant groupings	N/A
Population characteristics	N/A
Recruitment	N/A
Ethics oversight	N/A

Note that full information on the approval of the study protocol must also be provided in the manuscript.

Field-specific reporting

Please select the one below that is the best fit for your research. If you are not sure, read the appropriate sections before making your selection.

☒ Life sciences ☐ Behavioural & social sciences ☐ Ecological, evolutionary & environmental sciences

For a reference copy of the document with all sections, see nature.com/documents/nr-reporting-summary-flat.pdf

Life sciences study design

All studies must disclose on these points even when the disclosure is negative.

Sample size	n=1 (one EM dataset). We analyzed the electron microscopy data from both hemispheres of a single female Drosophila brain. We compared the result to another female brain and male brain when possible. Two photon calcium imaging was performed in female flies for two genotypes, each with 11 flies (no sample size calculation was performed but we preset this number before performing any statistical analyses).
Data exclusions	No data was excluded.
Replication	Data from two hemispheres were compared. This result was also compared to the data from one hemisphere of another brain, which was performed independently by another group (Hulse et al. 2021). Because n=1 for each case, the success of replication cannot be meaningfully addressed. Instead, the details of comparisons in this replication effort is described in the main text. The physiological results were replicated in four separate experiments (not included in this work). All replications showed consistent results, thus successfully reproduced the results described in the study.
Randomization	The study is based on n=1 (one EM dataset). Thus, randomization is impossible. The order of visual stimuli presented to flies in the calcium imaging were fully randomized.
Blinding	The study is based on n=1 (one EM dataset). Thus, blinding is impossible. Blinding the calcium imaging was impossible because neurons are recognizable by morphology.

Reporting for specific materials, systems and methods

We require information from authors about some types of materials, experimental systems and methods used in many studies. Here, indicate whether each material, system or method listed is relevant to your study. If you are not sure if a list item applies to your research, read the appropriate section before selecting a response.

Materials & experimental systems

n/a	Involved in the study
☒	☐ Antibodies
☒	☐ Eukaryotic cell lines
☒	☐ Palaeontology and archaeology
☐	☒ Animals and other organisms
☒	☐ Clinical data
☒	☐ Dual use research of concern
☒	☐ Plants

Methods

n/a	Involved in the study
☒	☐ ChIP-seq
☒	☐ Flow cytometry
☒	☐ MRI-based neuroimaging

Animals and other research organisms

Policy information about studies involving animals; ARRIVE guidelines recommended for reporting animal research, and Sex and Gender in Research

Laboratory animals	Drosophila melanogaster. EM data: 7-day old adult female flies (see Zheng et al. 2018 for more details). Physiology: 7-9 days old female flies.
Wild animals	No wild animals were used in this study.
Reporting on sex	Females were used in physiological experiments
Field-collected samples	The study did not use any field-collected samples.
Ethics oversight	The study did not require an ethical approval.

Note that full information on the approval of the study protocol must also be provided in the manuscript.

Plants

Seed stocks	N/A
Novel plant genotypes	N/A
Authentication	N/A
