# Supplementary figures and images for: Connectomic reconstruction predicts visual features used for navigation
Source: Nature. 2024 Oct 2;634(8032):181–90. doi: 10.1038/s41586-024-07967-z (PMC11446847; doi:10.1038/s41586-024-07967-z)

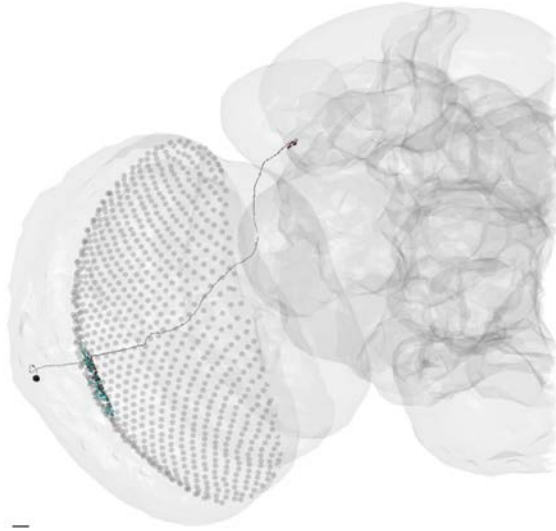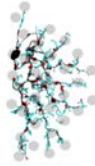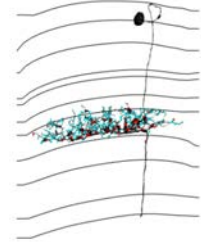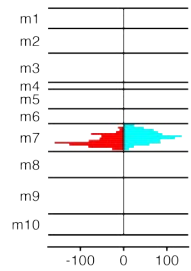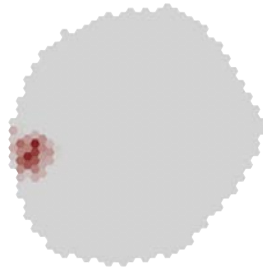

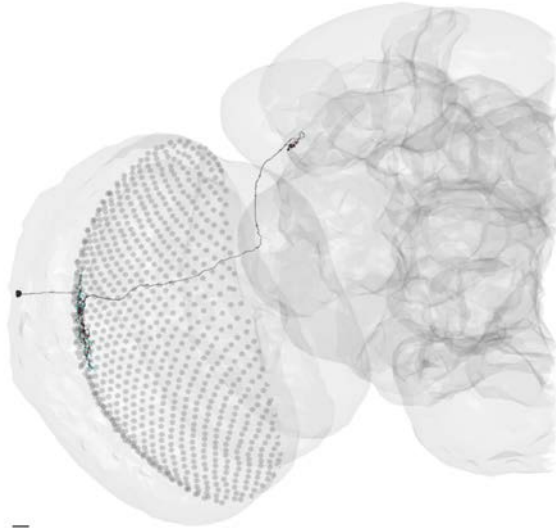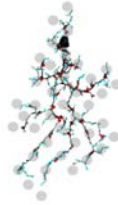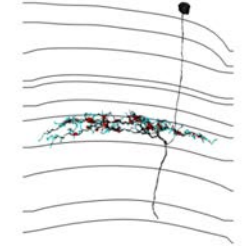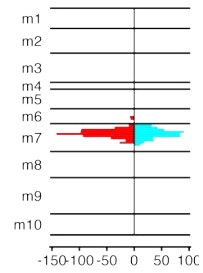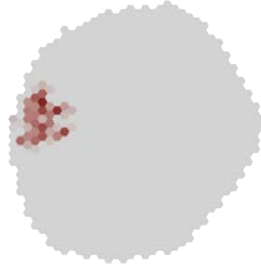

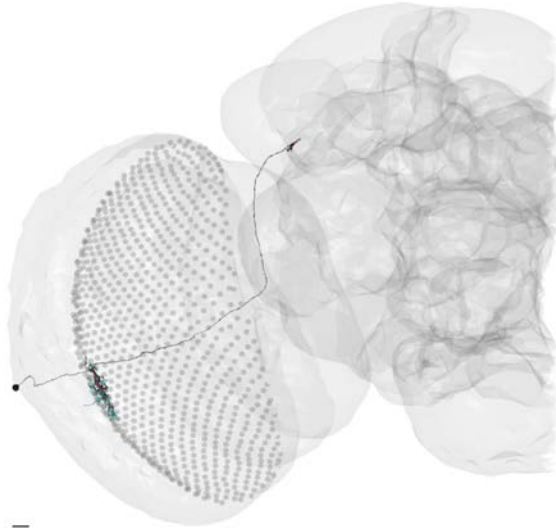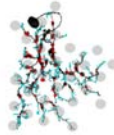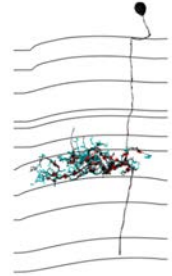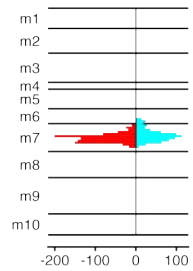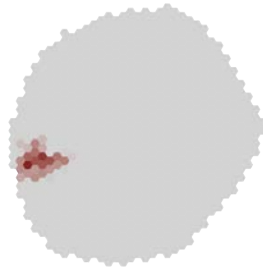

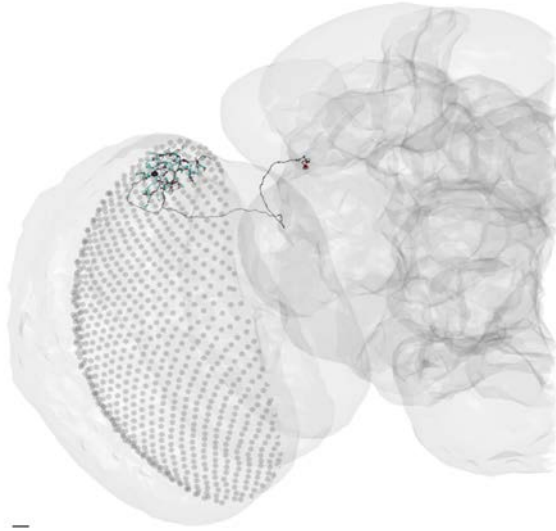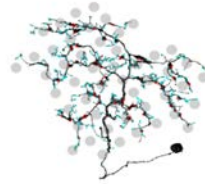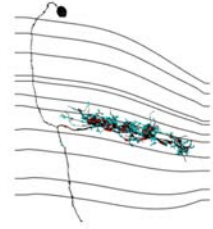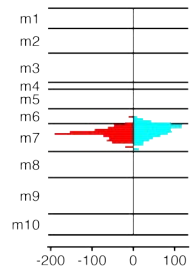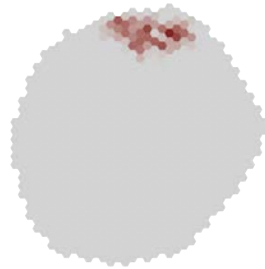

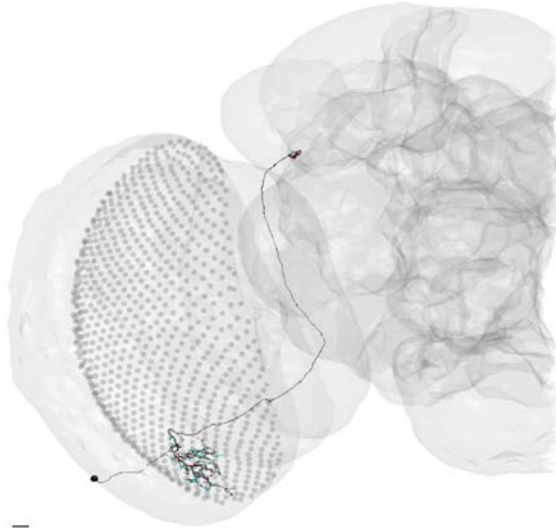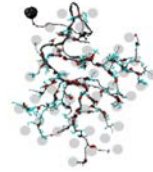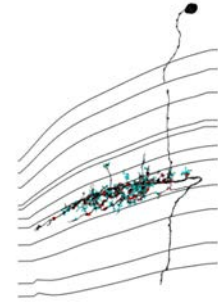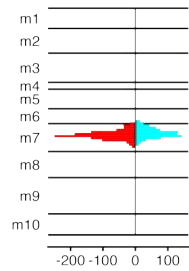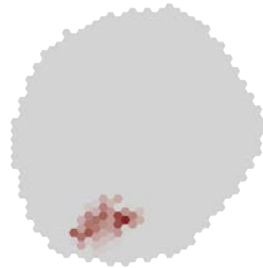

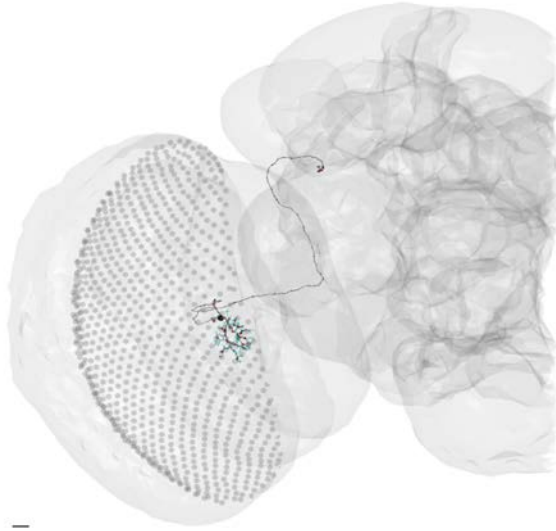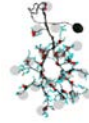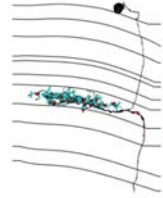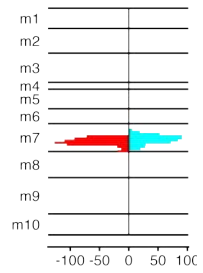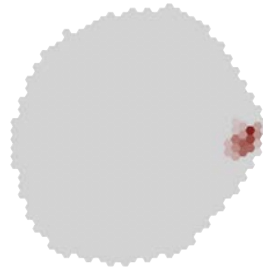

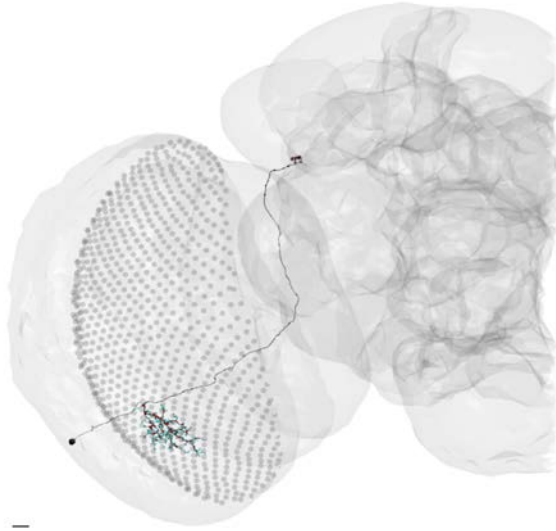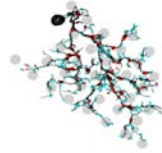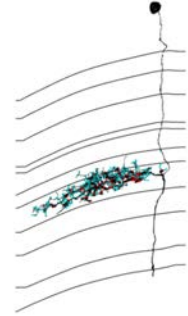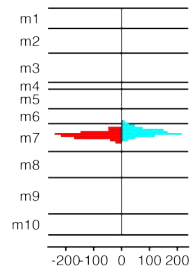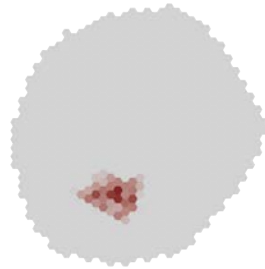

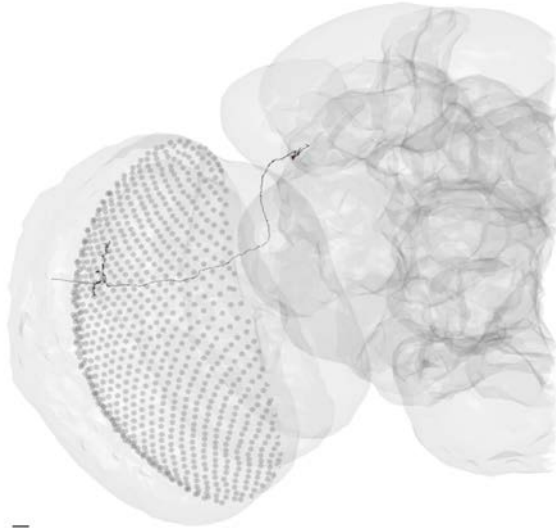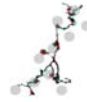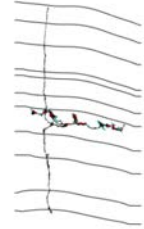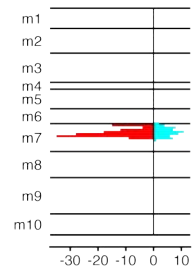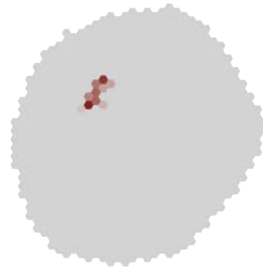

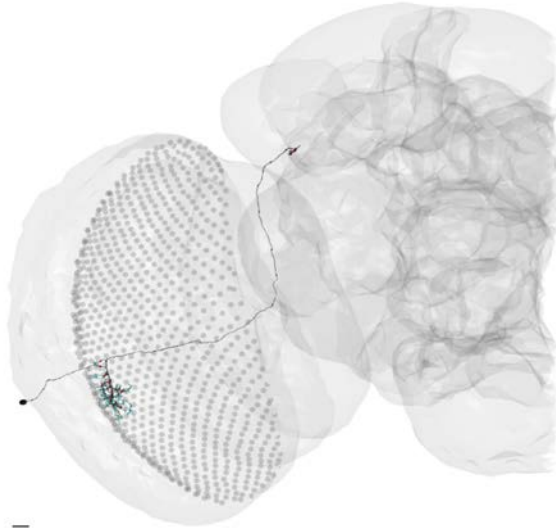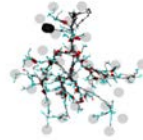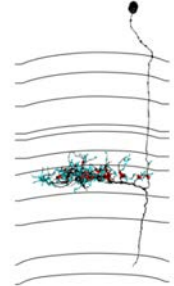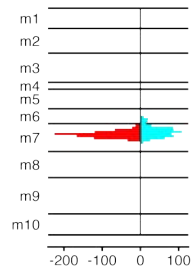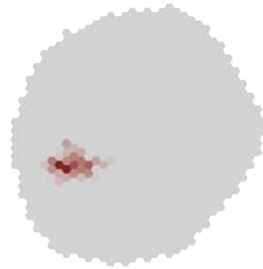

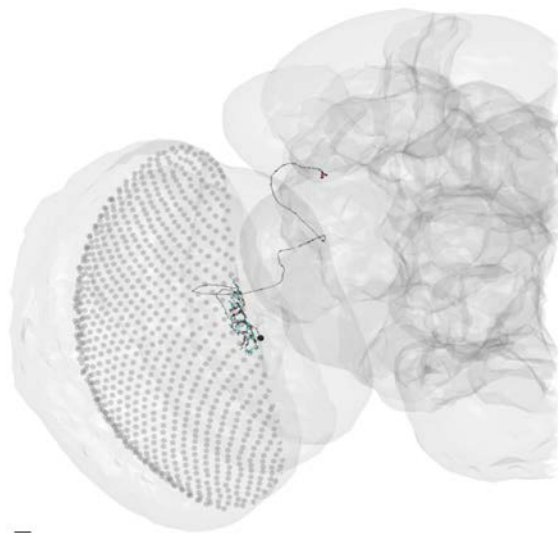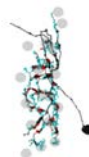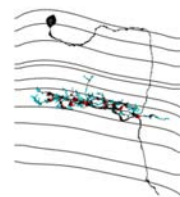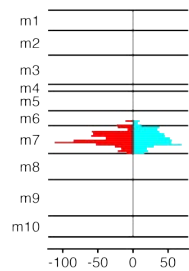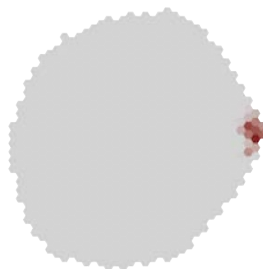

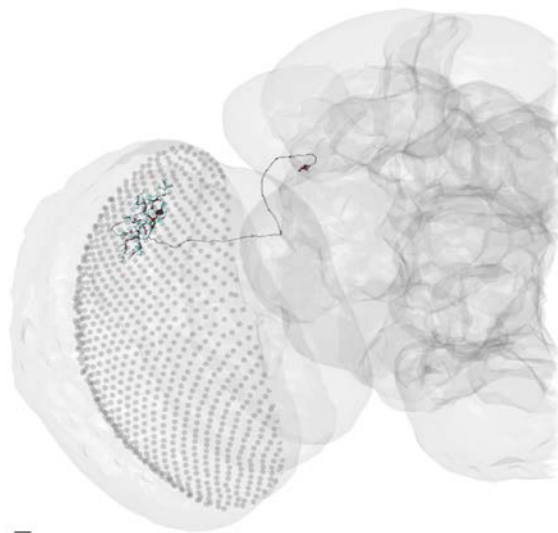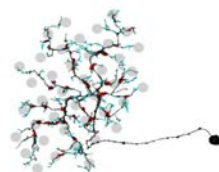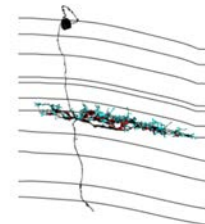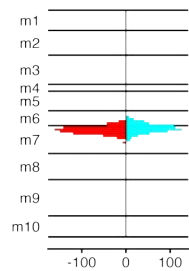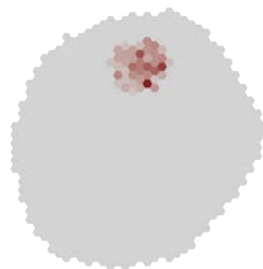

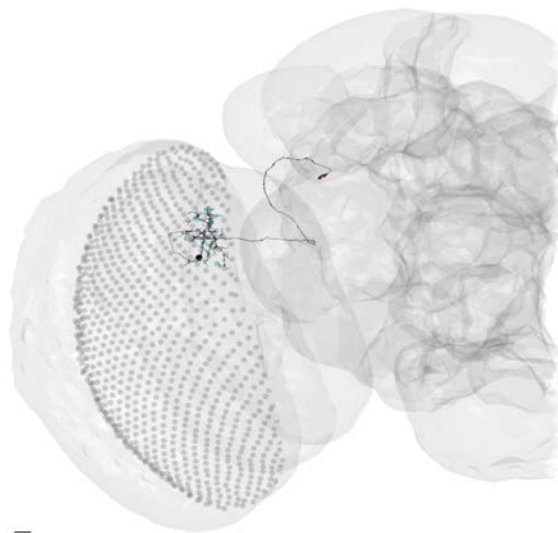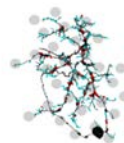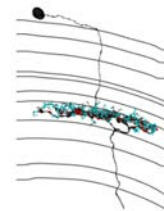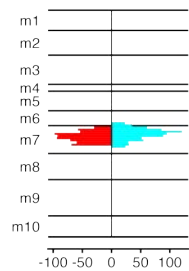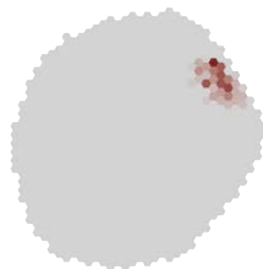

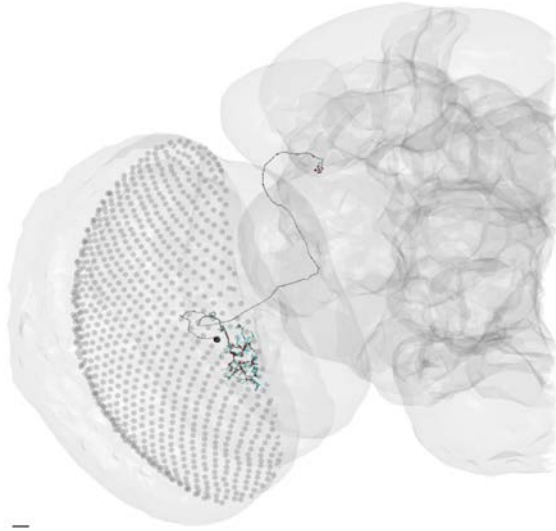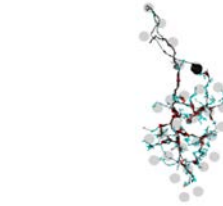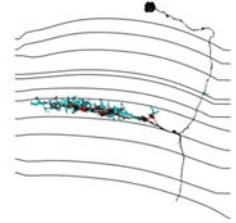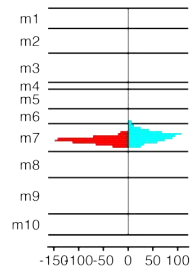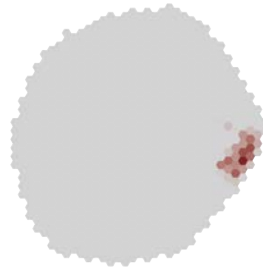

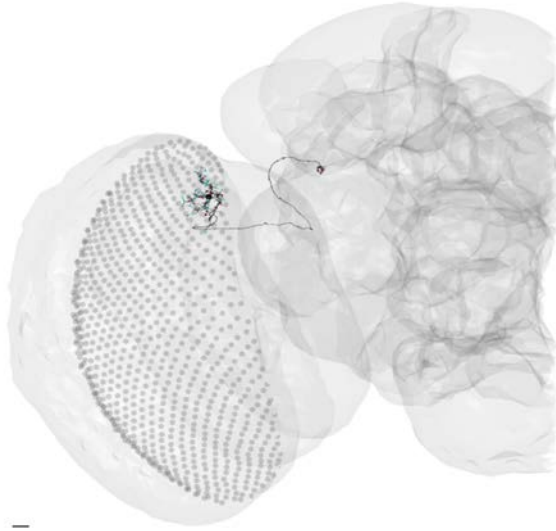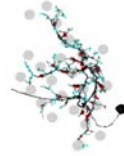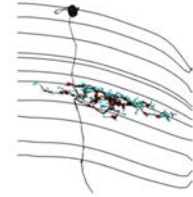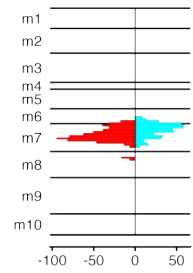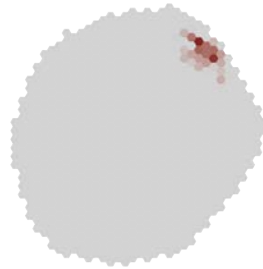

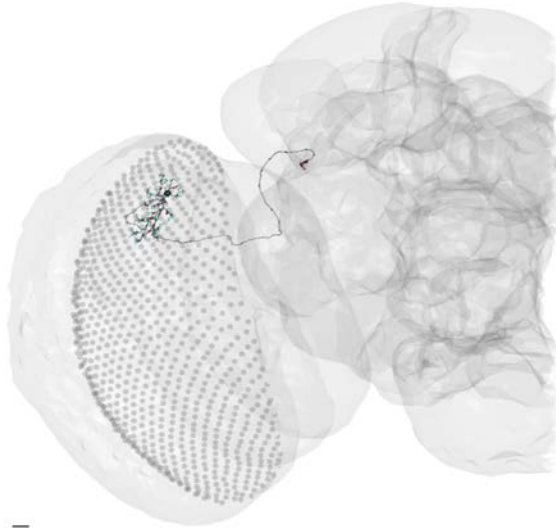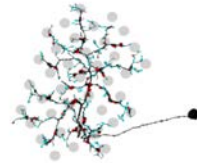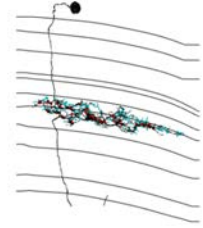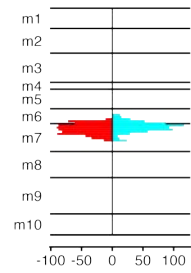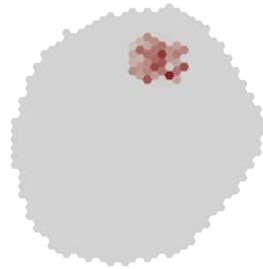

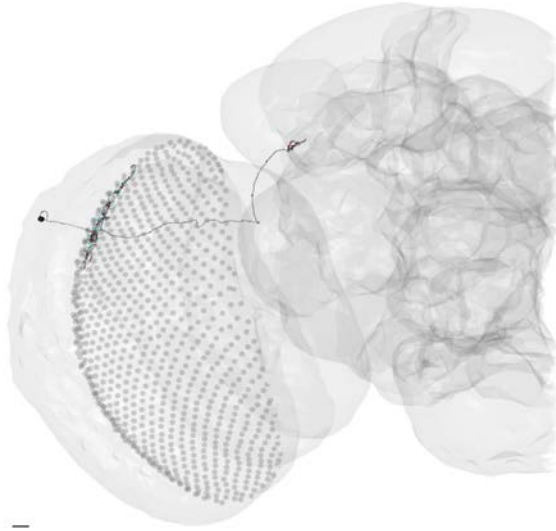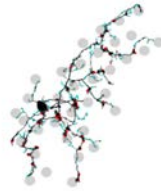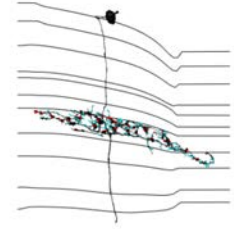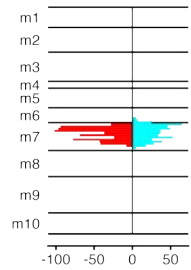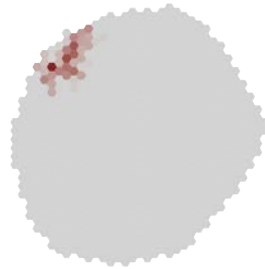

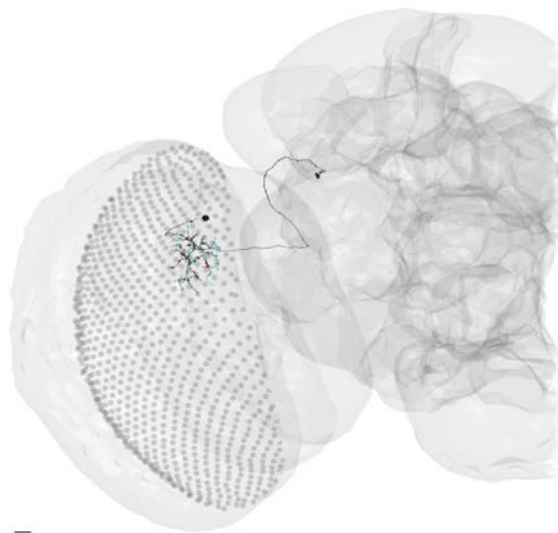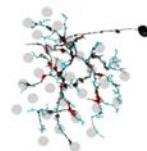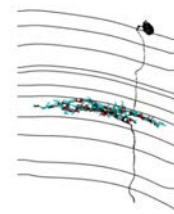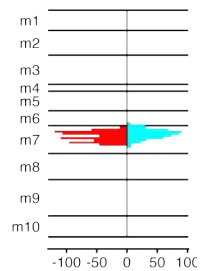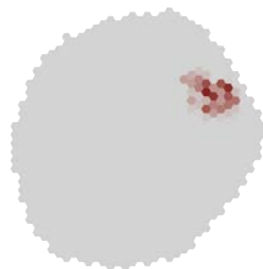

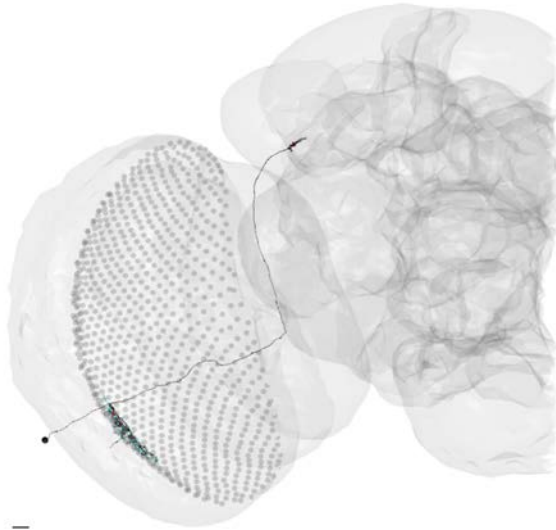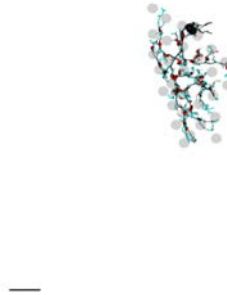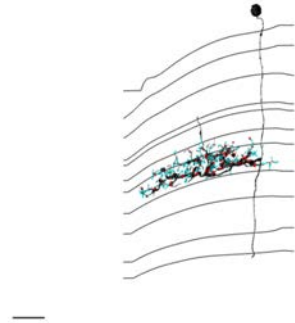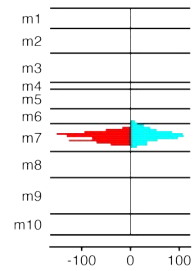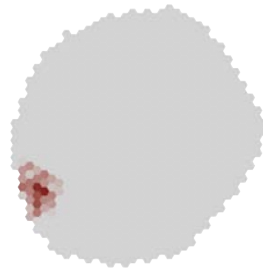

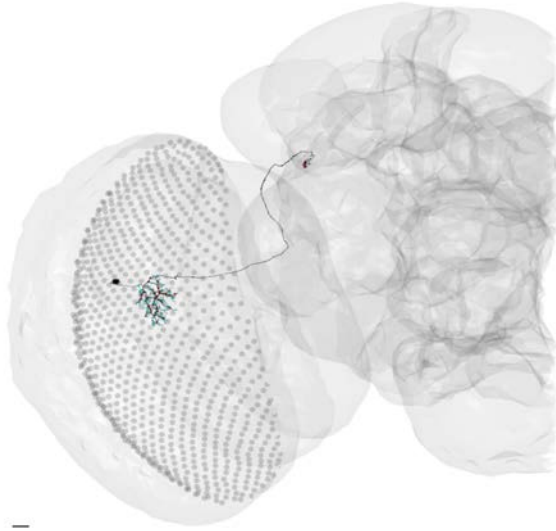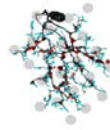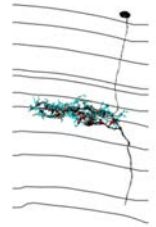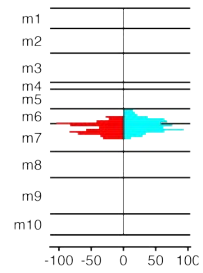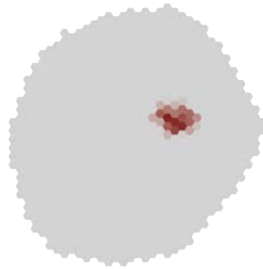

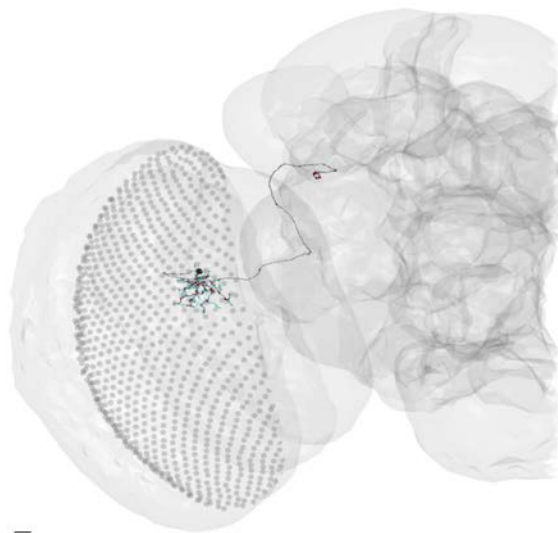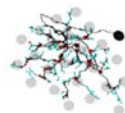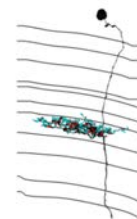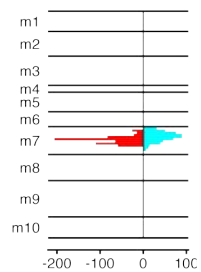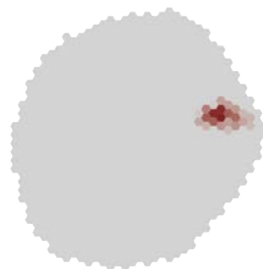

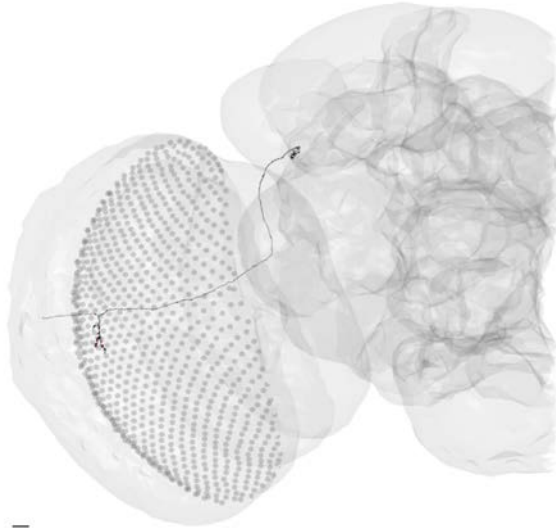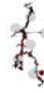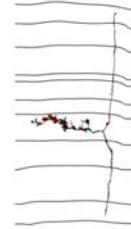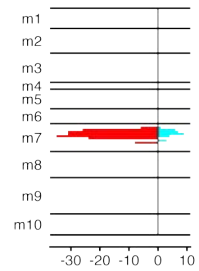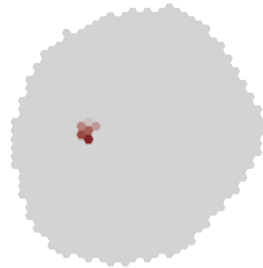

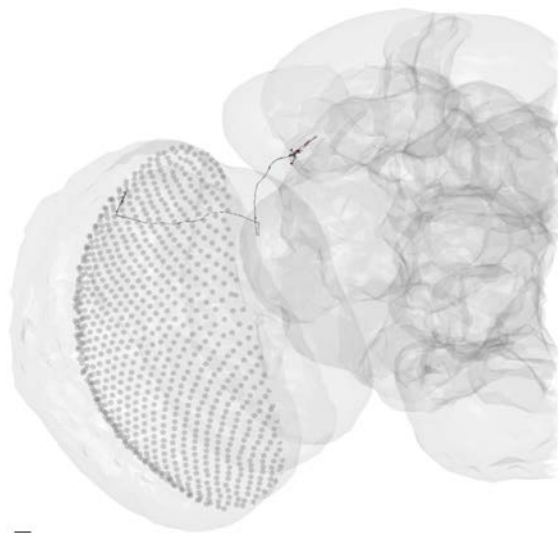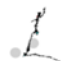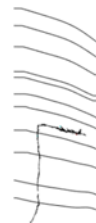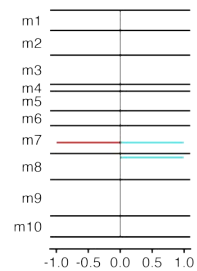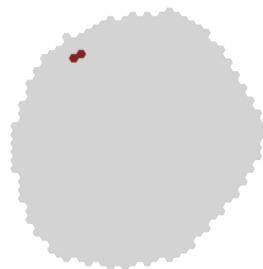

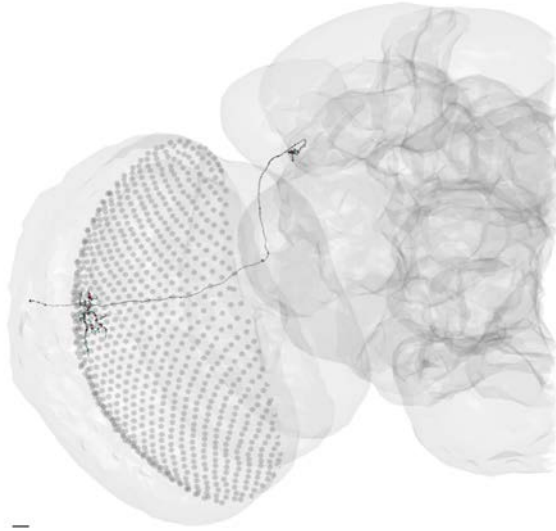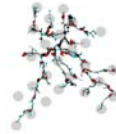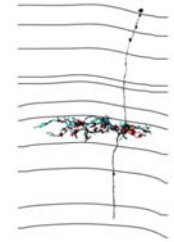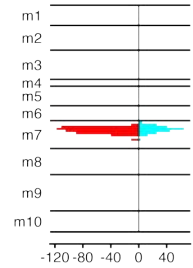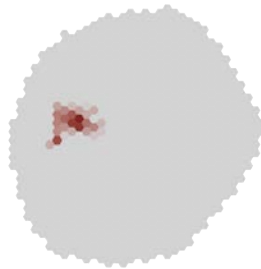

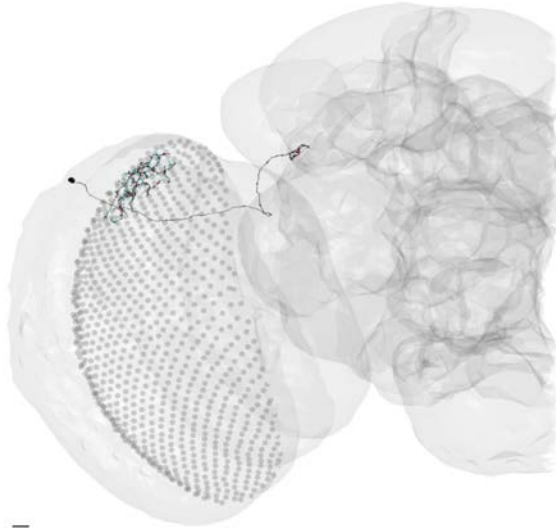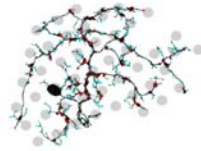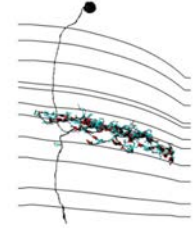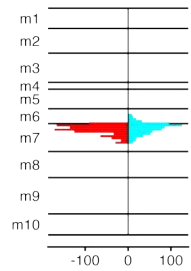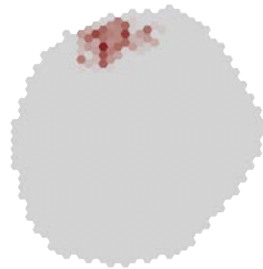

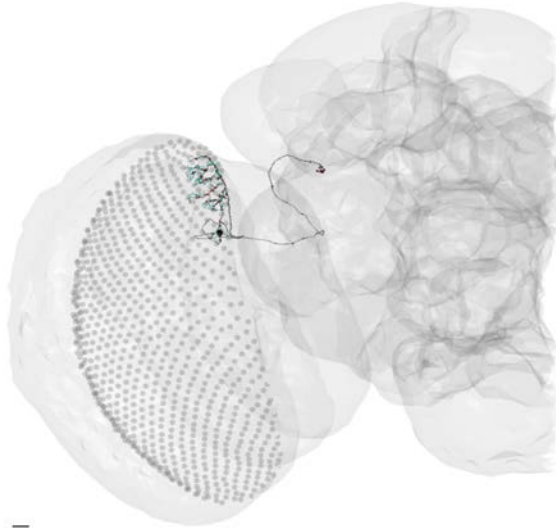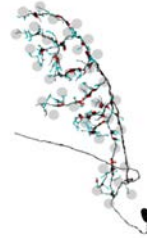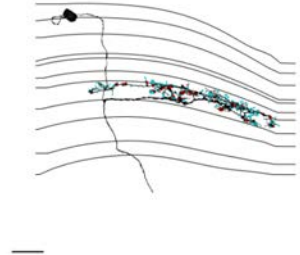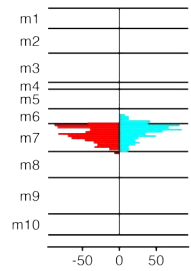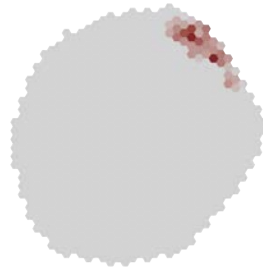

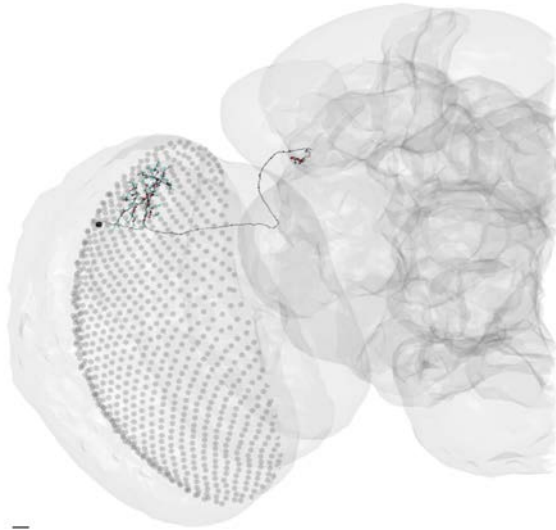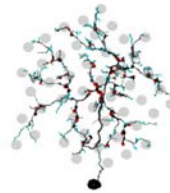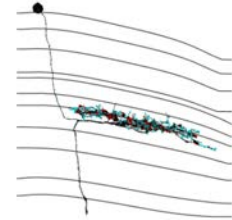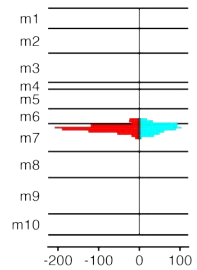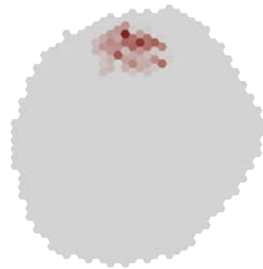

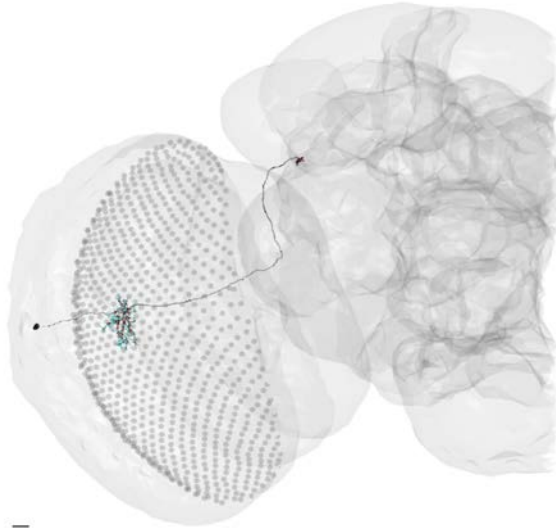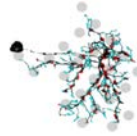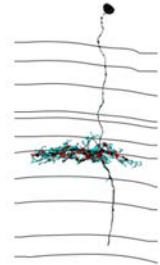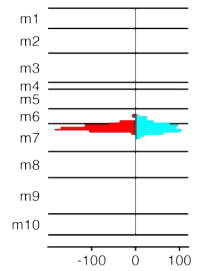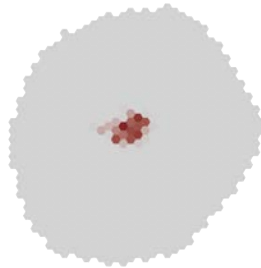

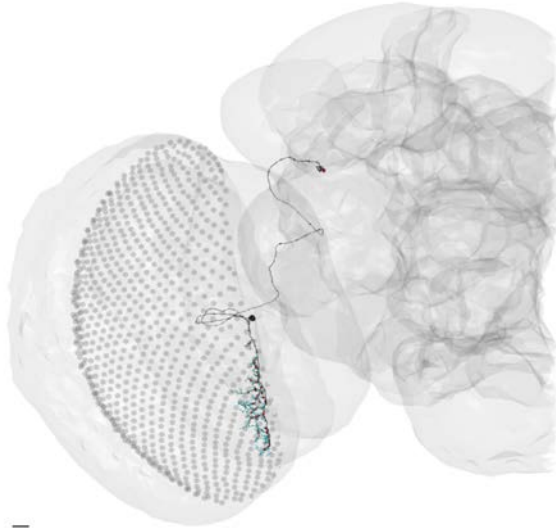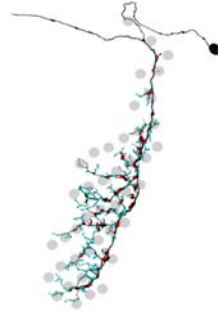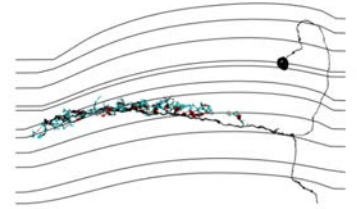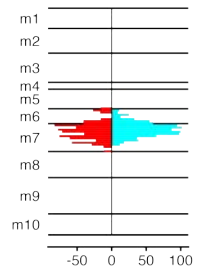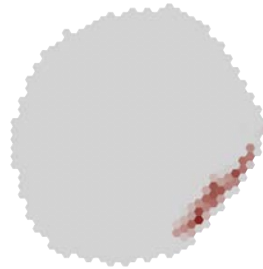

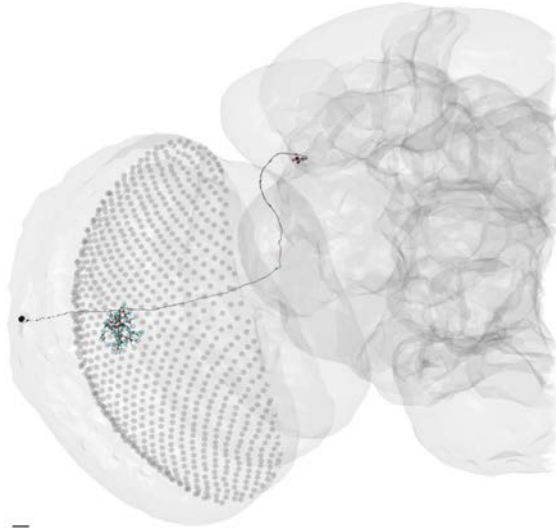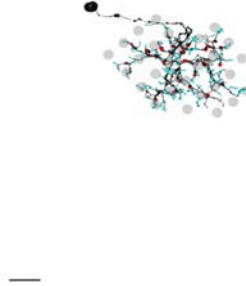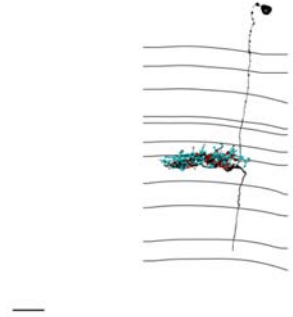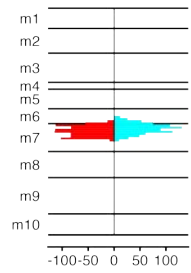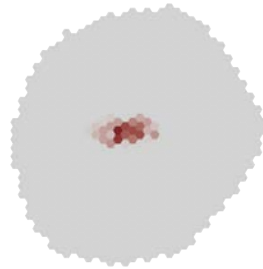

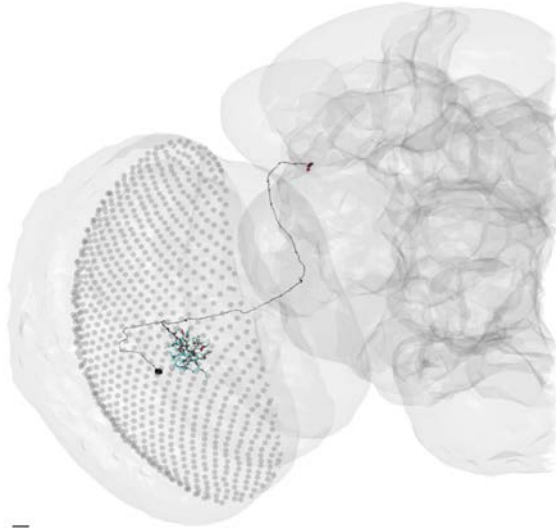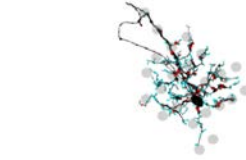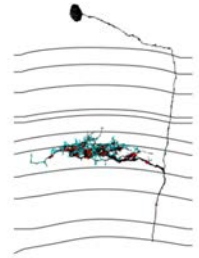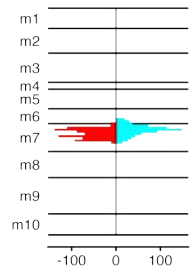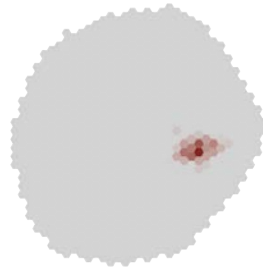

MeTu1\_720575940618408943

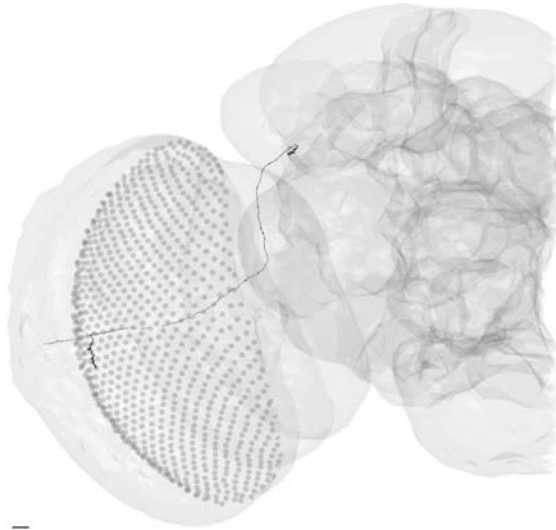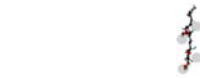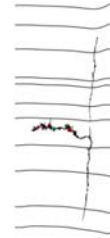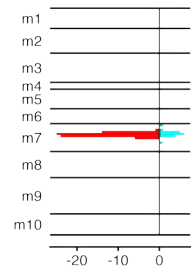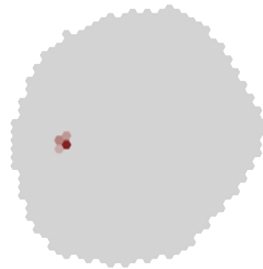

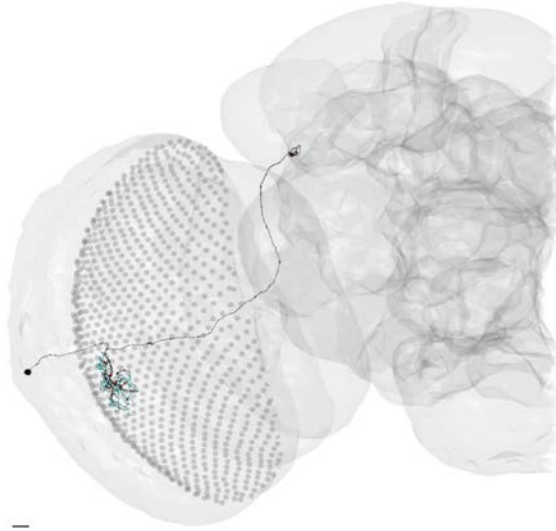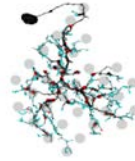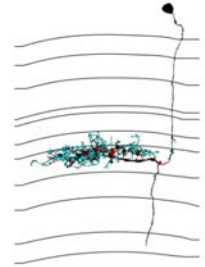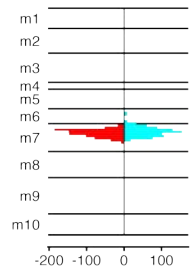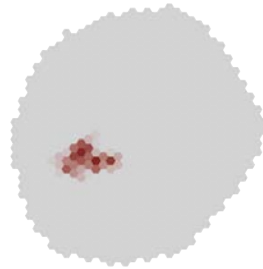

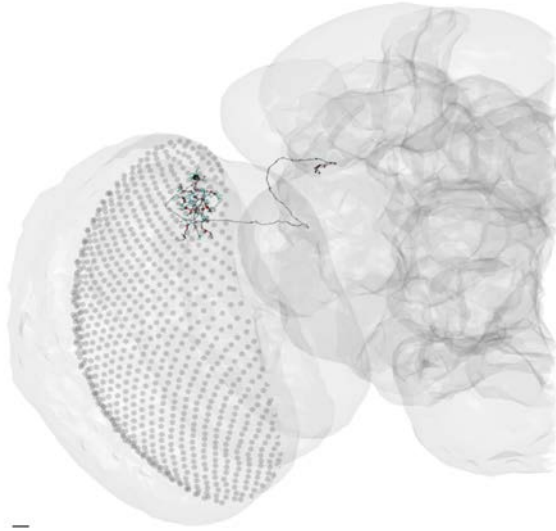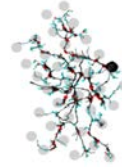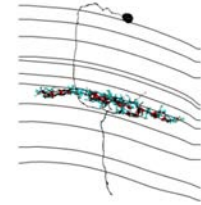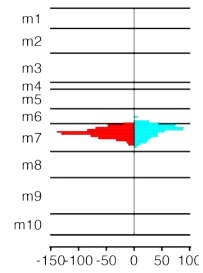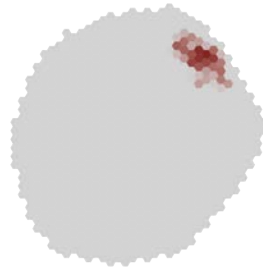

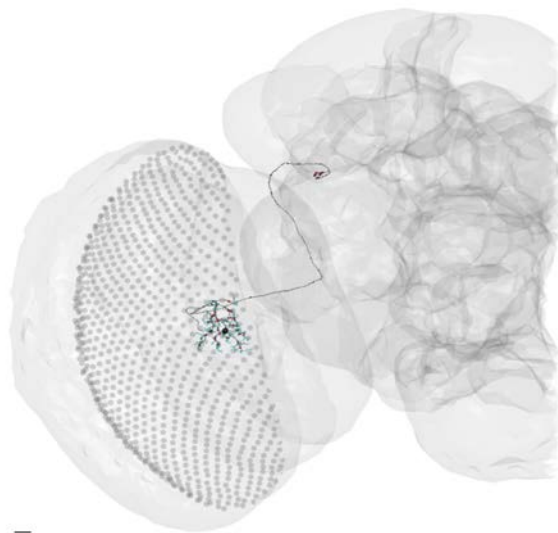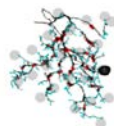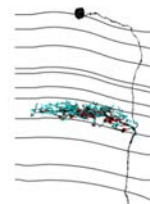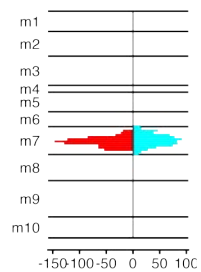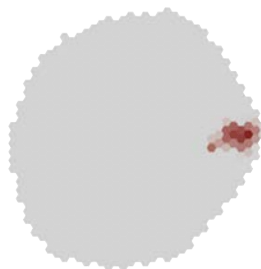

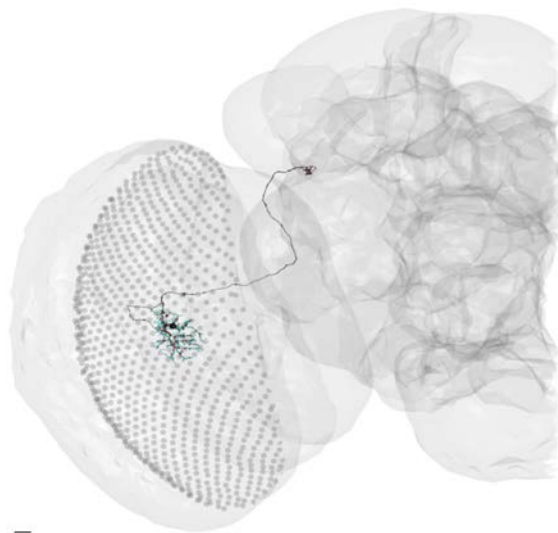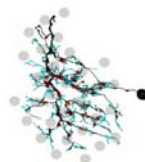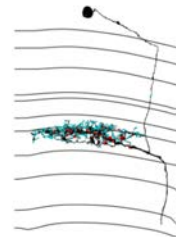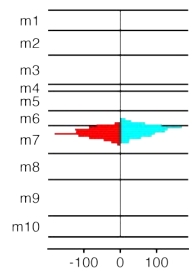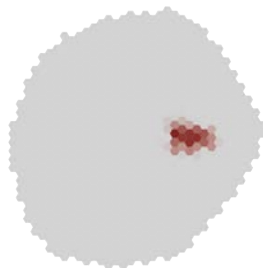

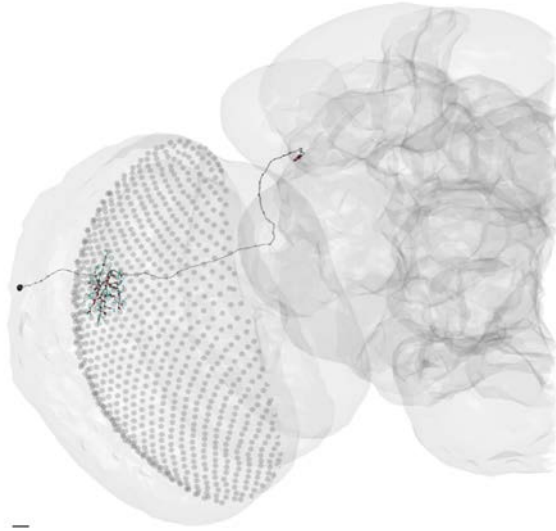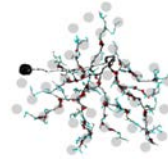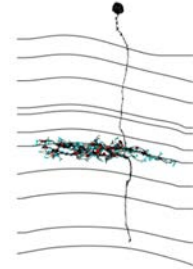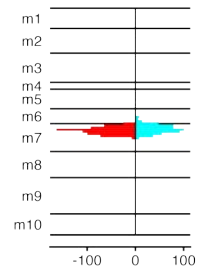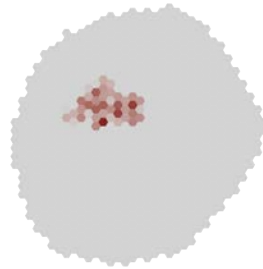

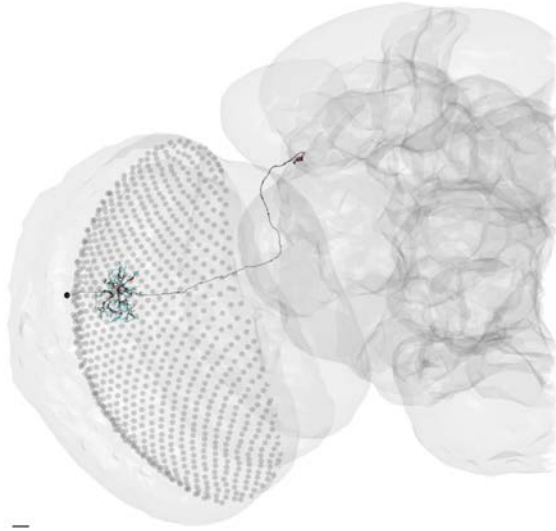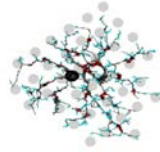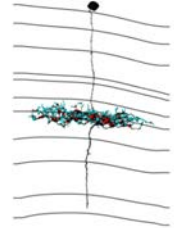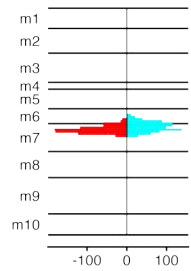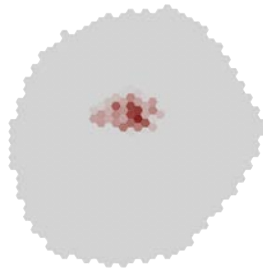

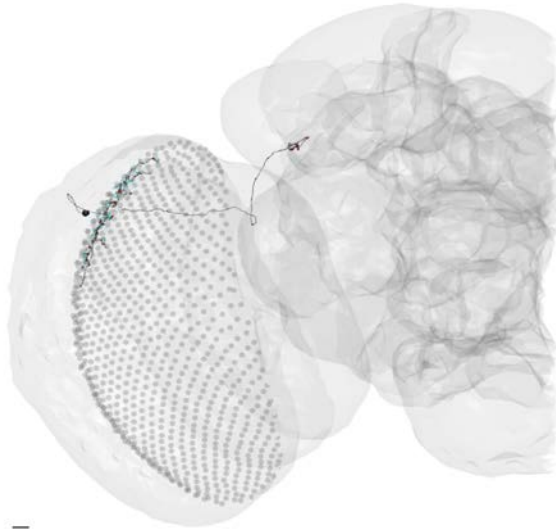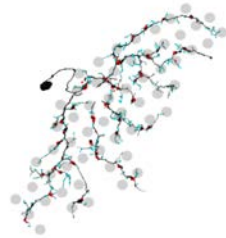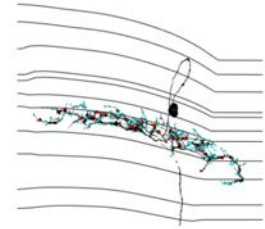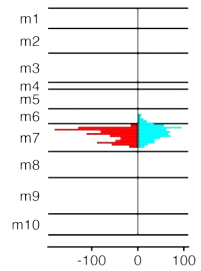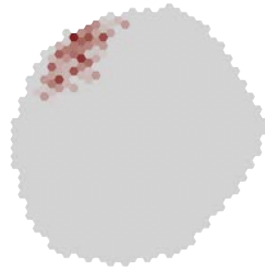

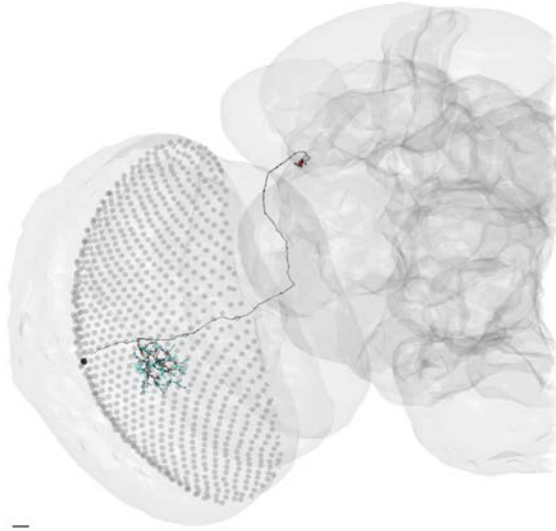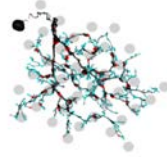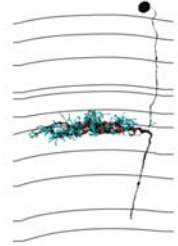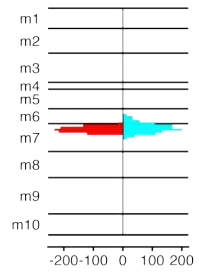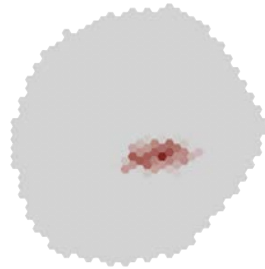

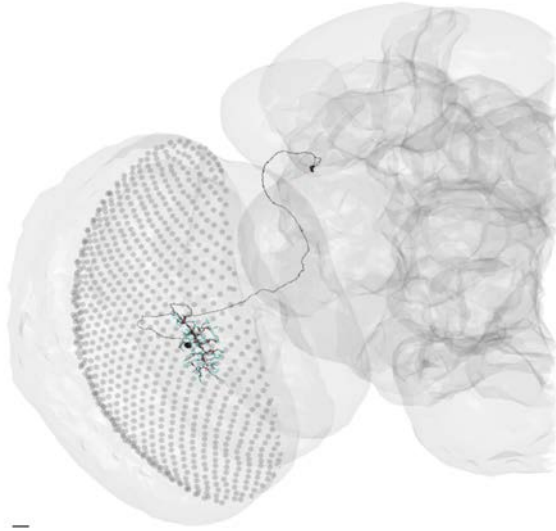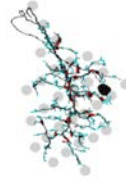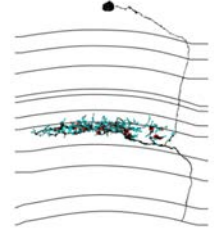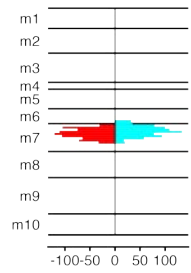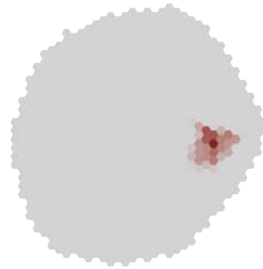

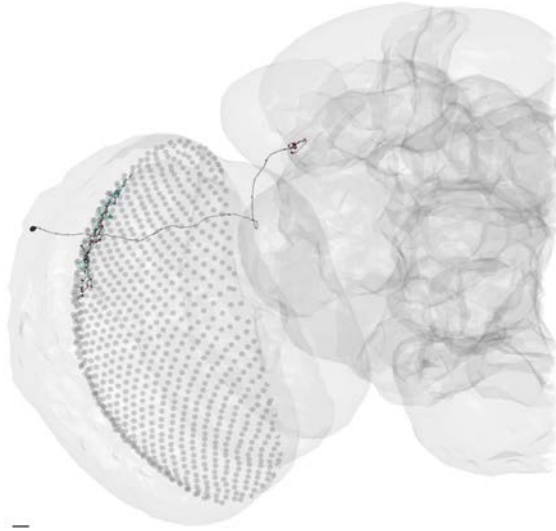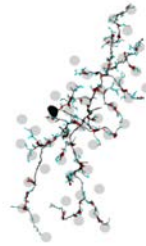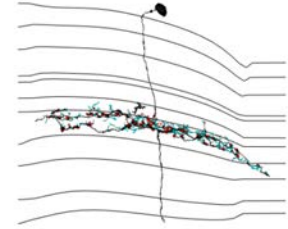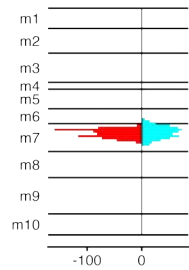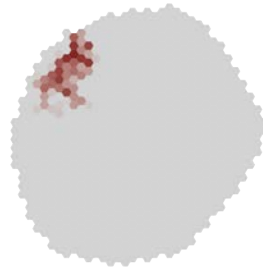

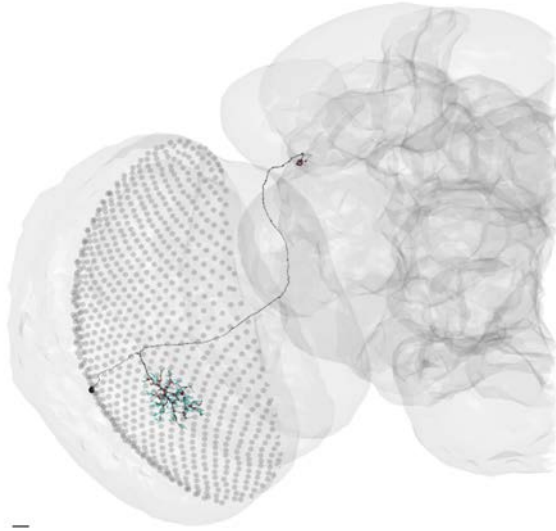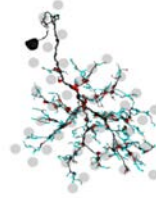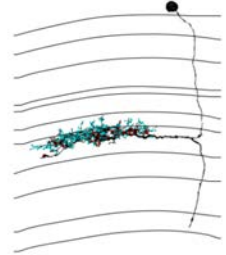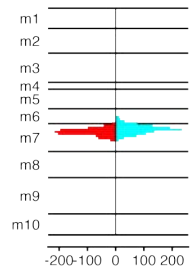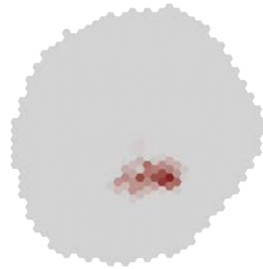

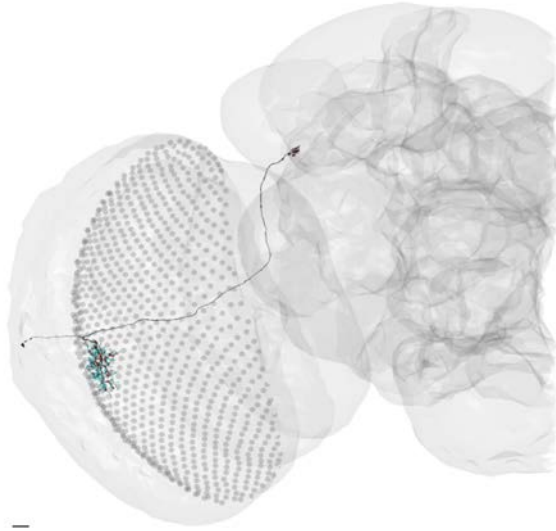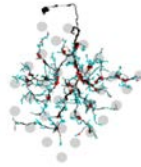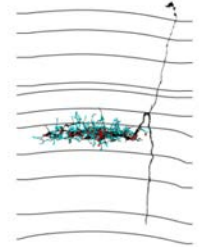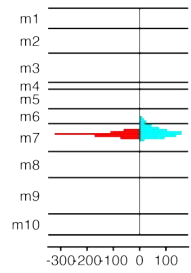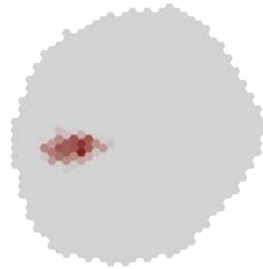

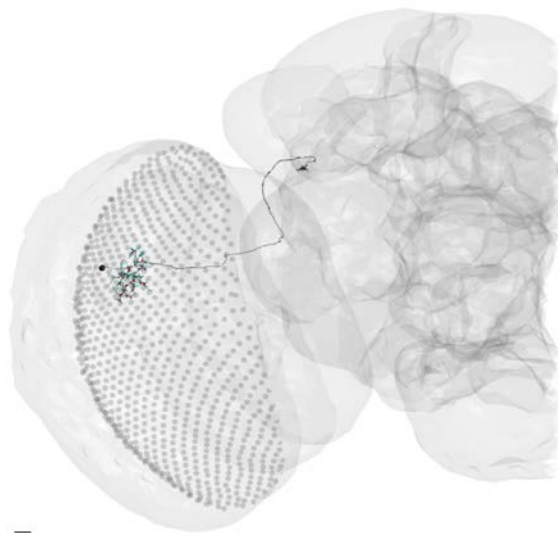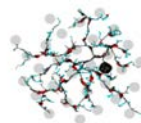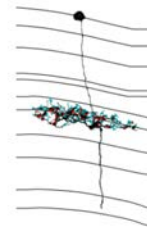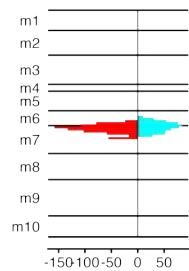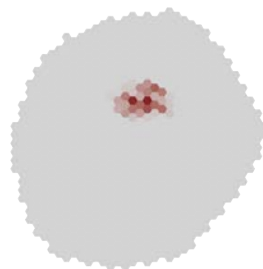

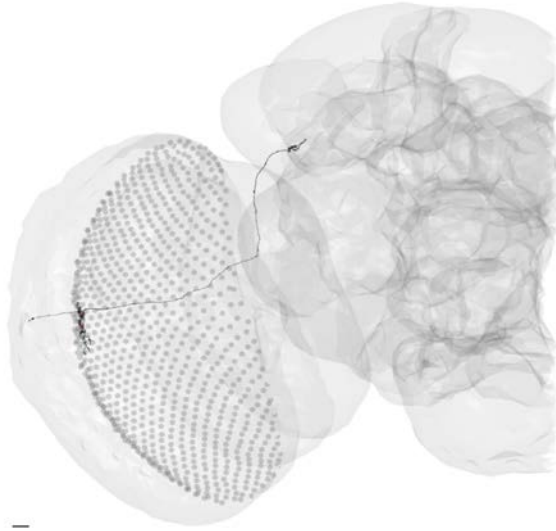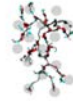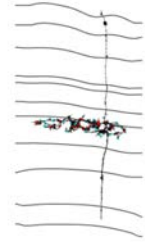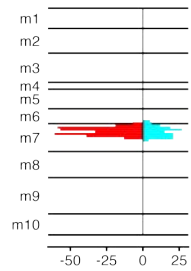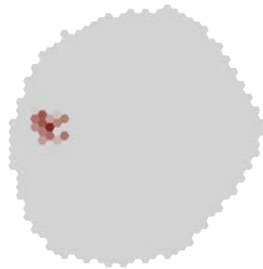

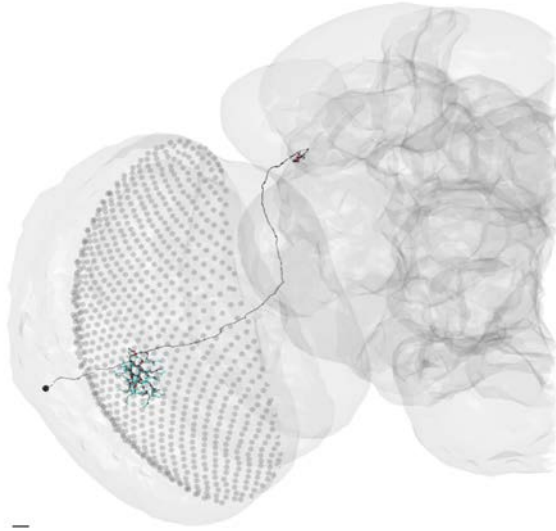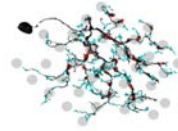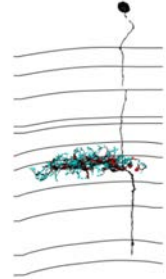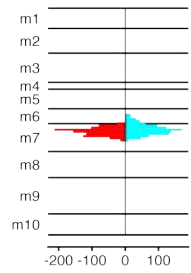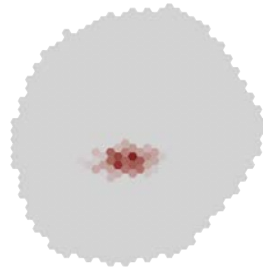

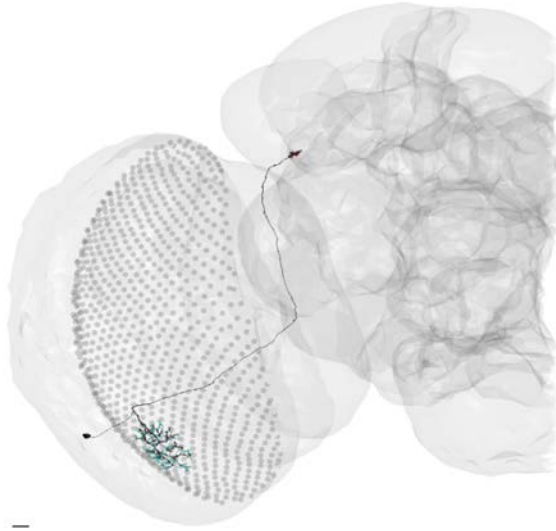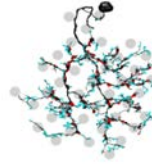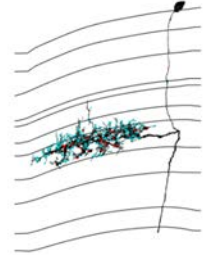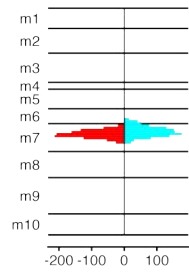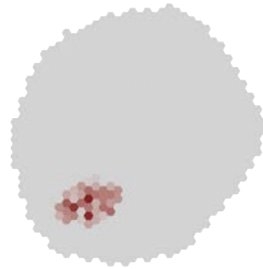

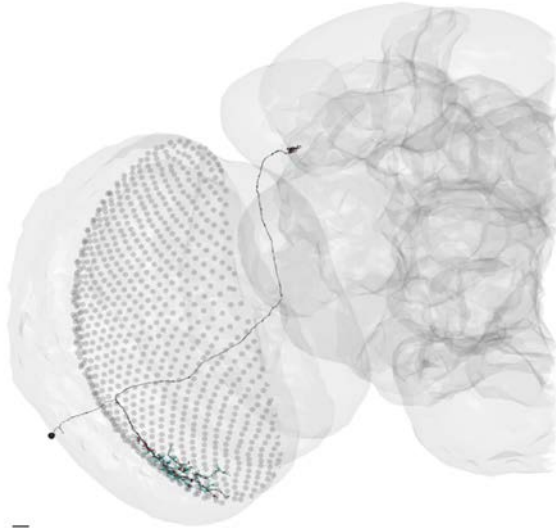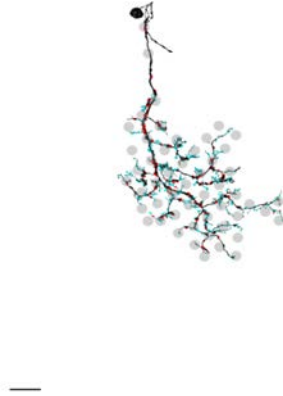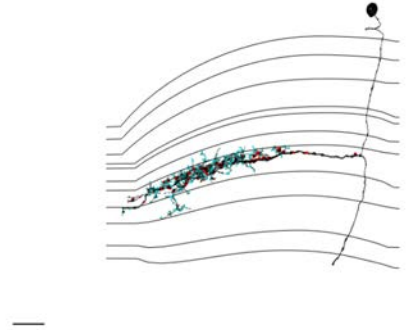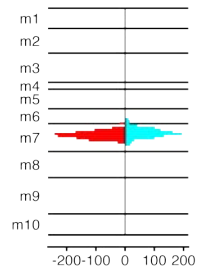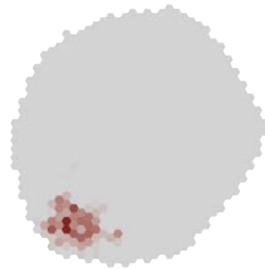

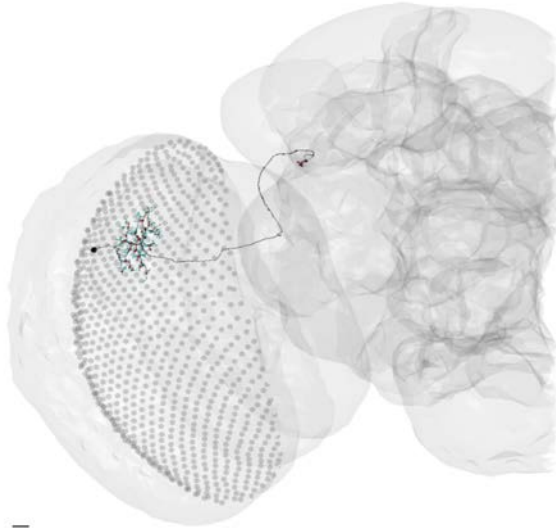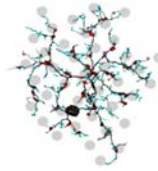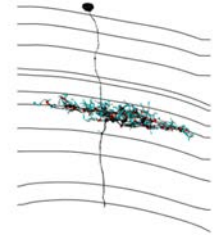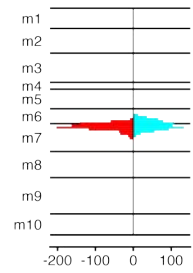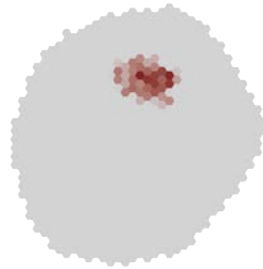

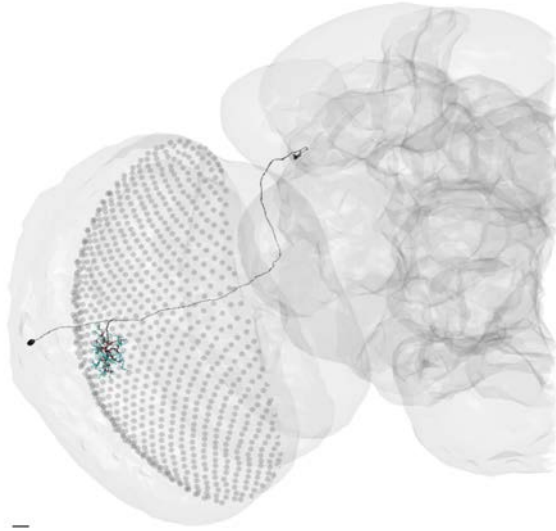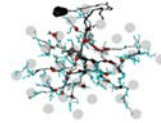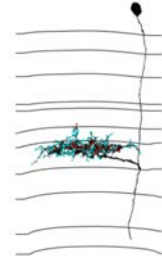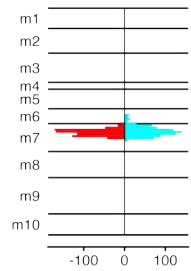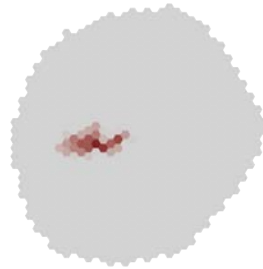

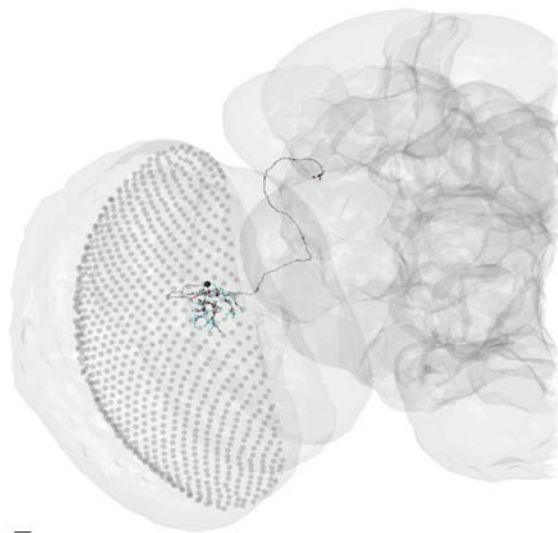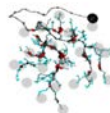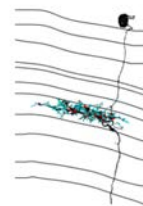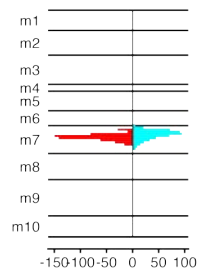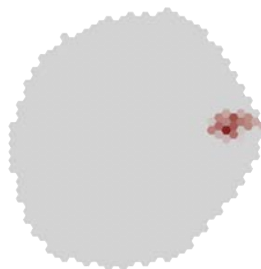

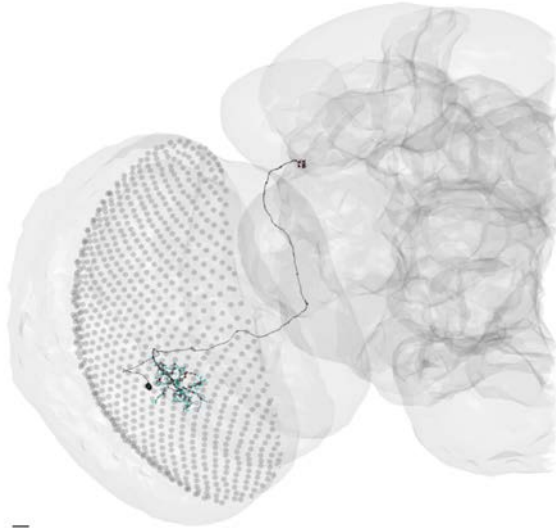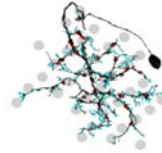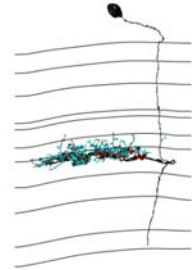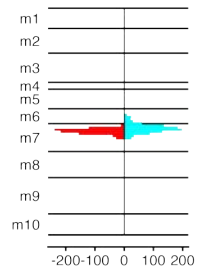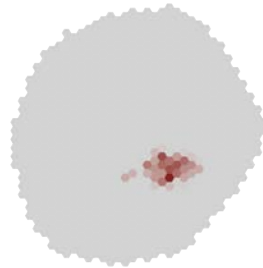

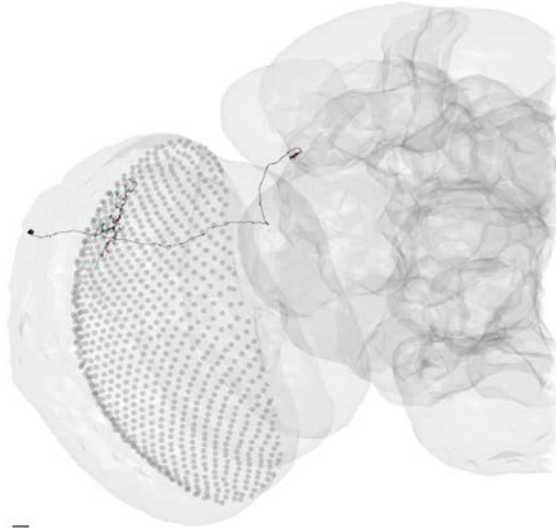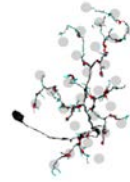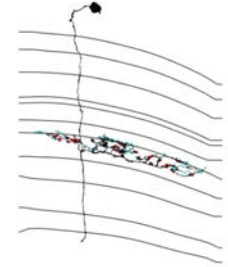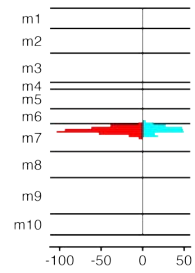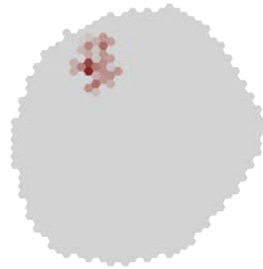

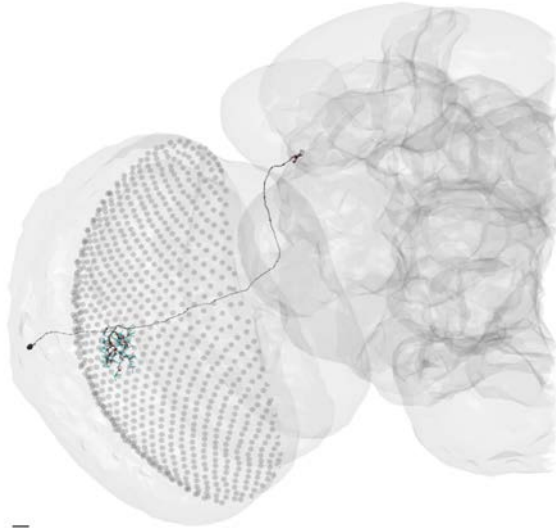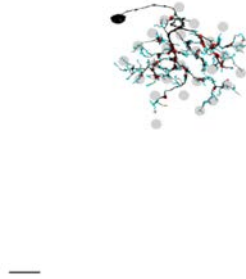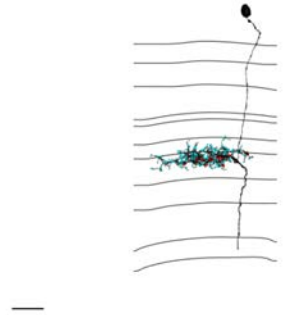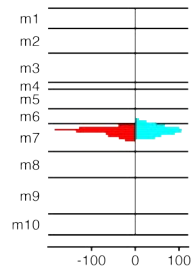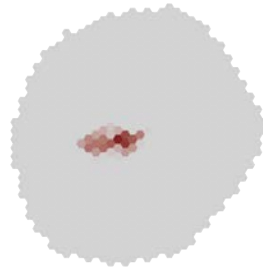

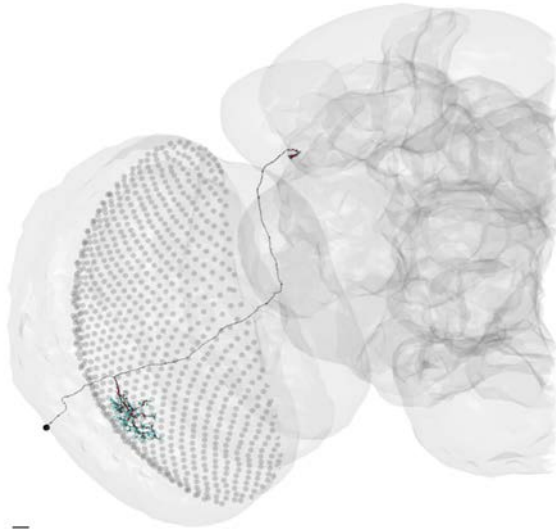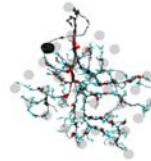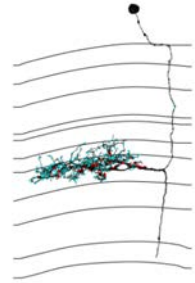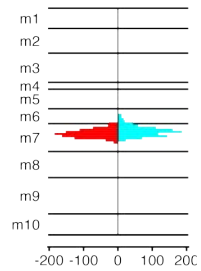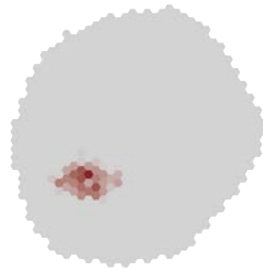

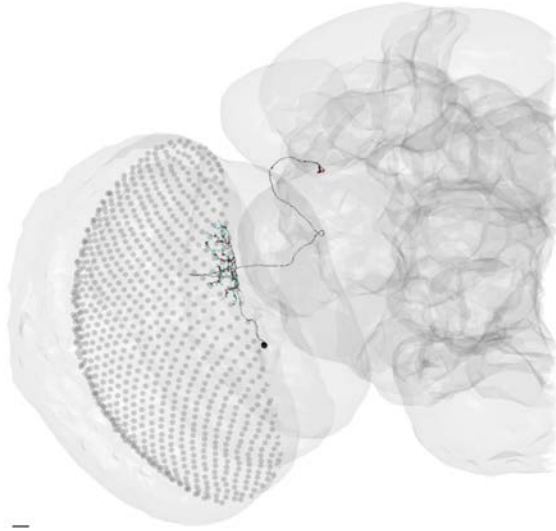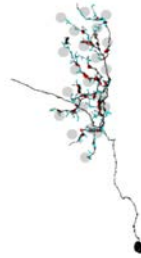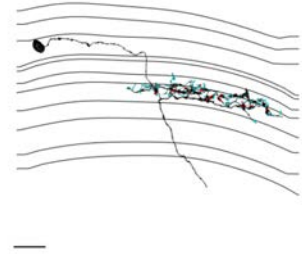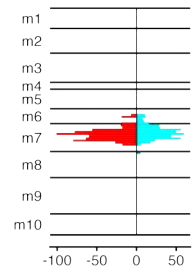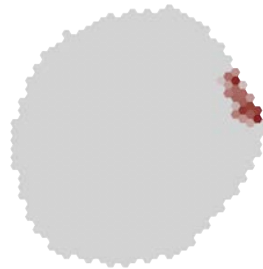

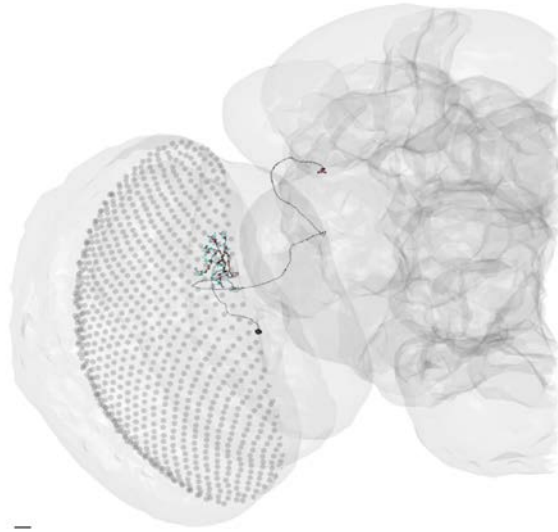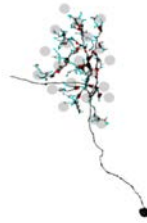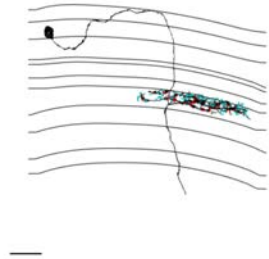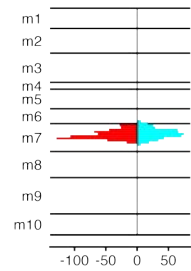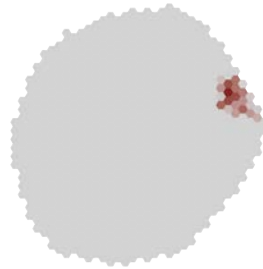

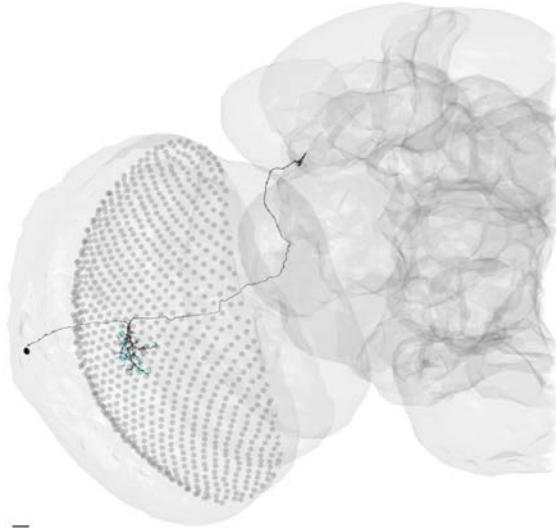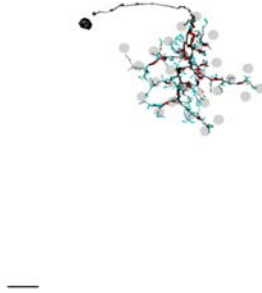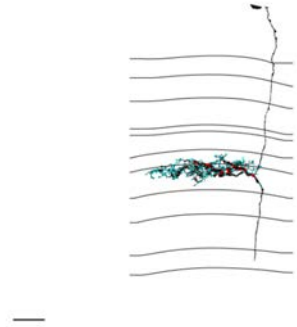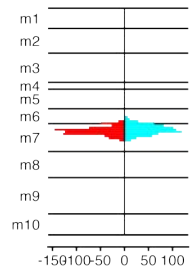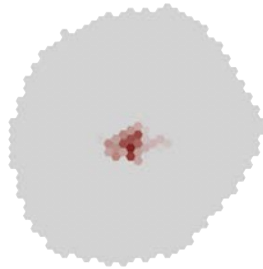

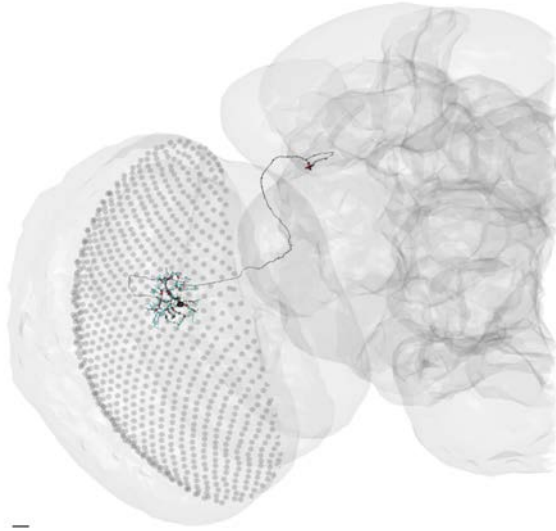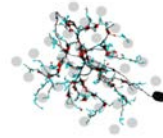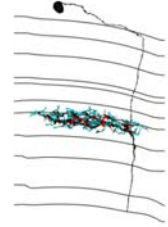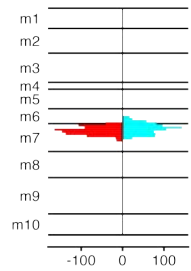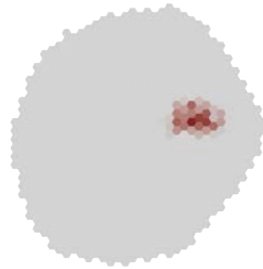

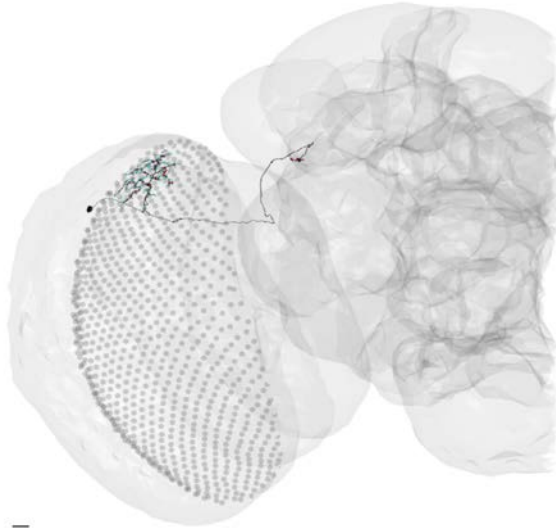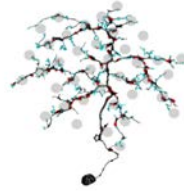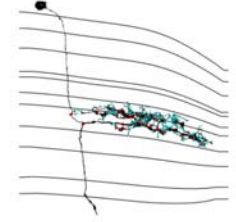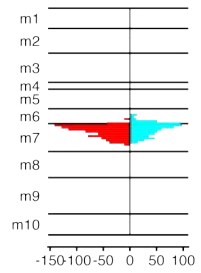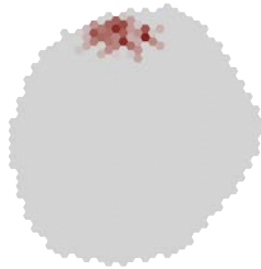

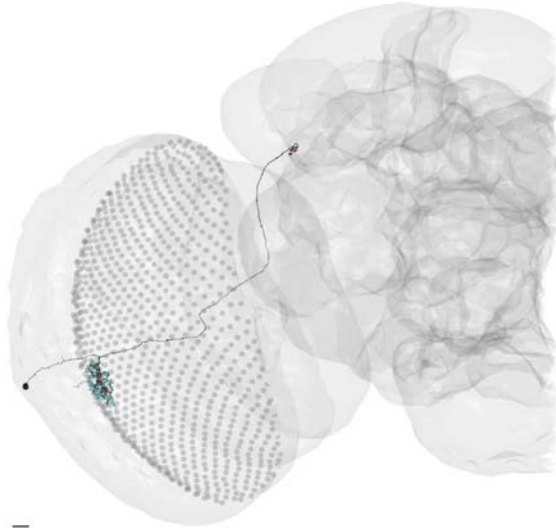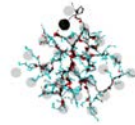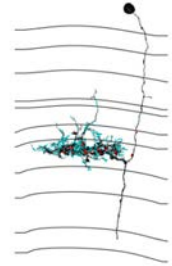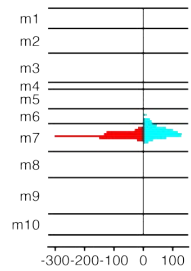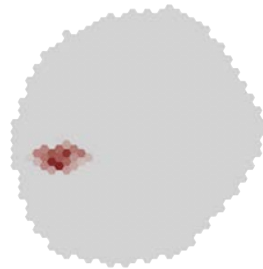

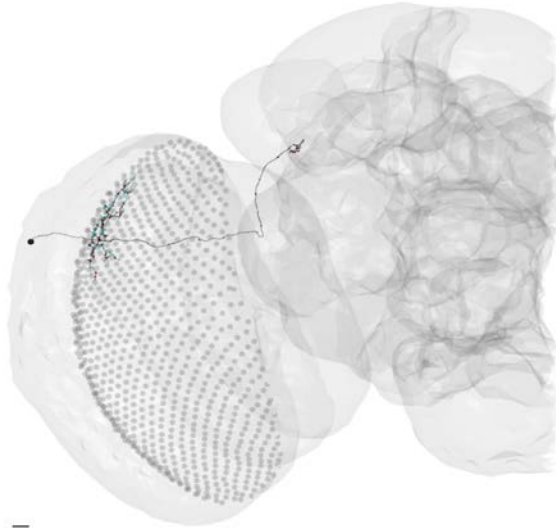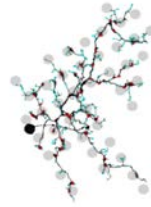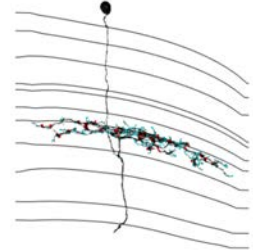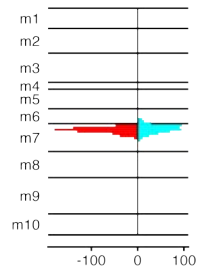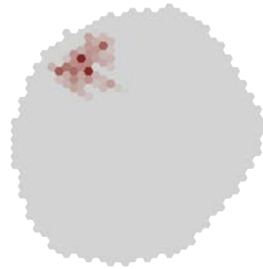

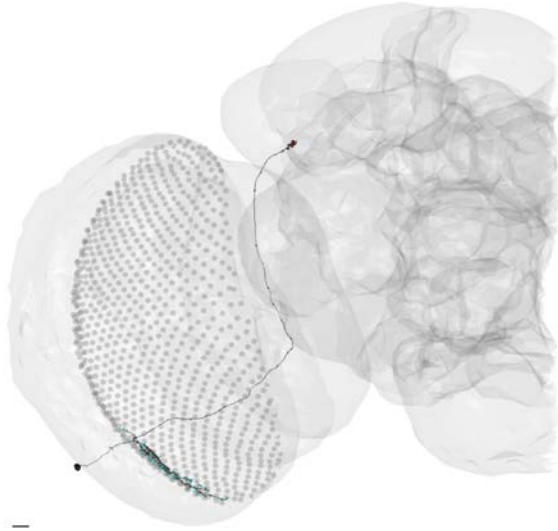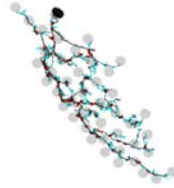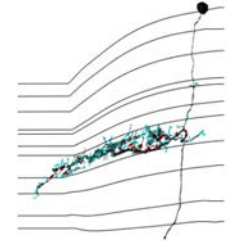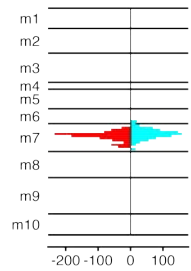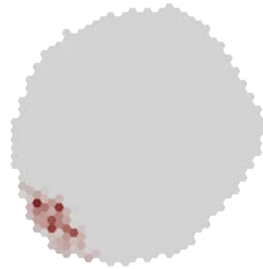

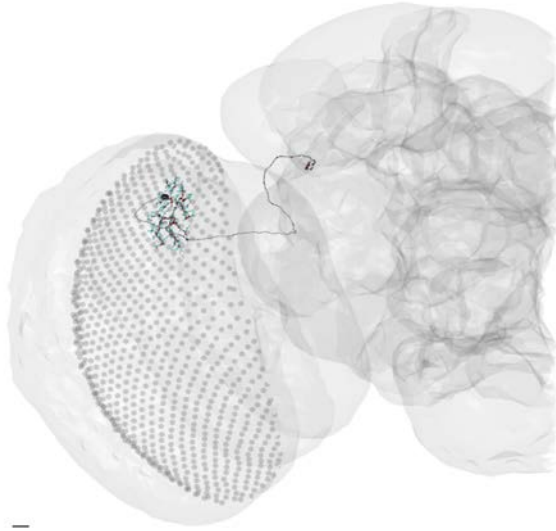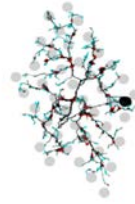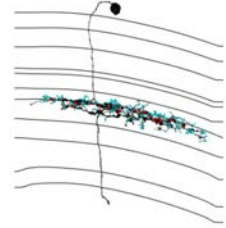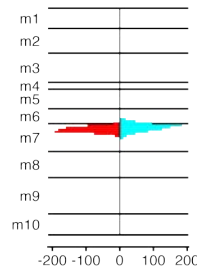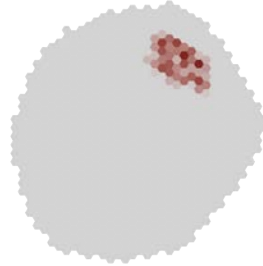

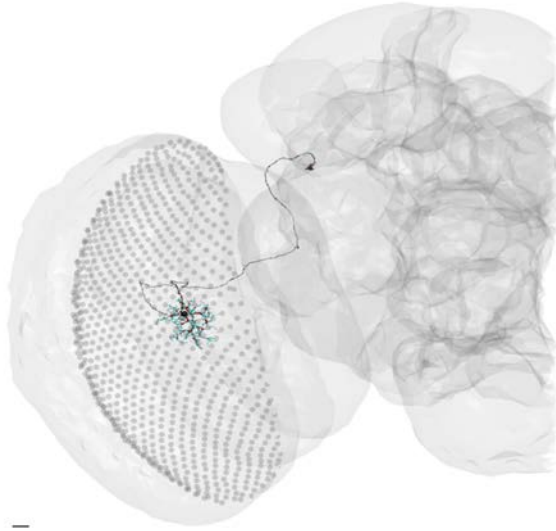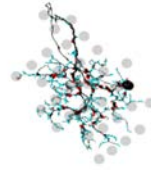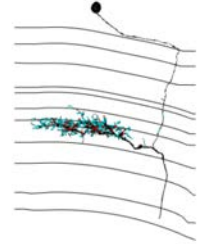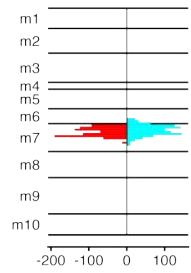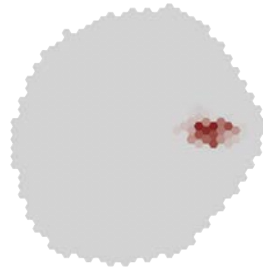

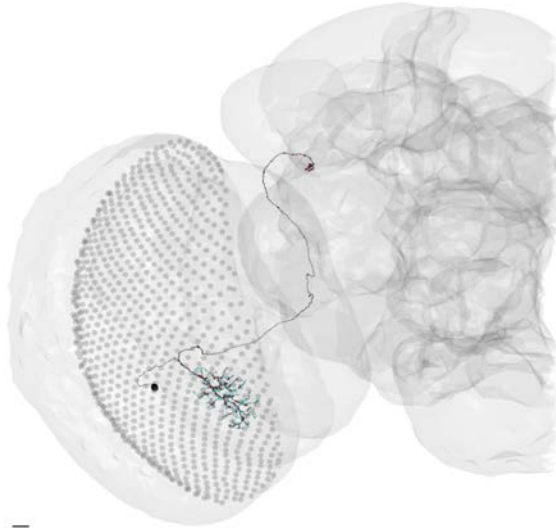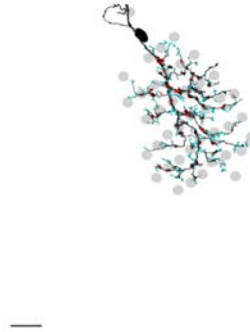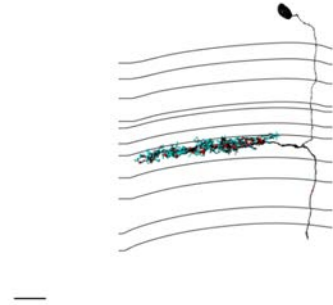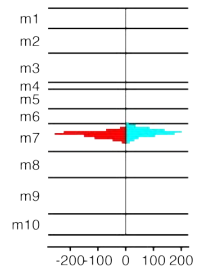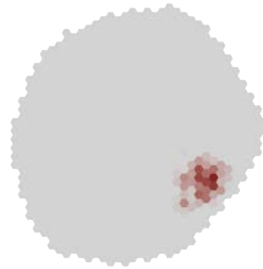

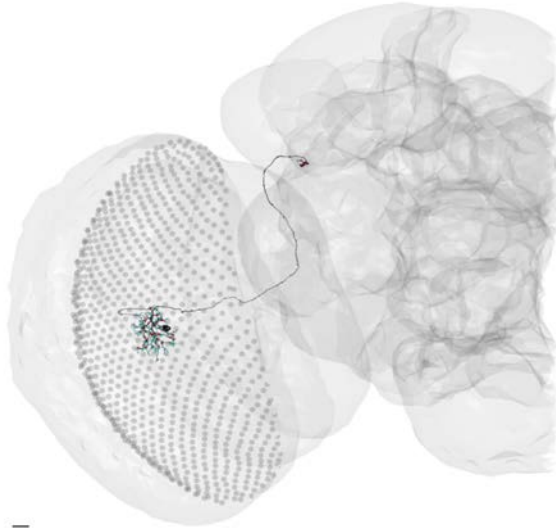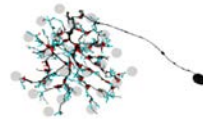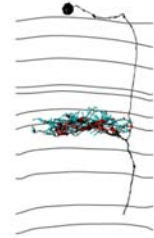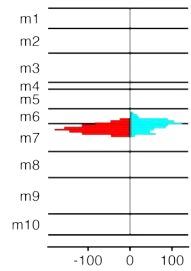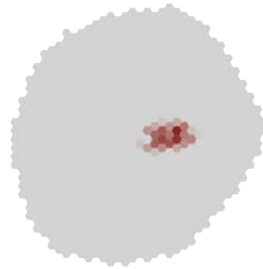

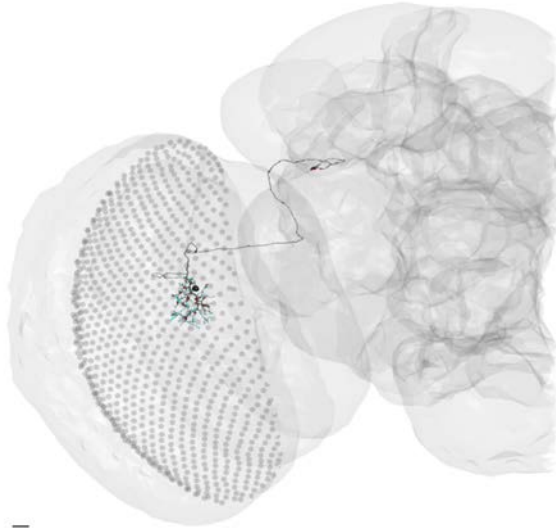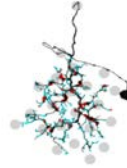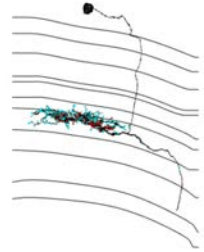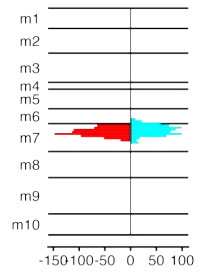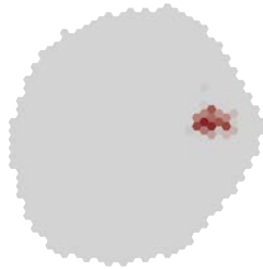

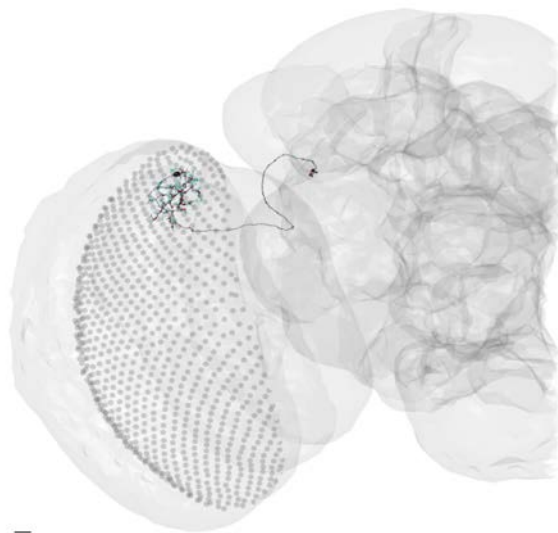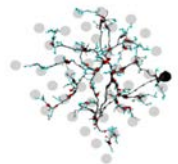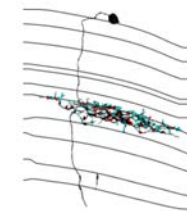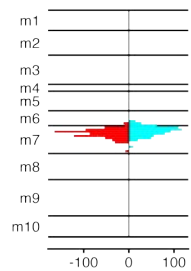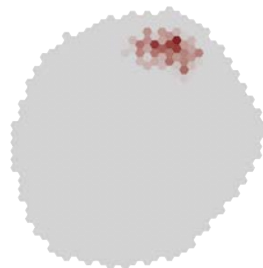

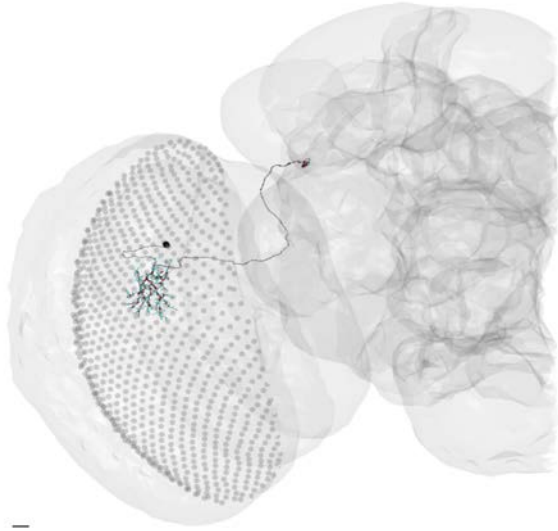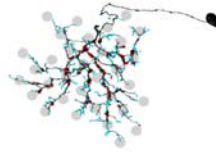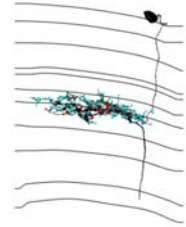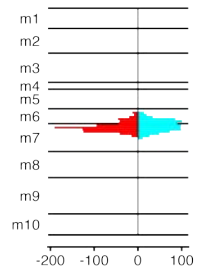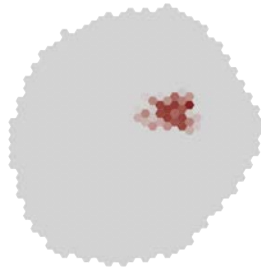

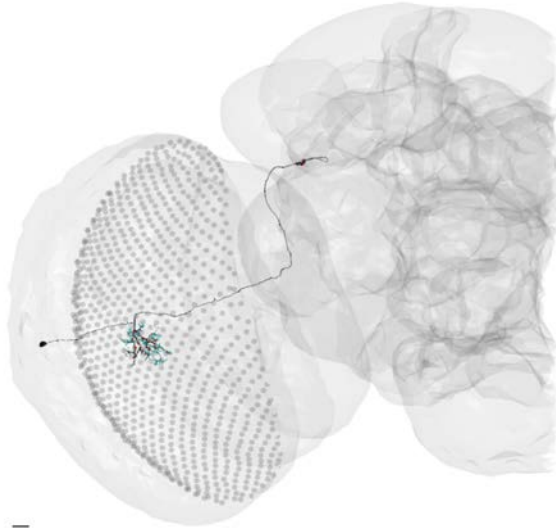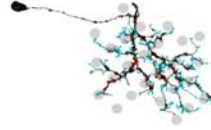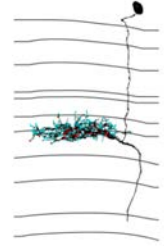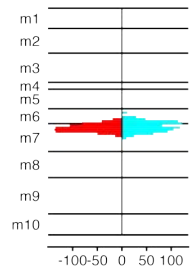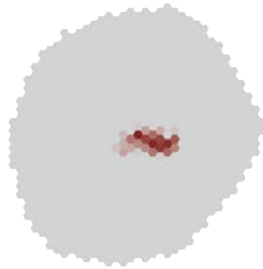

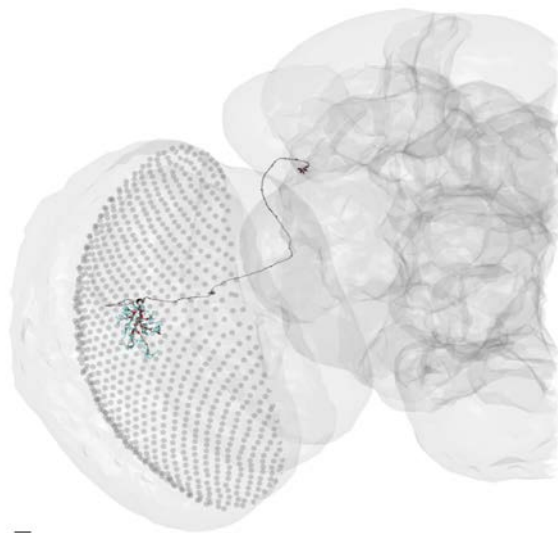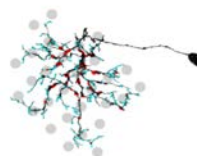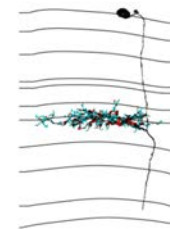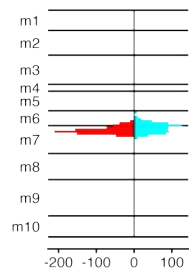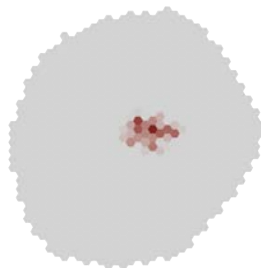

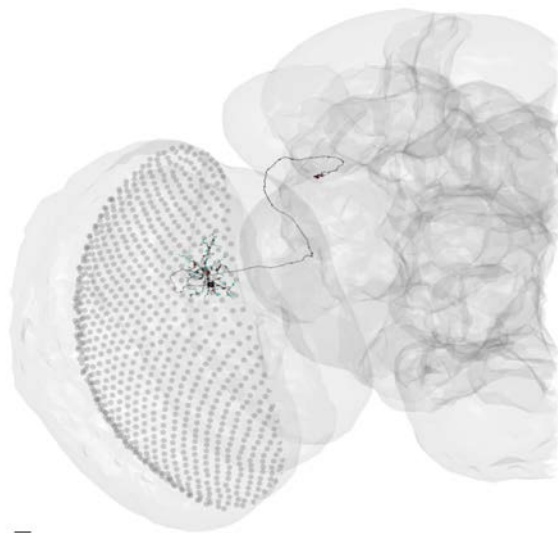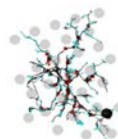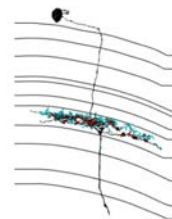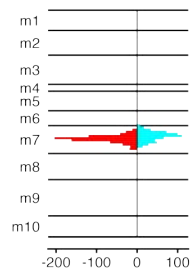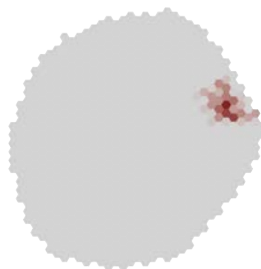

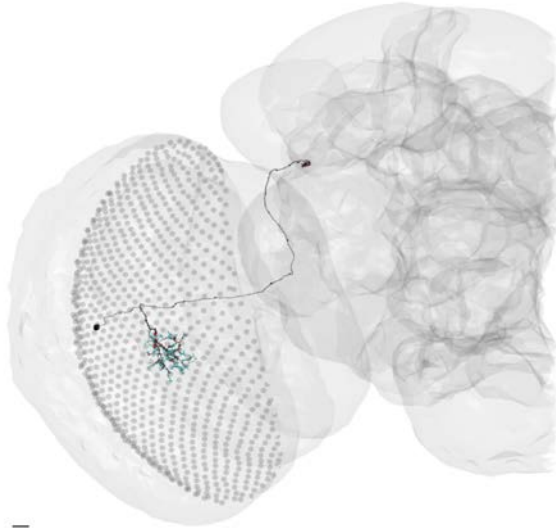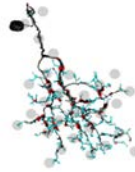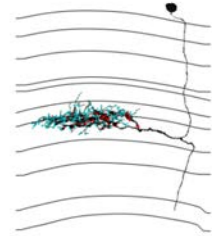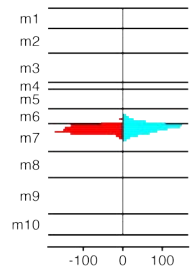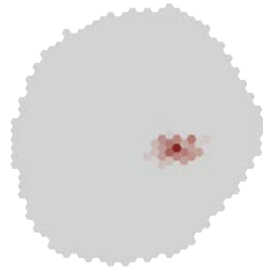

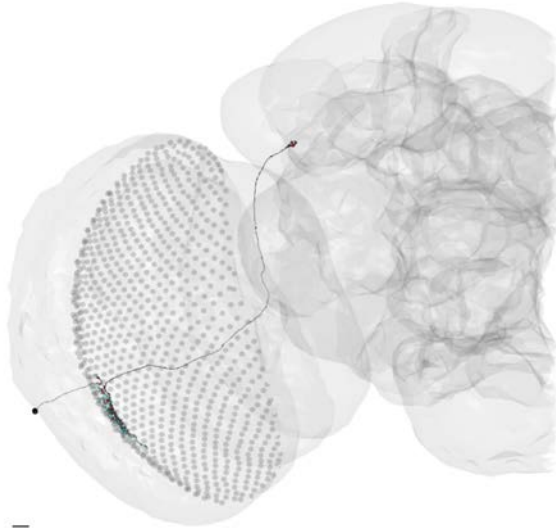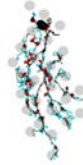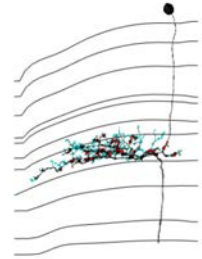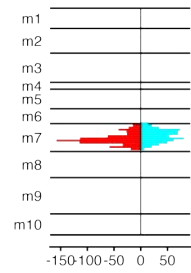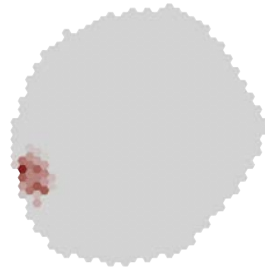

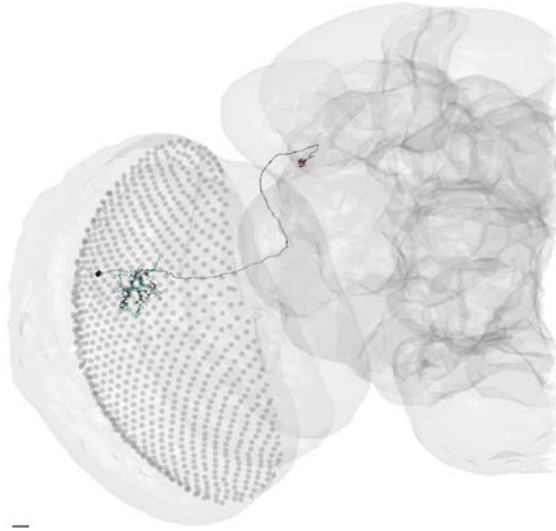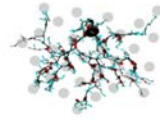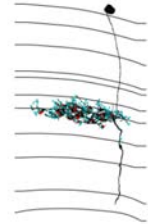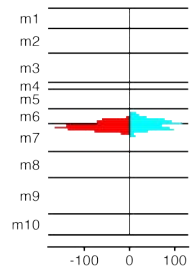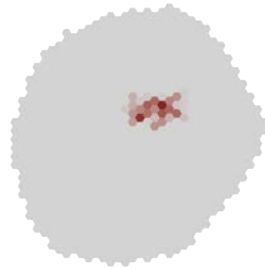

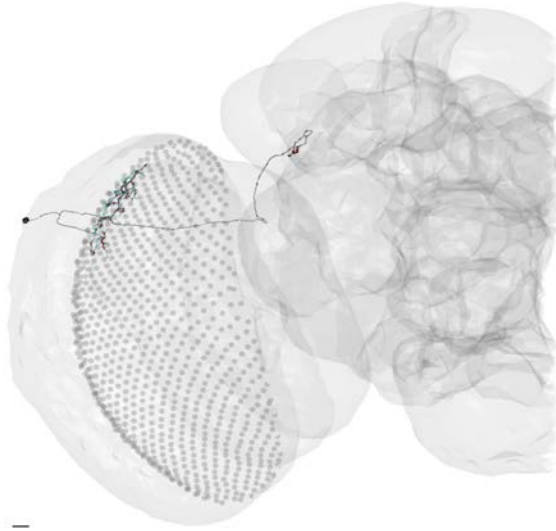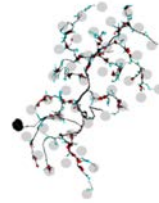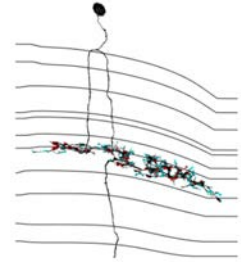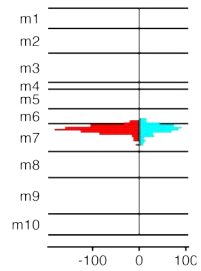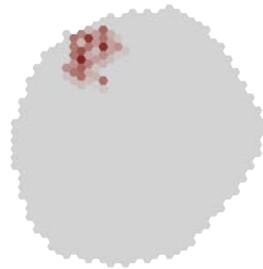

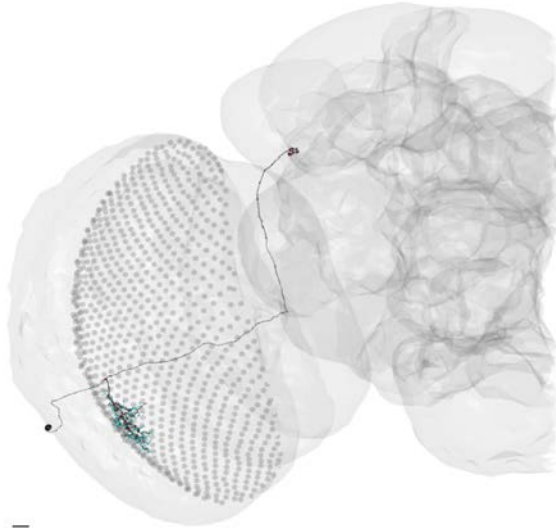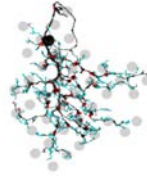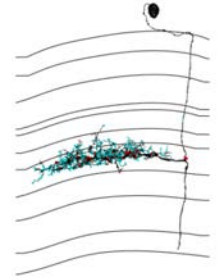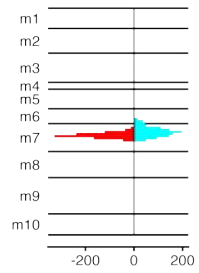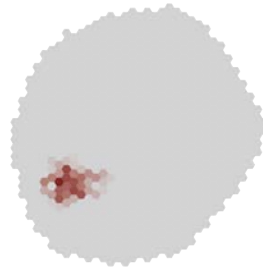

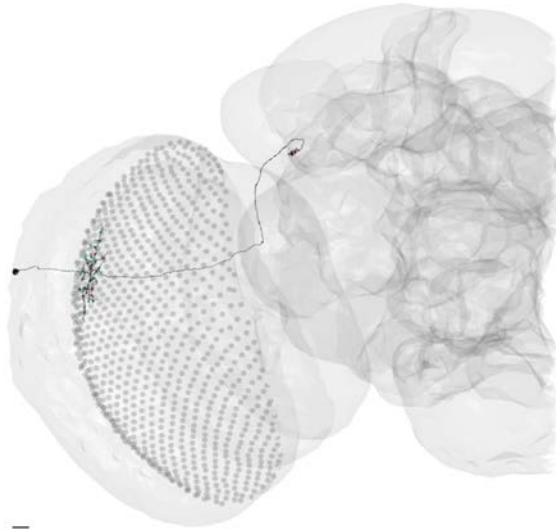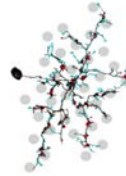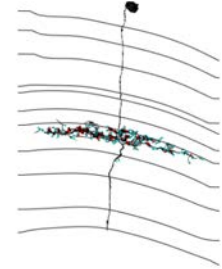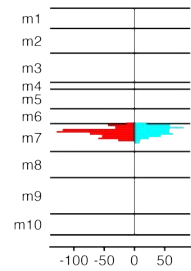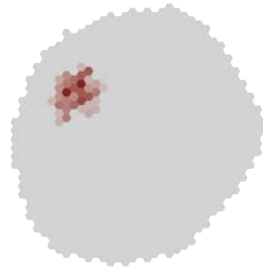

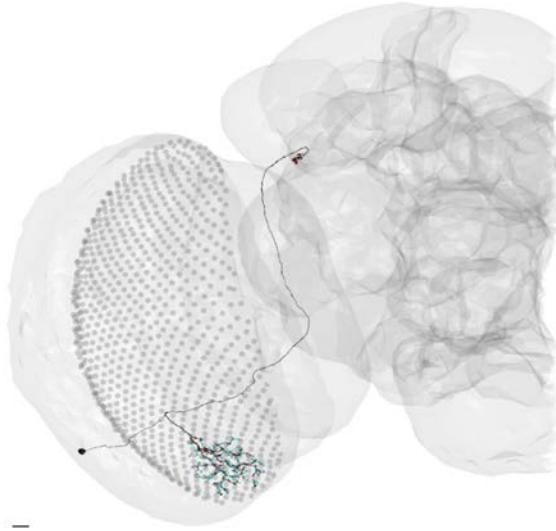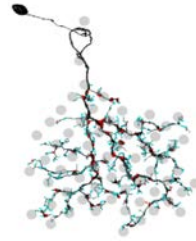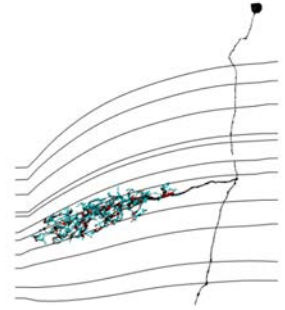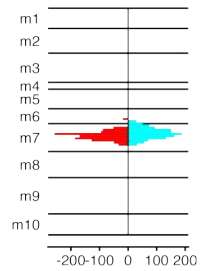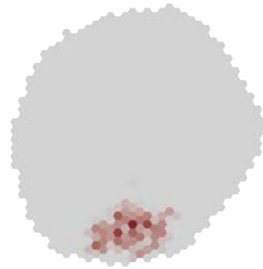

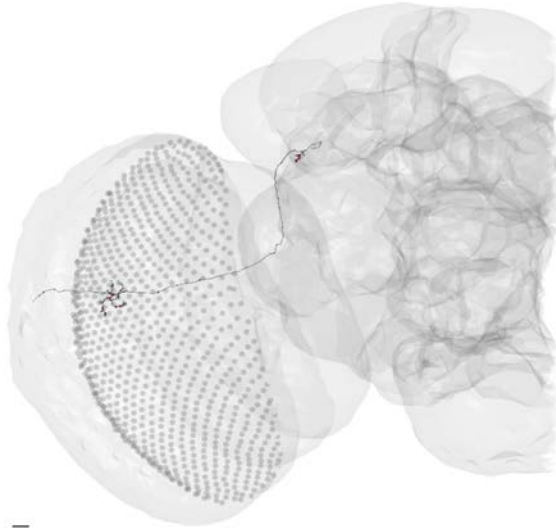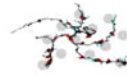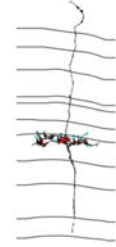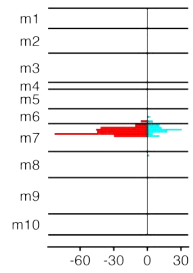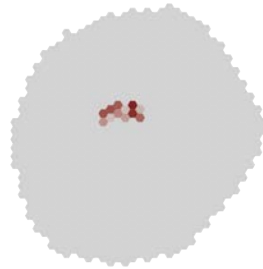

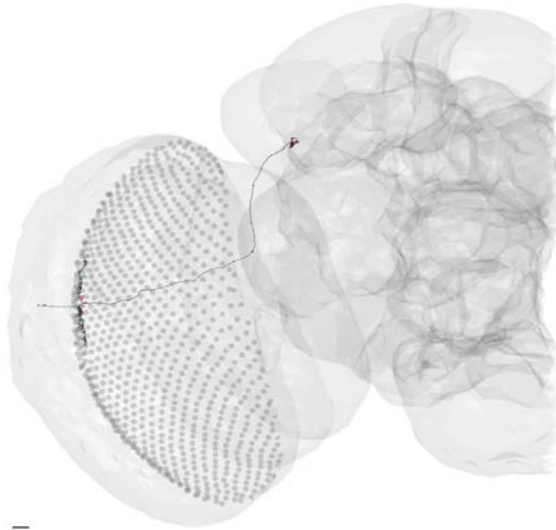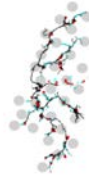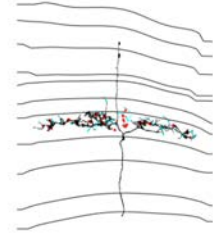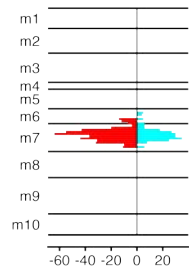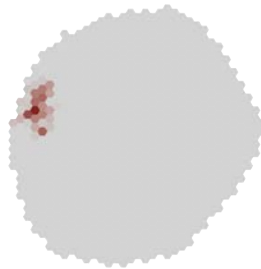

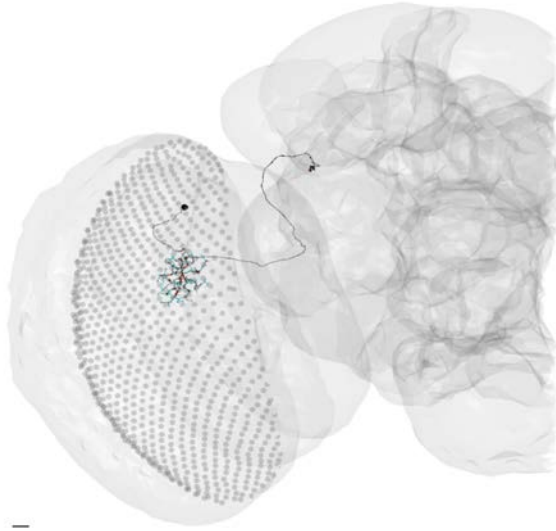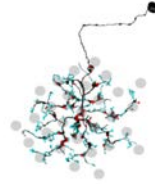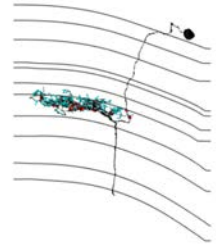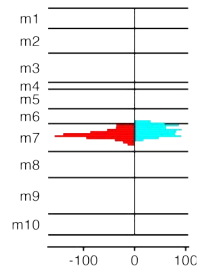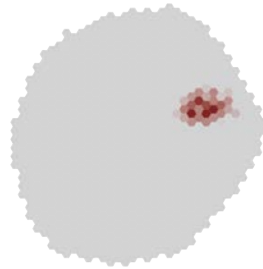

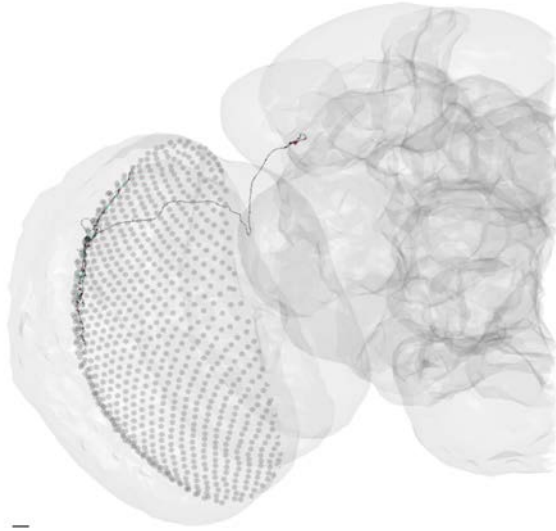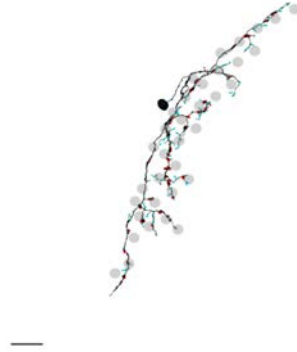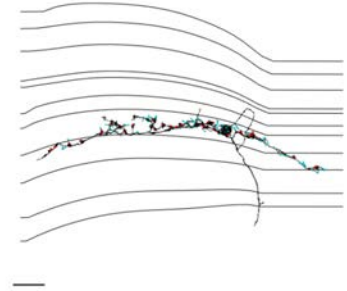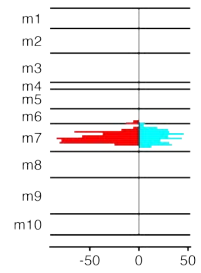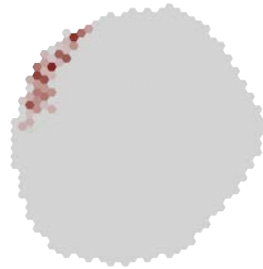

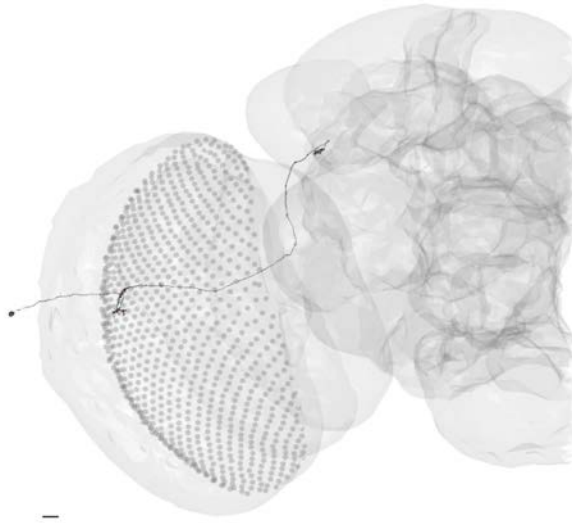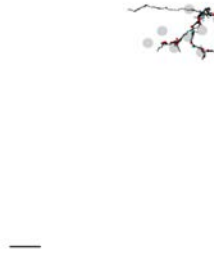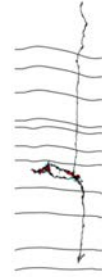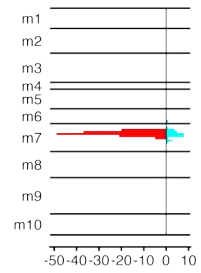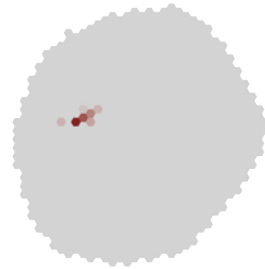

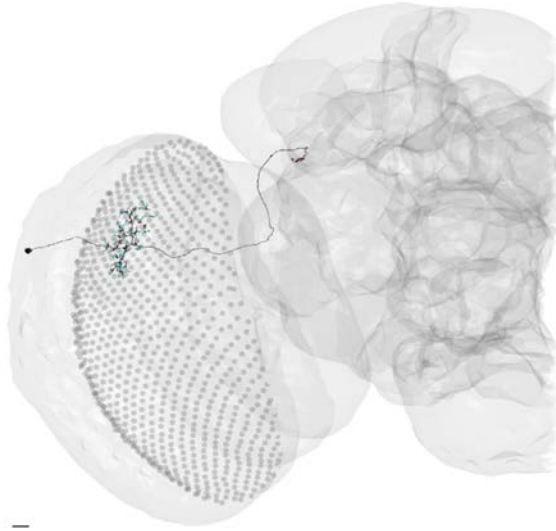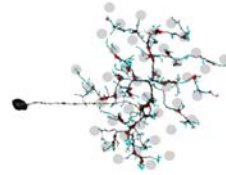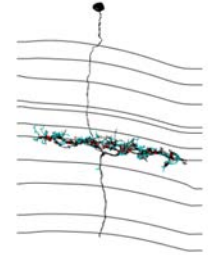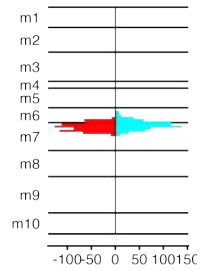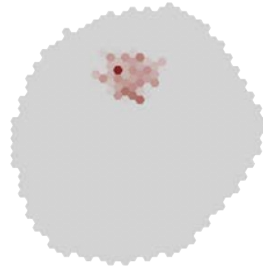

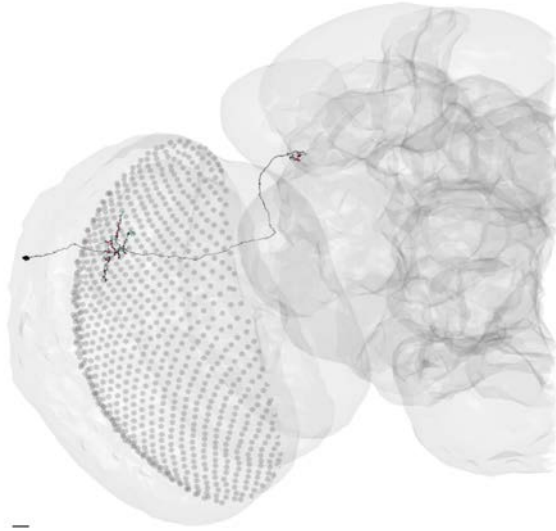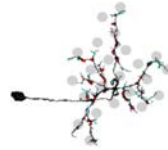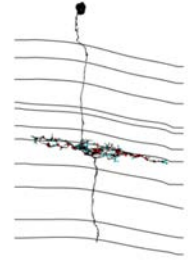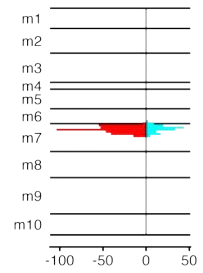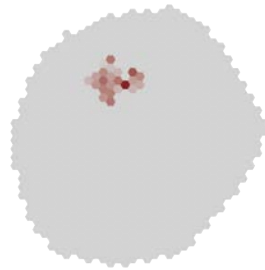

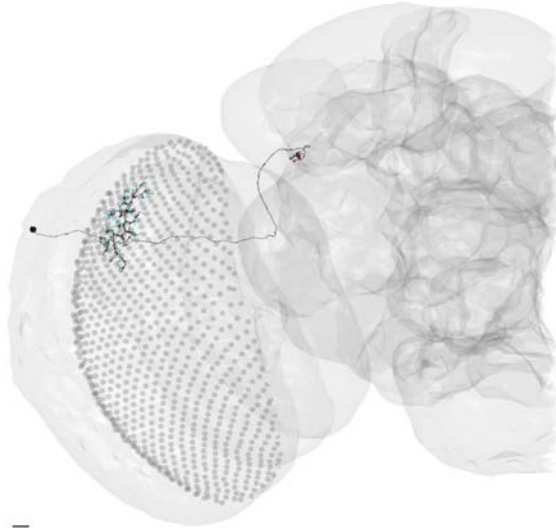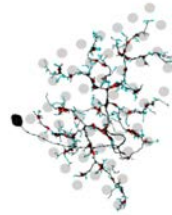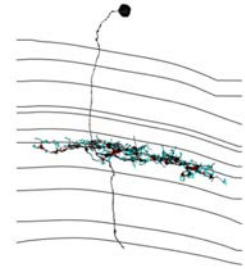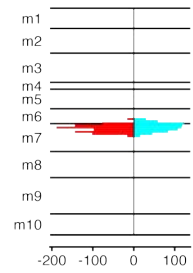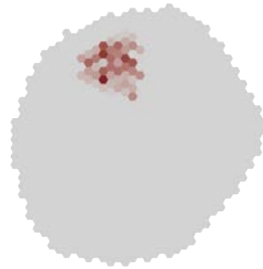

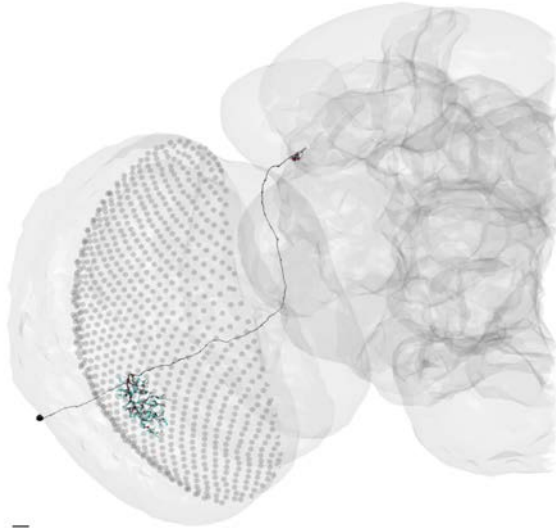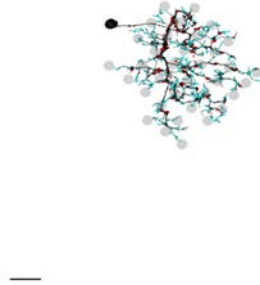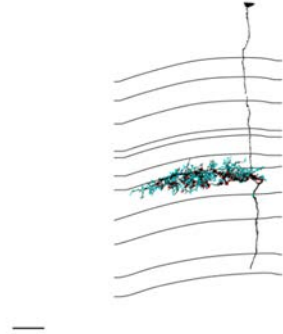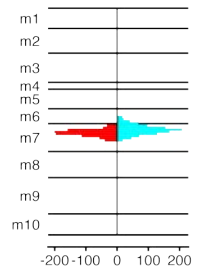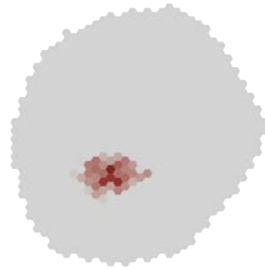

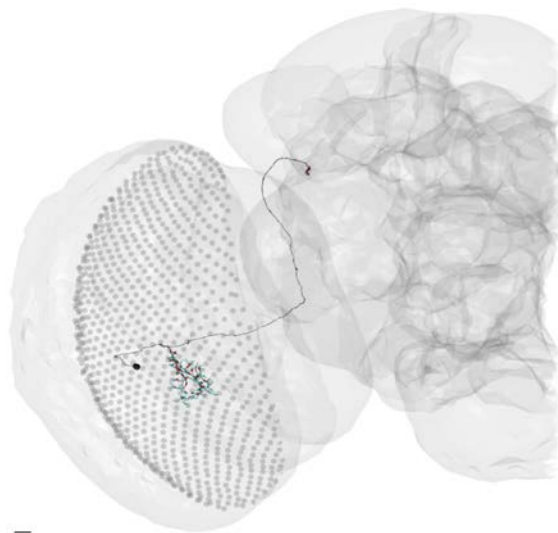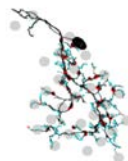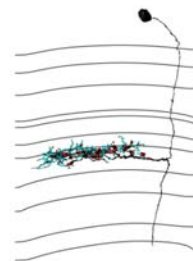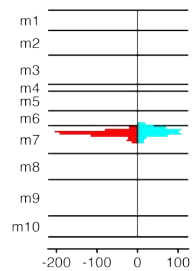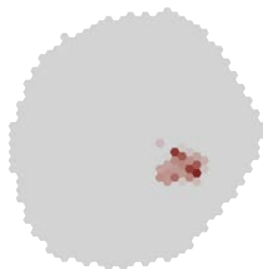

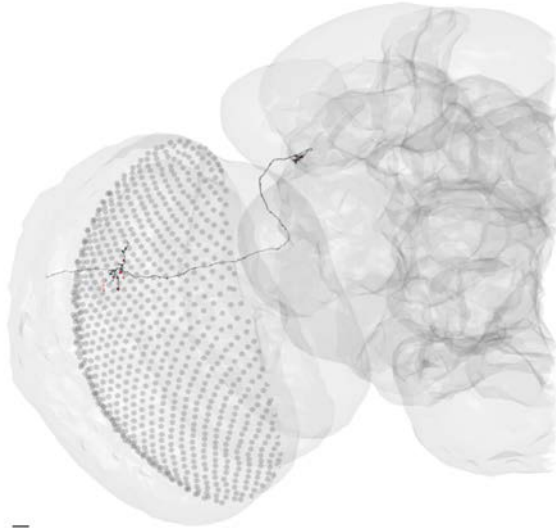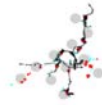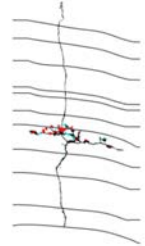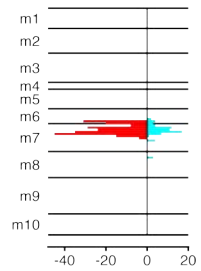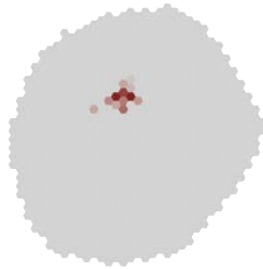

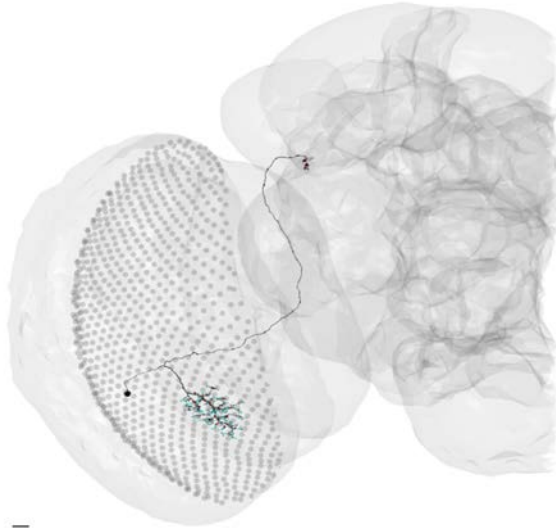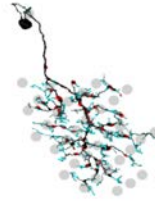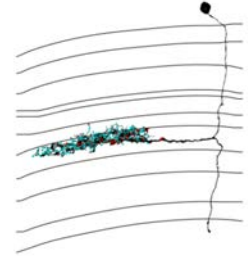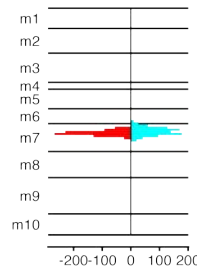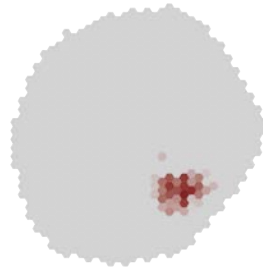

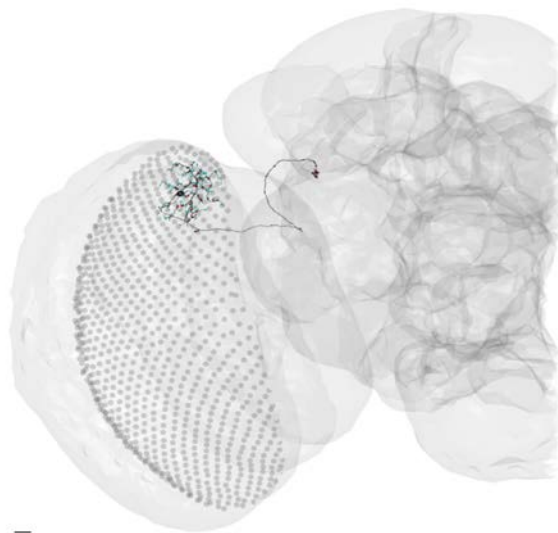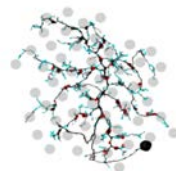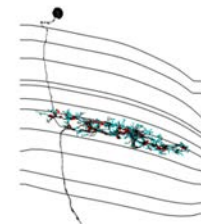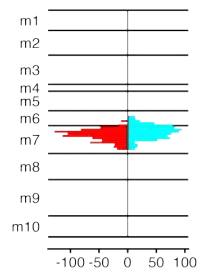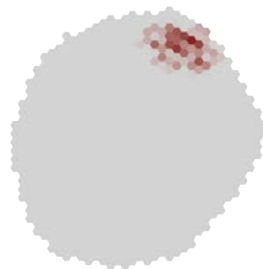

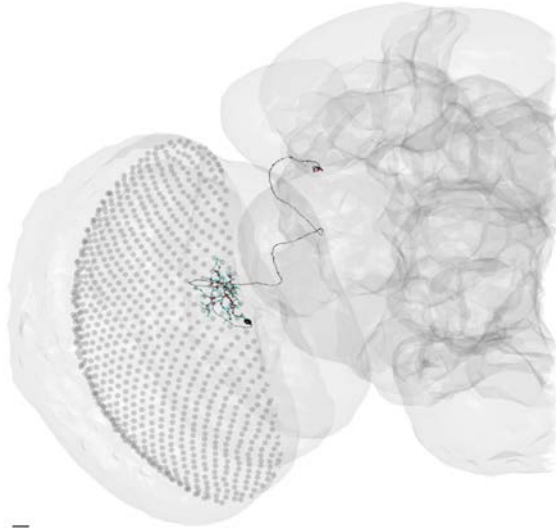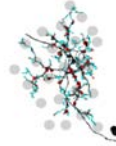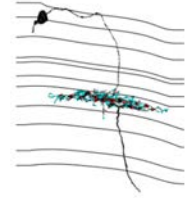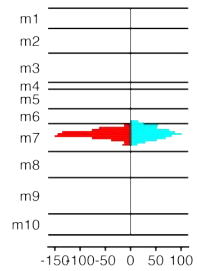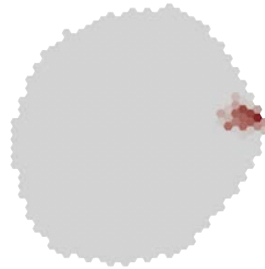

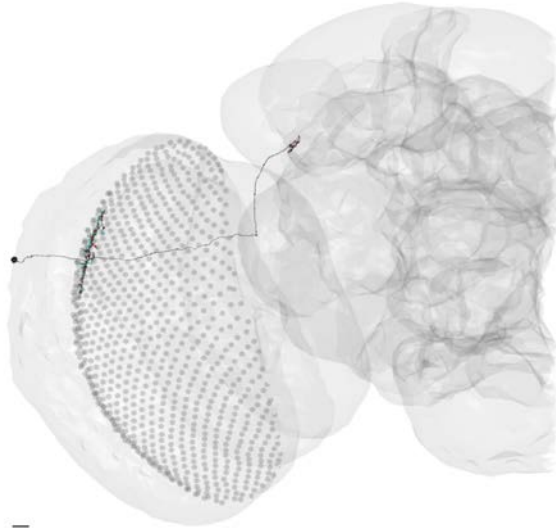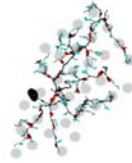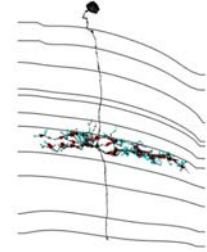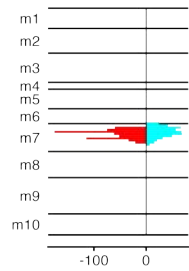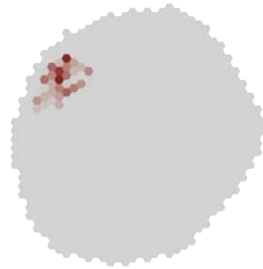

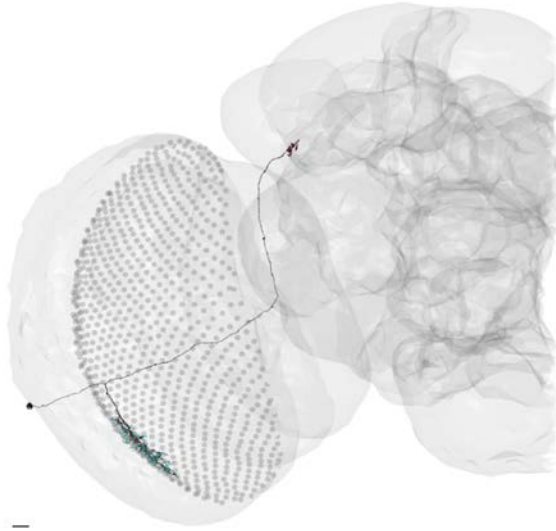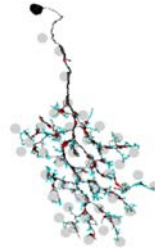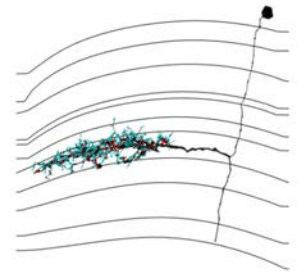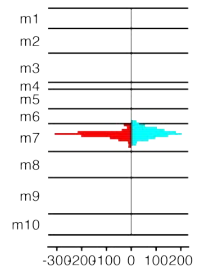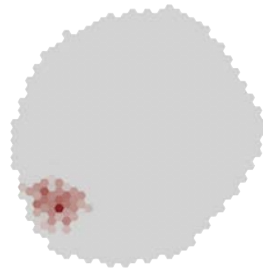

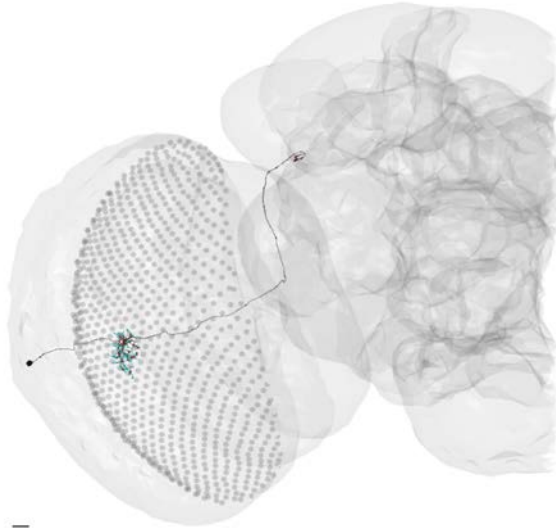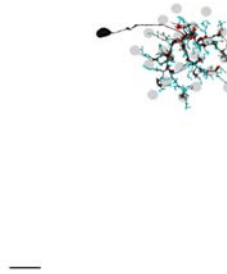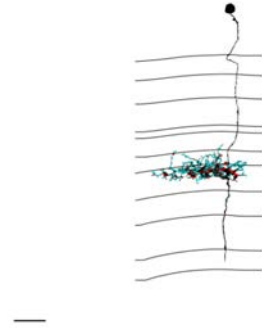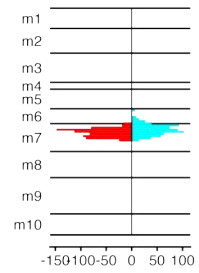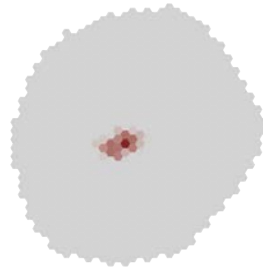

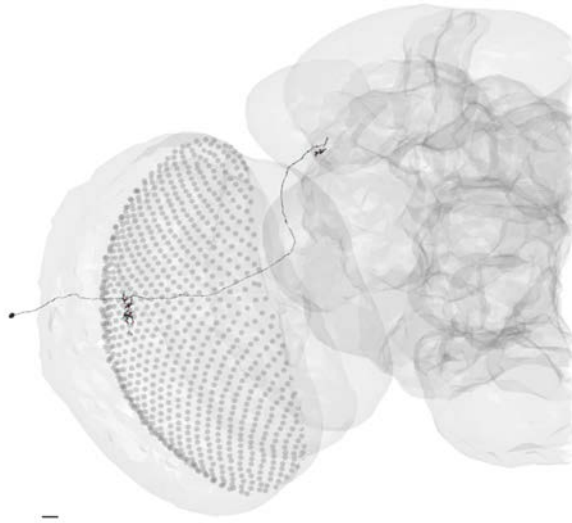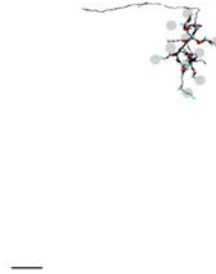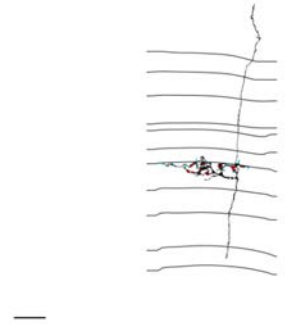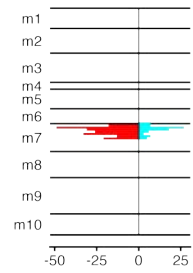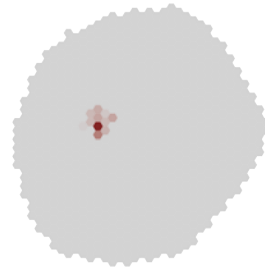

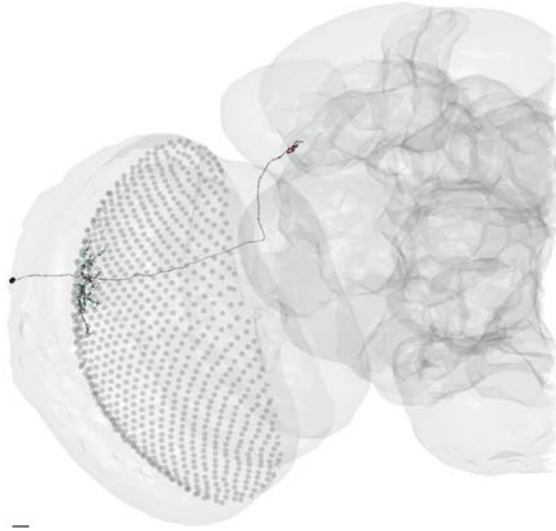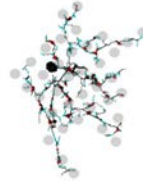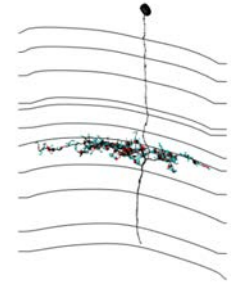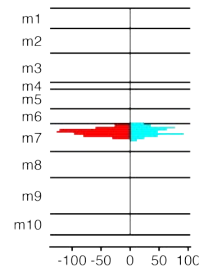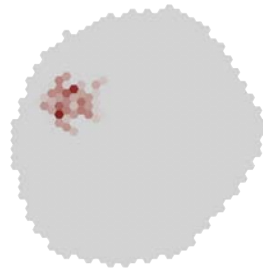

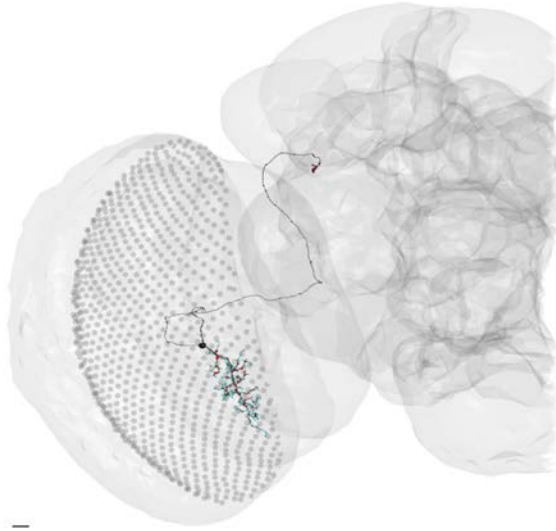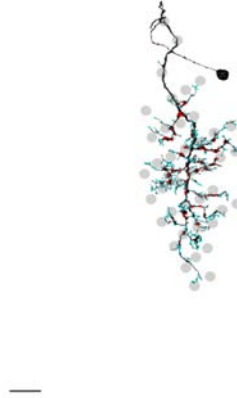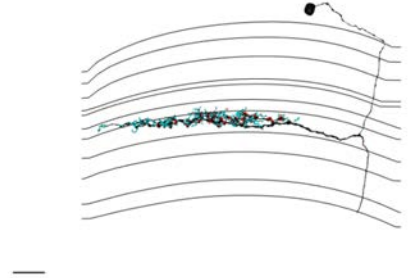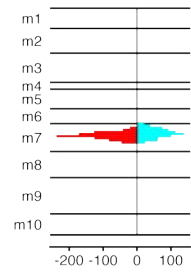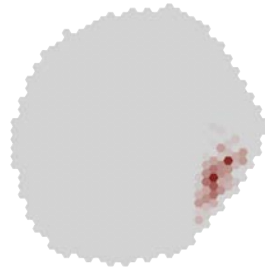

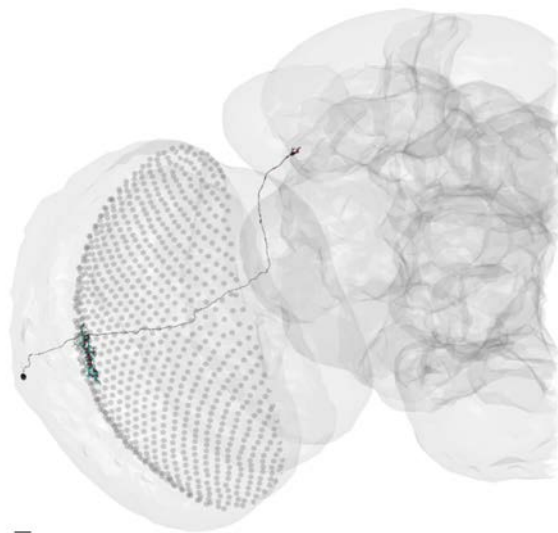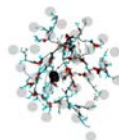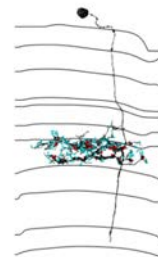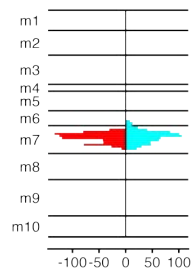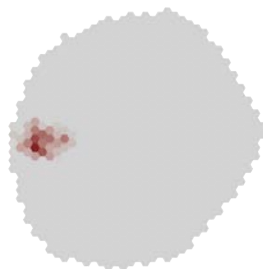

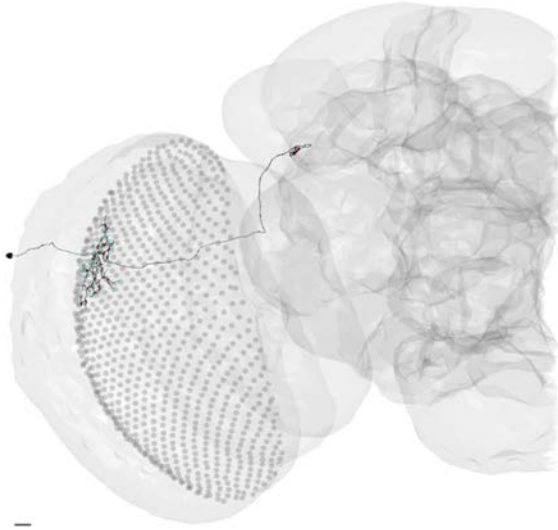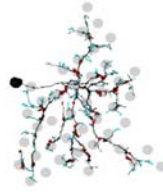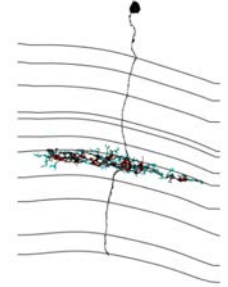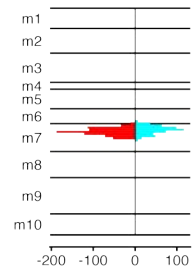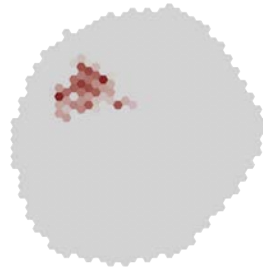

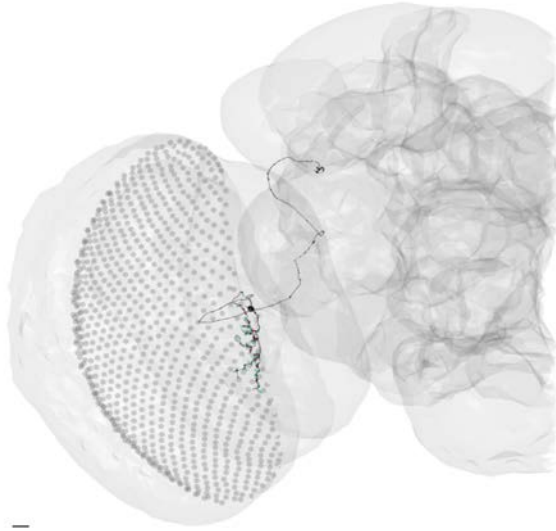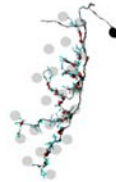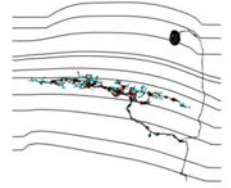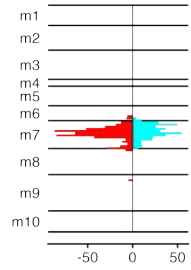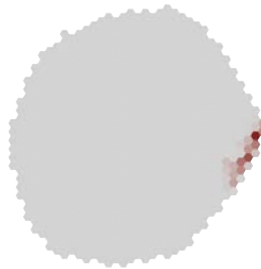

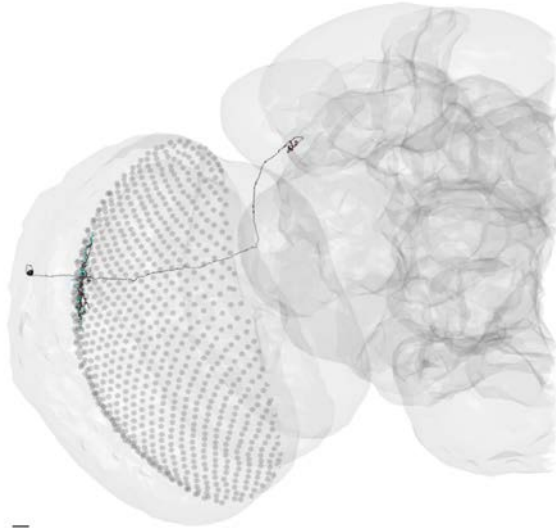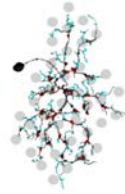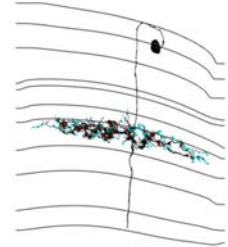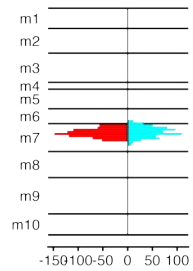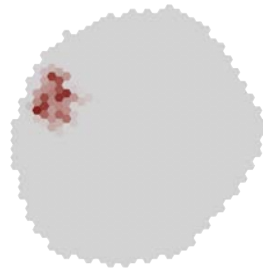

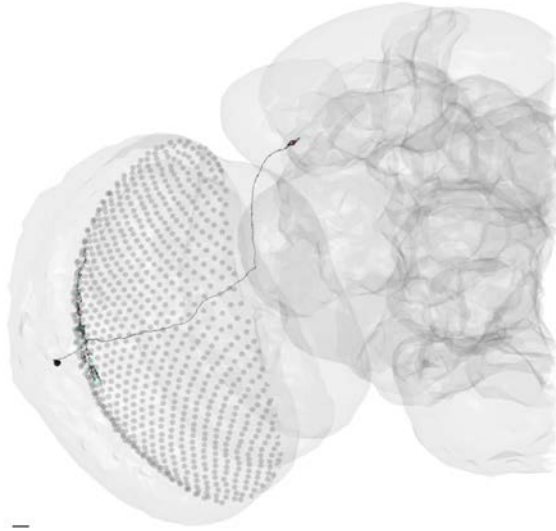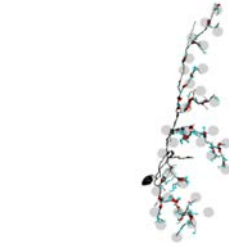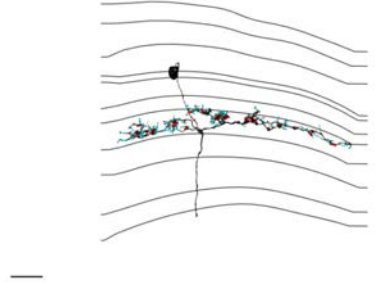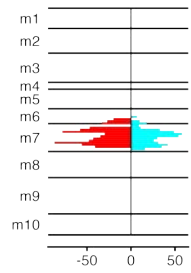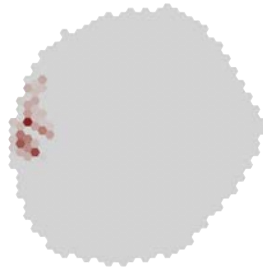

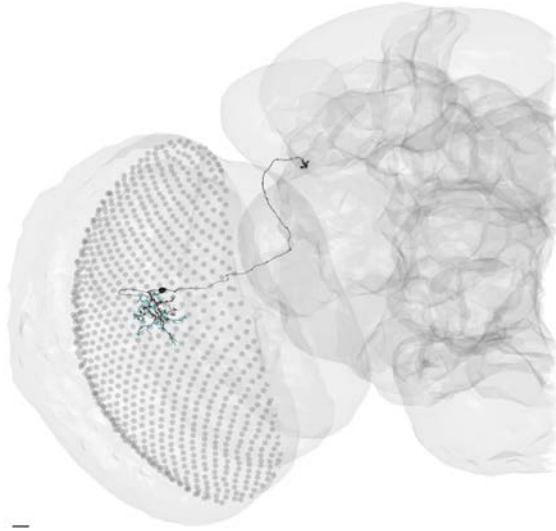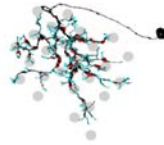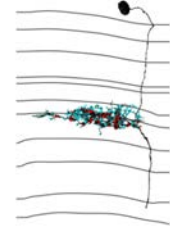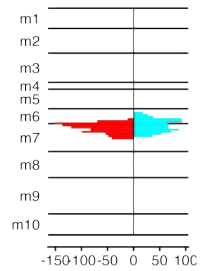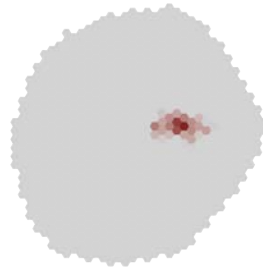

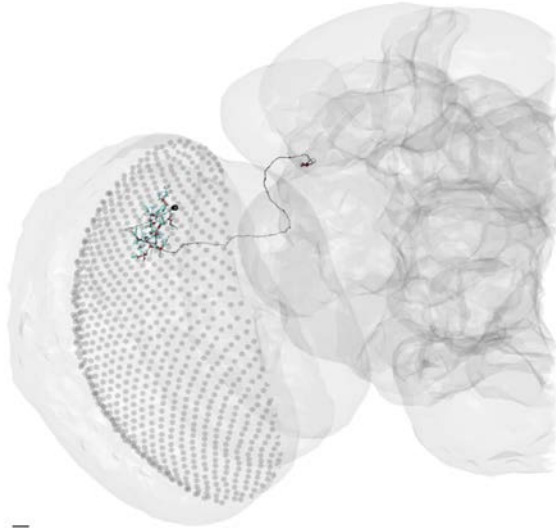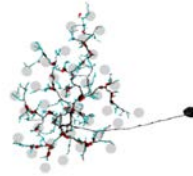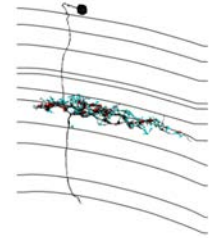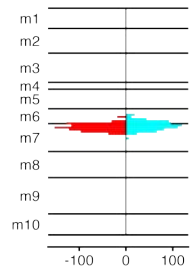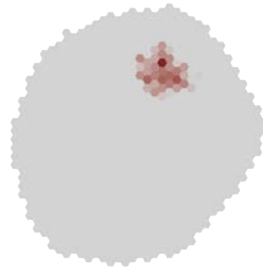

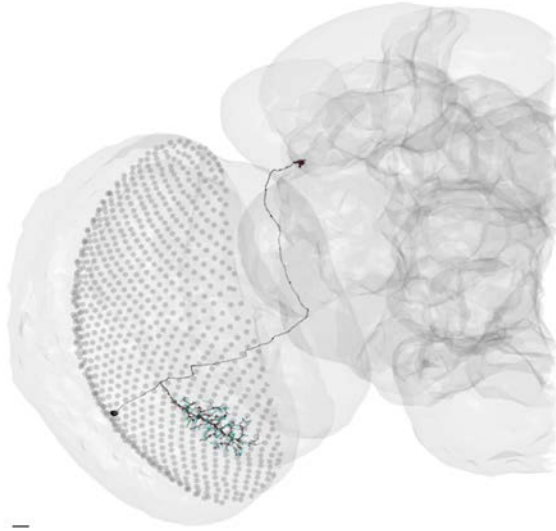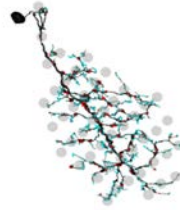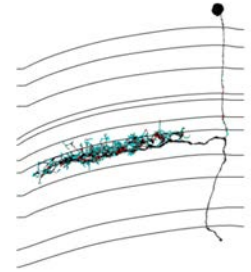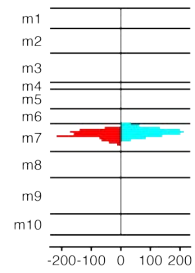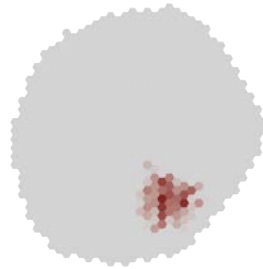

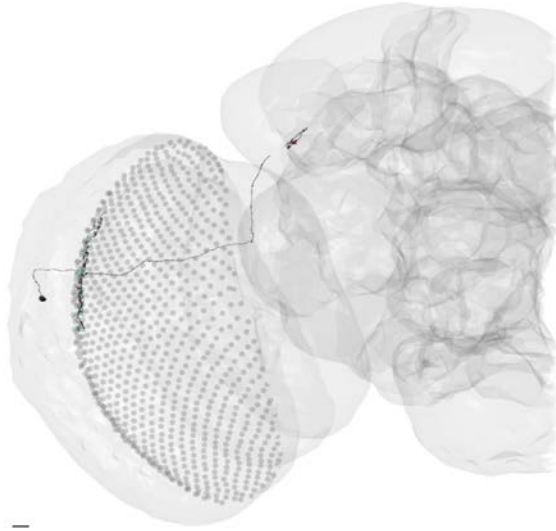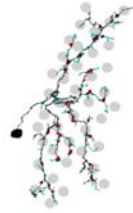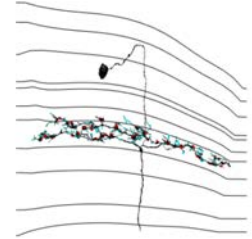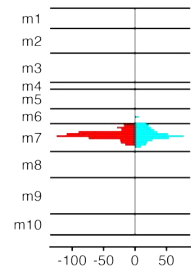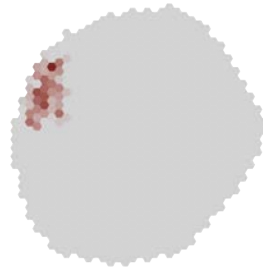

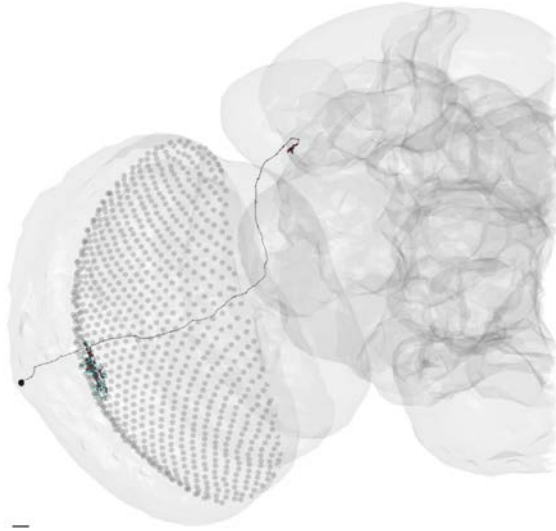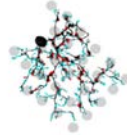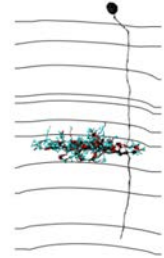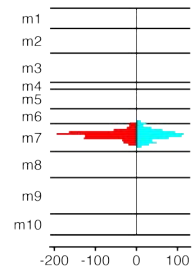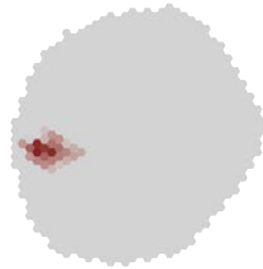

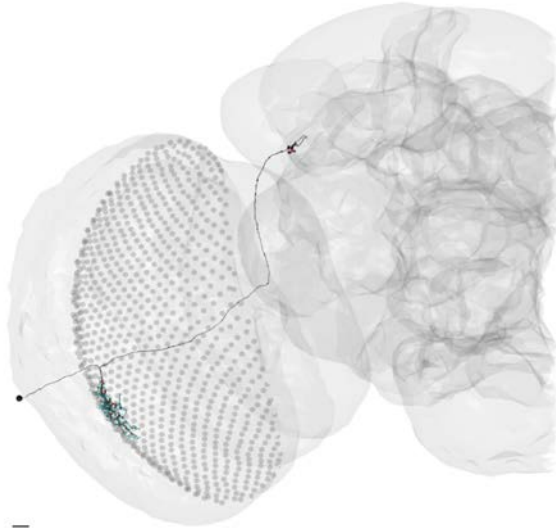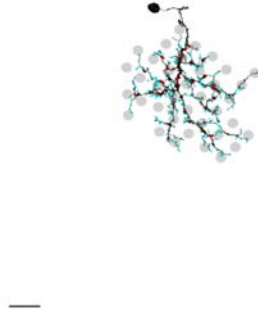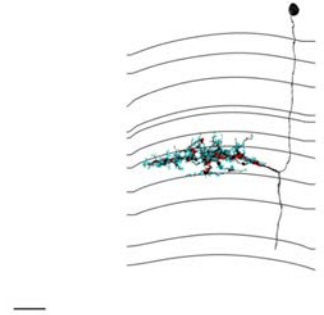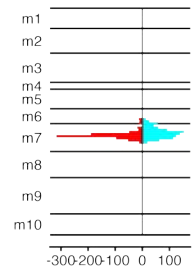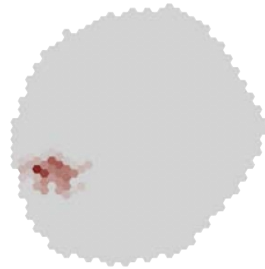

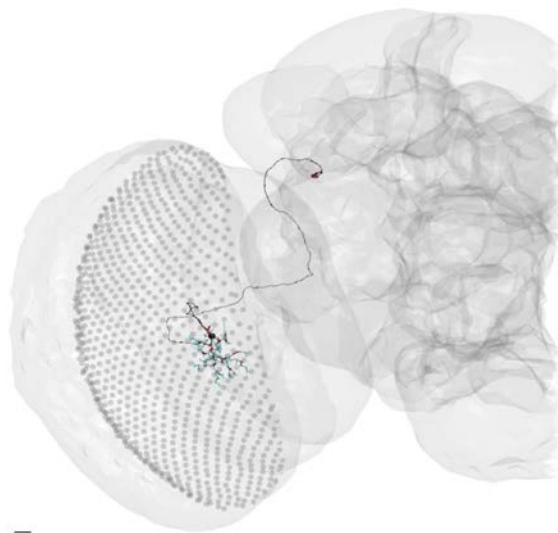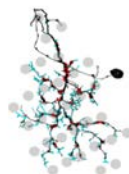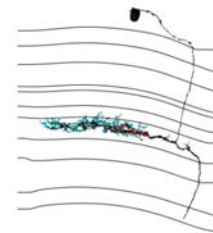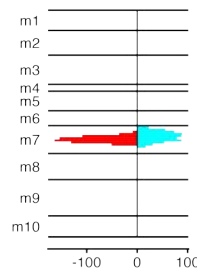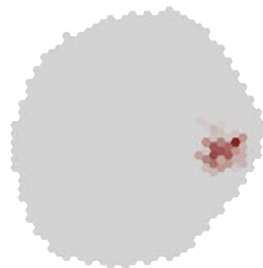

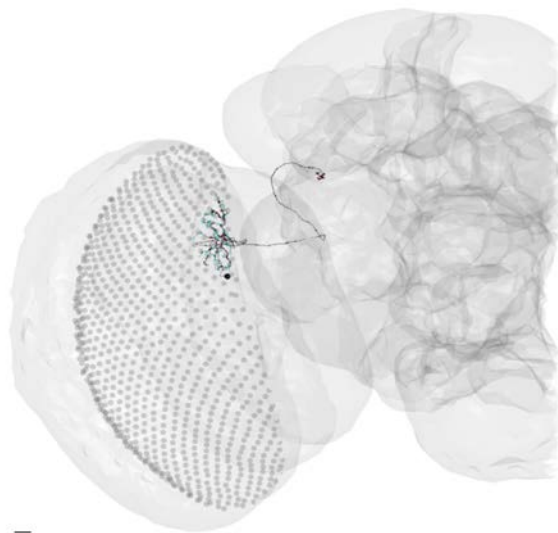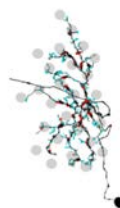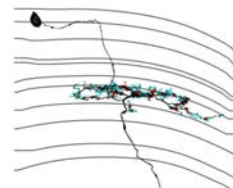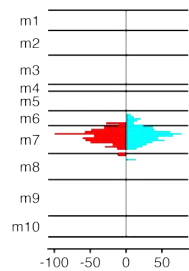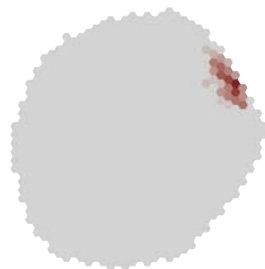

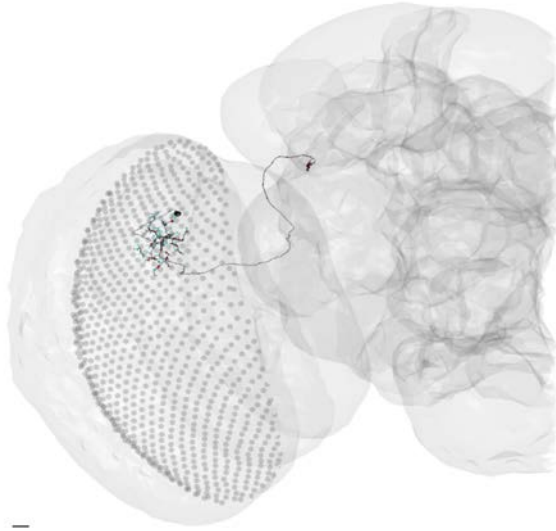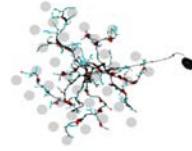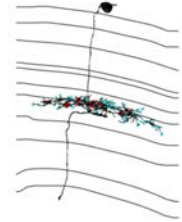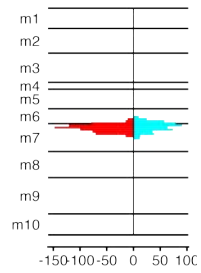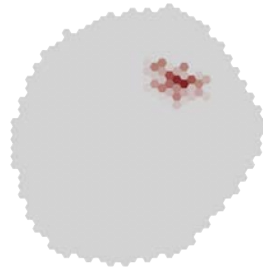

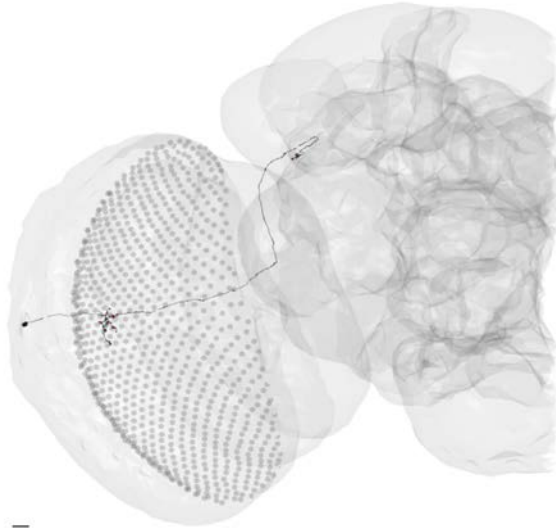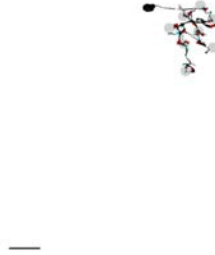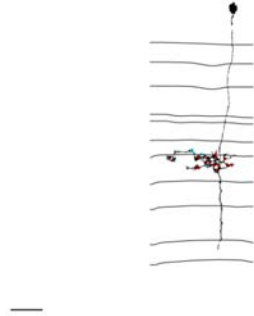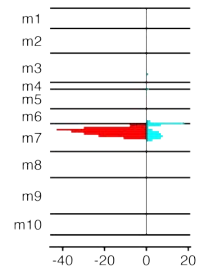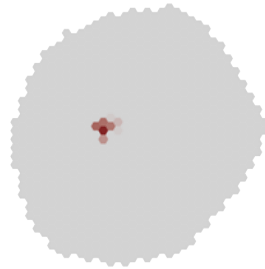

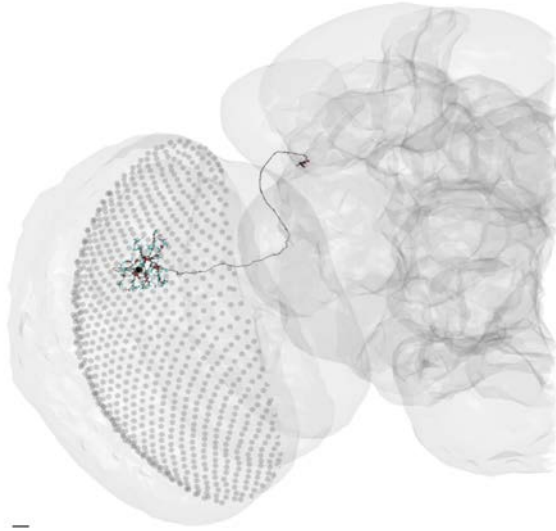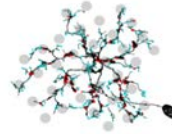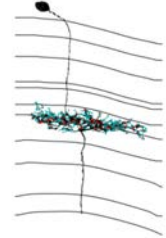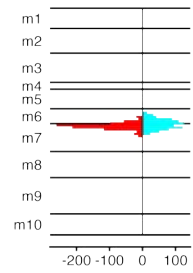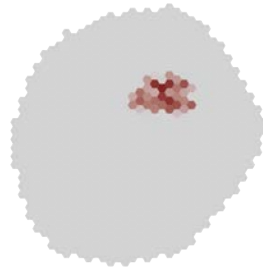

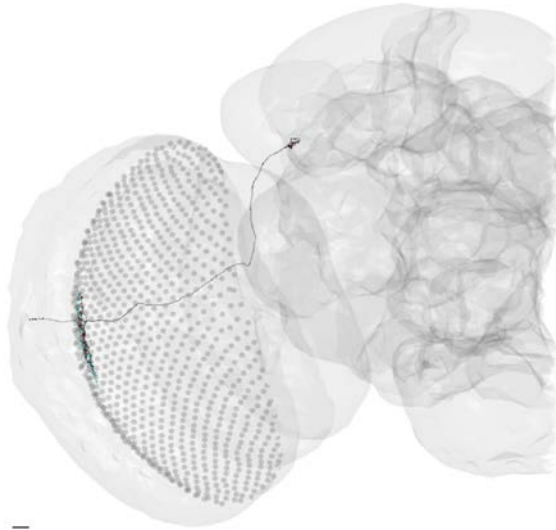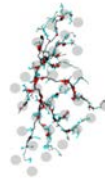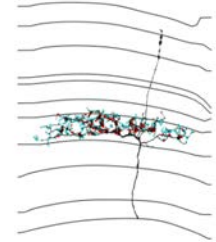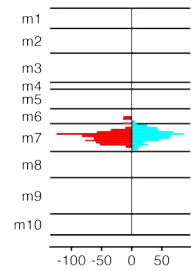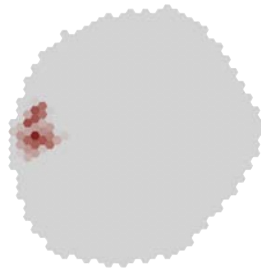

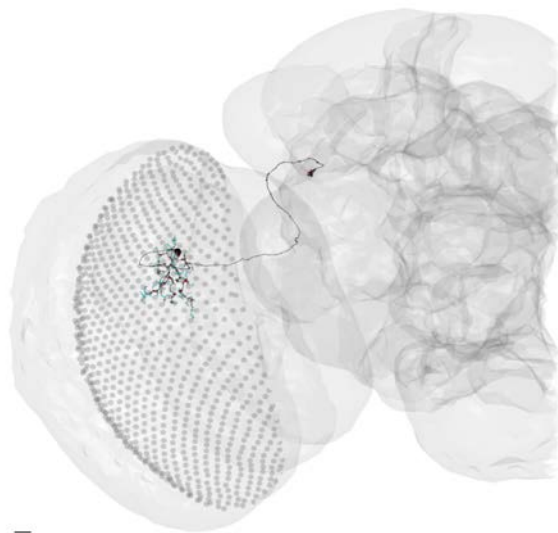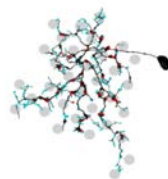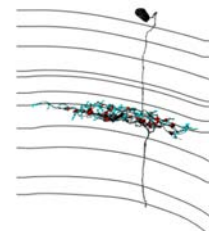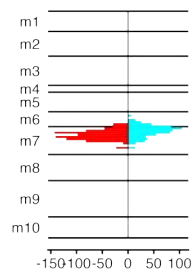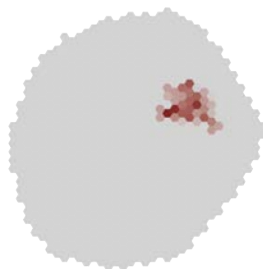

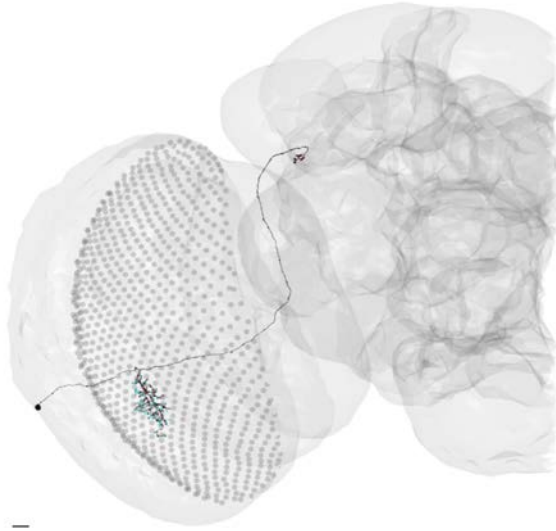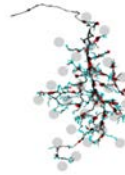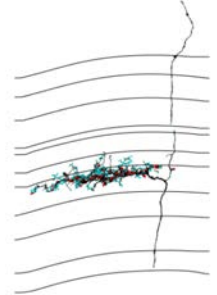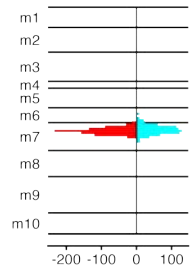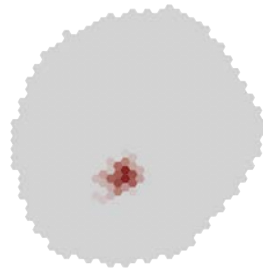

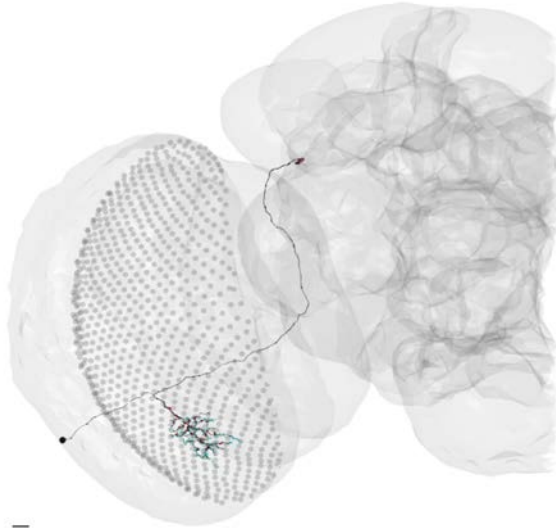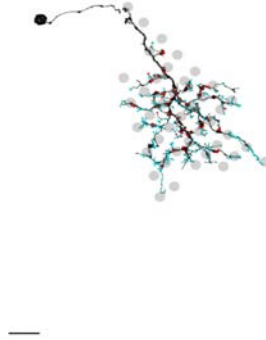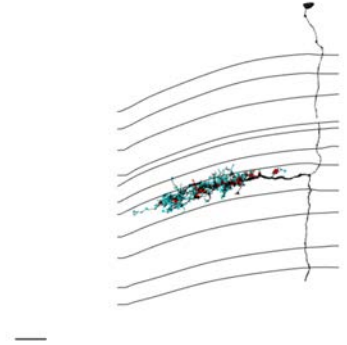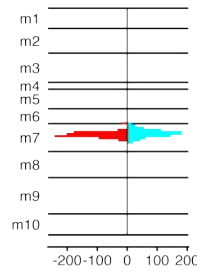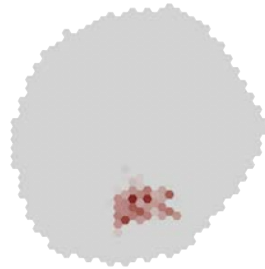

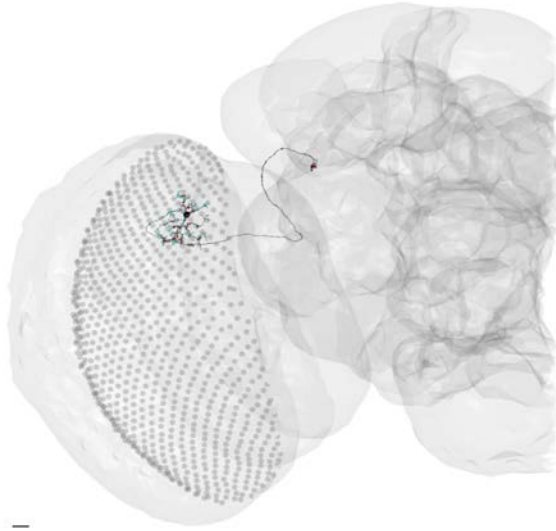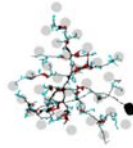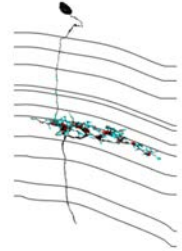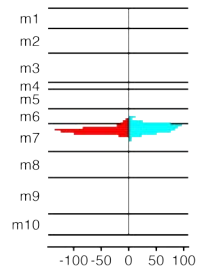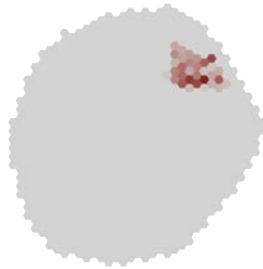

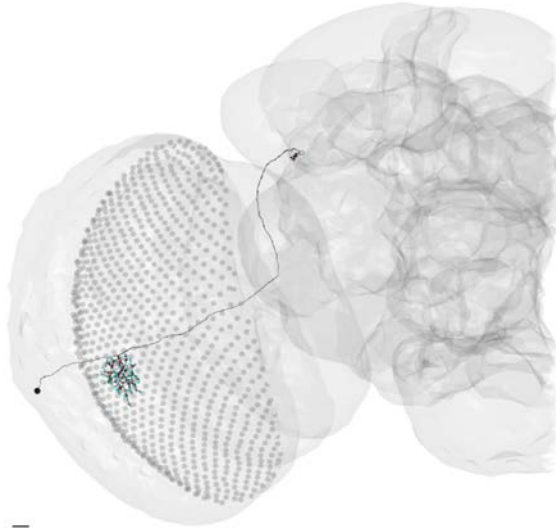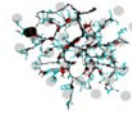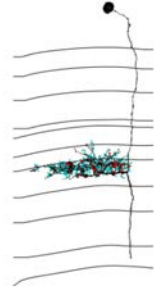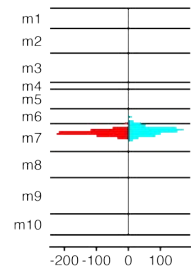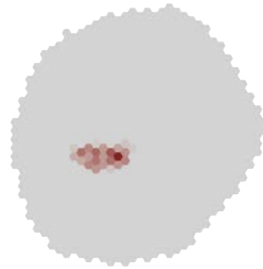

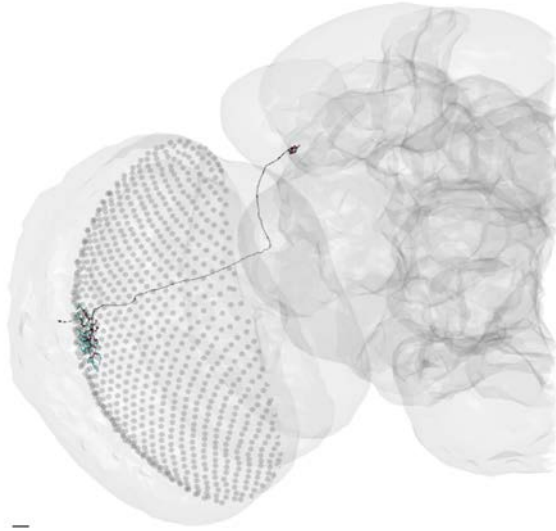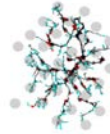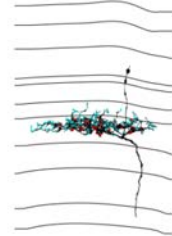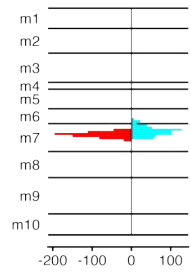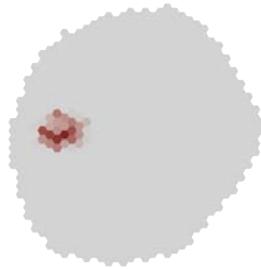

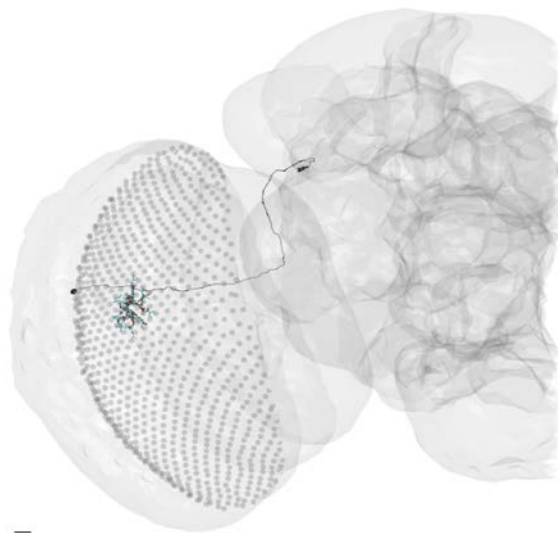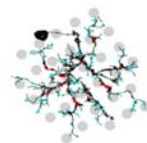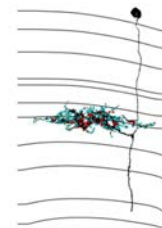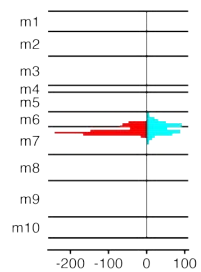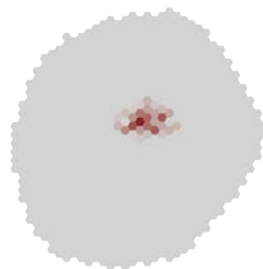

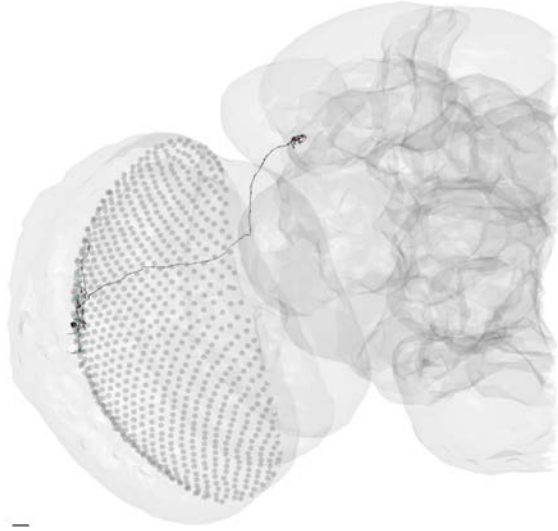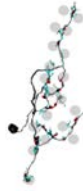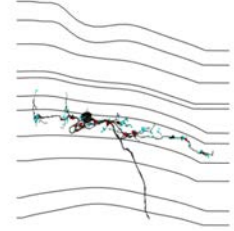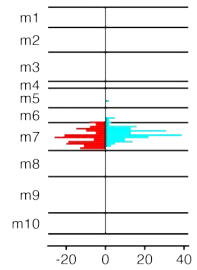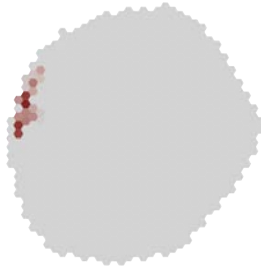

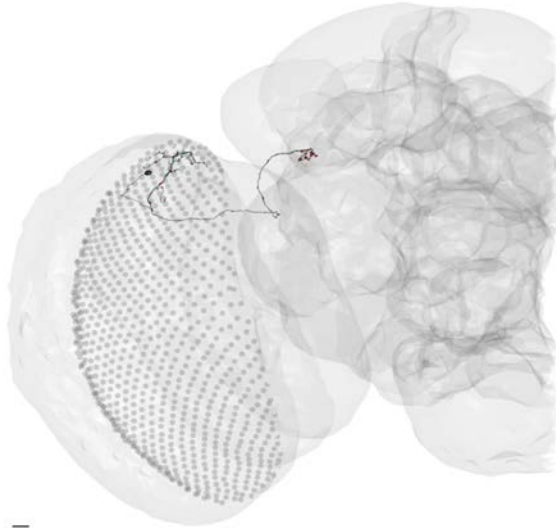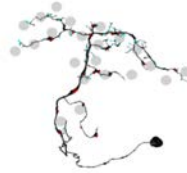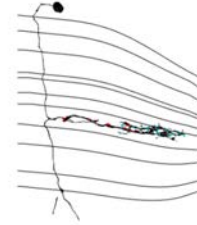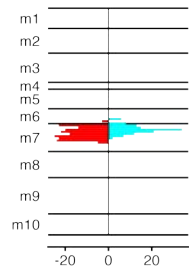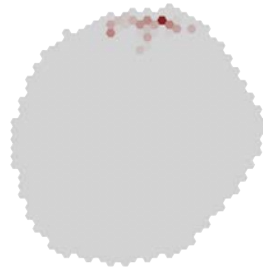

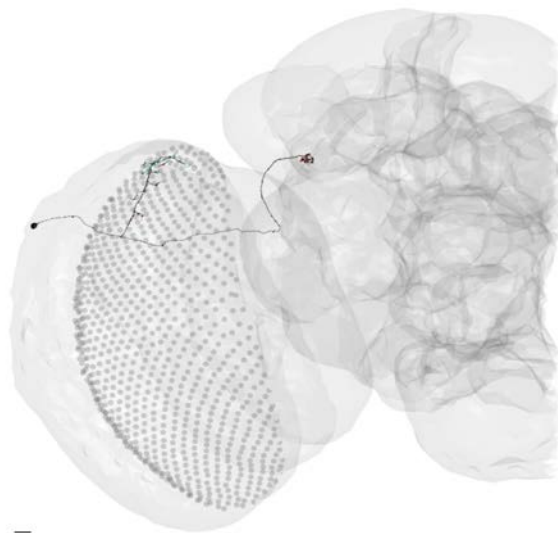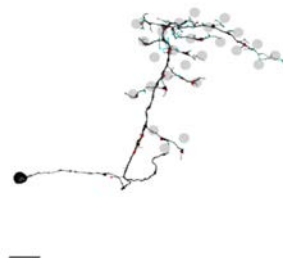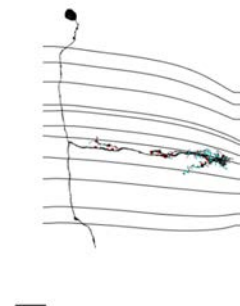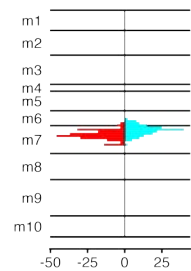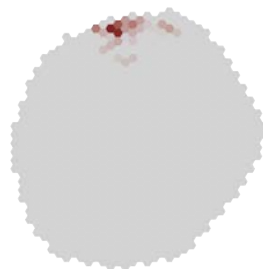

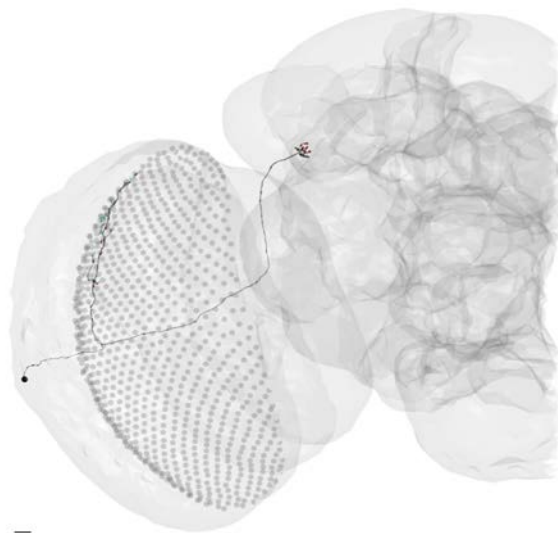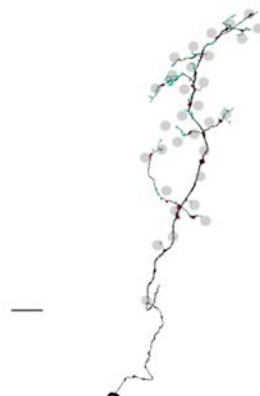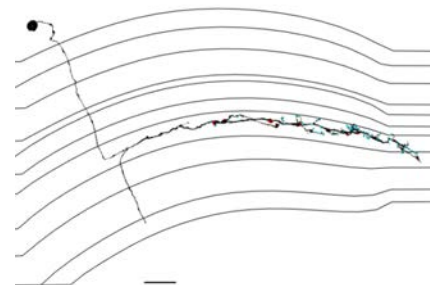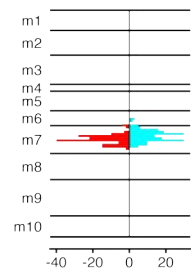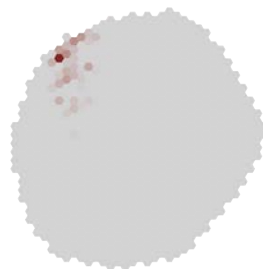

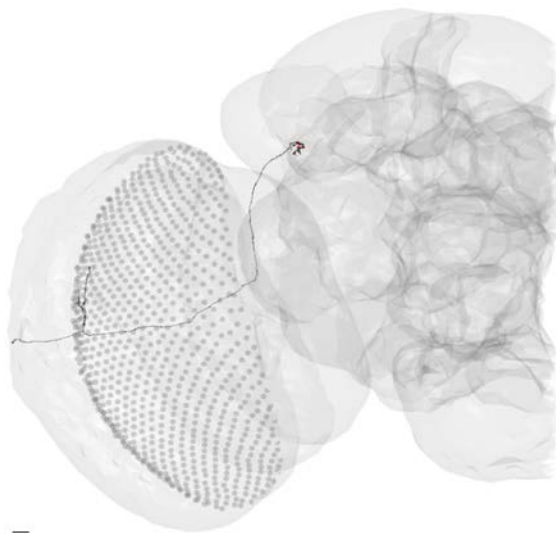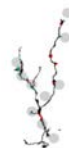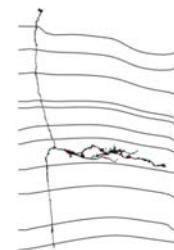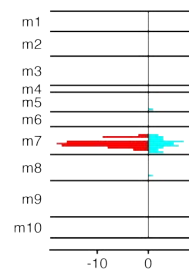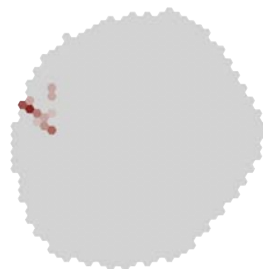

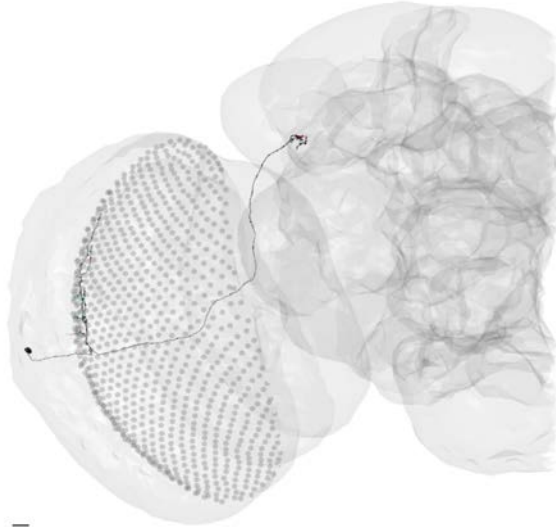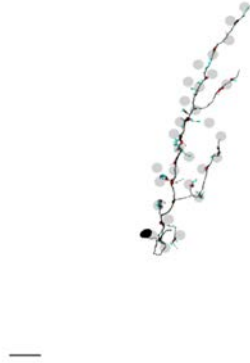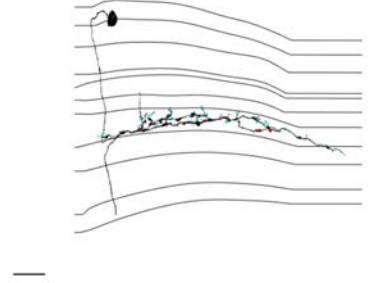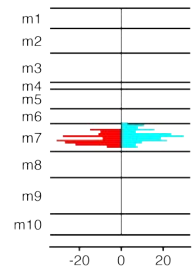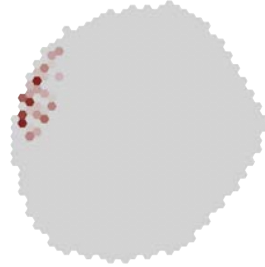

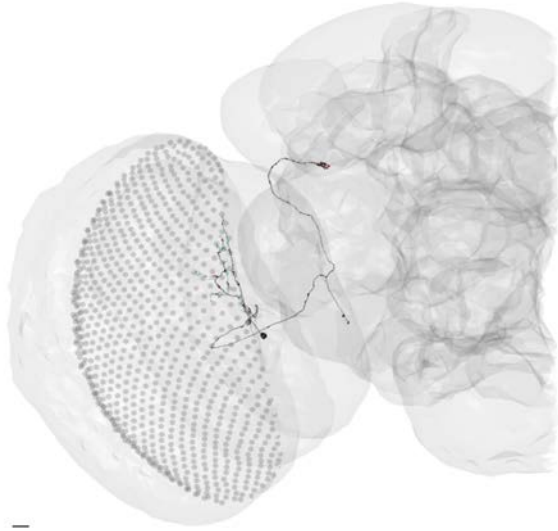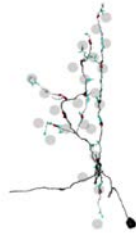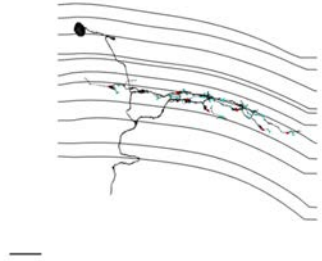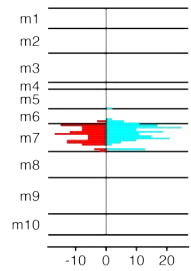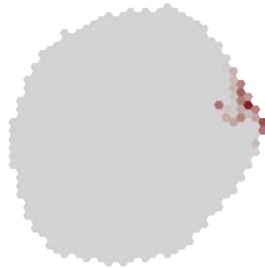

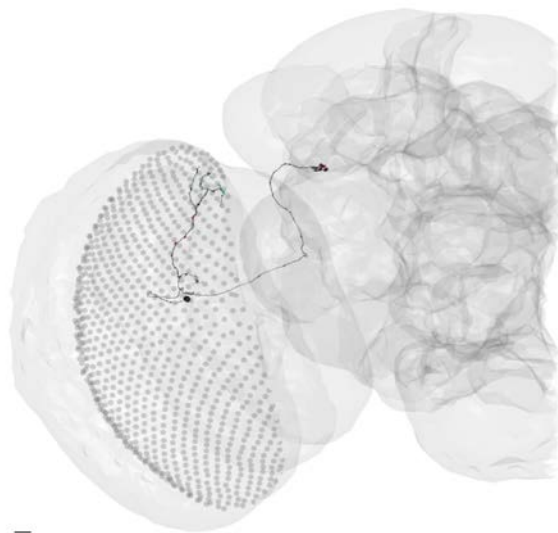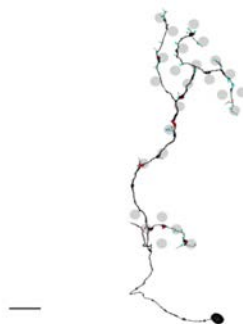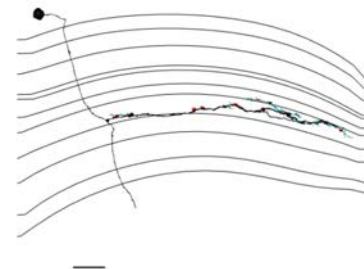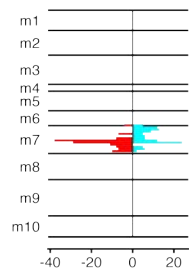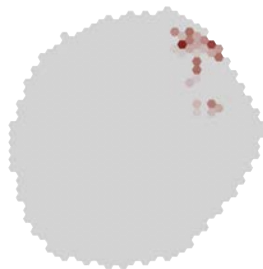

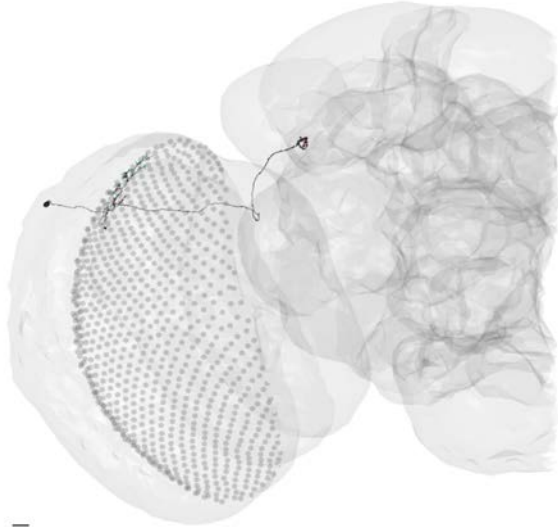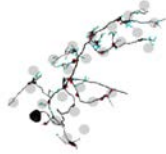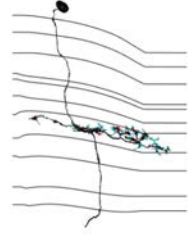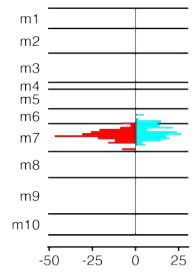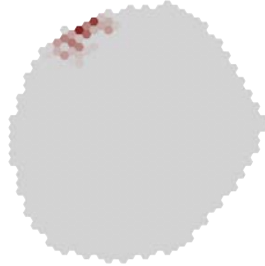

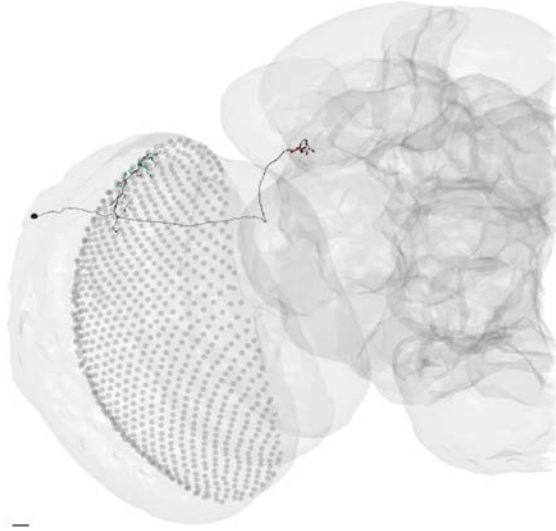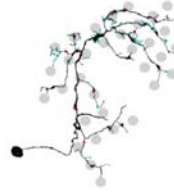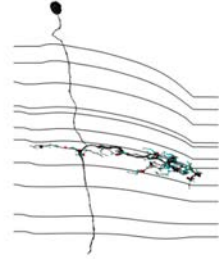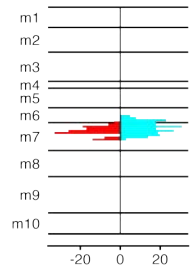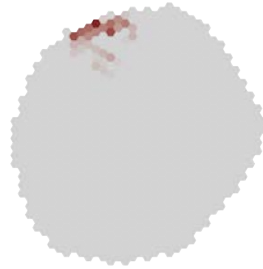

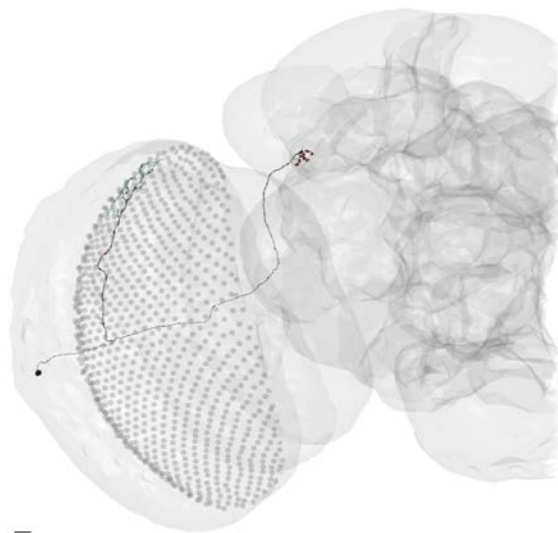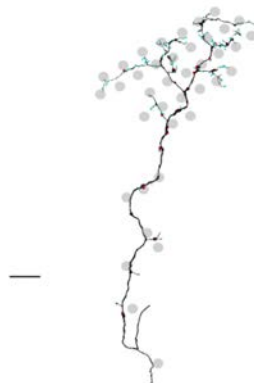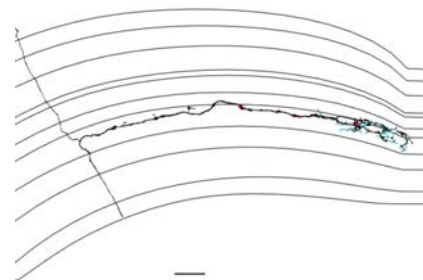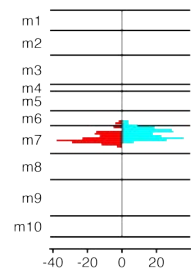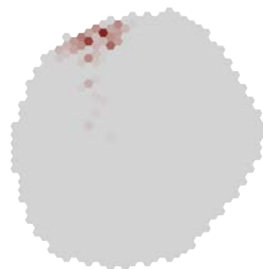

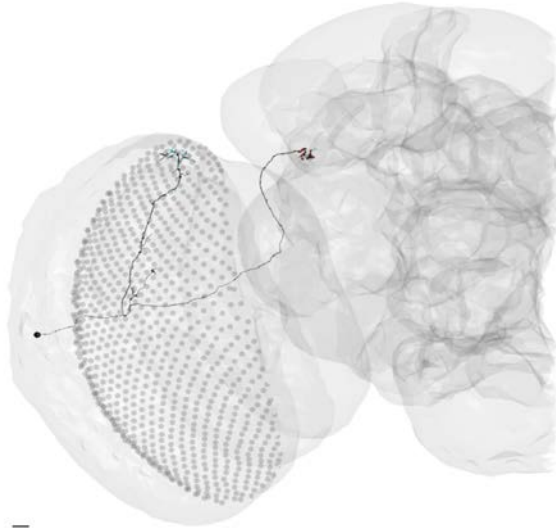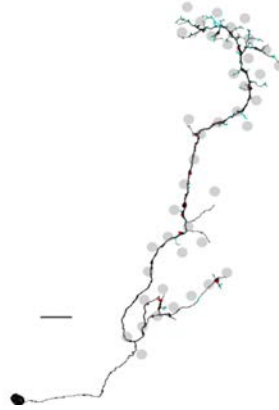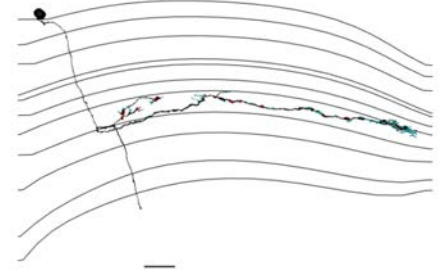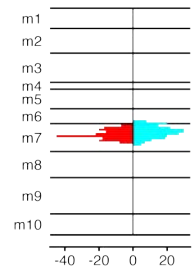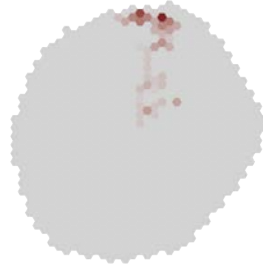

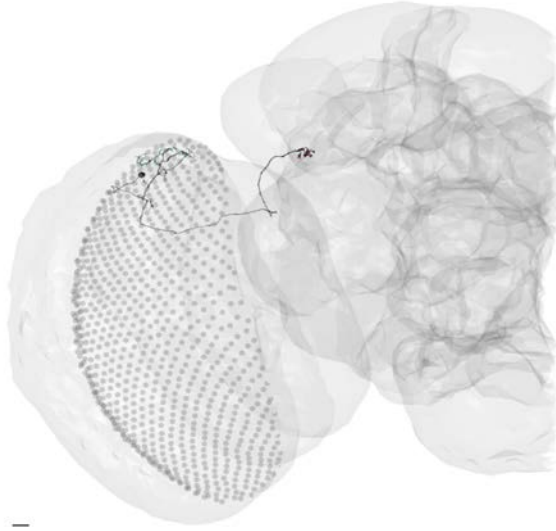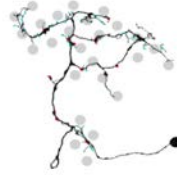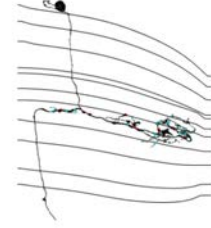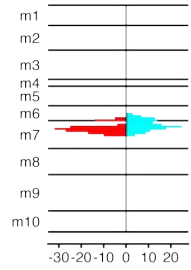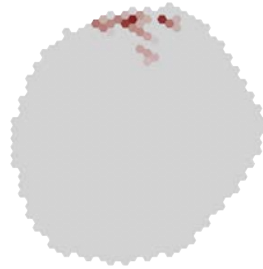

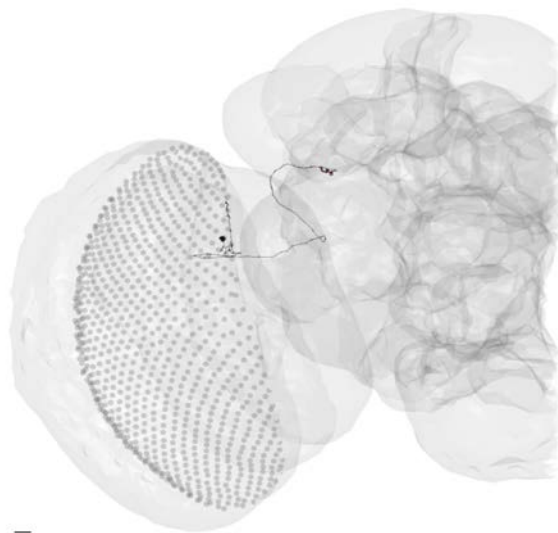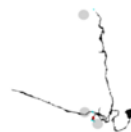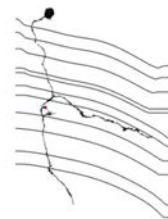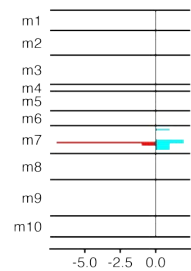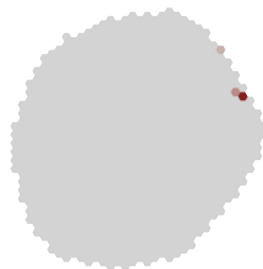

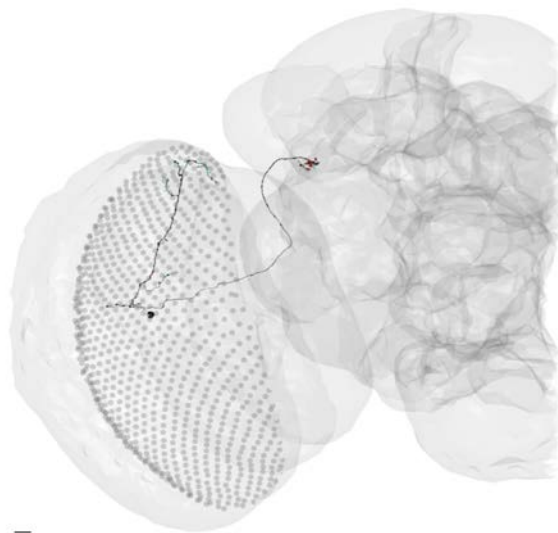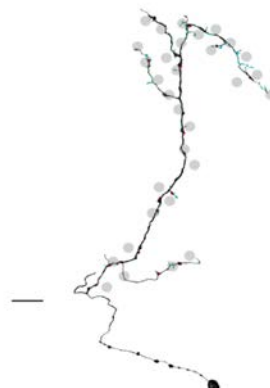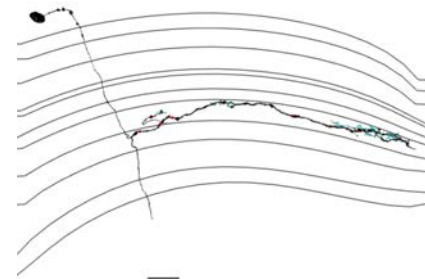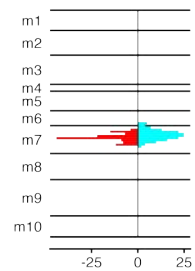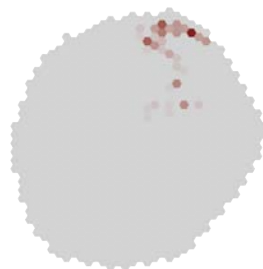

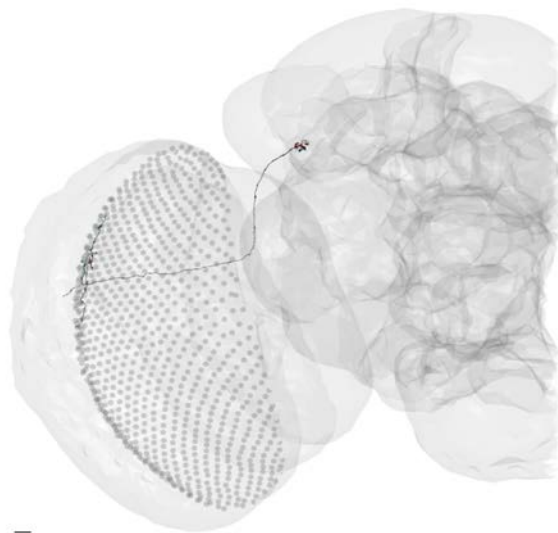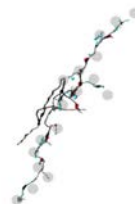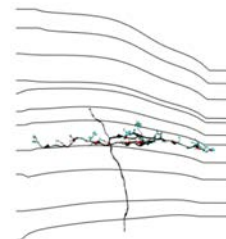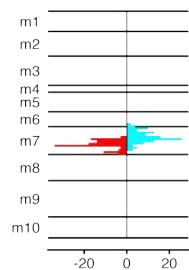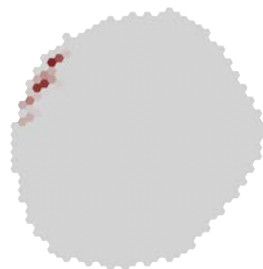

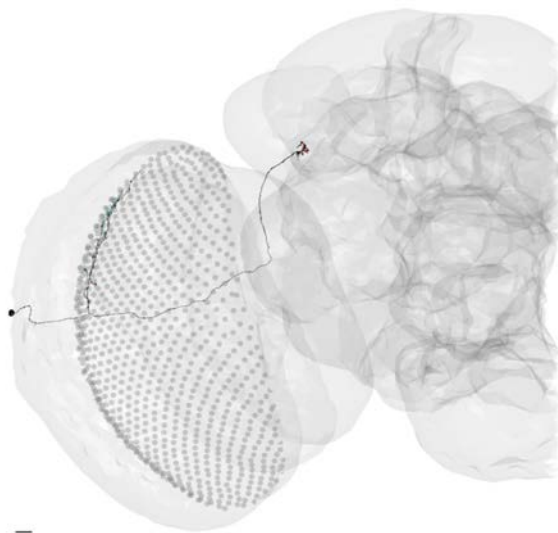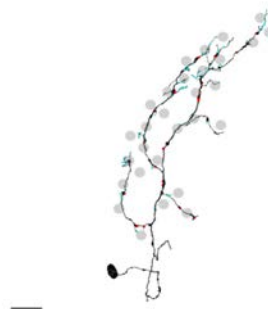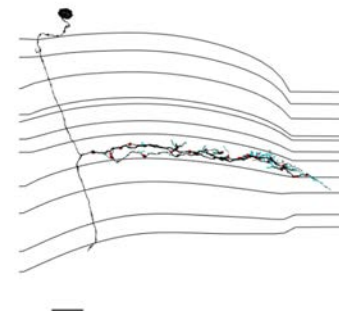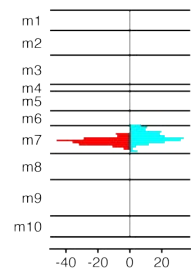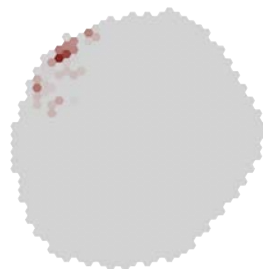

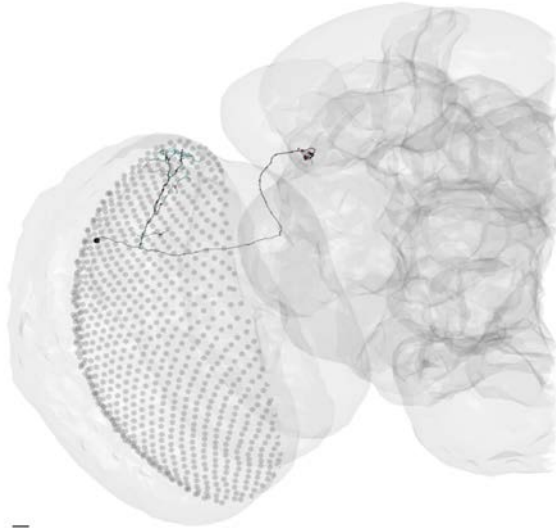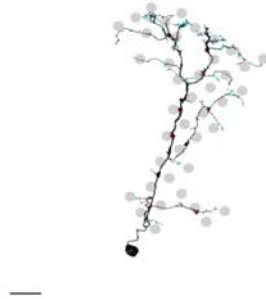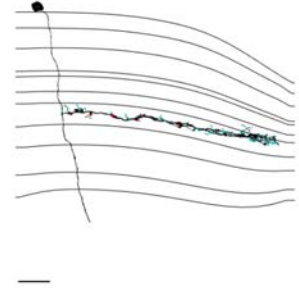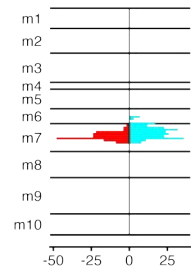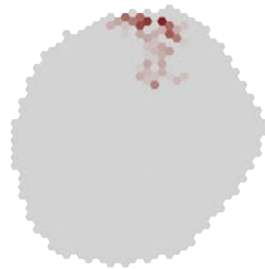

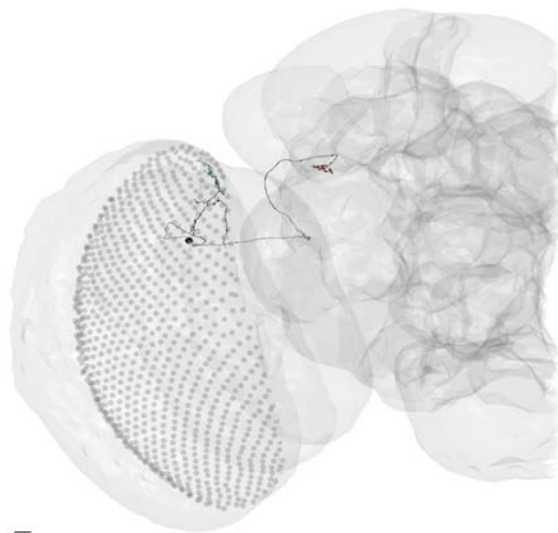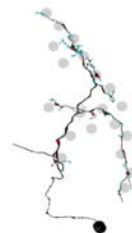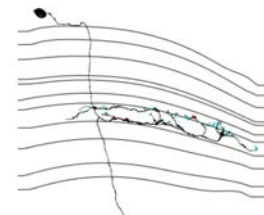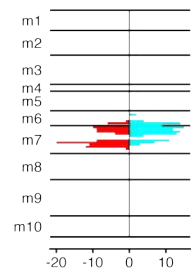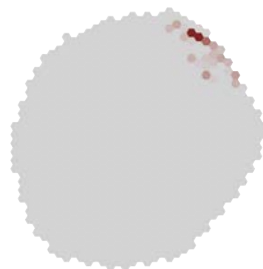

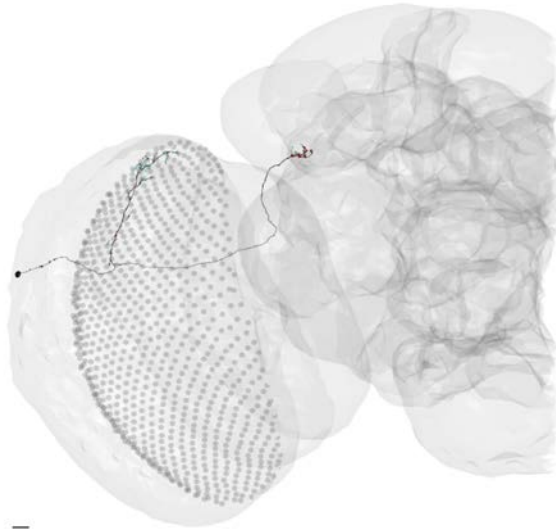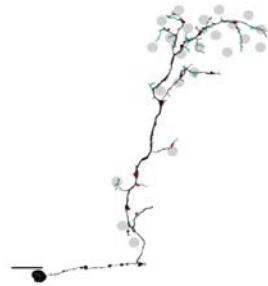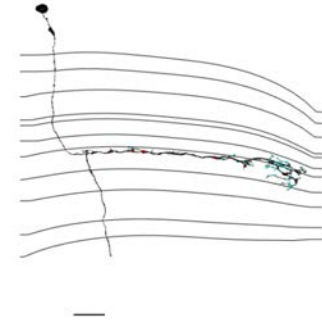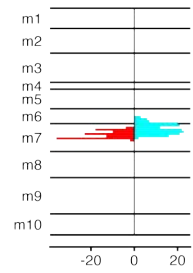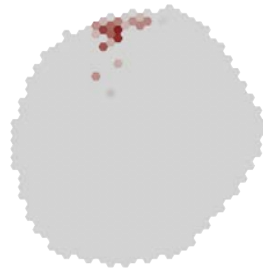

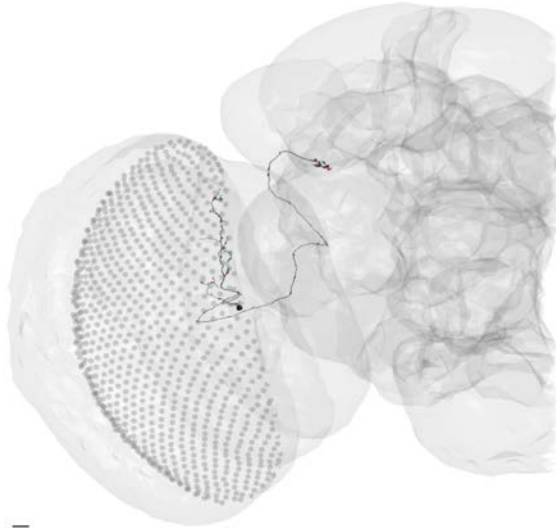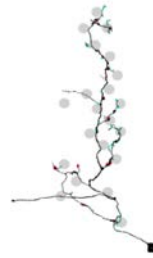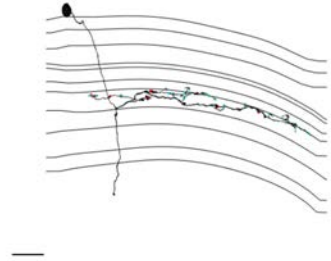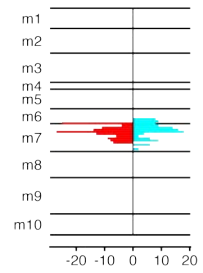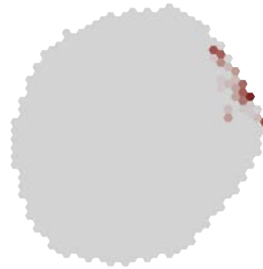

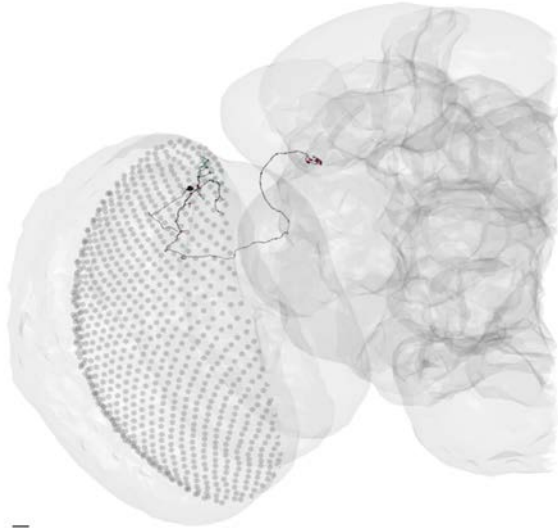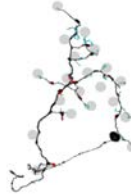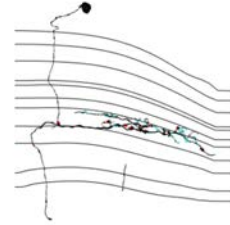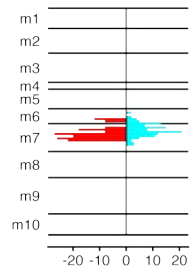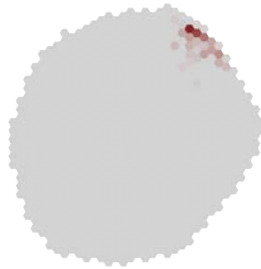

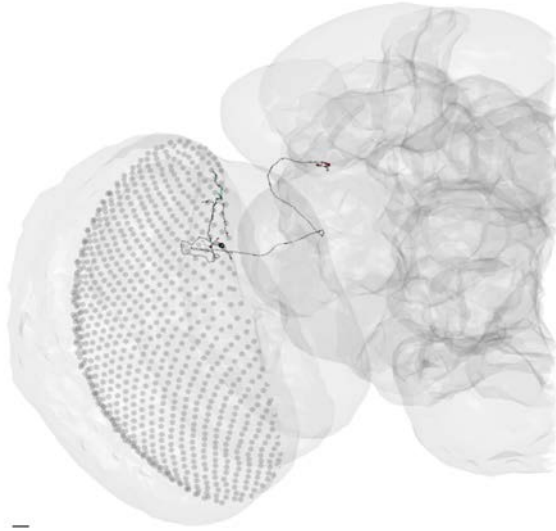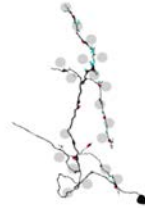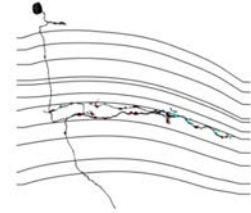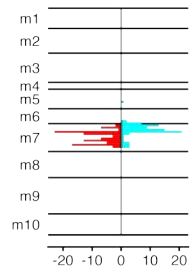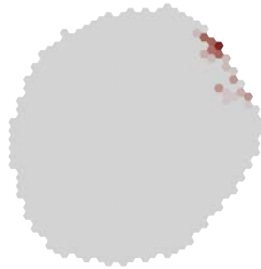

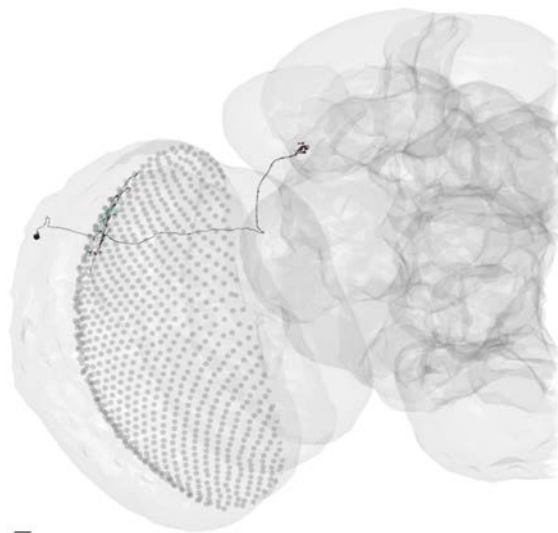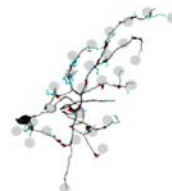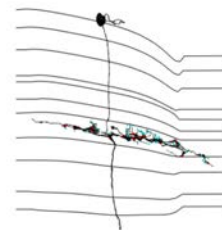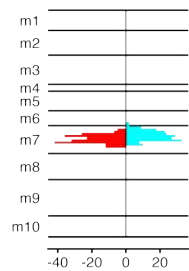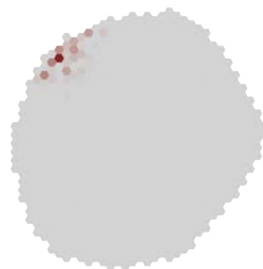

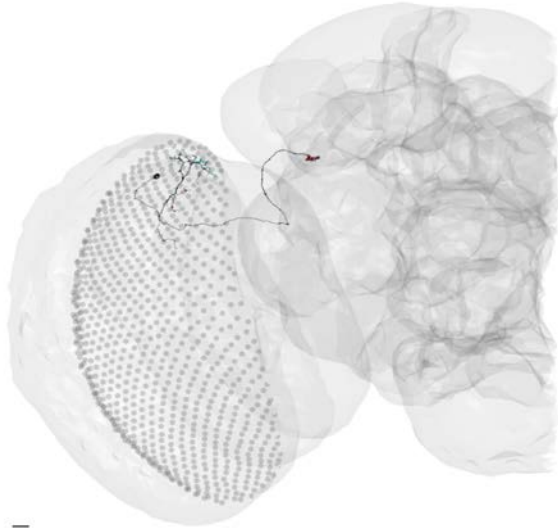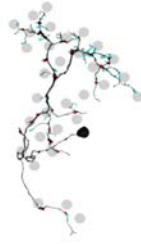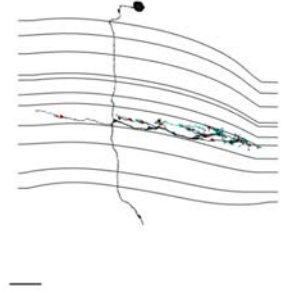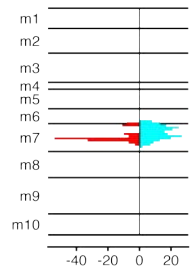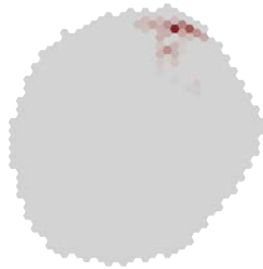

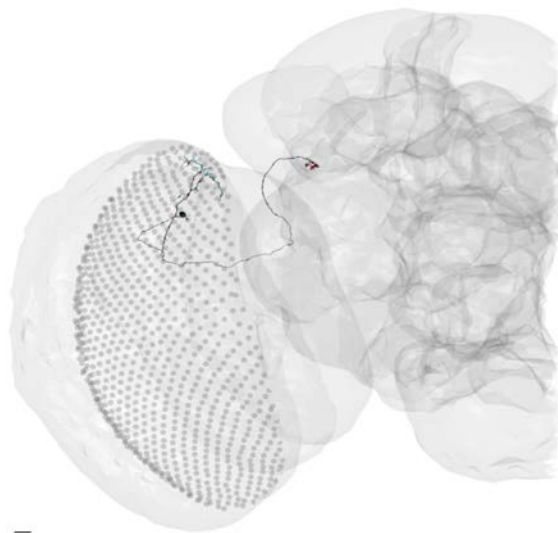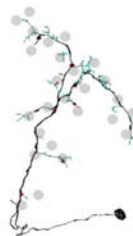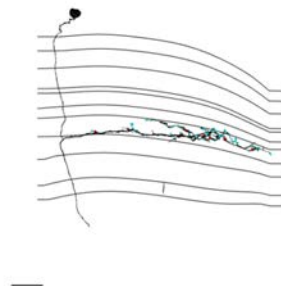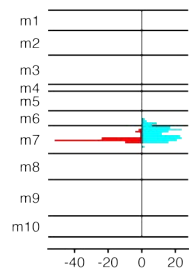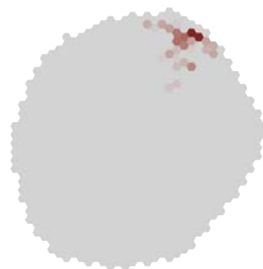

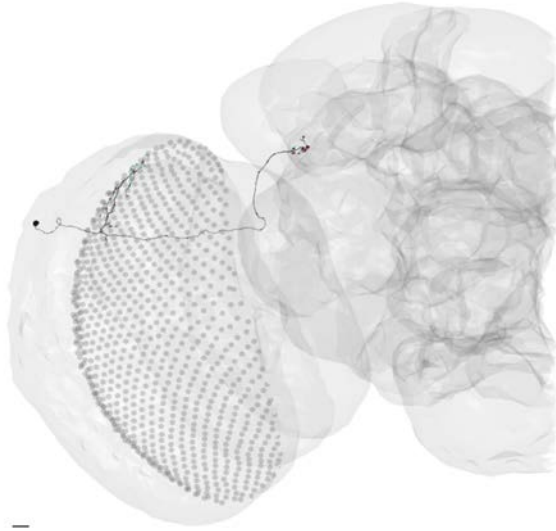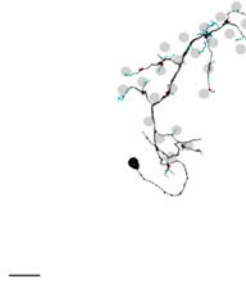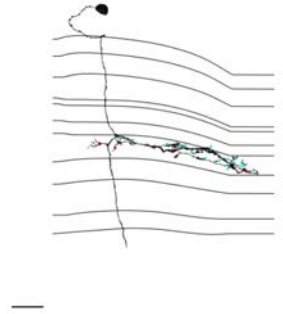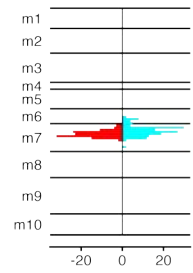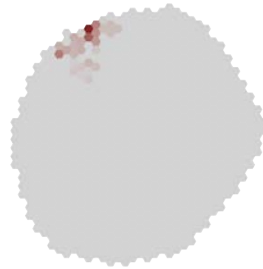

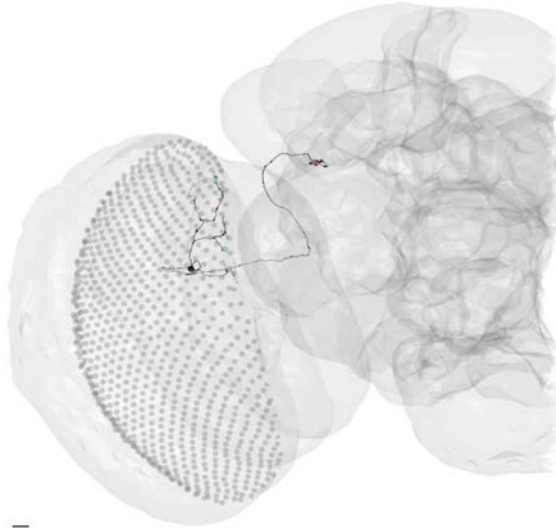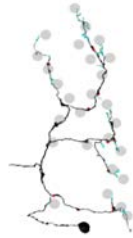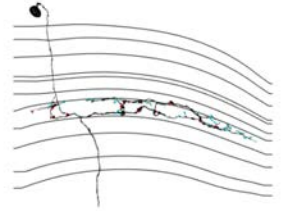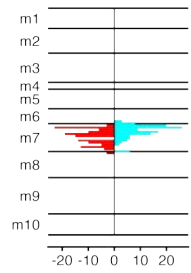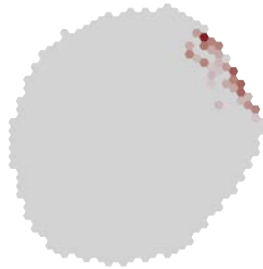

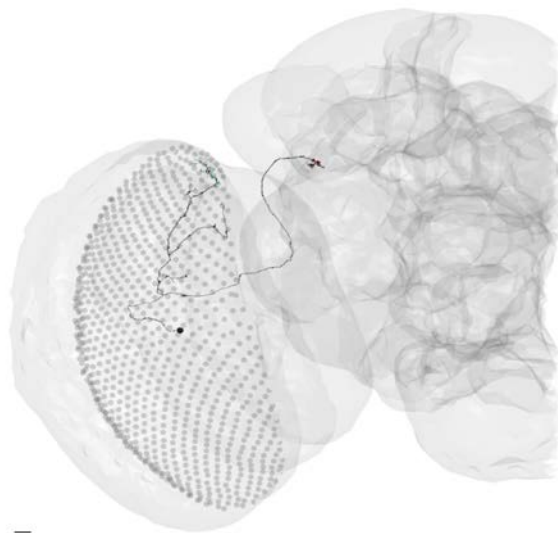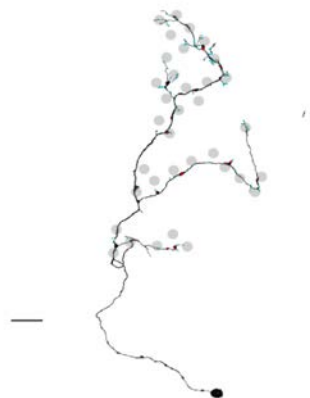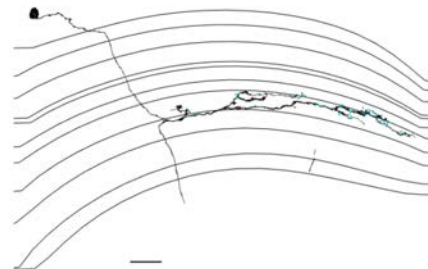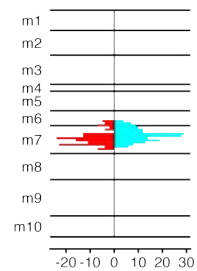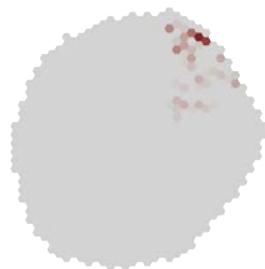

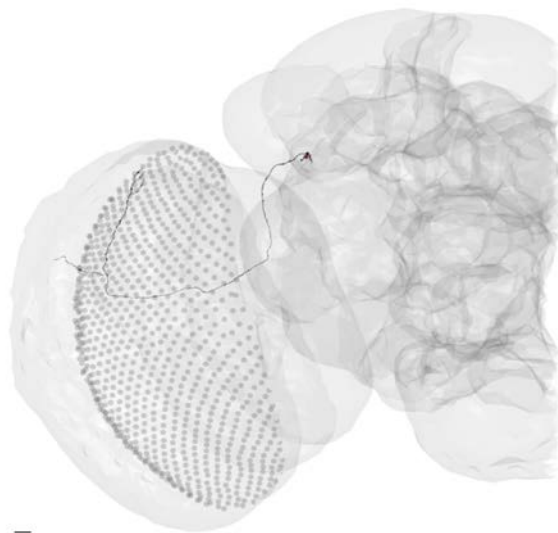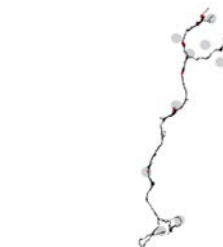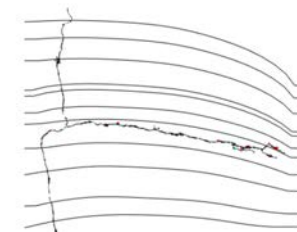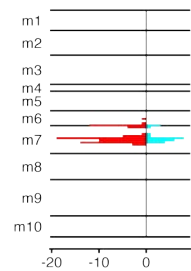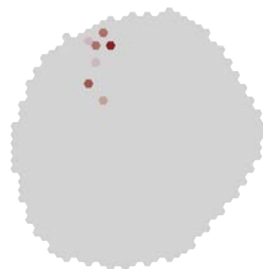

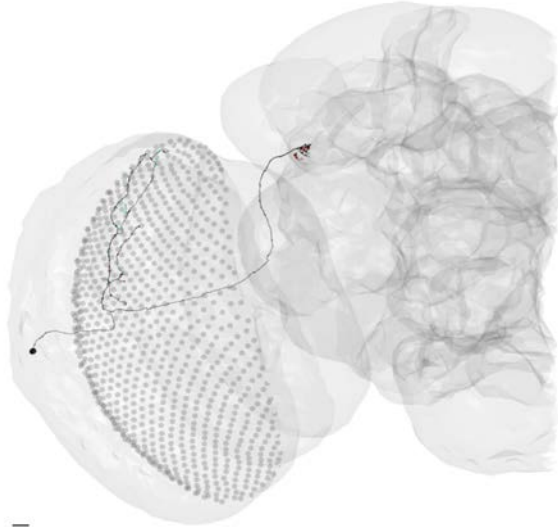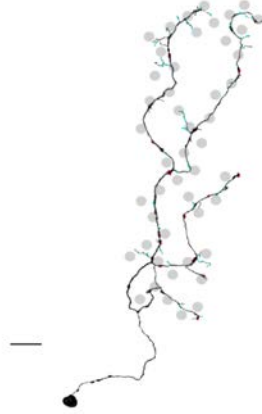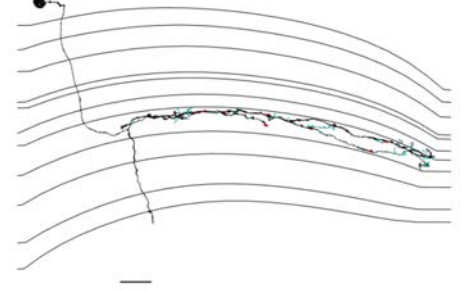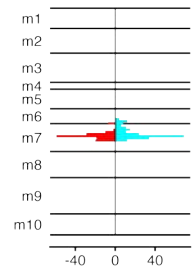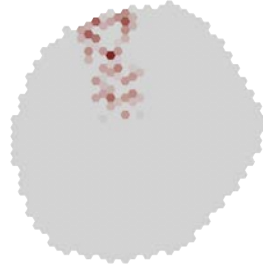

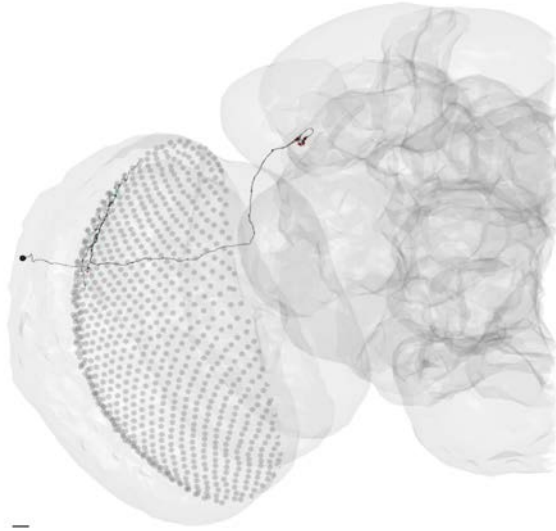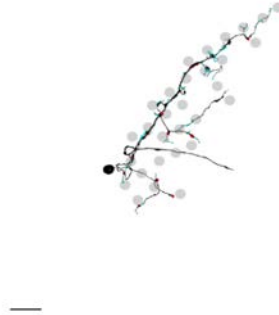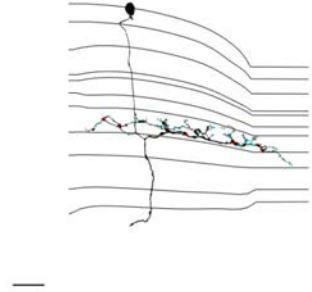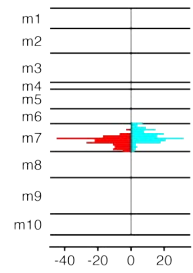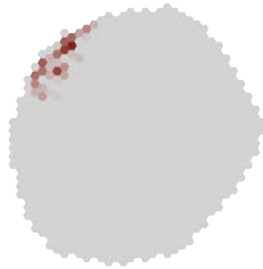

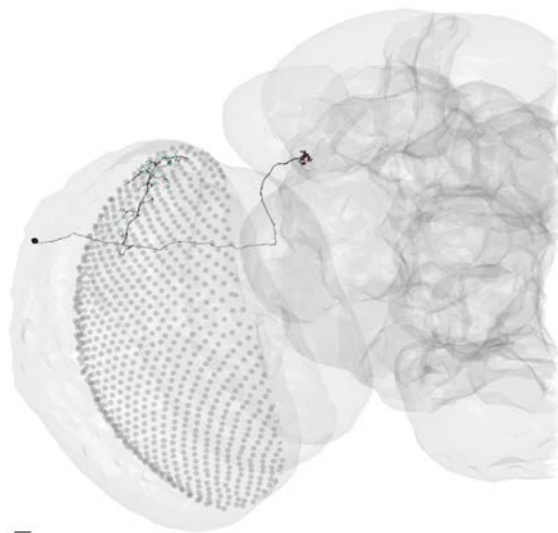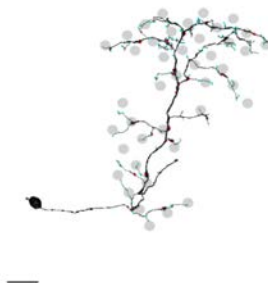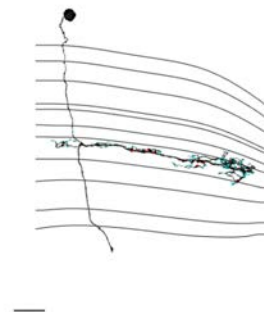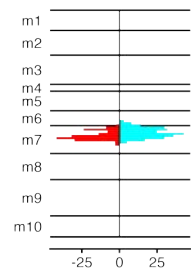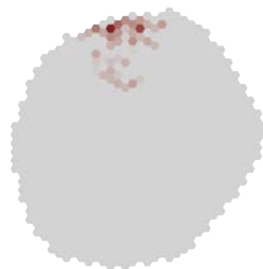

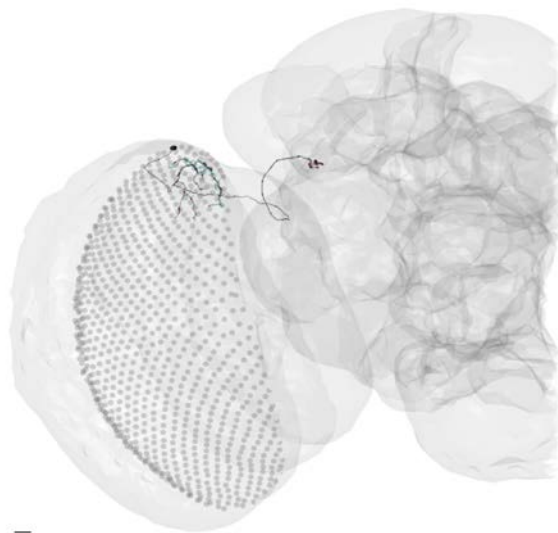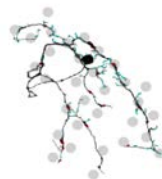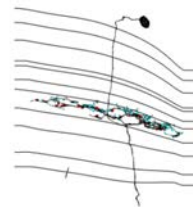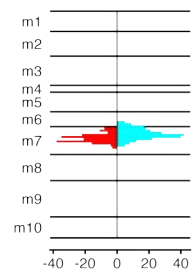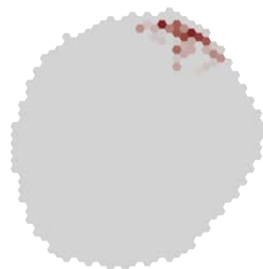

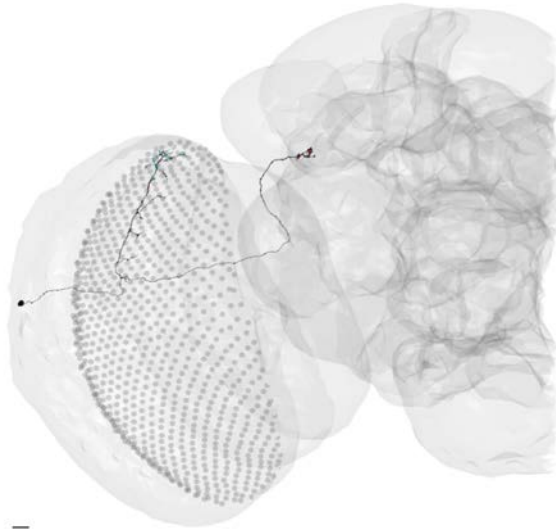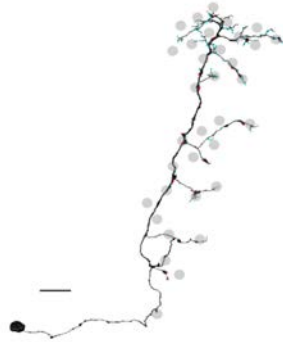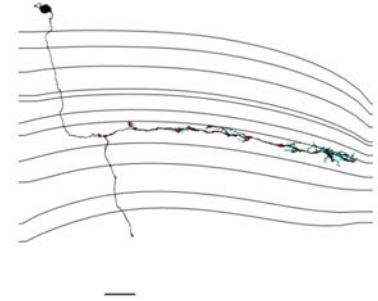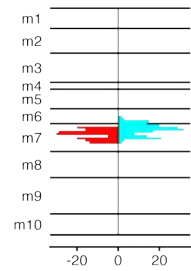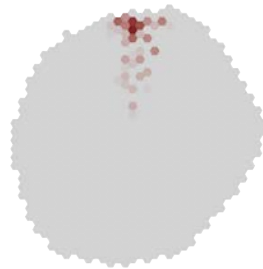

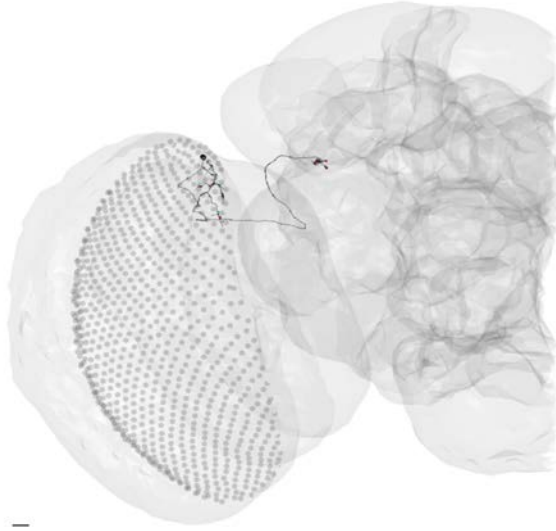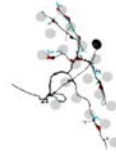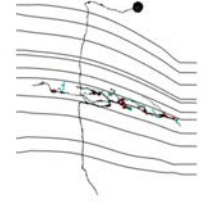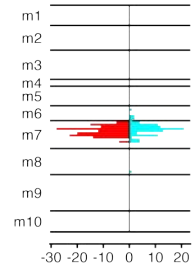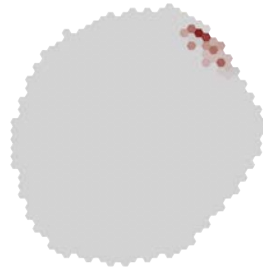

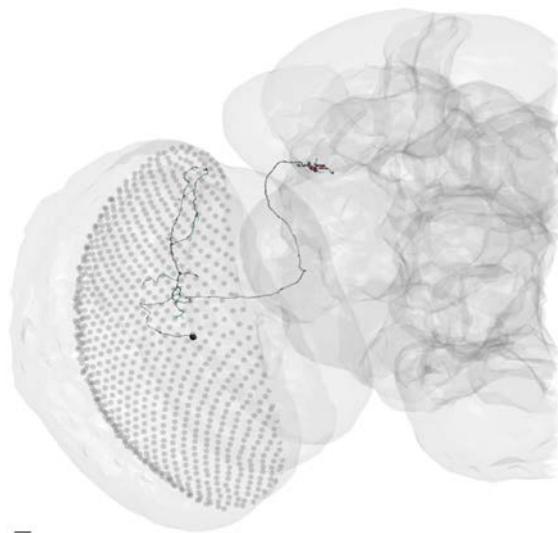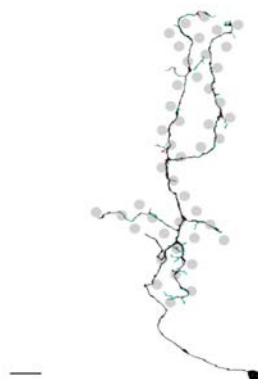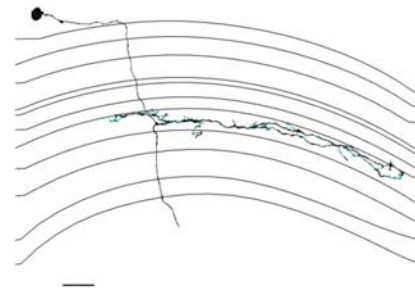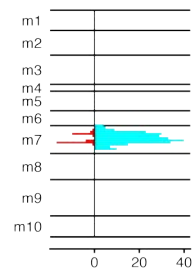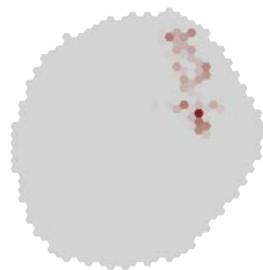

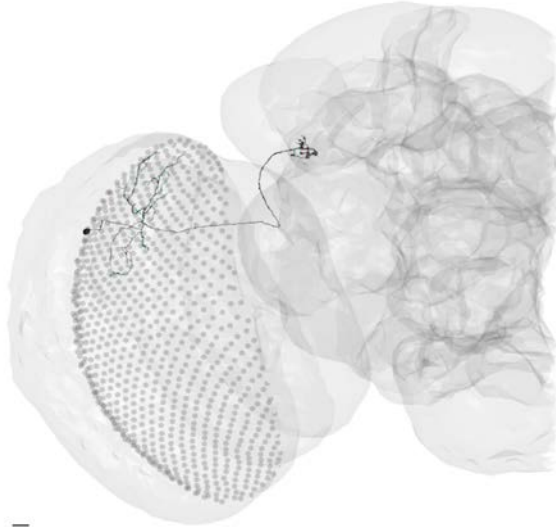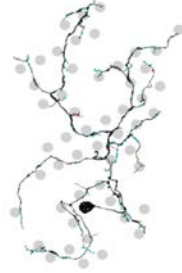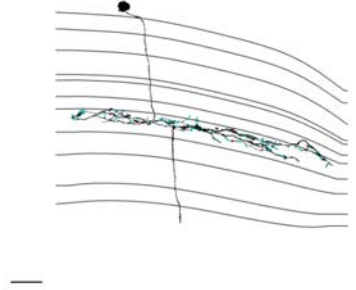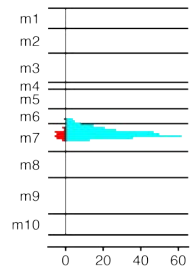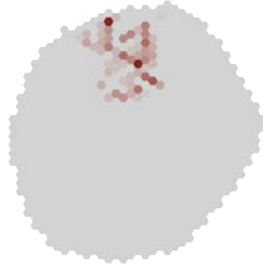

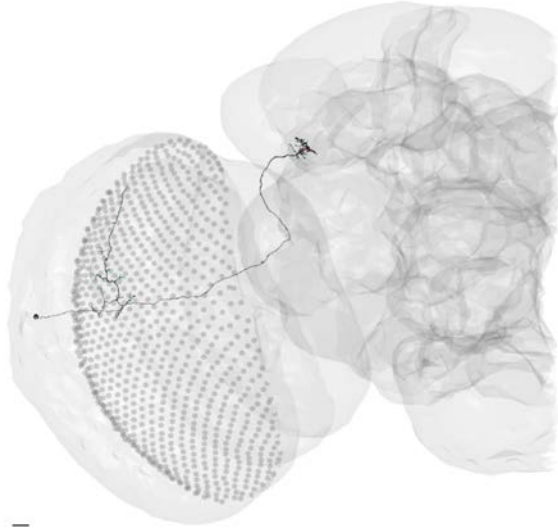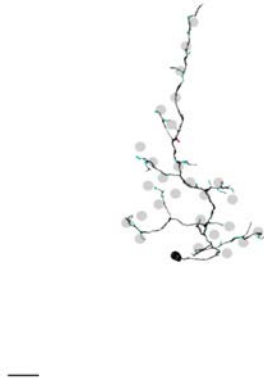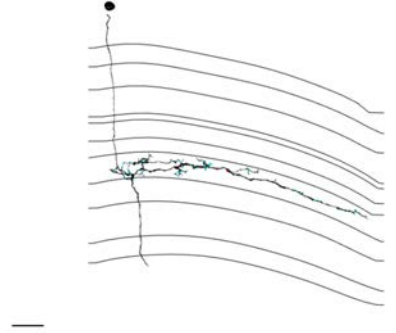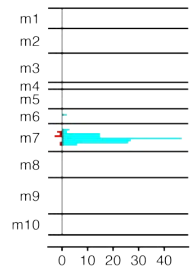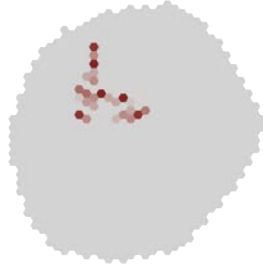

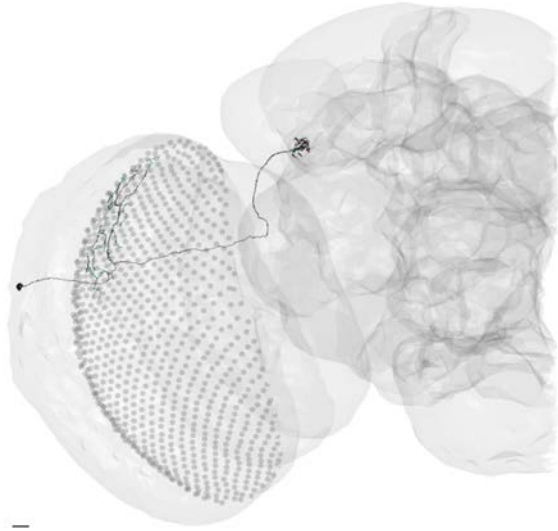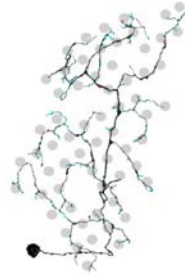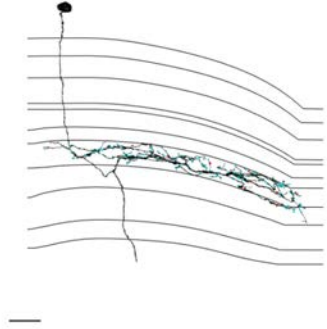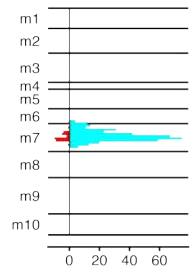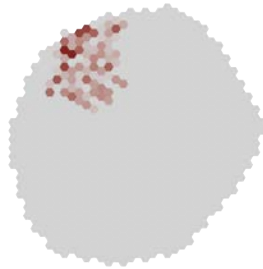

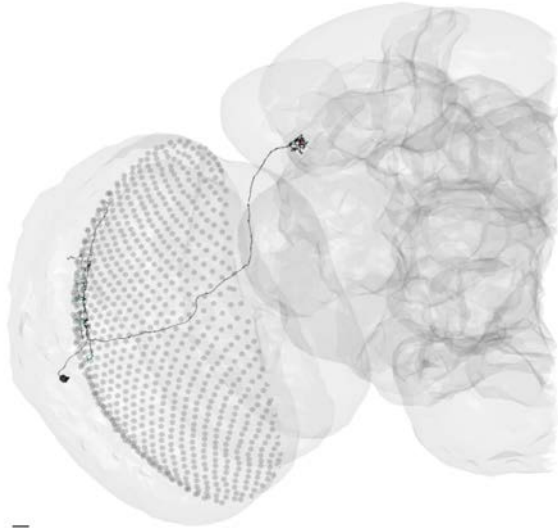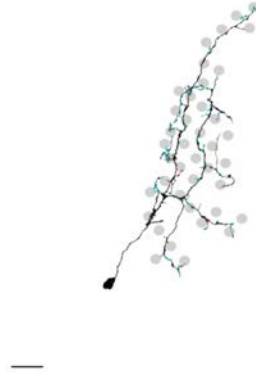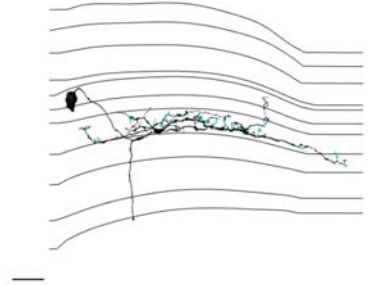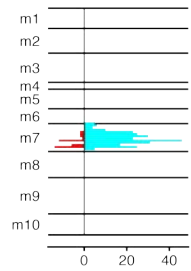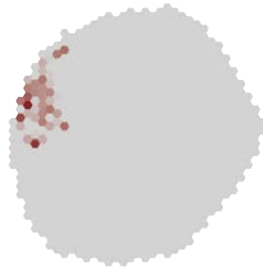

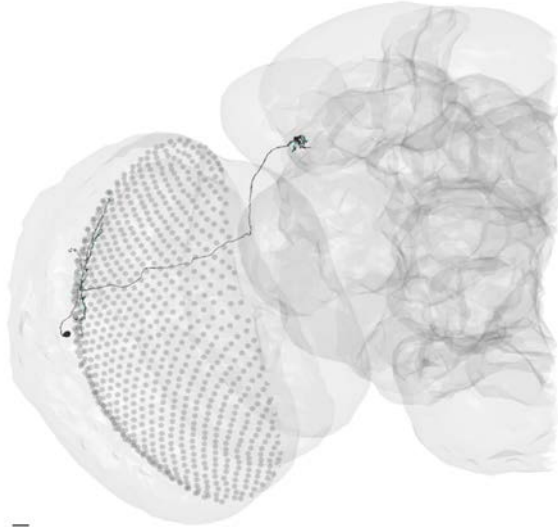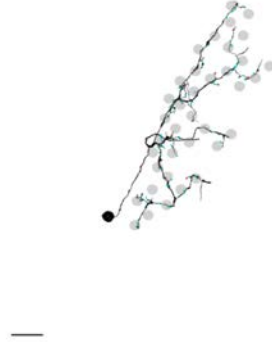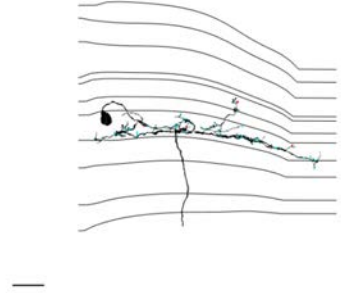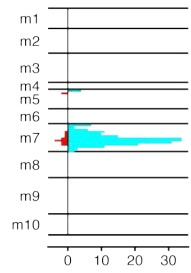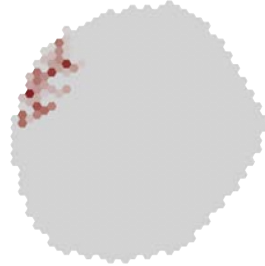

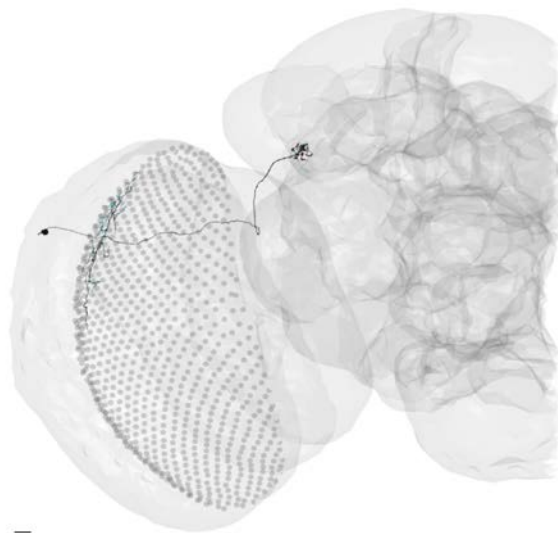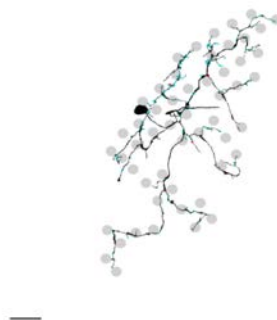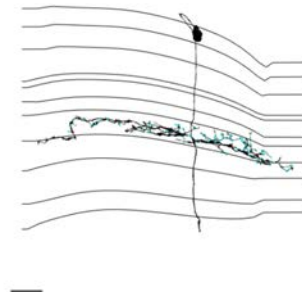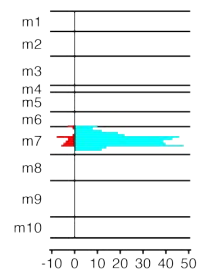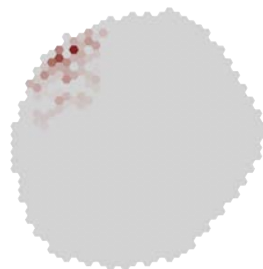

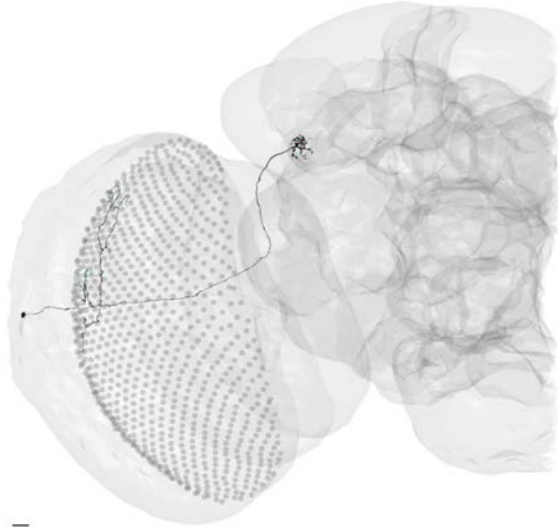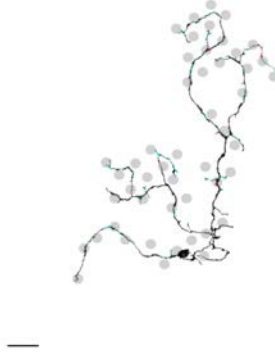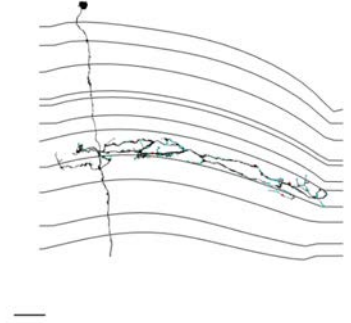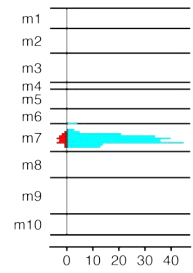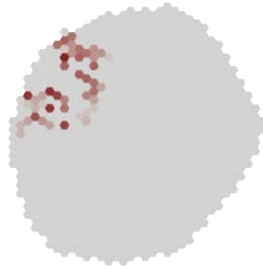

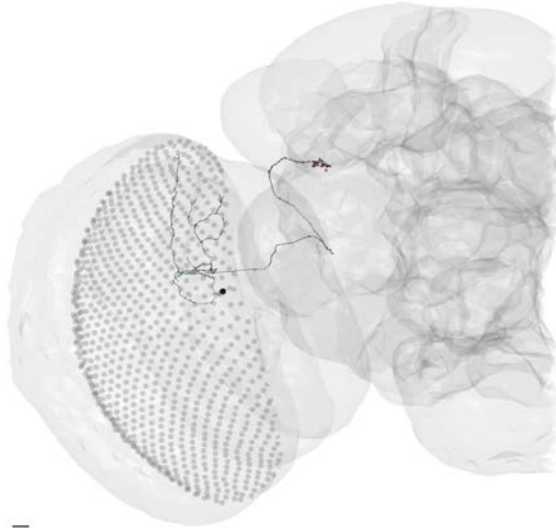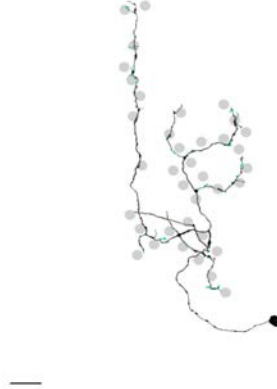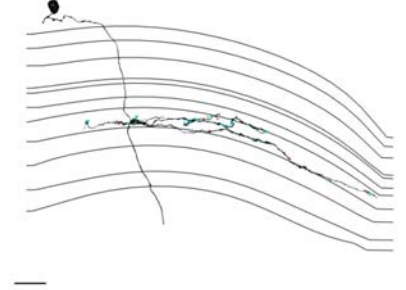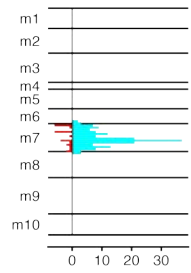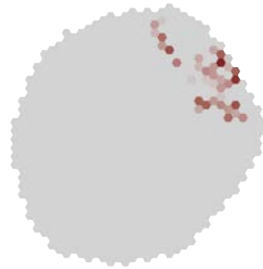

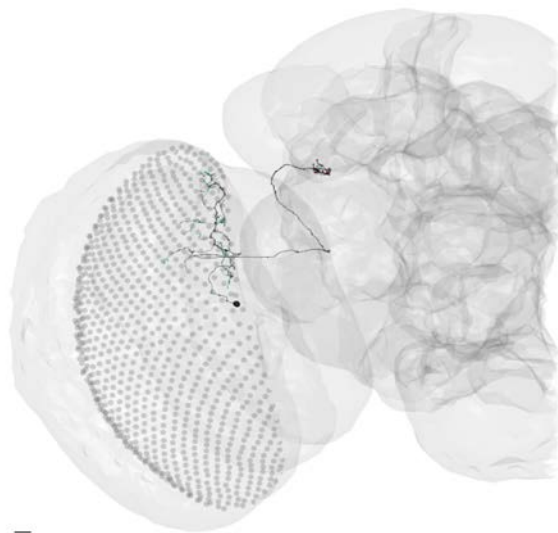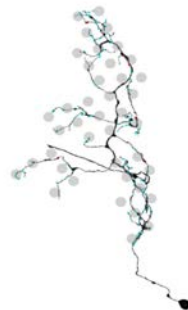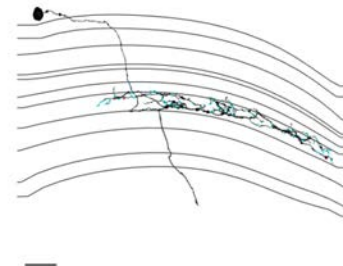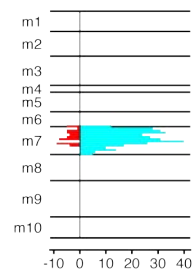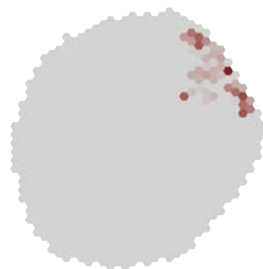

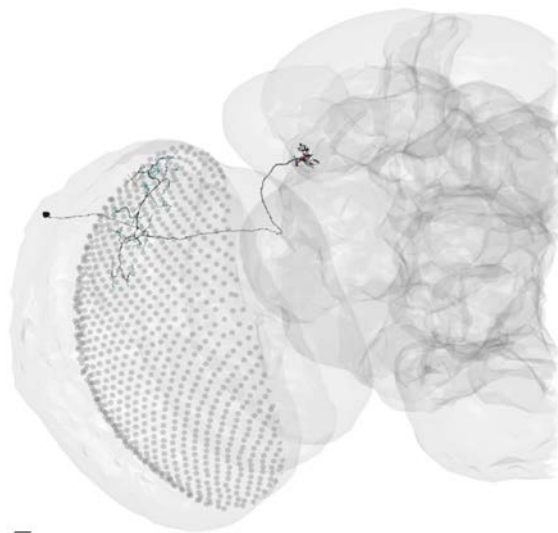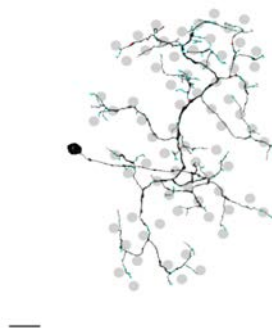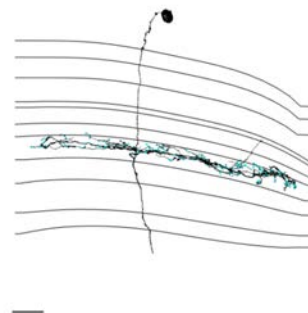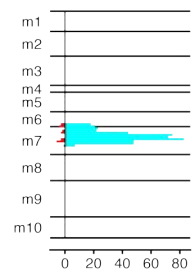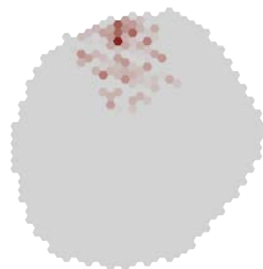

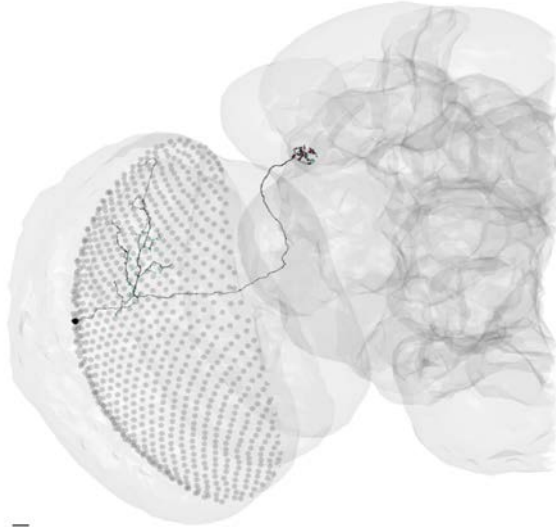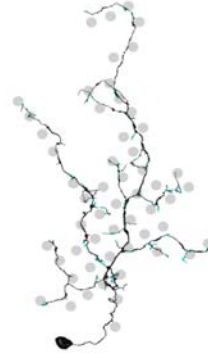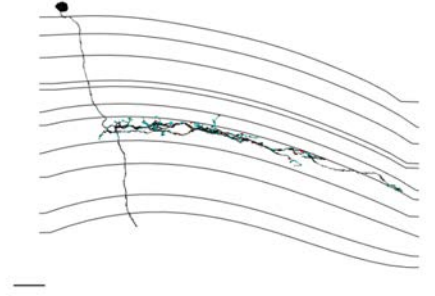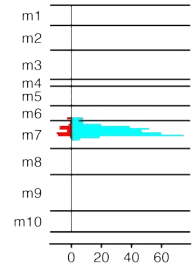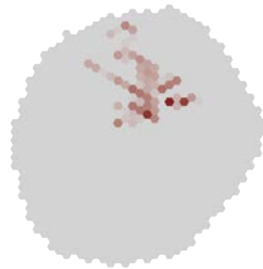

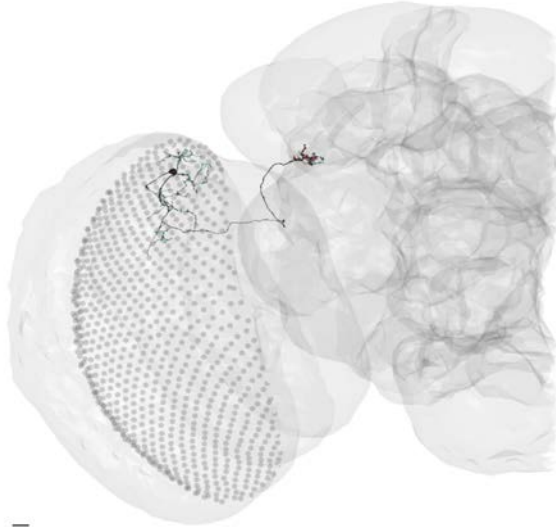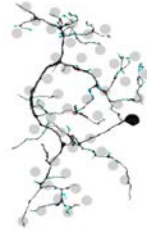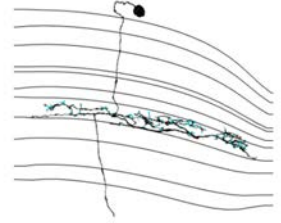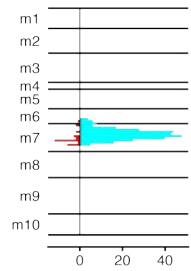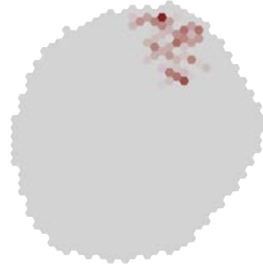

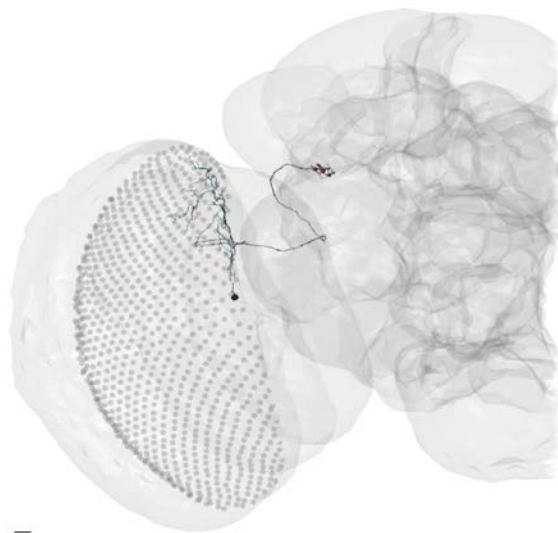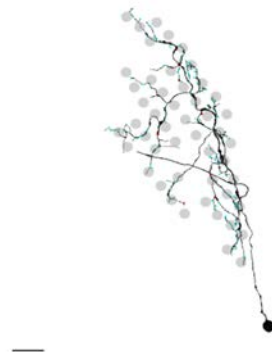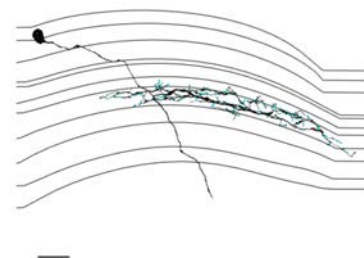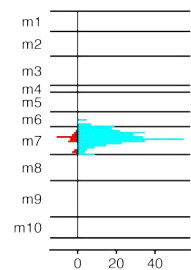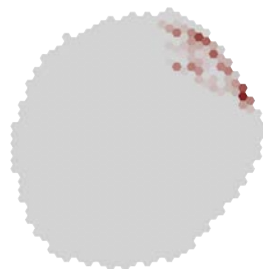

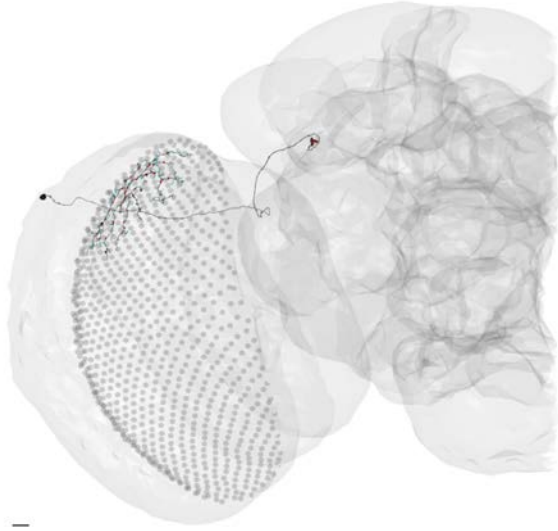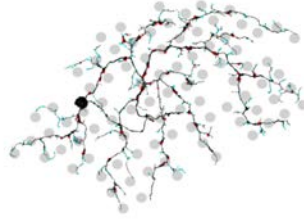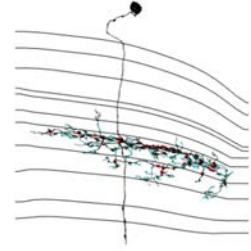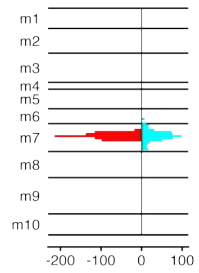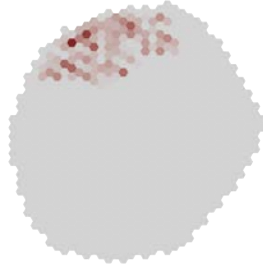

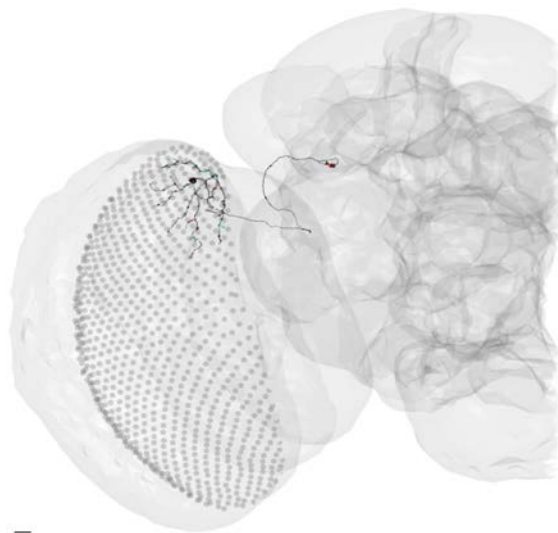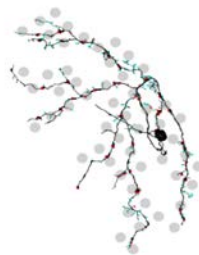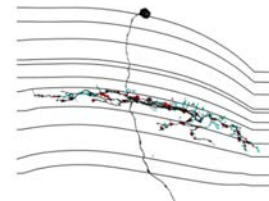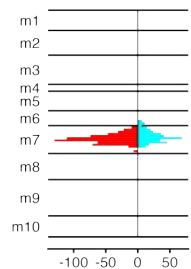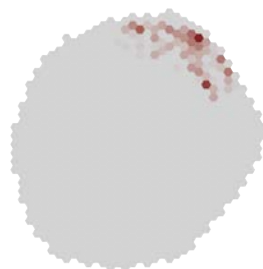

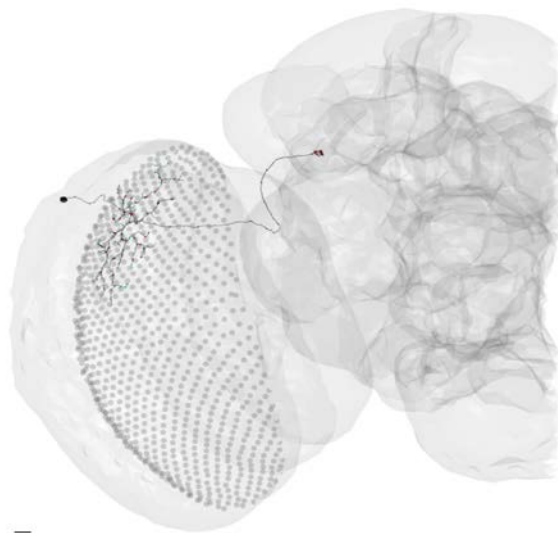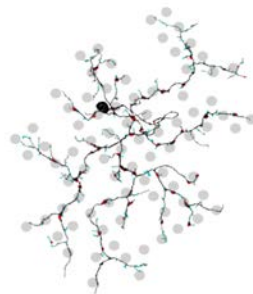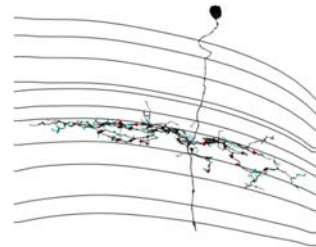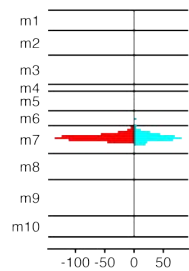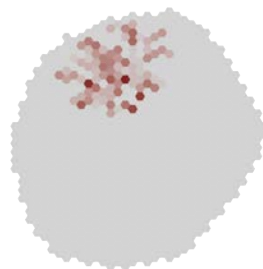

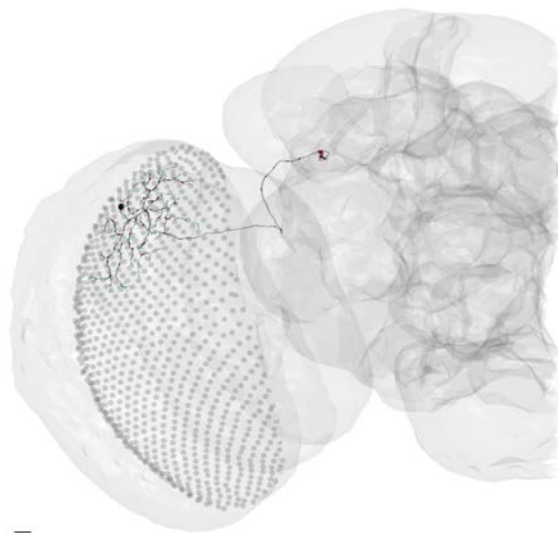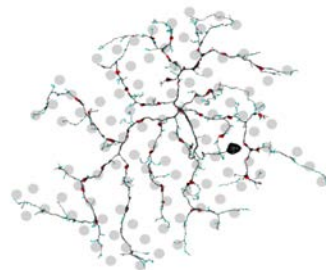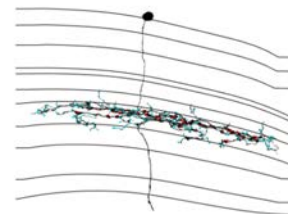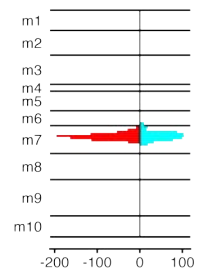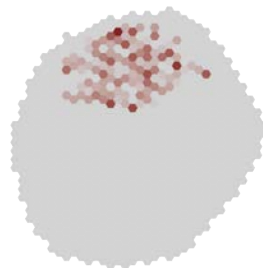

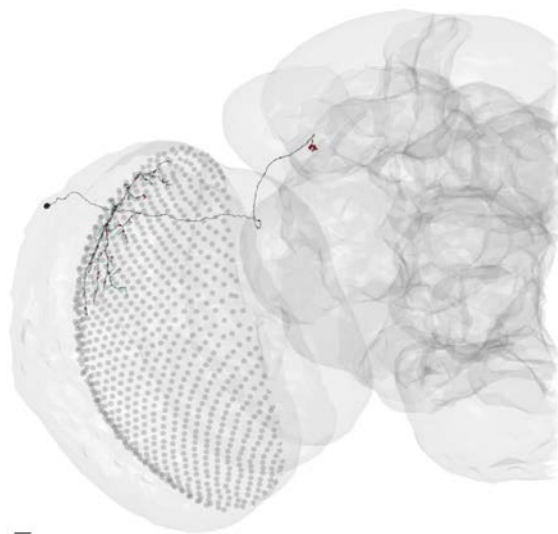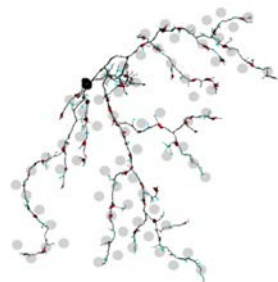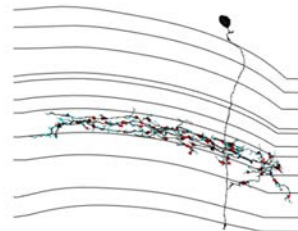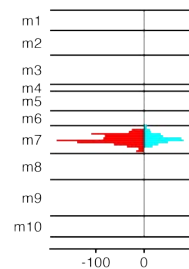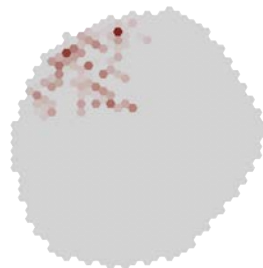

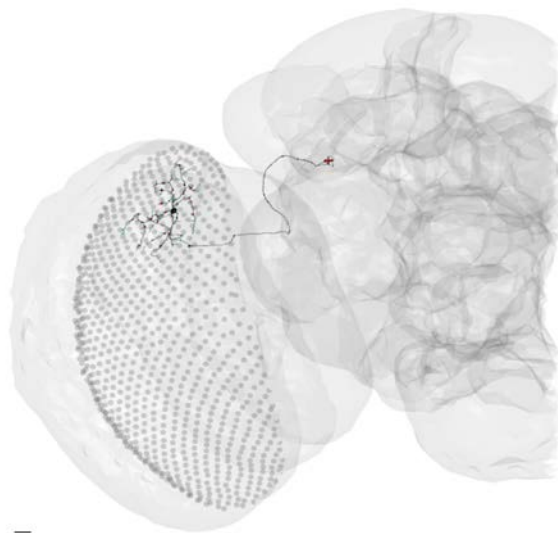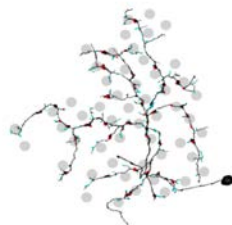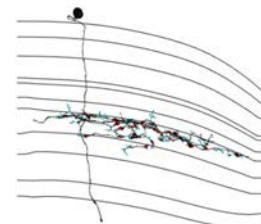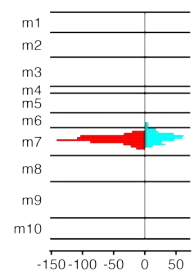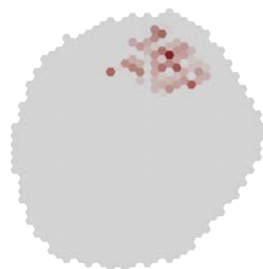

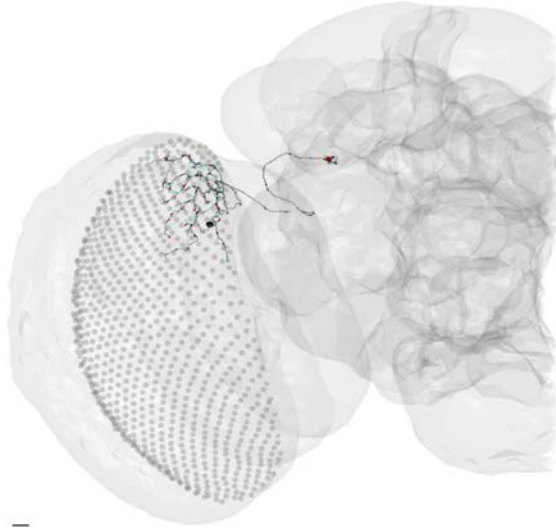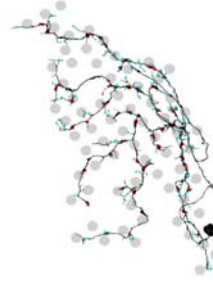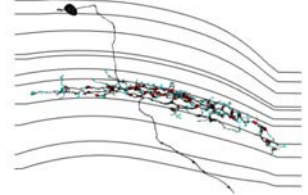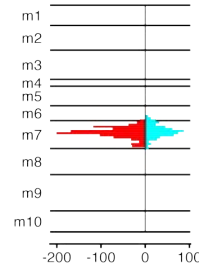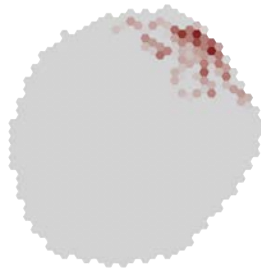

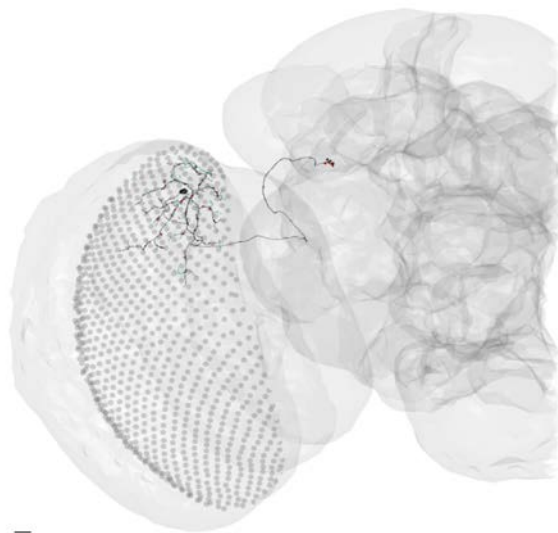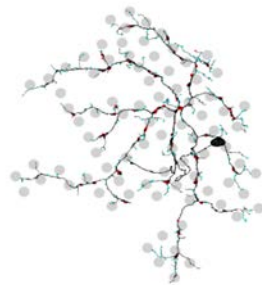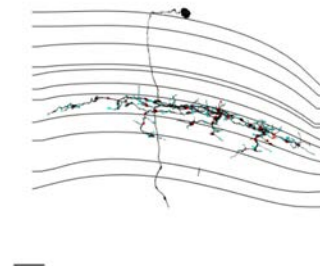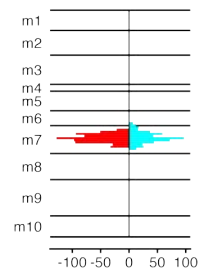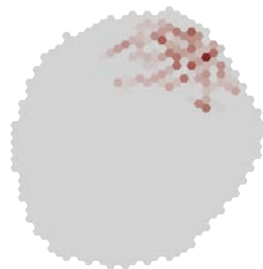

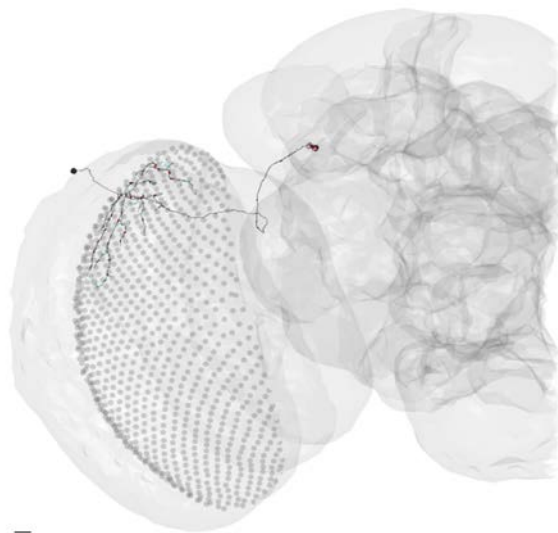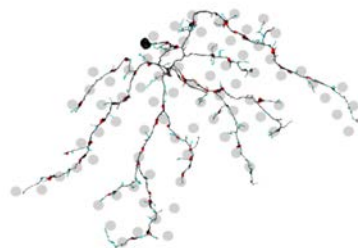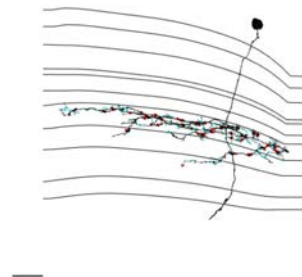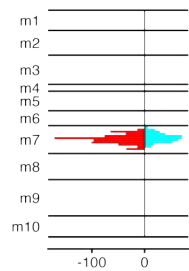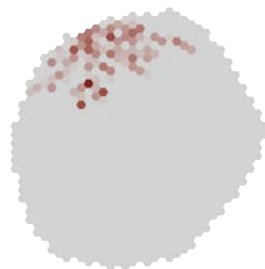

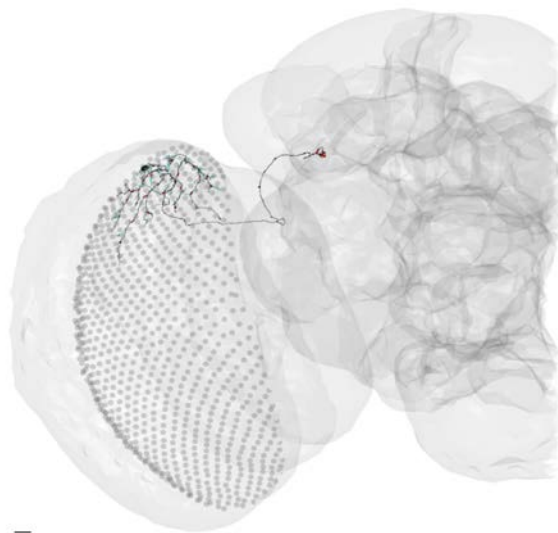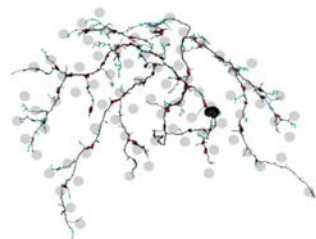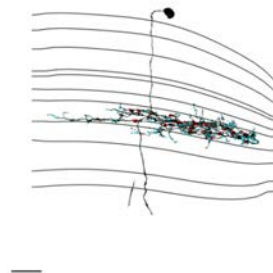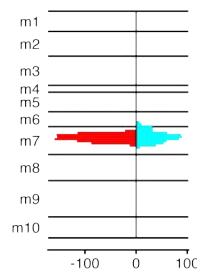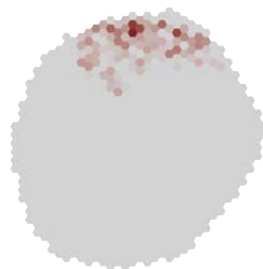

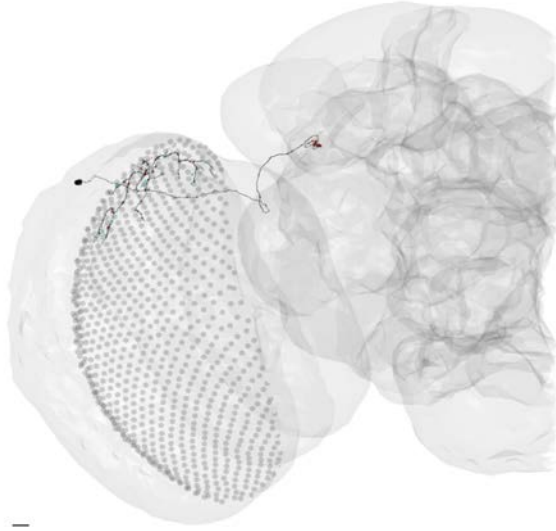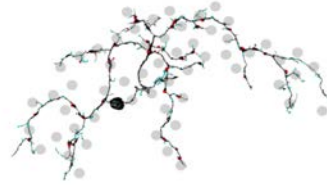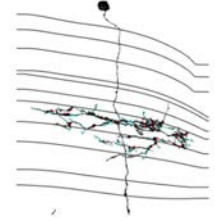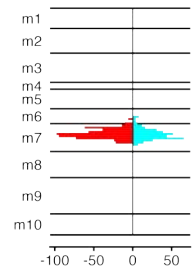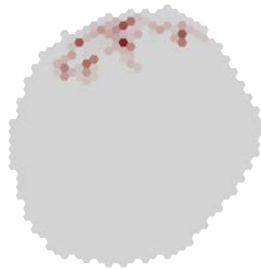

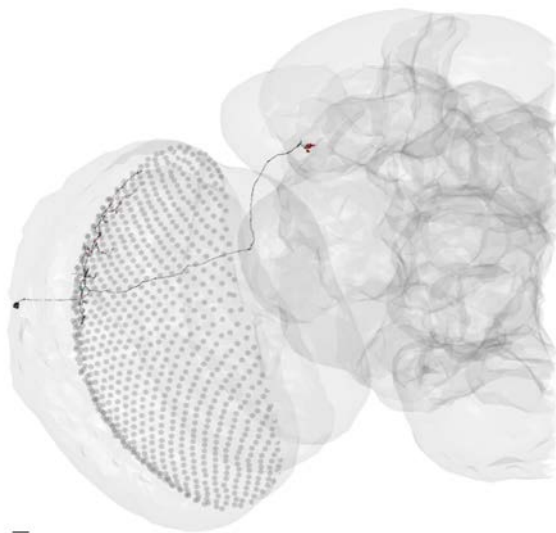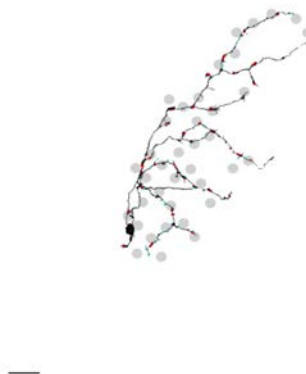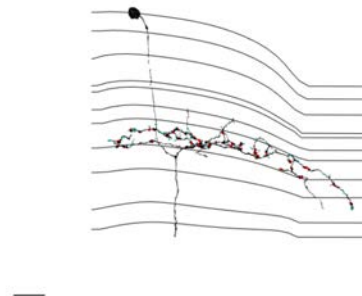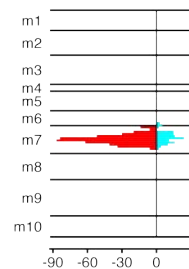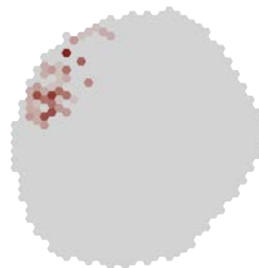

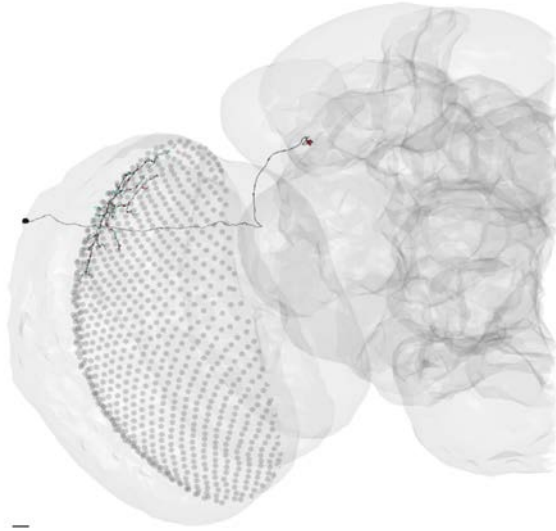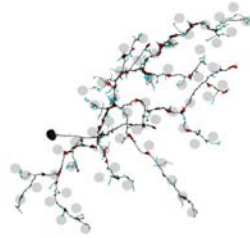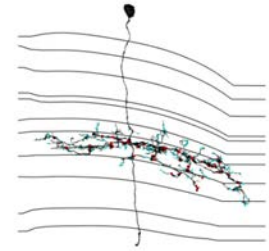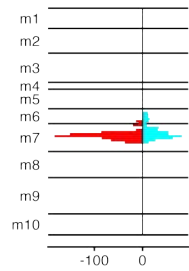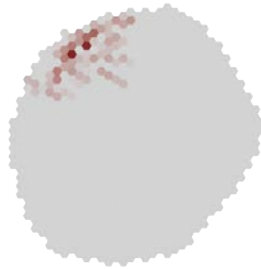

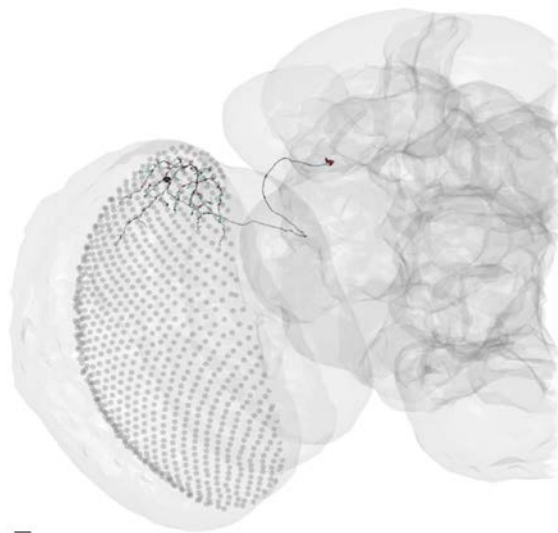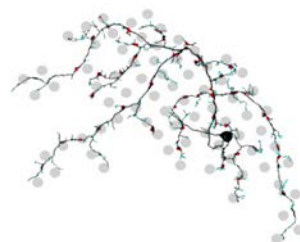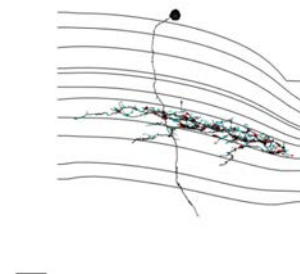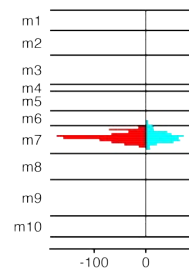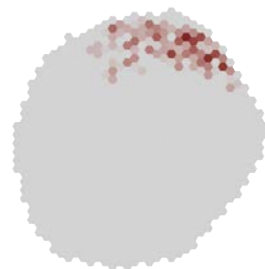

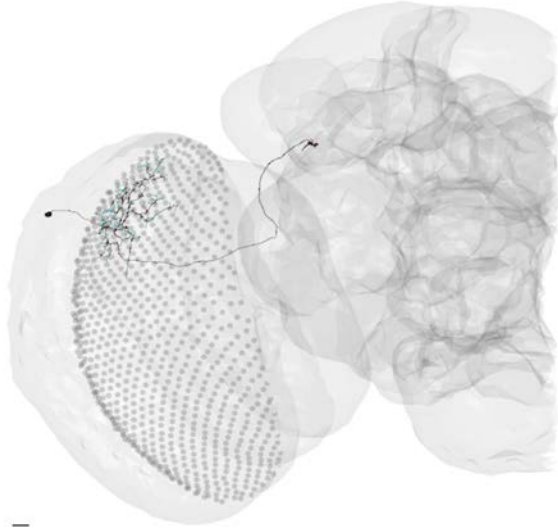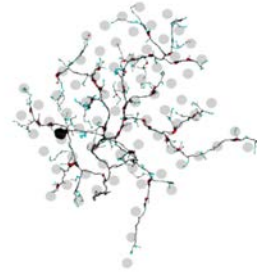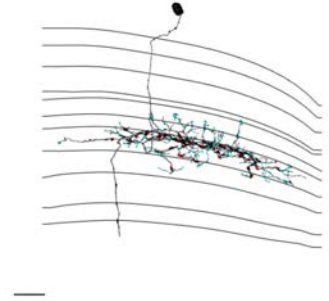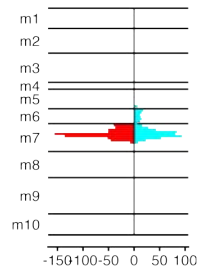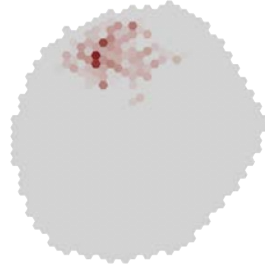

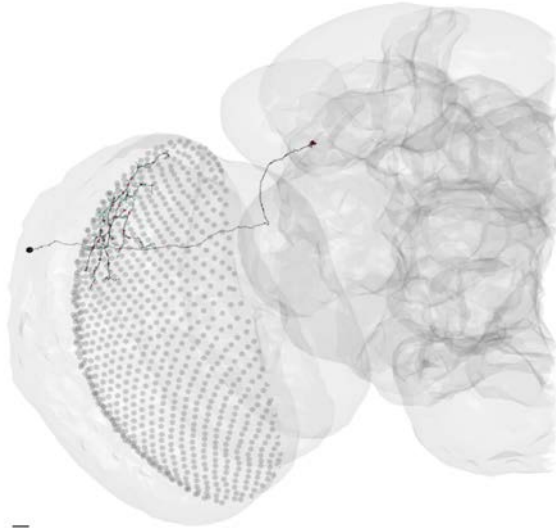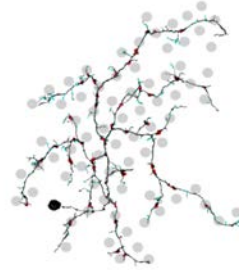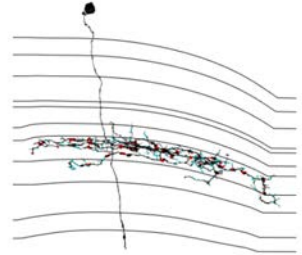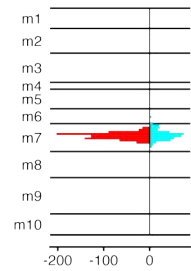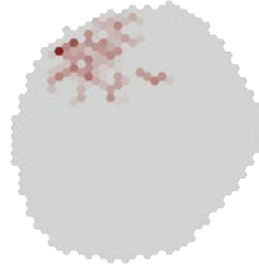

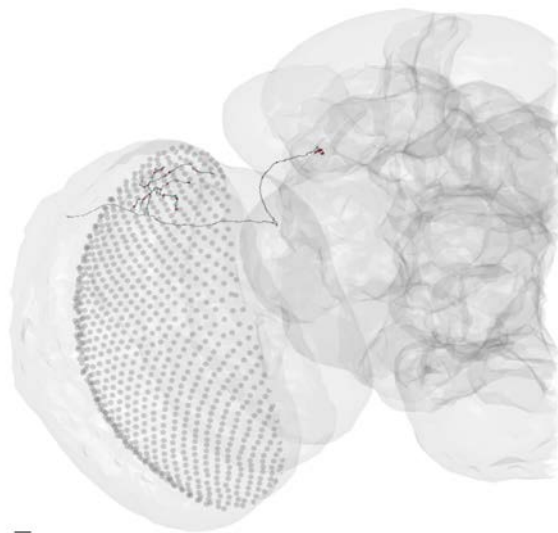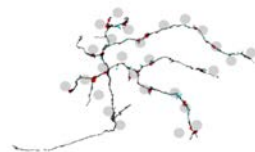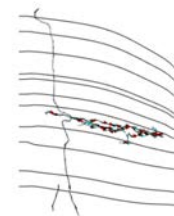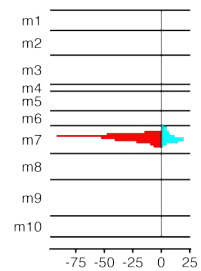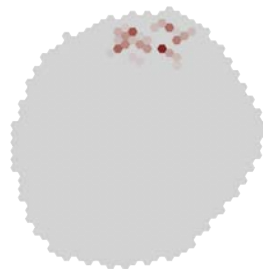

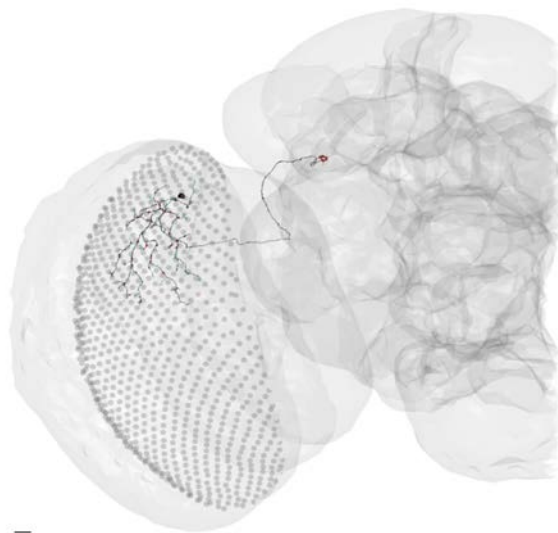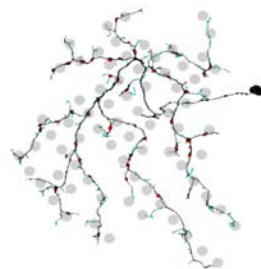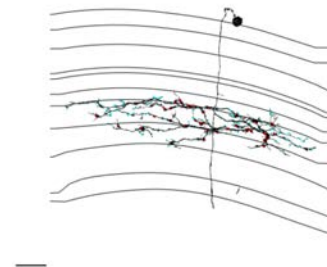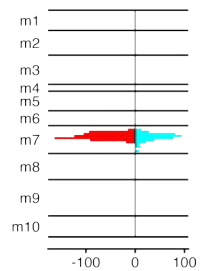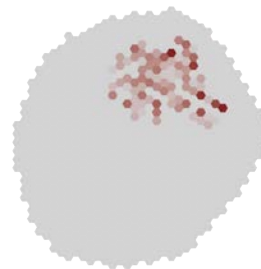

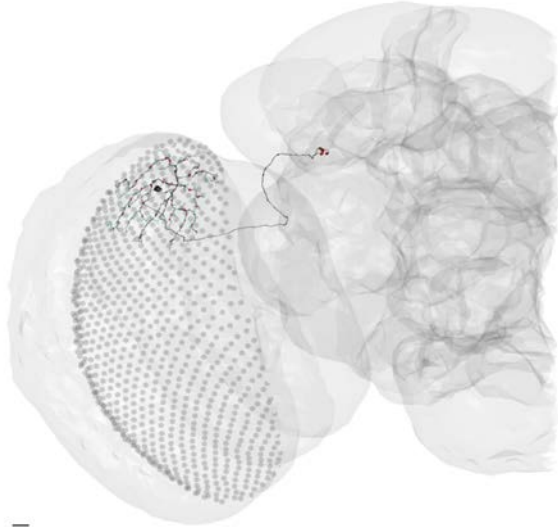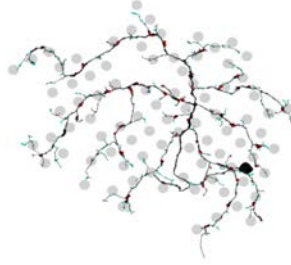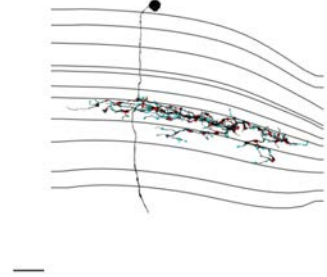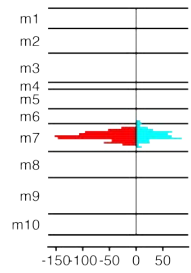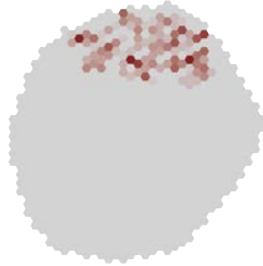

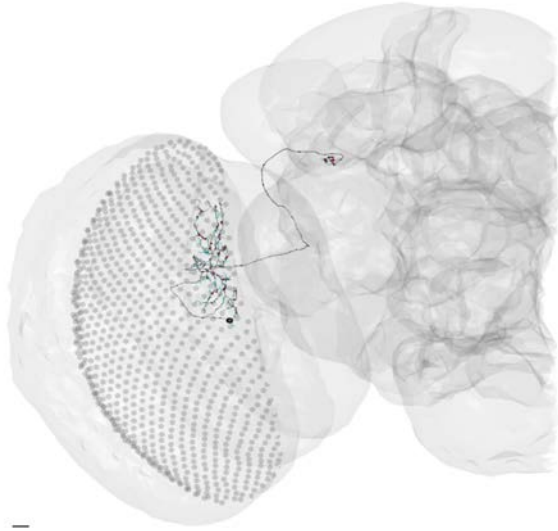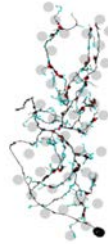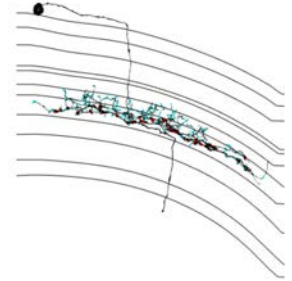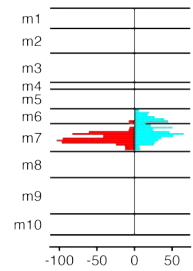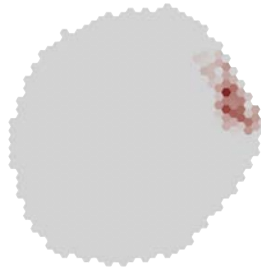

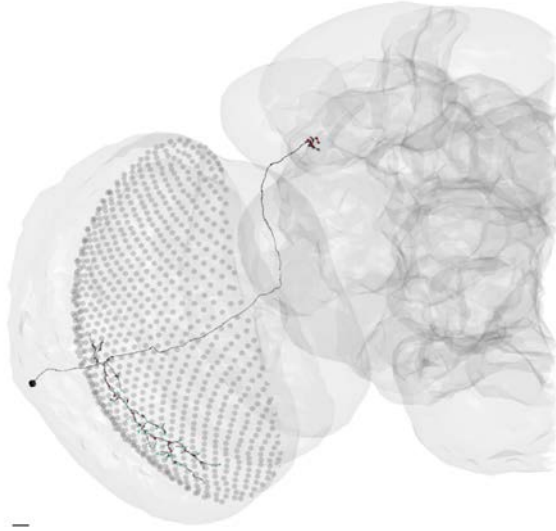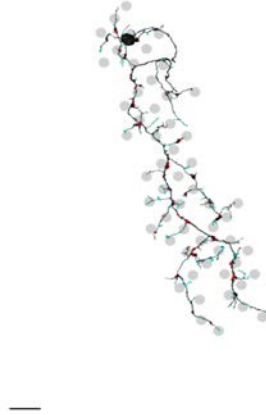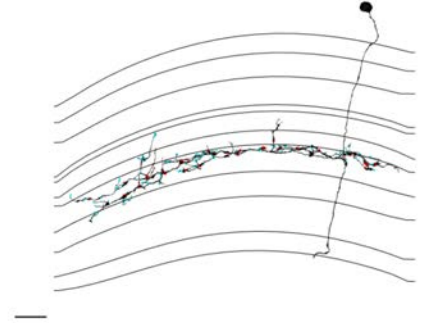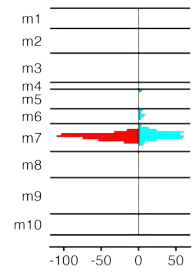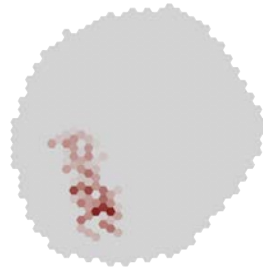

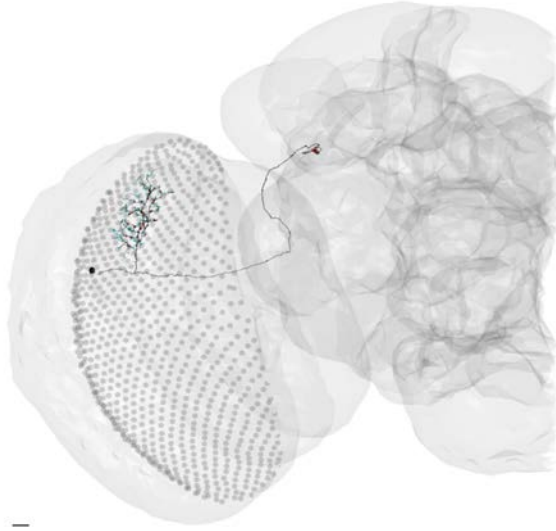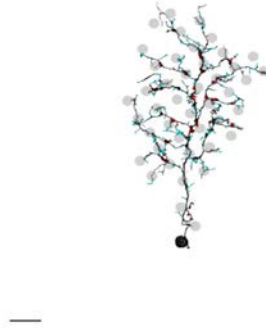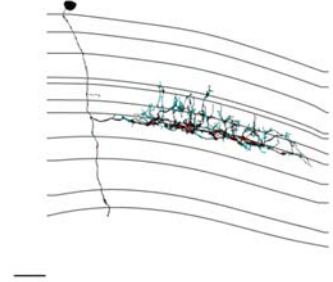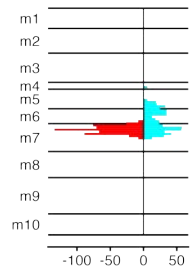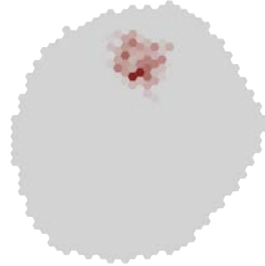

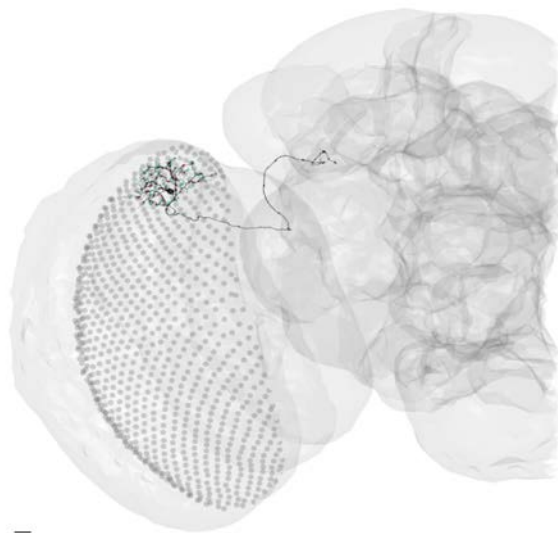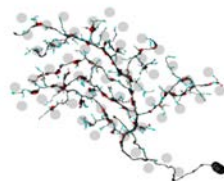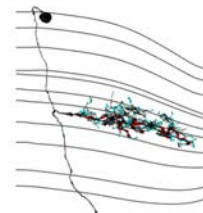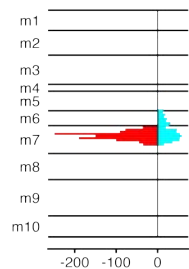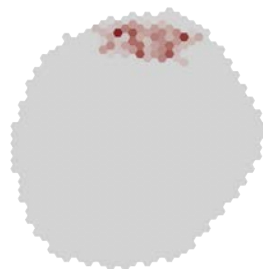

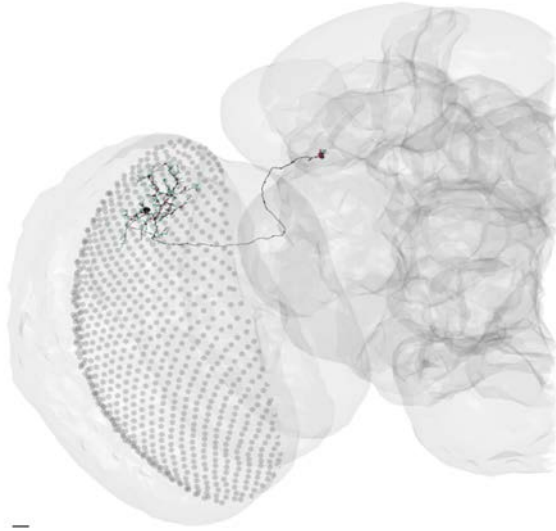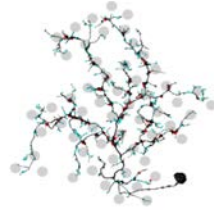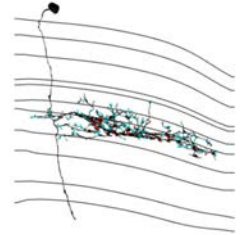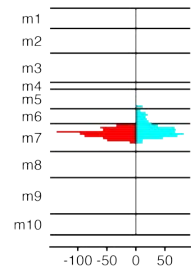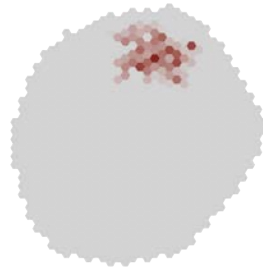

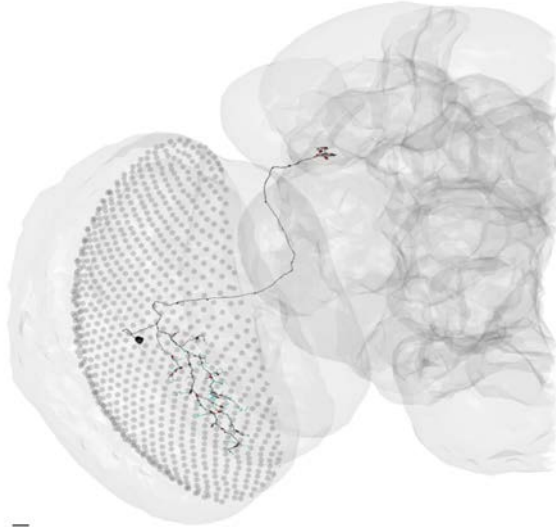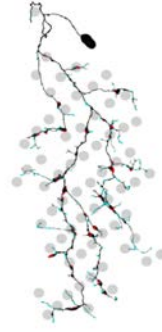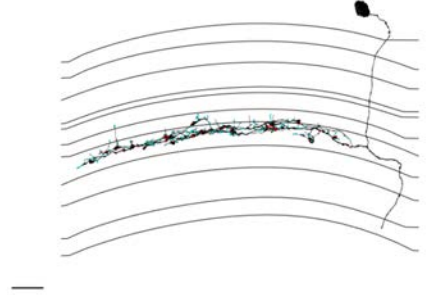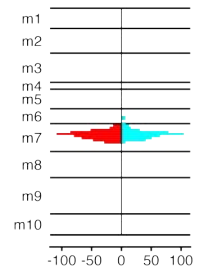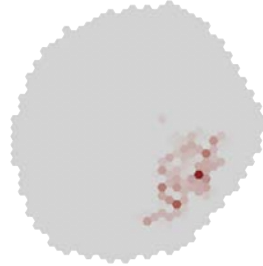

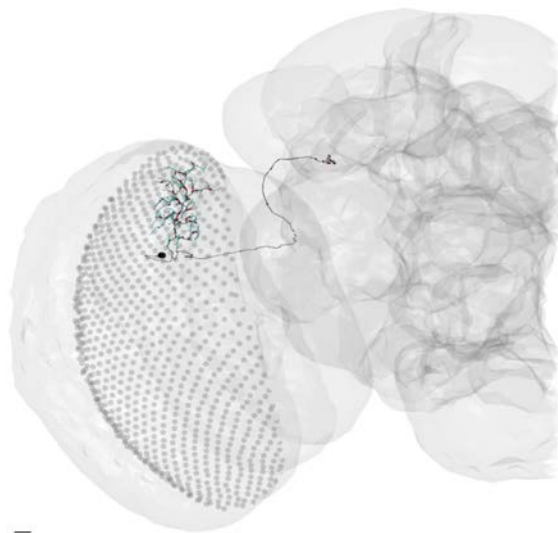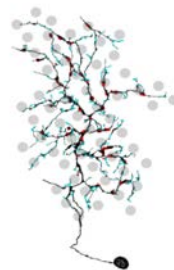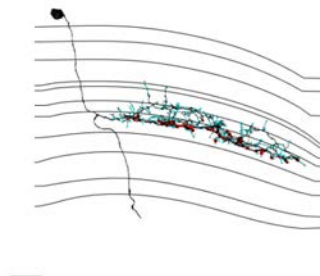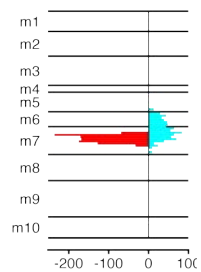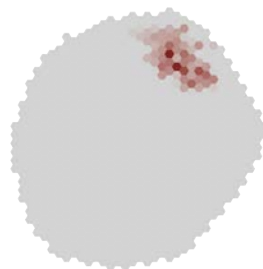

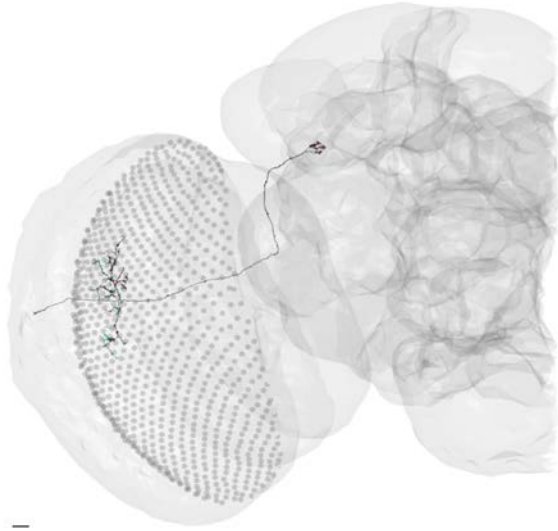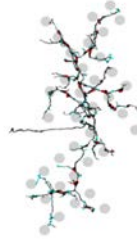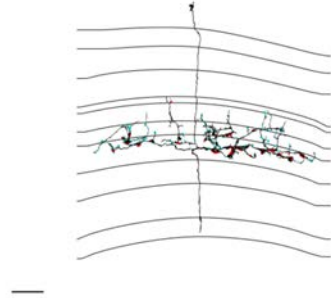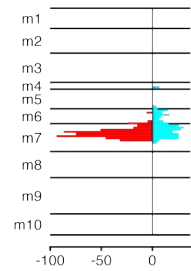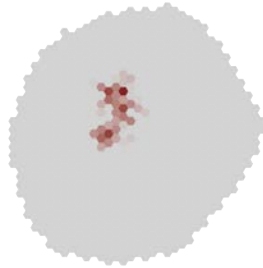

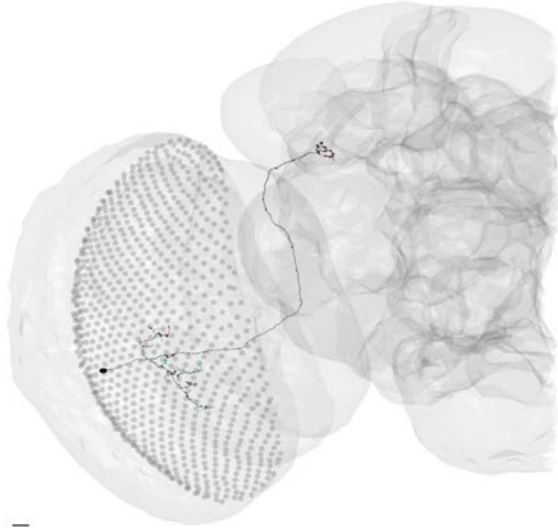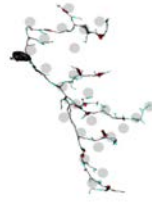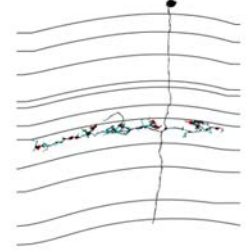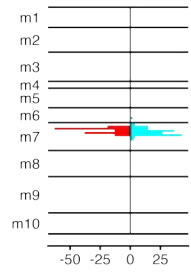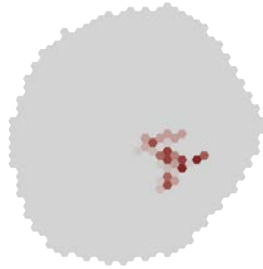

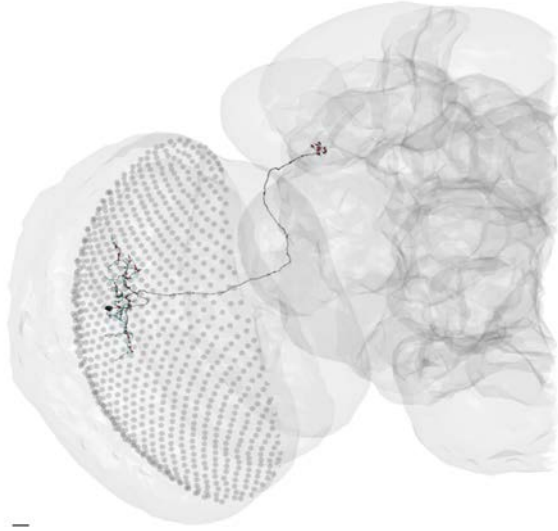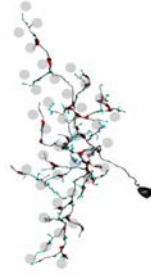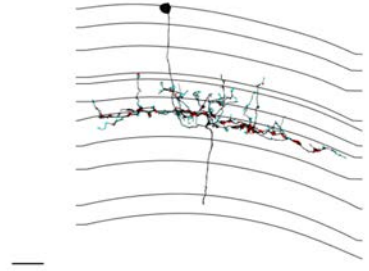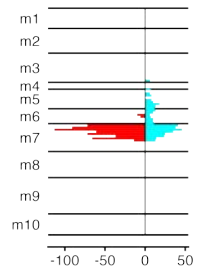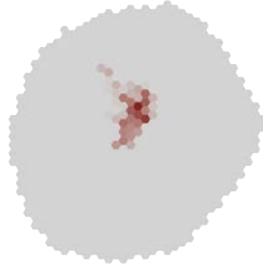

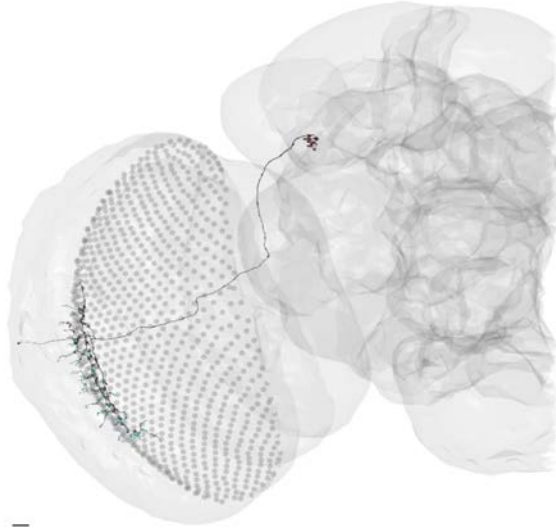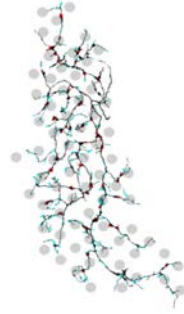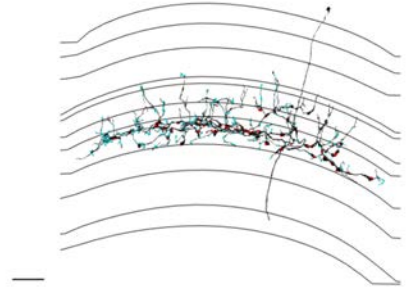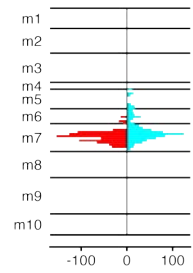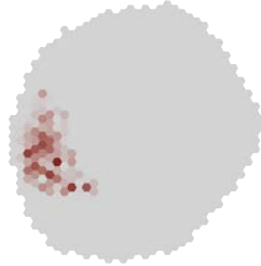

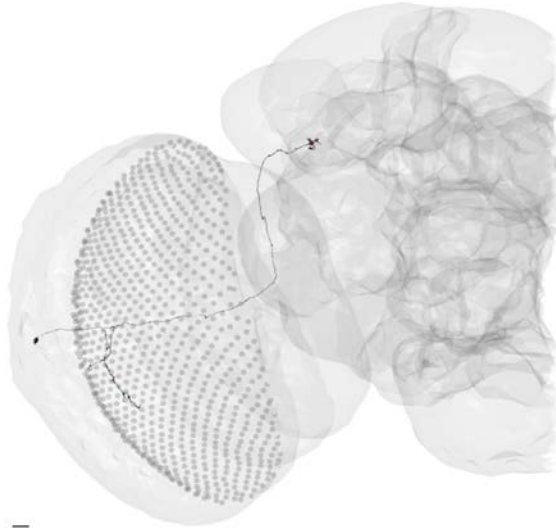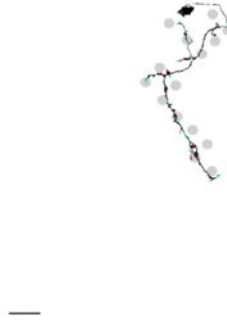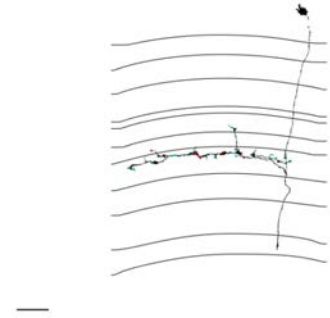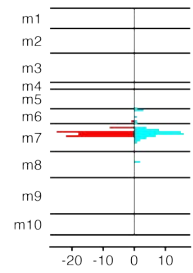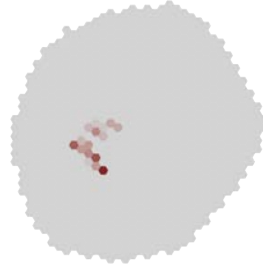

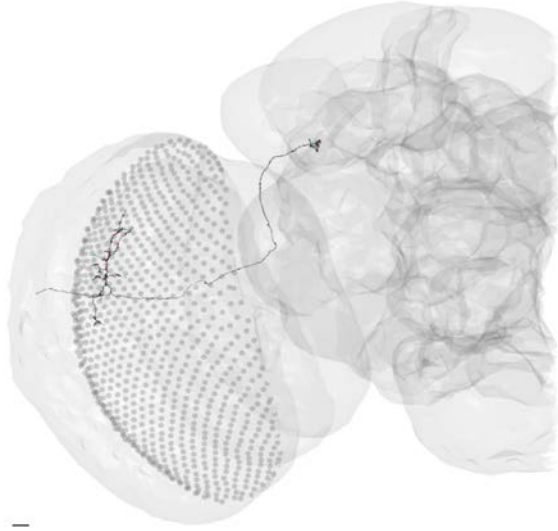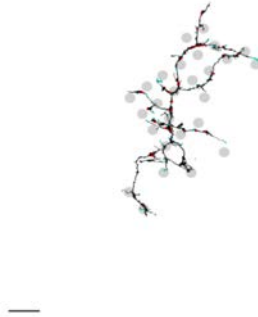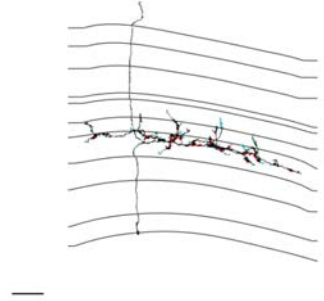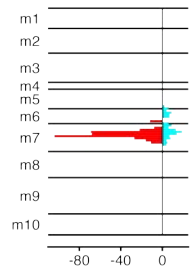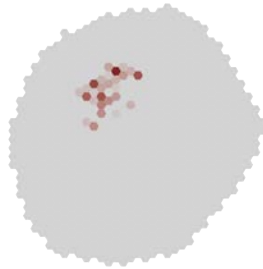

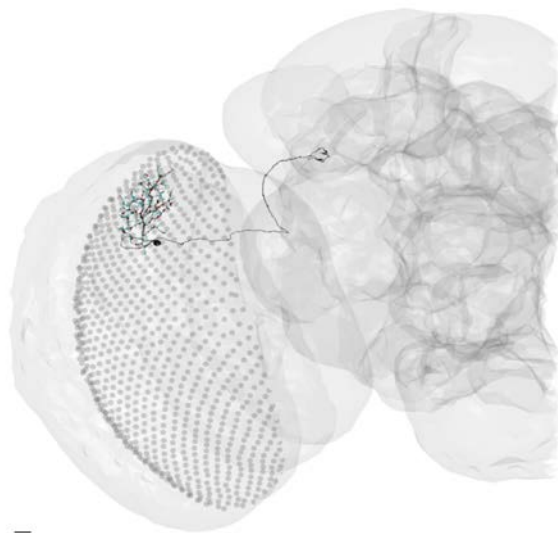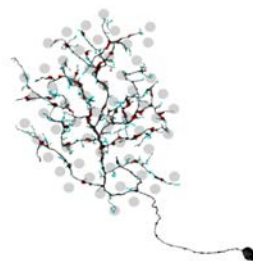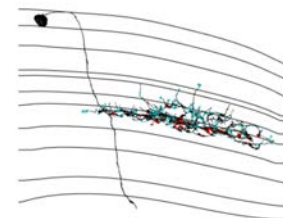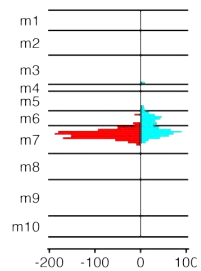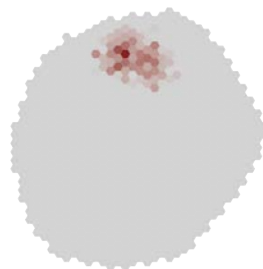

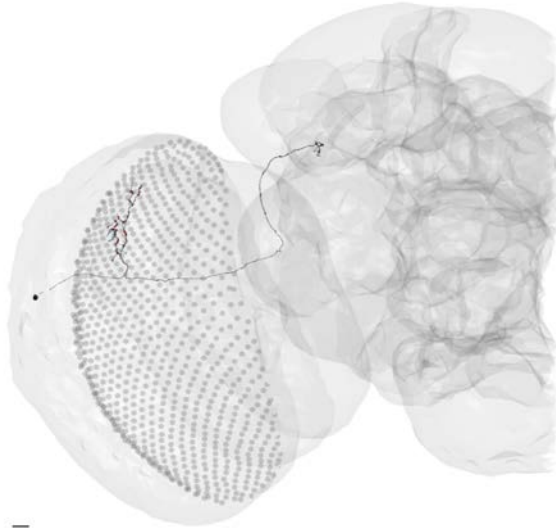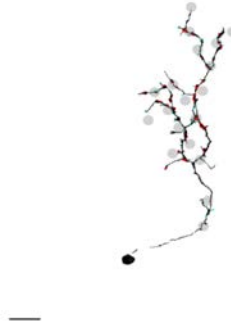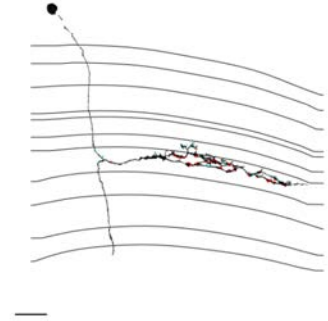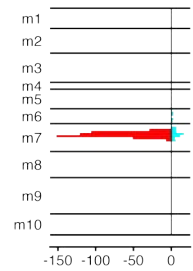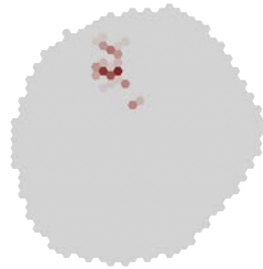

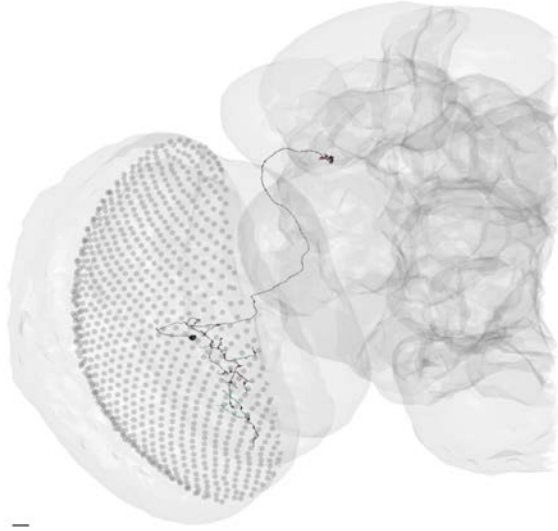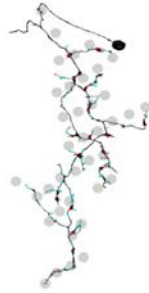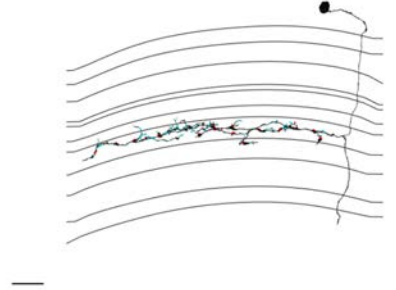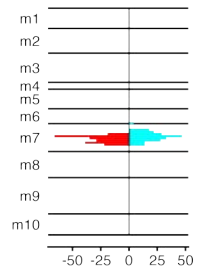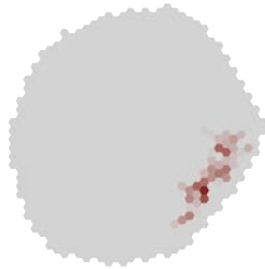

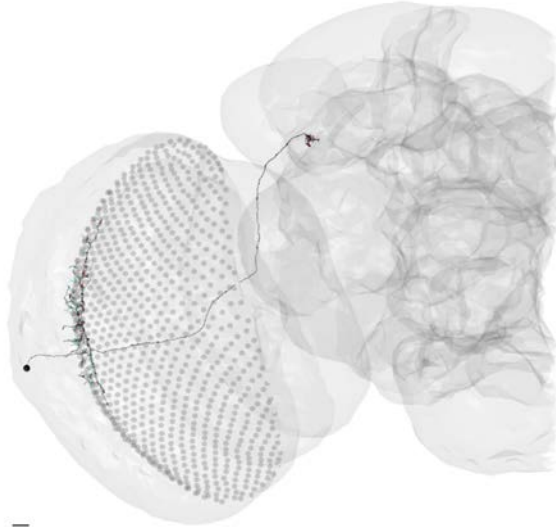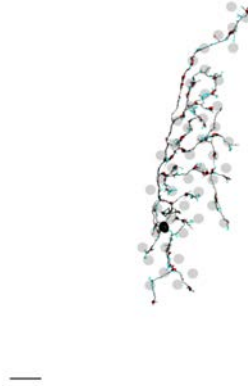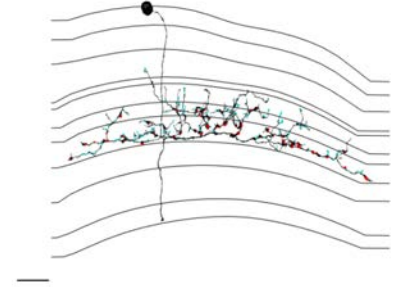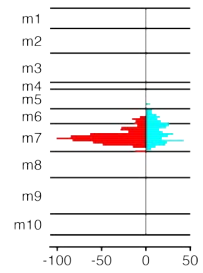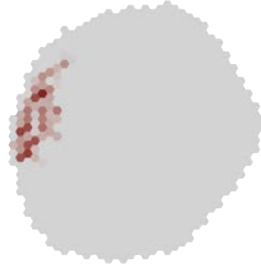

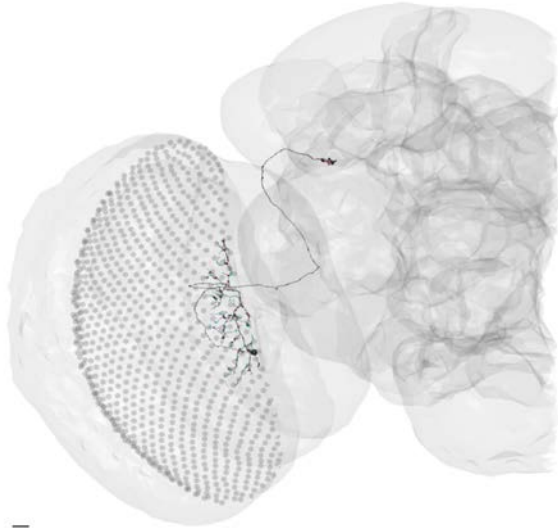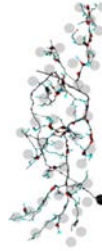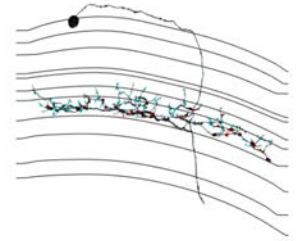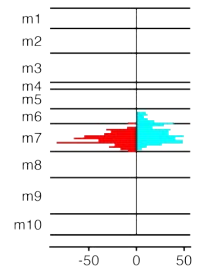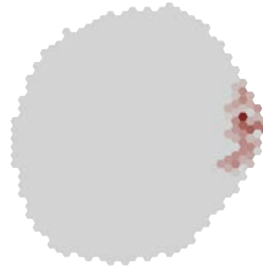

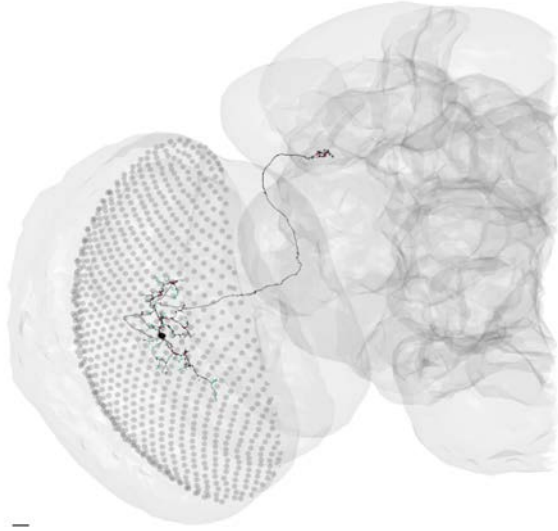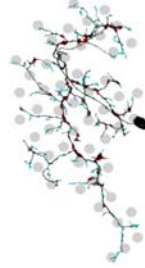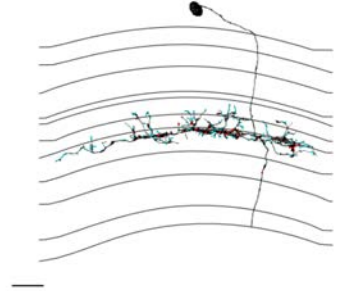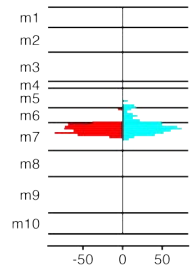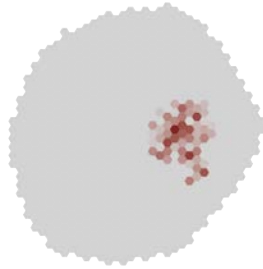

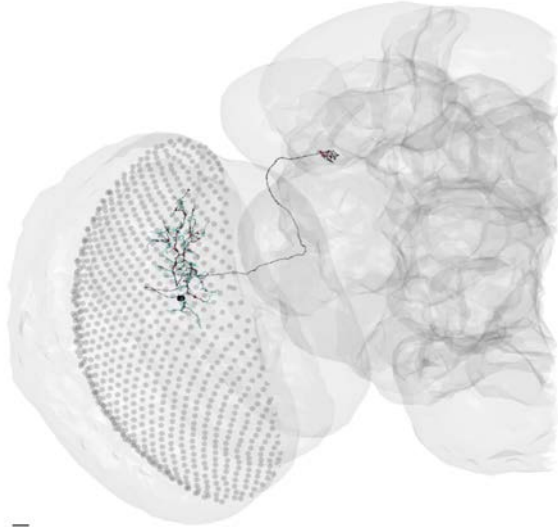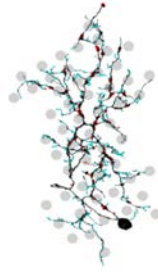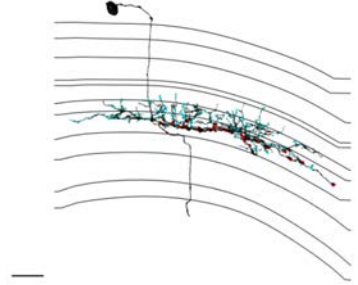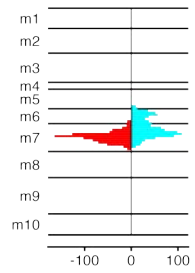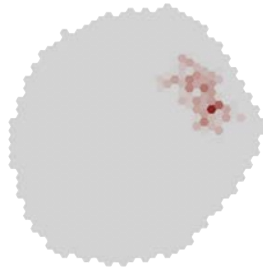

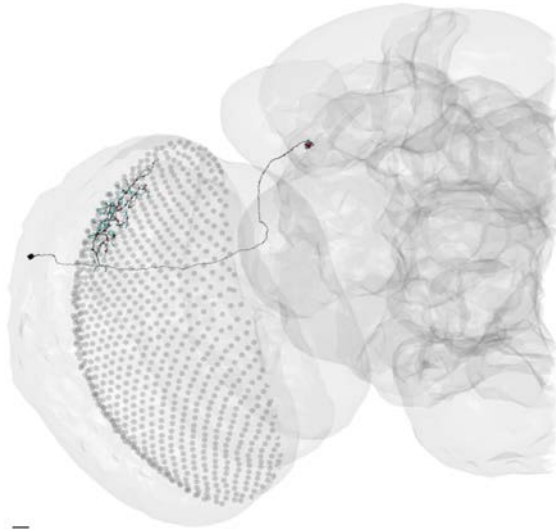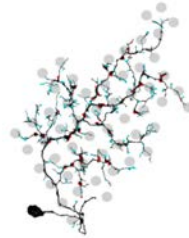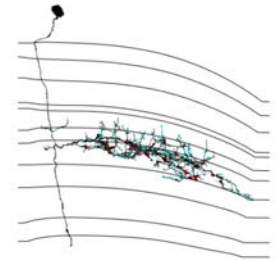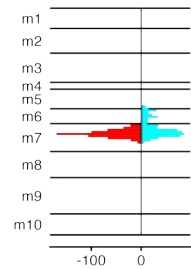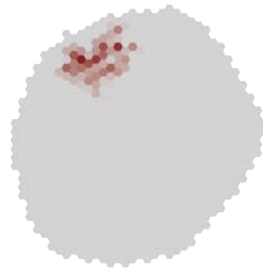

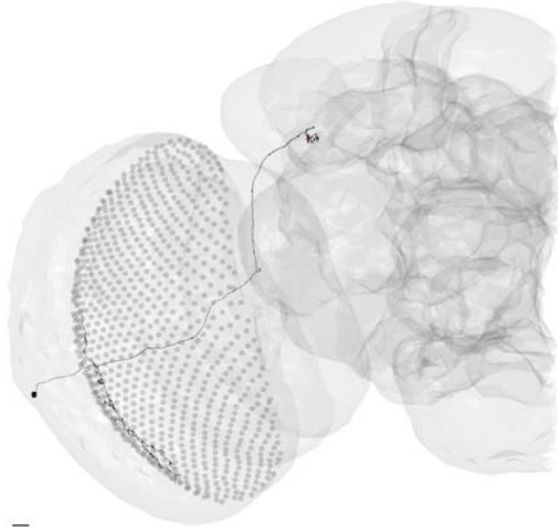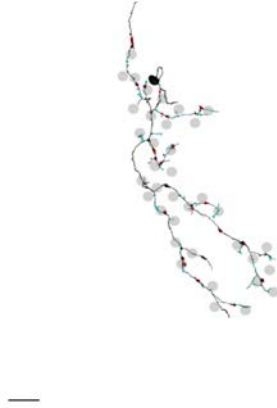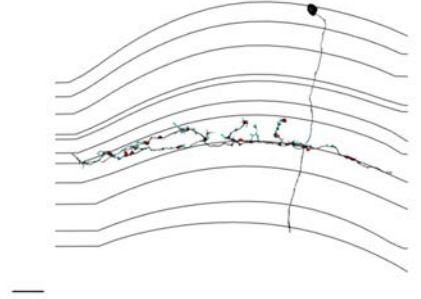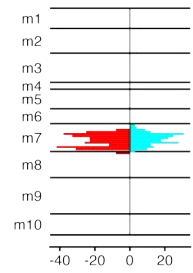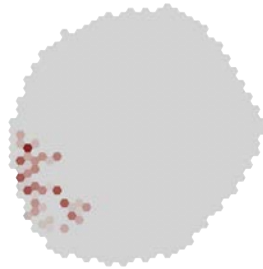

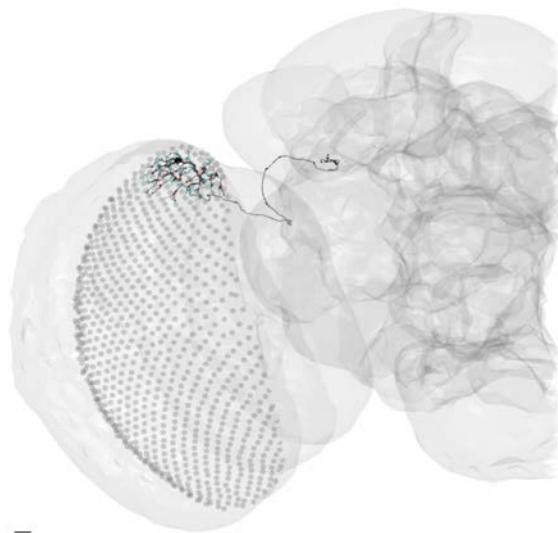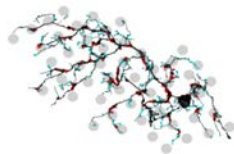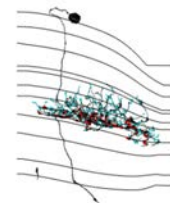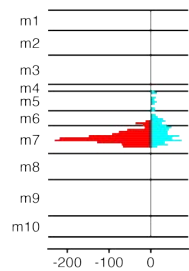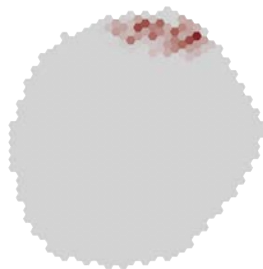

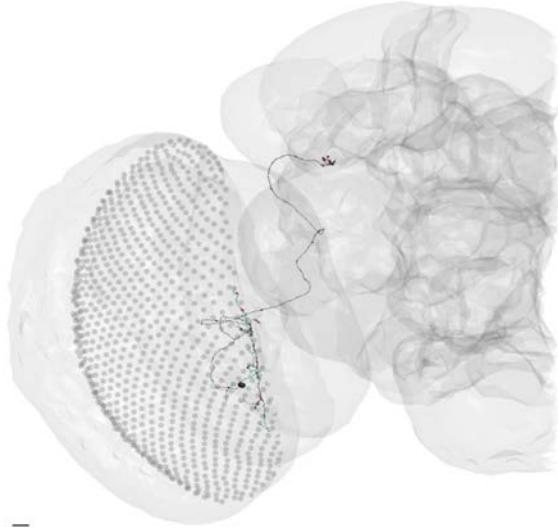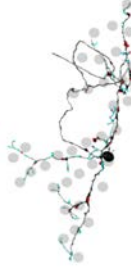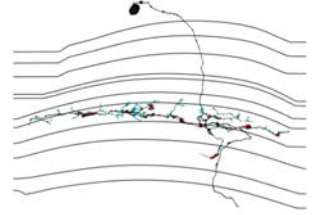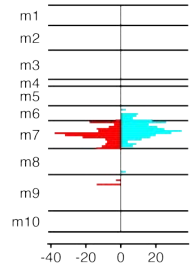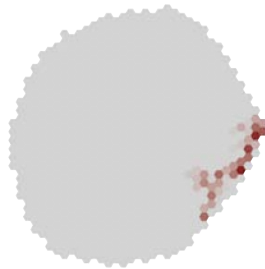

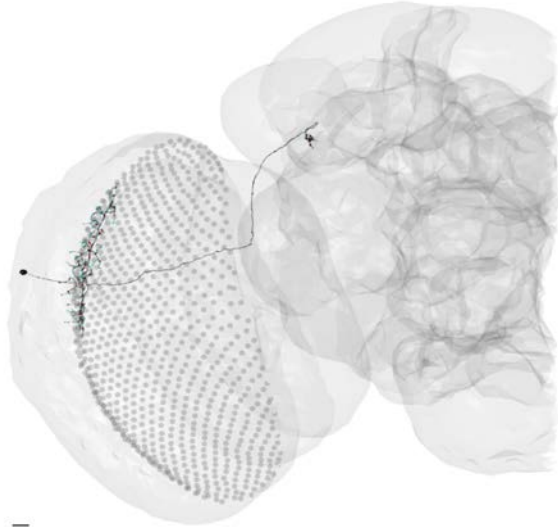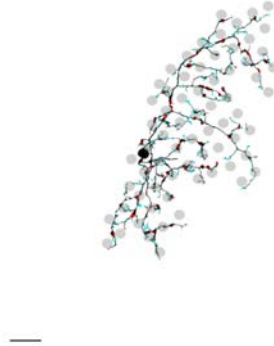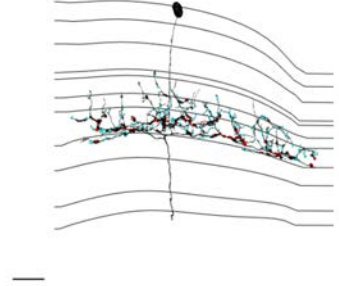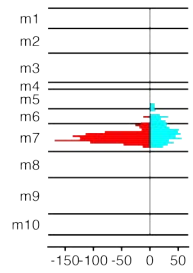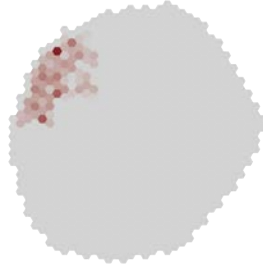

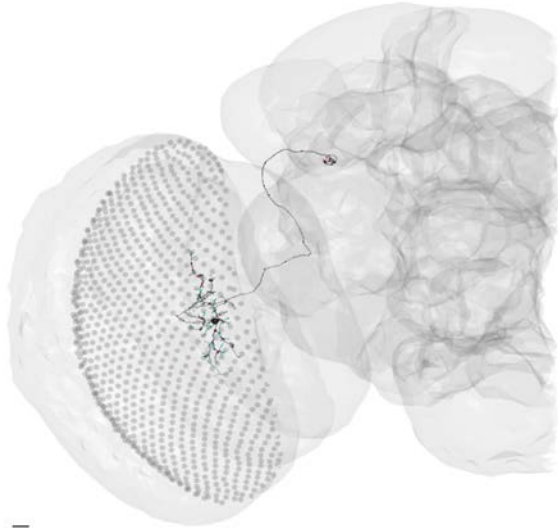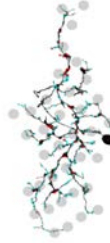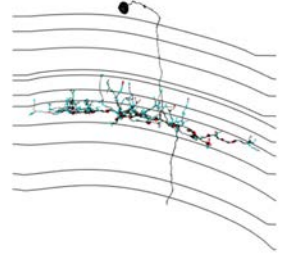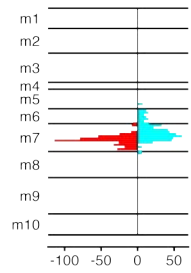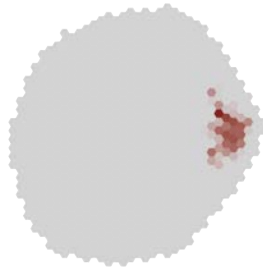

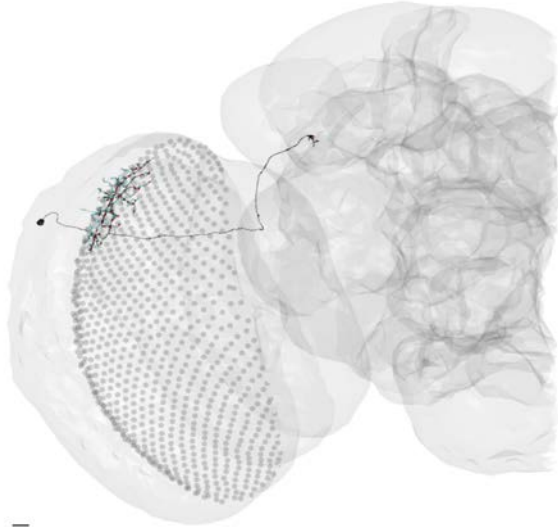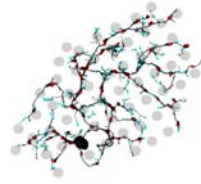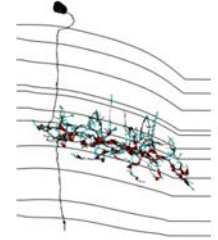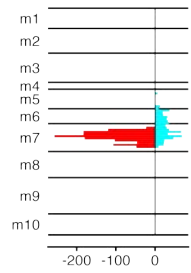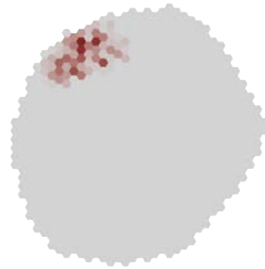

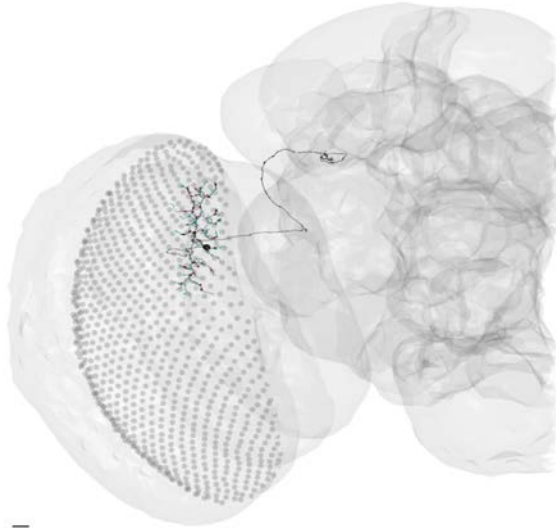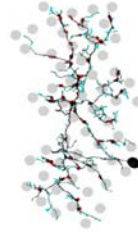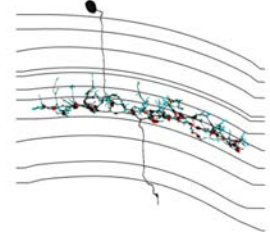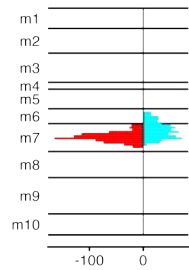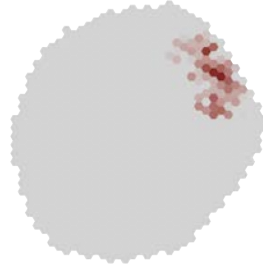

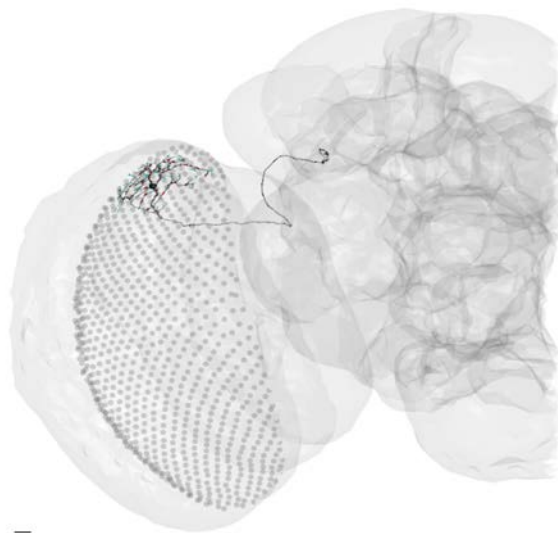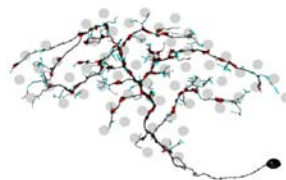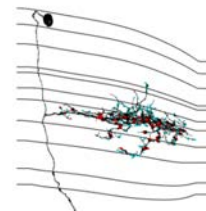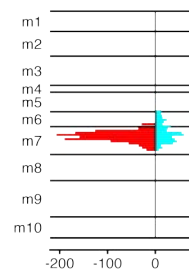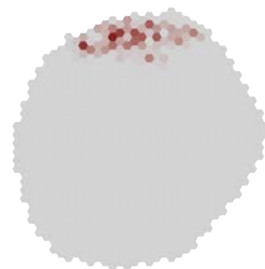

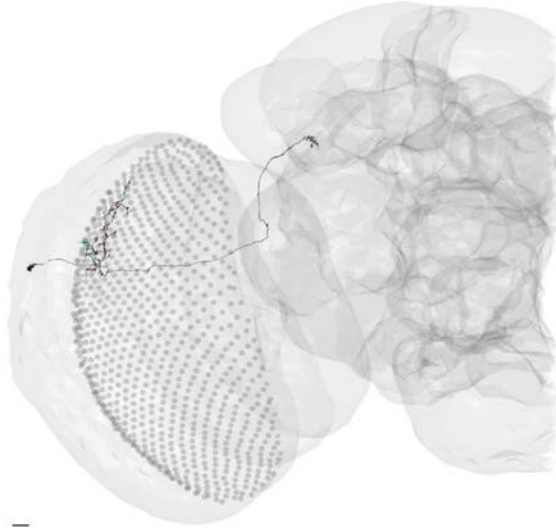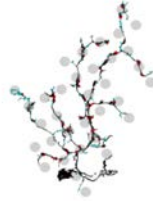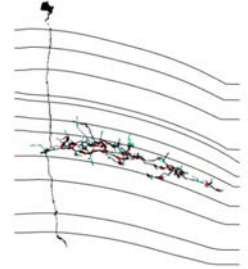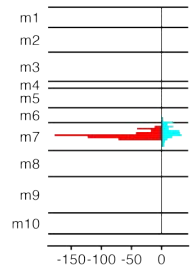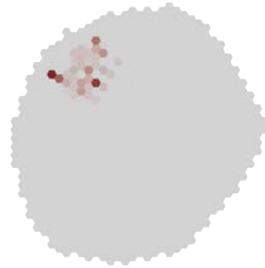

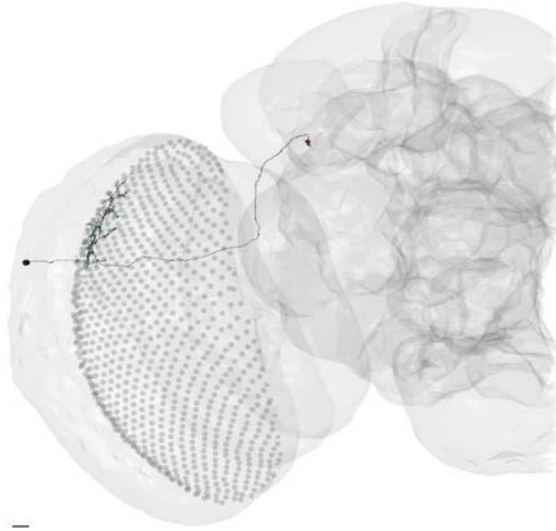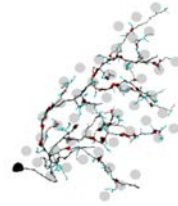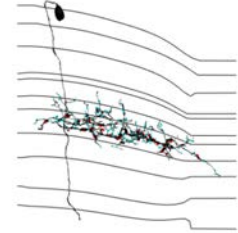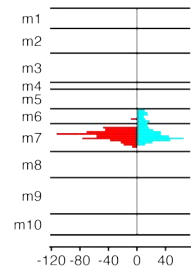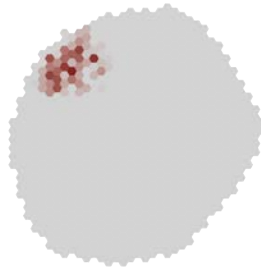

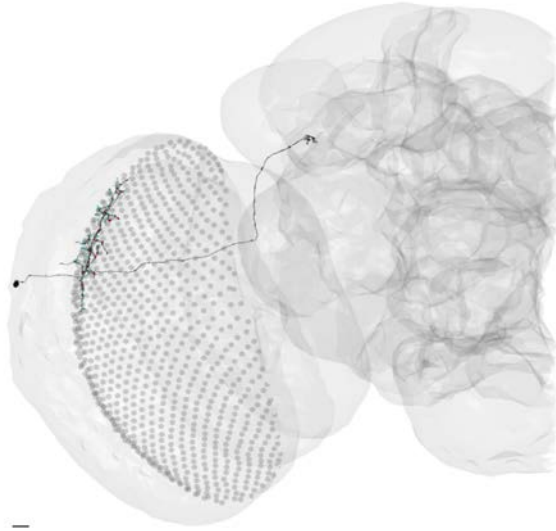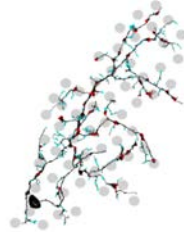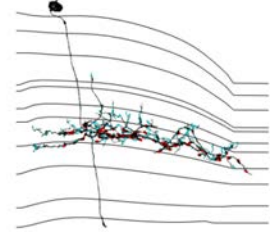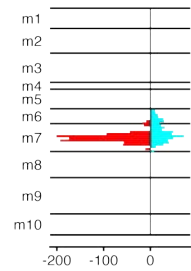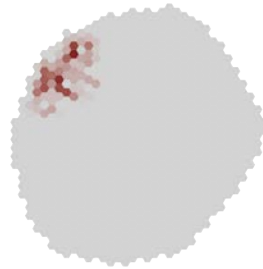

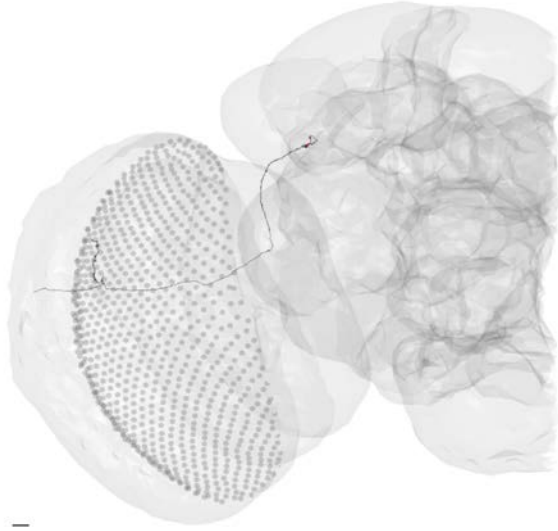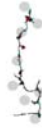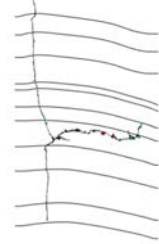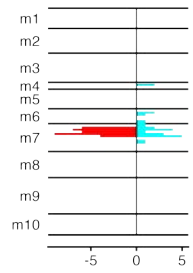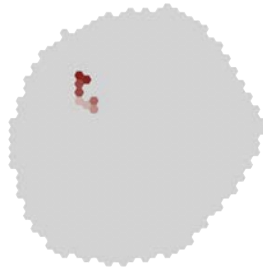

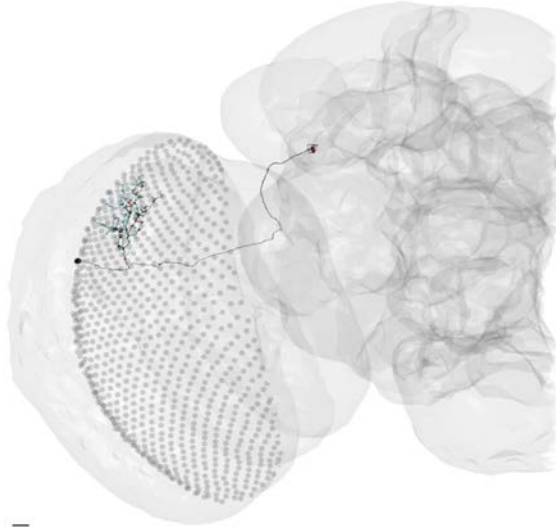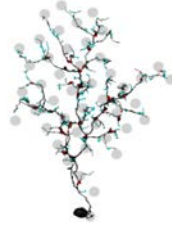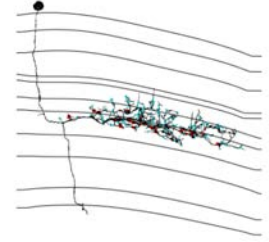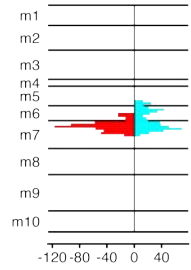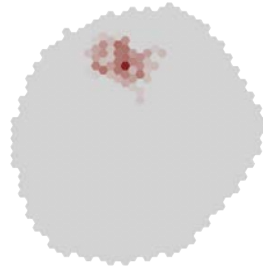

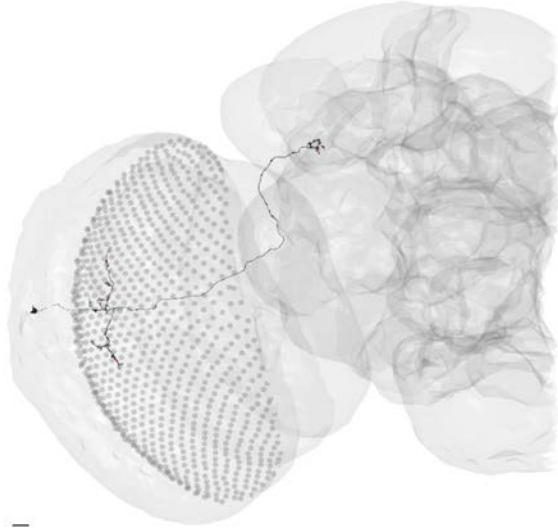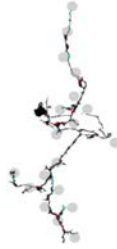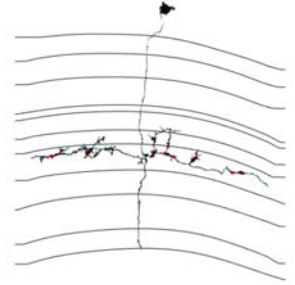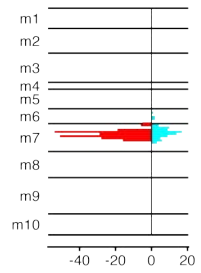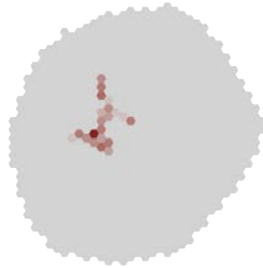

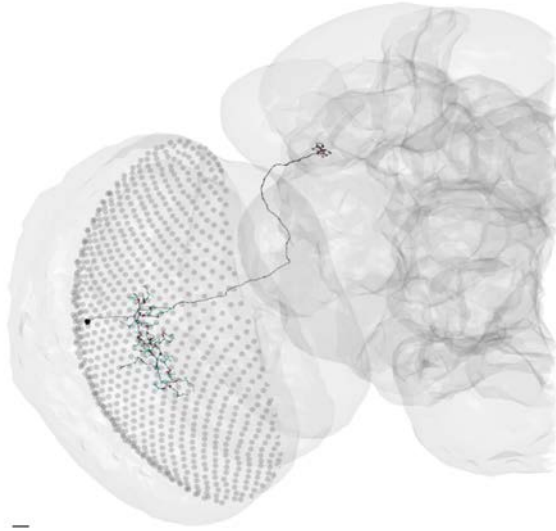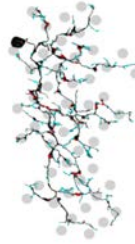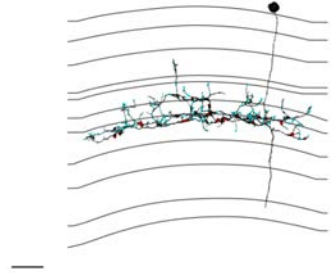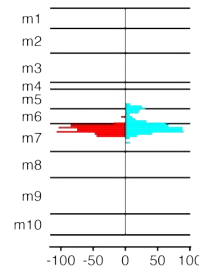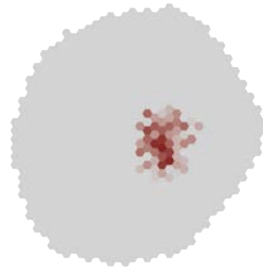

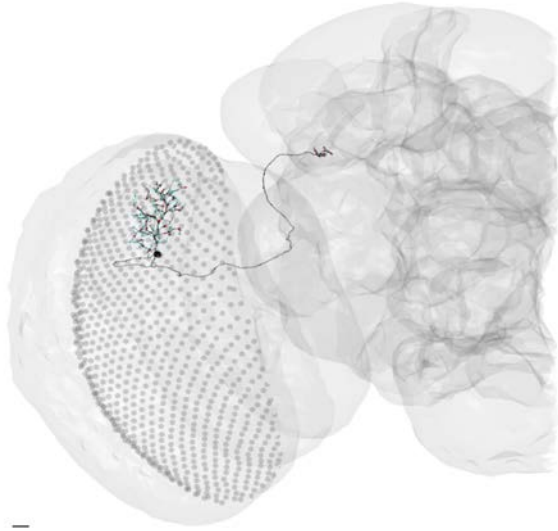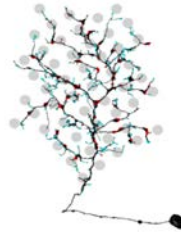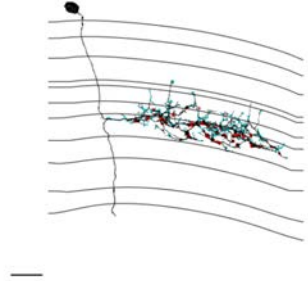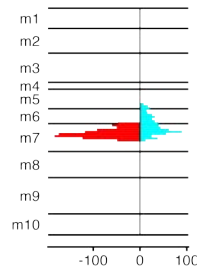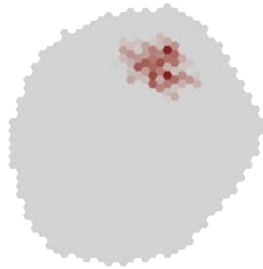

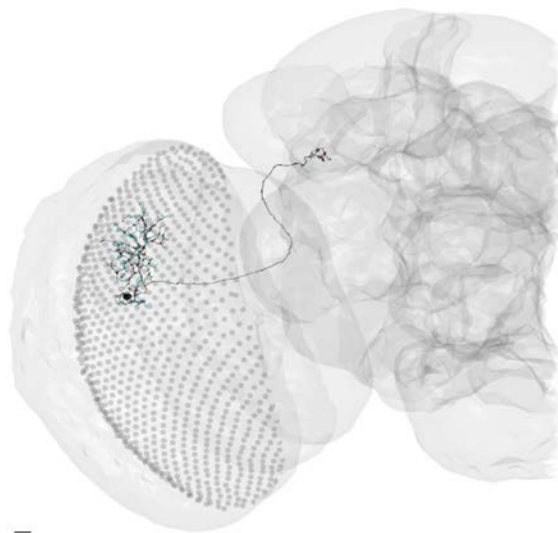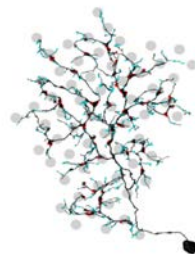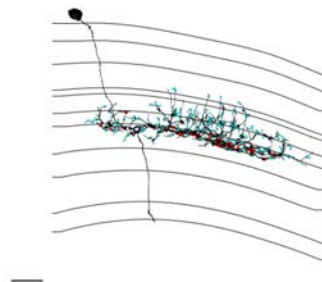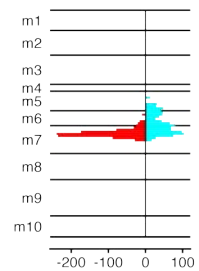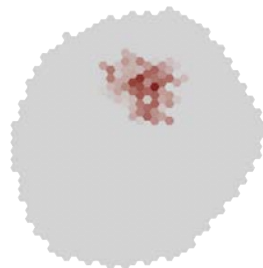

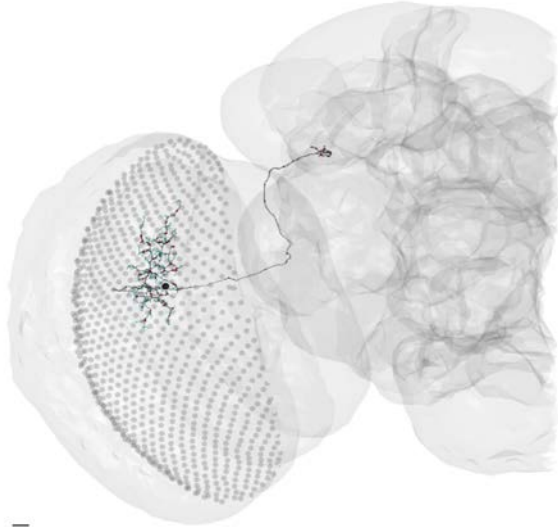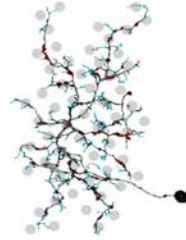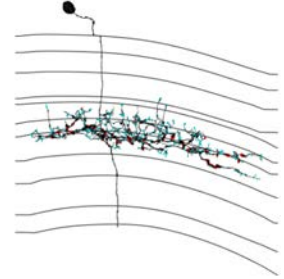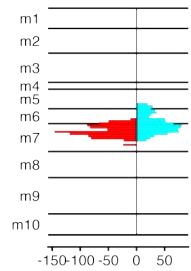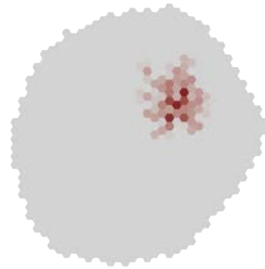

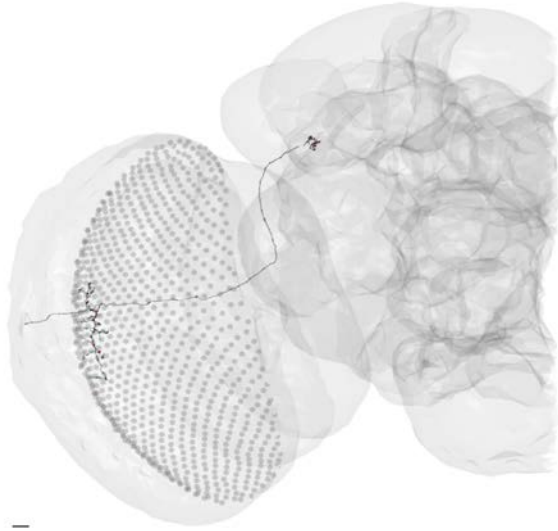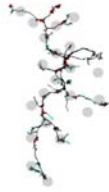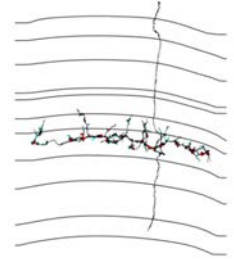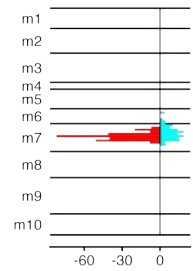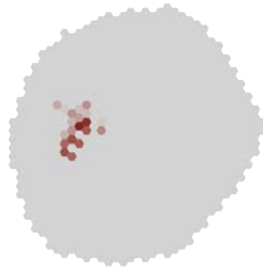

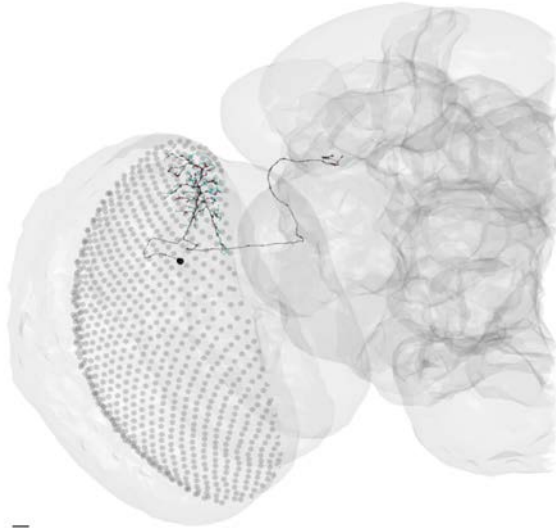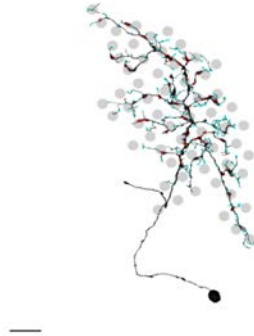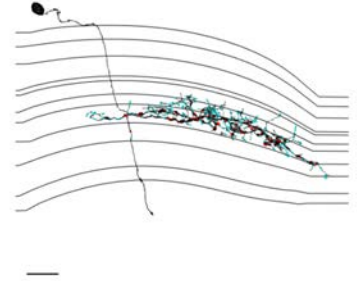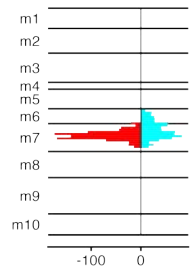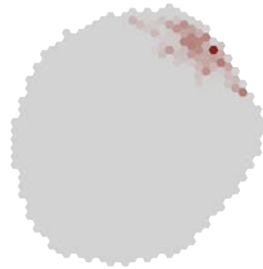

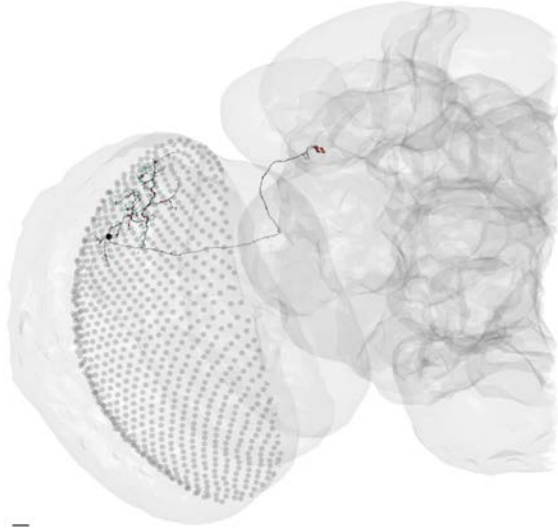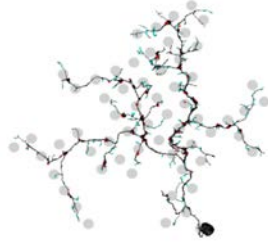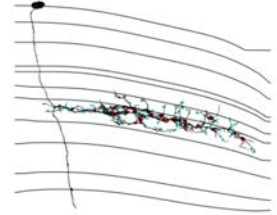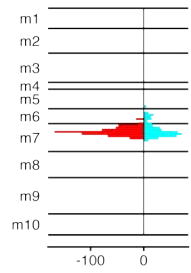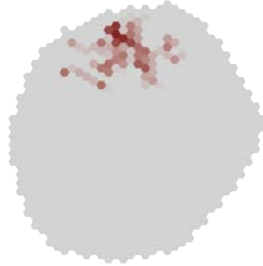

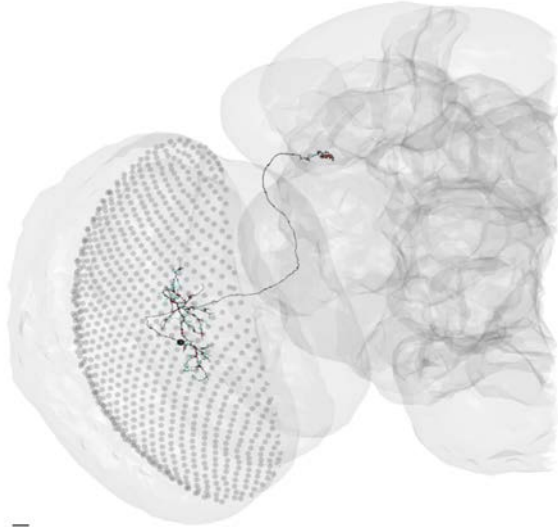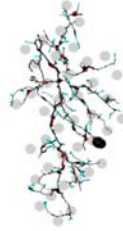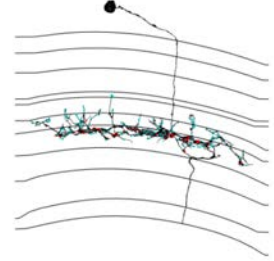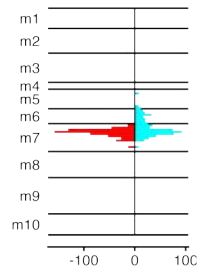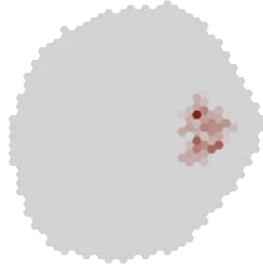

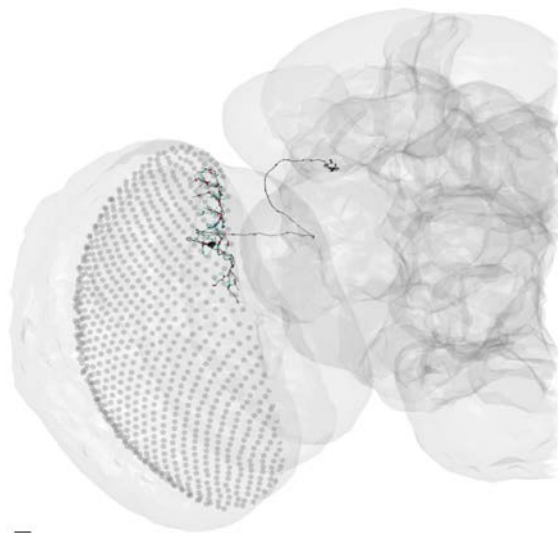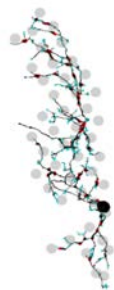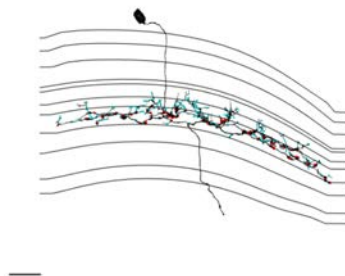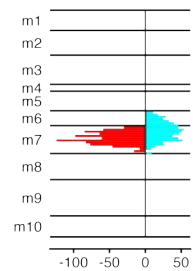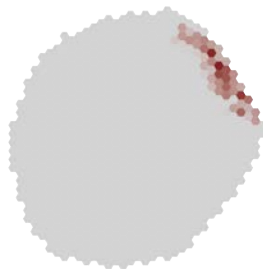

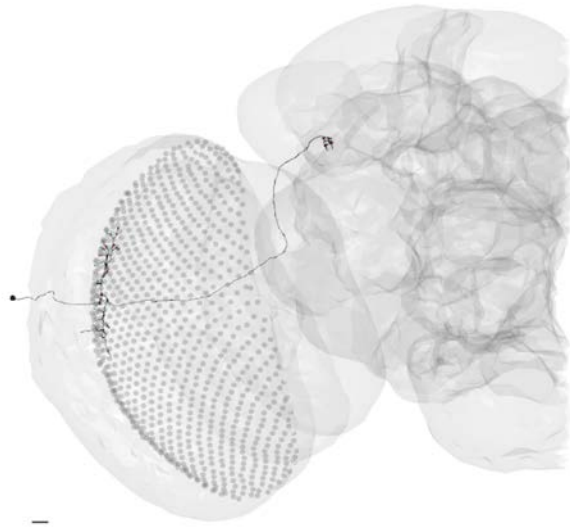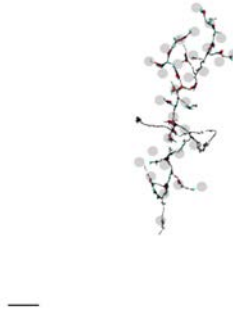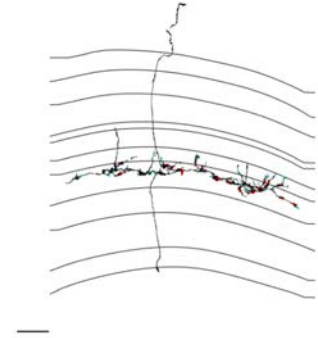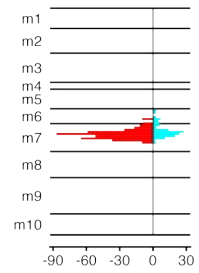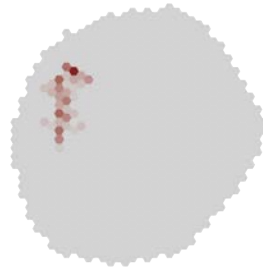

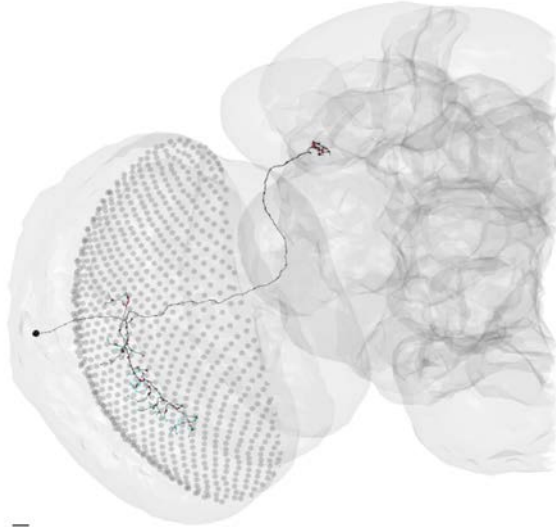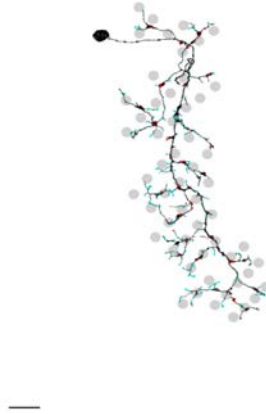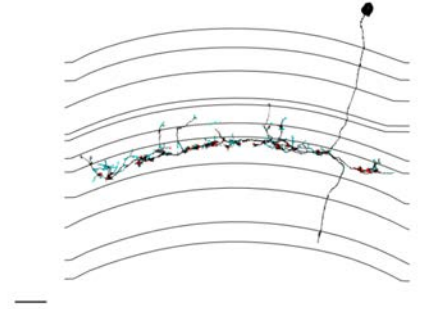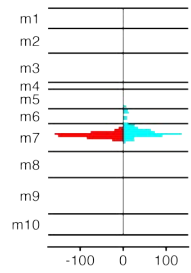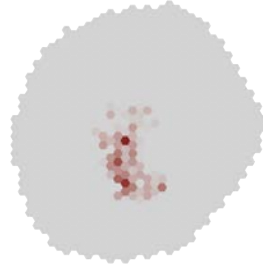

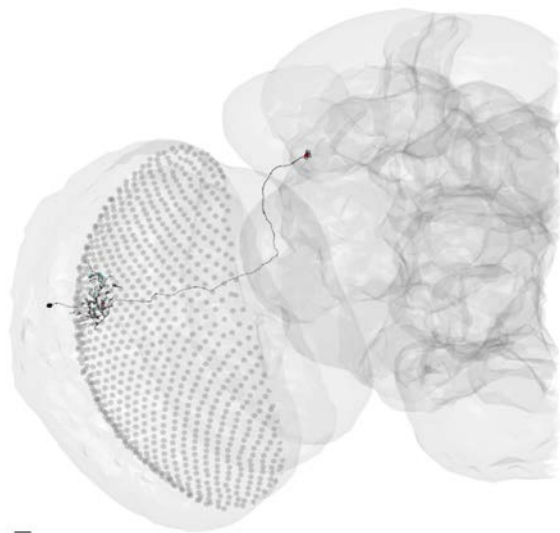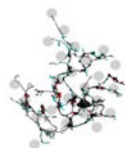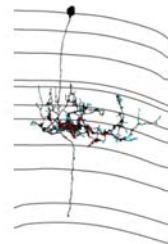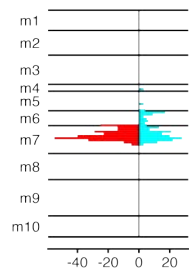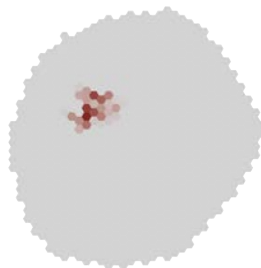

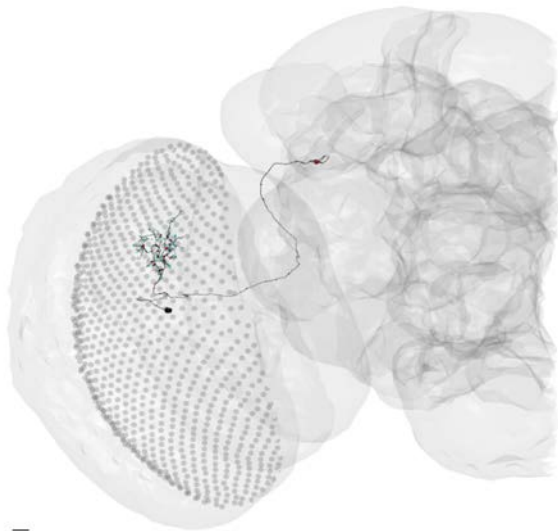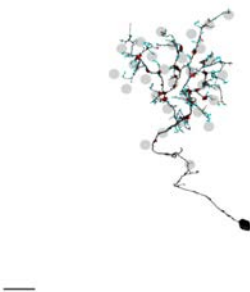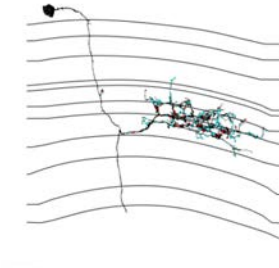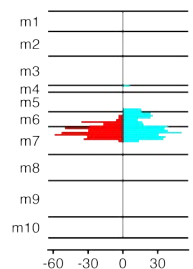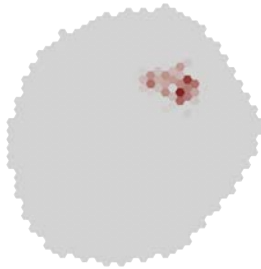

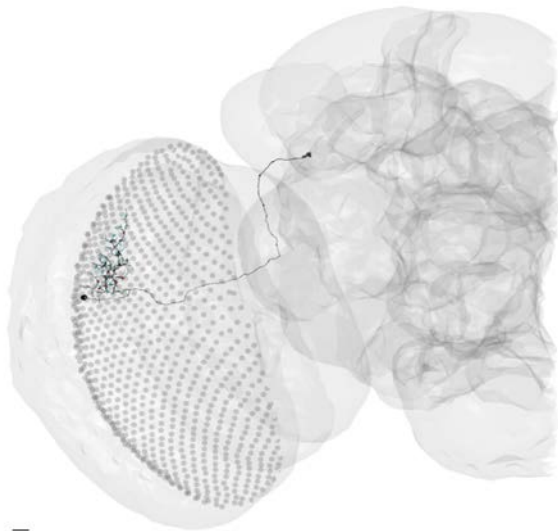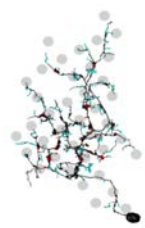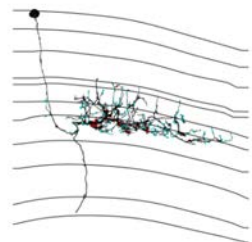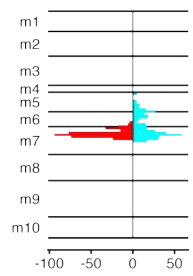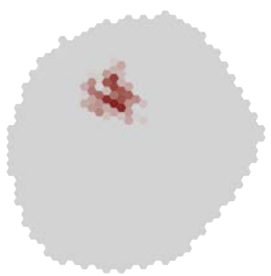

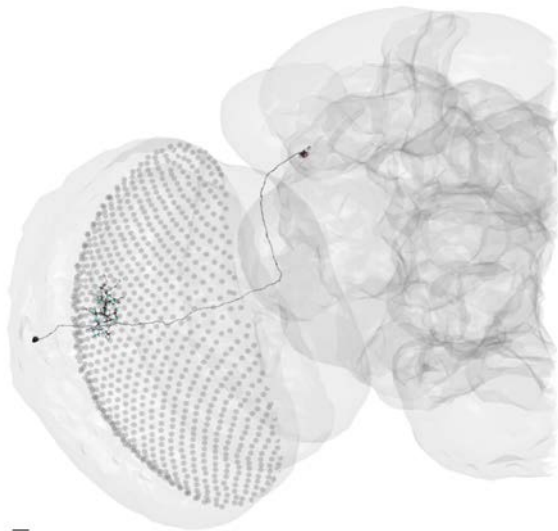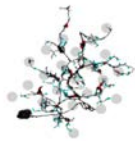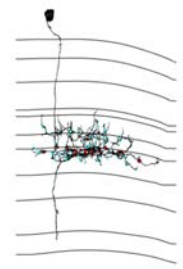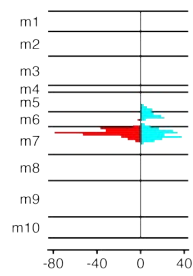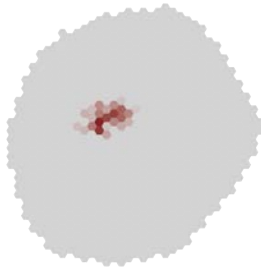

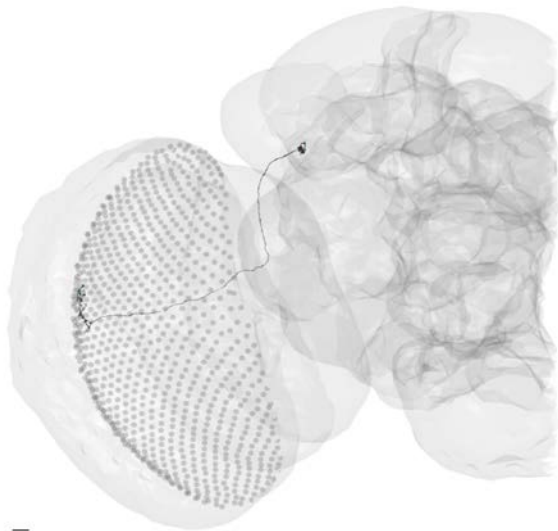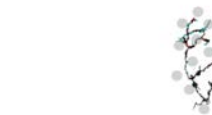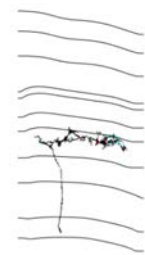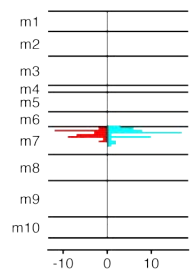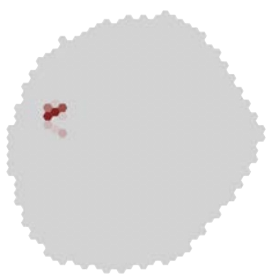

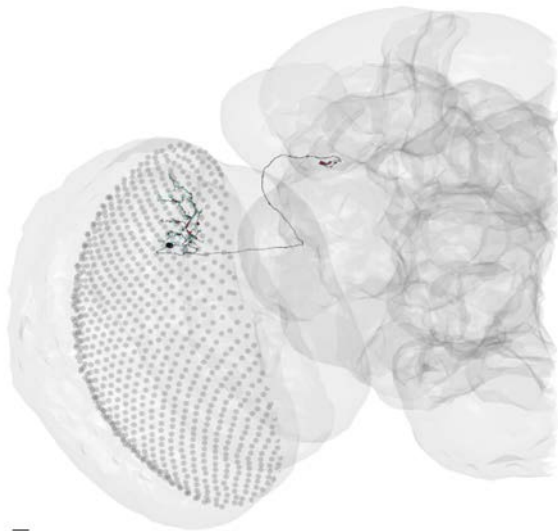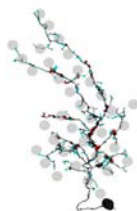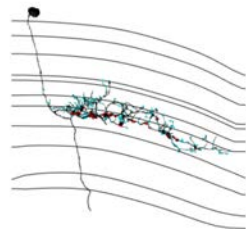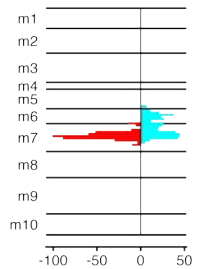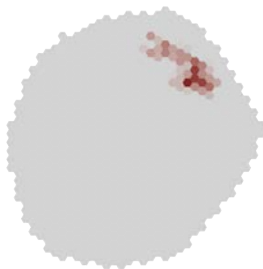

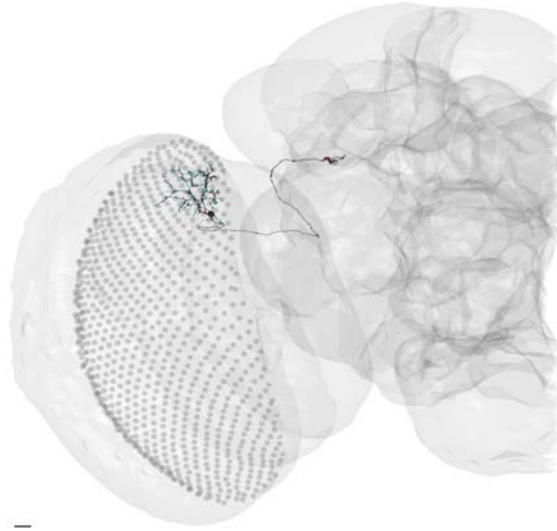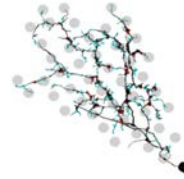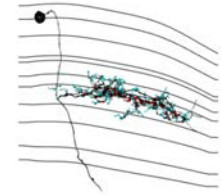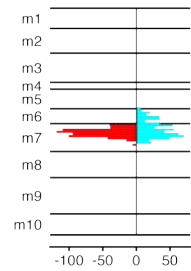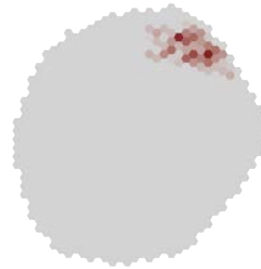

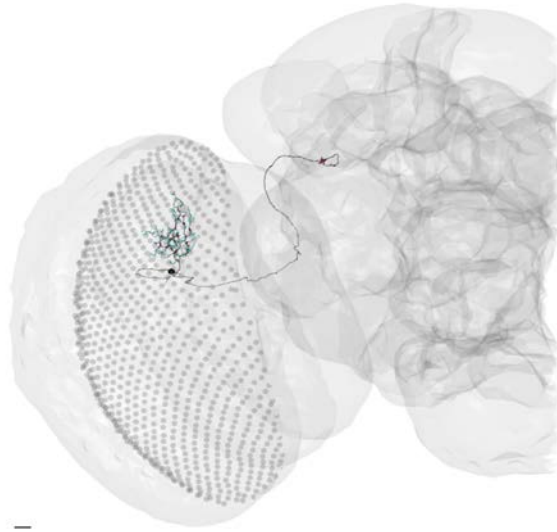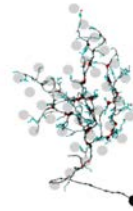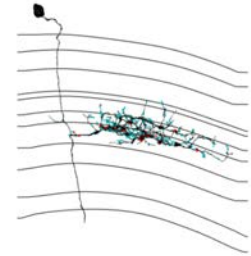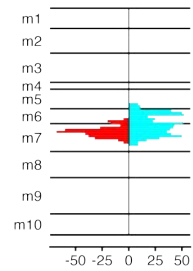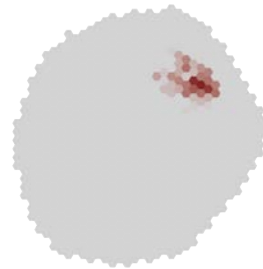

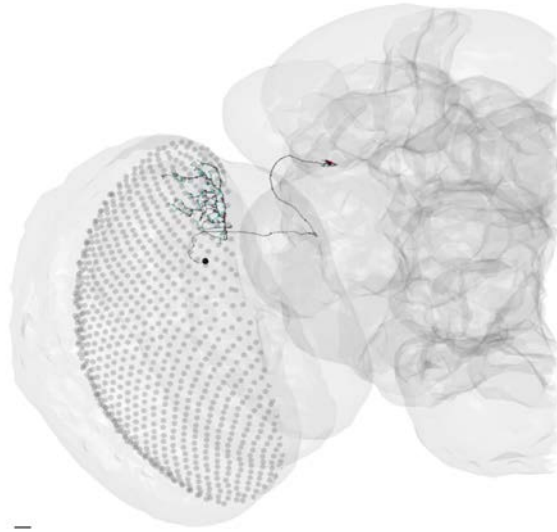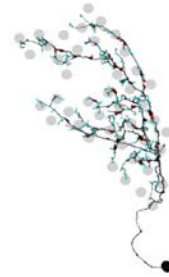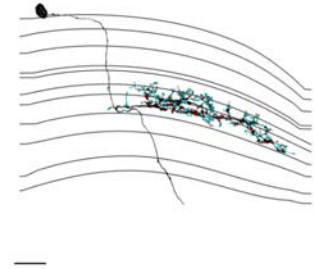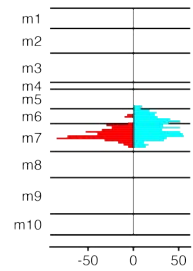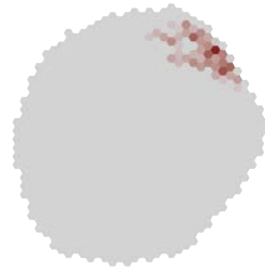

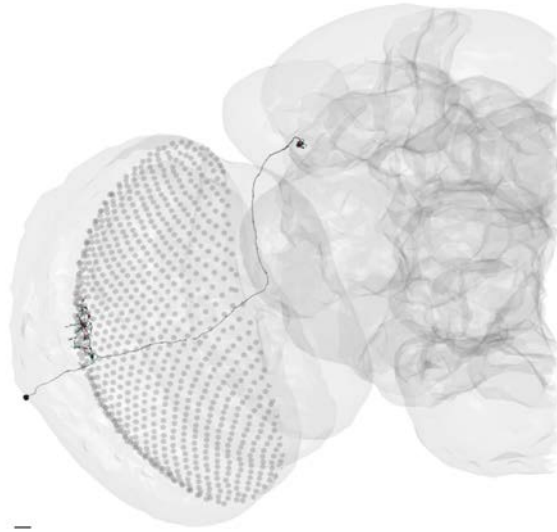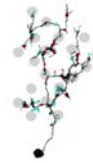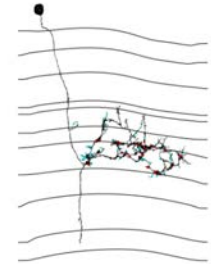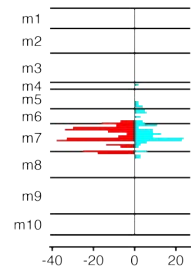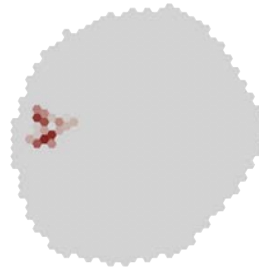

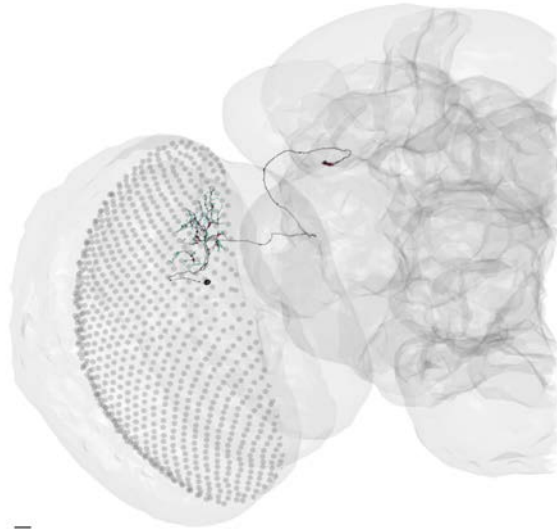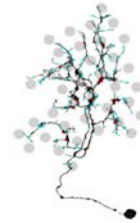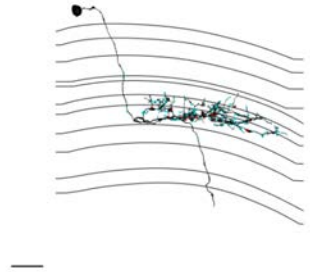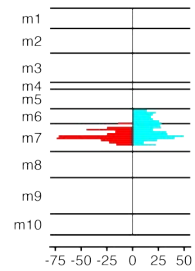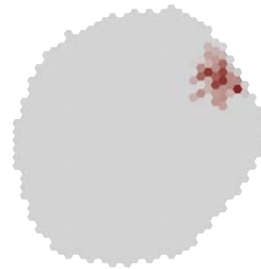

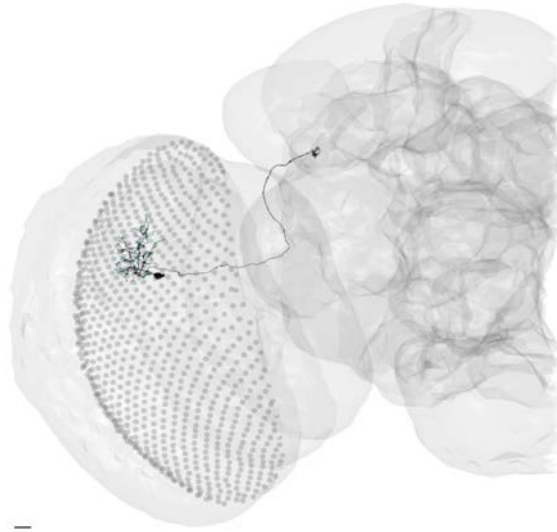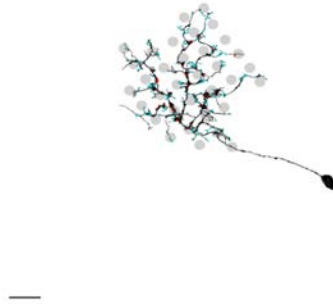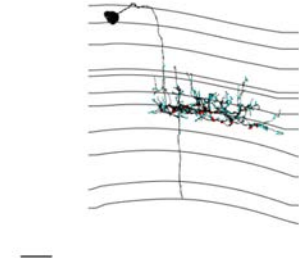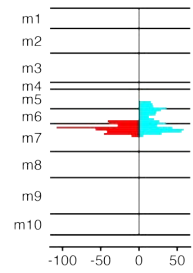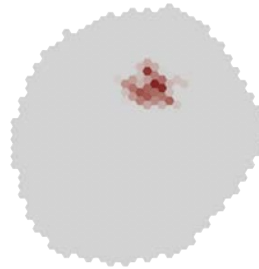

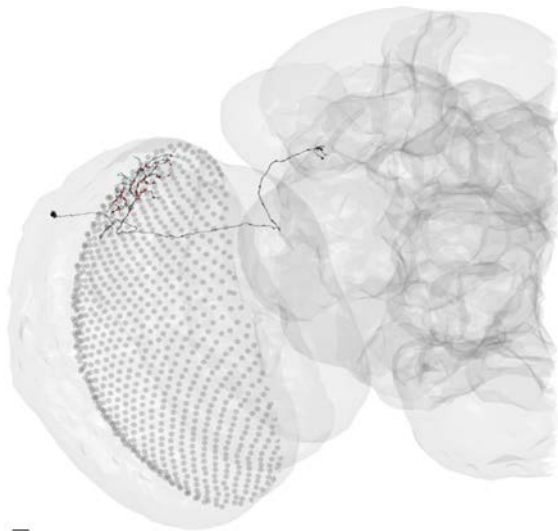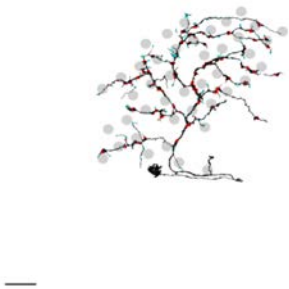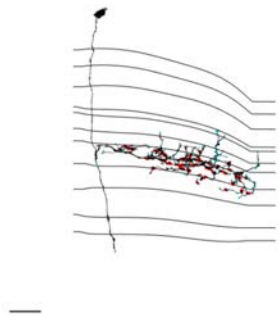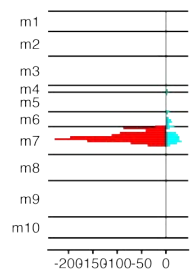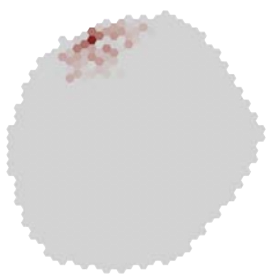

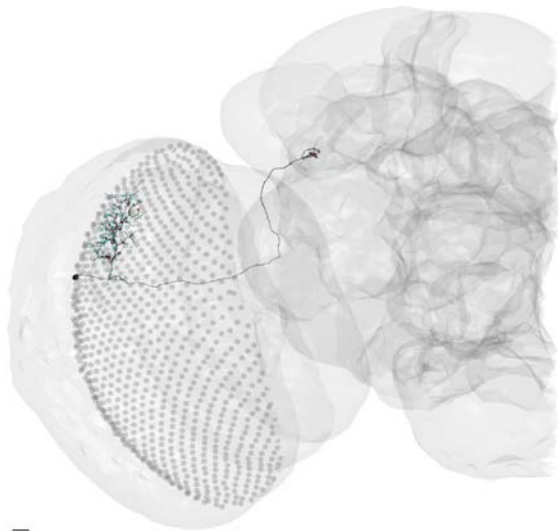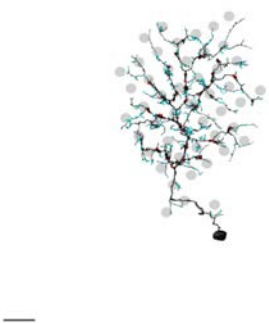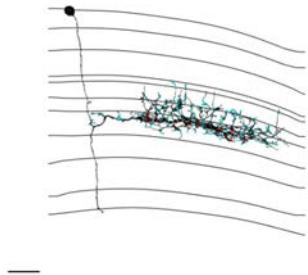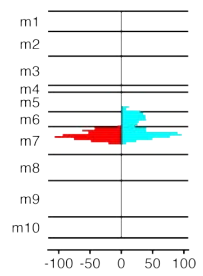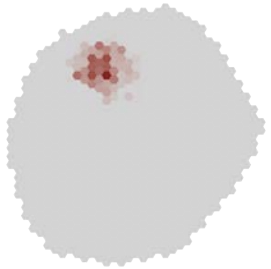

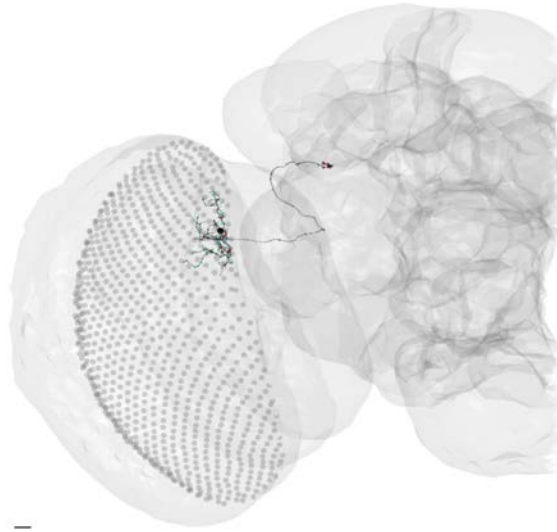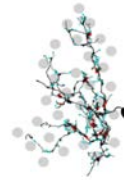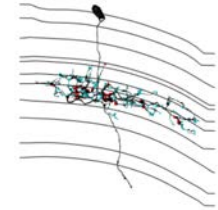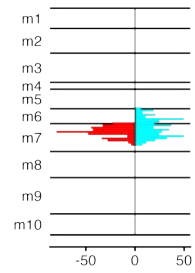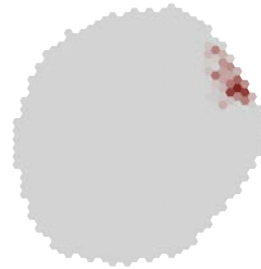

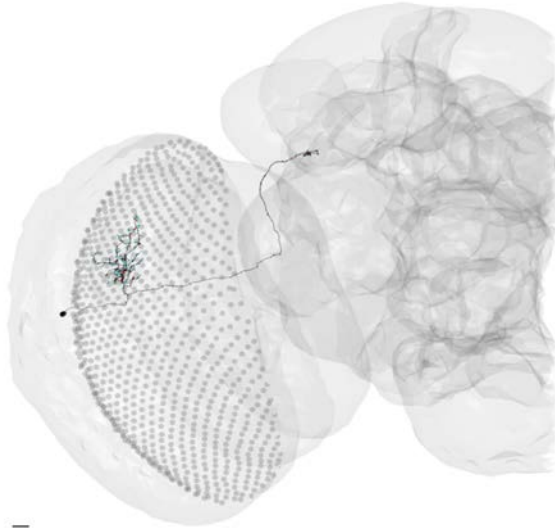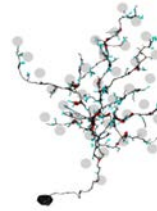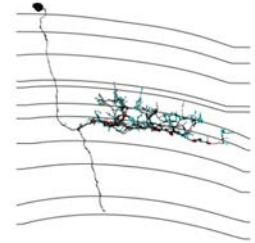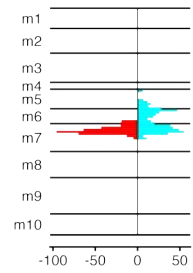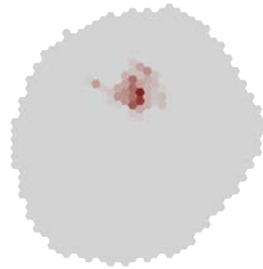

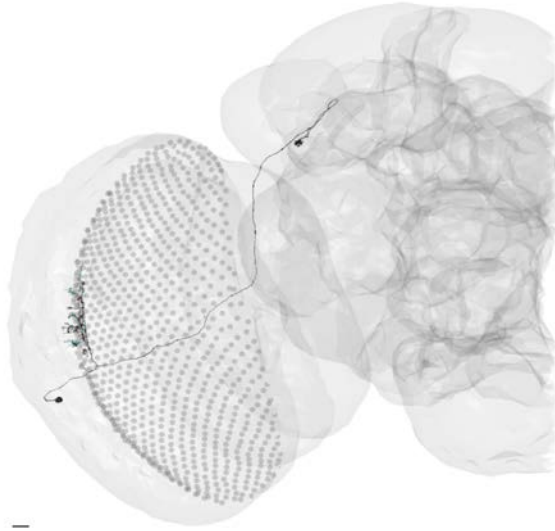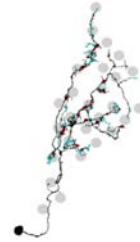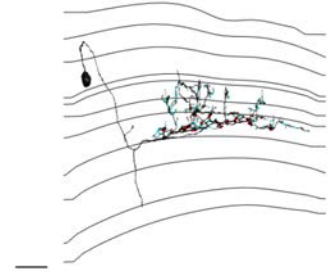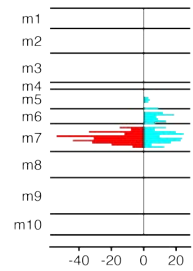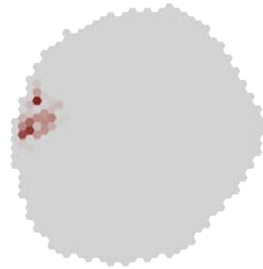

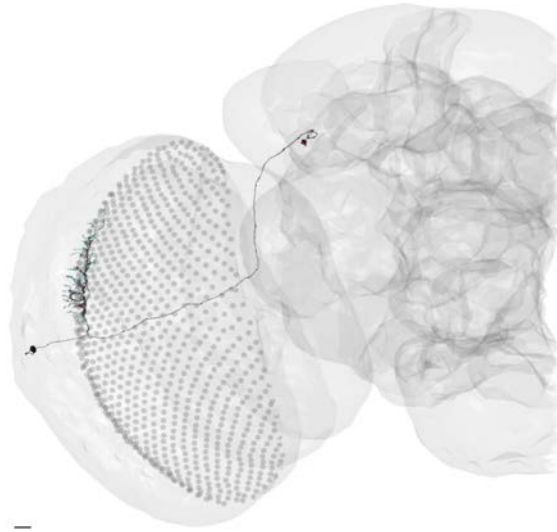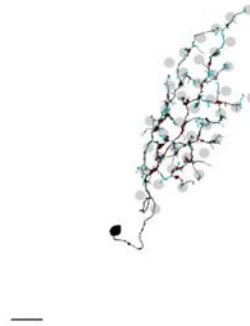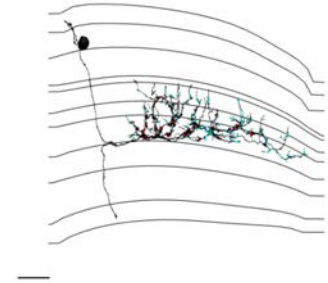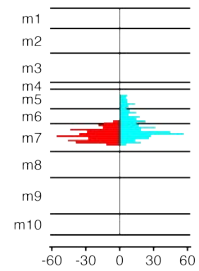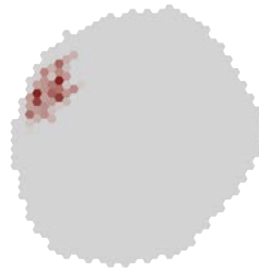

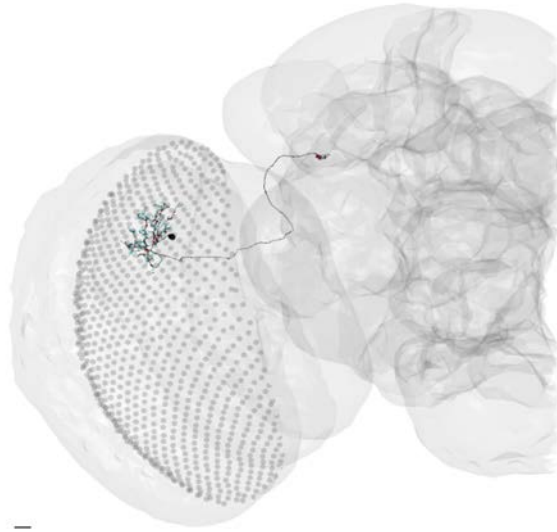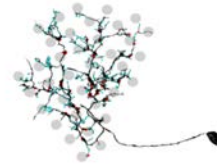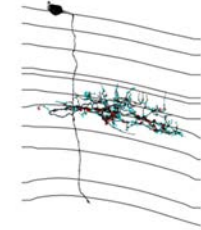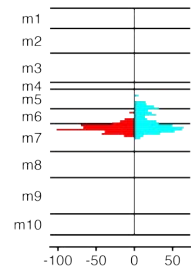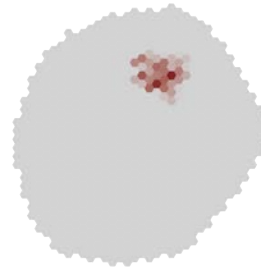

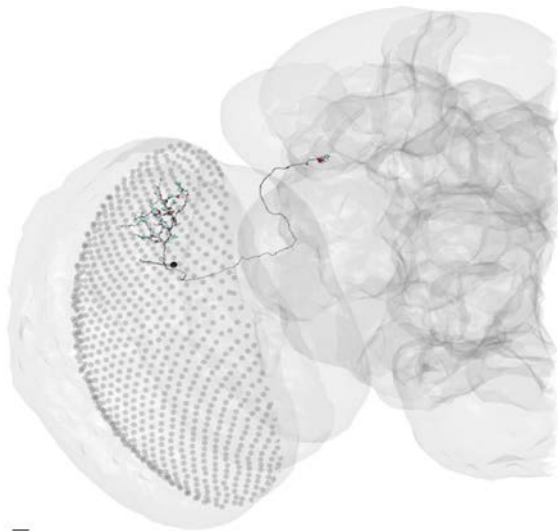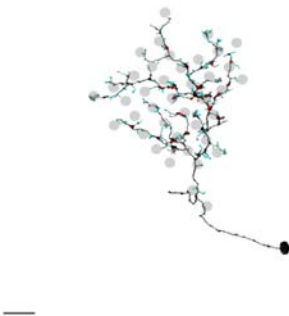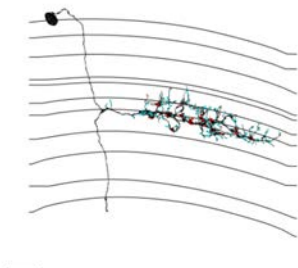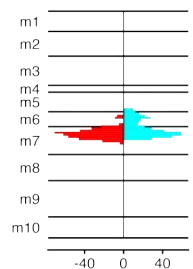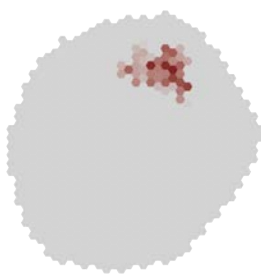

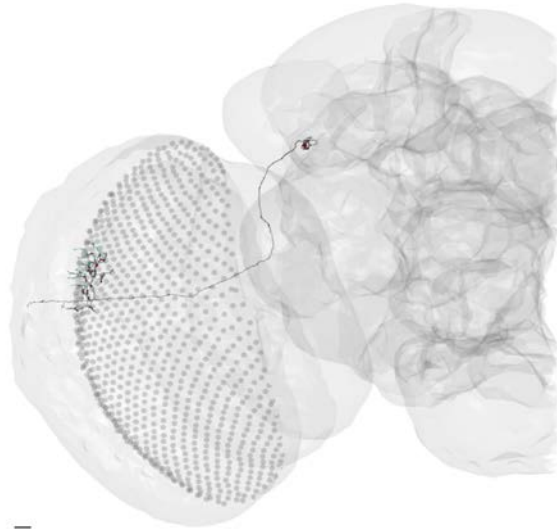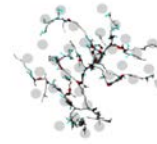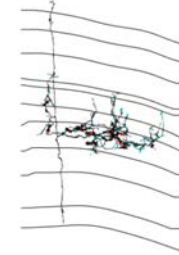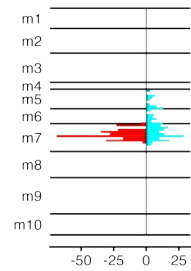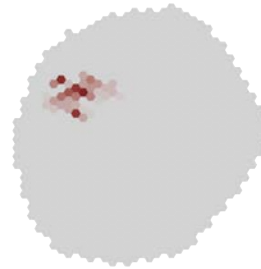

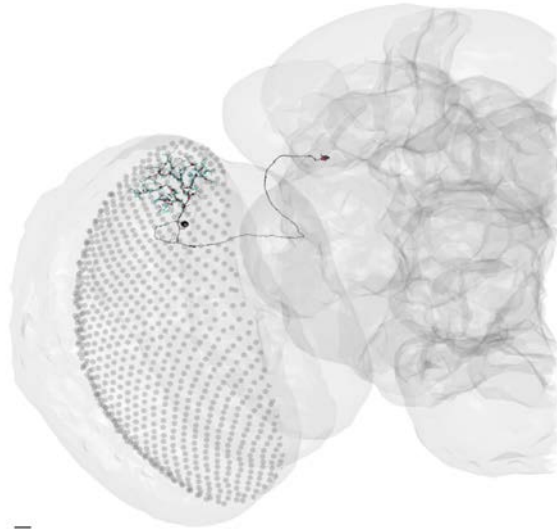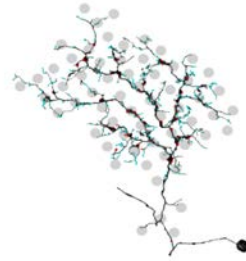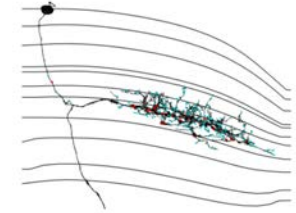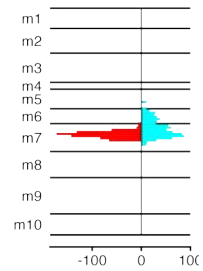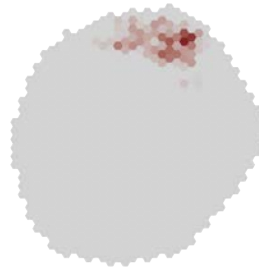

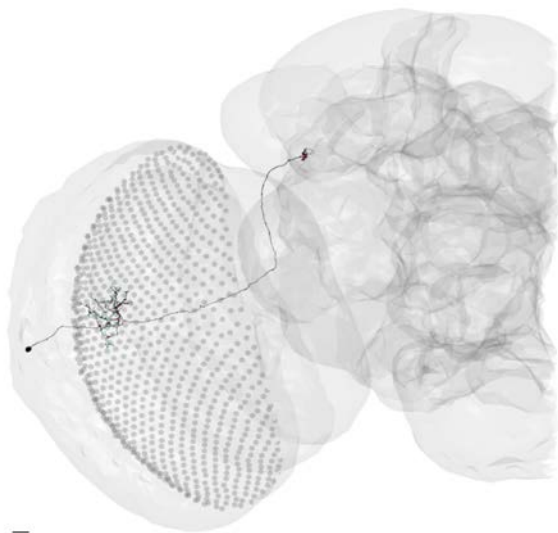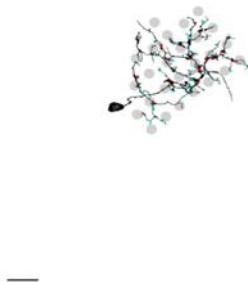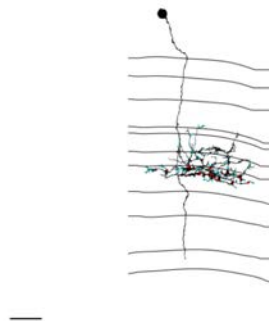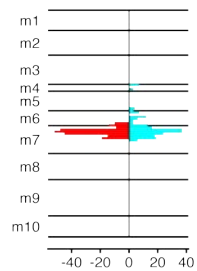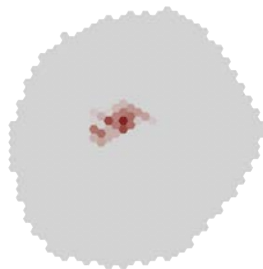

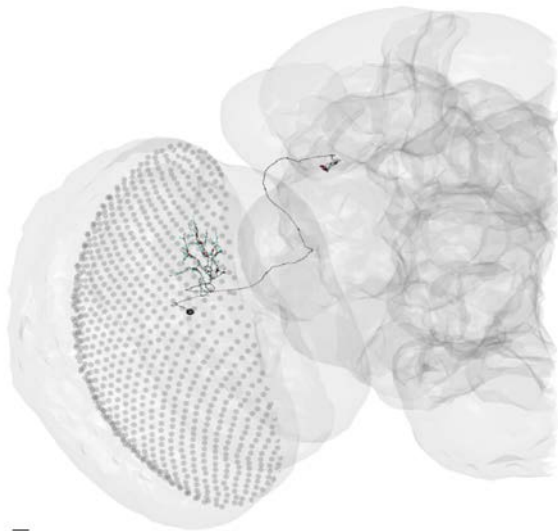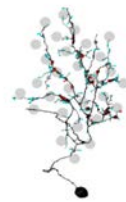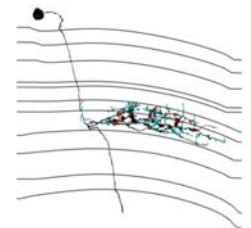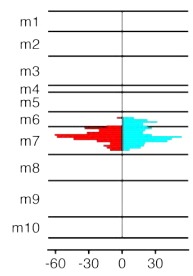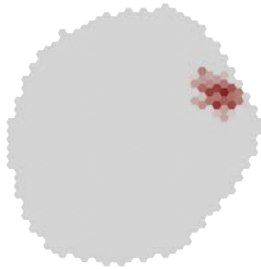

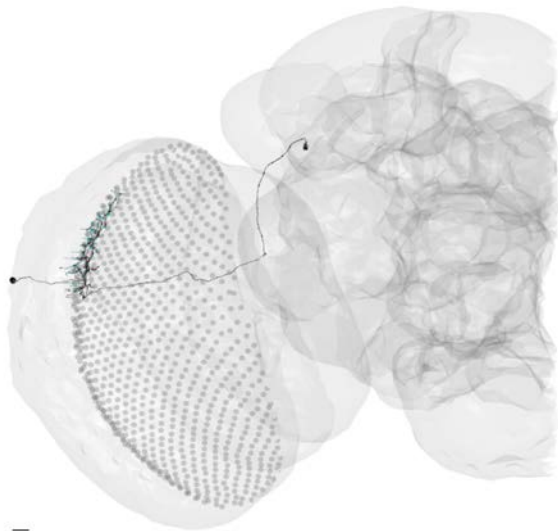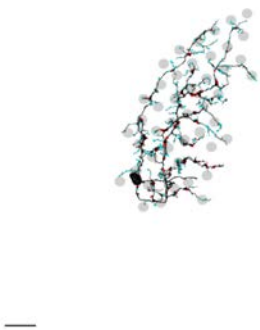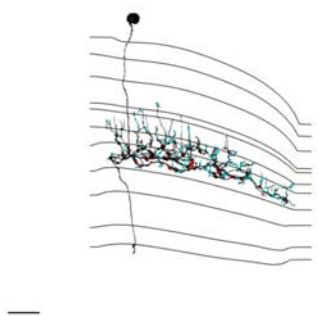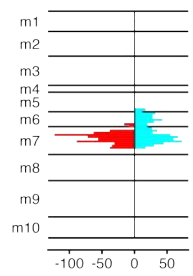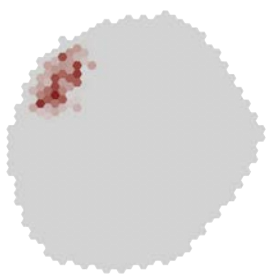

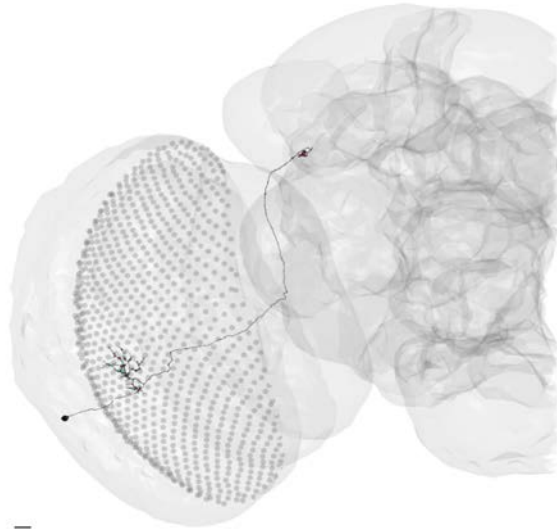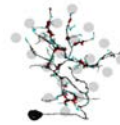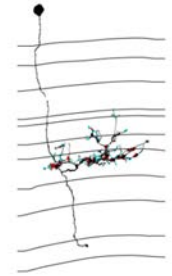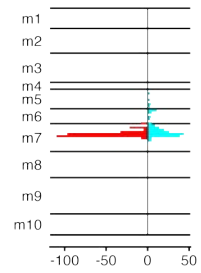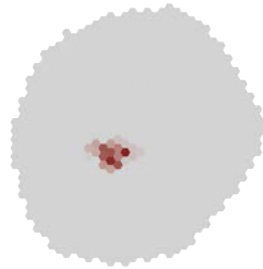

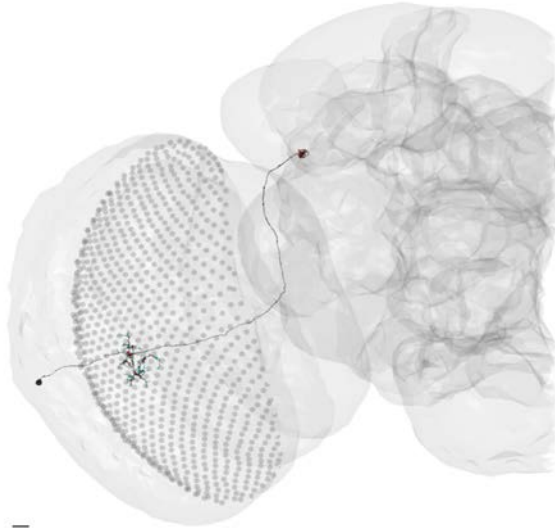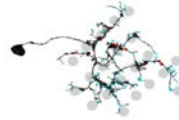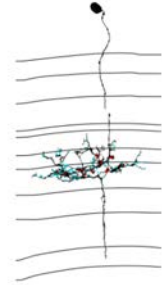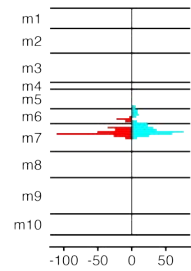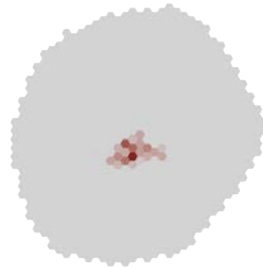

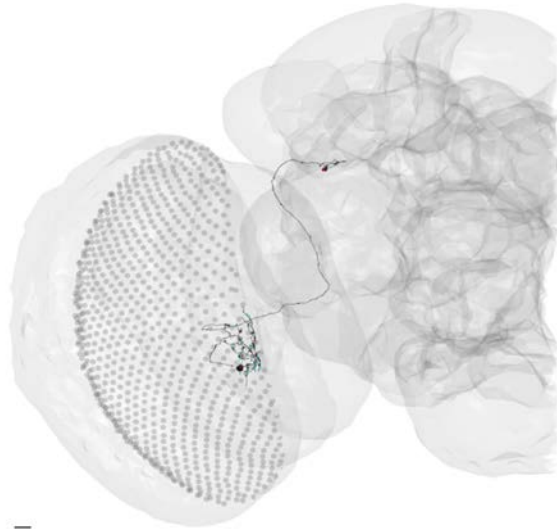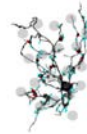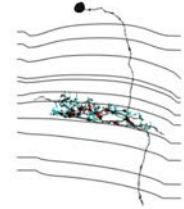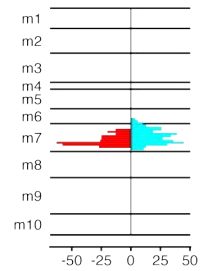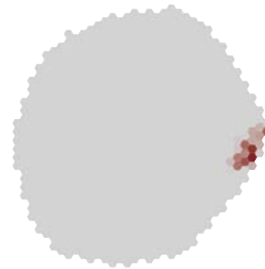

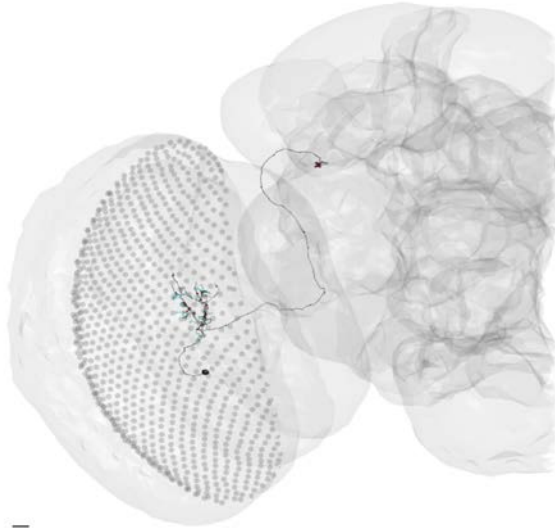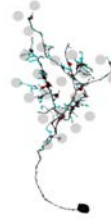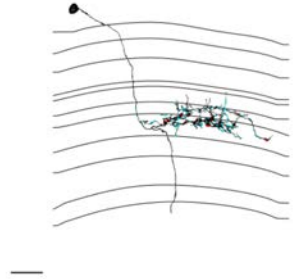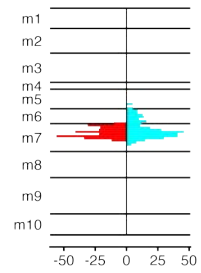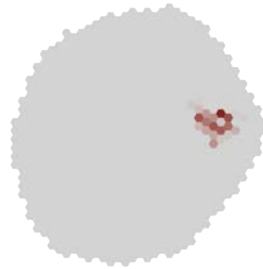

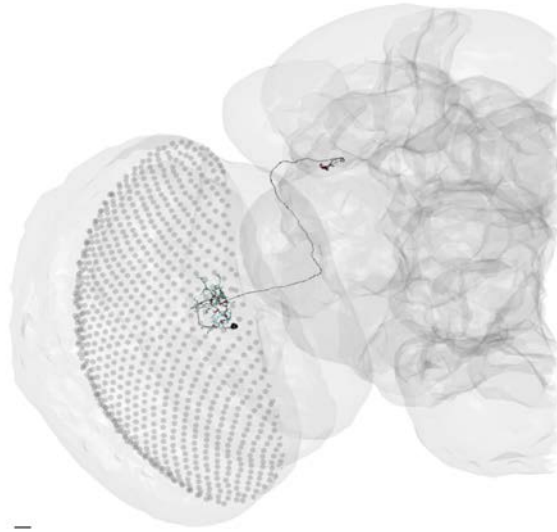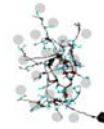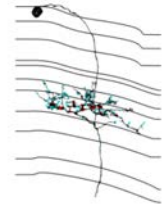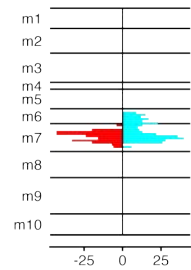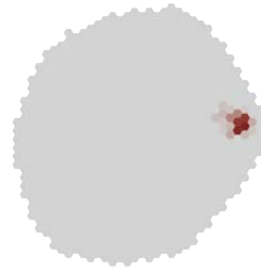

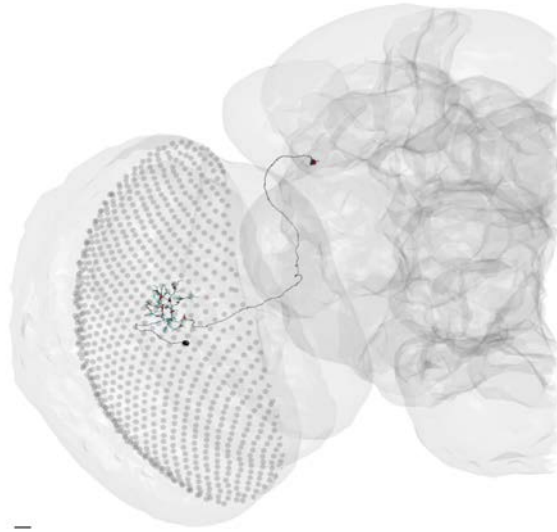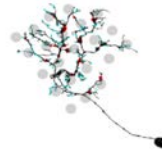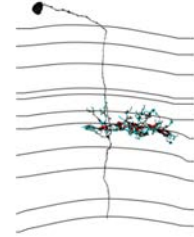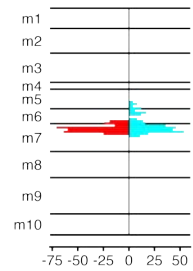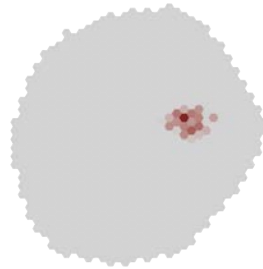

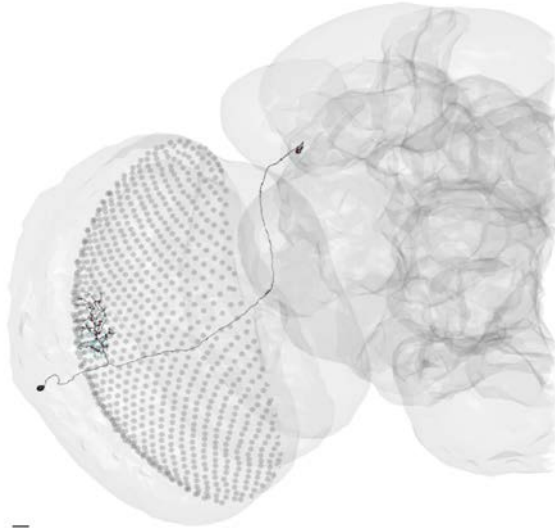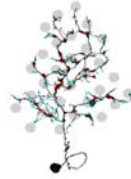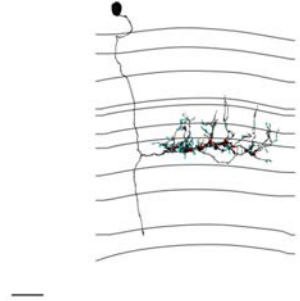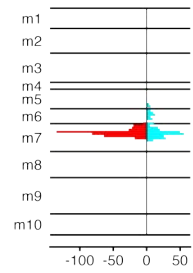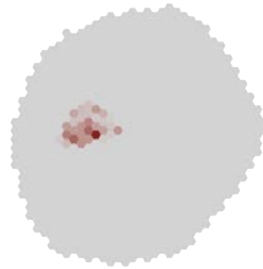

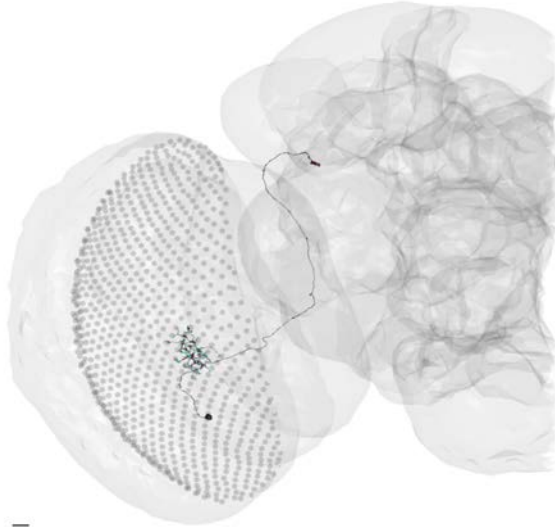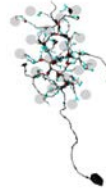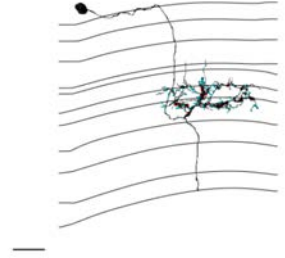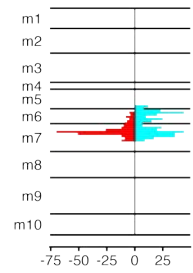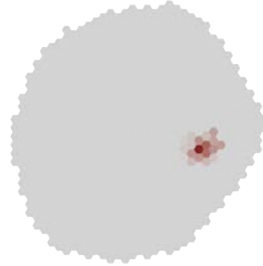

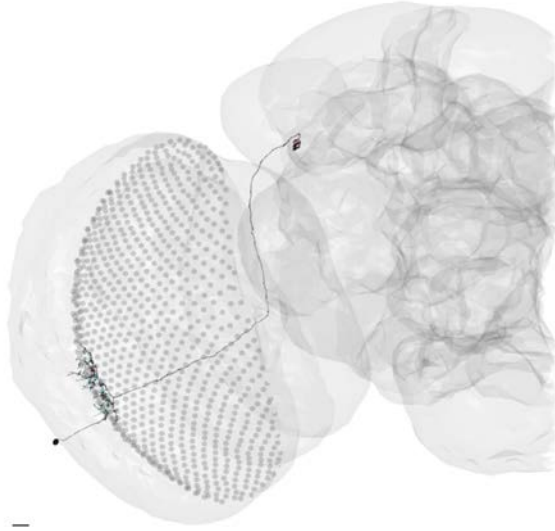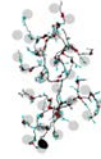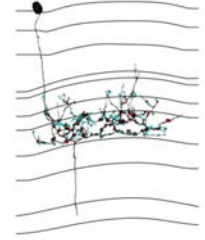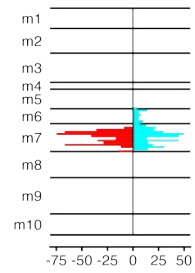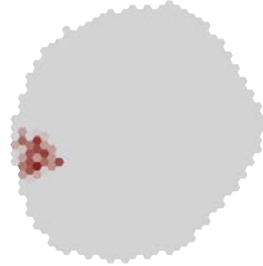

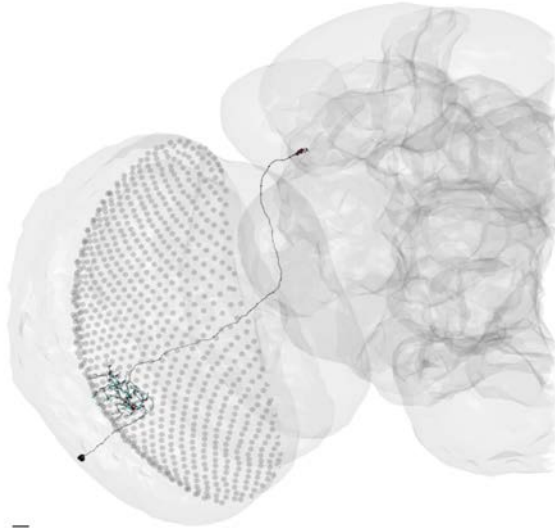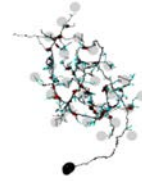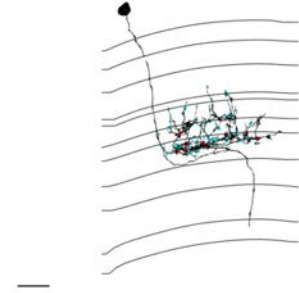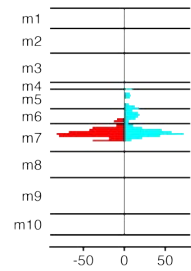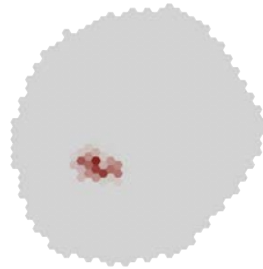

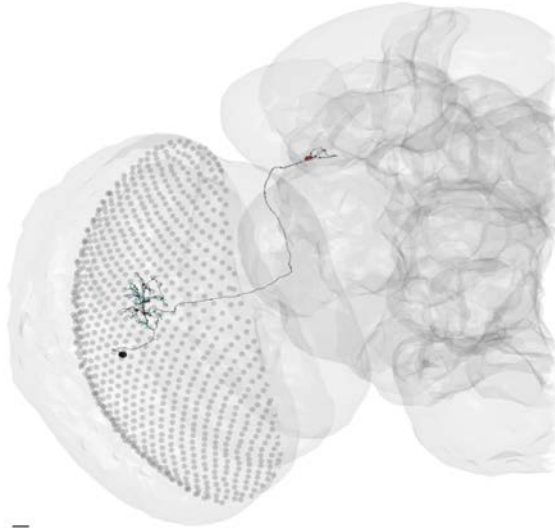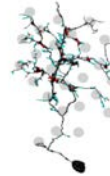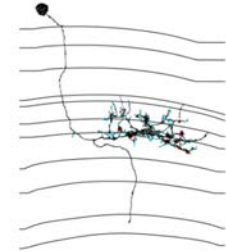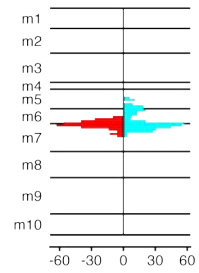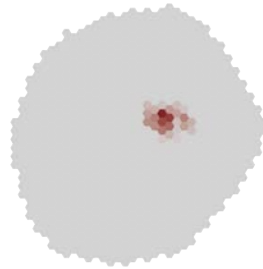

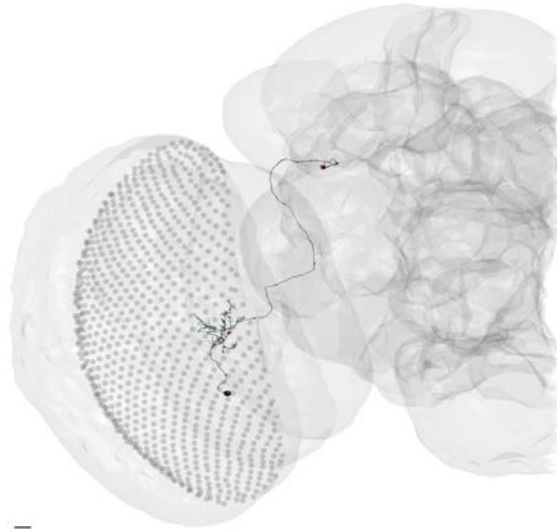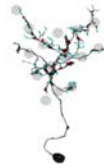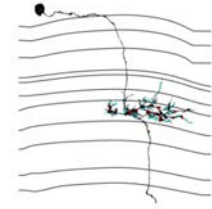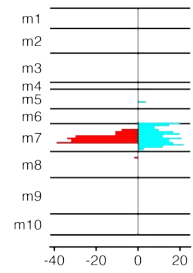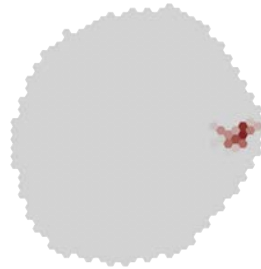

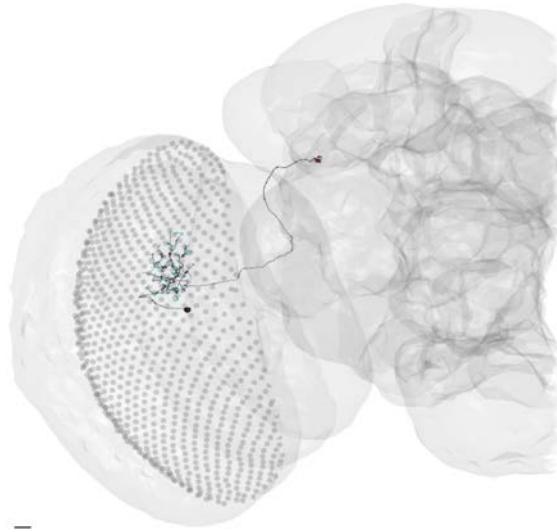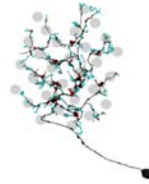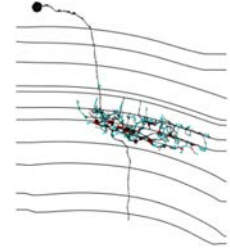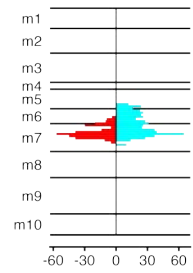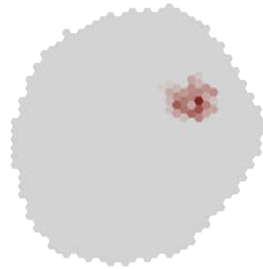

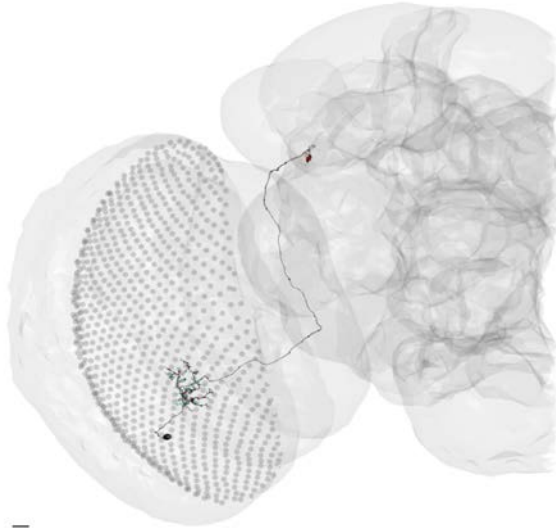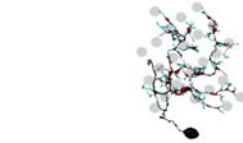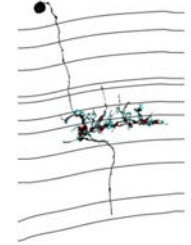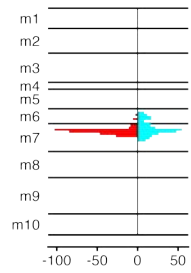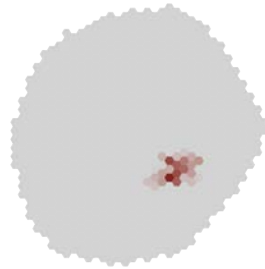

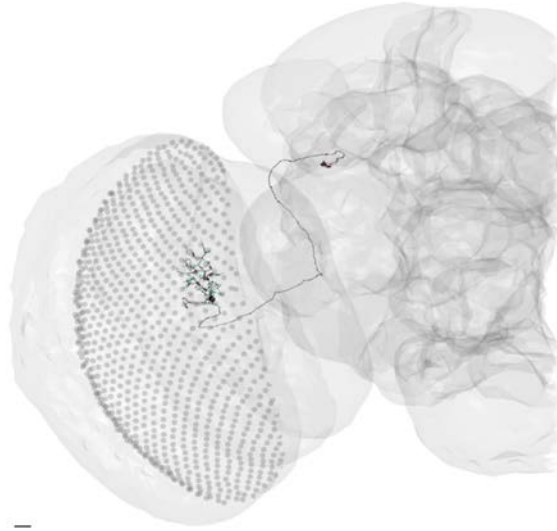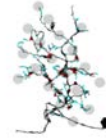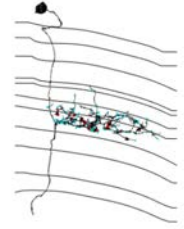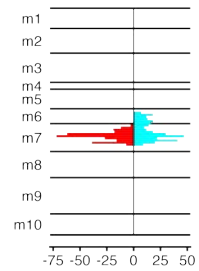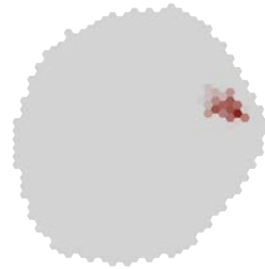

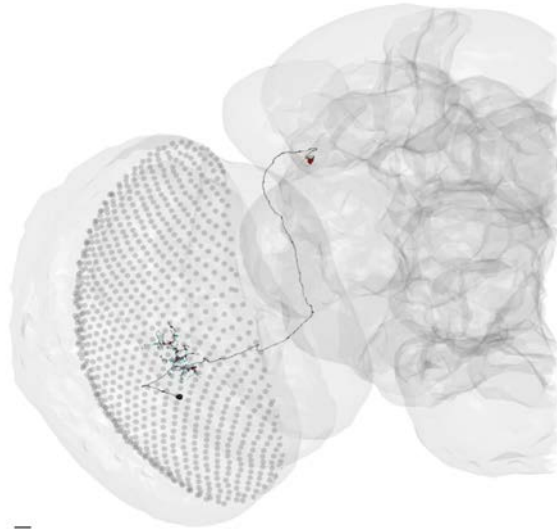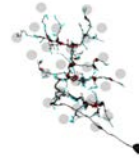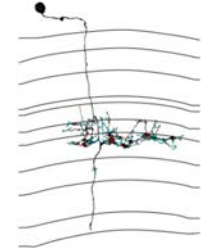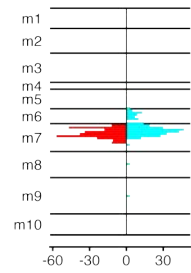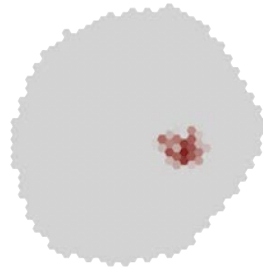

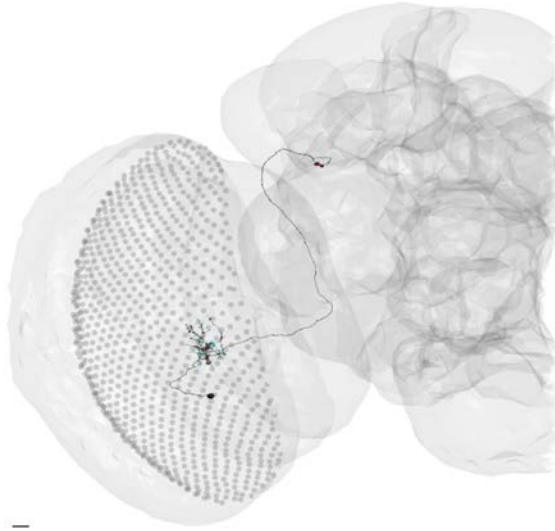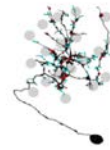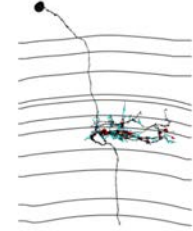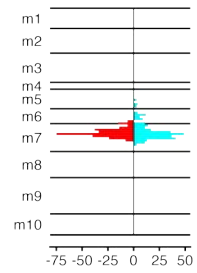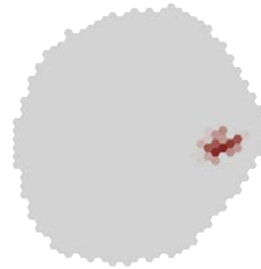

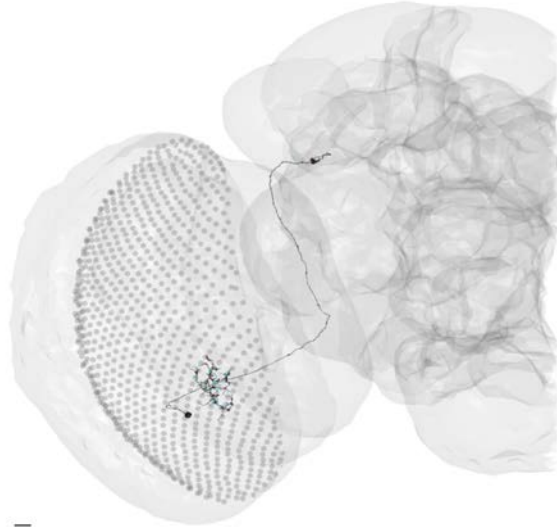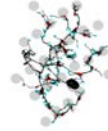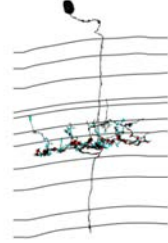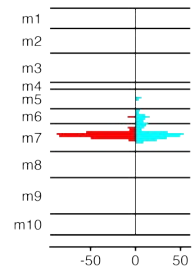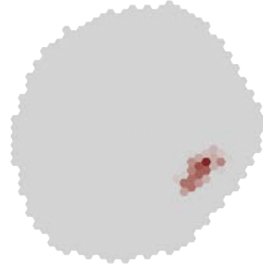

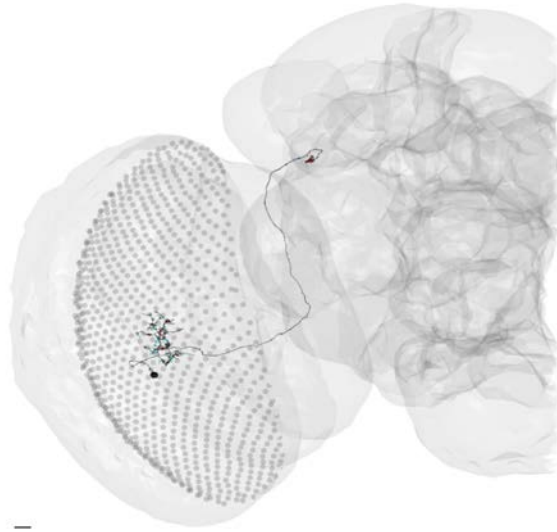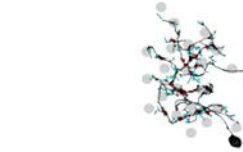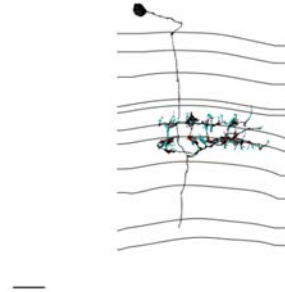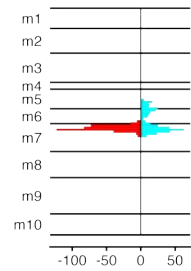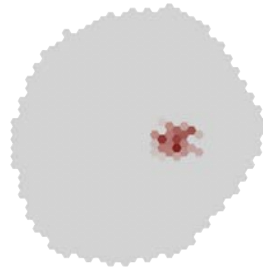

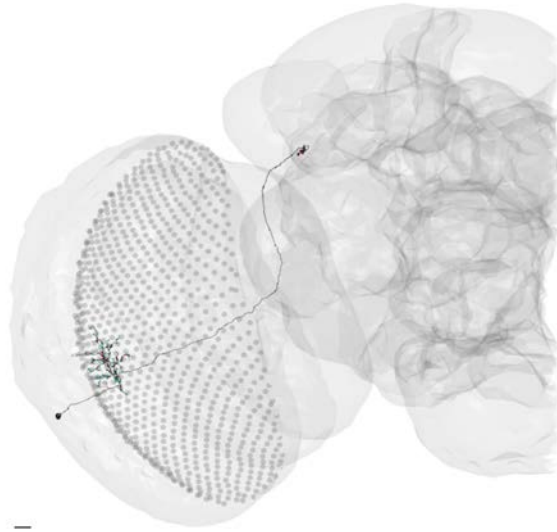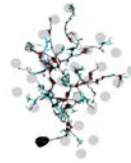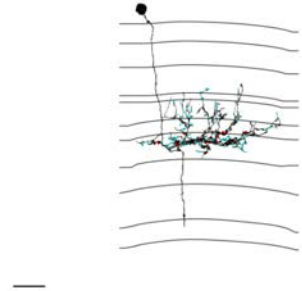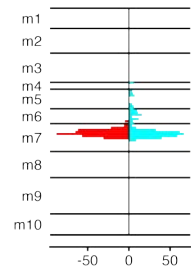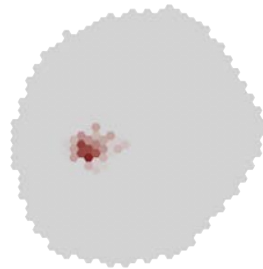

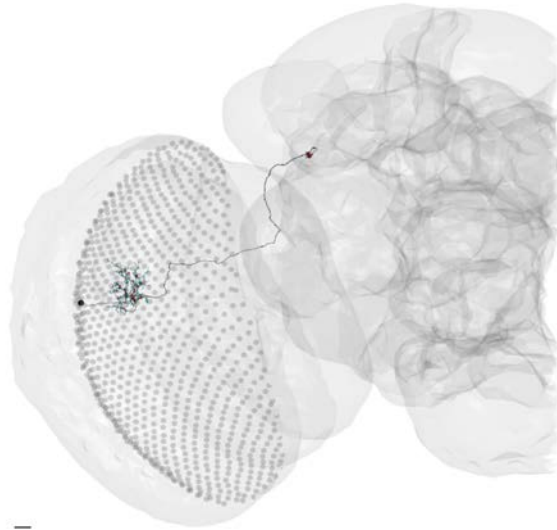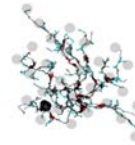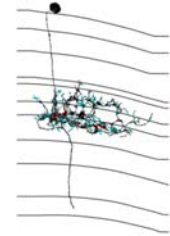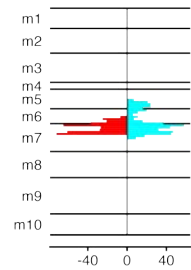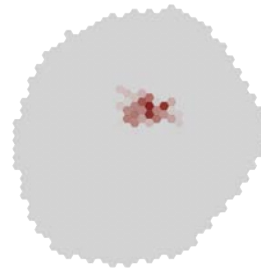

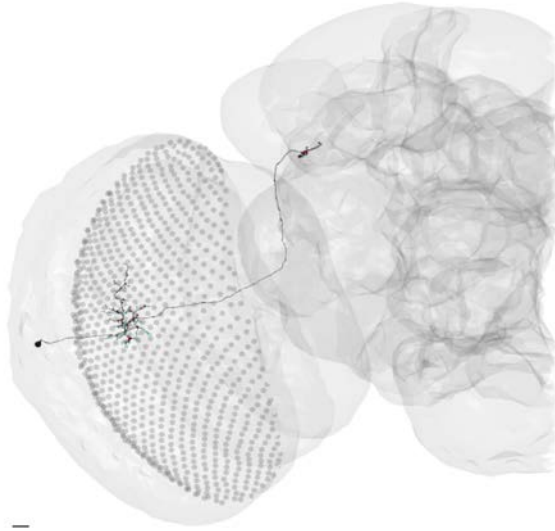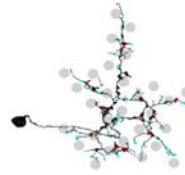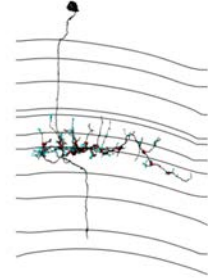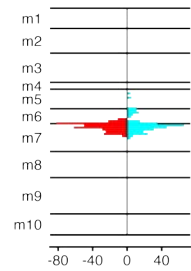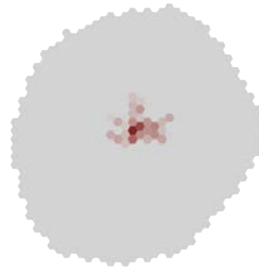

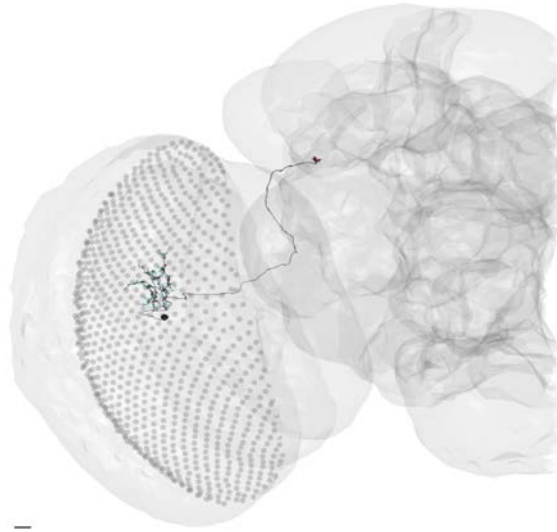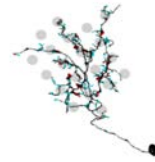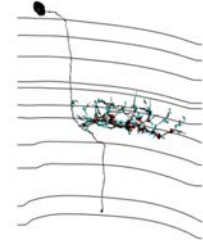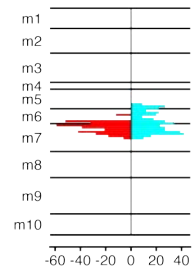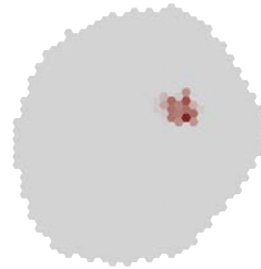

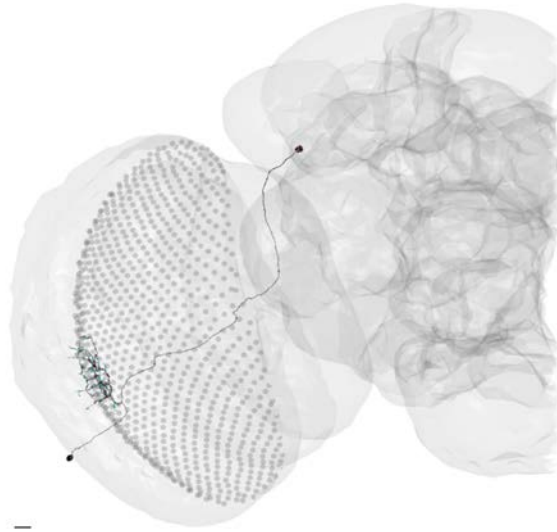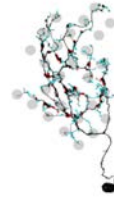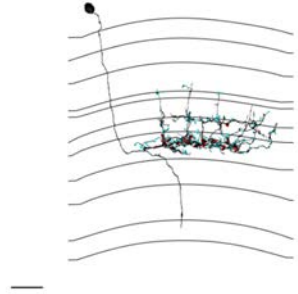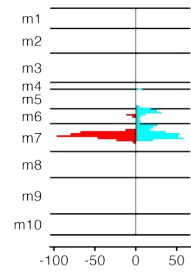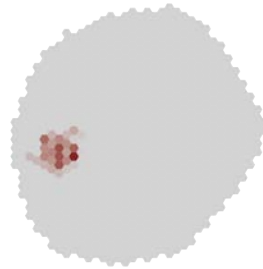

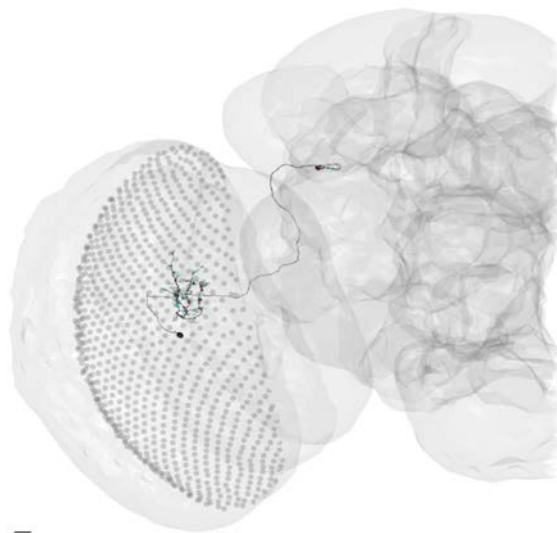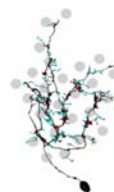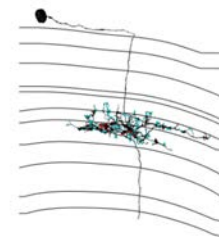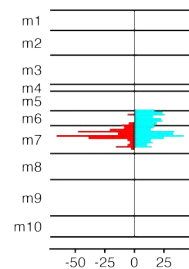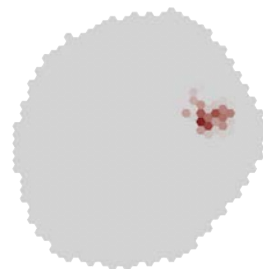

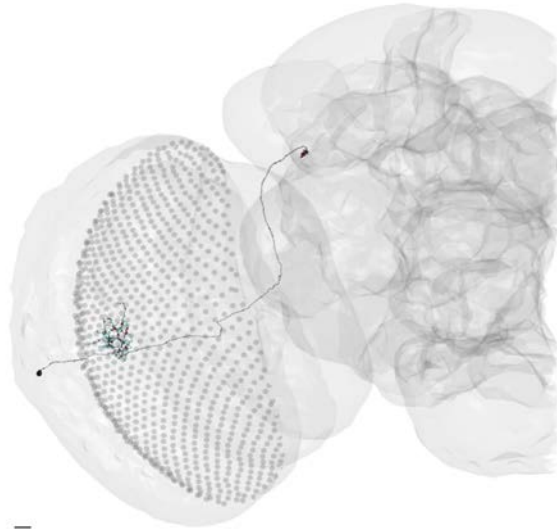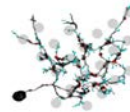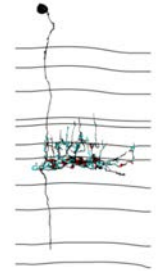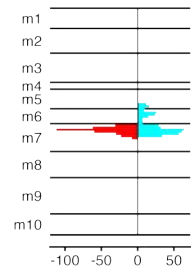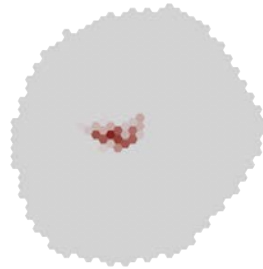

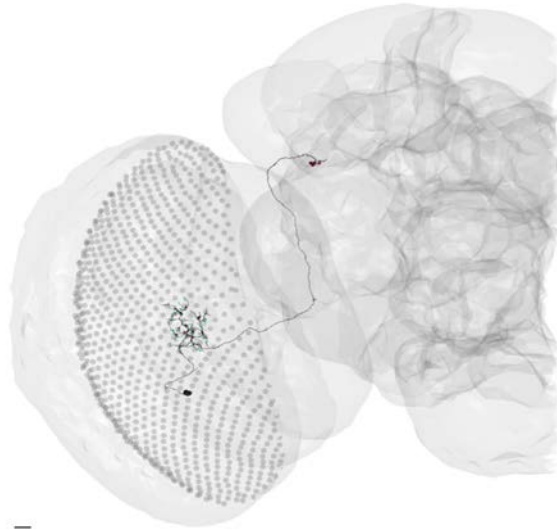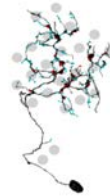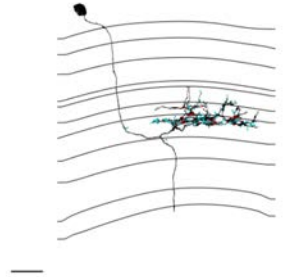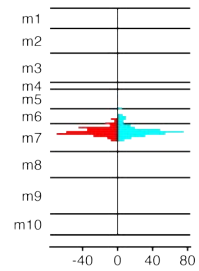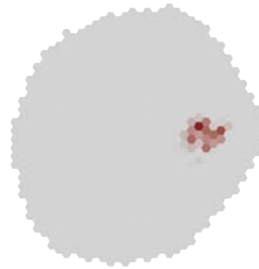

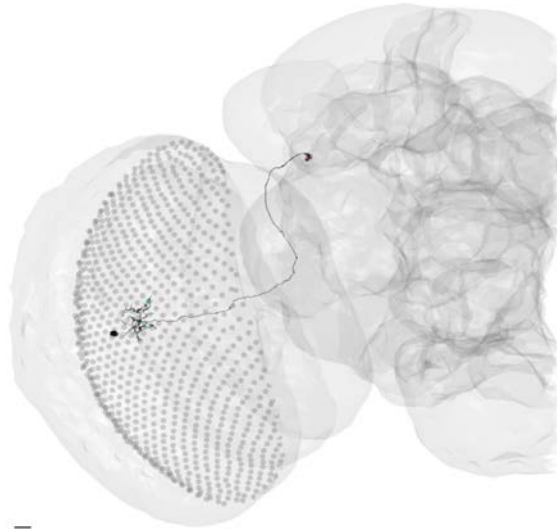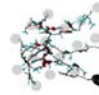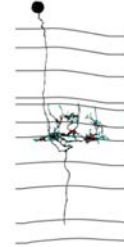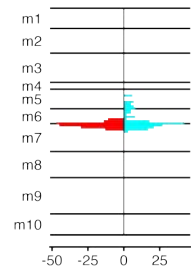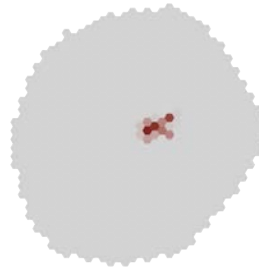

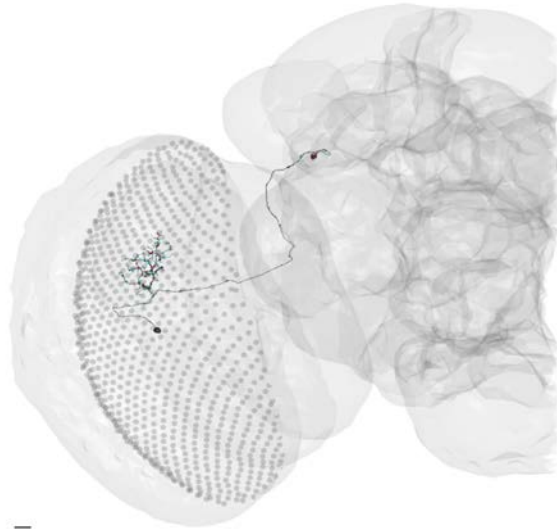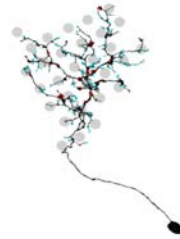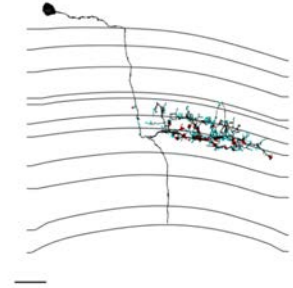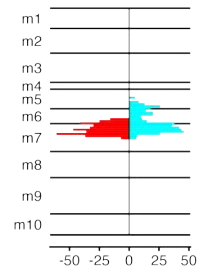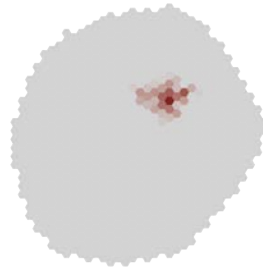

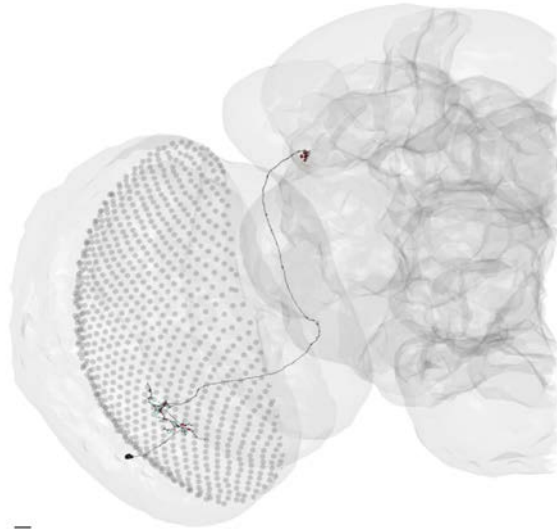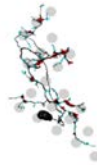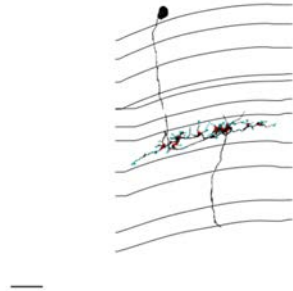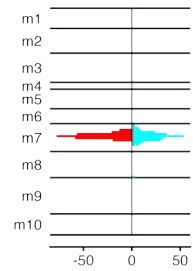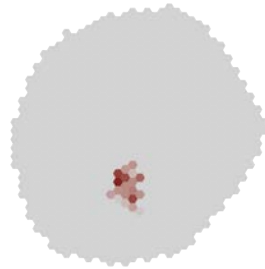

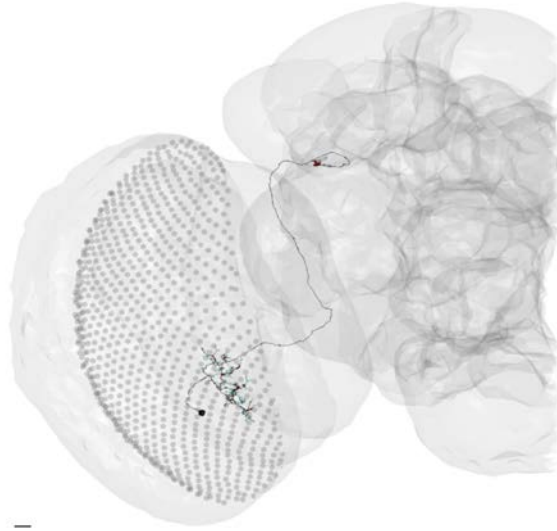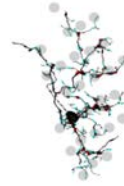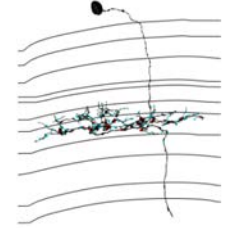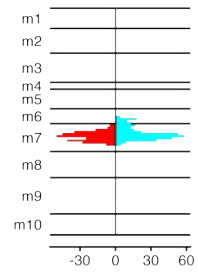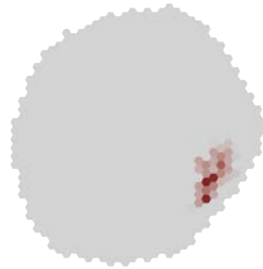

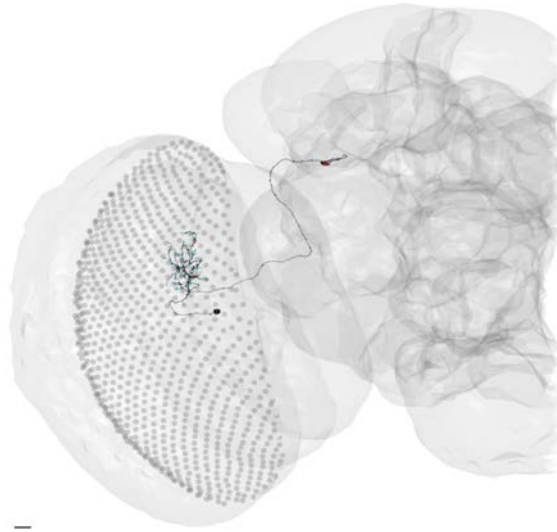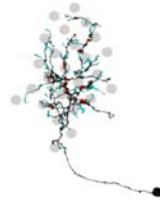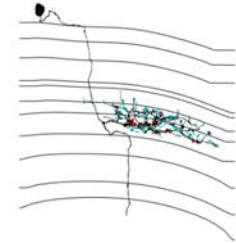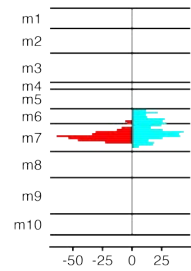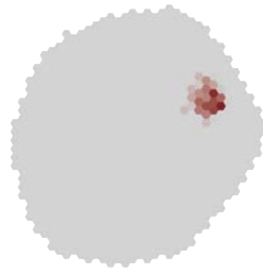

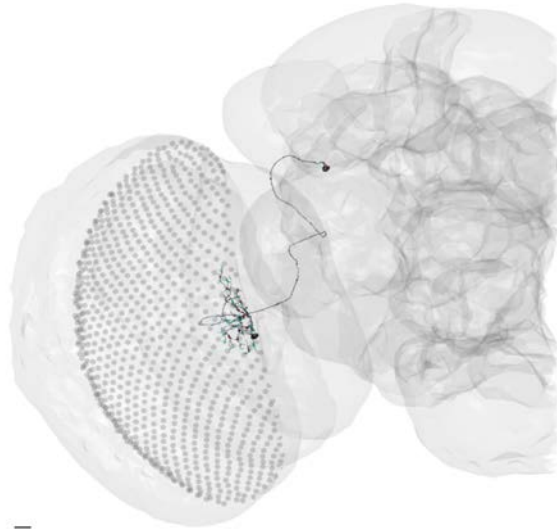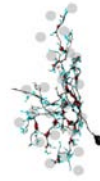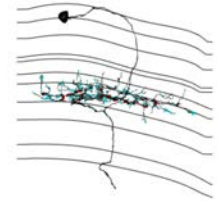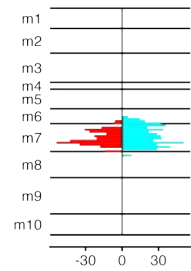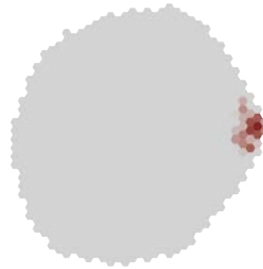

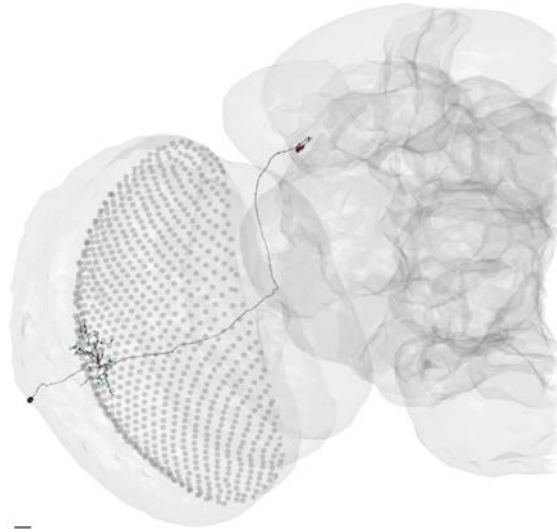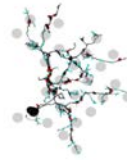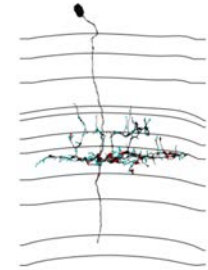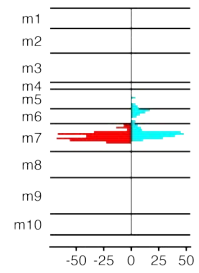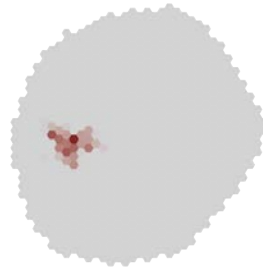

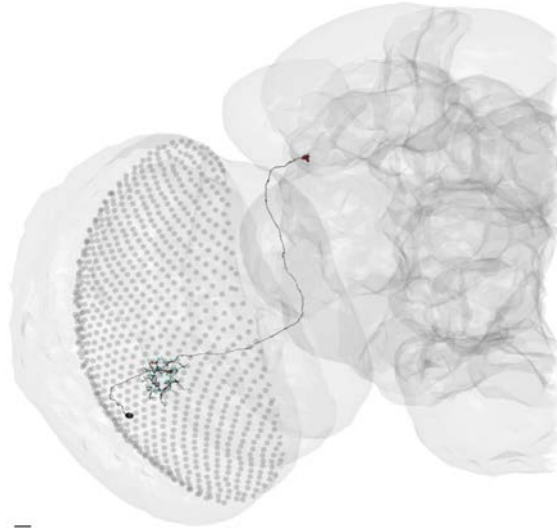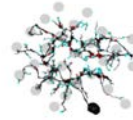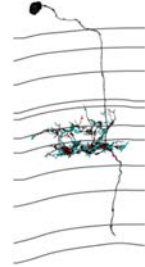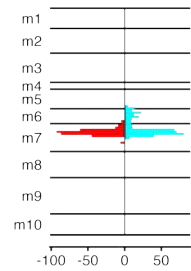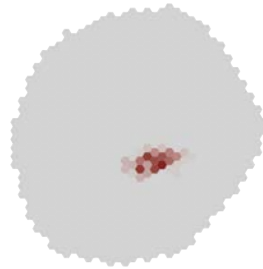

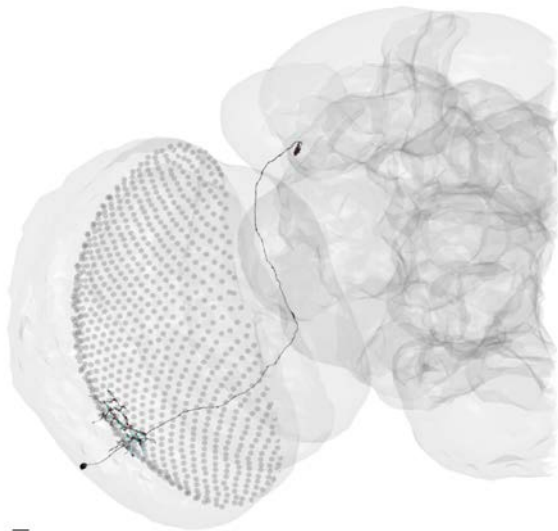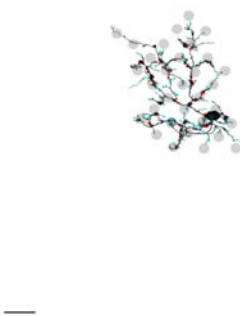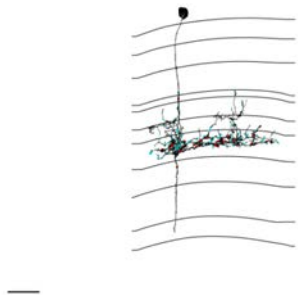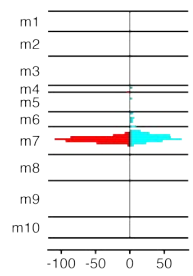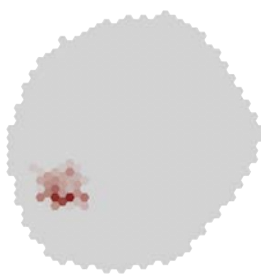

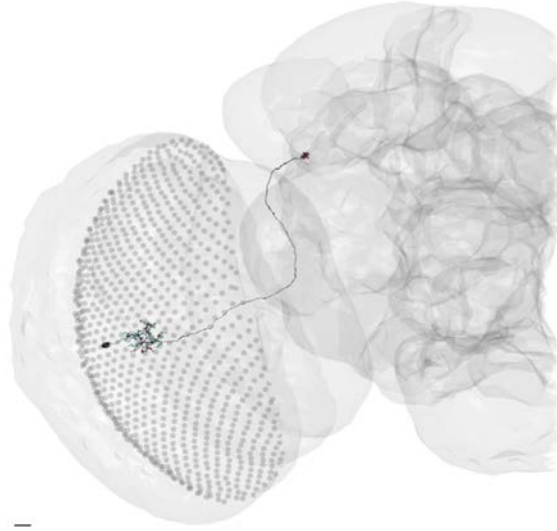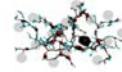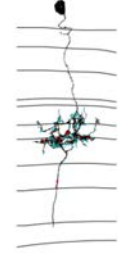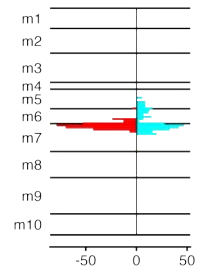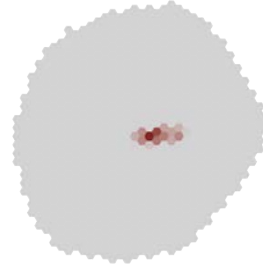

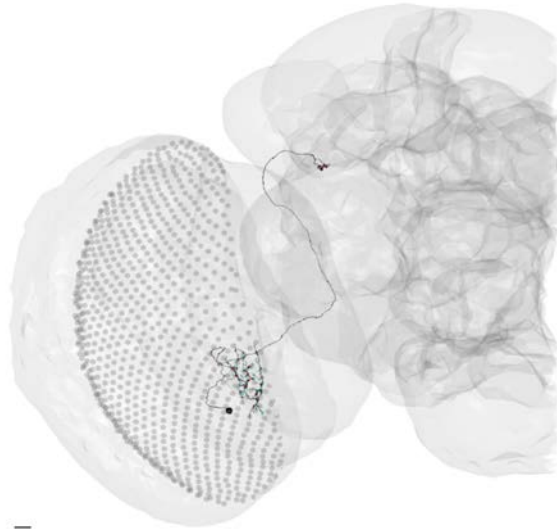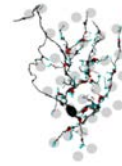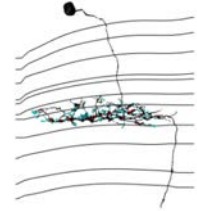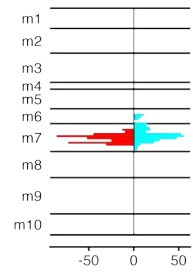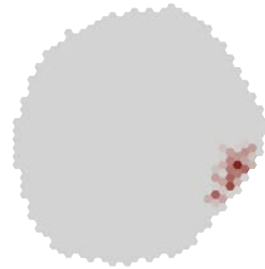

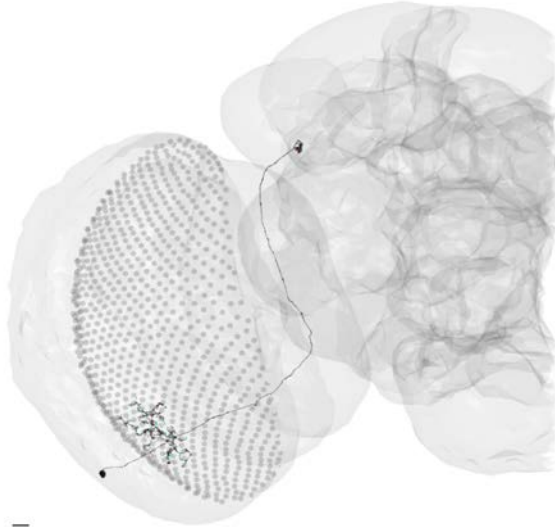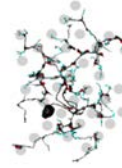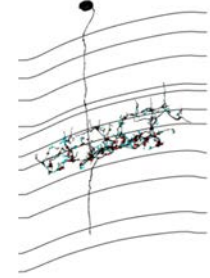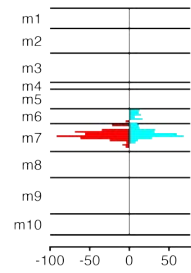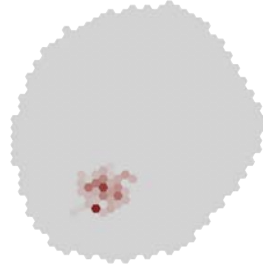

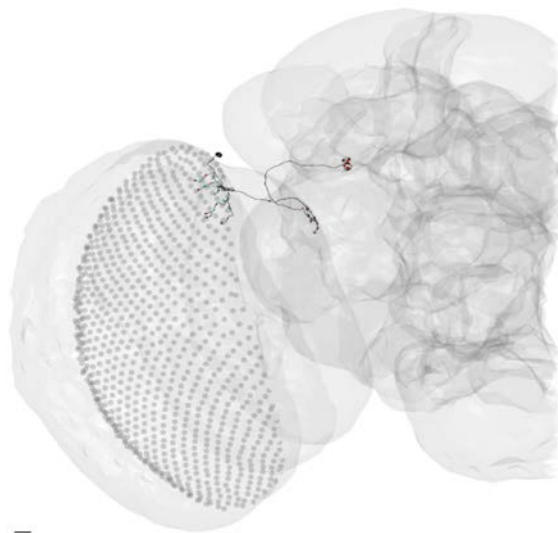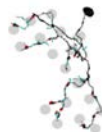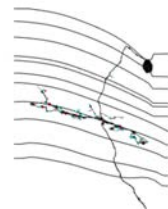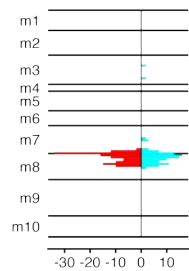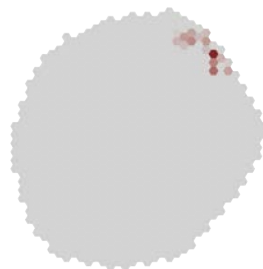

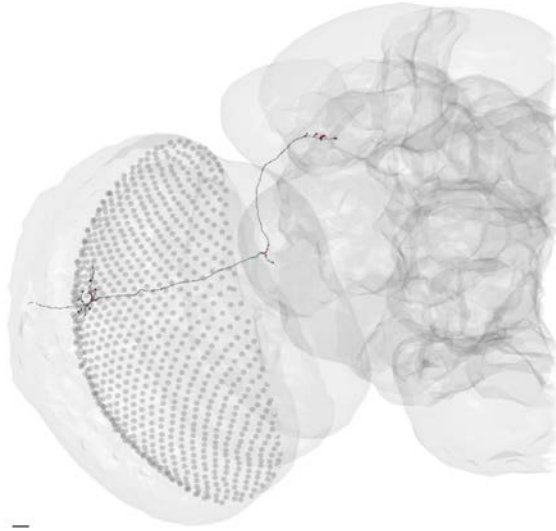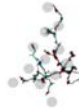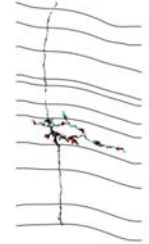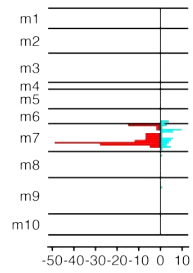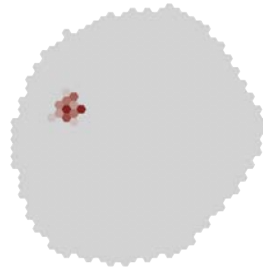

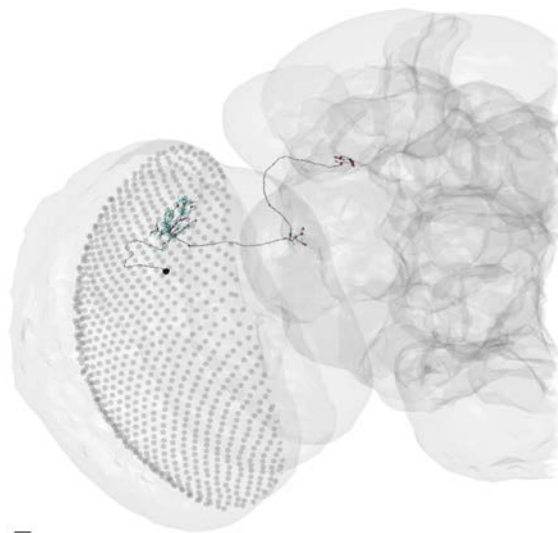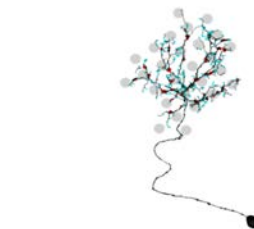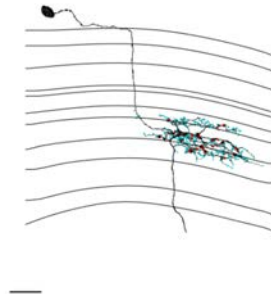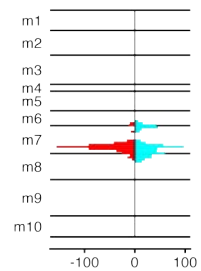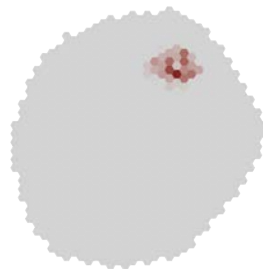

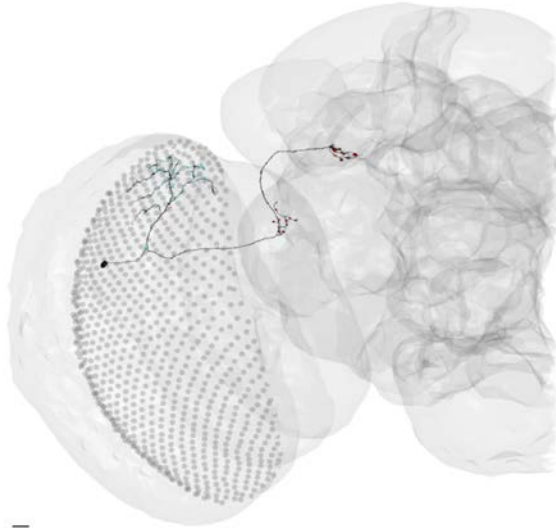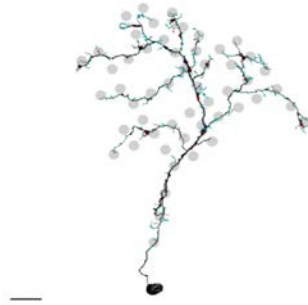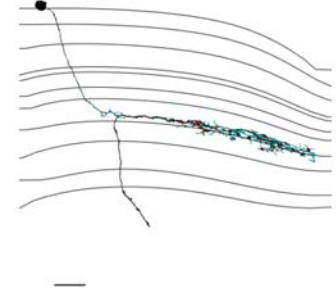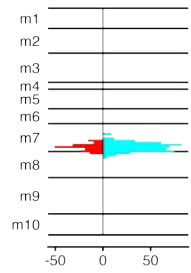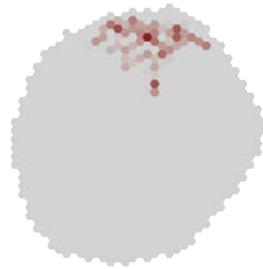

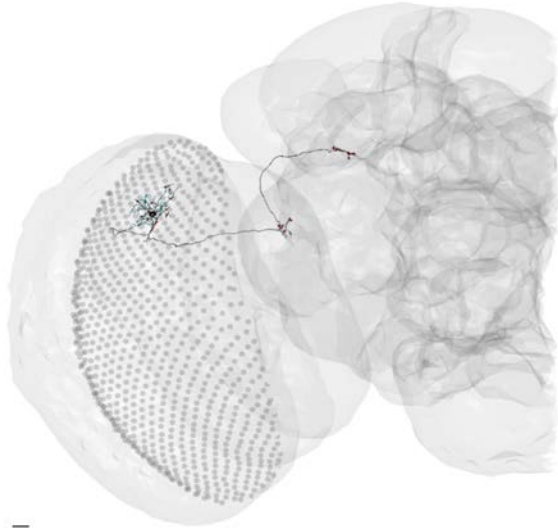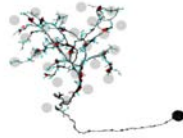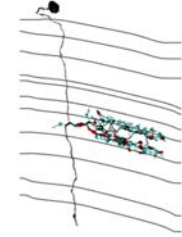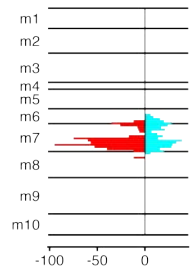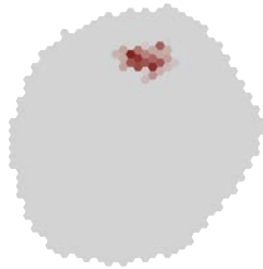

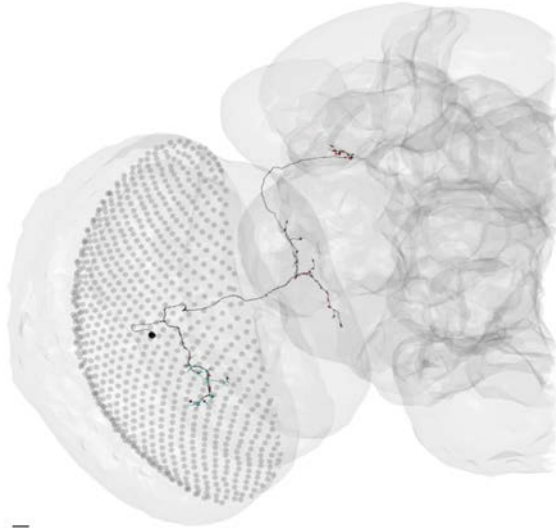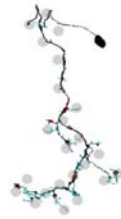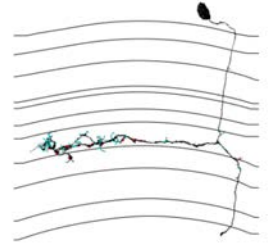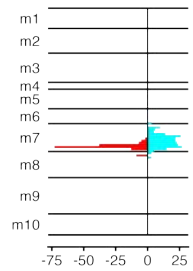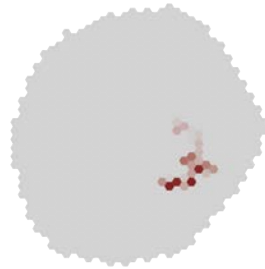

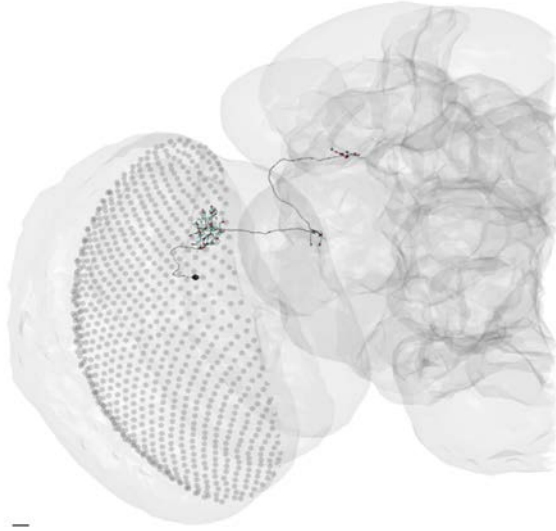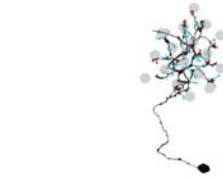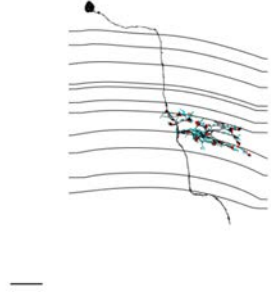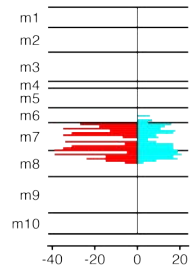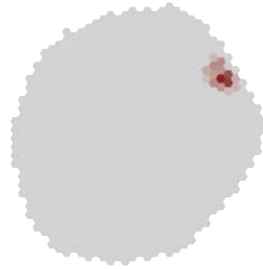

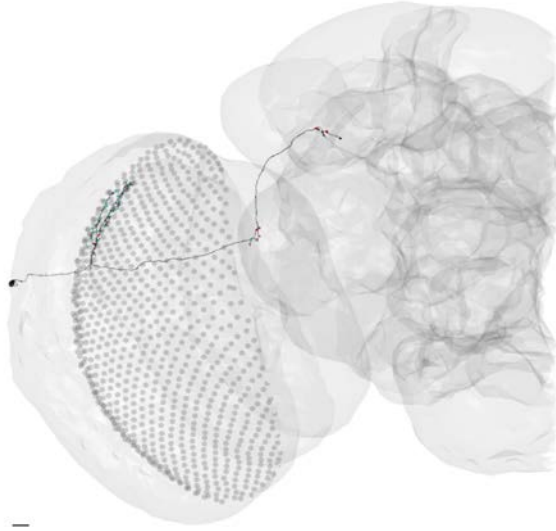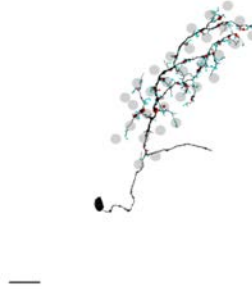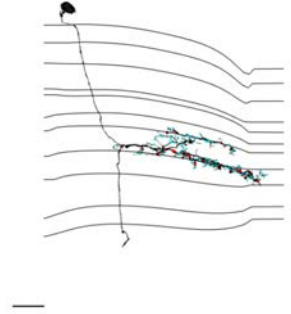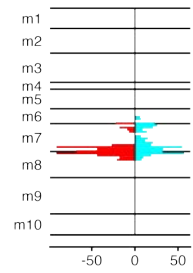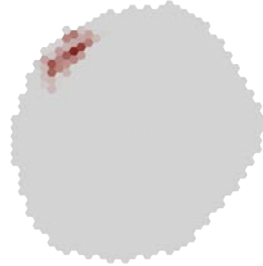

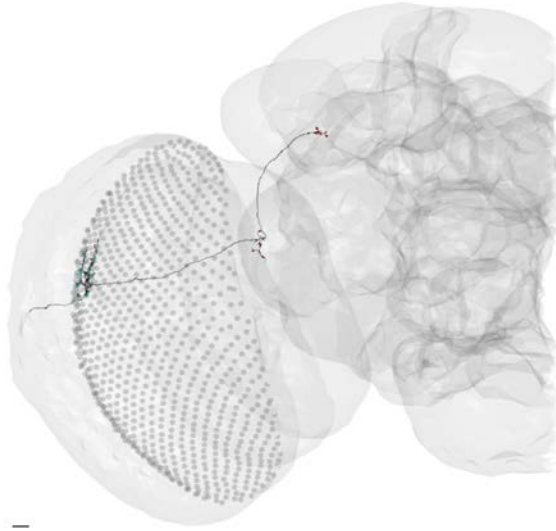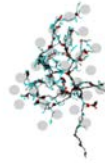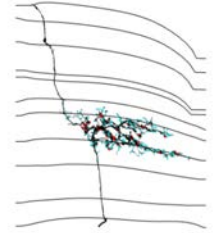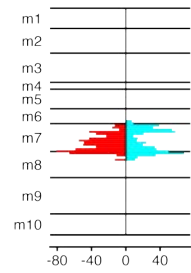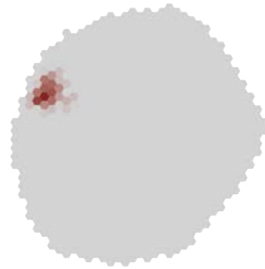

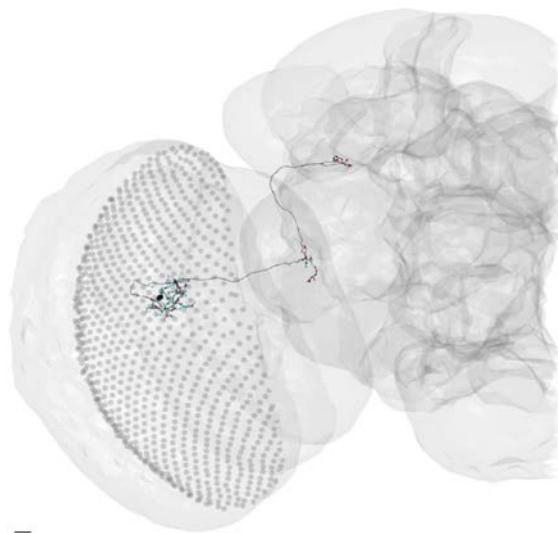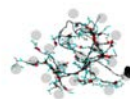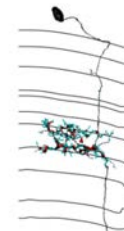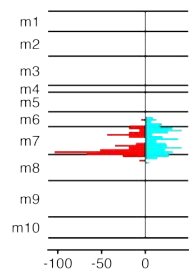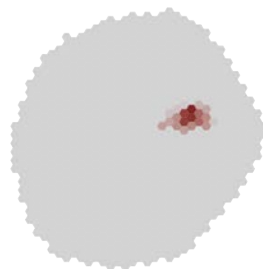

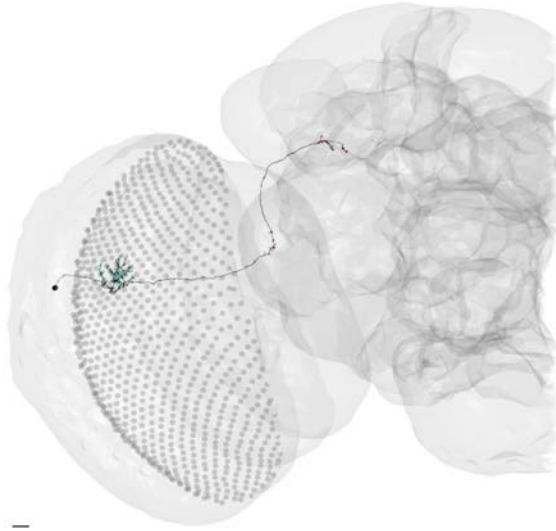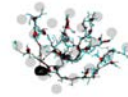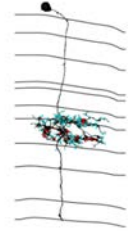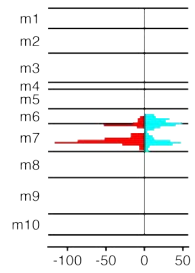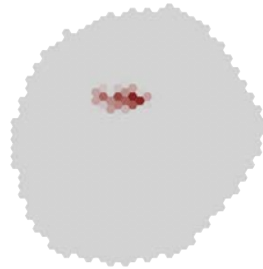

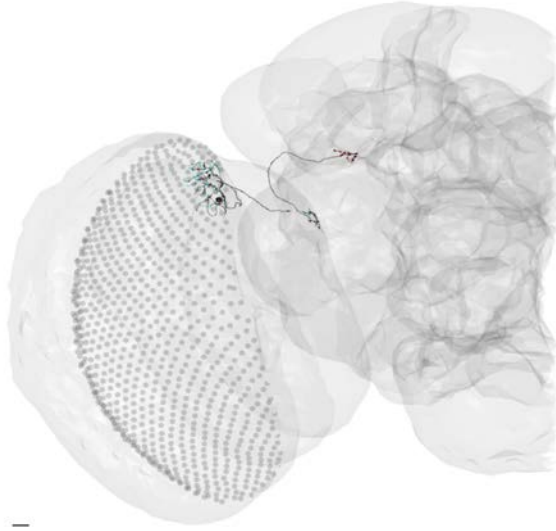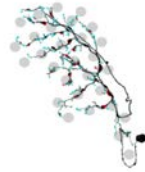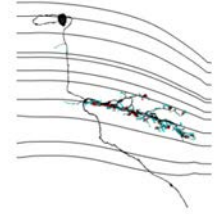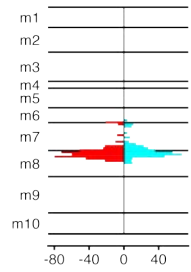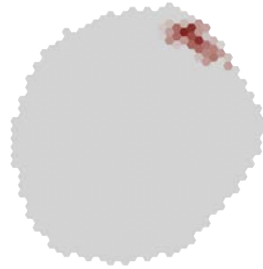

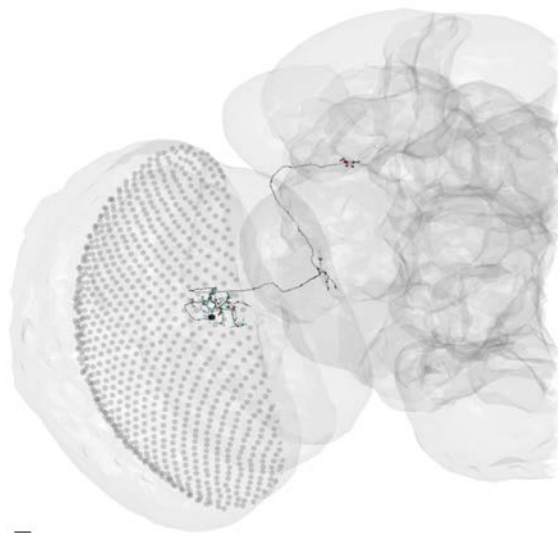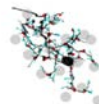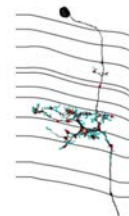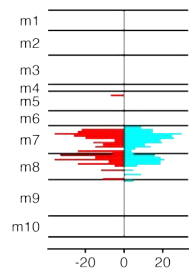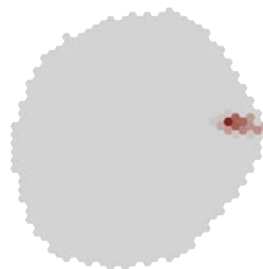

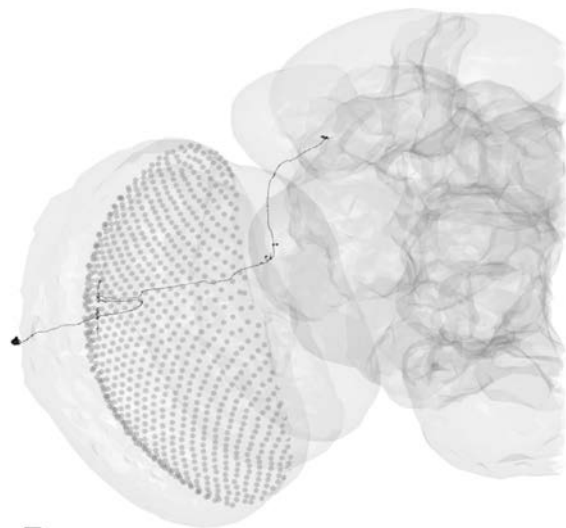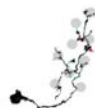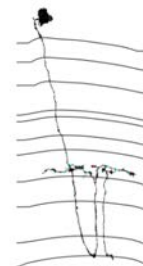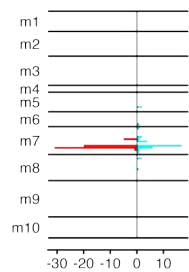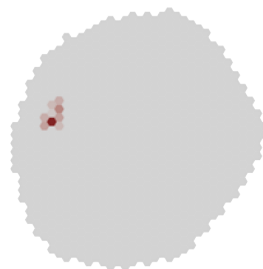

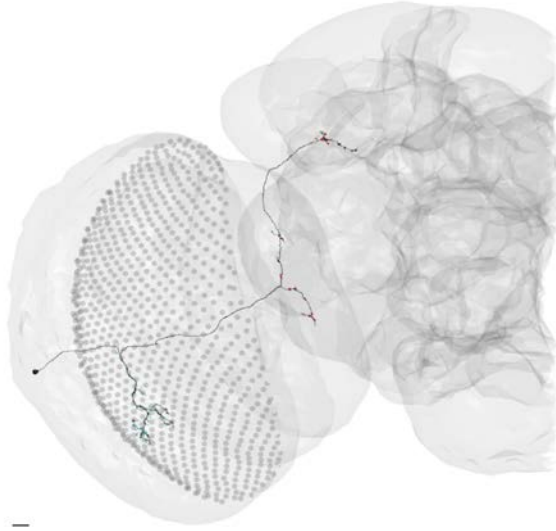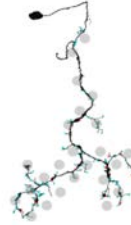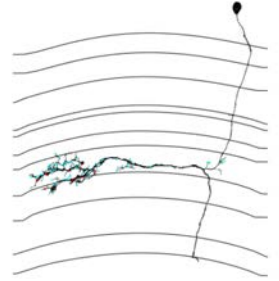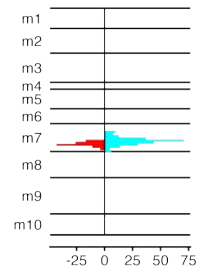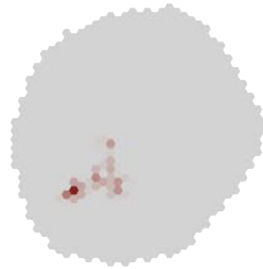

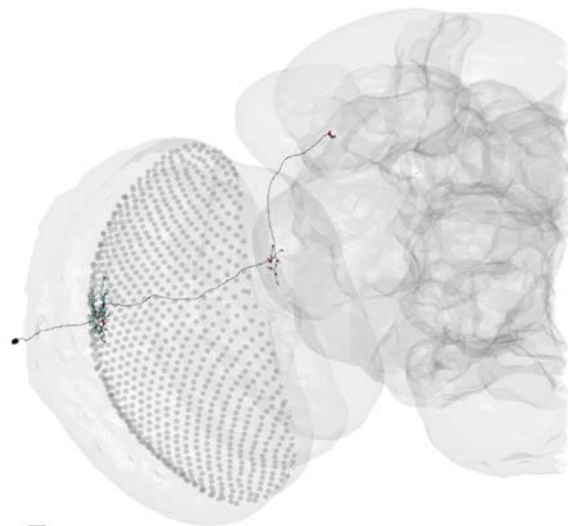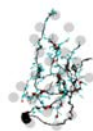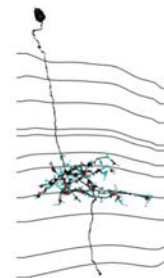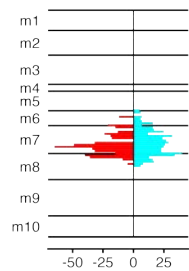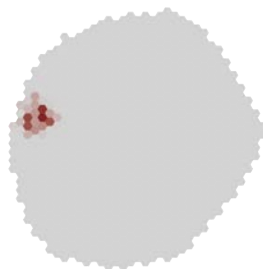

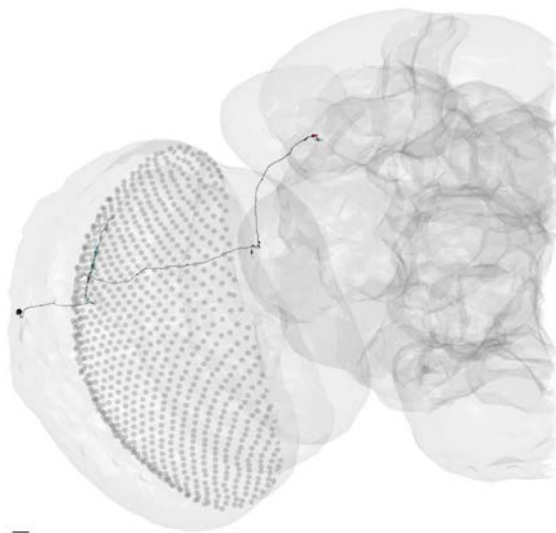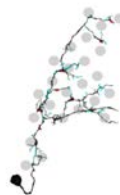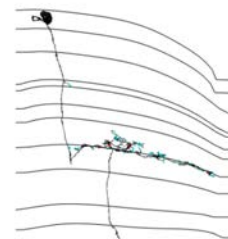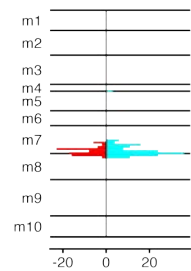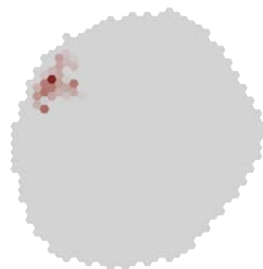

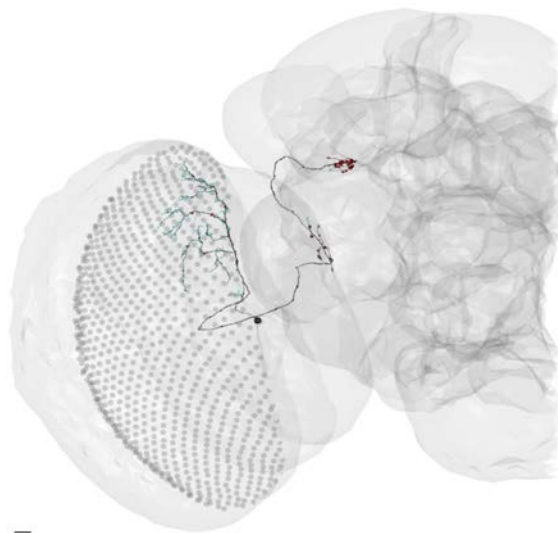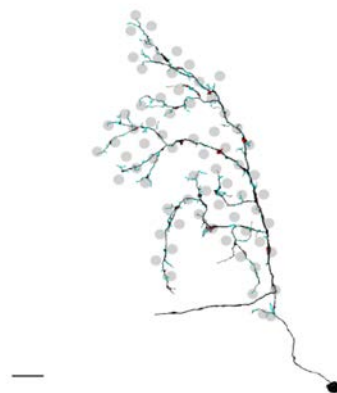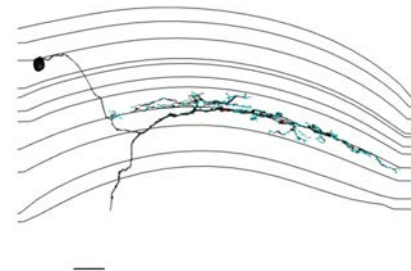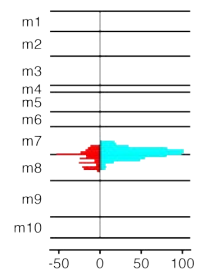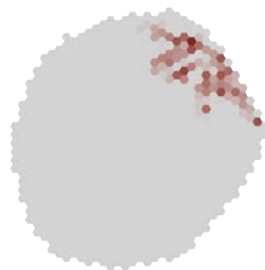

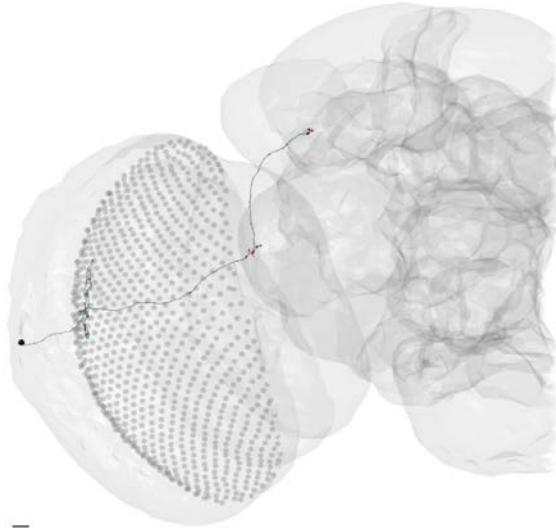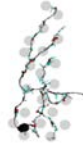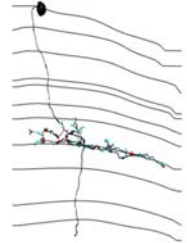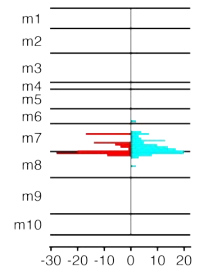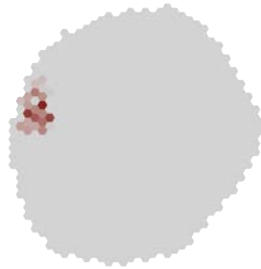

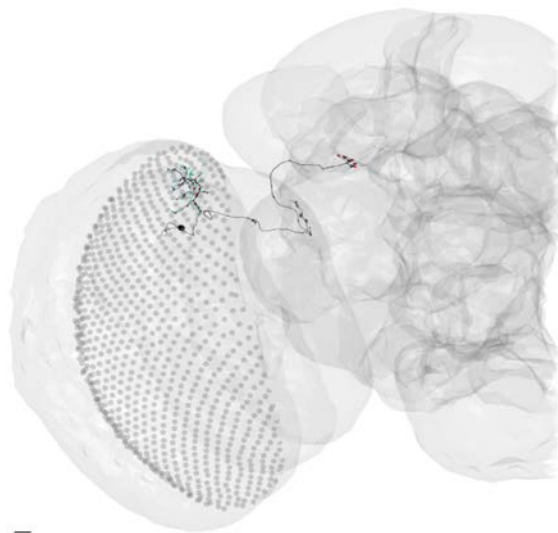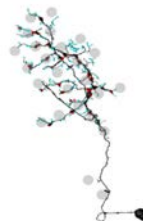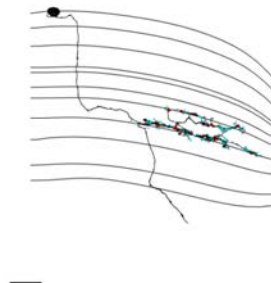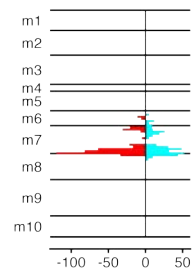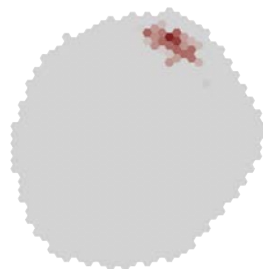

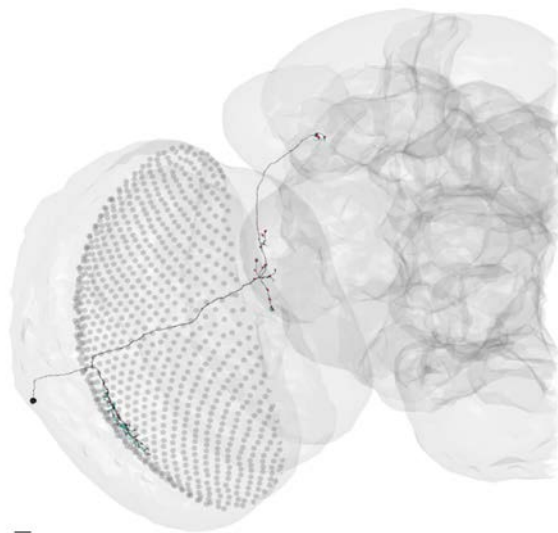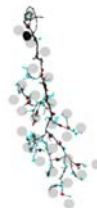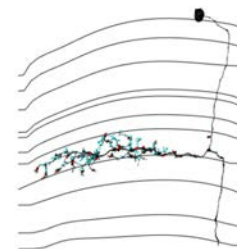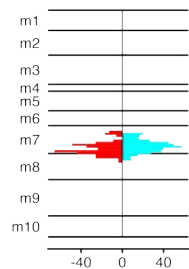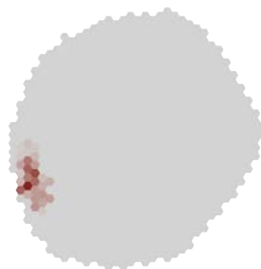

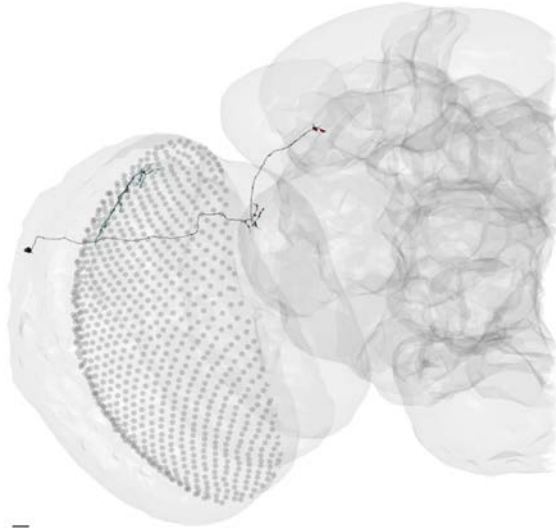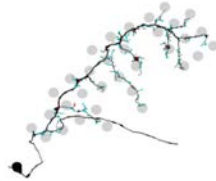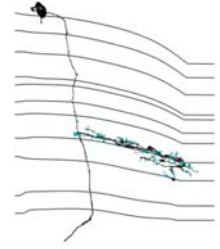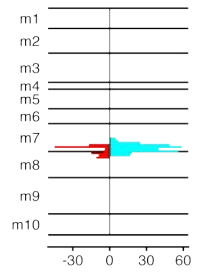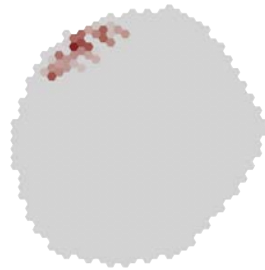

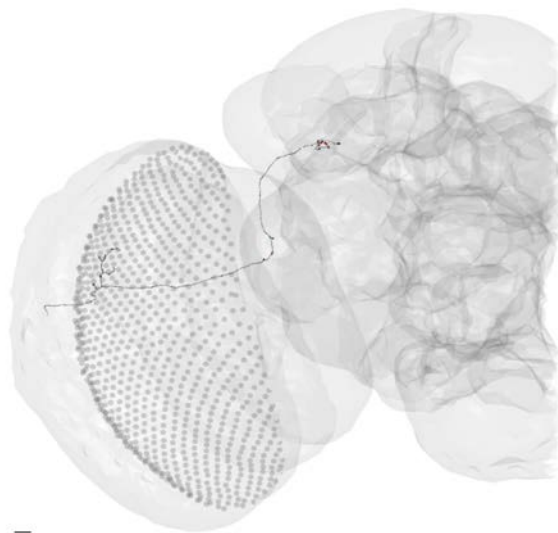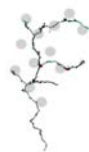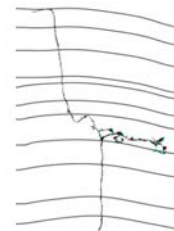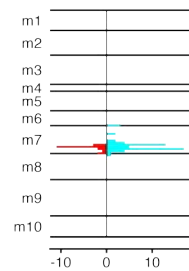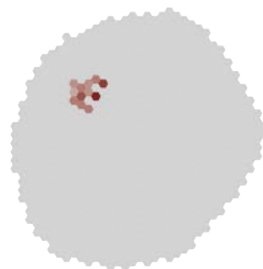

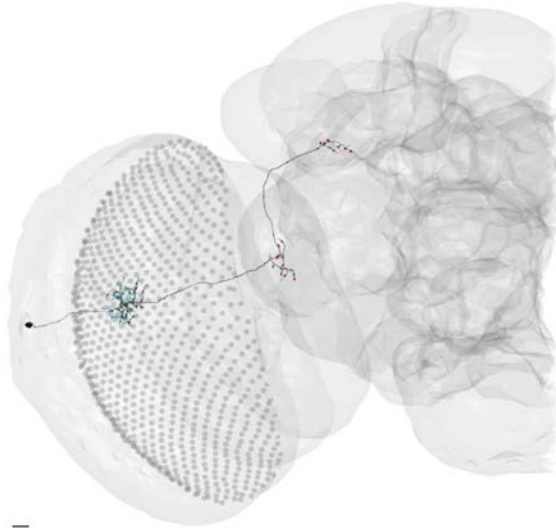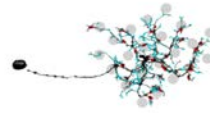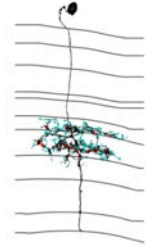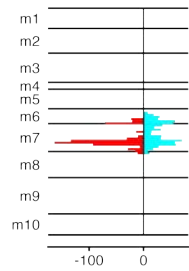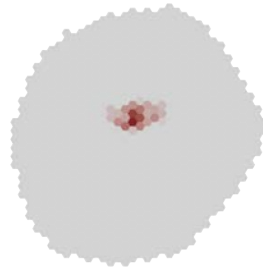

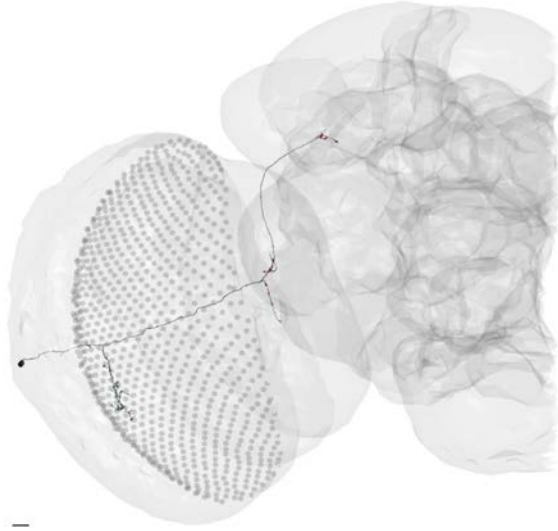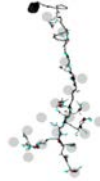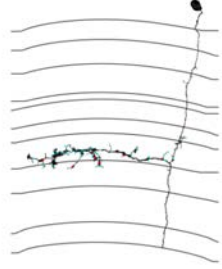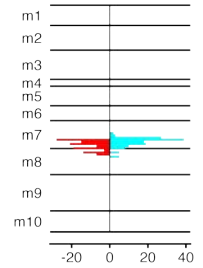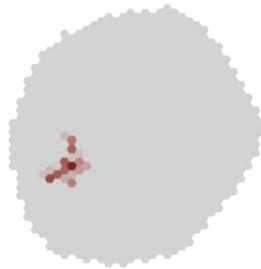

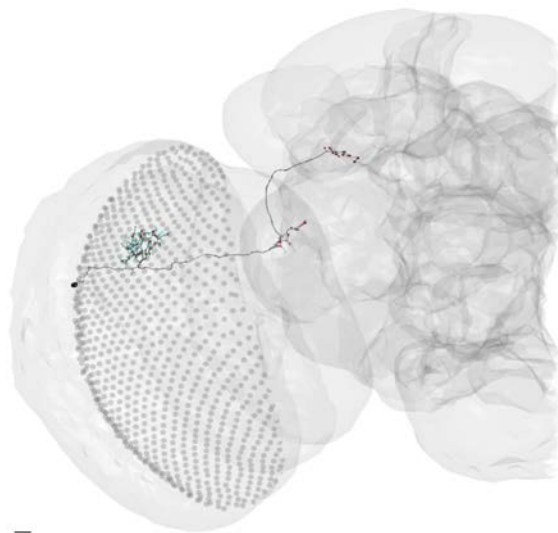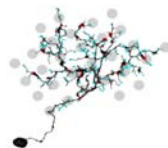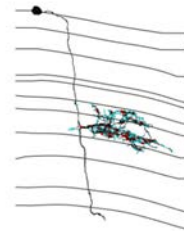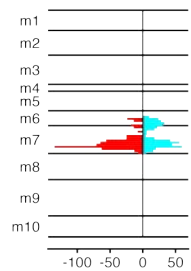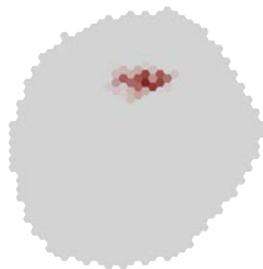

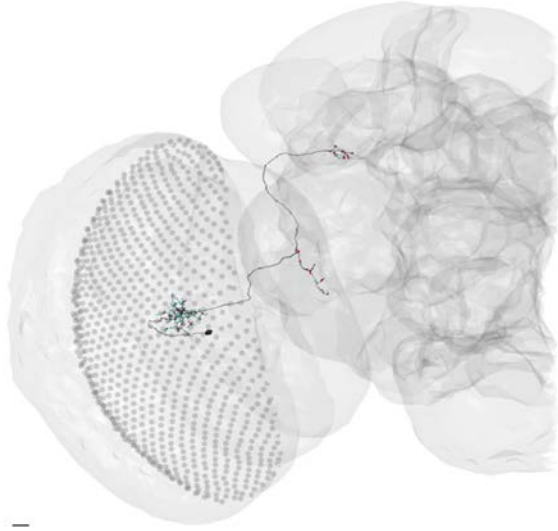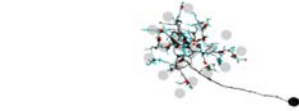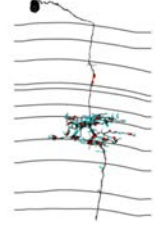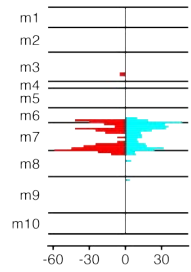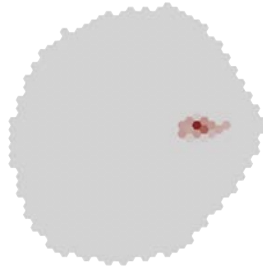

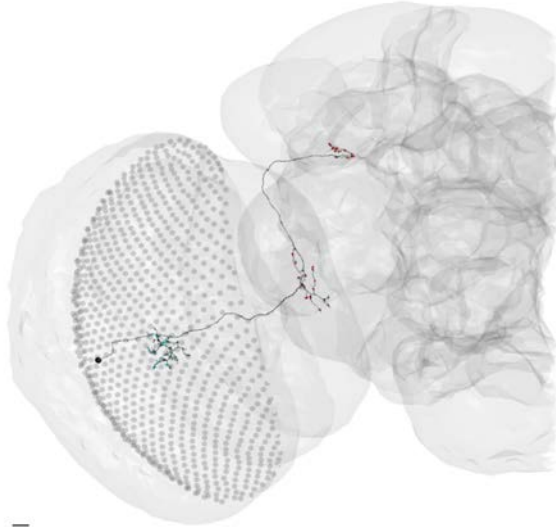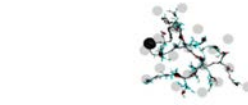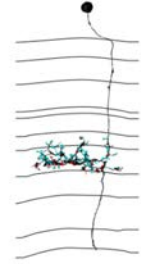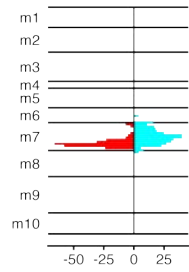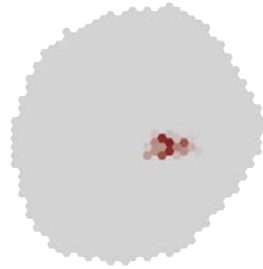

MeTu4a\_720575940624728551

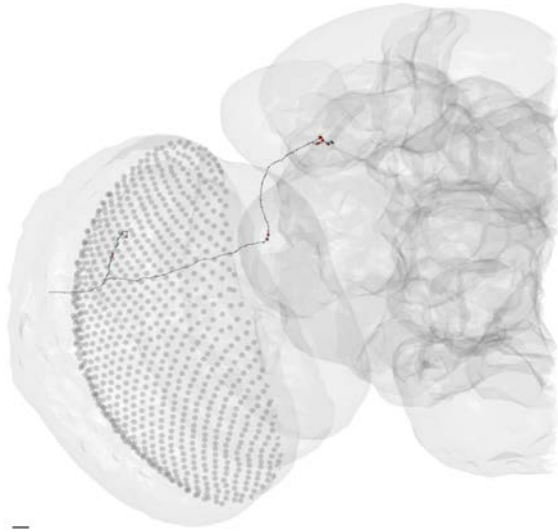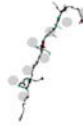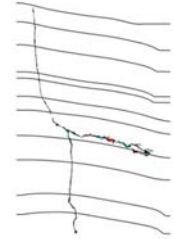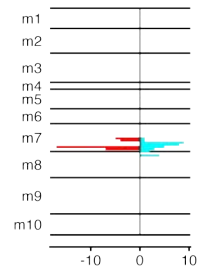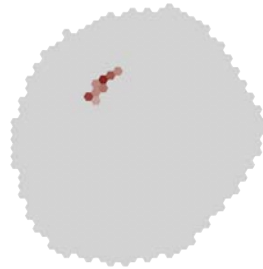

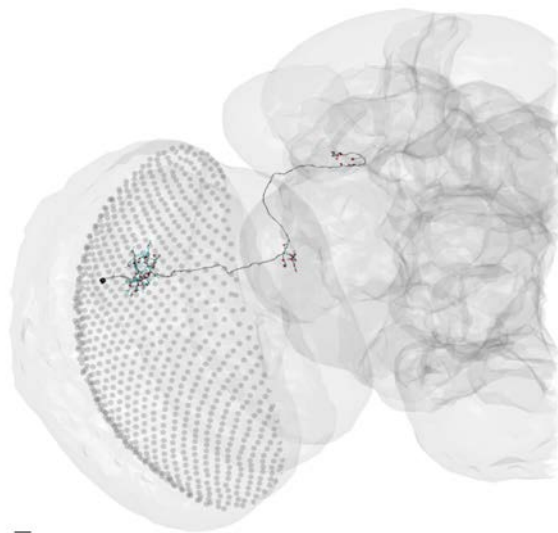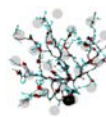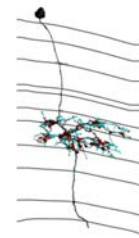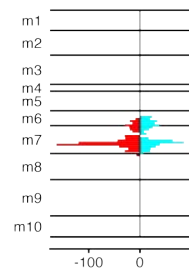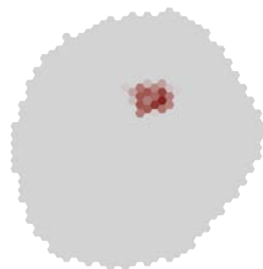

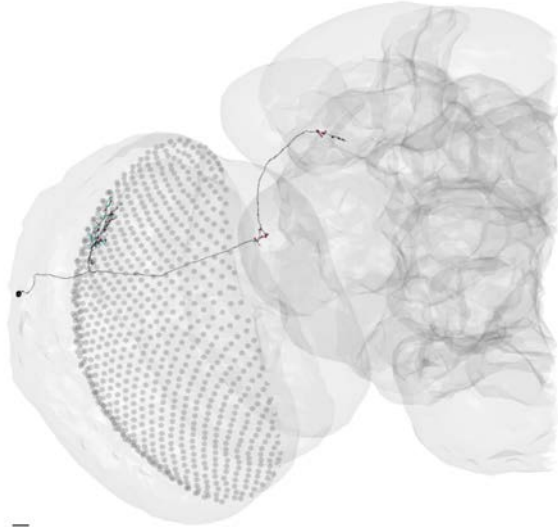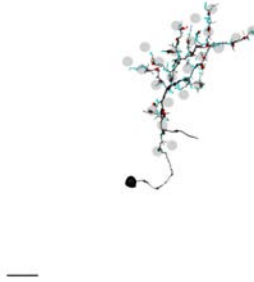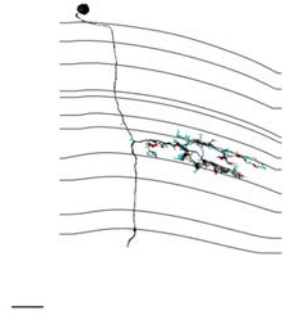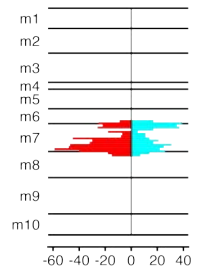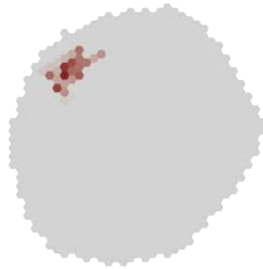

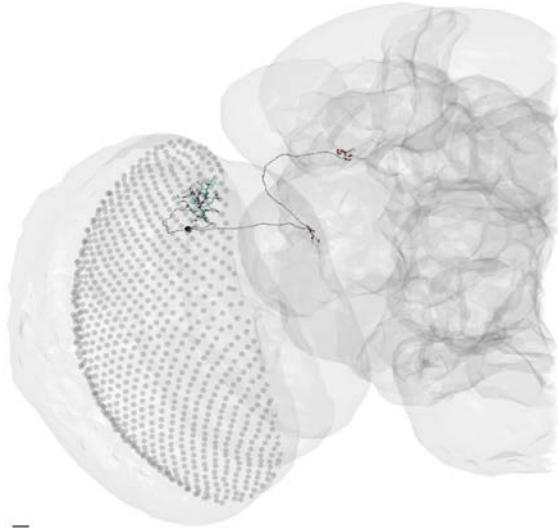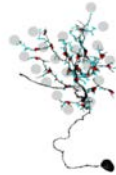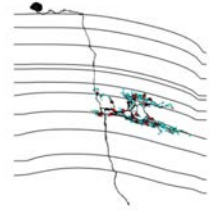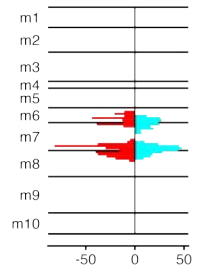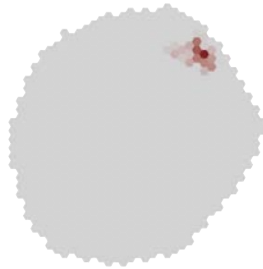

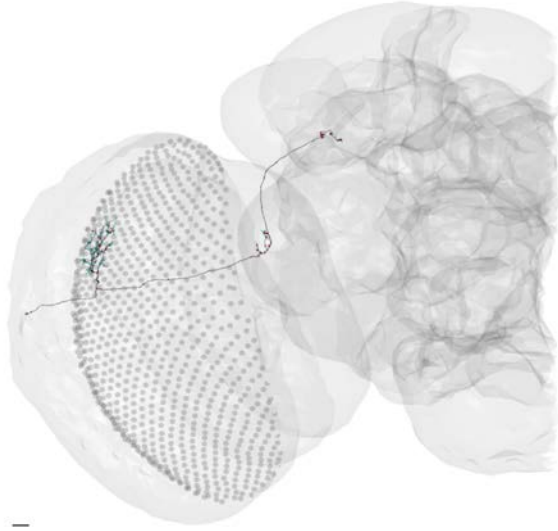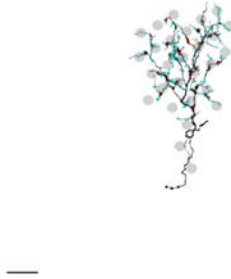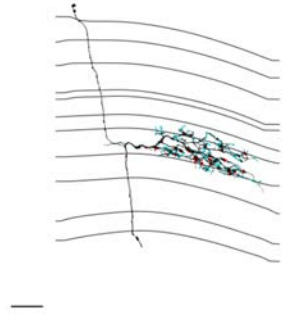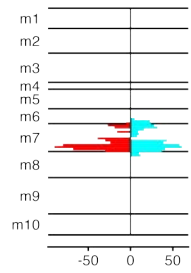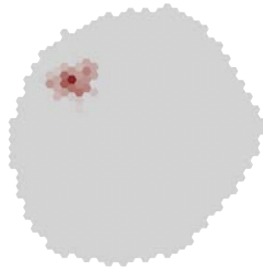

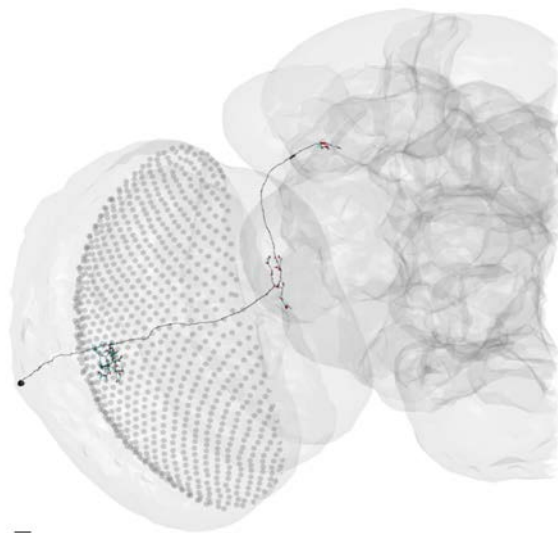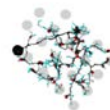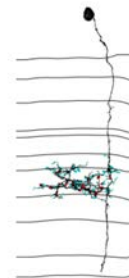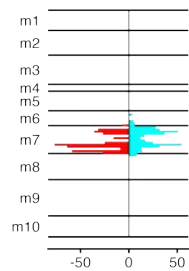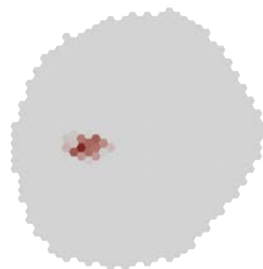

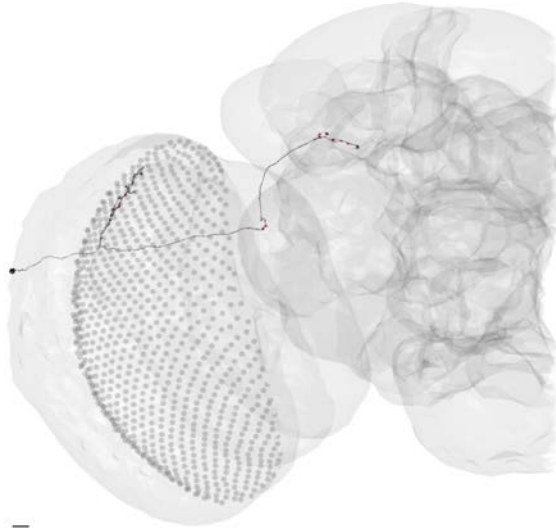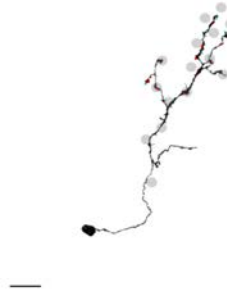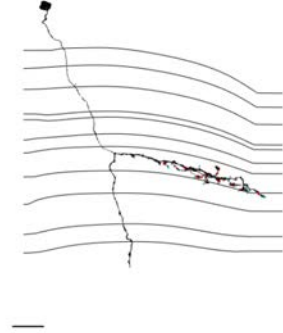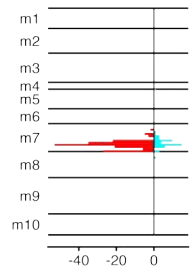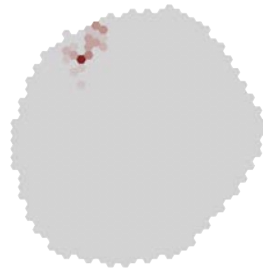

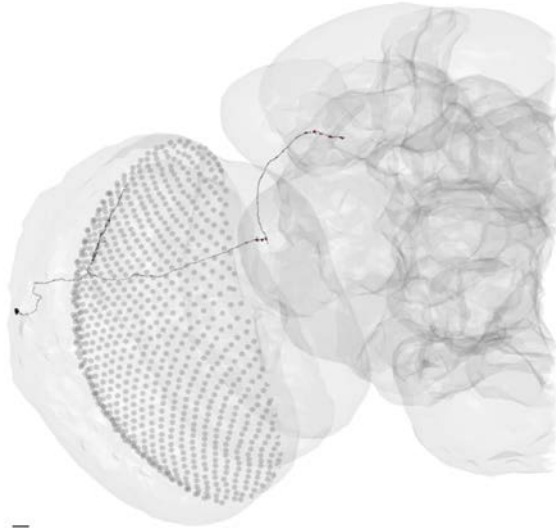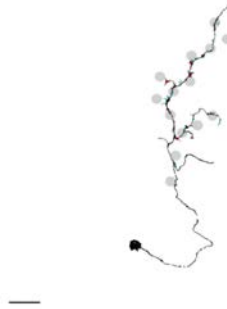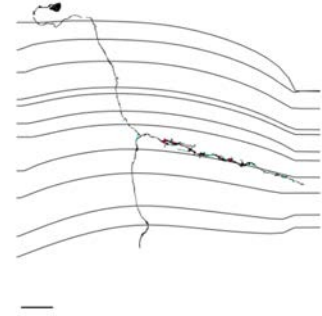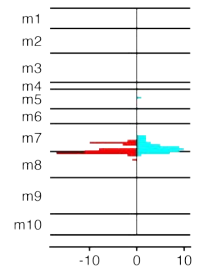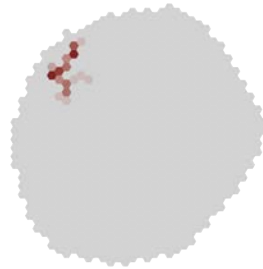

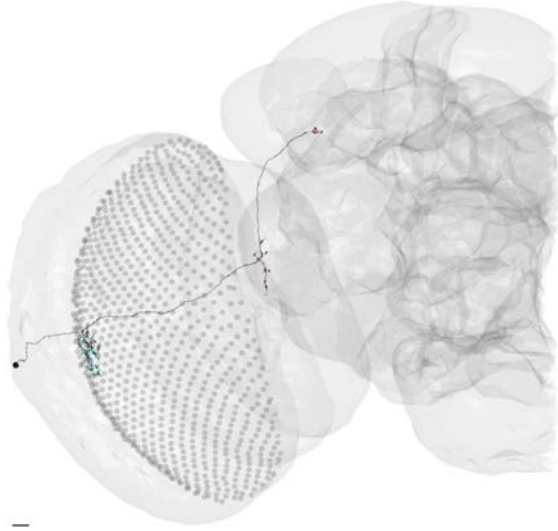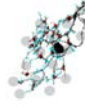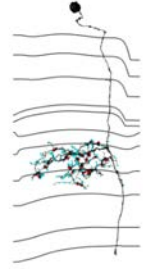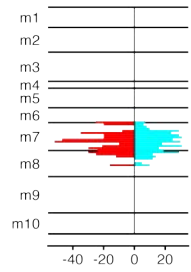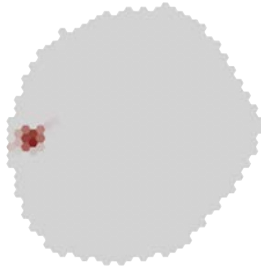

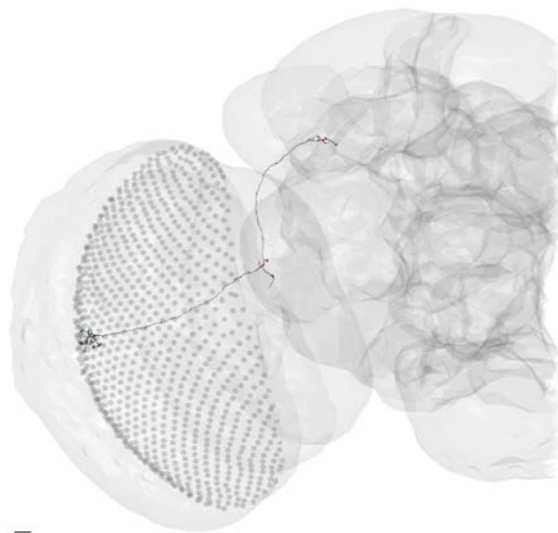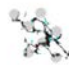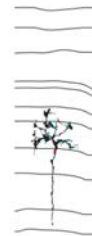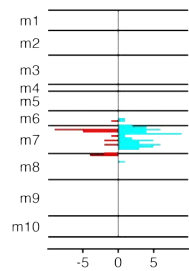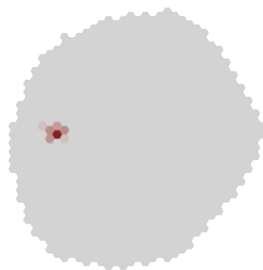

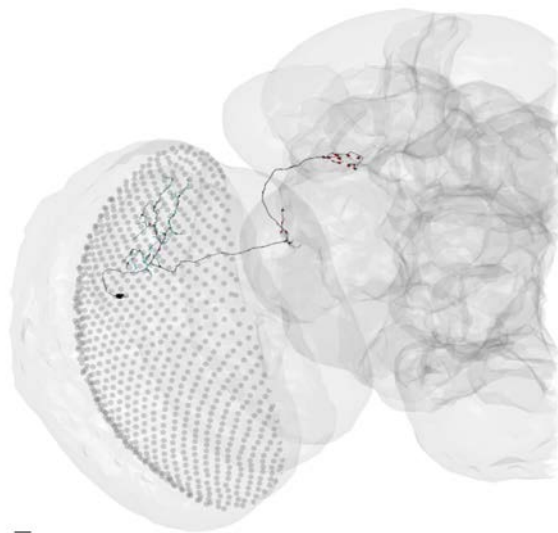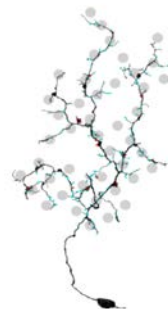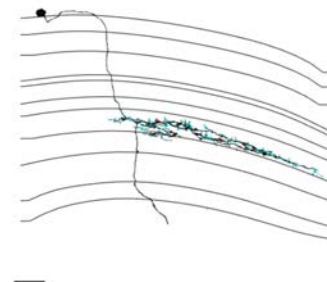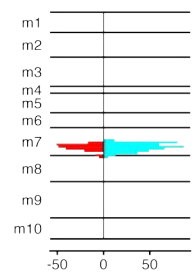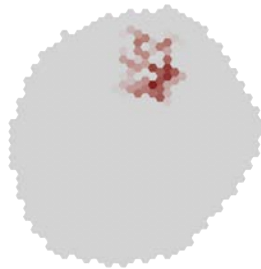

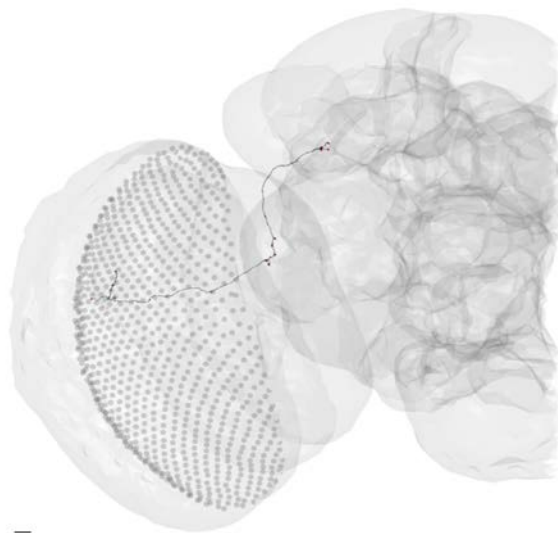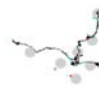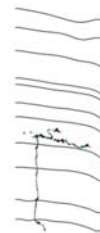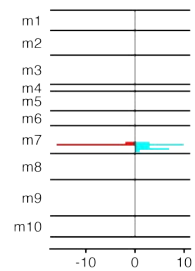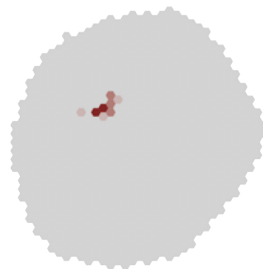

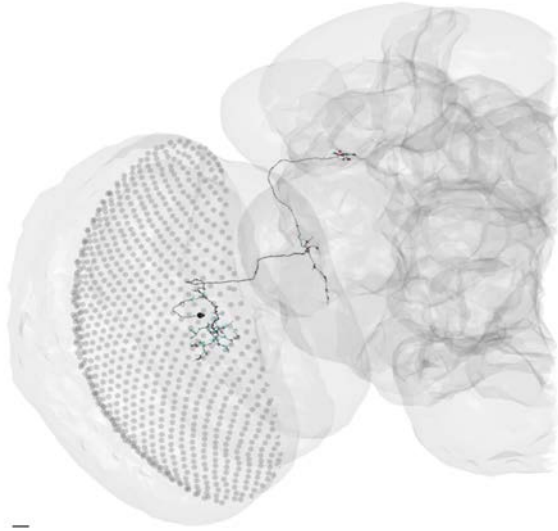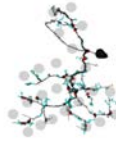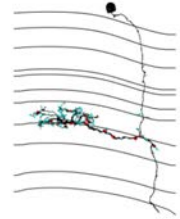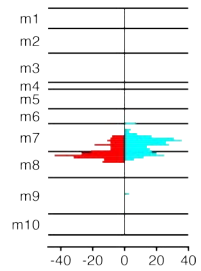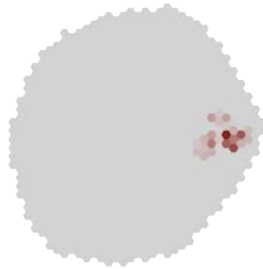

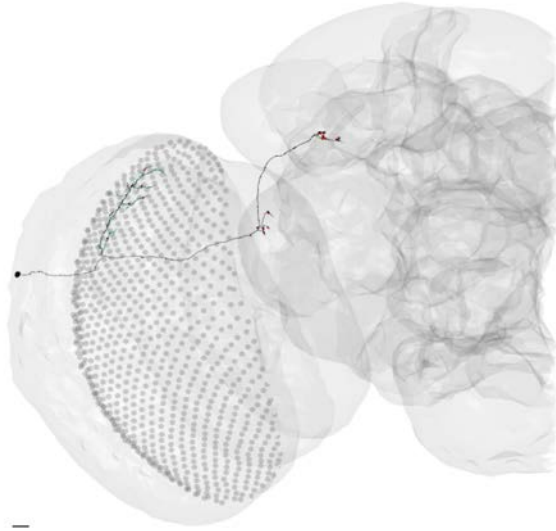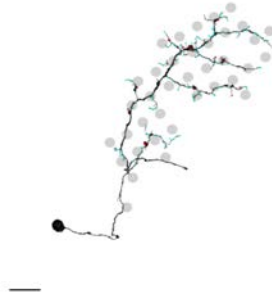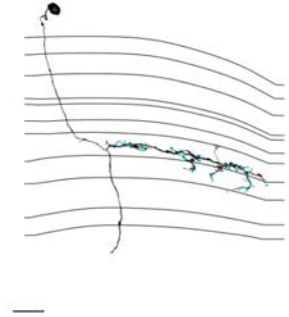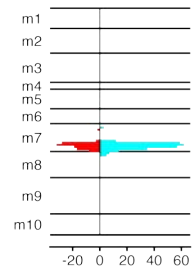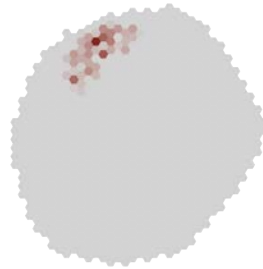

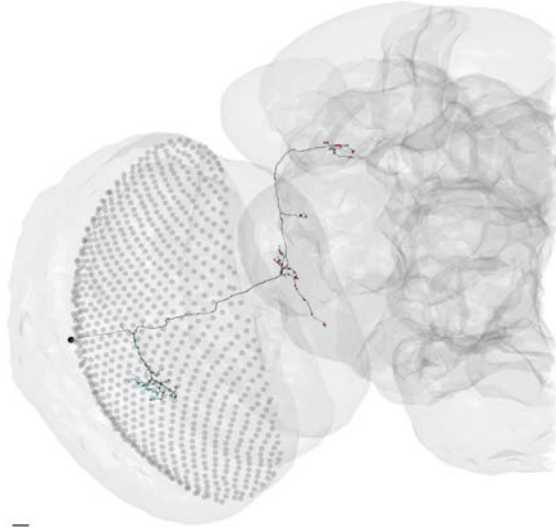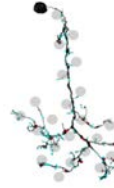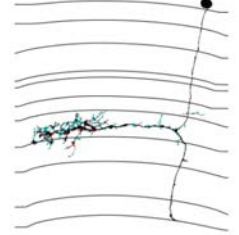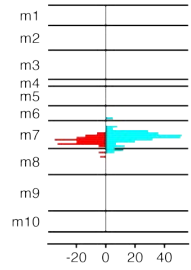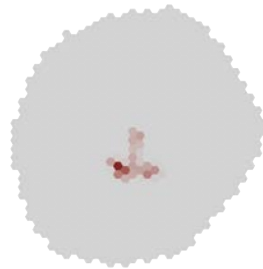

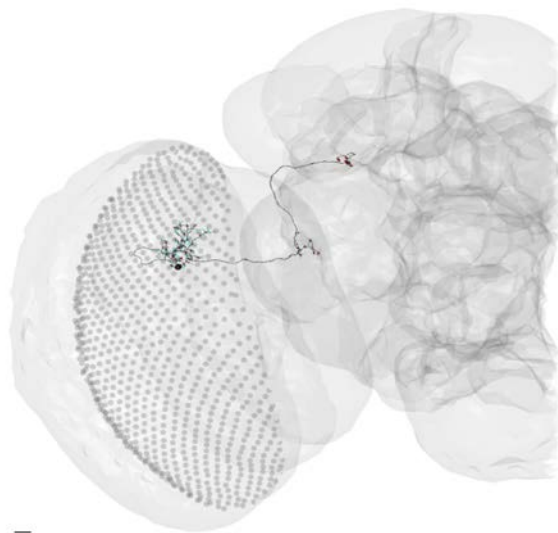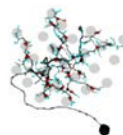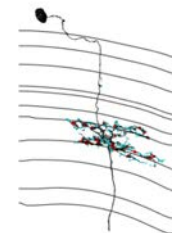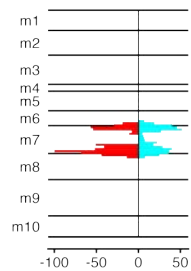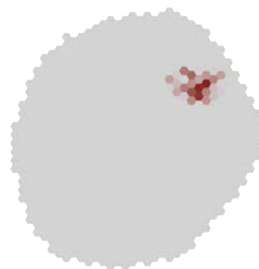

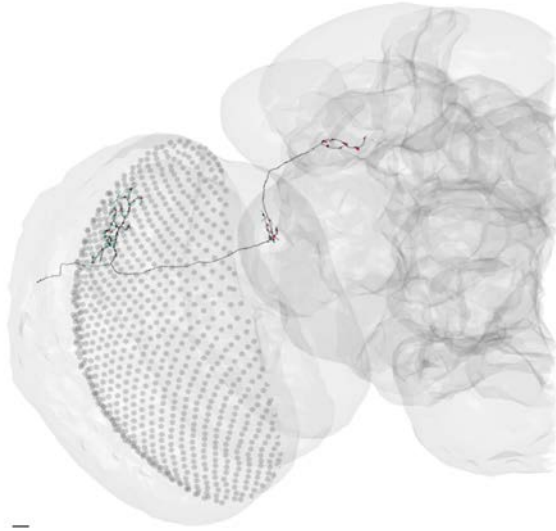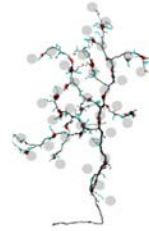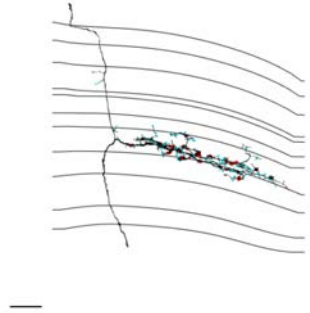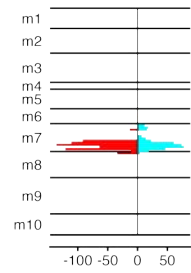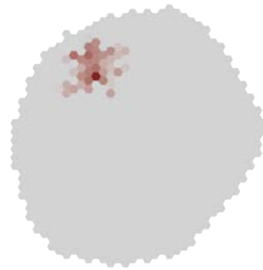

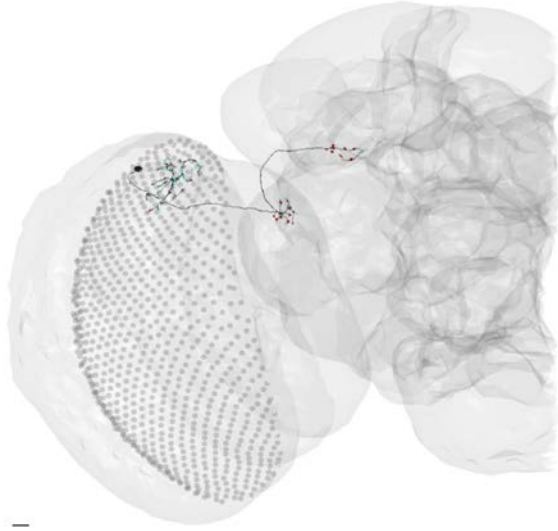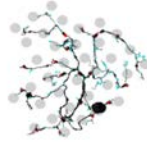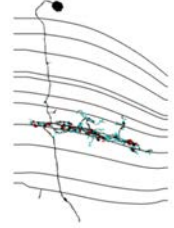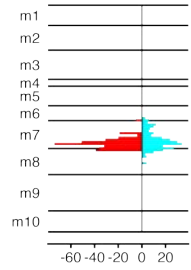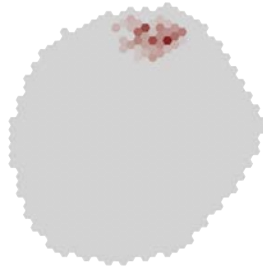

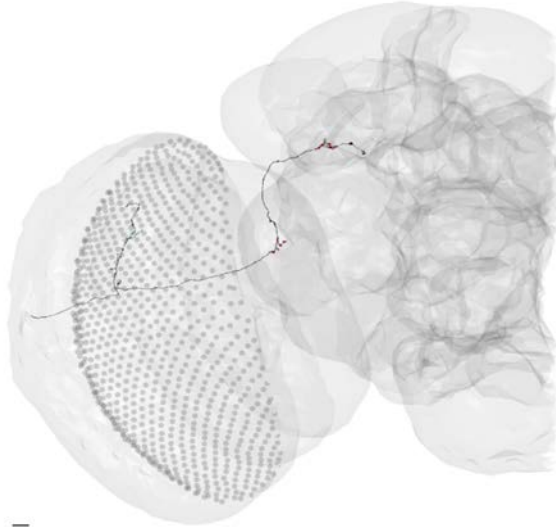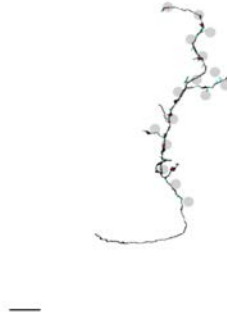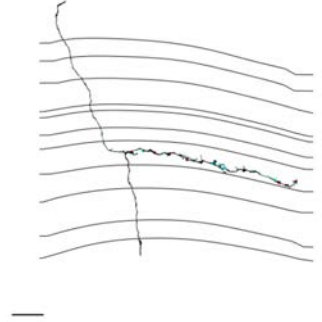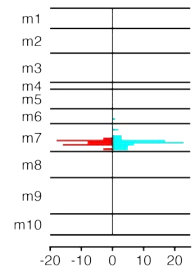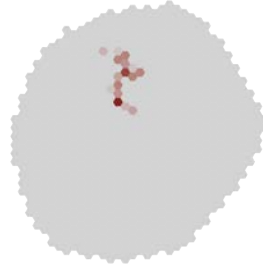

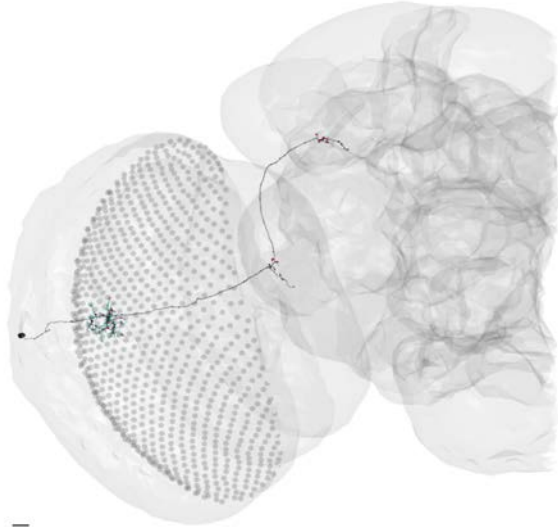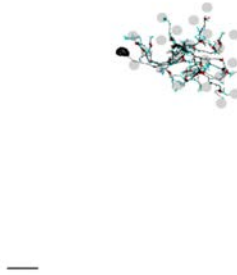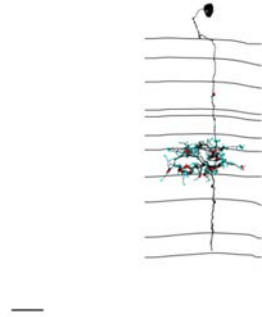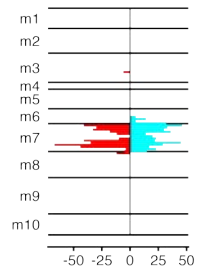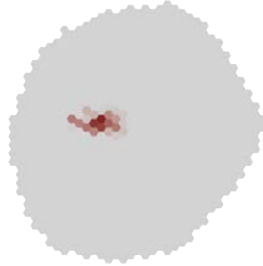

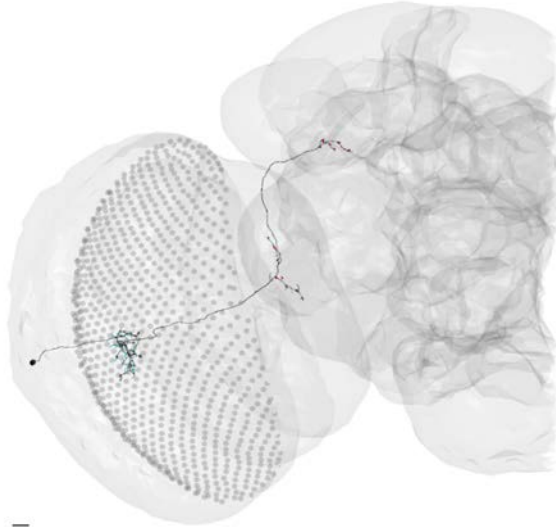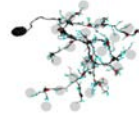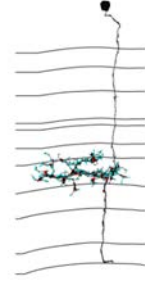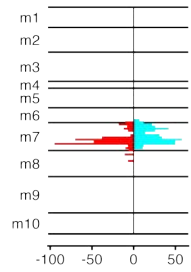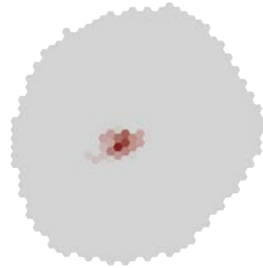

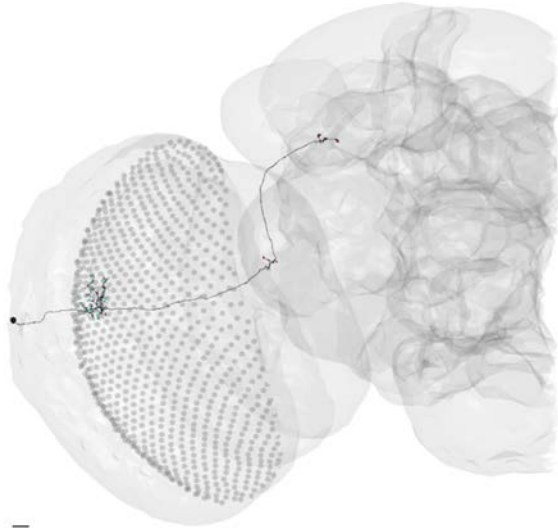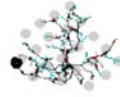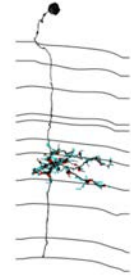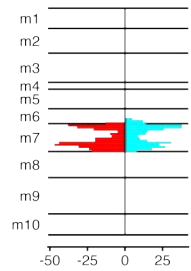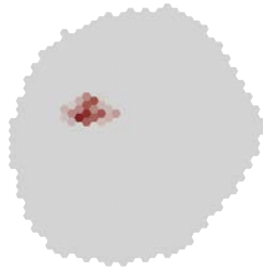

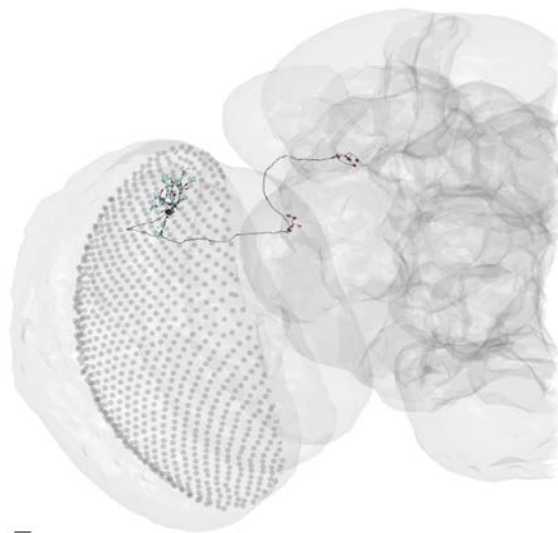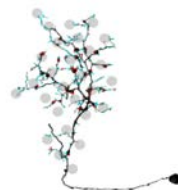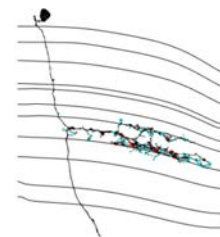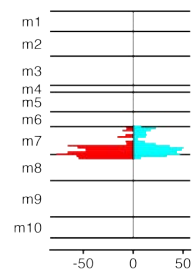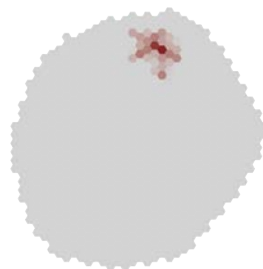

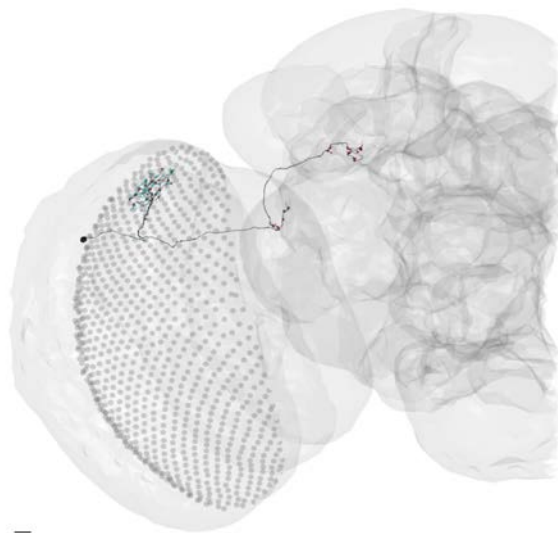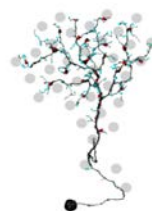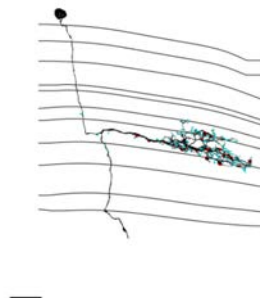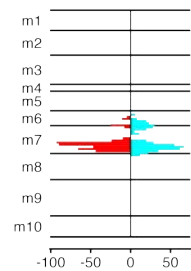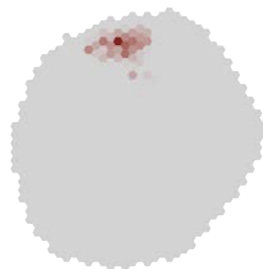

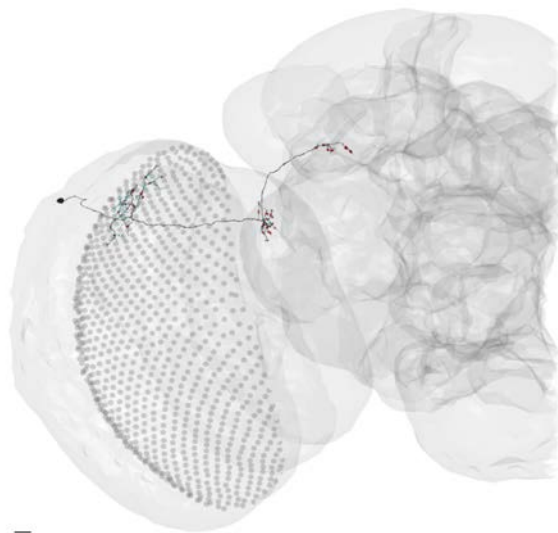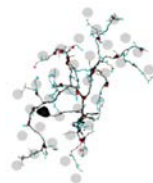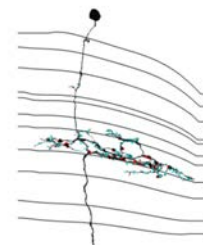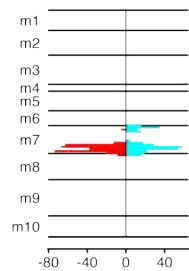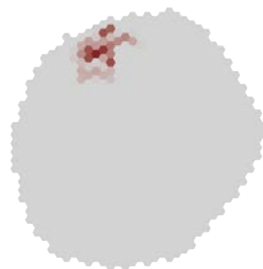

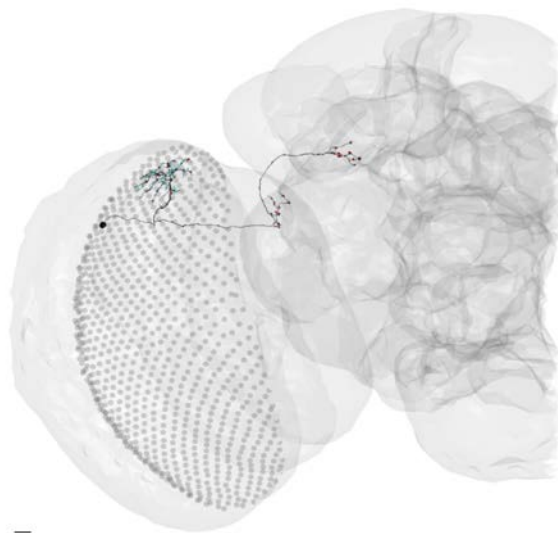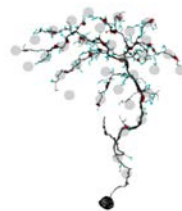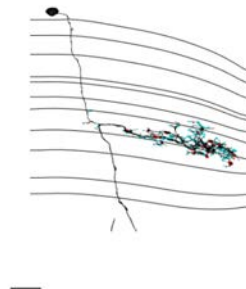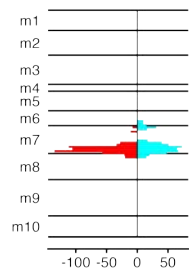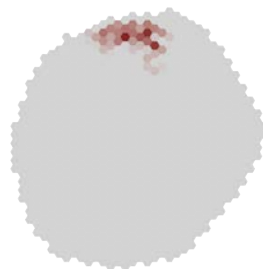

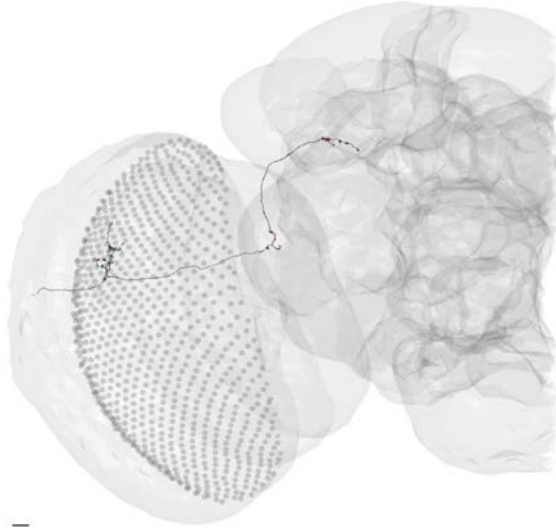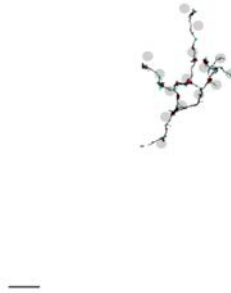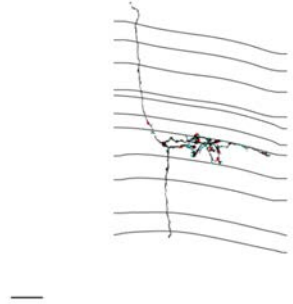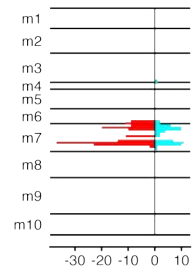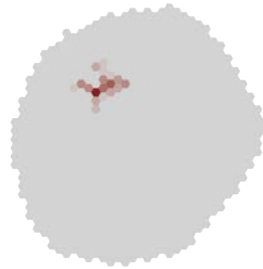

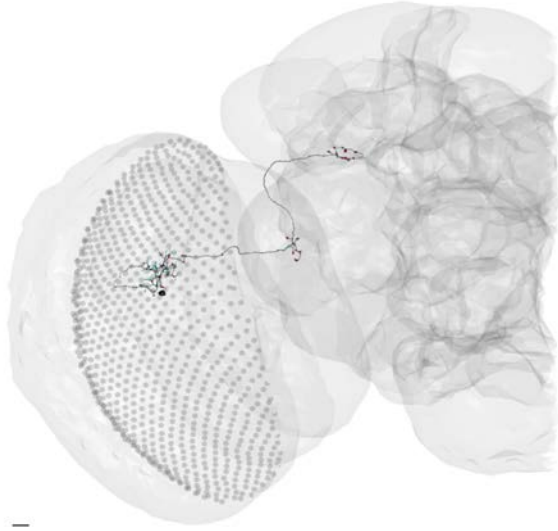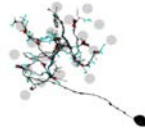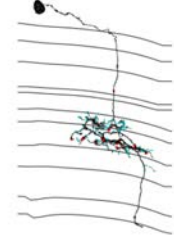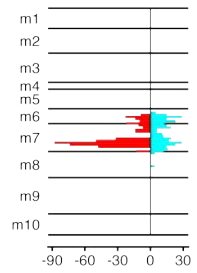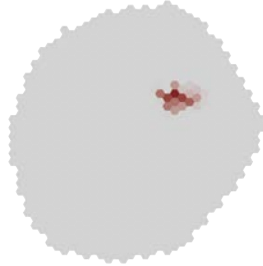

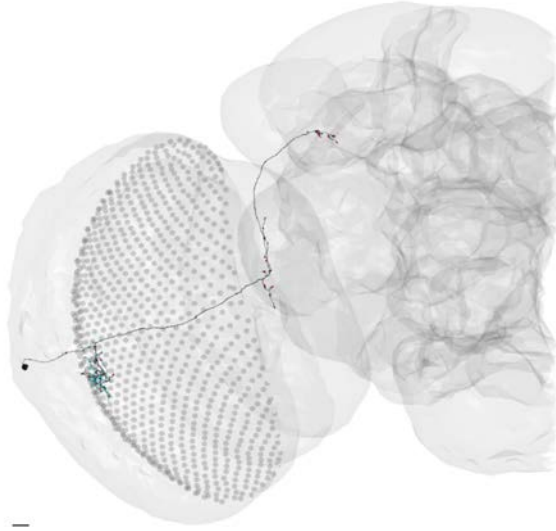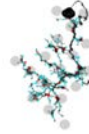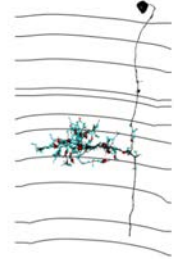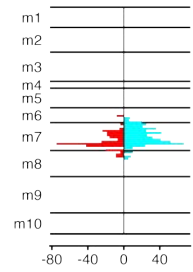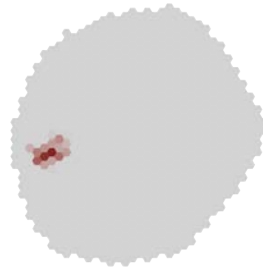

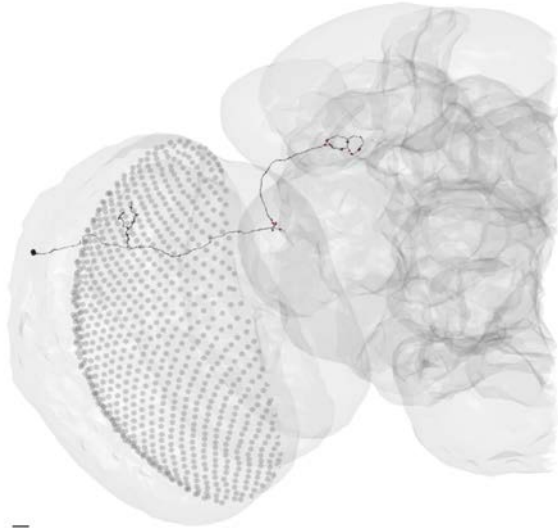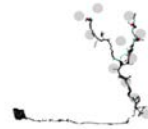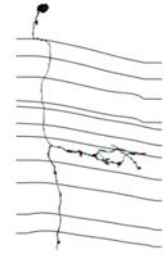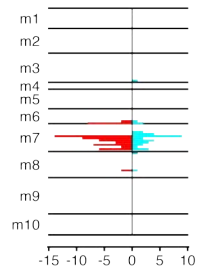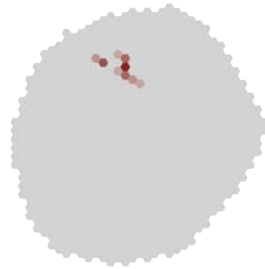

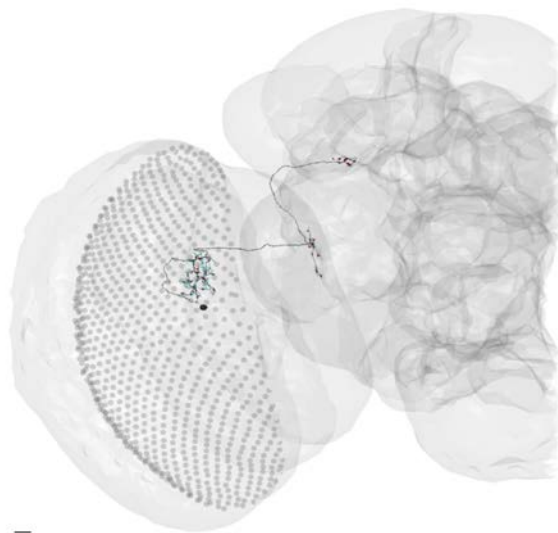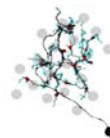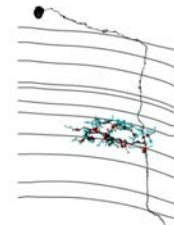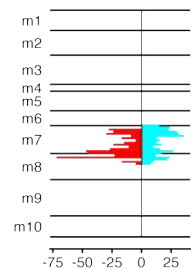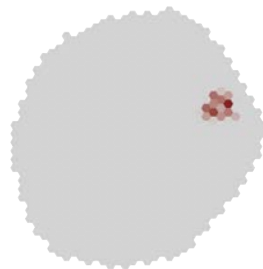

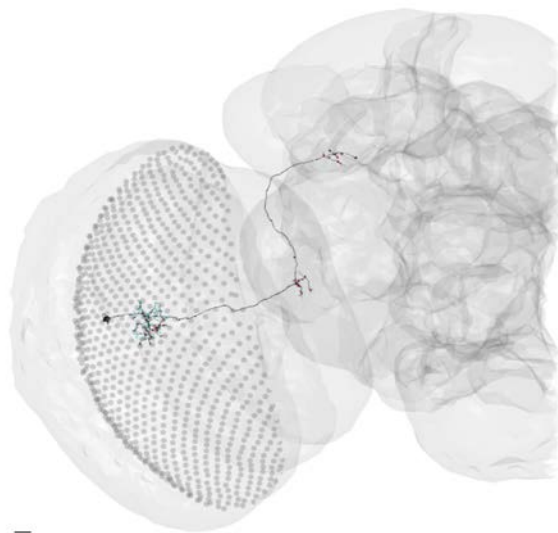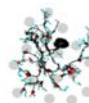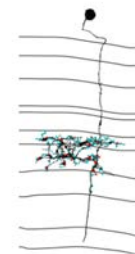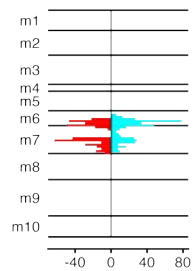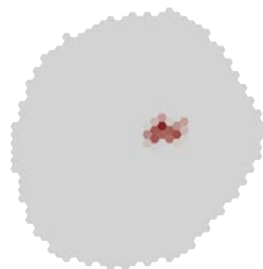

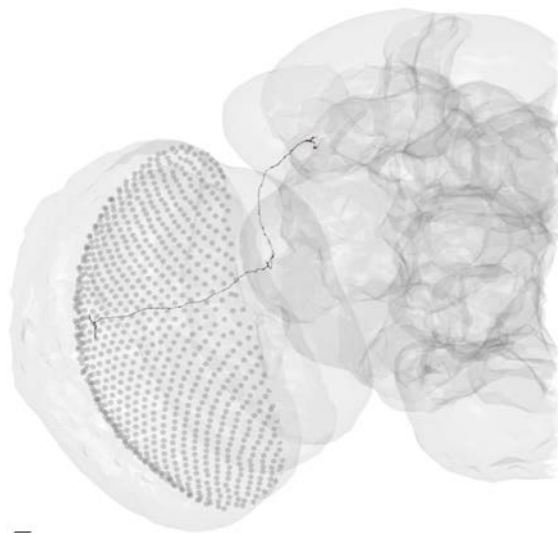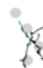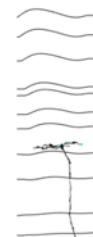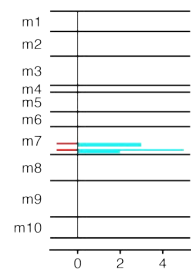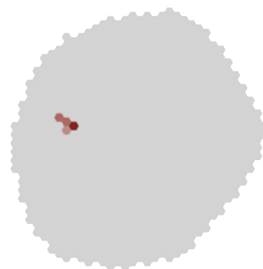

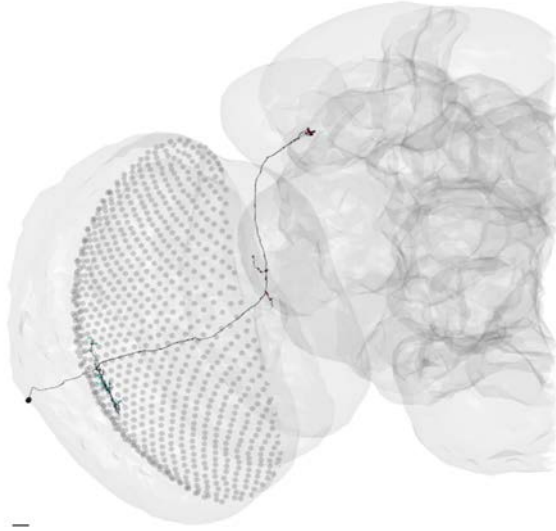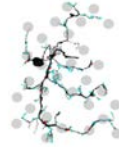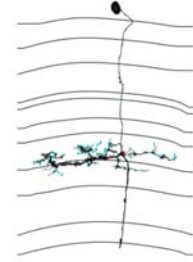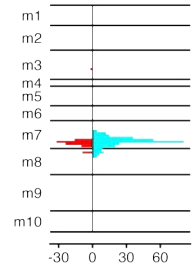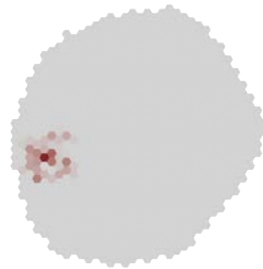

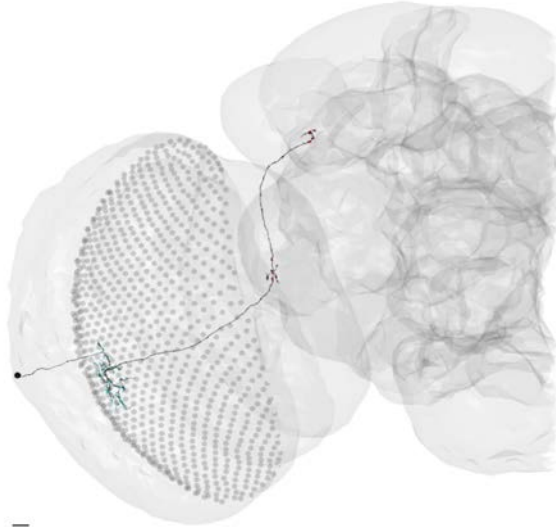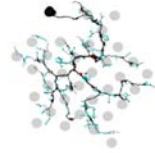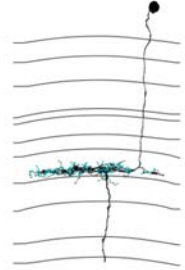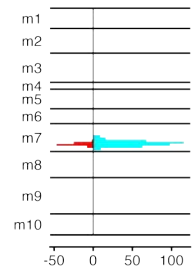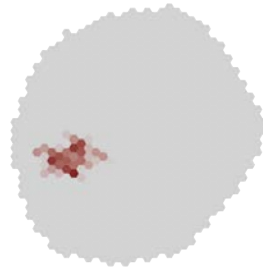

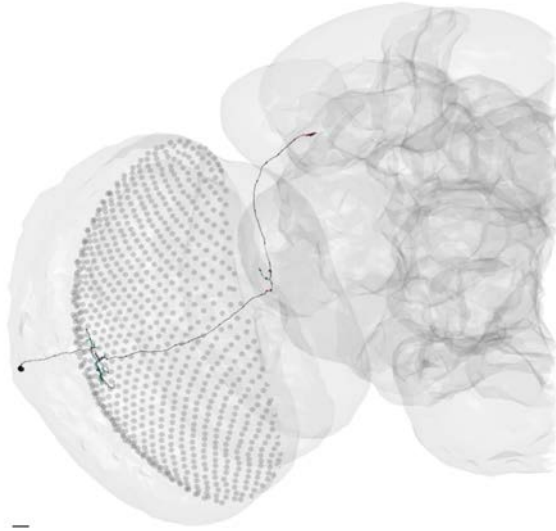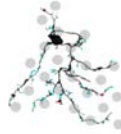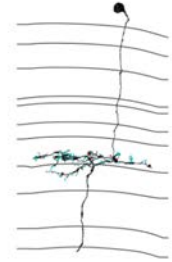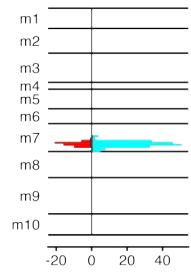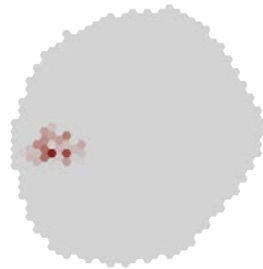

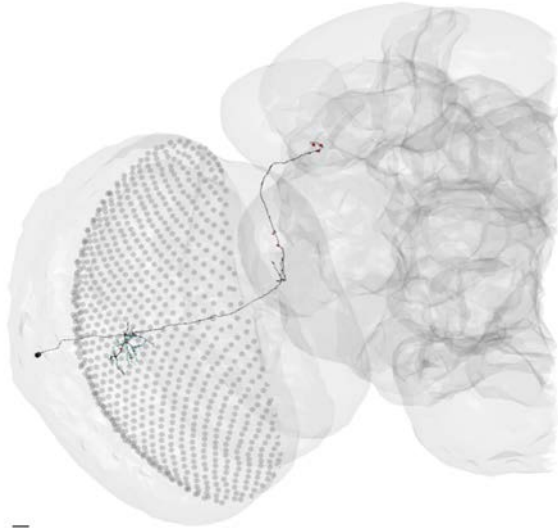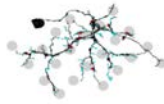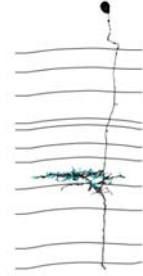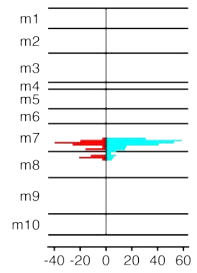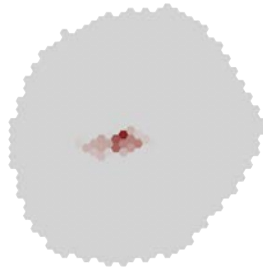

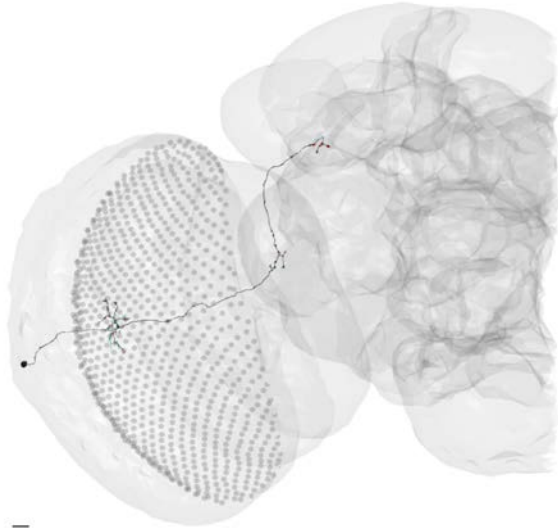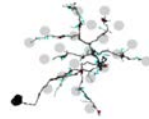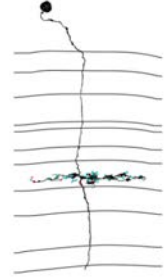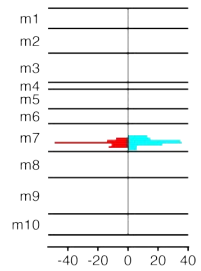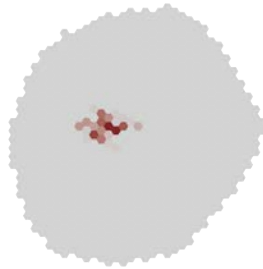

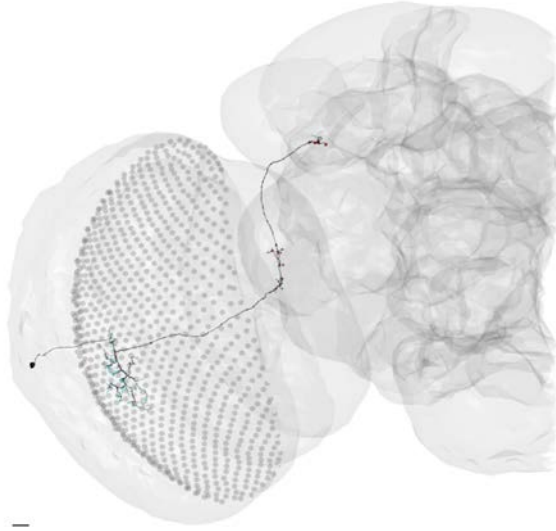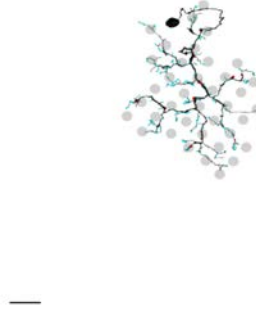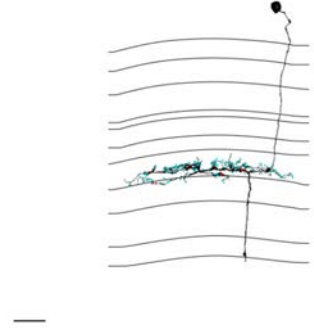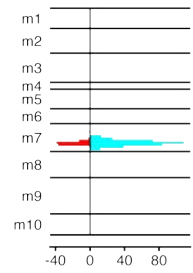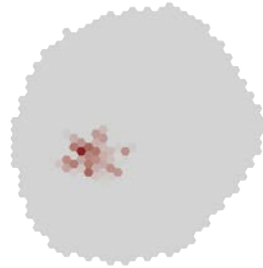

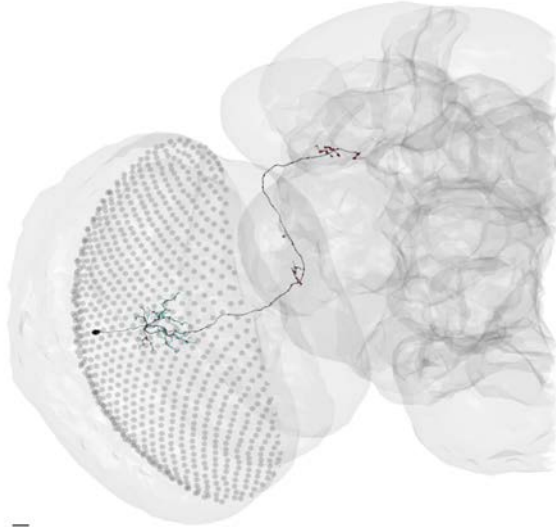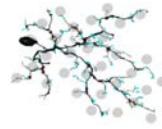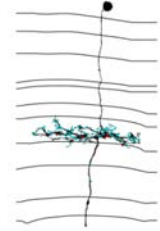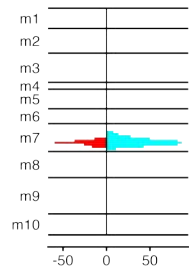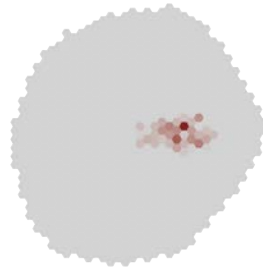

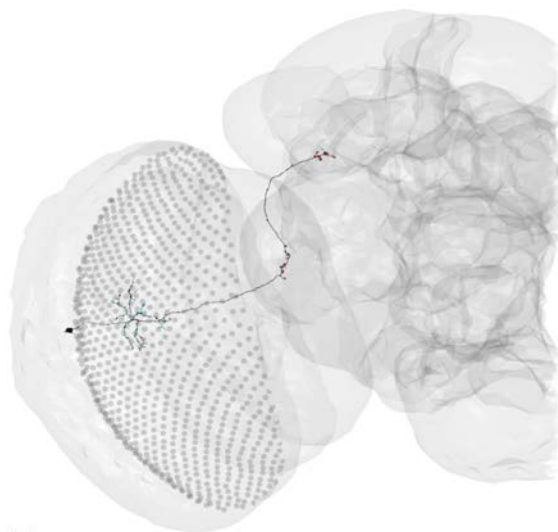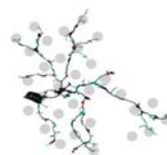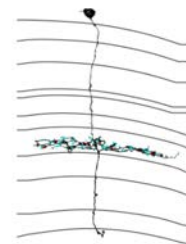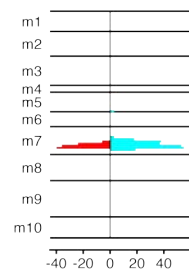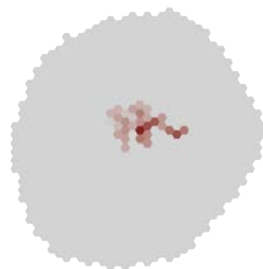

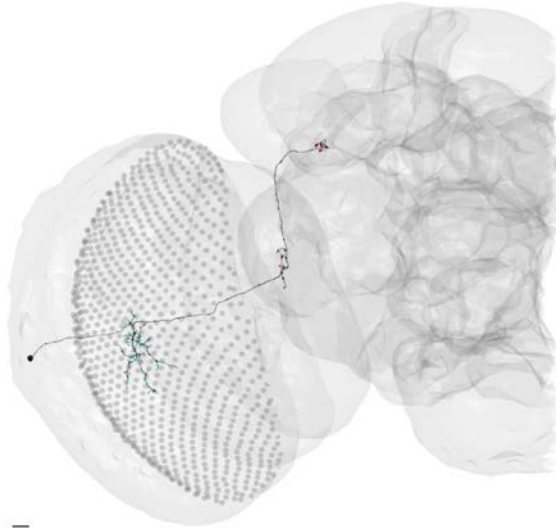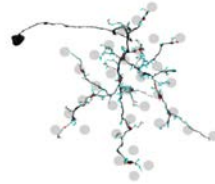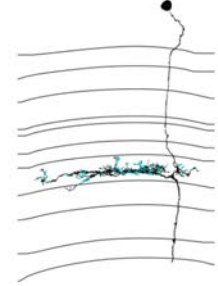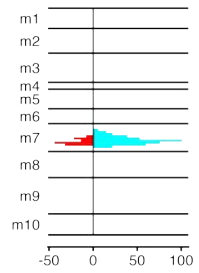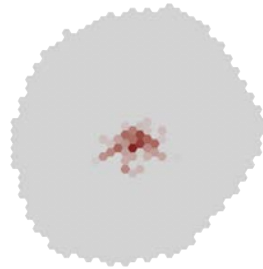

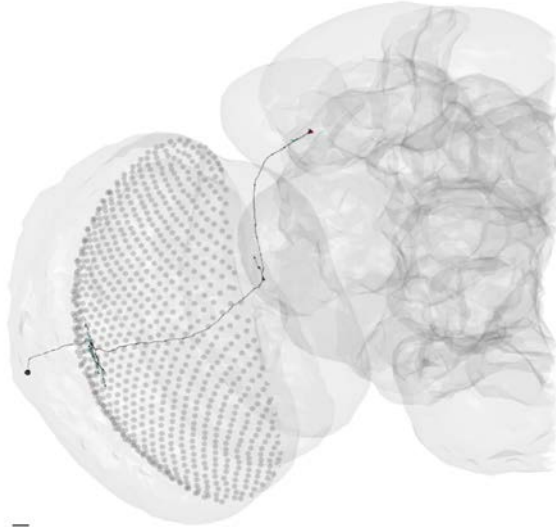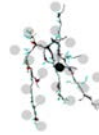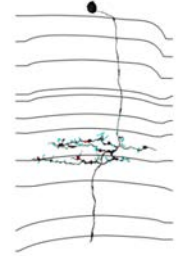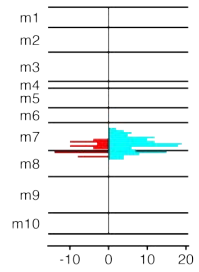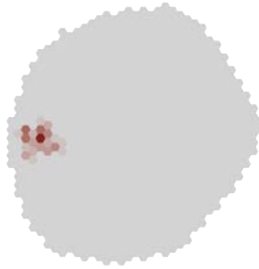

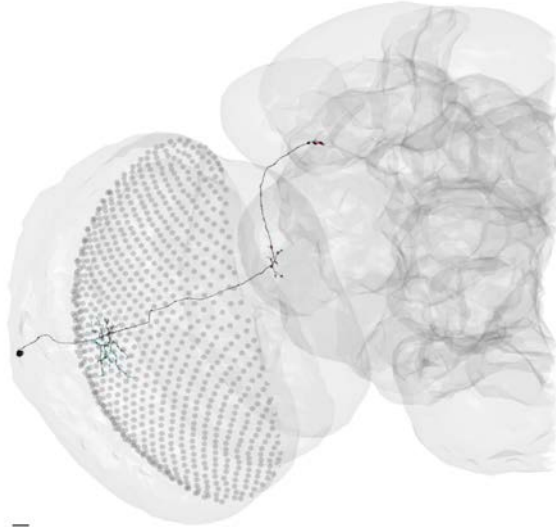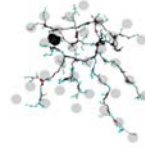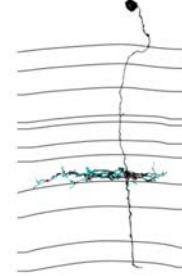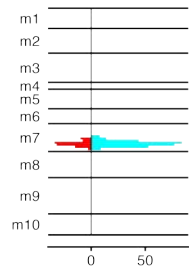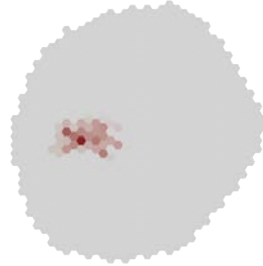

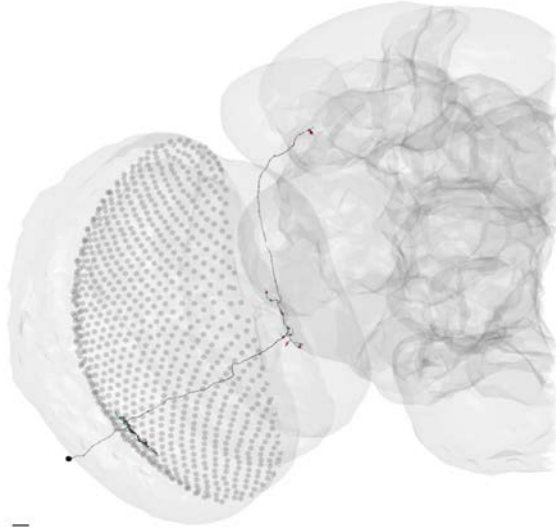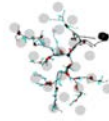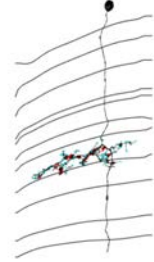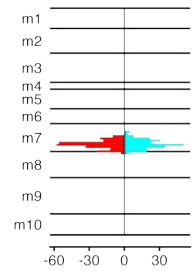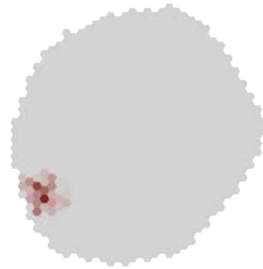

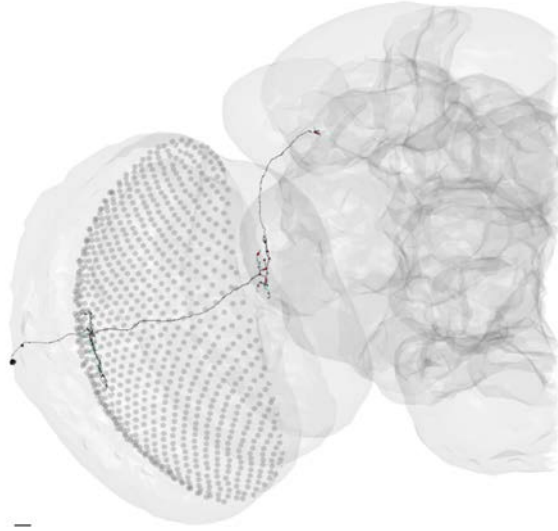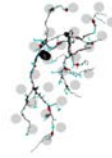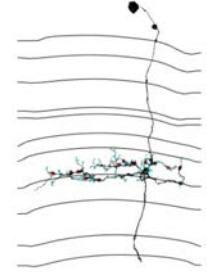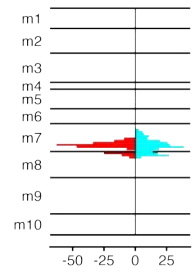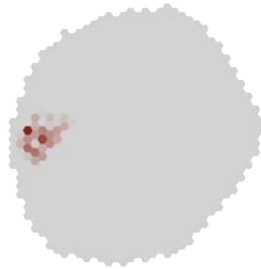

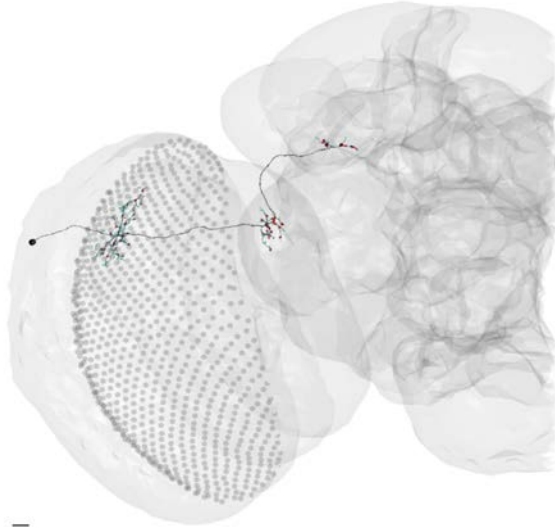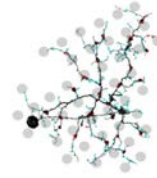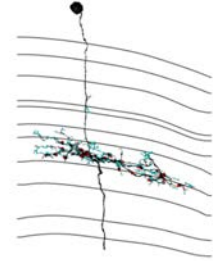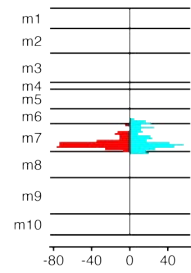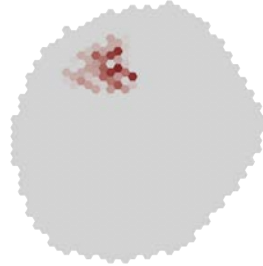

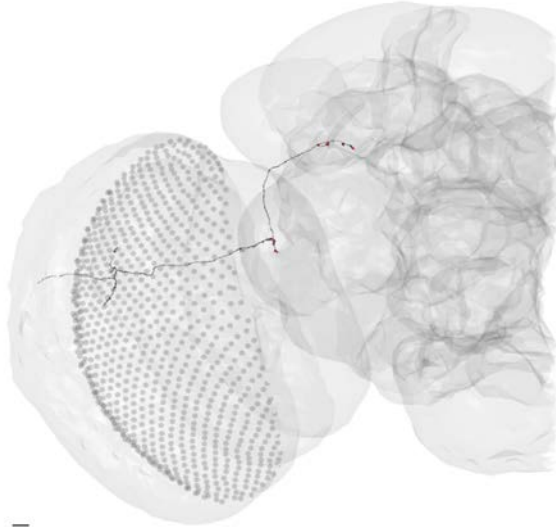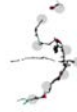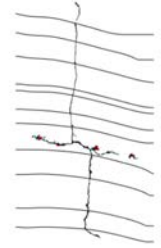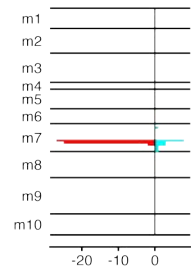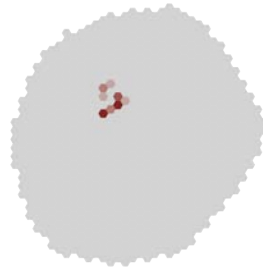

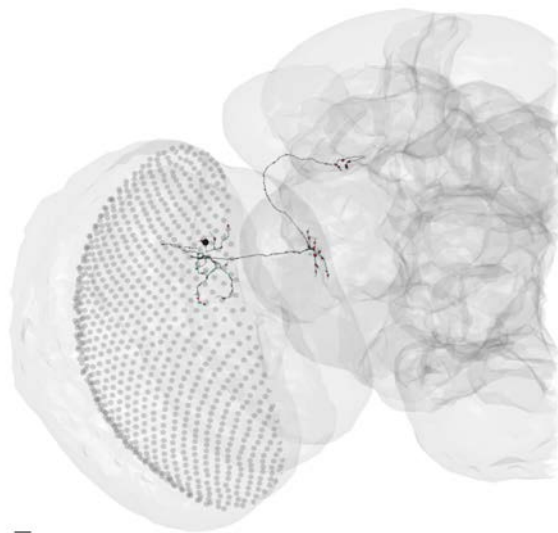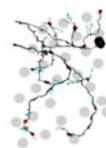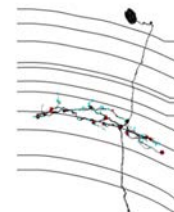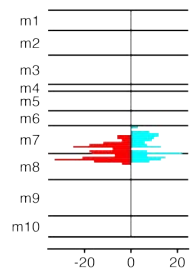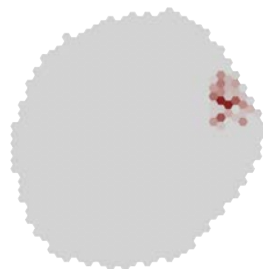

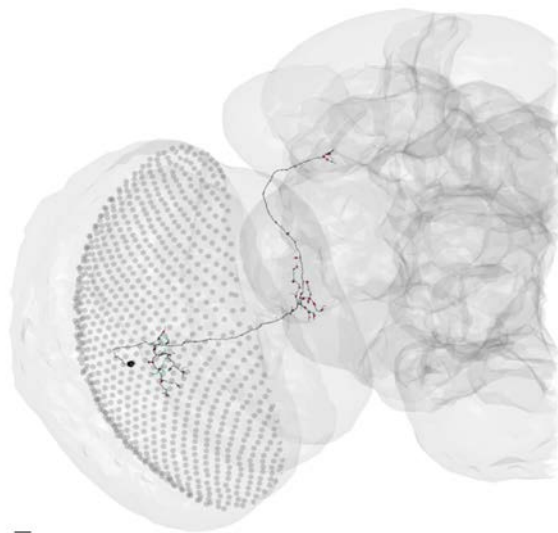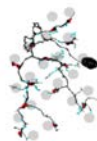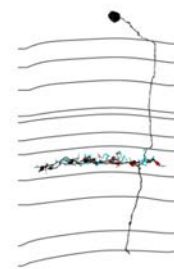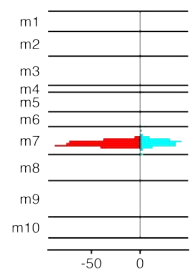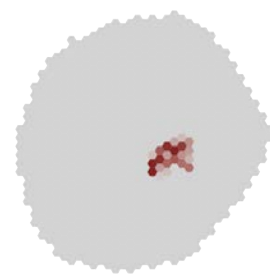

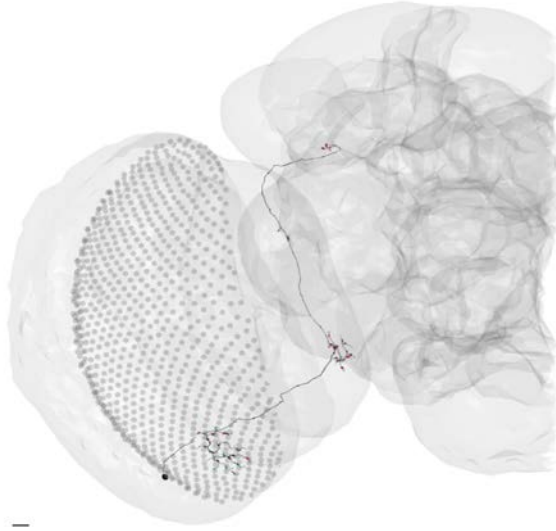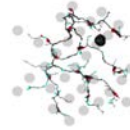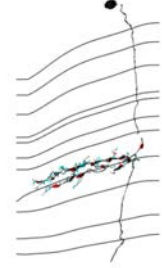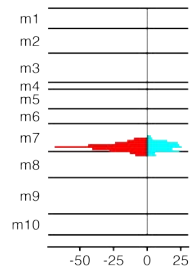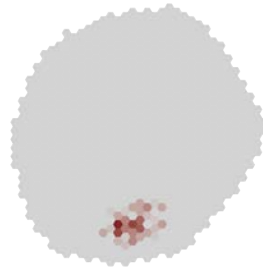

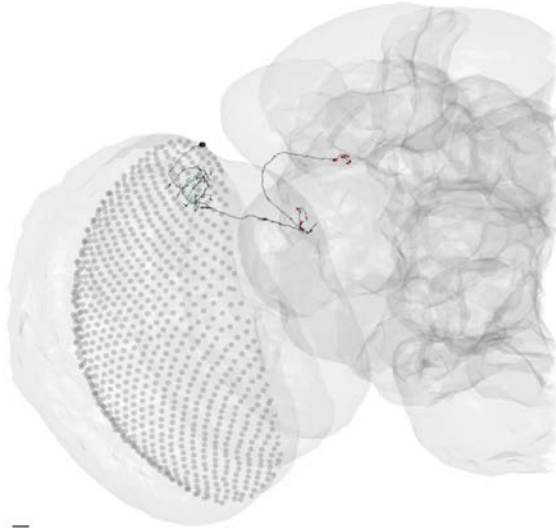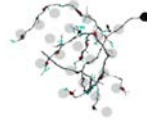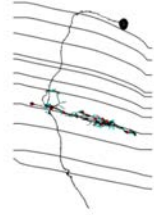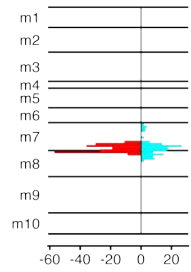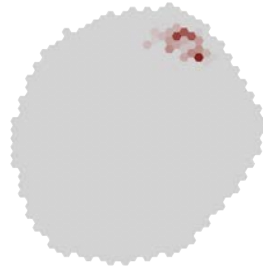

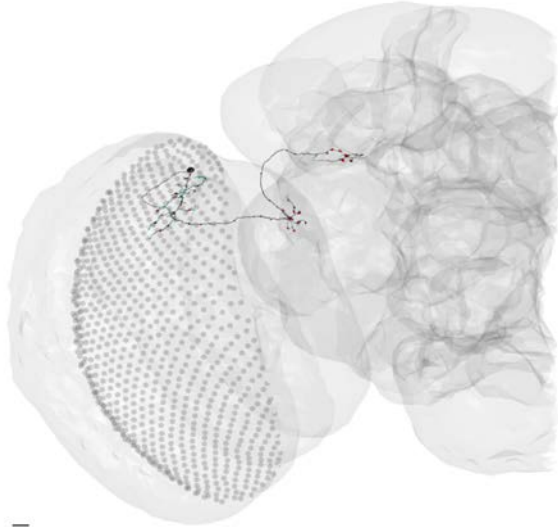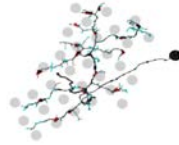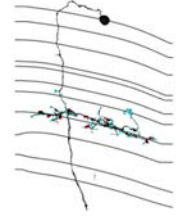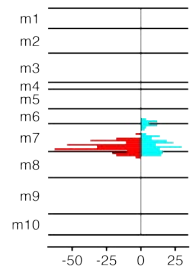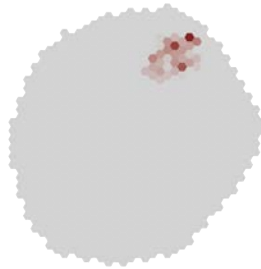

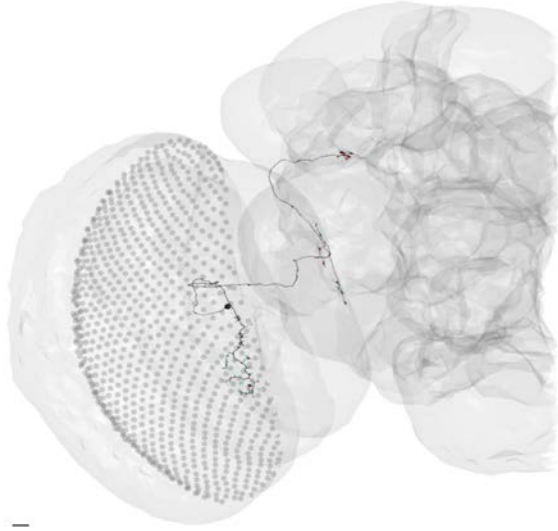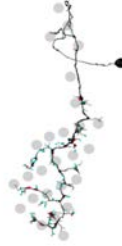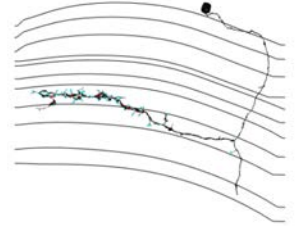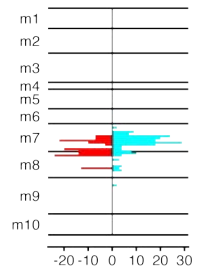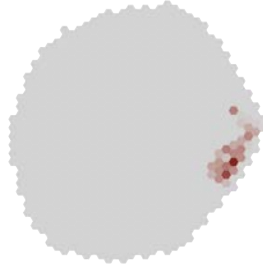

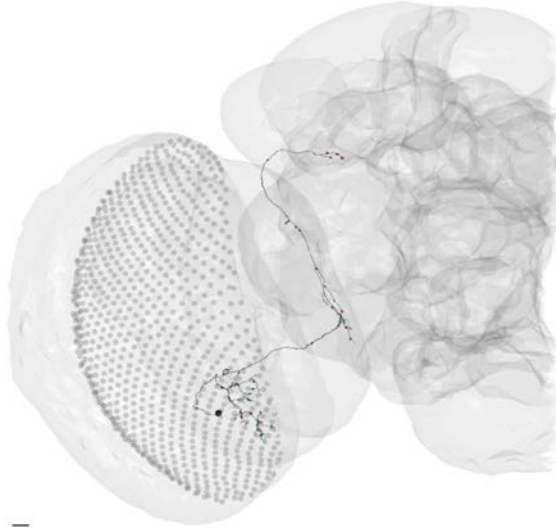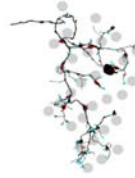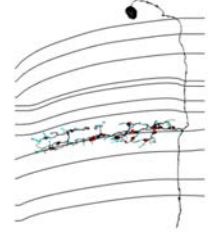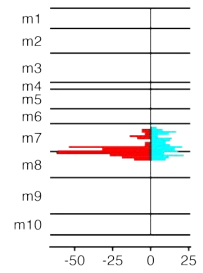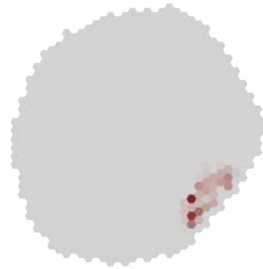

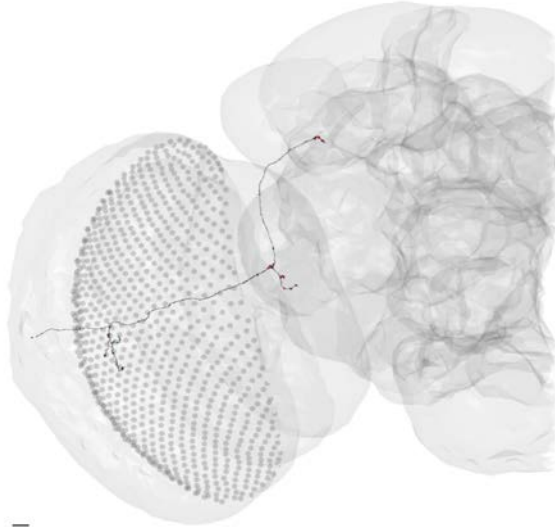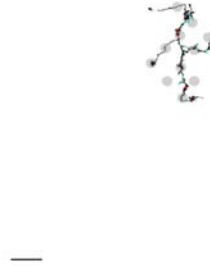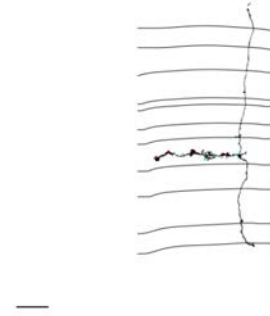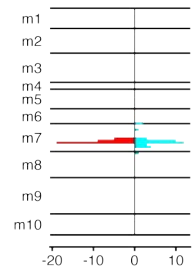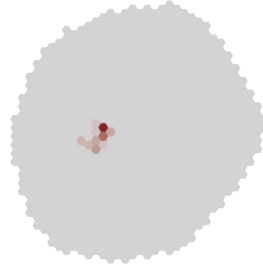

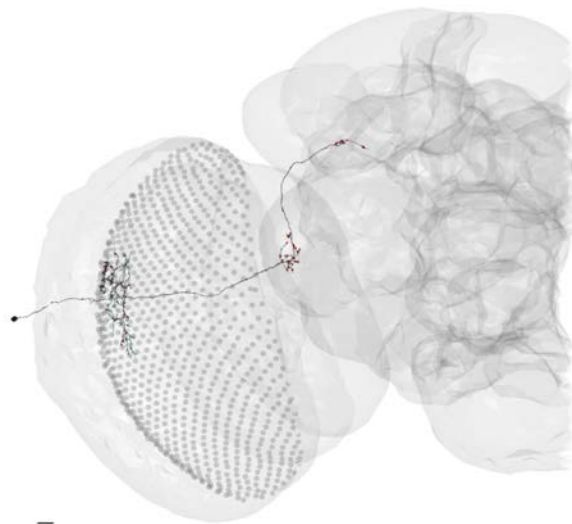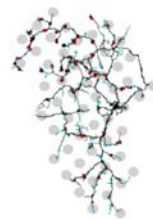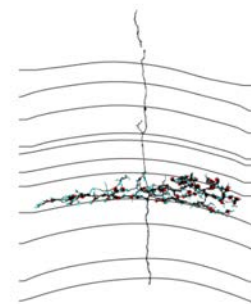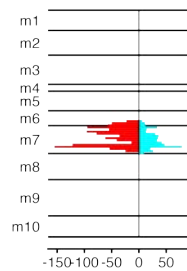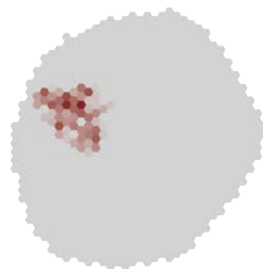

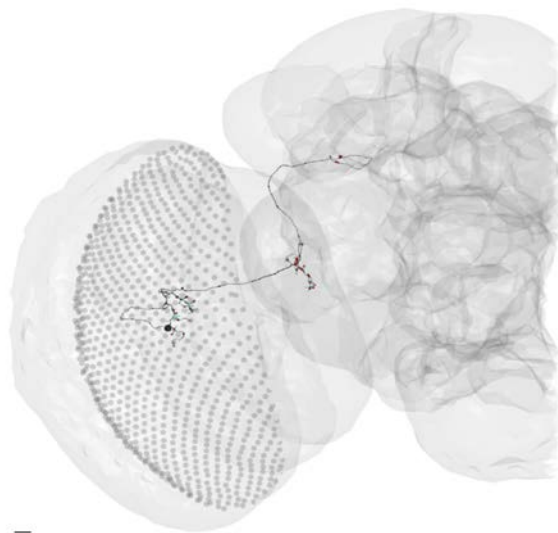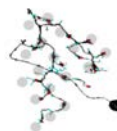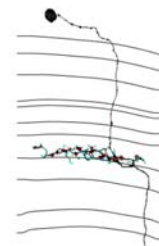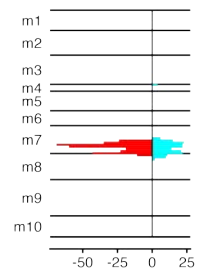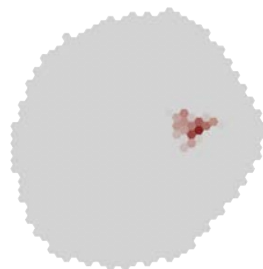

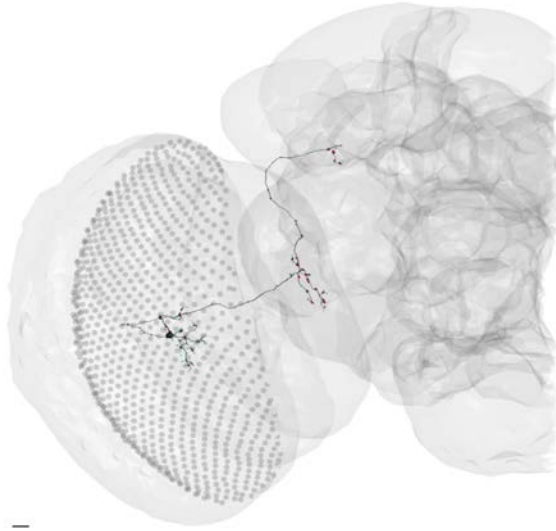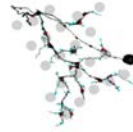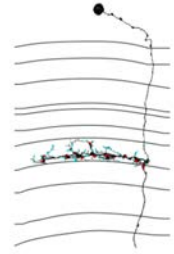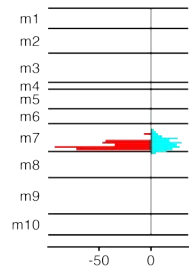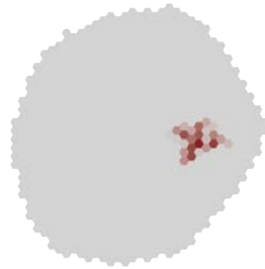

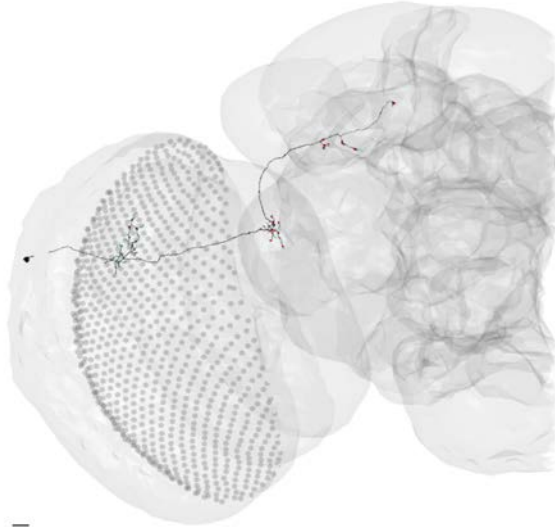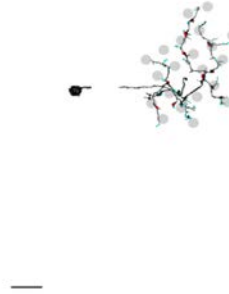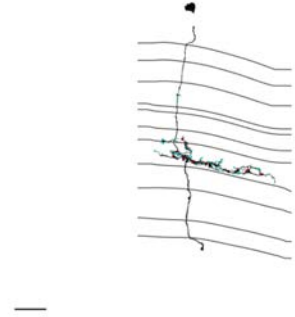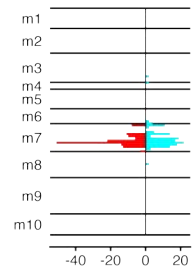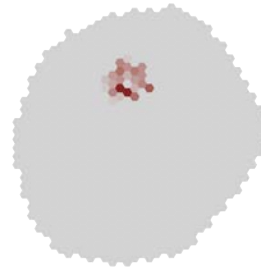

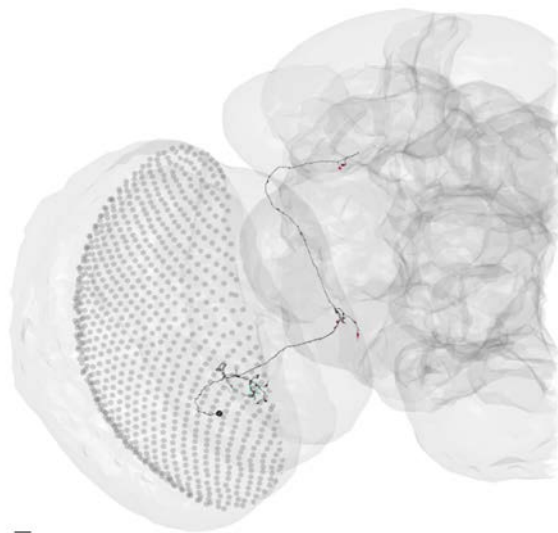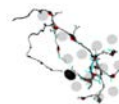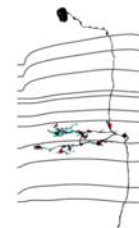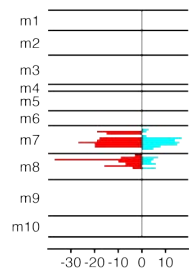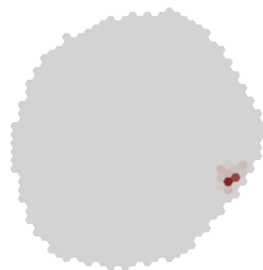

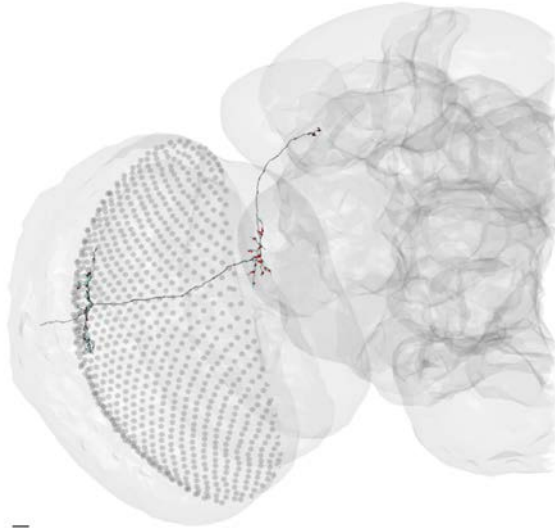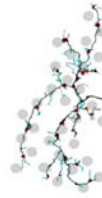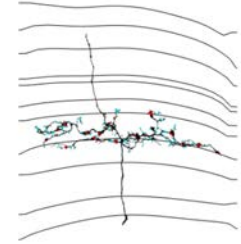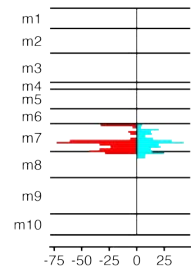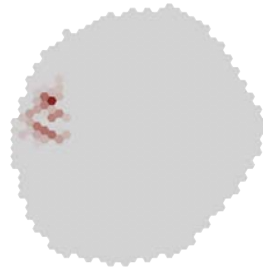

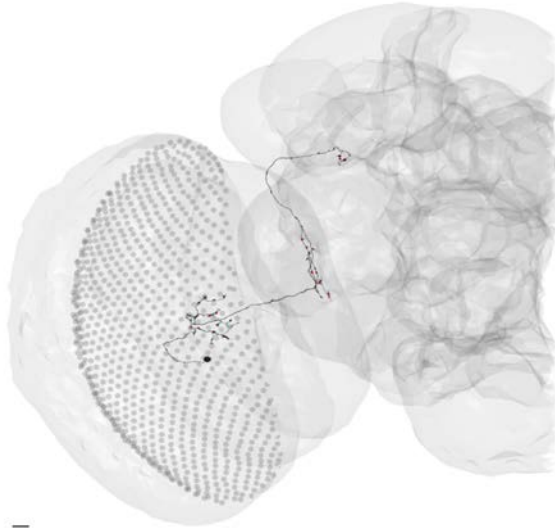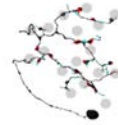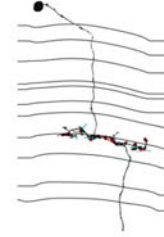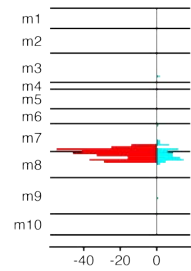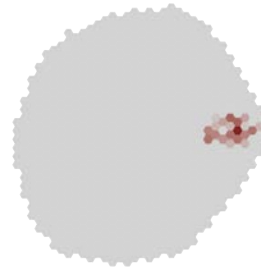

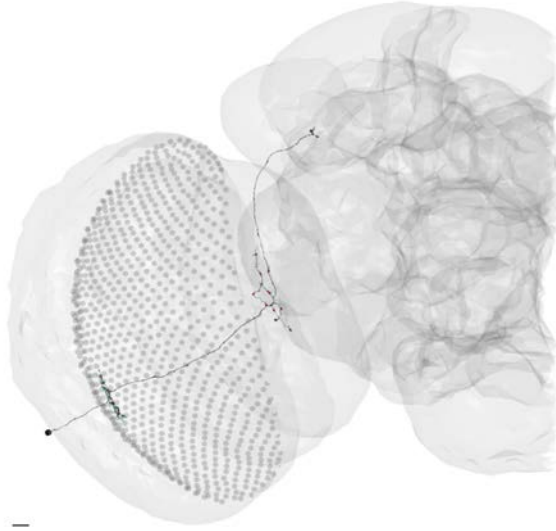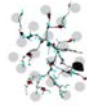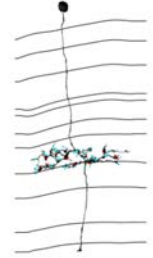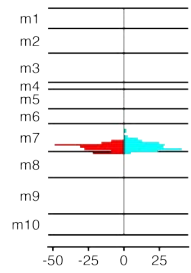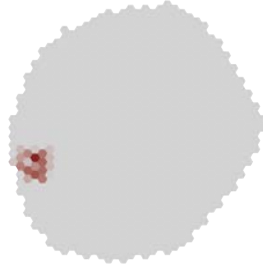

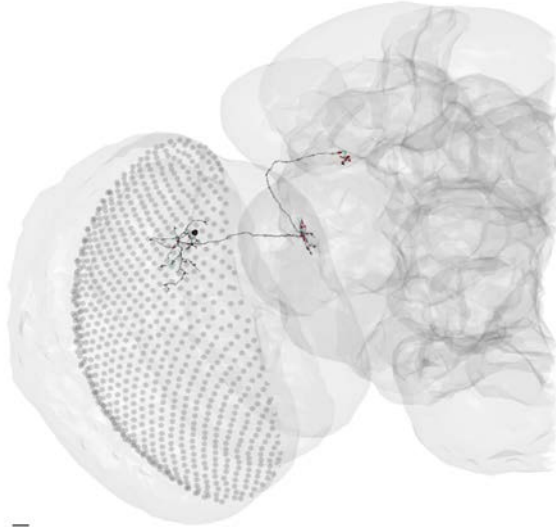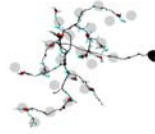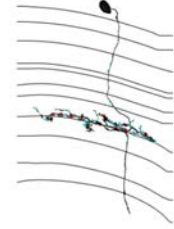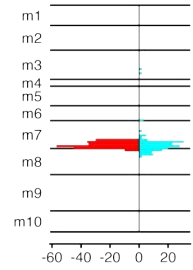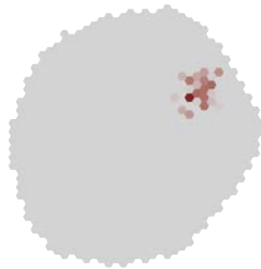

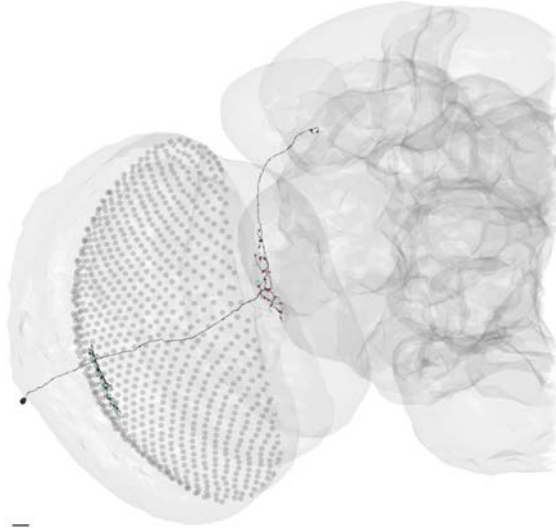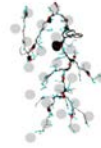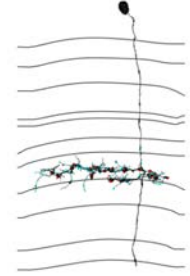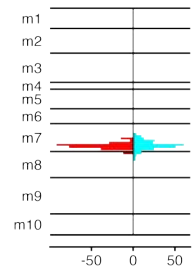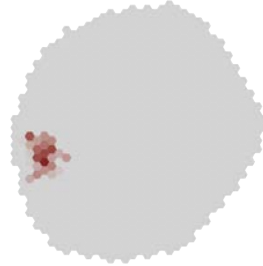

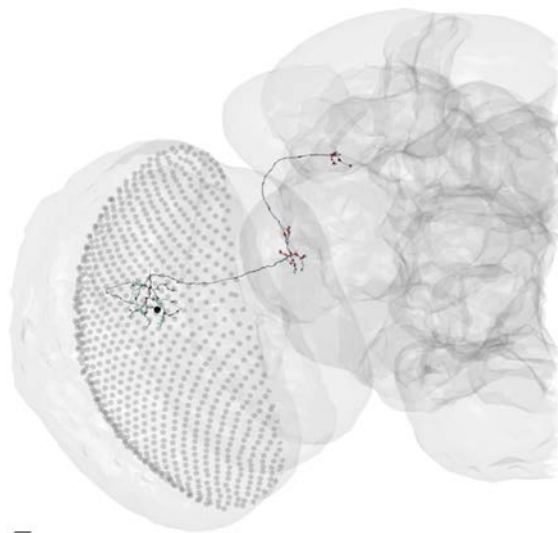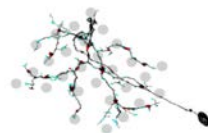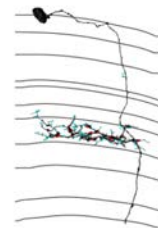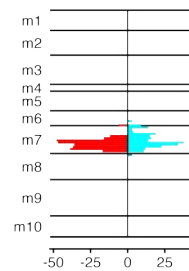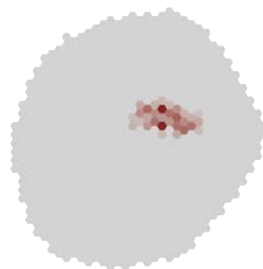

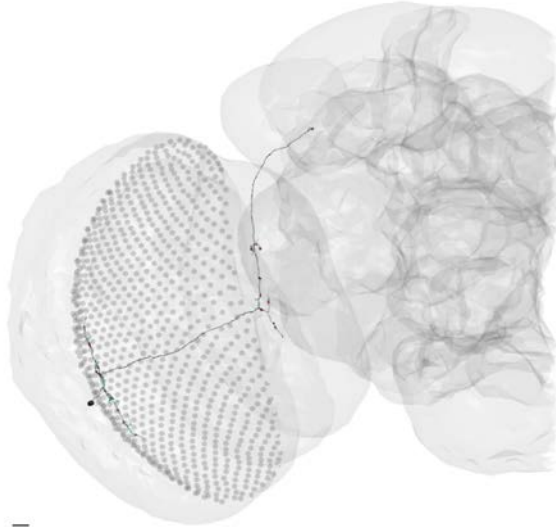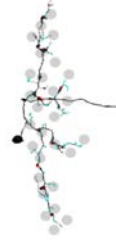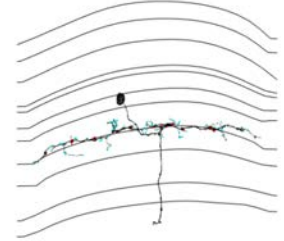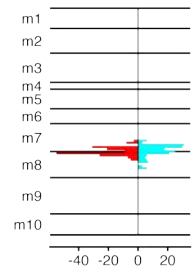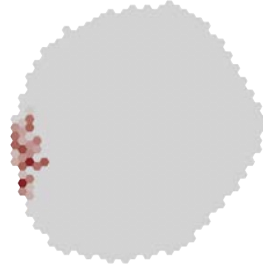

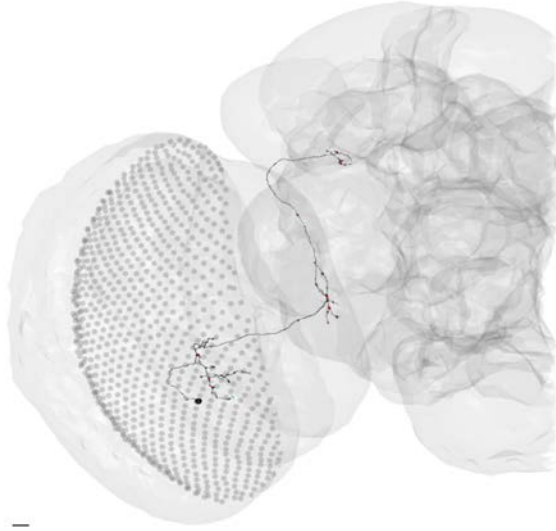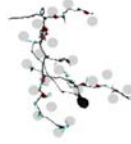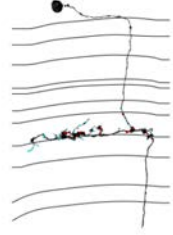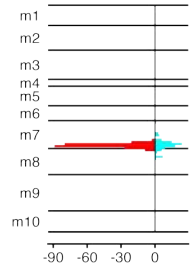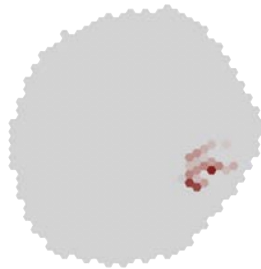

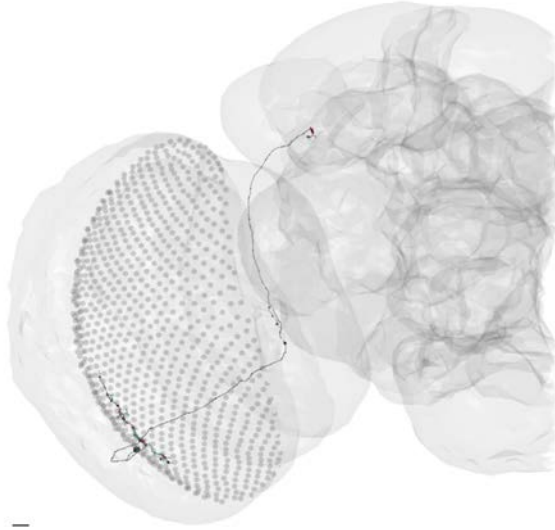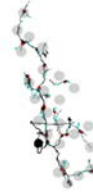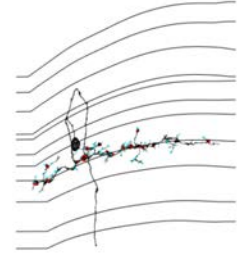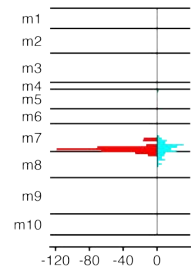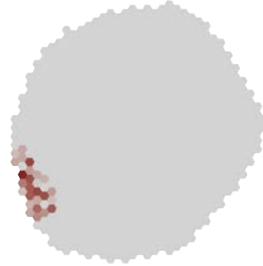

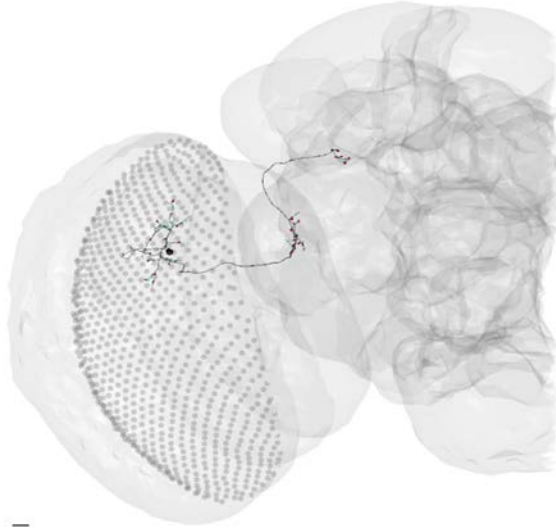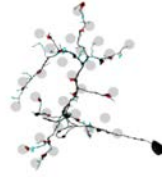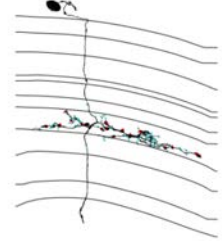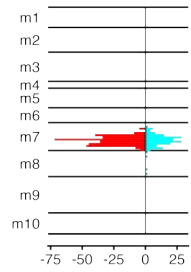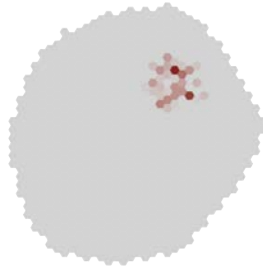

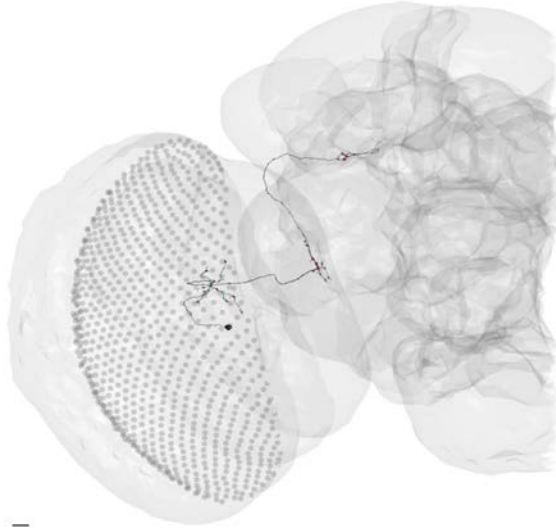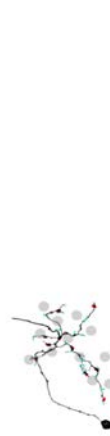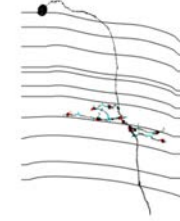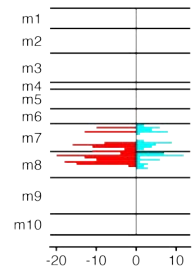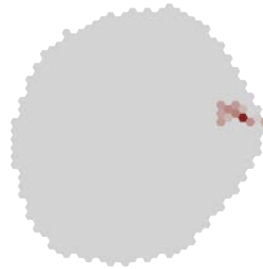

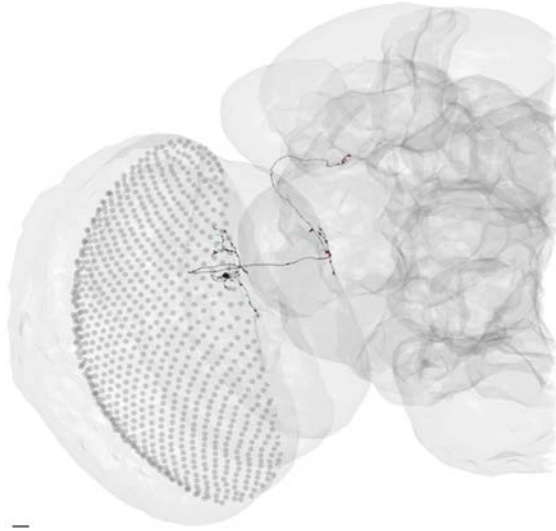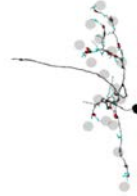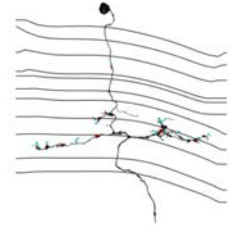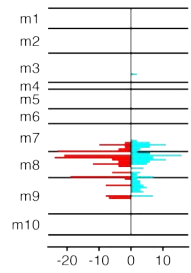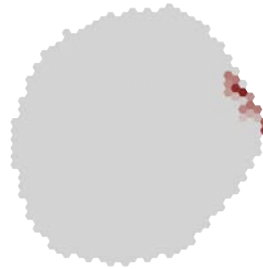

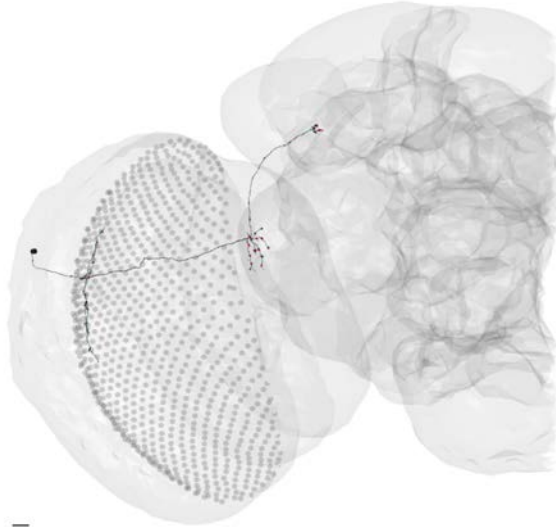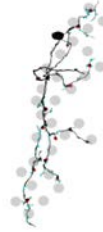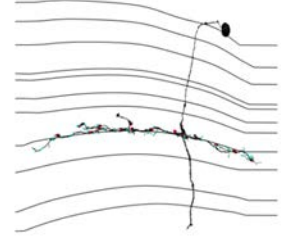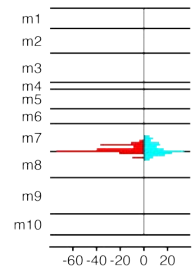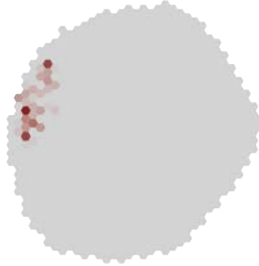

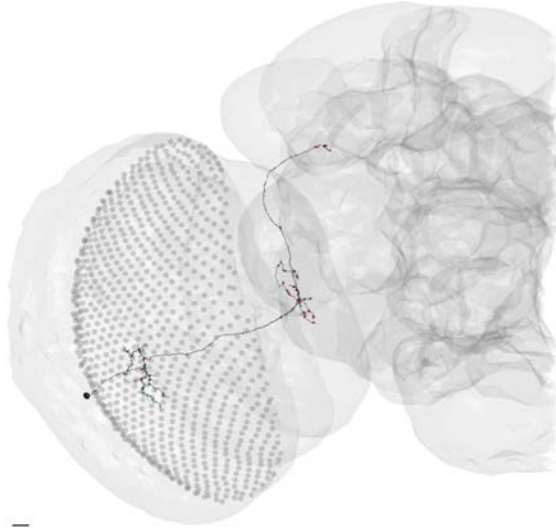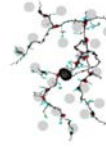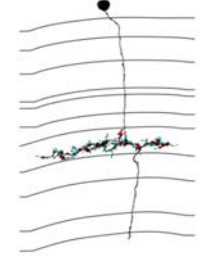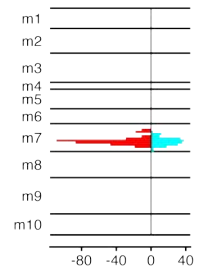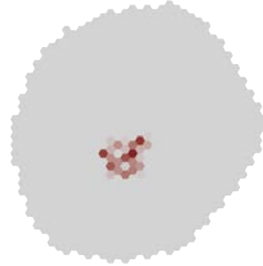

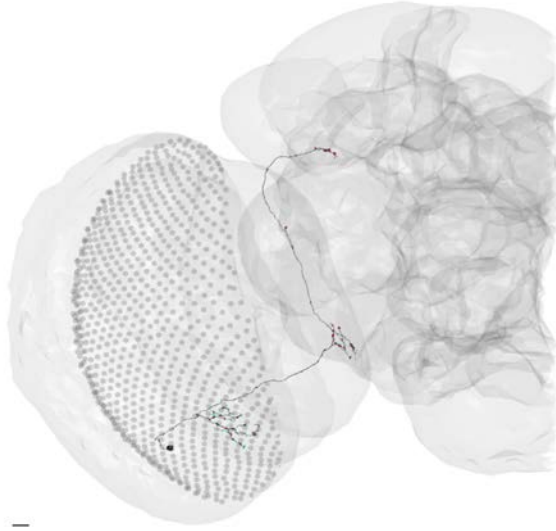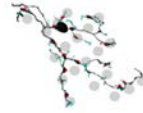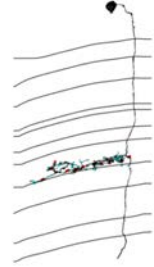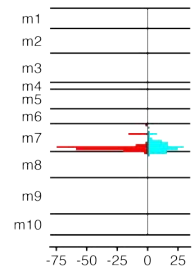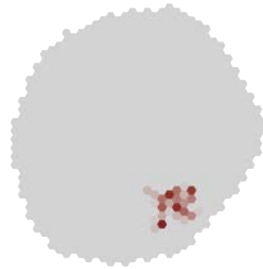

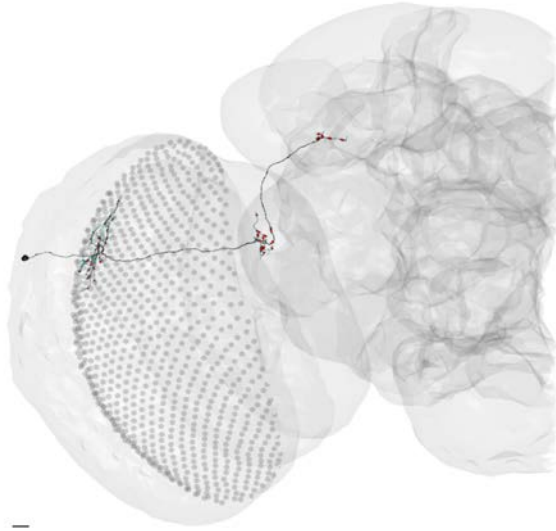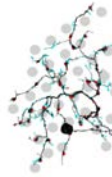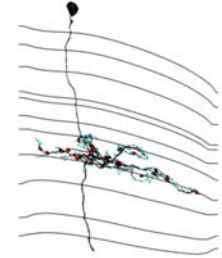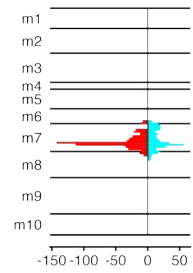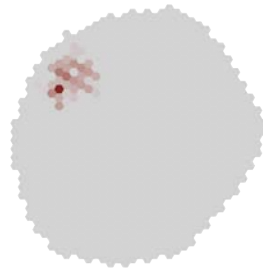

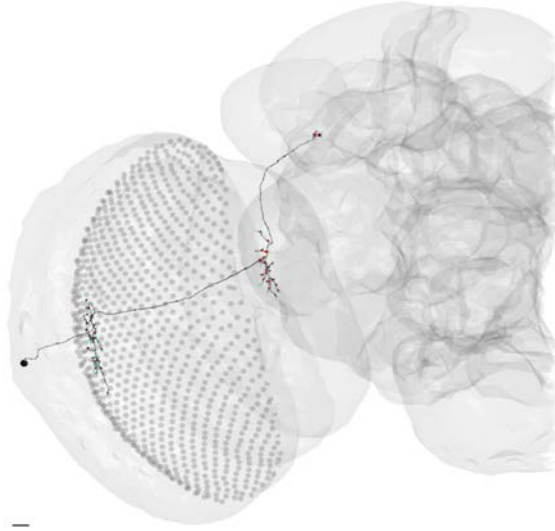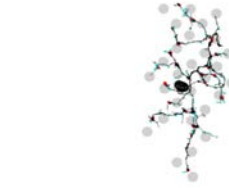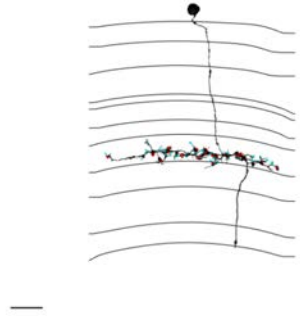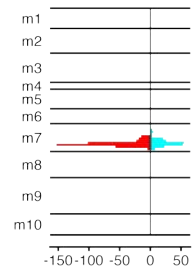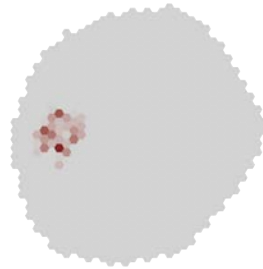

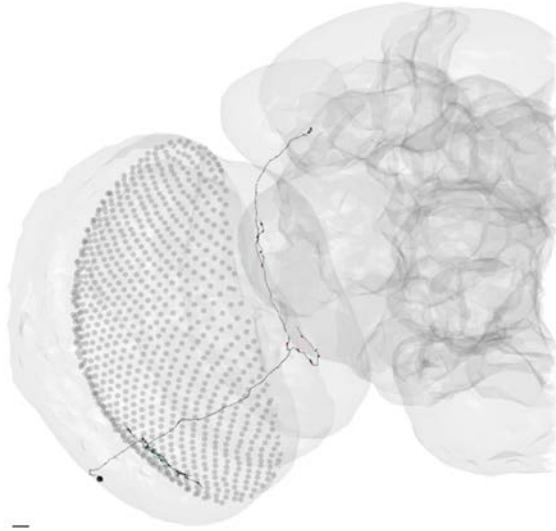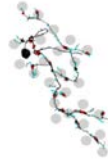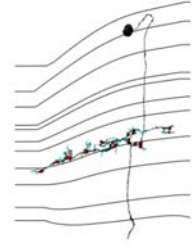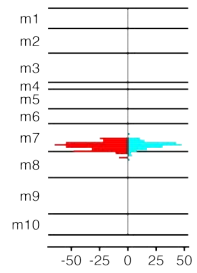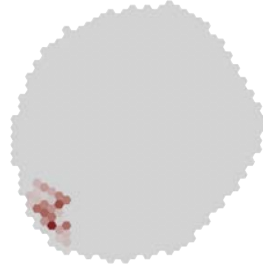

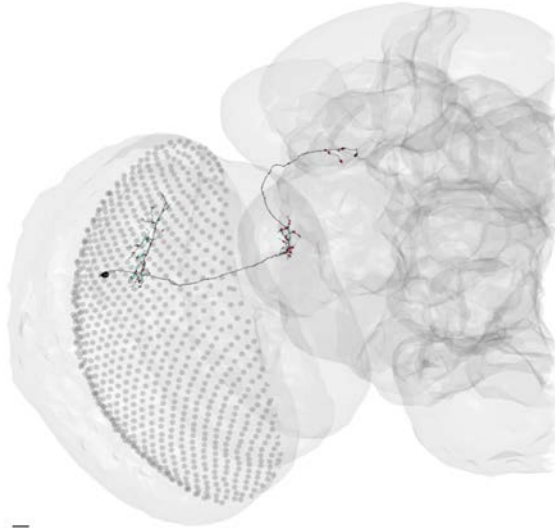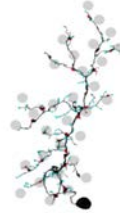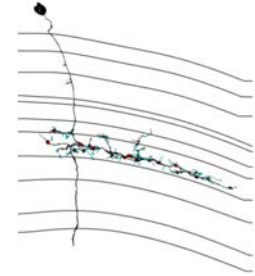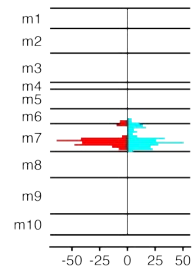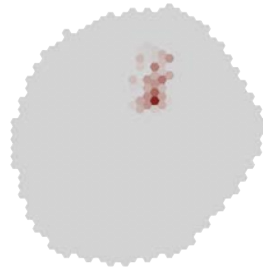

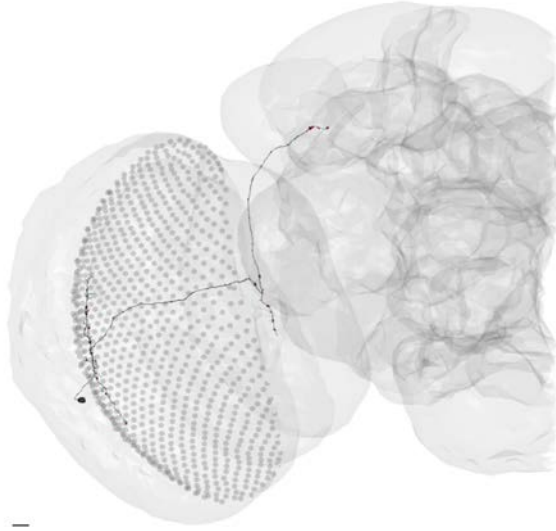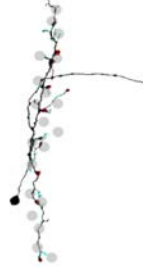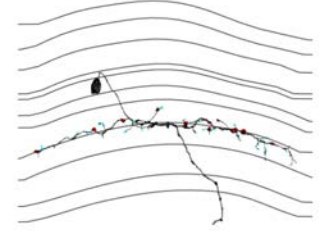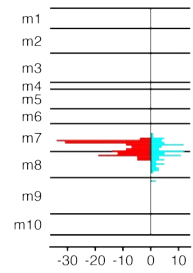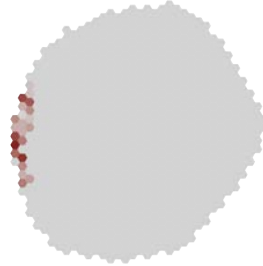

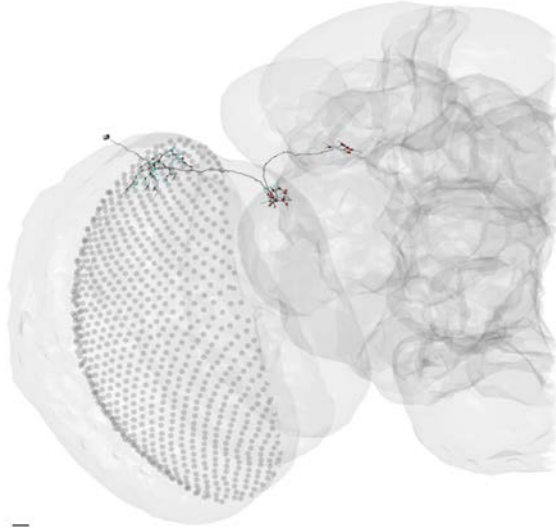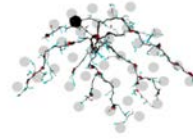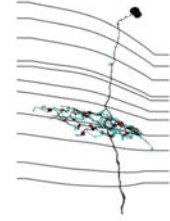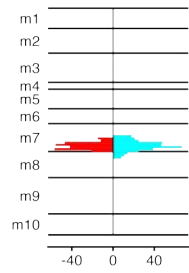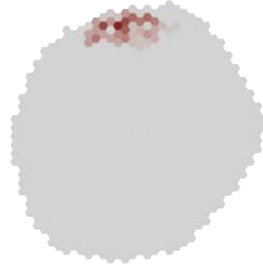

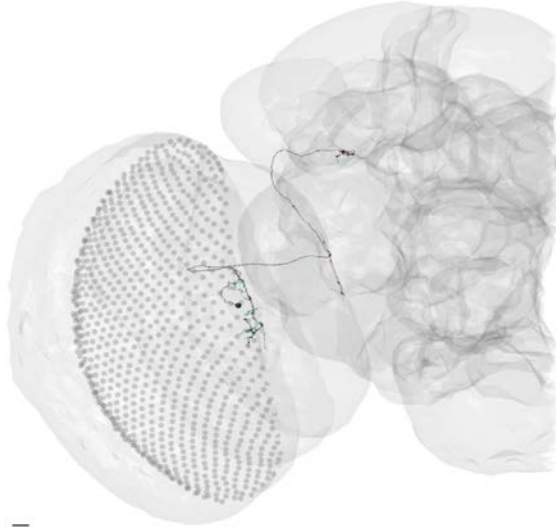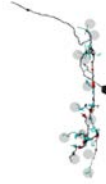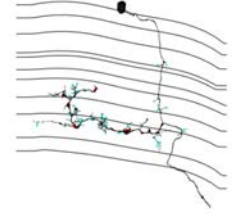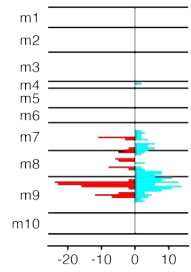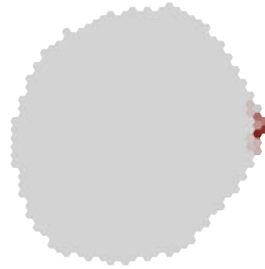

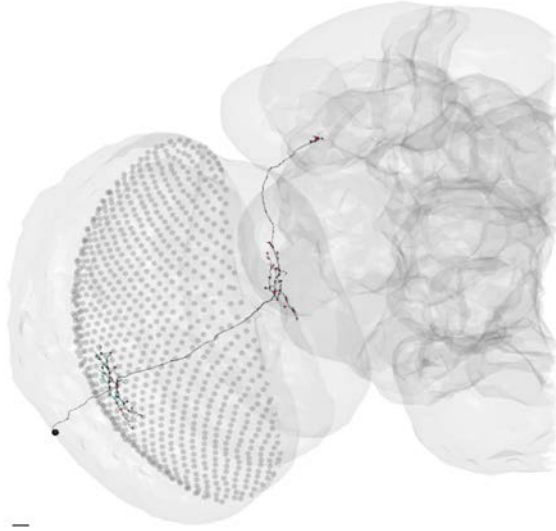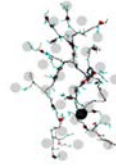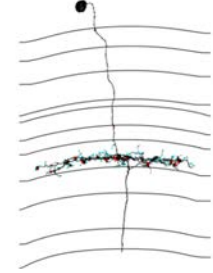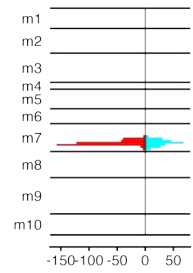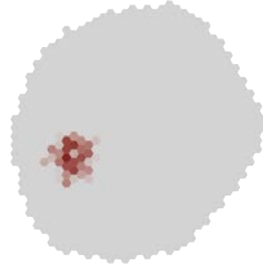

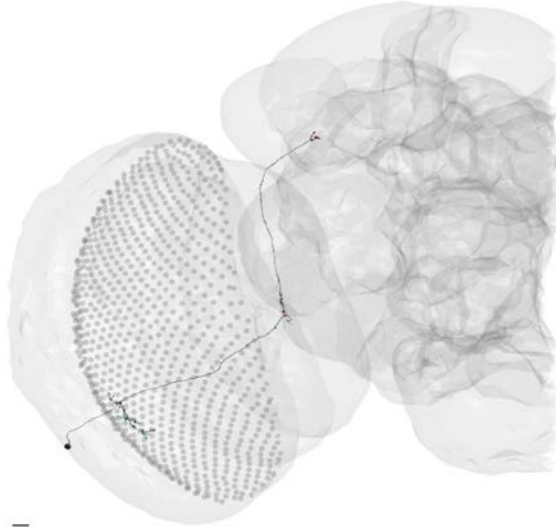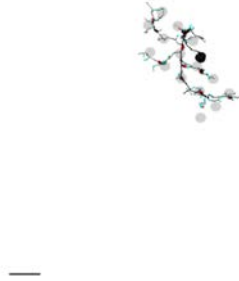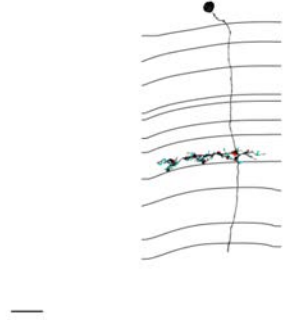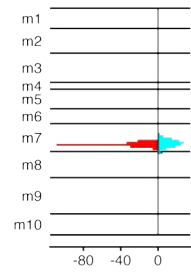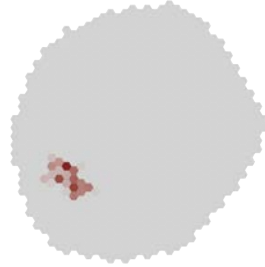

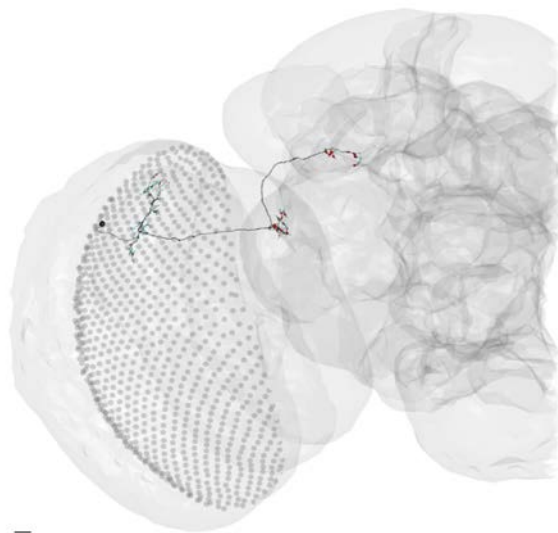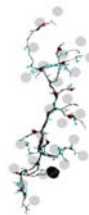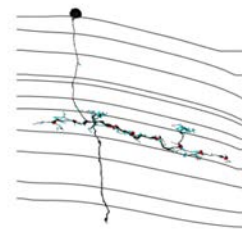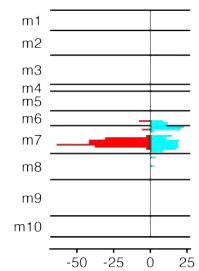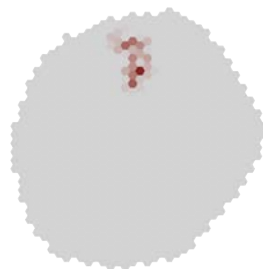

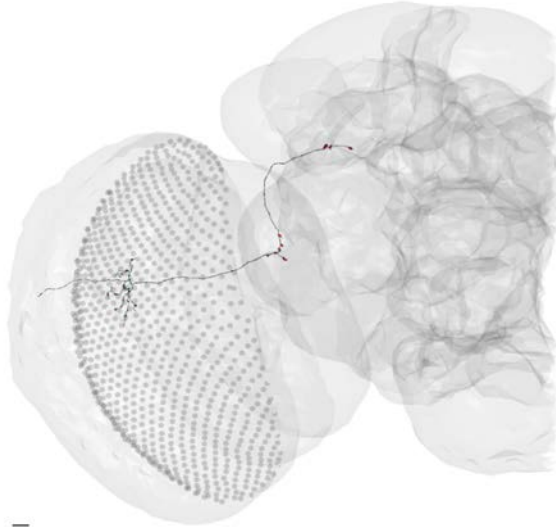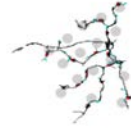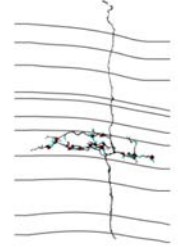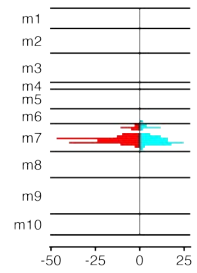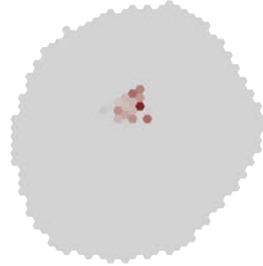

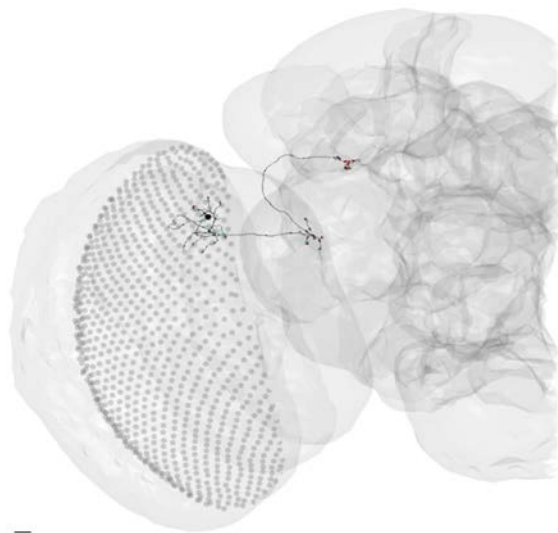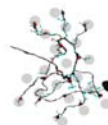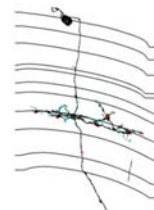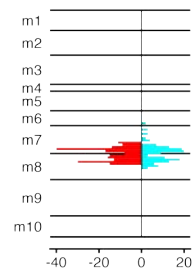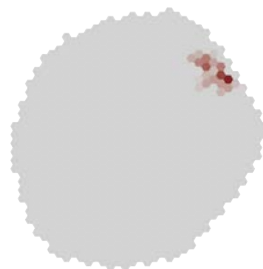

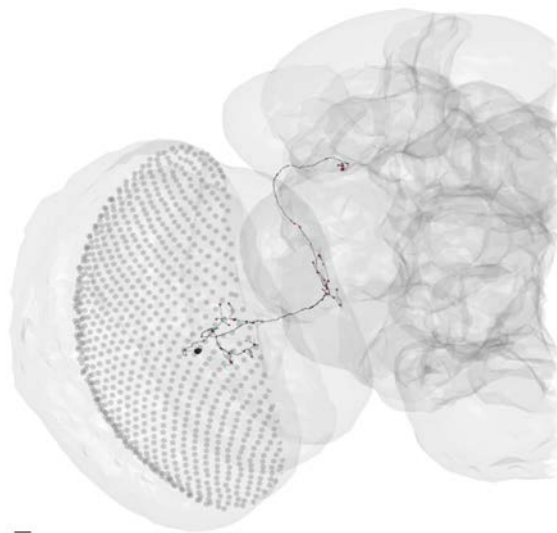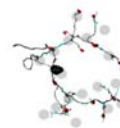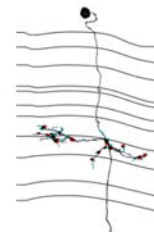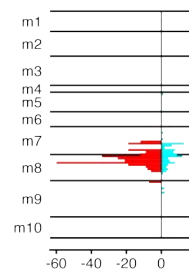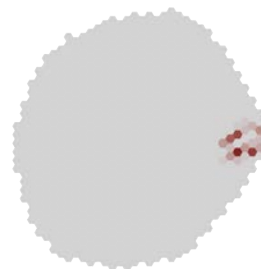

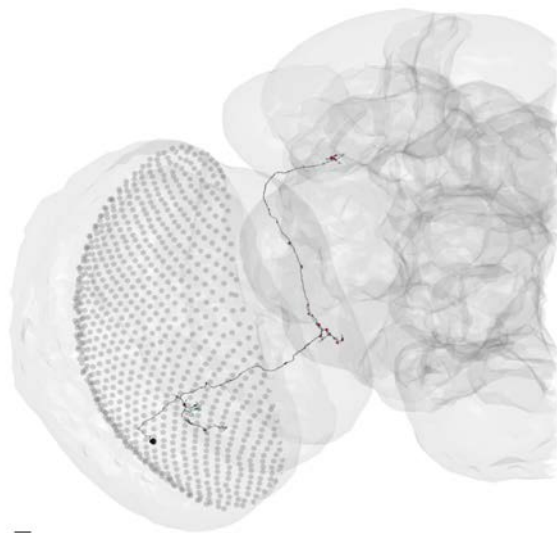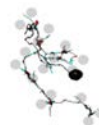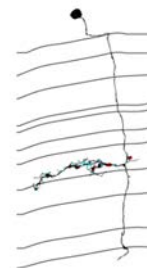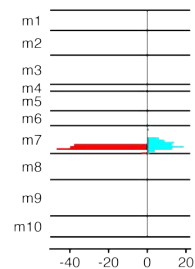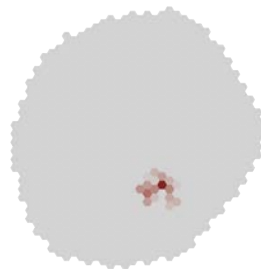

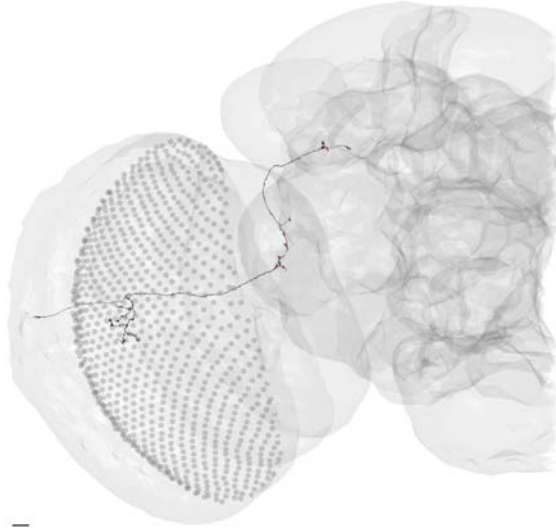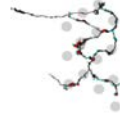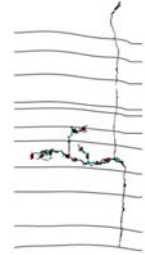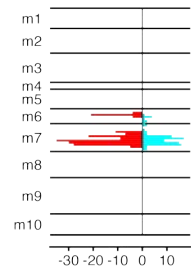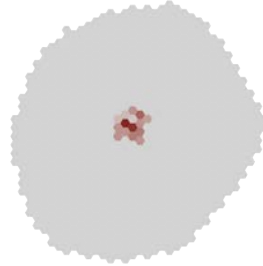

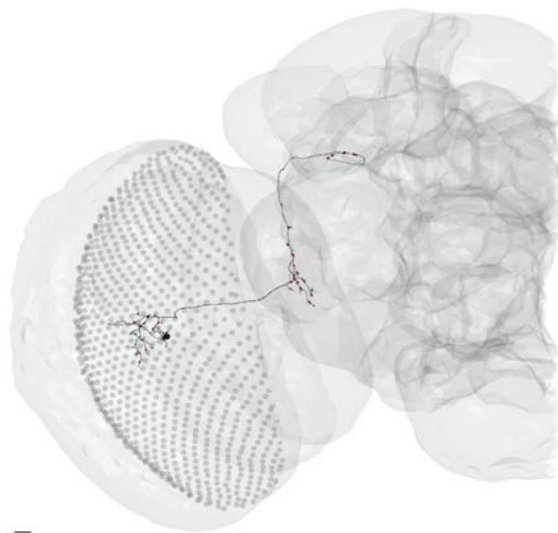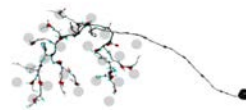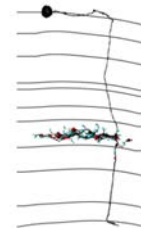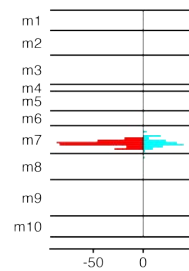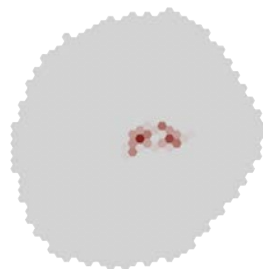

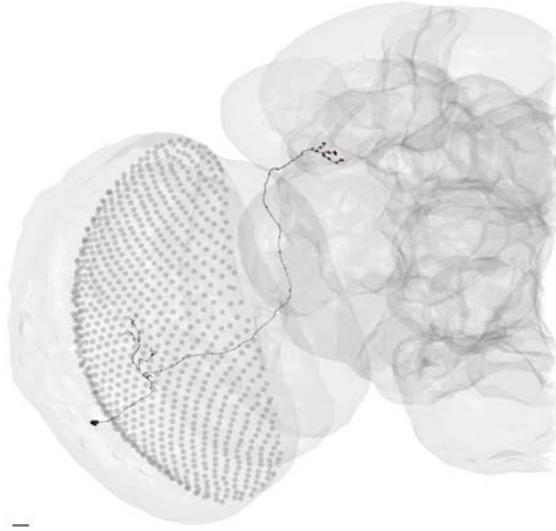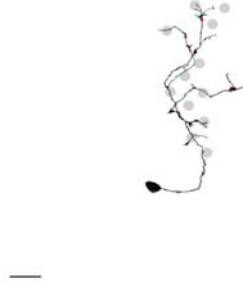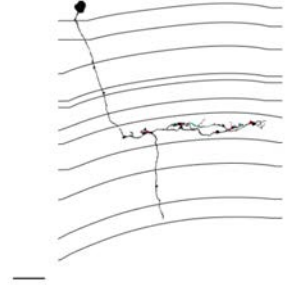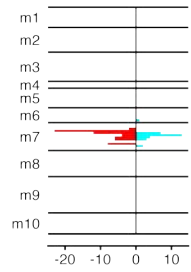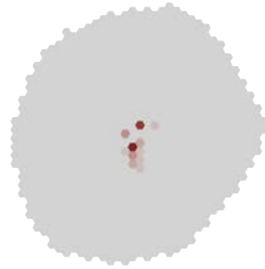

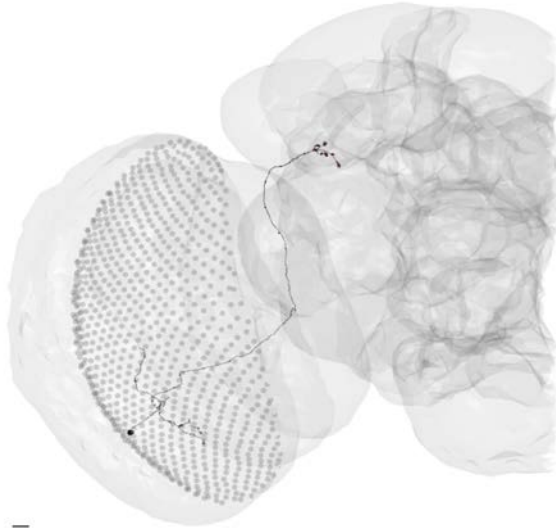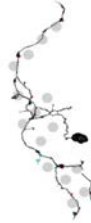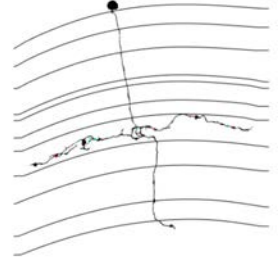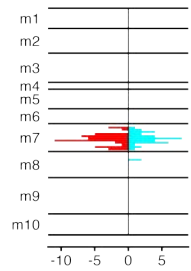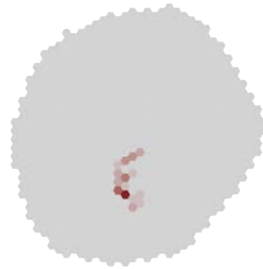

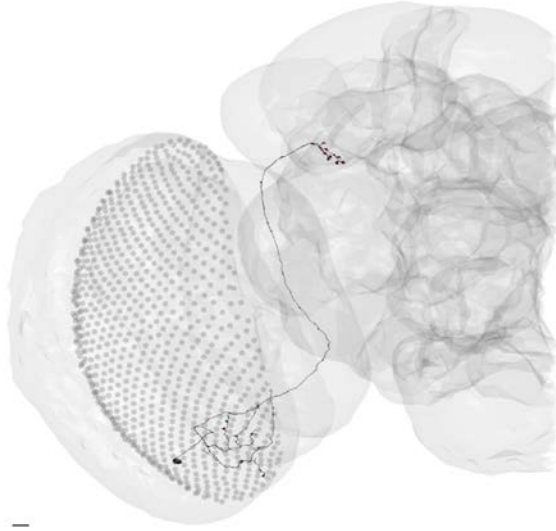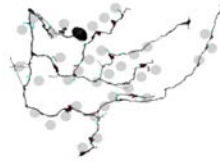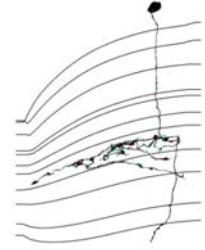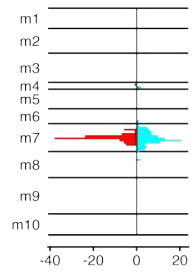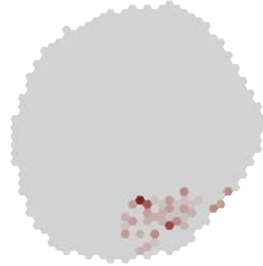

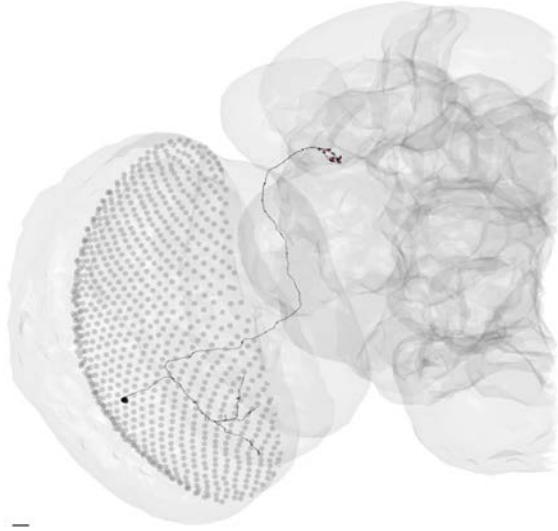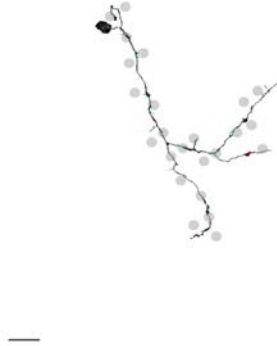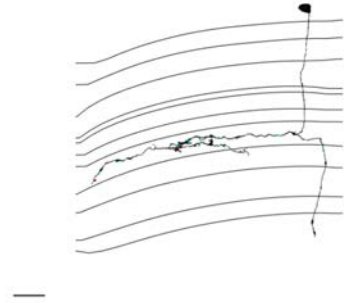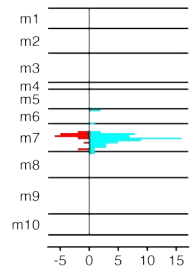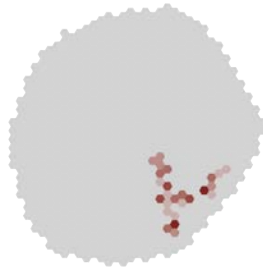

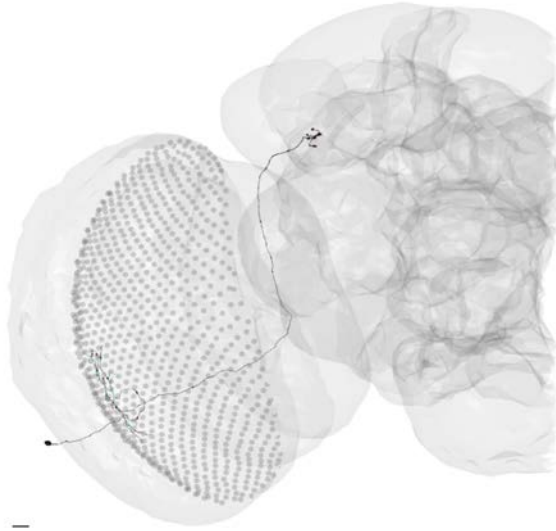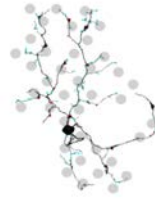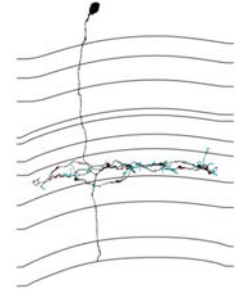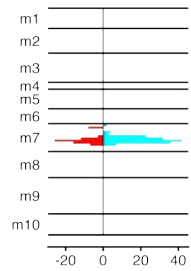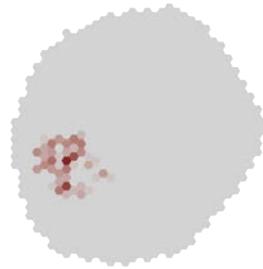

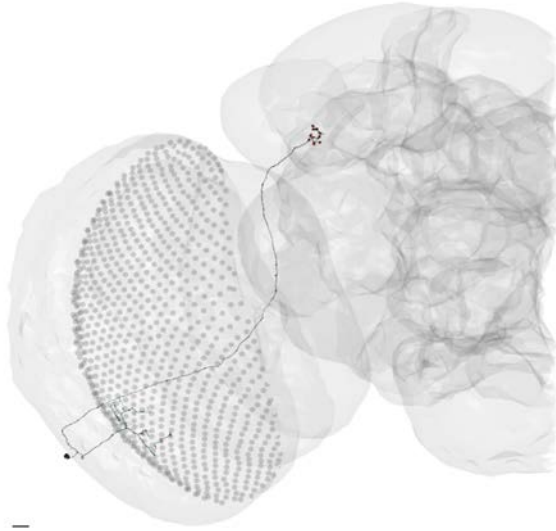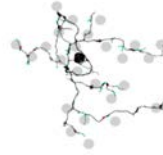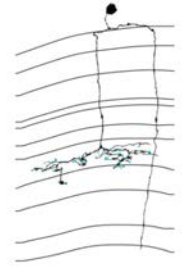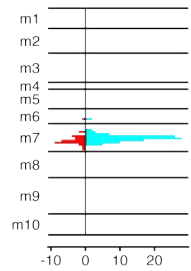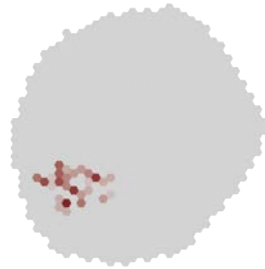

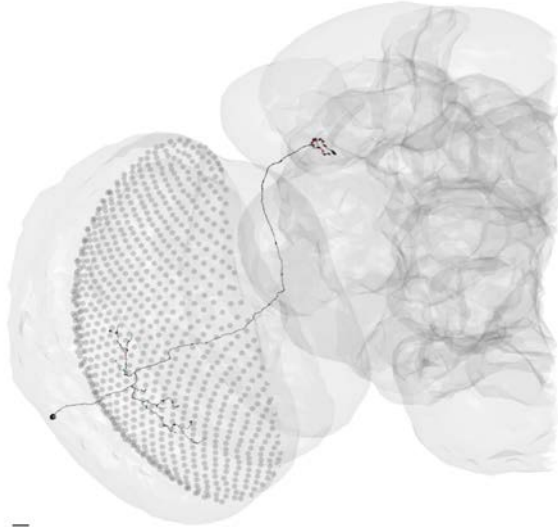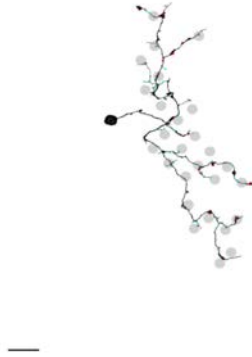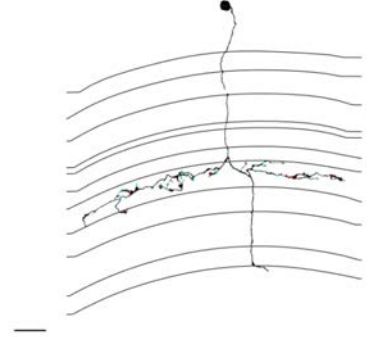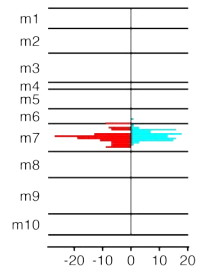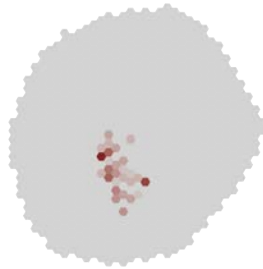

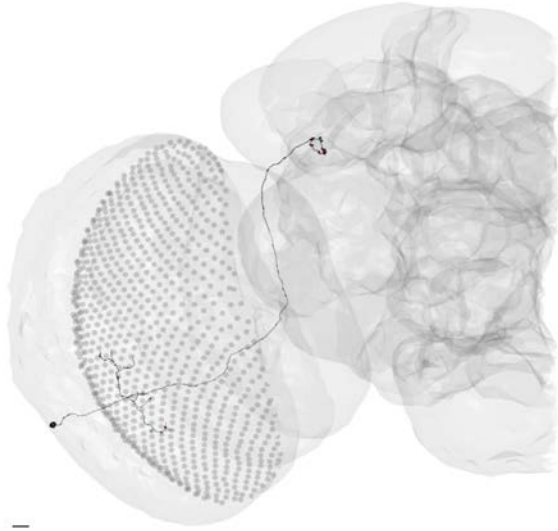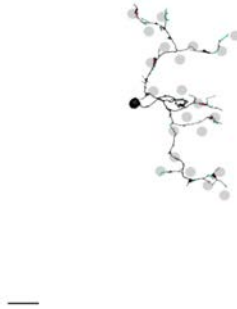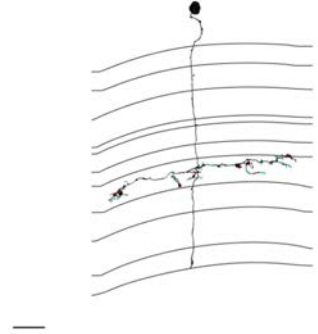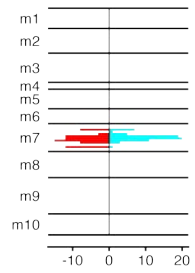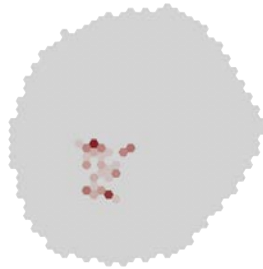

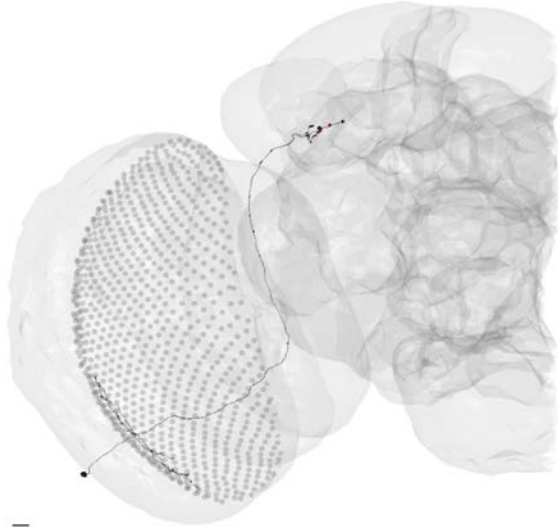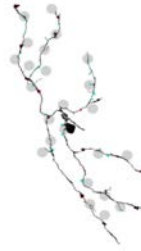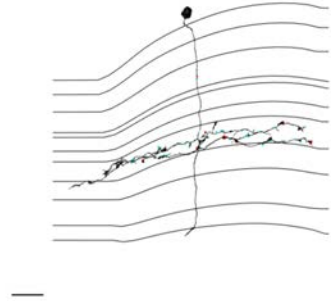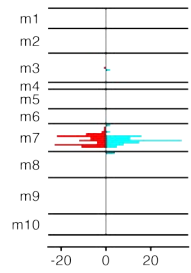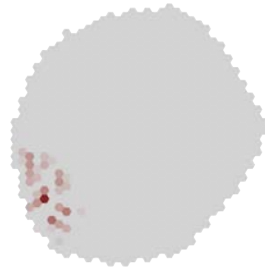

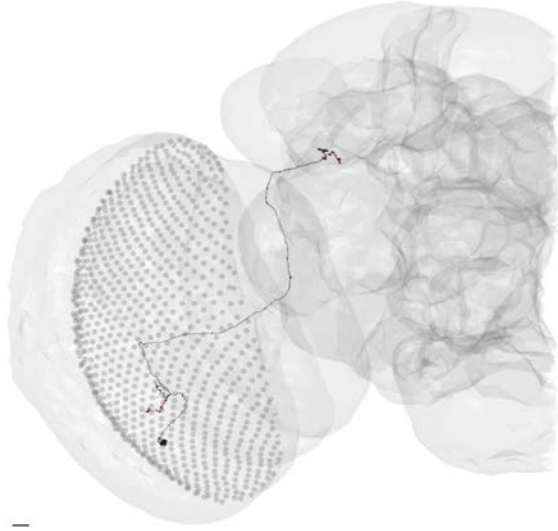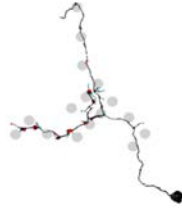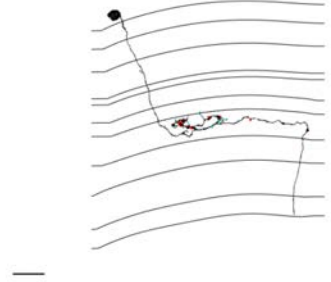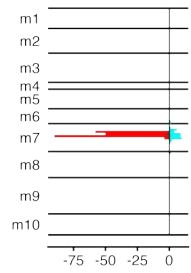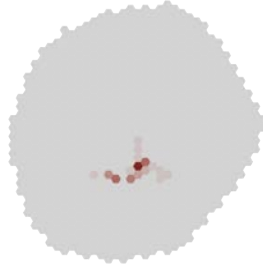

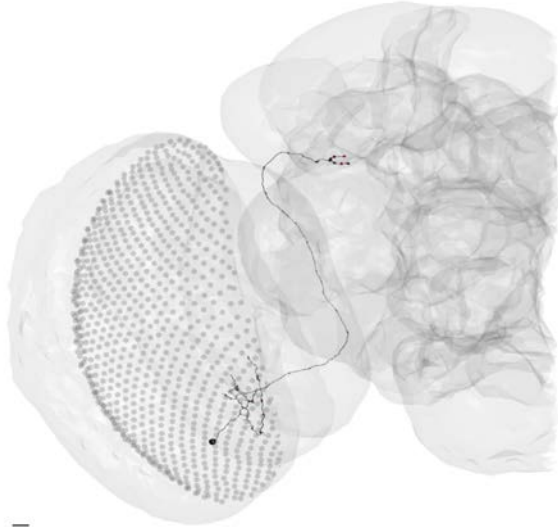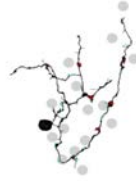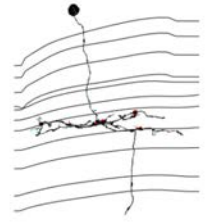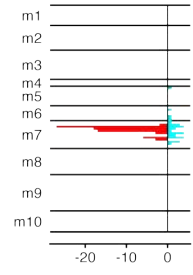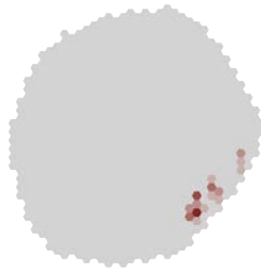

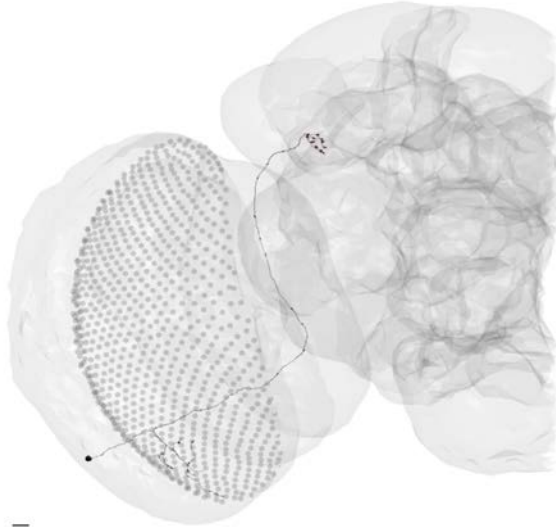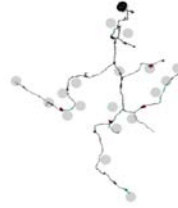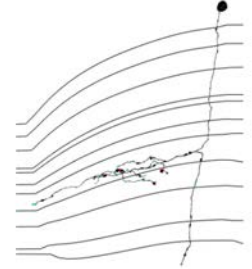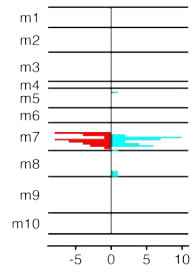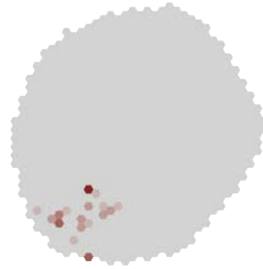

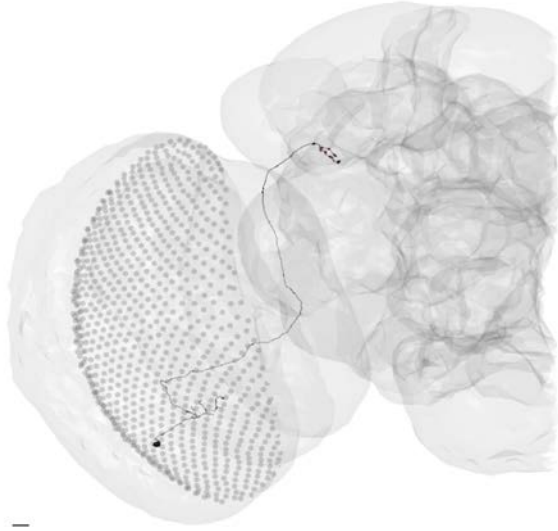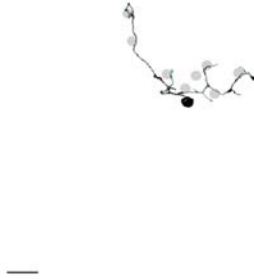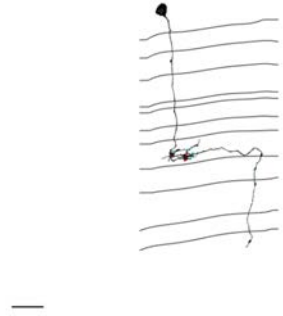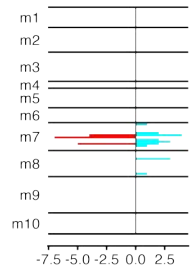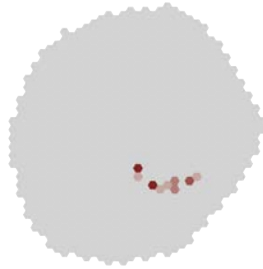

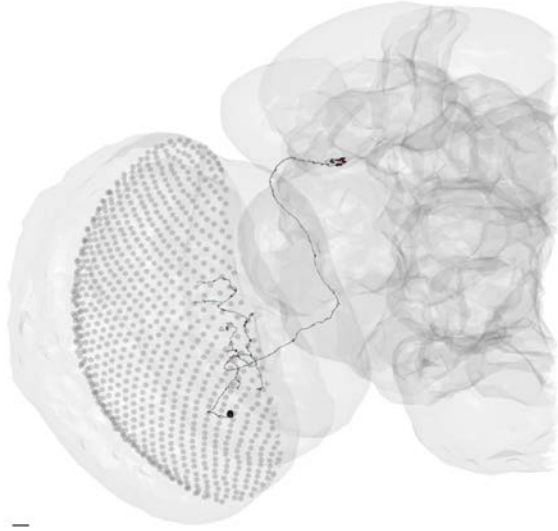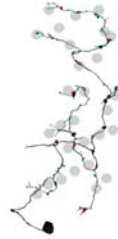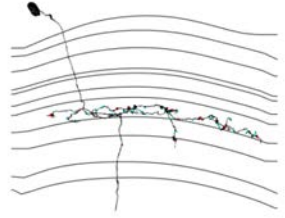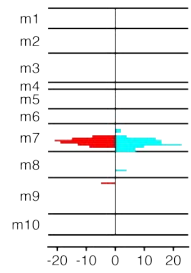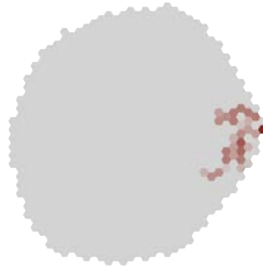

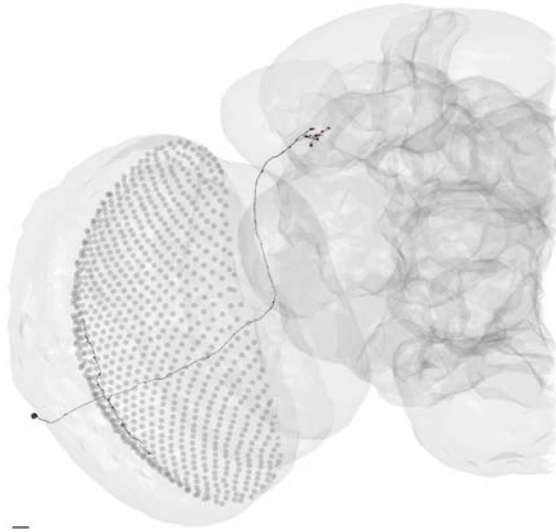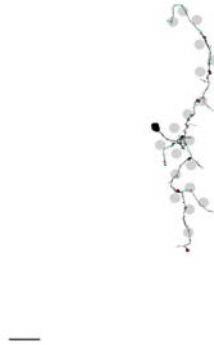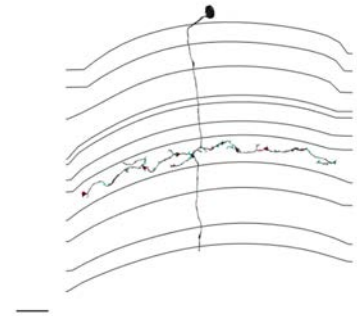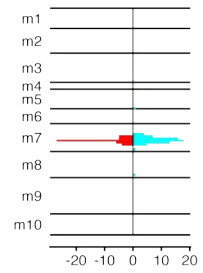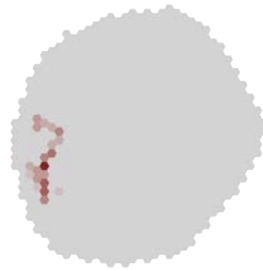

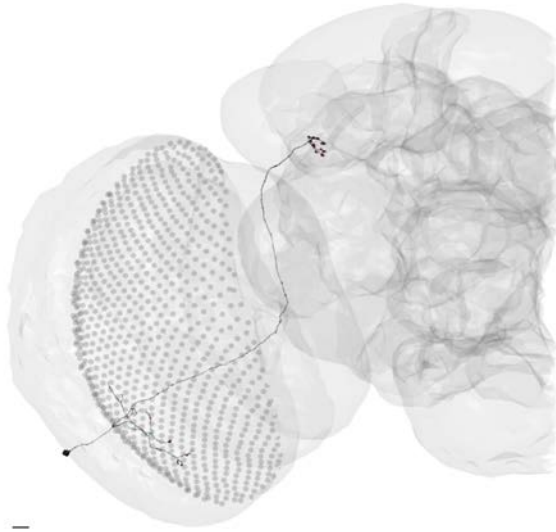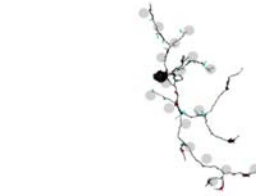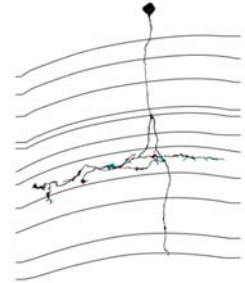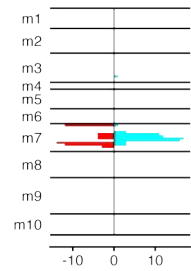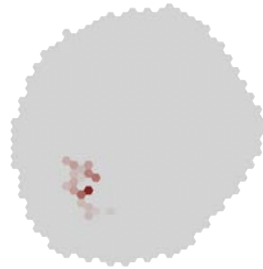

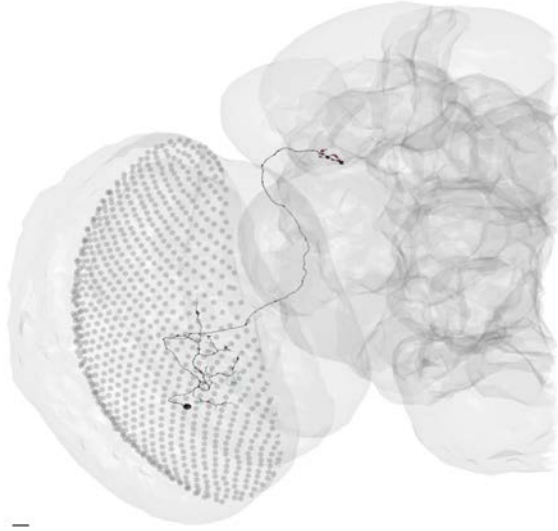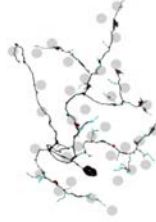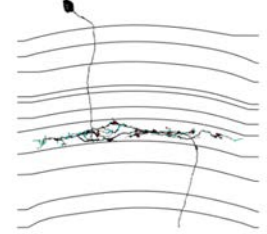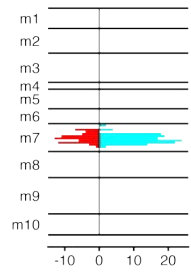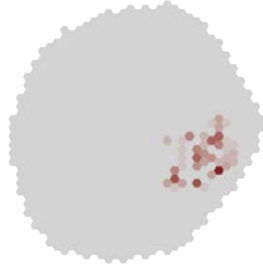

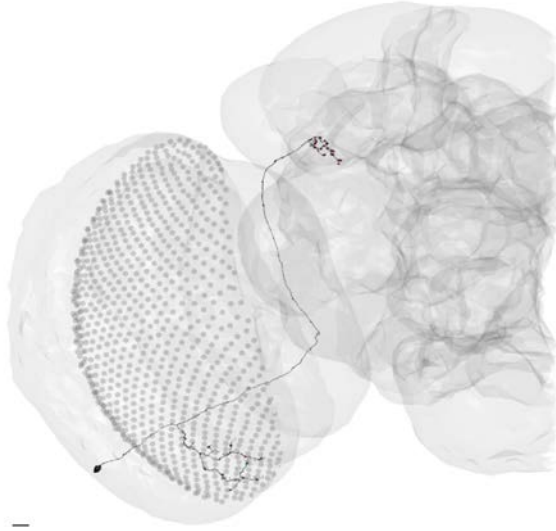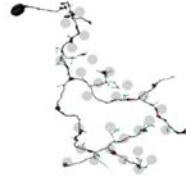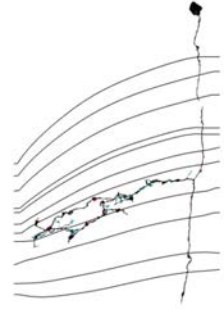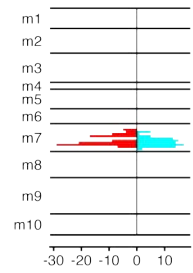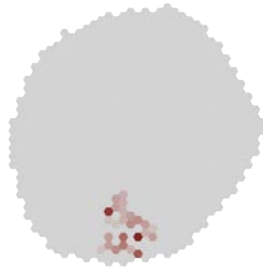

Supplement: Supplementary file 3 — A gallery of all MeTu_R neurons and their receptive fields. This file contains information on all right MeTu neurons (n=441). Each page has a render of the MeTu from an anterior view in the brain, a view from the top with its relevant medulla columns, a view from the side with medulla layers, locations of its presynaptic (red) and postsynaptic (cyan) connections, and its putative receptive field. At the top is a label of the MeTu subtype and its FlyWire ID during materialization 783. [file 41586_2024_7967_MOESM3_ESM.pdf]
