# Supplementary material for: Connectomic reconstruction predicts visual features used for navigation
Source: Nature. 2024 Oct 2;634(8032):181–90. doi: 10.1038/s41586-024-07967-z (PMC11446847; doi:10.1038/s41586-024-07967-z)
Supplement: Supplementary file 4 — A gallery of all ER_R putative receptive fields. Each page contains a collection of right ring neurons of a given type, and shows their putative receptive fields. Included are their types and FlyWire IDs during materialization 783. [file 41586_2024_7967_MOESM4_ESM.pdf]

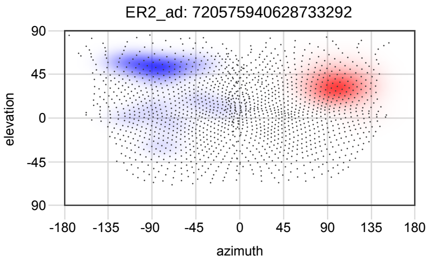
ER2_ad: 720575940628733292
elevation
azimuth
90
45
0
-45
90
-180
-135
-90
-45
0
45
90
135
180
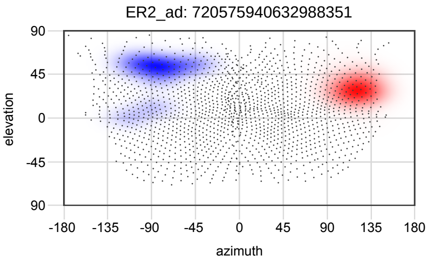
ER2_ad: 720575940632988351
elevation
azimuth
90
45
0
-45
90
-180
-135
-90
-45
0
45
90
135
180
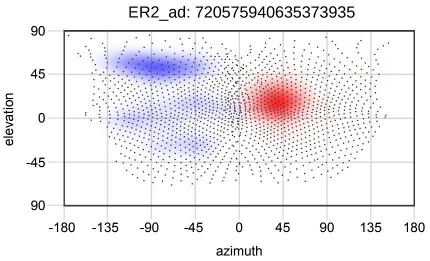
ER2_ad: 720575940635373935
elevation
azimuth
90
45
0
-45
90
-180
-135
-90
-45
0
45
90
135
180

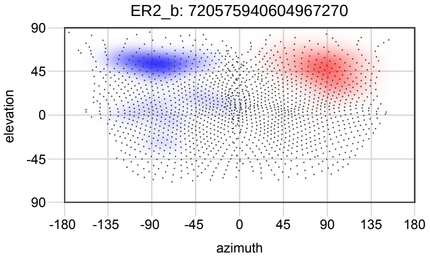
ER2_b: 720575940604967270
elevation
90
45
0
-45
90
-180 -135 -90 -45 0 45 90 135 180
azimuth

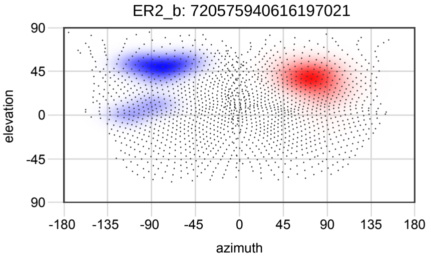
ER2_b: 720575940616197021
elevation
90
45
0
-45
90
-180 -135 -90 -45 0 45 90 135 180
azimuth

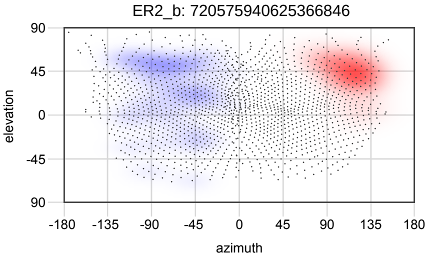
ER2_b: 720575940625366846
elevation
90
45
0
-45
90
-180 -135 -90 -45 0 45 90 135 180
azimuth

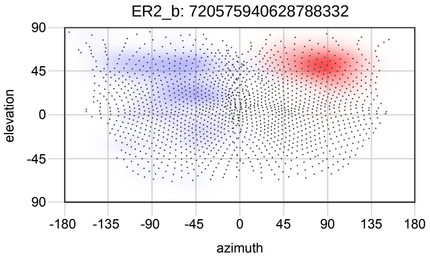
ER2_b: 720575940628788332
elevation
90
45
0
-45
90
-180 -135 -90 -45 0 45 90 135 180
azimuth

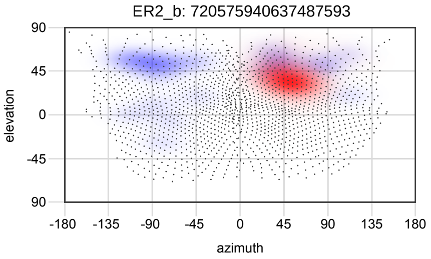
ER2_b: 720575940637487593
elevation
90
45
0
-45
90
-180 -135 -90 -45 0 45 90 135 180
azimuth

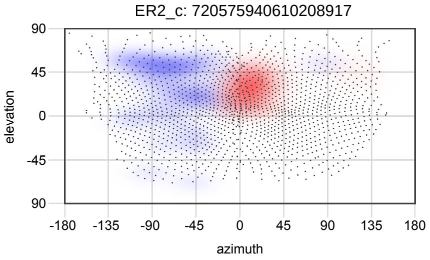
ER2_c: 720575940610208917
90 45 0 -45 90
elevation
-180 -135 -90 -45 0 45 90 135 180
azimuth

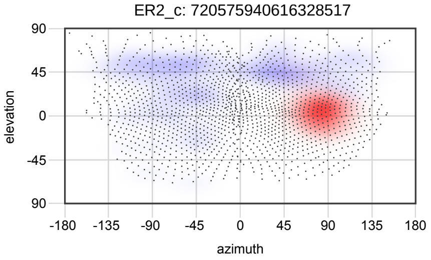
ER2_c: 720575940616328517
90 45 0 -45 90
elevation
-180 -135 -90 -45 0 45 90 135 180
azimuth

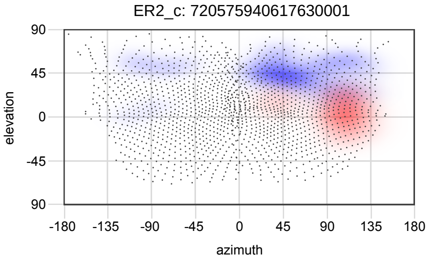
ER2_c: 720575940617630001
90 45 0 -45 90
elevation
-180 -135 -90 -45 0 45 90 135 180
azimuth

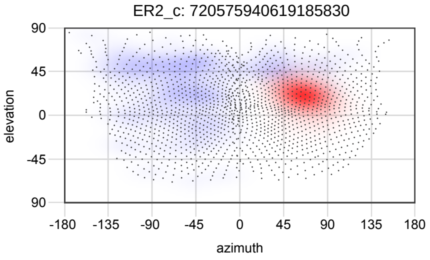
ER2_c: 720575940619185830
90 45 0 -45 90
elevation
-180 -135 -90 -45 0 45 90 135 180
azimuth

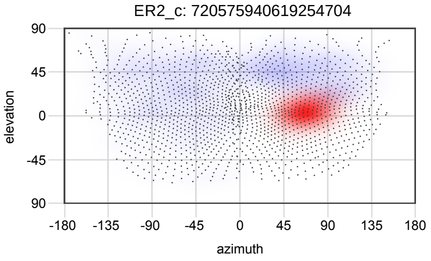
ER2_c: 720575940619254704
90 45 0 -45 90
elevation
-180 -135 -90 -45 0 45 90 135 180
azimuth

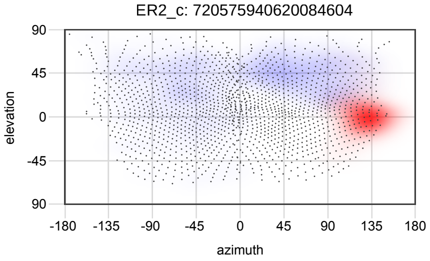
ER2_c: 720575940620084604
90 45 0 -45 90
elevation
-180 -135 -90 -45 0 45 90 135 180
azimuth

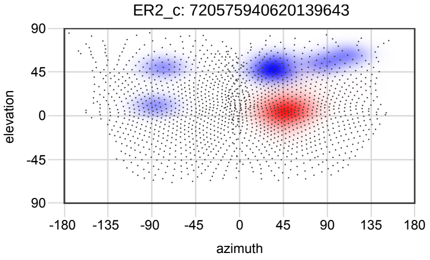
ER2_c: 720575940620139643
90 45 0 -45 90
elevation
-180 -135 -90 -45 0 45 90 135 180
azimuth

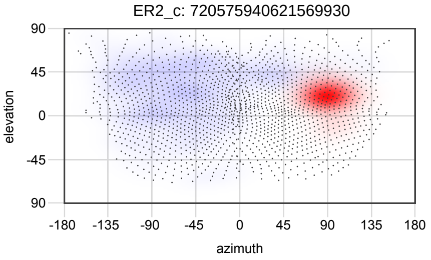
ER2_c: 720575940621569930
90 45 0 -45 90
elevation
-180 -135 -90 -45 0 45 90 135 180
azimuth

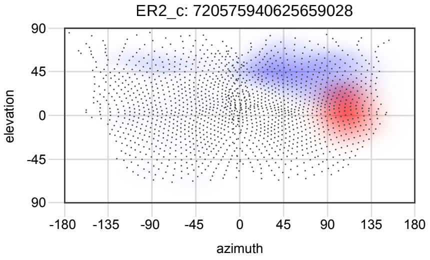
ER2_c: 720575940625659028
90 45 0 -45 90
elevation
-180 -135 -90 -45 0 45 90 135 180
azimuth

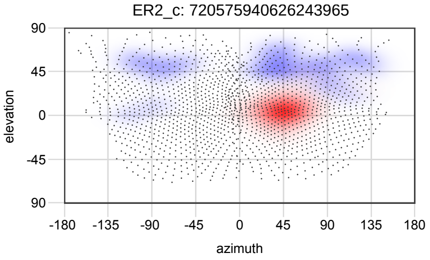
ER2_c: 720575940626243965
90 45 0 -45 90
elevation
-180 -135 -90 -45 0 45 90 135 180
azimuth

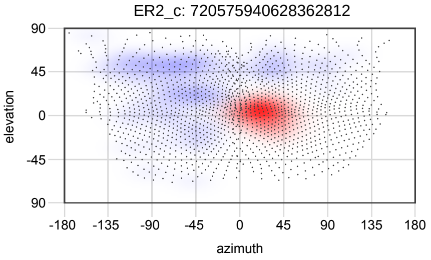
ER2_c: 720575940628362812
90 45 0 -45 90
elevation
-180 -135 -90 -45 0 45 90 135 180
azimuth

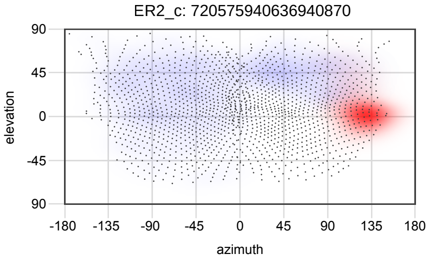
ER2_c: 720575940636940870
90 45 0 -45 90
elevation
-180 -135 -90 -45 0 45 90 135 180
azimuth

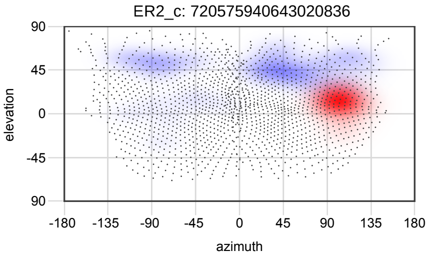
ER2_c: 720575940643020836
90 45 0 -45 90
elevation
-180 -135 -90 -45 0 45 90 135 180
azimuth

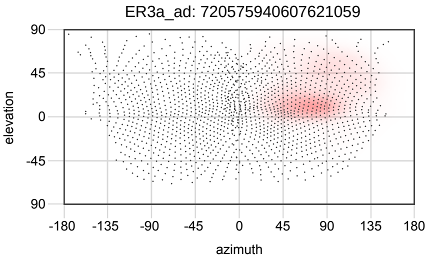
ER3a_ad: 720575940607621059
90
45
0
-45
90
elevation
-180 -135 -90 -45 0 45 90 135 180
azimuth

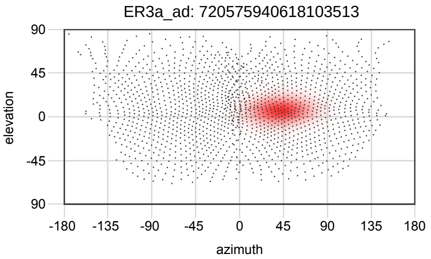
ER3a_ad: 720575940618103513
90
45
0
-45
90
elevation
-180 -135 -90 -45 0 45 90 135 180
azimuth

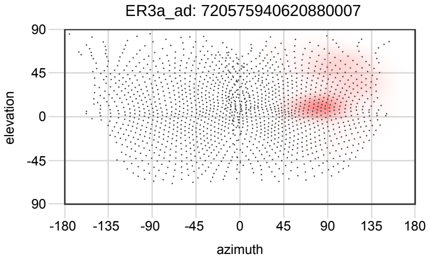
ER3a_ad: 720575940620880007
90
45
0
-45
90
elevation
-180 -135 -90 -45 0 45 90 135 180
azimuth

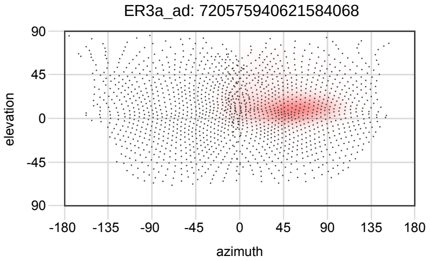
ER3a_ad: 720575940621584068
90
45
0
-45
90
elevation
-180 -135 -90 -45 0 45 90 135 180
azimuth

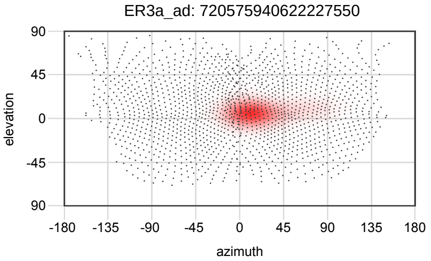
ER3a_ad: 720575940622227550
90
45
0
-45
90
elevation
-180 -135 -90 -45 0 45 90 135 180
azimuth

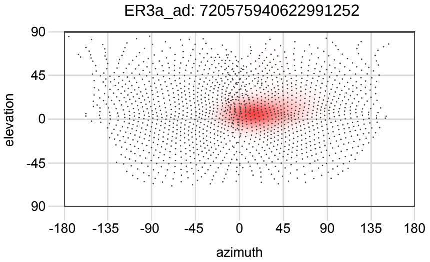
ER3a_ad: 720575940622991252
90
45
0
-45
90
elevation
-180 -135 -90 -45 0 45 90 135 180
azimuth

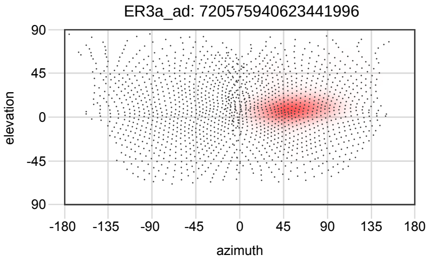
ER3a_ad: 720575940623441996
90
45
0
-45
90
elevation
-180 -135 -90 -45 0 45 90 135 180
azimuth

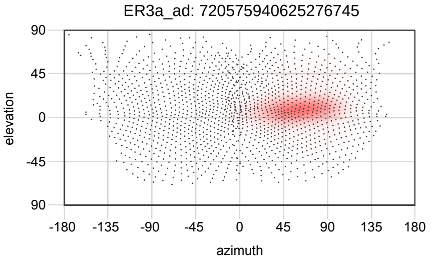
ER3a_ad: 720575940625276745
90
45
0
-45
90
elevation
-180 -135 -90 -45 0 45 90 135 180
azimuth

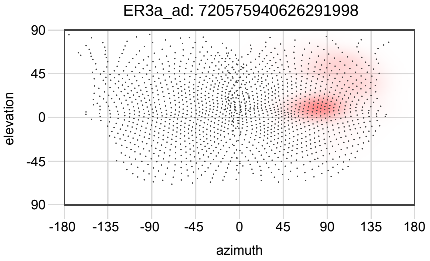
ER3a_ad: 720575940626291998
90
45
0
-45
90
elevation
-180 -135 -90 -45 0 45 90 135 180
azimuth

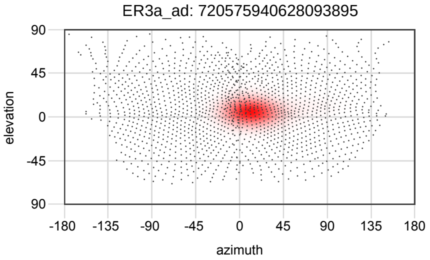
ER3a_ad: 720575940628093895
90
45
0
-45
90
elevation
-180 -135 -90 -45 0 45 90 135 180
azimuth

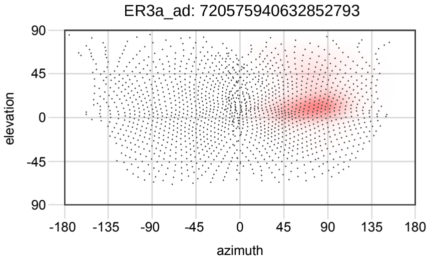
ER3a_ad: 720575940632852793
90
45
0
-45
90
elevation
-180 -135 -90 -45 0 45 90 135 180
azimuth

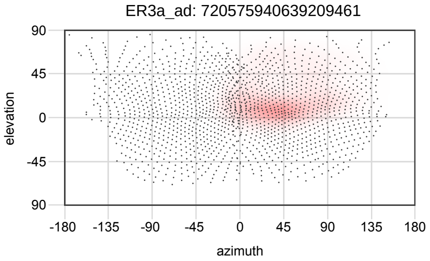
ER3a_ad: 720575940639209461
90
45
0
-45
90
elevation
-180 -135 -90 -45 0 45 90 135 180
azimuth

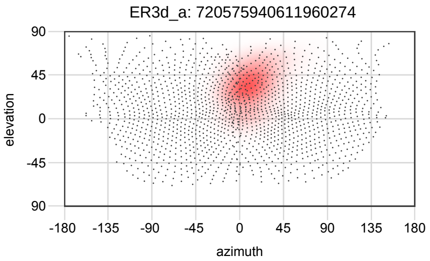
ER3d_a: 720575940611960274
elevation
90 45 0 -45 90
-180 -135 -90 -45 0 45 90 135 180
azimuth

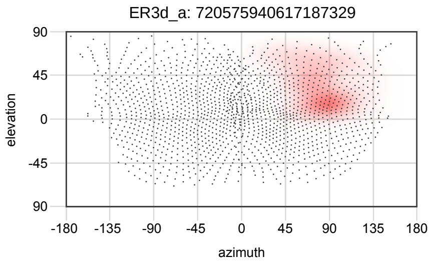
ER3d_a: 720575940617187329
elevation
90 45 0 -45 90
-180 -135 -90 -45 0 45 90 135 180
azimuth

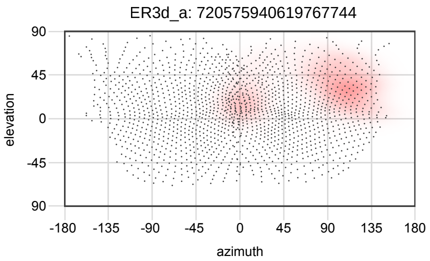
ER3d_a: 720575940619767744
elevation
90 45 0 -45 90
-180 -135 -90 -45 0 45 90 135 180
azimuth

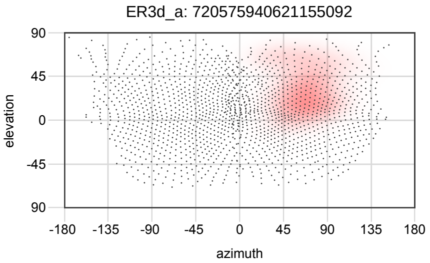
ER3d_a: 720575940621155092
elevation
90 45 0 -45 90
-180 -135 -90 -45 0 45 90 135 180
azimuth

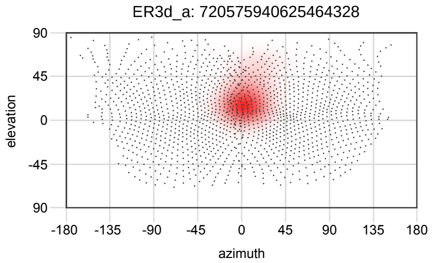
ER3d_a: 720575940625464328
elevation
90 45 0 -45 90
-180 -135 -90 -45 0 45 90 135 180
azimuth

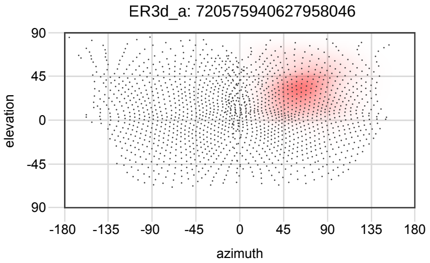
ER3d_a: 720575940627958046
elevation
90 45 0 -45 90
-180 -135 -90 -45 0 45 90 135 180
azimuth

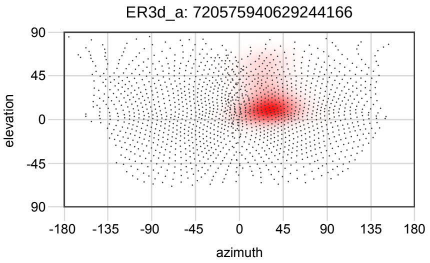
ER3d_a: 720575940629244166
elevation
90 45 0 -45 90
-180 -135 -90 -45 0 45 90 135 180
azimuth

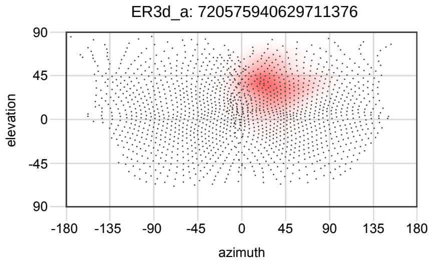
ER3d_a: 720575940629711376
elevation
90 45 0 -45 90
-180 -135 -90 -45 0 45 90 135 180
azimuth

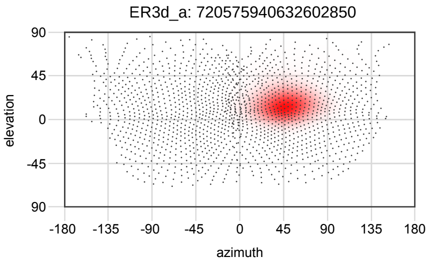
ER3d_a: 720575940632602850
elevation
90 45 0 -45 90
-180 -135 -90 -45 0 45 90 135 180
azimuth

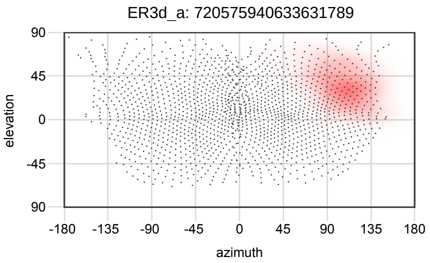
ER3d_a: 720575940633631789
elevation
90 45 0 -45 90
-180 -135 -90 -45 0 45 90 135 180
azimuth

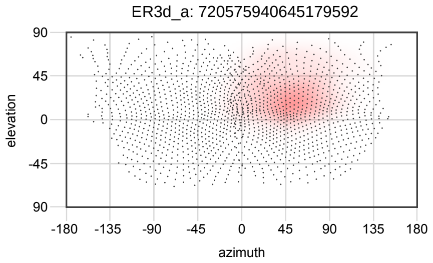
ER3d_a: 720575940645179592
elevation
90 45 0 -45 90
-180 -135 -90 -45 0 45 90 135 180
azimuth

ER3d_b: 720575940616445250

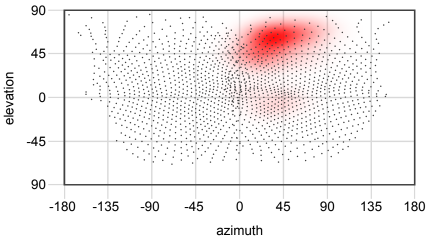

ER3d_b: 720575940619658111

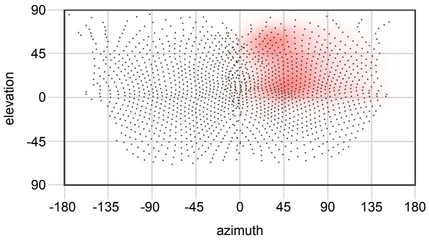

ER3d_b: 720575940625218685

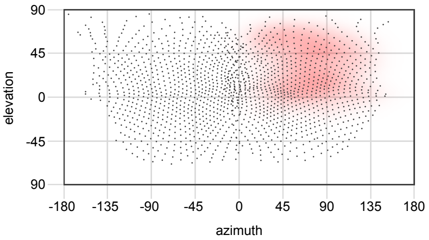

ER3d_b: 720575940626827024

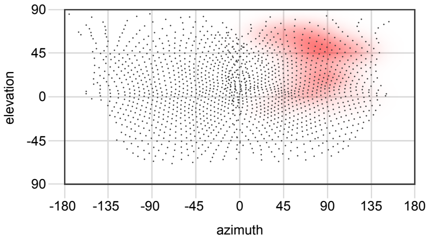

ER3d_b: 720575940627299227

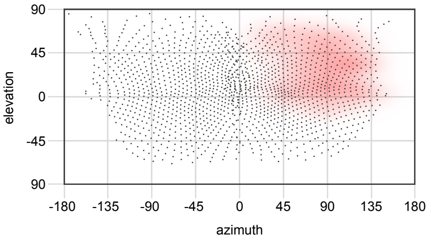

ER3d_b: 720575940627299483

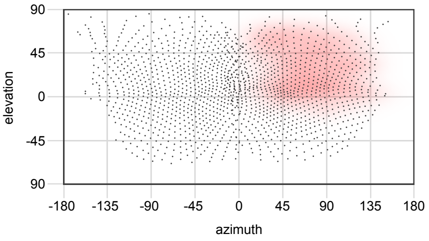

ER3d_b: 720575940630755276

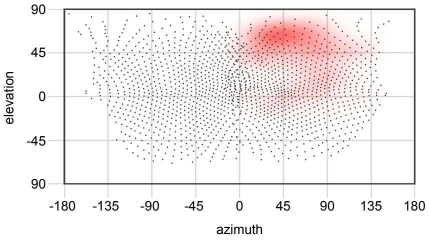

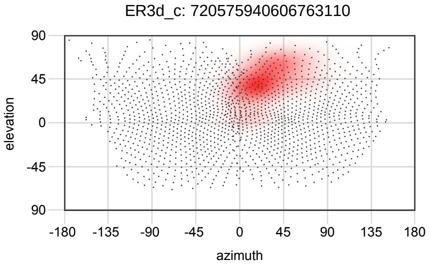
ER3d_c: 720575940606763110
90
45
0
-45
90
elevation
-180 -135 -90 -45 0 45 90 135 180
azimuth

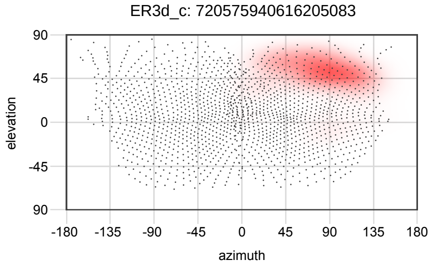
ER3d_c: 720575940616205083
90
45
0
-45
90
elevation
-180 -135 -90 -45 0 45 90 135 180
azimuth

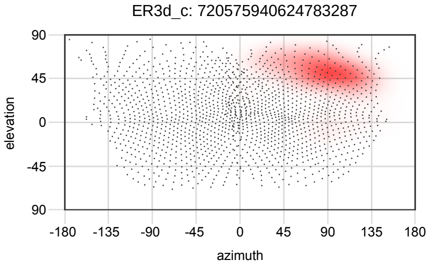
ER3d_c: 720575940624783287
90
45
0
-45
90
elevation
-180 -135 -90 -45 0 45 90 135 180
azimuth

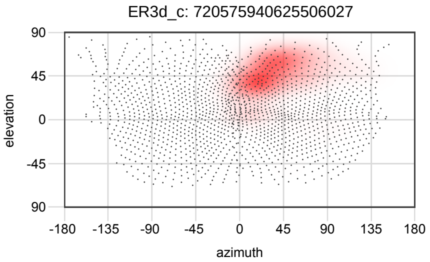
ER3d_c: 720575940625506027
90
45
0
-45
90
elevation
-180 -135 -90 -45 0 45 90 135 180
azimuth

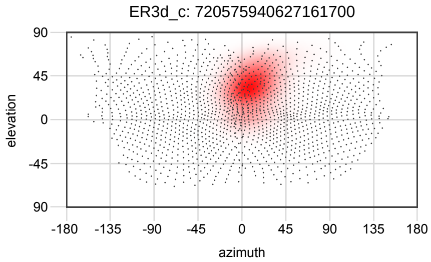
ER3d_c: 720575940627161700
90
45
0
-45
90
elevation
-180 -135 -90 -45 0 45 90 135 180
azimuth

ER3d_d: 720575940606591836

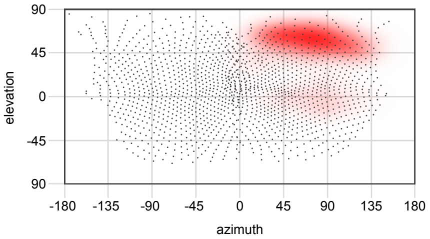

ER3d_d: 720575940614073315

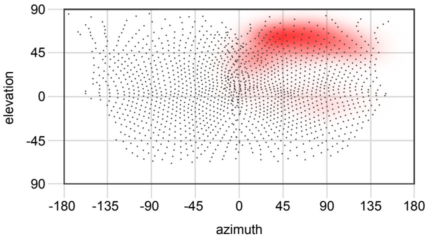

ER3d_d: 720575940619829765

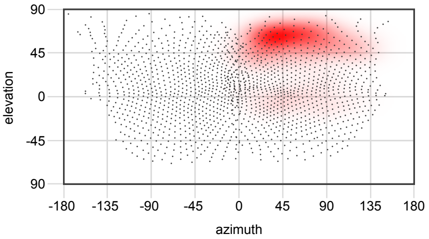

ER3m: 720575940605837024

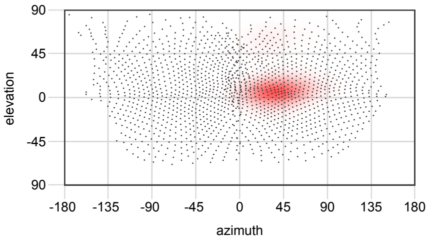

ER3m: 720575940609621003

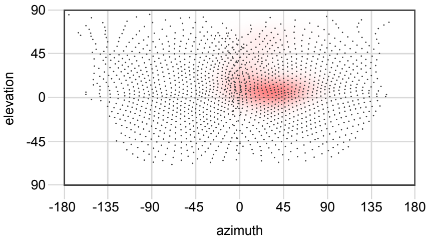

ER3m: 720575940610263665

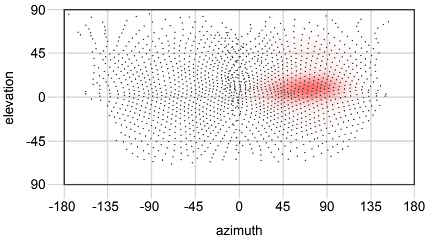

ER3m: 720575940613676402

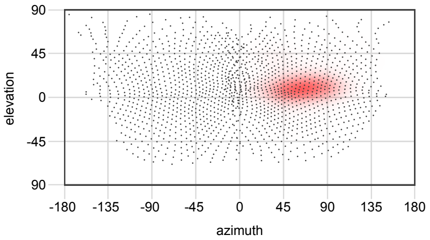

ER3m: 720575940616441410

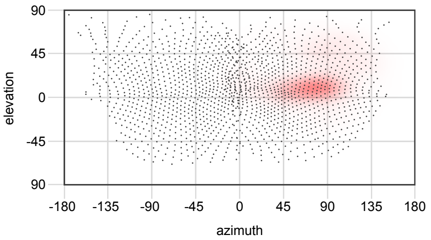

ER3m: 720575940618045986

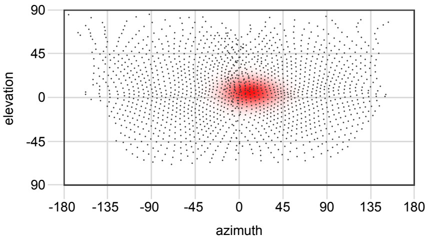

ER3m: 720575940630658807

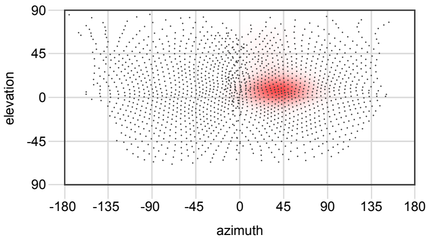

ER3p_ab: 720575940613700674

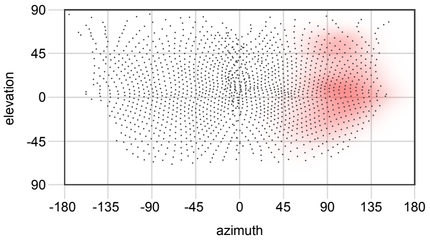

ER3p_ab: 720575940618732782

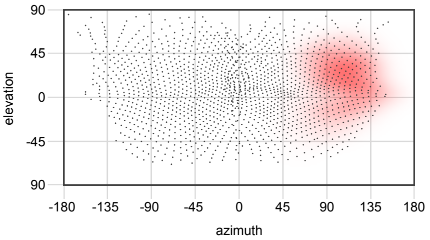

ER3p_ab: 720575940619477232

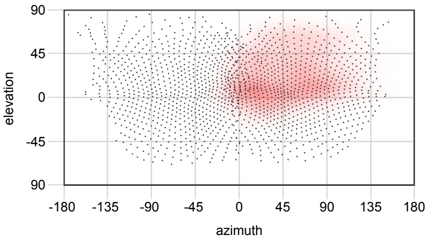

ER3p_ab: 720575940619957786

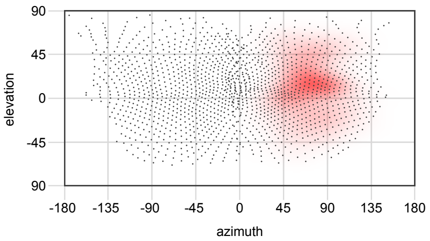

ER3p_ab: 720575940622431305

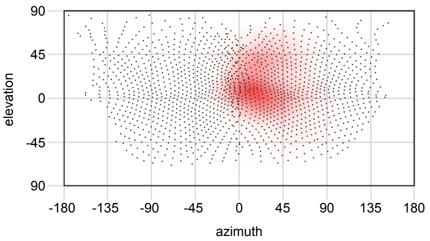

ER3p_ab: 720575940640424589

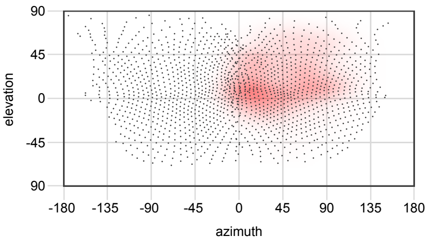

ER3p_ab: 720575940640424845

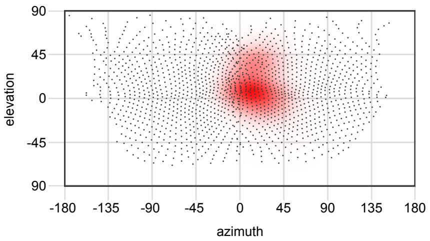

ER3p_ab: 720575940647731833

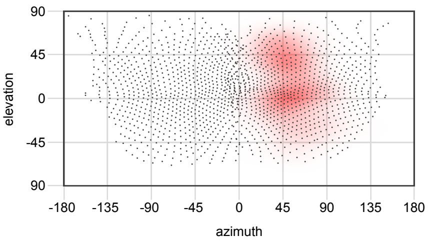

ER3w_ab: 720575940610505701

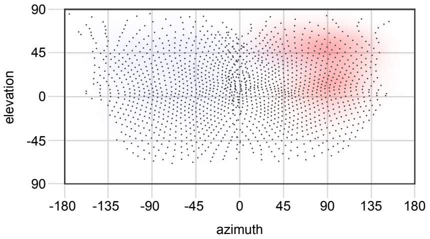

ER3w_ab: 720575940611856760

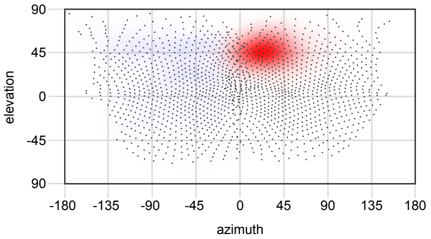

ER3w_ab: 720575940612979090

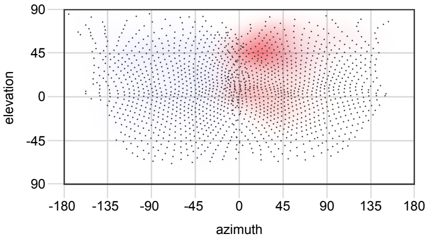

ER3w_ab: 720575940616025378

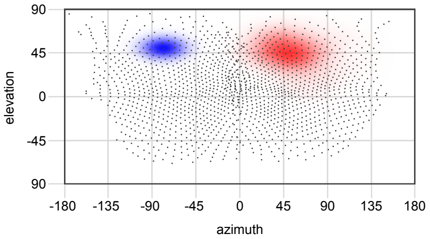

ER3w_ab: 720575940618306469

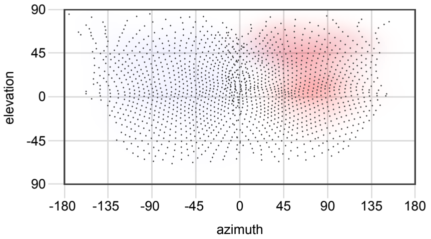

ER3w_ab: 720575940621066776

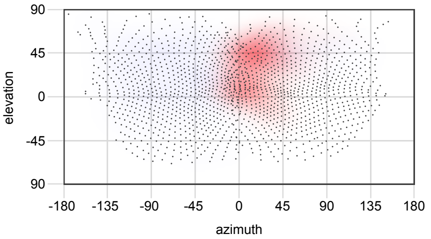

ER3w_ab: 720575940624470812

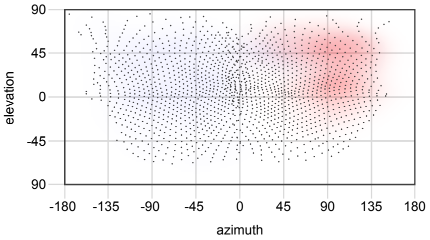

ER3w_ab: 720575940626363654

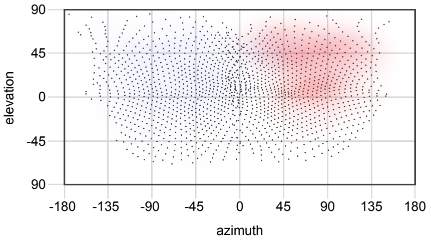

ER3w_ab: 720575940626421578

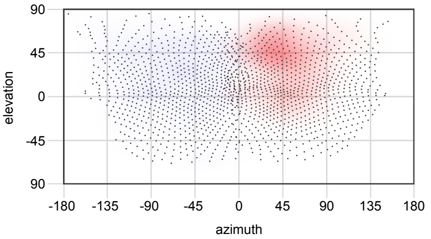

ER3w_ab: 720575940631248865

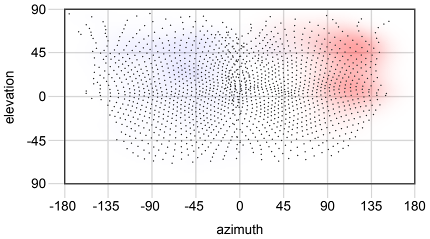

ER3w_ab: 720575940637681267

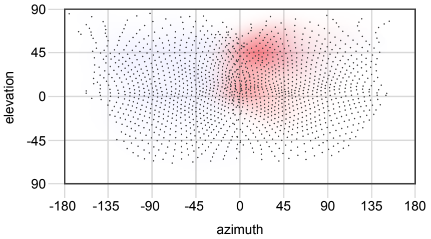

ER3w_ab: 720575940640760179

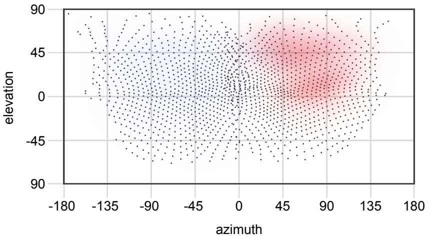

ER3w_ab: 720575940653326326

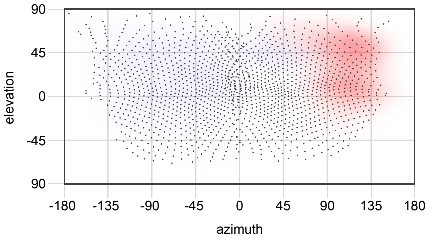

ER4d: 720575940603964774

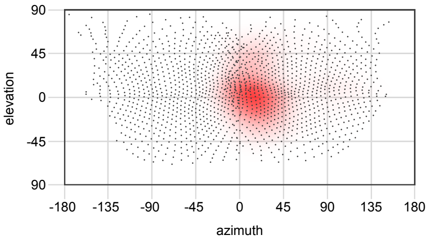

ER4d: 720575940604842924

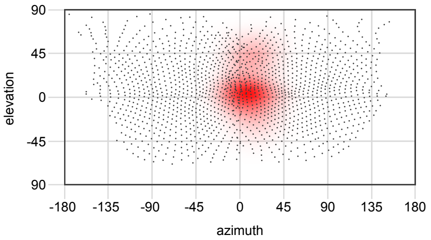

ER4d: 720575940606102729

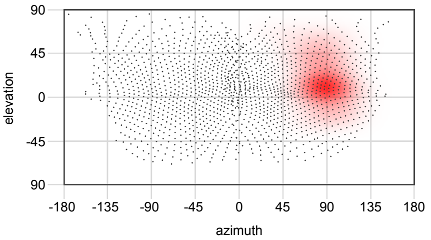

ER4d: 720575940623219463

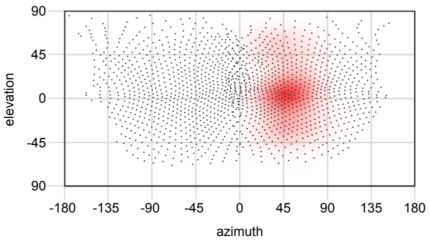

ER4d: 720575940624373837

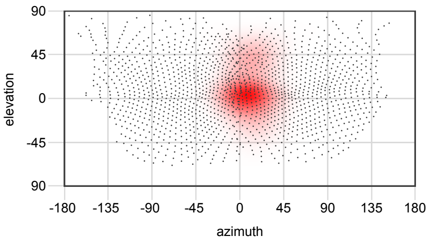

ER4d: 720575940625511762

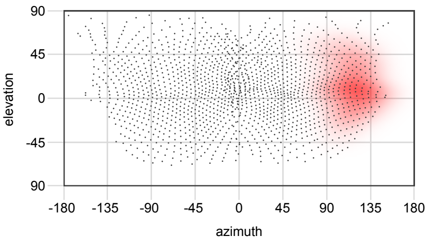

ER4d: 720575940627890072

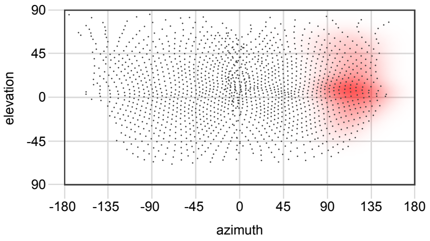

ER4d: 720575940628014488

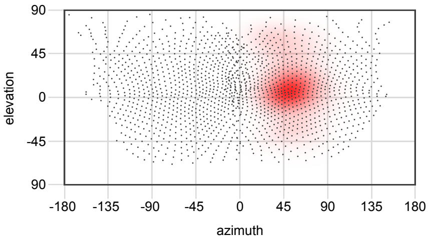

ER4d: 720575940629213639

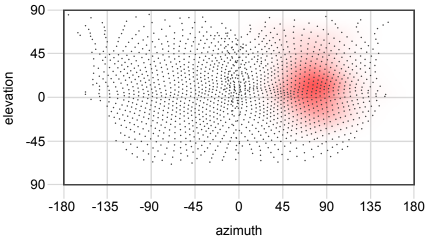

ER4d: 720575940629720848

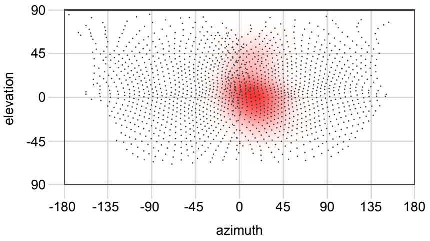

ER4d: 720575940635152153

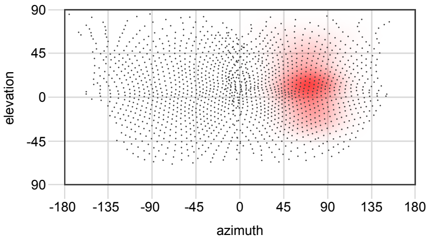

ER4d: 720575940642877088

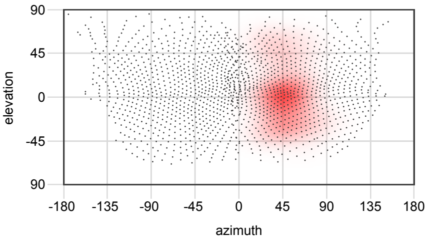

ER4d: 720575940643915300

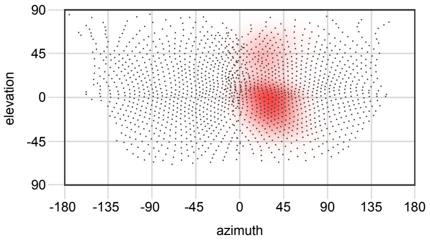

ER4m: 720575940615865238

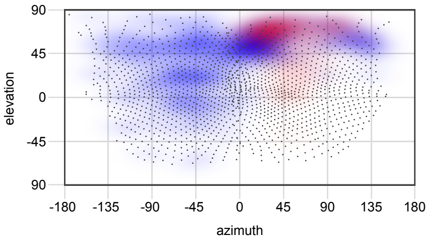

ER4m: 720575940619436342

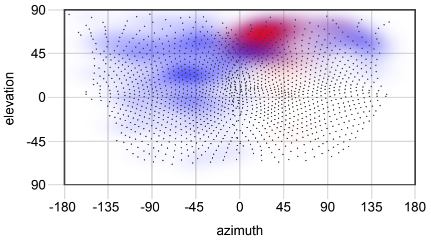

ER4m: 720575940631093303

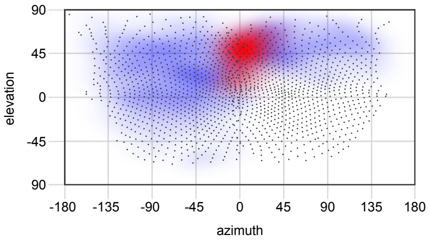

ER4m: 720575940634096339

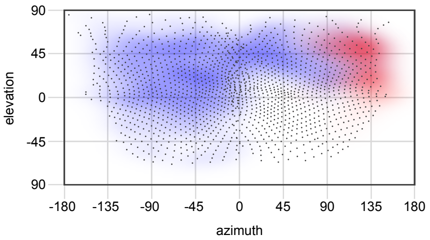

ER4m: 720575940635759002

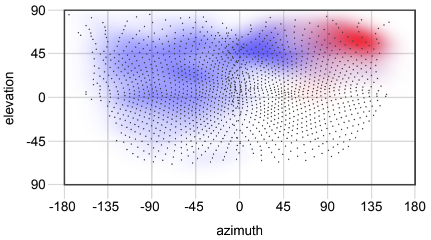

ER5: 720575940615650022

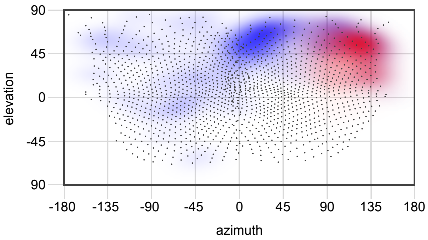

ER5: 720575940620169851

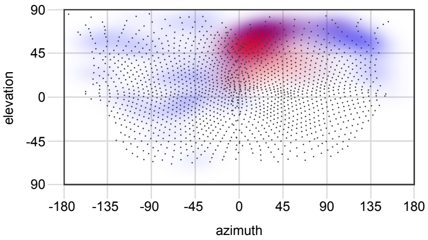

ER5: 720575940624837453

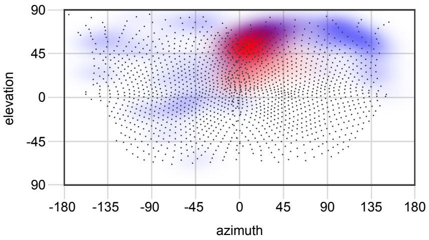

ER5: 720575940625829192

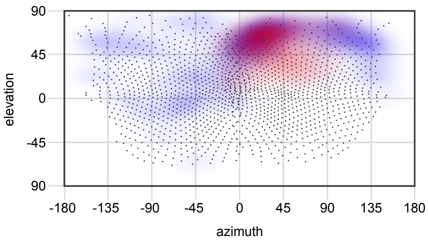

ER5: 720575940626610960

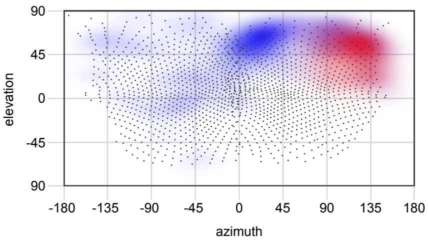

ER5: 720575940628772345

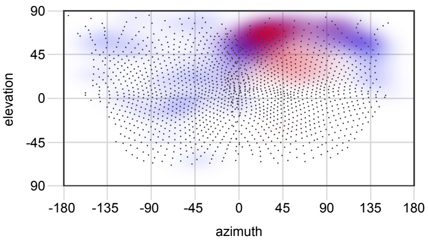

ER5: 720575940631416057

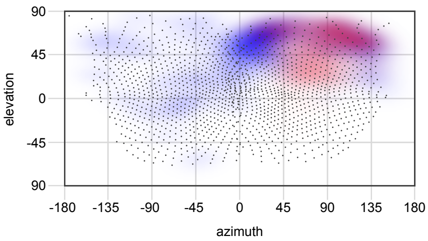

ER5: 720575940632366879

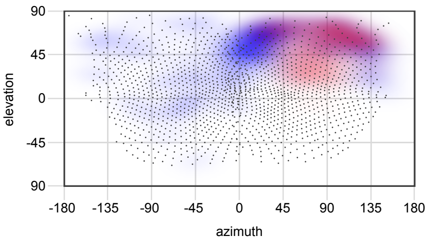

ER5: 720575940637126222

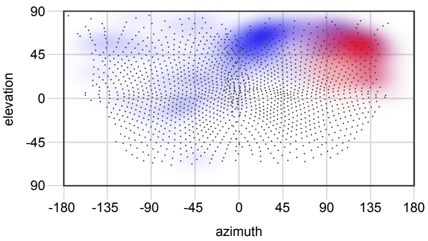

ER5: 720575940638169456

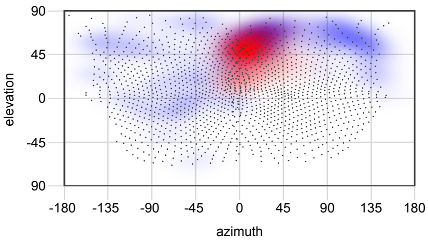

ER5: 720575940652809889

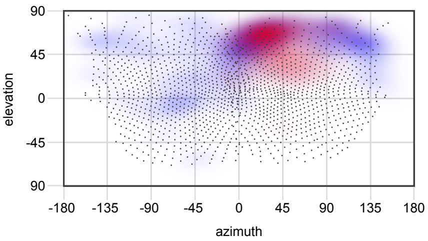

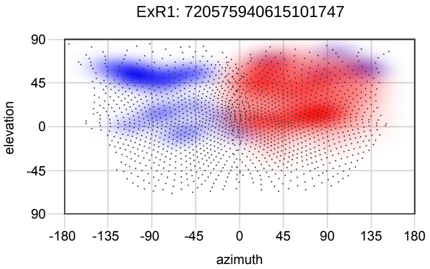
ExR1: 720575940615101747
90
45
0
-45
90
elevation
-180
-135
-90
-45
0
45
90
135
180
azimuth

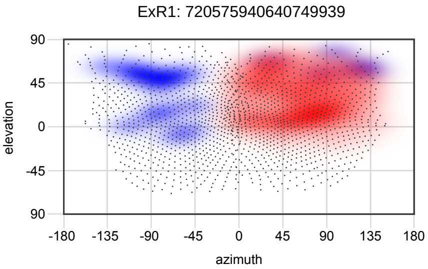
ExR1: 720575940640749939
90
45
0
-45
90
elevation
-180
-135
-90
-45
0
45
90
135
180
azimuth
