# Supplementary material for: Connectomic reconstruction predicts visual features used for navigation
Source: Nature. 2024 Oct 2;634(8032):181–90. doi: 10.1038/s41586-024-07967-z (PMC11446847; doi:10.1038/s41586-024-07967-z)
Supplement: Supplementary file 7 — Key resources table. [file 41586_2024_7967_MOESM7_ESM.pdf]

Supplementary Table 2

Key Resources				
**Reagent type (species) or resource**	**Designation**	**Source or reference**	**Identifiers**	**Additional information**
Software, algorithm	Python 3	http://www.python.org	RRID:SCR_008394	
Software, algorithm	pymaid	Jarrell et al. [75] and Schlegel et al. [76]; https://github.com/schlegelp/PyMaid		python library
Software, algorithm	FAFBseg	Schlegel et al. [46] and Dorkenwald et al. [44]; https://github.com/flyconnectome/fafbseg-py		python library
Software, algorithm	R	https://www.r-project.org/		
Software, algorithm	R Studio	https://www.rstudio.com/		
Software, algorithm	Natverse	Bates et al. 2020 [77]; https://natverse.org	DOI: 10.7554/eLife.53350	collection of R packages for neuroanatomical analysis
Software, algorithm	Tidyverse	https://www.tidyverse.org/		R package
Software, algorithm	alphashape3d	https://CRAN.R-project.org/package=alphashape3d		R package
other	FAFB	Zheng et al. 2018 [17]; https://v2.virtualflybrain.org/		EM data set
other	FlyWire	Dorkenwald et al. 2022 [29]; https://flywire.ai/		EM auto-segmentation

Fly line	Type	Source
MeTu split-Gal4 lines and original image data are available via https://splitgal4.janelia.org/cgi-bin/splitgal4.cgi. Some lines are not currently maintained as stocks but could be reconstructed from the AD and DBD hemidrivers.		
SS00385 (Fig. 2g-i)	MeTu1	This study - https://splitgal4.janelia.org/cgi-bin/view_splitgal4_imagery.cgi?line=SS00385
SS00336 (Fig. EDF5f-g)	MeTu2a	This study - https://splitgal4.janelia.org/cgi-bin/view_splitgal4_imagery.cgi?line=SS00336
SS03744 (Fig. EDF5h-i)	MeTu2b	This study - https://splitgal4.janelia.org/cgi-bin/view_splitgal4_imagery.cgi?line=SS03744
SS00988 (Fig. EDF6f-g)	MeTu3b	This study - https://flylight-raw.janelia.org/cgi-bin/view_raw_imagery.cgi?line=SS00988
SS03719 (Fig. EDF8f-g)	MeTu4a	This study - https://splitgal4.janelia.org/cgi-bin/view_splitgal4_imagery.cgi?line=SS03719

SS23880 (Fig. EDF8h-i)	MeTu4d	This study - https://splitgal4.janelia.org/cgi-bin/view_splitgal4_imagery.cgi?line=SS23880
SS04147 (Fig. 5)	ER4d	w[1118]; P{y[+t7.7] w[+mC]=R72D06-p65.AD}attP40; P{y[+t7.7] w[+mC]=R72D01-GAL4.DBD}attP2 (BDSC# 71126 & 69696)
VT059775 (Fig. 5)	ER2	w[1118]; P{y[+t7.7] w[+mC]=VT059775-p65.AD}attP40; P{y[+t7.7] w[+mC]=VT059775-GAL4.DBD}attP2 (BDSC# 73221 & 73951)
jGCaMP8f (Fig. 5)	GECI	w+; PBac{y[+mDint2] w[+mC]=20XUAS-IVS-jGCaMP8f}VK00005 (white-eyed parents are BDSC# 92587)

75 Jarrell, T. A. *et al.* The connectome of a decision-making neural network. *science* **337**, 437-444 (2012).

76 Schlegel, P. *et al.* Synaptic transmission parallels neuromodulation in a central food-intake circuit. *eLife* **5**, e16799 (2016).

77 Bates, A. S. *et al.* The natverse, a versatile toolbox for combining and analysing neuroanatomical data. *eLife* **9**, e53350 (2020).
